# Prehistoric pottery styles of the Jutland Peninsula and their effect on human attention

**DOI:** 10.1057/s41599-026-08601-1

**Published:** 2026-08-15

**Authors:** Lizzie Scholtus, Bruno Vindrola-Padrós, Niels Nørkjær Johannsen, Johannes Müller

**Affiliations:** 1 Institute of Pre- and Protohistoric Archaeology, Kiel University, Kiel, Germany; 2 Department of Archaeology and Heritage Studies, School of Culture and Society, Aarhus University, Aarhus, Denmark; 3 Moesgaard Museum, Aarhus, Denmark

**Keywords:** Anthropology, Psychology, Psychology

## Abstract

This paper examines how long-term transformations in pottery styles from the Jutland Peninsula (Denmark) may relate to patterns of visual attention between 4600 and 1 BCE. Free-viewing eye-tracking experiments were designed to record participants’ engagement with different prehistoric ceramic vessels from different chronological periods. Comparison with eye-tracking data from central Germany suggests that the prehistoric pottery styles from both regions are associated with broadly comparable attentional trajectories when examined through absolute chronology. These similarities are more consistent with periods characterised by supra-regional networks of interaction, exchange, and stylistic transmission linking both regions through time rather than to parallel archaeological periodisations. Periods characterised by more elaborate decorative traditions tend to show higher attentional engagement, whereas later phases marked by reduced decorative investment co-occur with an increasing prominence of other material categories, particularly metal artefacts, within broader systems of symbolic and social expression. More broadly, the study illustrates how eye-tracking approaches can be used to explore relationships between perceptual engagement, stylistic variation, and long-term material transformations in prehistoric societies.

## Introduction

During much of prehistory, the Jutland Peninsula and more generally southern Scandinavia, have often been regarded as deeply influenced by, yet sometimes peripheral to, developments in other parts of the European continent, particularly central Europe. Consequently, some of the effects of these developments were frequently felt at a different pace and in distinct forms (Herschend, 2018). Such influences are evident in economic activities (e.g. agriculture), mortuary practices, and the built environment, including burial mound traditions and house constructions, but also in locally produced material culture, such as hammer axes, bronze octagonal sword hilts, and Tumulus and Lusatian-like pottery styles (Jensen, 2013, 124; Thrane, 2013; Bech and Rasmussen, 2018).

Material culture is typically approached through typologies, styles, decorative systems, and production techniques. However, it can also be examined from a perceptual and cognitive perspective. The stylistic attributes of objects (such as shape, colour, and luminance) possess visual properties that influence visual attention, that is, the selective allocation of attention to specific elements within a visual scene. In this respect, decorative patterns do not only convey symbolic or cultural information, but also shape visual exploration, as reflected in eye movements such as fixations and saccades directed towards salient or information-rich areas (Parr et al., 2021). Variations in these properties can, in turn, influence the cognitive load associated with visual exploration, understood here as the mental effort required to interpret visual information. From this perspective, stylistic transformations in material culture are not only reflections of socio-historical change, but also transformations in the way objects structure perceptual engagement. This raises the question of whether broader cultural dynamics may have influenced not only what was produced, but also the visual-attentional properties of material culture.

Pottery styles provide a particularly valuable medium for addressing this question, as they are not only strong chronological markers, but are also highly responsive to broader socio-historical transformations. More importantly, the study of their stylistic attributes can yield insights into visual cognition and perceptual engagement. In ceramic vessels, decorative patterns, surface organisation and contrast in form or colour can influence eye movements and visual attention. These features influence both the distribution and duration of fixations, thereby shaping patterns of visual exploration and are associated with variations in cognitive load. Eye-tracking methods make it possible to capture these processes empirically by recording viewers’ eye movements during the observation of objects. Recent studies further support this theory, showing that ceramic stylistic features, most notably decorative patterns, can significantly influence attentional patterns and impose varying demands on visual processing, that is, cognitive load (Criado-Boado et al., 2019; 2023; Vindrola-Padrós et al., 2026).

When situated within their chronological and socio-historical contexts, eye-tracking measures offer a means of investigating how patterns of visual attention may vary over time (Vindrola-Padrós et al., 2025; Vindrol-Padrós et al., 2026). This approach has been applied to a five-thousand-year sequence of pottery styles from central Germany. Its results suggested that shifts in patterns of attention coincided with socio-historical dynamics that shaped pottery design. These included the adoption of alternative media for symbolic expression (e.g. copper and bronze artefacts), the intermingling of supra-regional and local pottery networks, processes of specialisation and standardisation of pottery associated with growing social differentiation, and technological innovations such as the potter’s wheel (Vindrola-Padrós et al., 2026). The culmination of these processes was associated with a gradual decline in the production of attention-grabbing or visually captivating ceramics styles. This case study provides a valuable framework for assessing whether pottery designs influenced by similar socio-historical processes may have influenced the attentional visual responses of pottery traditions beyond central Europe.

**Fig. 1:**
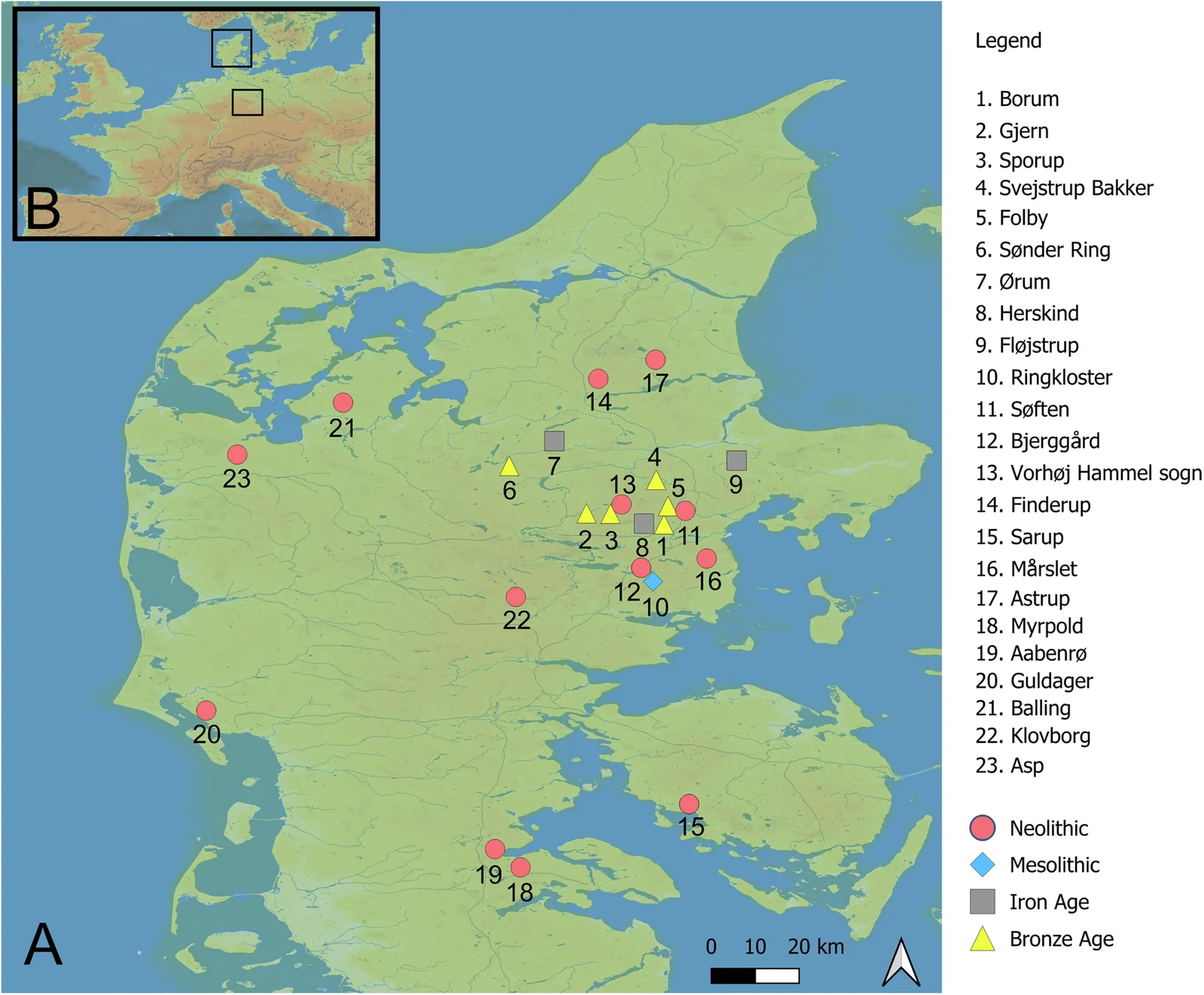
Location of the Jutland study area and provenance of the ceramic vessels included in the experiment (**A**), together with the central German comparative region discussed throughout the study (**B**). (maps by L. Neiroba; base map: Mapzen Global Terrain and OpenStreetMap contributors and others, Rendering © EOX).

Building on this framework, the present study investigates whether stylistic designs in the Jutland region (Denmark) are associated with visual effects comparable to those observed in central Europe. More specifically, it examines whether stylistic developments in this northern context are associated with similar modulations in visual attention, or whether they follow a distinct trajectory. Beyond this comparison, the study explores how these variations might relate to broader socio-historical processes and to conventional archaeological periodisation. While such periodisations are grounded in major socio-historical transformations, they do not unfold synchronously across regions. For instance, the Early Neolithic in central Europe developed more than a millennium before than in northern Europe. In this respect, we further consider whether similarities in patterns of attention between Jutland and central Germany may reflect shared social dynamics, including networks of interaction and exchange linking these regions (Fig. 1). The present study focuses primarily on general perceptual responses to stylistic features, rather than directly reconstructing the intentions of producers or culturally specific interpretations by past consumers.

To address this question, we conducted a free-viewing eye-tracking experiment at the Moesgaard Museum in Højbjerg (Denmark), using a diachronic corpus of pottery vessels spanning from 4600 to 1 BCE. By analysing fixation duration and pupil diameter, which are commonly used as measures of visual processing demands, the study investigates whether stylistic transformations in pottery are associated with changes in patterns of visual attention through time and whether comparable trajectories can be identified between northern Jutland and central Germany.

## Socio-cultural development and pottery styles

This section contextualises the main pottery styles developed in the Jutland region (Denmark) in relation to the main developments in settlement organisation, economy, mortuary practices, craftwork production and pottery tradition. Given pottery appeared during the Final Mesolithic, this period serves as the point of departure for the following overview.

### Final Mesolithic

When pottery was first introduced in Jutland around 4800–4700 BCE (Andersen, 2009; Andersen, 2011; Povlsen, 2013), the Mesolithic in Denmark was entering its last stage before the introduction of agriculture. Final Mesolithic populations in Jutland were hunter-gatherer-fishers inhabiting ‘semi-permanent’ settlements along coastal and freshwater areas (Andersen, 1995a), which have been described as sedentary communities. Settlements typically consisted of artefact scatters (mainly bone, ceramic, lithic and wooden) and a few domestic dwelling structures. Large kitchen middens were also characteristically accumulated near shellfish banks (Andersen, 2000). Burial practices and grave goods varied considerably during this period. Pottery was not part of funerary practices, and bodies could be cremated, inhumated, or placed in boat burials (Skaarup and Grøn, 2004; Jensen, 2013, 113). While pottery was introduced during this period, it is mostly other types of crafts that appear to have held particular social or symbolic significance. Of note are the highly decorated deer antler axes, which contain a significant number of abstract patterns, including geometric motifs such as parallel lines, zig-zag lines, dots, cross hatches, as well as ornamented amber pieces, often in animal shapes (Andersen, 1980; Andersen, 1995a), and wooden implements like paddles, with similar decorative patterns (Andersen, 2013). Central European influence is attested in the distribution of shoe-last and greenstone axes in Jutland (Klassen, 2004). These artefacts suggest that visually elaborate decorative traditions already formed an important component of material.

There are several hypotheses regarding the introduction of pottery in the region, most of them include the influence of Baltic hunter-gatherer pointed-base traditions, and some inspiration from the central European Neolithic pottery (Louwe Kooijmans, 2010; Andersen, 2011; Dumpe et al., 2011). Ertebølle pottery (4800–3900 BCE) consisted mainly of large pointed-based vessels (Prangsgaard, 1992; Andersen, 2011, 199; Povlsen, 2013, 147) and smaller oval bowls, generally considered to be blubber lamps. Decorative investment remained relatively limited and typically consisted of fingernail impressions placed below vessel rims or directly on bowl surfaces (Andersen, 2011). Ertebølle pottery was multifunctional, used for food processing and cooking (Craig et al., 2007; Craig et al., 2011; Papakosta et al., 2019).

### Neolithic

The beginning of the Neolithic period (4000–3300 BCE) in the Jutland region, as in southern Scandinavia, was marked by a gradual transition to agricultural economies and associated technologies. aDNA evidence nevertheless suggests that migrating populations associated with Funnel-beaker ceramics played a significant role in this process (Allentoft et al., 2024). Similar to long-standing traditions in central Europe, settlement systems consisted primarily of farmsteads and hamlets located further inland compared to the Late Mesolithic (Boas, 2019; Nielsen, 2019, 898; Sarauw, 2019). During the later Early Neolithic, causewayed enclosures emerged as important communal and ritual spaces (Andersen, 1997; Müller, 2010, 12; Madsen, 2024), while mortuary practices diversified, including both continuity with Mesolithic traditions and the emergence of earthen long barrows associated with Funnel Beaker pottery (Madsen, 1993; Sjögren and Fischer, 2023), paralleling developments in central Europe (i.e. *Baalberg* mounds). These developments indicate increasing social differentiation and more formalised collective practices, without clear evidence for strong hierarchical structures. Flint mining remained of major importance throughout the Neolithic, while amber continued to hold social and symbolic importance, with highly elaborate necklaces, sometimes as part of votive offerings (Ramstad et al., 2015; Earle et al., 2023). The first copper items also appeared in Jutland during this period, including spiral arm rings, flat axe blades and plates, mostly found in graves or hoards (Jensen, 2013, 222; Gebauer et al., 2021; Brozio et al., 2023).

**Fig. 2:**
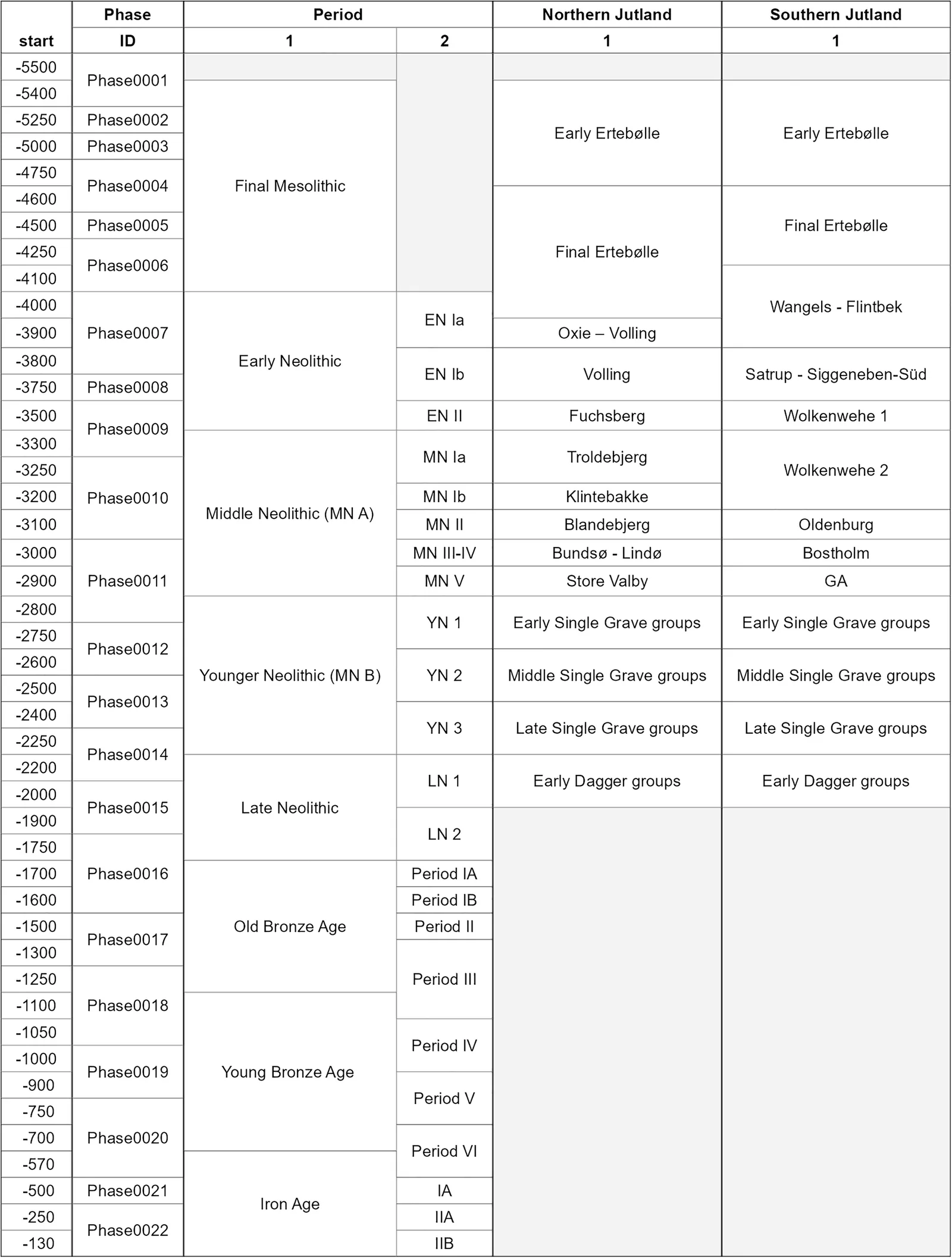
Typo-chronological chart of northern and southern Jutland (Hübner, 2005; Jensen, 2013; Madsen, 2020; Martens, 1992; Müller and Peterson, 2015).

Pottery during the Early (EN) and Middle Neolithic (MN) in Jutland was associated with a series of richly decorated and regionally varied styles (Fig. 2) that nevertheless shared broad similarities across northern Germany and neighbouring regions (Müller, 1918; Madsen and Petersen, 1983, 115; Müller and Peterson, 2015), commonly grouped under the Funnel-necked Beaker ‘Culture’ or Complex (FBC). During the Early Neolithic, specific decorative schemes became associated with particular types of pottery, such as lugged beakers in long barrows. The earliest styles, including Volling in northern Jutland and Oxie further east, were followed by increasingly supra-regional decorative traditions from around 3600 BCE onwards (Müller, 2011, 14–15). At the transition to EN II, a marked shift occurred with the emergence of Fuchsberg styles, characterised by a strong intensification of ornamentation that continued throughout much of the Middle Neolithic (Madsen, 1982, 201; Madsen, 1997, 78; Müller, 2011, 17). Chevron motifs became especially prominent, while decorated bowls and lugged beakers also appeared in a wider range of contexts, including enclosures and ditches (Andersen, 1997).

During the Middle Neolithic (3300–2800 BCE), residential sites expanded and became more permanent (Madsen, 1982, 207), while causewayed enclosures fell out of use shortly after the beginning of the period (Madsen, 1988; KIassen, 2014). At the same time, the construction of large communal megalithic monuments and labour-intensive earthworks suggests increasing social differentiation and more formalised collective organisation (Müller, 2010; Madsen, 2024, 409). Megalithic graves peaked in importance and frequency (Ebbesen, 1985, 40; Jensen, 2013, 185), and passage graves linked to FBC pottery became widespread. Around 3100 BCE, in contrast, megalithic tomb building ceases and burials mostly re-used these structures (Madsen, 1997, 77). This period also saw increasing interaction with central European groups such as the Globular Amphora Culture, reflected in the appearance of cattle burials and changes in subsistence strategies (Johannsen and Laursen, 2010; Madsen, 2024, 479; Johannsen and Borup in press).

Highly decorated pottery forms, particularly bowls and lugged beakers deposited at tomb facades or causewayed enclosures, appear to have held supra-regional significance within communal and ritual practices (Madsen, 1997, 79). At the same time, ceramic traditions became increasingly diversified and regionally differentiated, with a growing formalisation of decorative schemes and vessel forms linked to expressions of cultural affiliation and identity (Müller and Peterson, 2015, 580; Madsen, 2024). In Jutland, these developments are reflected in a sequence of pottery styles primarily distinguished by their decorative patterns (Müller, 2011, 16–17). Early Middle Neolithic styles continued the highly decorated Fuchsberg tradition, characterised by ladders and banded chevron patterns. Over time, however, decorative compositions became progressively simplified, culminating in the Store Valby style, whose largely undecorated and thick-walled vessels marked a ‘decline’ in stylistic designs (Müller, 1918; Brozio et al., 2019; Madsen, 2024, 470).

The Younger Neolithic (2800–2200 BCE) is associated with the widespread distribution of the so-called Single Grave Culture (SGC), representing a major cultural transformation in Jutland strongly influenced, both genetically and culturally, by Corded Ware populations from central Europe (Kristiansen, 1989; Nielsen and Johannsen, 2023; Allentoft et al., 2024). While settlement systems, mainly known from the later part of the period, relied in part on farmsteads and hamlets (Simonsen, 2017, 62; Simonsen, 2018), mortuary practices shifted markedly towards individual burials placed beneath earthen mounds, in contrast to the collective traditions associated with the FBC (Hübner, 2005). Grave goods commonly included flint axes, amber ornaments, beads, stone battle axes and increasingly also pottery as the period progressed, reflecting new forms of symbolic display and social distinction (Tornberg and Vandkilde, 2025, 38). At the same time, animal husbandry became increasingly important within the economy, accompanied by expanded land clearance for pasture (Andersen, 1995b, 128; Jensen, 2013, 239).

Pottery traditions changed substantially during the Younger Neolithic, with the introduction of new production techniques (Engberg, 1986; Madsen, 2024, 479) and decorative styles influenced by Corded Ware traditions from central Europe (Hübner, 2005, 726; Jensen, 2013, 258–259). Early SGC pottery displayed a greater variability in form and decoration, including characteristic herringbone decoration (Hübner, 2005, 729). Over time, however, corded ornaments lost importance, while curved beakers with linear and zoned decorations became more common (Hübner, 2005, 731). In the later phase of SGC, straight-walled and flat-bottomed beakers progressively dominated ceramic assemblages (Hübner, 2005, 734; Jensen, 2013, 257), alongside the earliest appearance of Bell Beaker forms in Jutland (Simonsen, 1986; Vandkilde, 2005; Sarauw, 2007, 36).

The Late Neolithic (2200–1700 BCE) in Jutland was characterised by the continued influence of the Bell Beaker phenomenon in both pottery design and burial customs, alongside increasing integration into the metallurgical networks associated with the *Unètice* culture. Settlements systems largely continued earlier tradition, although houses increased markedly in size during the transition between LN I and LN II (Sarauw, 2006; Nielsen, 2019, 906). Mortuary practices remained focused on individual burials under mounds while stone cists and gallery graves became increasingly common throughout the period (Simonsen, 1978; Vandkilde, 2005, 22).

During the LN I, Jutland became a major centre for the production and distribution of flint daggers (Becker, 1993; Sarauw, 2006; Jensen, 2013, 270–275), while Bell Beakers and highly ornamented beakers remained widespread across the region (Müller and Peterson, 2015, 581). At the same time, large supra-regional exchange networks played an increasingly important role in socio-economic developments. During LN II, connections with central Germany facilitated the circulation of bronze objects, gold artefacts, and raw materials for local metal production (Vandkilde, 1996, 299; Nørgaard et al., 2021). By this stage, local metallurgy was fully established (Nørgaard et al., 2019), and bronze objects became increasingly important both in practical activities and as indicators of prestige and wealth (Vandkilde, 1996, 305).

Pottery traditions also changed substantially throughout the Late Neolithic. During LN I, Bell Beakers and other ornamented vessels remained common across Jutland, reflecting continuing investment in visually elaborate ceramic styles. By LN II, however, Bell Beakers declined and ceramic vessels became less frequent overall (Jensen, 2013, 277). This reduction in decorative investment coincided with the growing importance of metallurgy and the increasing social significance of bronze artefacts, while amber continued to occupy an important role within systems of ornamentation and exchange (Simonsen, 2017; Vandkilde et al., 2024).

### Bronze Age

The transition to the Bronze Age in Jutland was a gradual process, with several traditions continuing from preceding periods, including longhouse architecture (Thrane, 2013). Nevertheless, major transformations occurred in settlement organisation, mortuary practices, and craft production. The period is conventionally divided into an Early or Older Bronze Age (1700–1100 BCE; Montelius, 1885, Periods I–III) and a Late or Younger Bronze Age (1100–500 BCE; Periods IV–VI; Hornstrup et al., 2012), a distinction that is particularly reflected in changes in pottery traditions.

Settlement systems became increasingly dominated by dispersed farmsteads of varying size, while longhouses shifted from two-aisled to three-aisled constructions during Period II, marking a major architectural transformation (Jensen, 1982, 146; Nielsen, 1999). Mortuary practices centred on barrow burials with oak-coffin inhumations, before cremation became increasingly widespread from Period III onwards. Variations in burial size and grave goods, particularly bronze weapons and ornaments, indicate growing social differentiation during the Early Bronze Age (Randsborg, 1974; Jensen, 1993, 134; Kristensen, 2018).

Metallurgy became the dominant prestige craft of the Bronze Age and played a central role in both ritual and socio-economic life (Earle, 2004, 120). Tin bronze raw materials were imported through extensive exchange networks linking southern Scandinavia with central Europe (Vandkilde, 1993, p. 147), while evidence from hoards and workshops suggests increasing craft specialisation during the Early Bronze Age (Kristiansen, 1987; Johannsen, 2015, 79). During Period IB, both the quantity and typological diversity of metal artefacts increased markedly (Vandkilde, 1996), partly through the importation of finished objects such as Apa-, octagonal-, and flange-hilted swords (Bech and Rasmussen, 2018, p.l 76; Quillfeldt, 1996). Metal objects such as swords, axes, and spearheads were often visually elaborate, displaying geometric, spiral, concentric, and radial motifs that formed part of broader decorative traditions shared across Bronze Age Europe (Vandkilde, 1996; Thrane, 2013).

Pottery during the Early Bronze Age appears to have played a more limited role in ritual and symbolic practices and was primarily associated with domestic contexts (Rasmussen, 1994; Jensen, 2013, 349). Ceramic vessels were generally plain, with simple forms and only rare decoration (Kristensen, 2018). Some vessel types seem to have been more closely associated with mortuary contexts and are largely absent from settlement assemblages (Lolk, 2009; Kristensen, 2018). During Period II, influences from Tumulus and Lusatian traditions became visible through new vessel forms and smoother surface treatments (Thrane, 2013, 762). Compared with earlier Neolithic traditions, pottery decoration nevertheless appears more limited overall, particularly when contrasted with the visual elaboration of contemporary metalwork.

During the Late Bronze Age (1100–500 BCE), farmsteads remained the main social and economic units in Jutland, although increasingly integrated within broader exchange networks (Earle et al., 2022), as reflected for example in the construction of road systems and intensified circulation of goods (Jensen, 2002, 344). At the same time, burial practices underwent significant transformations, with cremation becoming dominant and urns progressively replacing earlier burial containers during Periods IV–VI (Thrane, 1984; Jensen, 1997). Metal grave goods became less common in funerary contexts, while bronze artefacts increasingly accumulated in hoards and depositional contexts instead (Vandkilde, 1996; Kneisel, 2013).

Bronze production also expanded considerably during this period. New casting techniques enabled the manufacture of more complex objects such as lurs (Jensen, 2002, 365; Nørgaard, 2018), while ornamented metal weapons and tools proliferated in Period V. Around 800 BC, decorative styles shifted away from the spiral motifs characteristic of the Early Bronze Age towards more varied geometric and curvilinear compositions, including wavy bands, concentric circles, and radial motifs (Jensen, 2002, p. 365, 368).

Pottery traditions also changed substantially during the Late Bronze Age. In contrast to the largely domestic and rarely decorated ceramics of the Early Bronze Age, Late Bronze Age pottery became increasingly linked to broader pan-European traditions associated with cremation burials, particularly those connected to the Urnfield and Lusatian spheres. Ceramic production and vessel forms appear more standardised overall, although some visually distinctive forms emerged, most notably the so-called “face urns”, whose surfaces displayed stylised facial features ranging from simple eyes to more elaborate brows and mouths (Lolk, 2009; Thrane, 2013, 762; Kneisel, 2019). Around 800 BCE (Period V), pottery styles also show growing influences from northern and central German regions (Jensen, 2002, 363).

### Pre-Roman Iron Age

The pre-Roman Iron Age (500–1 BCE; PRIA) can be subdivided into Early (500–250 BCE) and Late PRIA (250–1 BCE; phases I and IIA/IIB, respectively, in Martens, 1996). While many aspects of daily life continued from the Late Bronze Age, the presence of artefacts associated with late Hallstatt and La Tène traditions nevertheless marks an important shift in Jutland’s connections with central Europe exchange networks (Herschend, 2018).

Settlement organisation remained broadly continuous with the preceding period, although expansion is visible in both western and northern Jutland (e.g. Mathiassen, 1948; Jensen, 1982, 204). Farmsteads increasingly clustered into village-like agglomerations, sometimes comprising dozens of longhouses (Haue, 2023), while the development of so-called “Celtic fields” reflects a growing investment in agricultural organisation and land division (Webley, 2008, 42). Mortuary evidence remains relatively scarce, but cremation burials placed in pits dominated, occasionally accompanied by urns and only limited grave goods (Martens, 1992, 108–111).

More broadly, the PRIA marks an important transformation in exchange networks and craft production. Around 500 BCE, the supra-regional circulation of bronze prestige goods that had characterised the Late Bronze Age appears to have diminished considerably (Jensen, 1982). While some iron artefacts already appeared during the latest Bronze Age phases, the PRIA marks the beginning of the extraction, smelting, and smithing of bog iron on a larger scale in Jutland, where the resource was abundant (Jensen, 1982, 228). Evidence suggests that iron production was mainly practised at the household or village level, although archaeological evidence for furnaces remains limited during the Early PRIA in Denmark.

Pottery throughout the PRIA typically consisted predominantly of undecorated flat-based large jars, and smaller beakers. In contrast to the Bronze Age, however, the chronology of the Danish PRIA is primarily based on pottery seriation derived from grave assemblages (Becker, 1961; Martens, 1992; Martens, 1996). Given the broad similarity of vessel forms across the period, chronological distinctions mainly rely on relatively subtle variations in necks, rims, and handle shapes.

During the later part of the PRIA, from around the second century BCE, La Tène influences became increasingly visible in Jutland material culture (Herschend, 2018). Settlement organisation also changed, with clearer village nucleation, larger settlements, and the appearance of enclosures such as fences and palisades around farmsteads (Martens, 2007; Webley, 2008, 26–29). Mortuary practices were also significantly transformed during this period. Cremation burials continued to dominate, but cemeteries became more numerous and grave assemblages increasingly varied. Richer burials containing weapons, imported bronze vessels, and other prestige goods point to the emergence of warrior and chieftain elites (Martens, 1992, 111–113; Jensen, 2013).

Iron production also intensified during the Late PRIA, particularly in central Jutland, where larger quantities of slag and the first evidence of furnaces have been identified (Nørbach, 1998; Jauttijärvi and Voss, 2011; Carlie, 2012). Despite this expansion, metal objects remained relatively scarce in settlement contexts in Jutland, in contrast to graves and votive deposits, where jewellery, weapons, precious metals, and imported prestige goods occur more frequently (Martens, 1992; Webley, 2008, 131–132).

Pottery traditions nevertheless remained relatively conservative. Ceramic assemblages continued to be dominated by largely undecorated vessels, although pottery appears in greater quantities in settlements during the Late PRIA (Hvass, 1985; Bech, 1987). At the same time, the first evidence for pottery kilns in Denmark dates to this period, suggesting important changes in ceramic production techniques and organisation (Hvass, 1975; Hvass, 1985; Bech, 1987; Jensen, 2013). Other materials, however, increasingly structured everyday material culture, particularly metal cauldrons and wooden tableware (Herschend, 2018, 162).

From the Final Mesolithic to the pre-Roman Iron Age, pottery traditions in Jutland underwent substantial transformations in their forms, decorative organisation, and broader socio-cultural roles. These changes were closely intertwined with wider developments in settlement organisation, mortuary practices, craft production, and supra-regional exchange networks, particularly those linking southern Scandinavia with central Europe. At the same time, the visual properties of pottery styles also shifted considerably, from the highly ornamented and regionally varied ceramics of the Neolithic to more restrained and standardised traditions characteristic of much of the Bronze and pre-Roman Iron Ages.

This study approaches stylistic variation as a set of culturally transmitted visual configurations that can differentially structure perceptual engagement through their formal properties. These properties—such as size, contrast, luminance, shape, and decorative composition—are therefore treated as variables that may influence patterns of visual attention. Building on this premise, eye-tracking methods are used to empirically assess how viewers engage with ceramic forms across long-term chronological sequences.

## Method

In order to investigate how prehistoric pottery from Jutland affects patterns of visual exploration and processing of visual information, a free-viewing eye-tracking experiment was conducted. Eye-tracking is a method widely used in psychology and cognitive science to examine visual attention, perceptual exploration and information gathering through the recording of eye movements. Visual behaviour is typically analysed through rapid eye movements known as saccades, punctuated by fixations during which visual information is primarily processed (Rayner, 2009). Measures such as fixation location, fixation duration, and scanpath organisation can therefore provide insights into how viewers visually engage with material forms.

The experimental protocol was adapted from a previous study focusing on the prehistoric pottery from central Germany (Vindrola-Padrós et al., 2026). Participants were presented with a sequence of real archaeological pots representative of the prehistoric sequence of Jutland. Each object was displayed in profile view for 15 s inside a light-controlled display box (50 × 50 × 60 cm) positioned 80 cm from the participant and equipped with LED lighting to ensure a homogeneous illumination across recordings. The vessels were divided into size categories, and their presentation order was randomised within each category rather than following a chronological sequence. Transparent stands were assigned to each size group to ensure that the object was positioned at eye level and reduce eye or head movements caused by size differences.

This protocol was established to ensure that the same recording conditions could be reproduced across different locations. However, two main differences should be noted. First, whereas the lights were turned off during the experimental sessions in central Germany, it was not possible to replicate these conditions at the Moesgaard Museum, where a light-on setting was used. Secondly, the black background employed in the initial experiment was replaced with a grey background to reduce visual contrast and minimise its effect on pupil size.

### Vessels sampling

The sampling strategy followed the protocol previously established for the central German experiment (Vindrola-Padrós et al., 2026) in order to ensure direct comparability between regions. To minimise biases linked to uneven chronological representation, typological overrepresentation, and subjective object selection, the original 5500-year sequence analysed in the previous study was divided into 22 analytical intervals of 250 years each. These intervals were created to provide a standardised comparative framework applicable across different regions.

For each phase, one complete or reconstructed vessel representative of each documented ceramic style was selected (Fig. 2). Selection was based on regional typo-chronological frameworks from Jensen (2002, 1997), Klassen (2004), Martens (1992, 1996), Müller et al. (2010), and Vandkilde (2005). Because pottery is not currently documented in Jutland before around 4800–4700 BCE, the earliest analytical phases could not be represented.

The objective of this strategy was to represent the stylistic diversity potentially present within each analytical interval rather than to establish rigid chronological boundaries between ceramic traditions. Consequently, when a ceramic style extended across multiple phases, the same vessel could be included in more than one interval in order to reflect the continued presence of that style within the chronological sequence, rather than implying that individual artefacts belonged exclusively to a single phase.

**Fig. 3:**
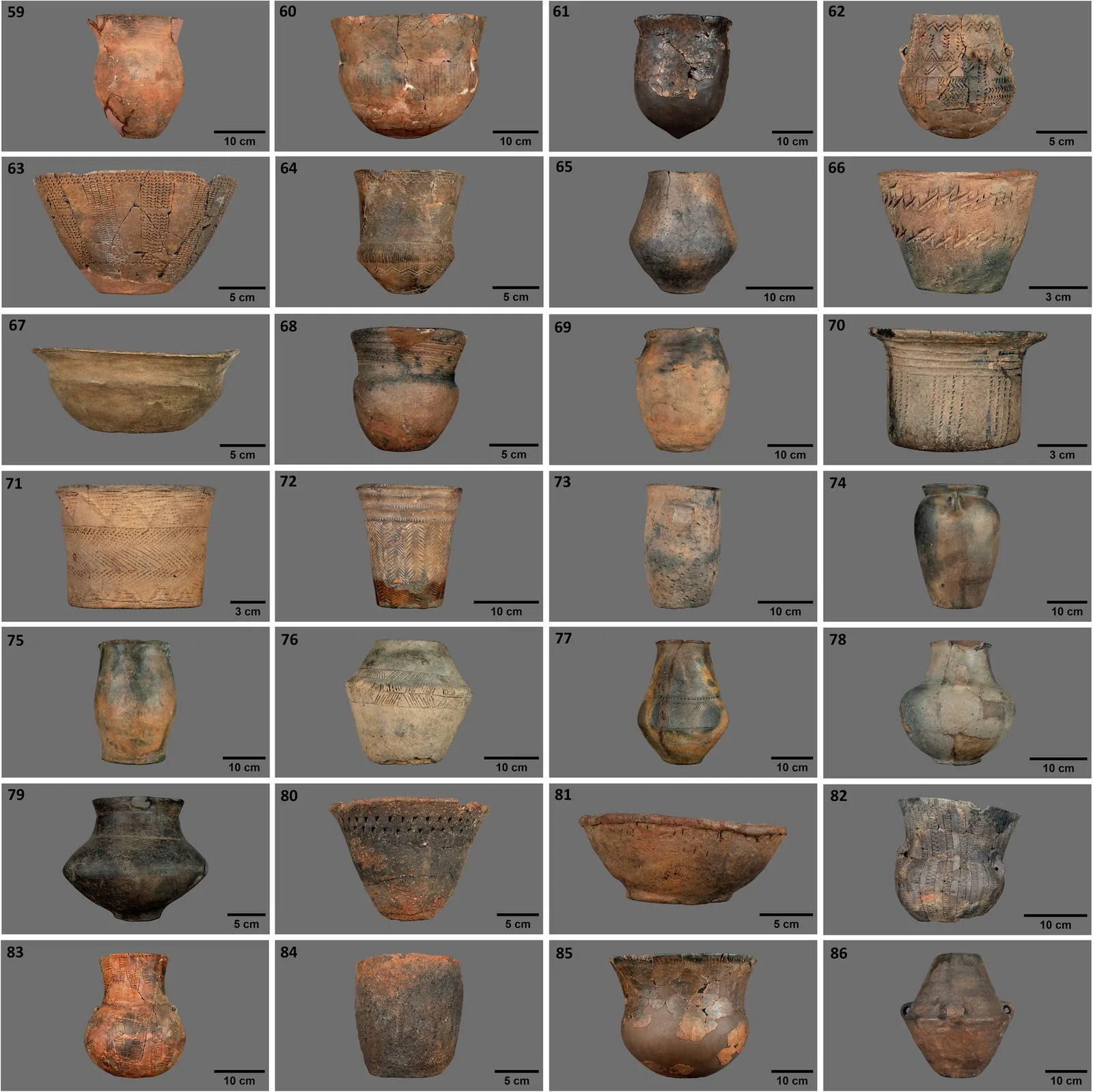
Pottery sampled from Aarhus (assets are not presented chronologically).

This procedure resulted in the selection of 28 complete or fully reconstructed pots spanning the period between c. 4800 BCE and 1 BCE, from the final Mesolithic to the late Pre-Roman Iron Age (Fig. 3). In addition, vessel surface area, aspect ratio, and luminance were recorded in order to evaluate the extent to which basic visual properties influenced attentional patterns independently from decorative organisation.

### Participants sampling

A total of 27 participants, mainly residing in the central Jutland area, took part in this study. Recruitment procedures took place between 18th December 2023 to 20th January 2024. One participant was excluded due to invalid data quality (see Supplementary Information: Experiment Report). The sampling criteria were similar as those used in the previous study (Vindrola-Padrós et al., 2026): age between 18 and 65, gender balance (women = 15, men = 10, non binary = 1, other = 1), and visual acuity of less than three dioptres of refractive error. Participants also represented different levels of familiarity with archaeology and ceramic production, including archaeologists (*n* = 12), non-archaeologist academics and naïve participants (*n* = 15), as well as individuals with experience in pottery making (*n* = 10). Socio-demographic information was recorded for each participant, including childhood environment, current residence, and level of education (see Supplementary Appendix A).

This heterogeneous sampling strategy was intended to avoid restricting the experiment to either specialised or entirely naïve viewers. Instead, it allowed the assessment of whether attentional patterns associated with prehistoric ceramic styles remained broadly consistent across participants with different degrees of familiarity with archaeological material culture, ceramic technologies, and stylistic traditions. Such variation may also better reflect the diversity of engagements that prehistoric communities themselves could have had with ceramic objects, whether as producers, users, or observers.

This protocol was approved by the Ethics Committee of Kiel University (ZEK-22-2023), and all participants provided written informed consent.

### Data recording and processing

The Pupil Core wearable eye-tracking headset, operating at a maximum sampling rate of 200 Hz, in combination with the Pupil Capture software (version 3.5.1; Pupil Labs GmbH), was used to record gaze and related data. Calibration was performed at the beginning and at the end of each session using the single-marker choreography, in which participants were asked to follow a calibration marker around the display box.

**Fig. 4:**
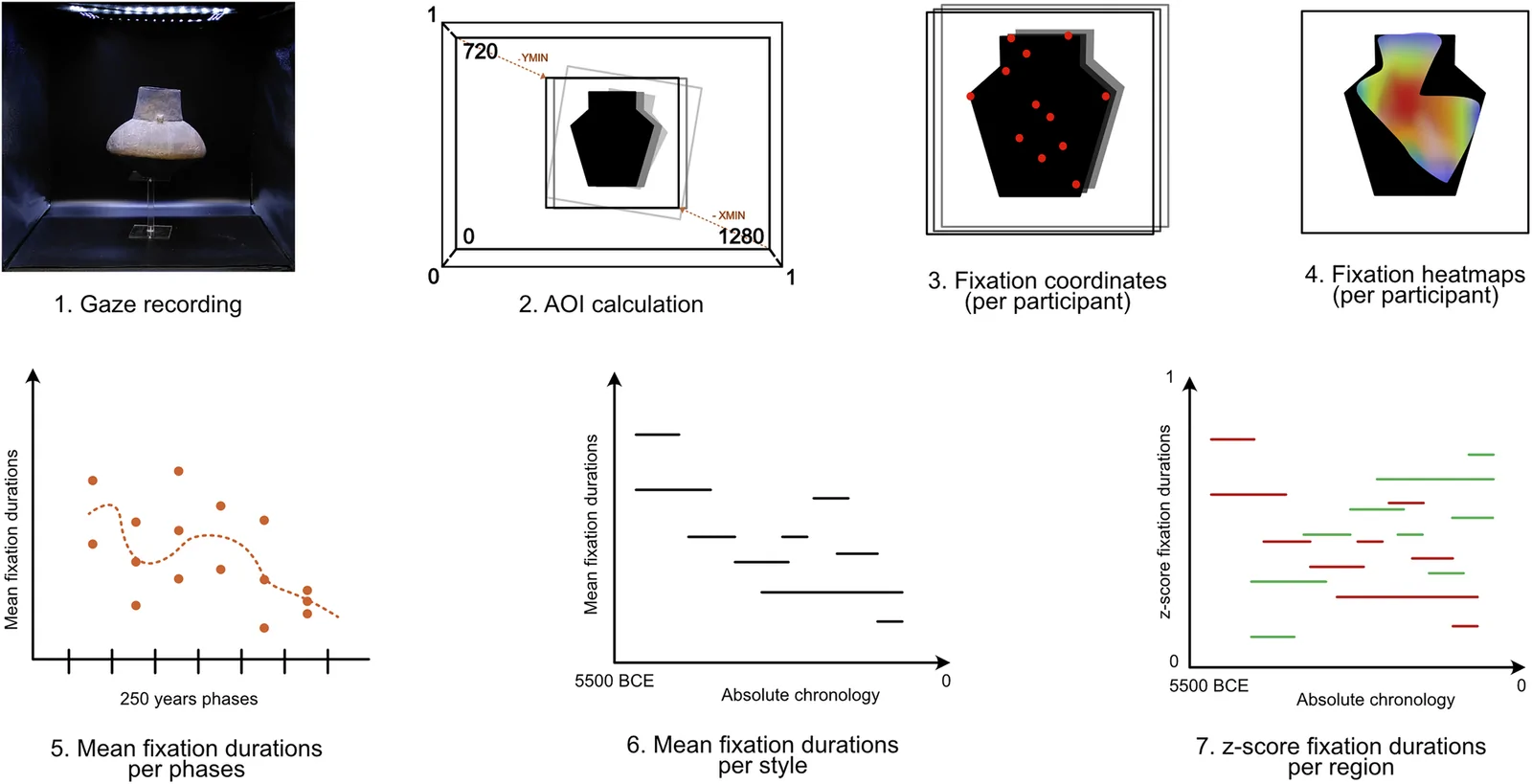
Workflow of the eye-tracking recording, processing, and analytical procedure. The figure illustrates the successive stages of the analysis, including gaze recording, AOI spatial normalisation, fixation extraction, heatmap generation, phase-based averaging, continuous chronological analysis by pottery style, and inter-regional z-score comparison.

Eye-tracking data processing and curation (Fig. 4) also followed the protocol established in the previous central German study (Vindrola-Padrós et al., 2026). Pupil Player software (version 3.5.1) was used to export gaze and fixation positions, pupil sizes and blink phases. Fixations were defined between 100 and 500 ms with a maximum dispersion of 1.50 degrees; blinks were defined using a filter length of 0.25, onset of 0.50, and offset of 0.30 confidence. Any recorded data with pupil detection quality below 0.7 were excluded.

Because participants viewed real objects rather than static digital images, fixation coordinates required spatial normalisation before visualisation (Fig. 4, step 2). For each vessel, all frames from the scene camera recordings were extracted and the edges of the display box were automatically detected in each frame using ImageJ (v1.54 f). These edges were used both to calculate rotational changes occurring during the recording and to recalculate fixation coordinates within a standardised spatial reference system, using the lower left corner of the box as the origin point (0,0).

For each participant-object recording, the corrected fixation data from all frames were then projected onto a single reference image (Fig. 4, step 3) extracted from the corresponding video and representing the photography box area of interest (AOI). This procedure made it possible to generate stabilised heatmaps for each recording while preserving the spatial relationship between gaze behaviour and the viewed object despite small head movements during the experiment.

Heatmaps were then generated for each participant-object recording in order to visualise the spatial distribution of fixations across vessel surfaces (Fig. 4, step 4). Although fixation patterns varied between individuals, recurrent concentrations of fixations made it possible to identify which types of visual features most frequently attracted attention during vessel exploration.

In addition to fixation location, mean fixation duration was calculated for each participant-object recording. In eye-tracking research, fixations correspond to periods during which visual information is preferentially processed, while fixation duration reflects the amount of time visual attention remains allocated to a specific region or object (Rayner, 2009). In the present study, fixation duration was therefore used as the principal indicator of attentional engagement during the visual exploration of ceramic forms. These values were then used in two complementary analytical procedures. First, fixation durations were averaged by chronological phase (Fig. 4, step 5) in order to examine long-term diachronic variations in attentional engagement across the prehistoric sequence. Second, object-level fixation duration values were retained (Fig. 4, step 6) for analyses comparing individual vessels and assessing the influence of specific visual attributes independently of chronological grouping.

Pupil diameter was also extracted and processed following standard normalisation and baseline-correction procedures (Fink et al., 2024), as variations in pupil size have previously been associated with processing demands and cognitive effort under controlled viewing conditions. However, because pupil measurements are highly sensitive to small variations in ambient lighting conditions (Mathôt and Vilotijević, 2023), and because recording conditions differed slightly between the Jutland and central German experiments, pupil data were considered less reliable for direct inter-regional comparison and were therefore treated only as supplementary measures (Meghanathan et al., 2015).

To assess whether attentional differences could be explained by basic visual properties rather than decorative organisation alone, vessel surface area, aspect ratio, and luminance were also recorded and statistically compared with fixation duration values.

Finally, fixation durations were transformed into z-scores to enable direct comparison between the Jutland and central German datasets (Fig. 4, step 7) despite differences in absolute fixation scales between experiments. In addition to phase-based averaging, object-level fixation durations were also visualised against their associated chronological attribution using a temporal axis (250-year bins) in order to capture continuous variation in attentional responses independent of the predefined analytical phases.

Although fixation duration and fixation location provide useful indicators of overt visual attention, these measures do not directly access symbolic interpretation or stylistic meaning. Visual attention may also be influenced by prior familiarity, individual viewing strategies, or other top-down processes, particularly within free-viewing paradigms. Consequently, the present analyses are interpreted primarily as reflecting differences in attentional engagement with the visual properties of the vessels rather than direct access to prehistoric cognitive or symbolic processes.

## Results

### Spatial distribution of visual attention

**Fig. 5:**
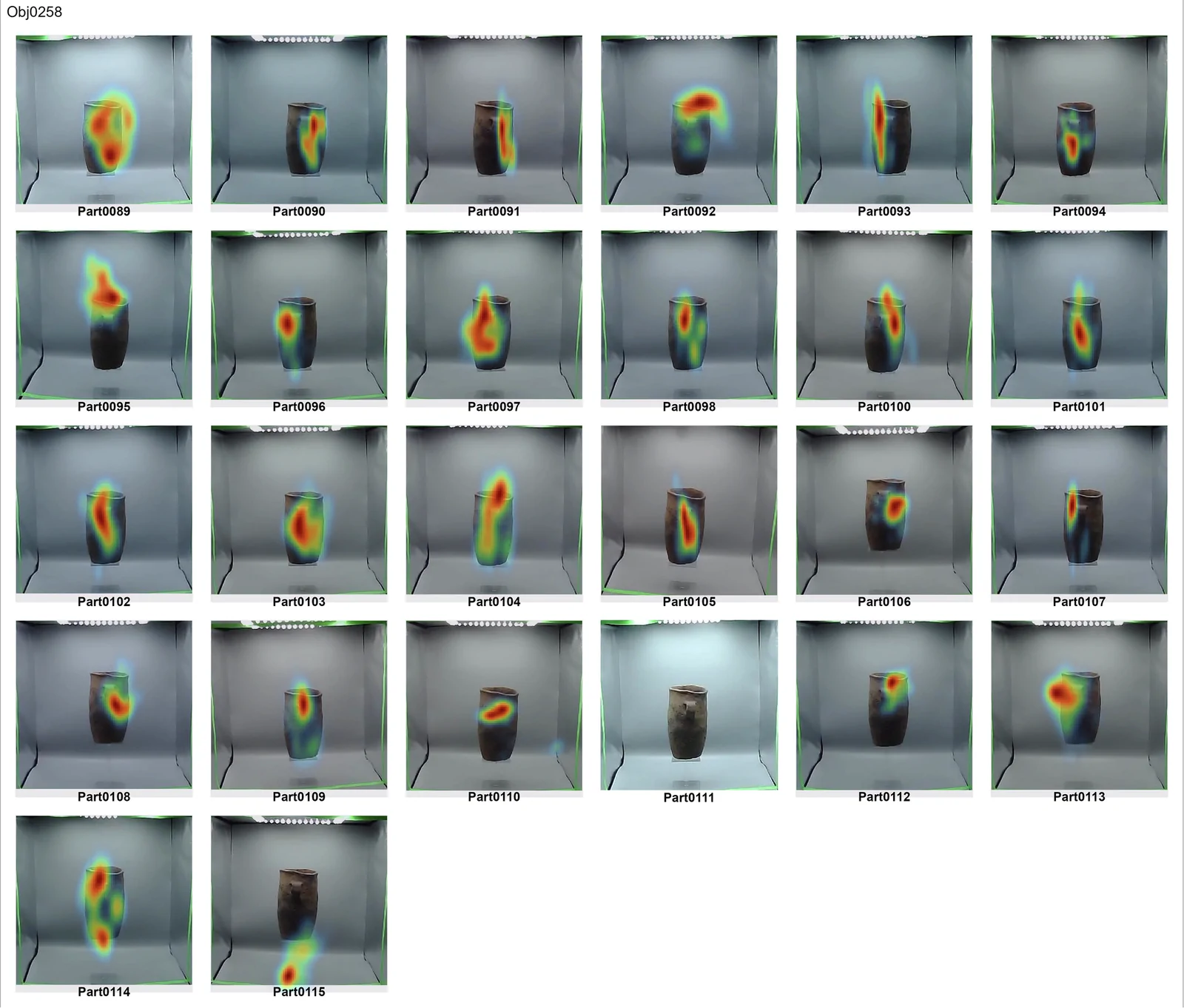
Fixations heatmaps for Asset 73 (scale from blue-low to red-high fixation density).

Heatmaps generated from participant-object recordings visualise the spatial distribution of fixations across each vessel surface (Fig. 5). Warmer colours (red) indicate areas with higher fixation density, while cooler colours correspond to regions that received less visual attention. Because participants viewed real objects rather than static images, these heatmaps represent participant-specific recordings projected onto reference images of the vessels and are therefore used here as an exploratory visualisation tool rather than as a statistical measure in themselves.

Across the dataset, fixations were predominantly concentrated on decorative elements and structural features such as handles, whereas vessel outlines and unmodified surfaces generally attracted fewer fixations. Although spatial patterns varied between individuals, these recurrent concentrations indicate stable tendencies in how viewers visually engaged with ceramic forms. For example, Fig. 5 shows that fixation density is consistently higher along decorative zones (e.g. Part0102 or Part0105), as well as around handles (e.g. Part0096 or Part0110), compared to the overall vessel silhouette. These observations are intended as qualitative indications of recurring attentional patterns and are complemented below by quantitative analyses of fixation behaviour across objects and chronological phases.

Because complete pots were required, several objects included visible reconstruction cracks. In undecorated pots, these cracks occasionally attracted fixations (see Supplementary Information: OBJ0234, e.g. Part0104 or Part0113). In more heavily decorated vessels, however, fixation density appeared more concentrated on decorative elements than on reconstructed areas. For instance, in Asset 65 (see Supplementary Information: OBJ0242), a ‘face’ urn, participants primarily fixated on the facial motif rather than on visible cracks. These observations suggest that decorative features constituted visually salient regions during the exploration of many vessels, despite the presence of other potentially attention-grabbing surface irregularities. Consequently, the following analyses examine whether broader variations in fixation behaviour can be identified across ceramic styles and chronological phases.

### Fixation duration and pupil diameter across chronological phases

**Fig. 6:**
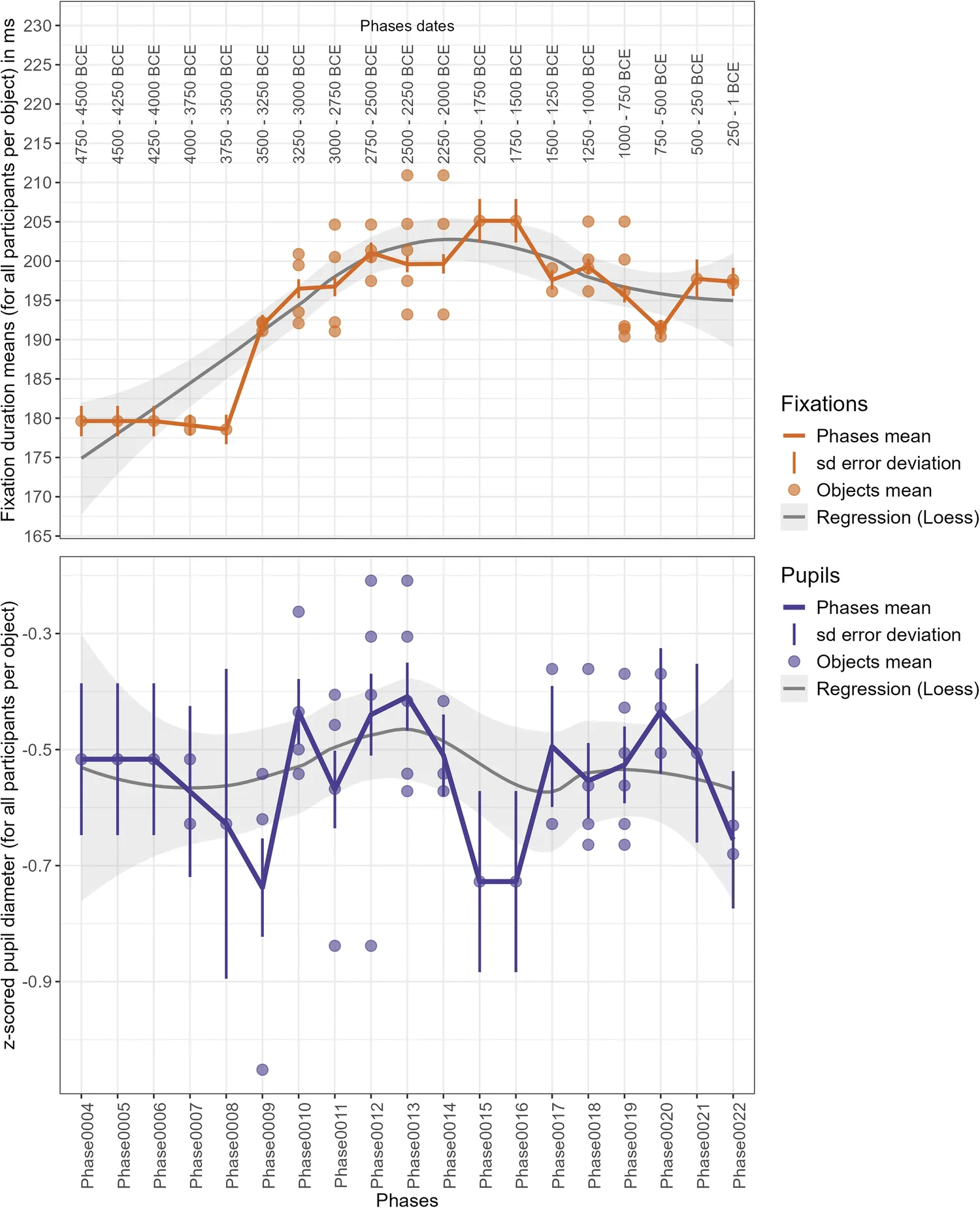
Mean fixation durations (top) and mean pupil diameter (bottom) per phase (line) and per objects (dots). Error bars represent the standard error of the mean.

The top part of Fig. 6 presents mean fixation duration values calculated for each object across participants (individual points), together with chronological phase averages (connected line) and a LOESS regression curve highlighting the overall trend through time. The bottom part Fig. 6 displays the same procedure applied to baselined pupil diameter measurements.

Mean fixation duration values vary across the chronological sequence. The lowest values occur during phases 04–08 (4750–3500 BCE), followed by a marked peak in phase 09 (3500–3250 BCE). From phases 10–16 (3250–1500 BCE), fixation durations show a gradual increase accompanied by greater inter-object variability. From phase 16 onwards, mean fixation durations generally decrease toward the end of the sequence, with the exception of a smaller peak during phase 21 (500–250 BCE). This later decline broadly parallels the reduction in both stylistic diversity and fixation-duration variability observed in the dataset. However, the absolute differences between many chronological phases remain relatively small, often below 10 ms. Consequently, these variations should not be interpreted as discrete cognitive shifts between archaeological periods, but rather as broader fluctuations in attentional engagement across the long-term stylistic sequence.

**Fig. 7:**
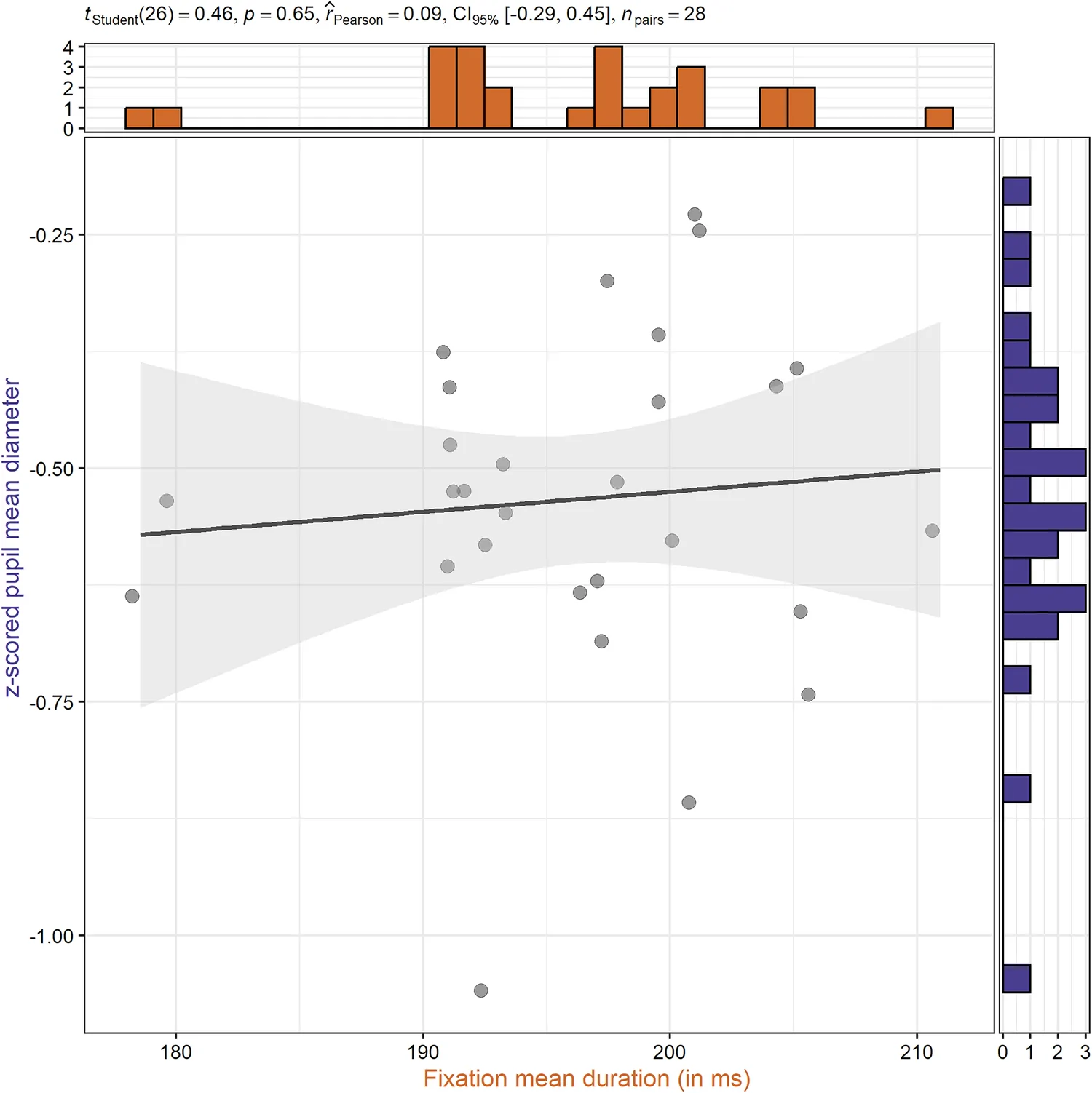
Correlation of the distribution of fixation duration and z-scored pupil diameter.

In contrast to the results obtained for the central Germany assemblage (Vindrola-Padrós at al., 2026), no clear correlation was observed between fixation duration and pupil diameter in the Jutland dataset (Fig. 7). Pupil measurements also displayed substantially greater variability than fixation duration values. As discussed in the methodological section, pupil diameter was therefore treated only as a supplementary measure, and subsequent analyses rely primarily on fixation duration.

### Influence of basic visual properties

**Fig. 8:**
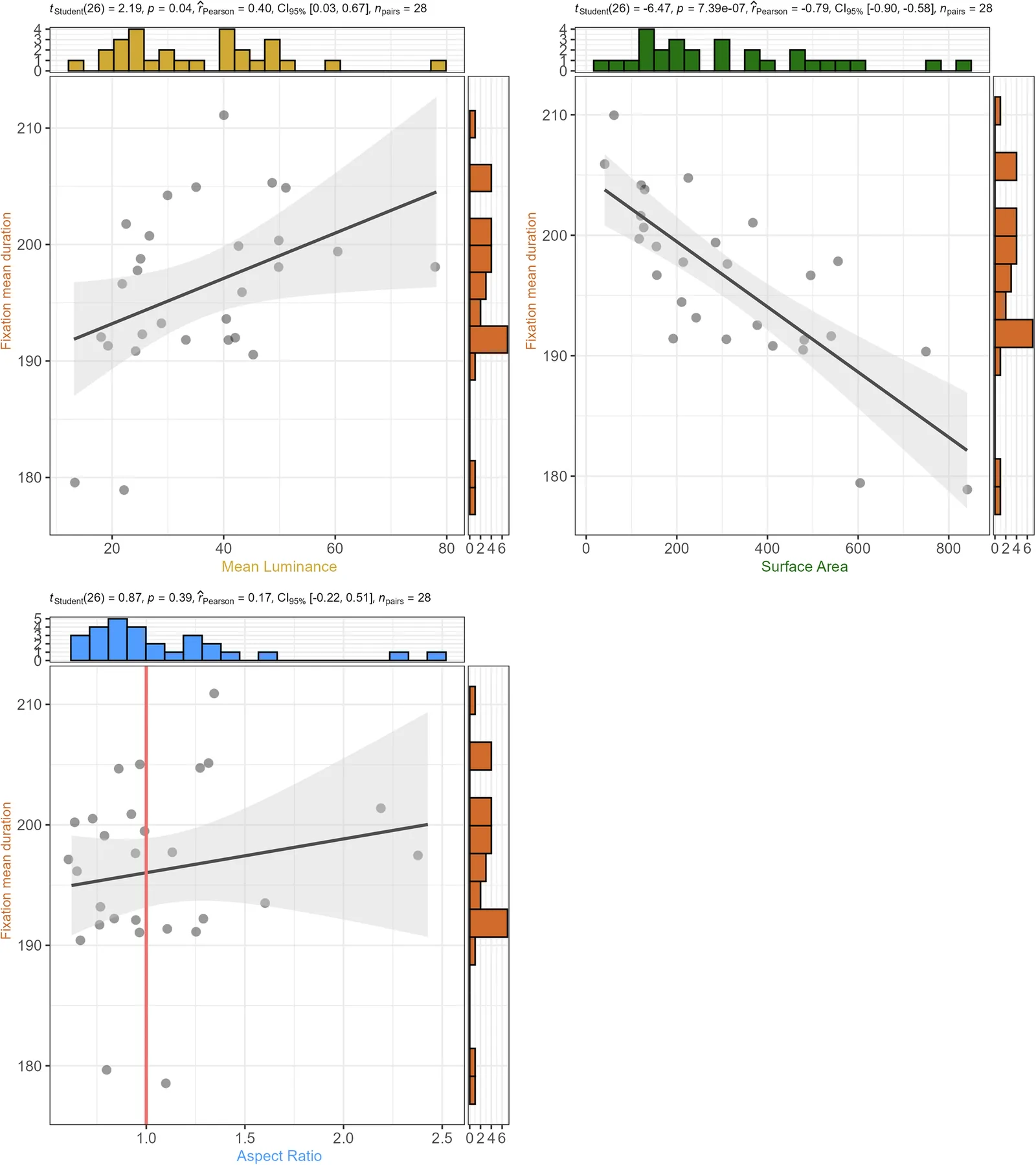
Correlation between object attributes—luminance, size (surface area) and aspect ratio (<1 for vertically elongated, >1 for horizontally elongated objects) with fixation duration.

To ensure that fluctuations in fixation duration reflected participants’ responses to changes in decorative patterns rather than other visual properties of the vessels, we attempted to correlate luminance, size (surface area), and shape (aspect ratio) with mean fixation duration (Fig. 8).

Among these variables, only vessel size displayed a strong negative correlation with fixation duration, confirming previous observations that larger objects tend to produce shorter fixation durations (Nuthmann et al., 2020; Vindrola-Padrós et al., 2026). This relationship may partly explain the particularly low fixation duration values observed during the earliest phases of the sequence (phases 04–08), which correspond to the largest vessels in the sample. This pattern, however, reflects broader characteristics of the archaeological record itself, as the earliest pottery traditions in Jutland are predominantly represented by large vessels.

In later phases, however, vessel sizes become more evenly distributed despite continued variation in fixation duration values (see Supplementary Appendix B). This suggests that vessel size alone cannot fully account for the diachronic patterns observed in Fig. 6.

Combined with the heatmap analyses showing recurrent fixation concentrations on decorative zones and structural features, these results suggest that stylistic organisation likely contributed to observed variations in attentional engagement. Nevertheless, because multiple visual and contextual factors may simultaneously influence eye movements, the present results should be interpreted as indicating associations between stylistic variation and visual attention rather than direct causal relationships.

### Inter-regional comparison with central Germany

The analysis previously conducted on central Germany ceramics yielded two main results: fixation locations were primarily concentrated on decorative elements, and fixation duration values varied across stylistic phases, with an overall decline from the Neolithic to the Iron Age (Vindrola-Padrós et al., 2026).

The aim of the present paper is to determine whether comparable long-term attentional patterns could also be observed in Jutland. As shown above, both regions display recurrent fixation concentrations on decorative zones, and fixation duration values that vary across chronological phases. A central challenge in comparing the two regions lies in differences in their underlying archaeological chronological frameworks. In addition, the fixation durations values differ in their scale, which limits direct comparison of raw values across regions. These constraints necessitate a comparative approach based on standardised and multi-scalar temporal representations of the data.

**Fig. 9:**
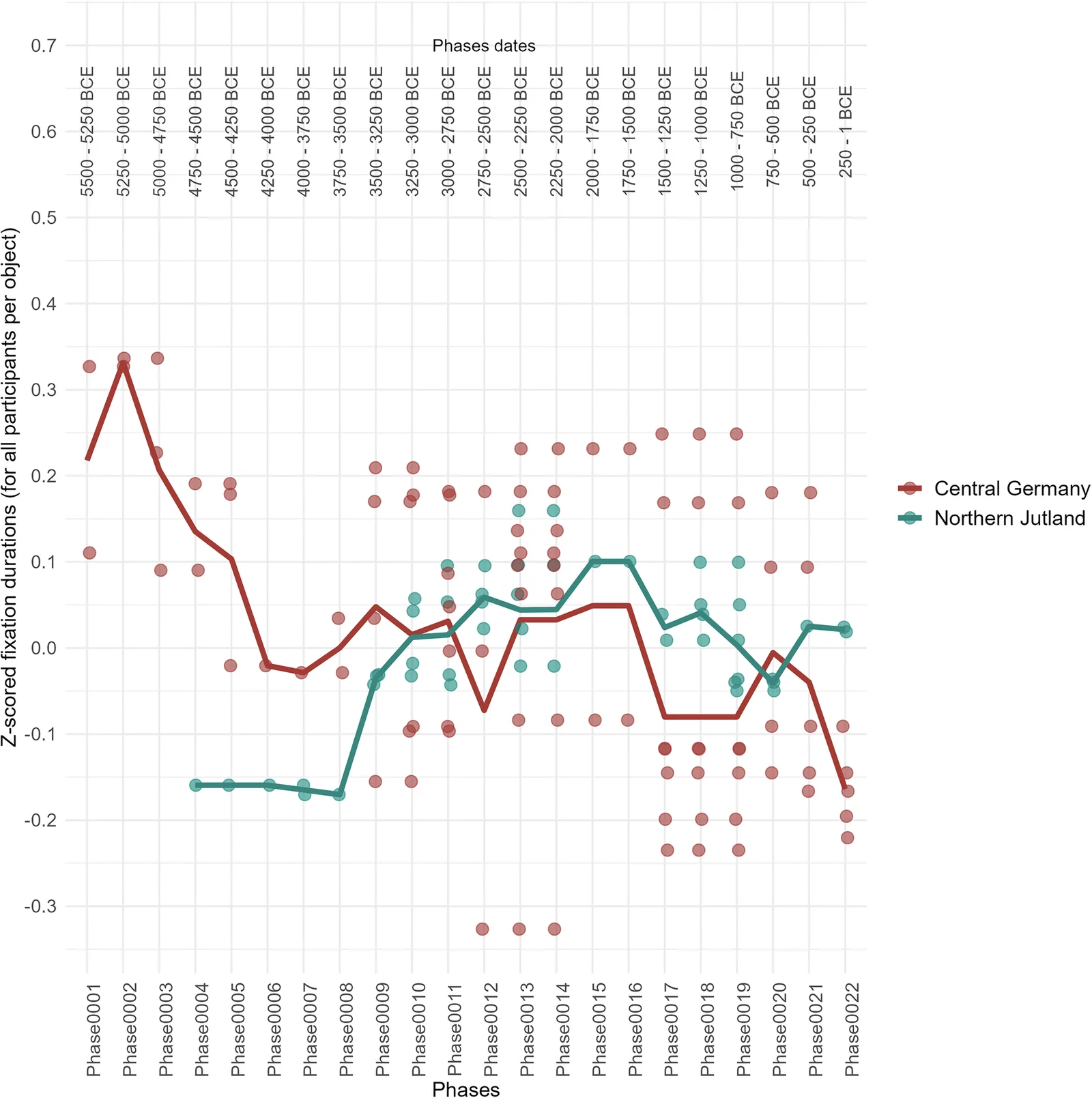
Compared z-scored mean fixation durations for central Germany (red) and Jutland (green) by phases.

When the two regional datasets are compared using the previously defined 250-year analytical intervals (Fig. 9), Jutland and central Germany do not display identical phase-by-phase fluctuations. Nevertheless, fixation duration values follow broadly similar long-term trajectories from phase 09 (3500–3250 BCE) onwards until phase 20 (1000–750 BCE).

In contrast, when fixation duration values were reorganised according to broader archaeological chronological classifications shared between the two regions (e.g. Neolithic, Bronze Age, Iron Age), no clear correspondence between the datasets could be observed (see Supplementary Appendix C). The resulting trajectories follow substantially different trends, indicating that similarities in attentional patterns cannot be explained simply through equivalent archaeological periodisations or socio-economic transitions.

**Fig. 10:**
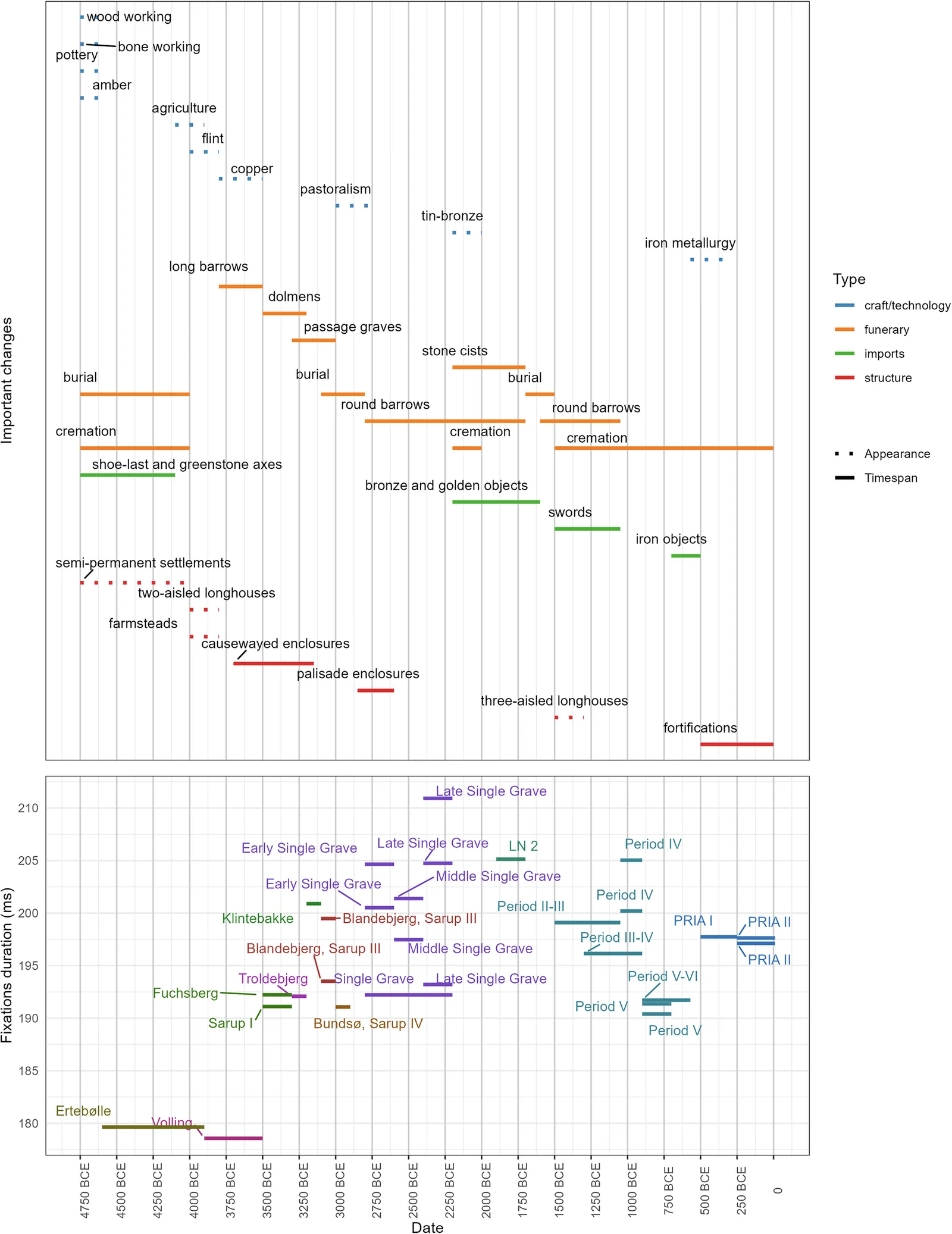
Integration of important social and economic changes (top) with mean fixation duration of each pottery style type for all participants in chronological order (bottom).

**Fig. 11:**
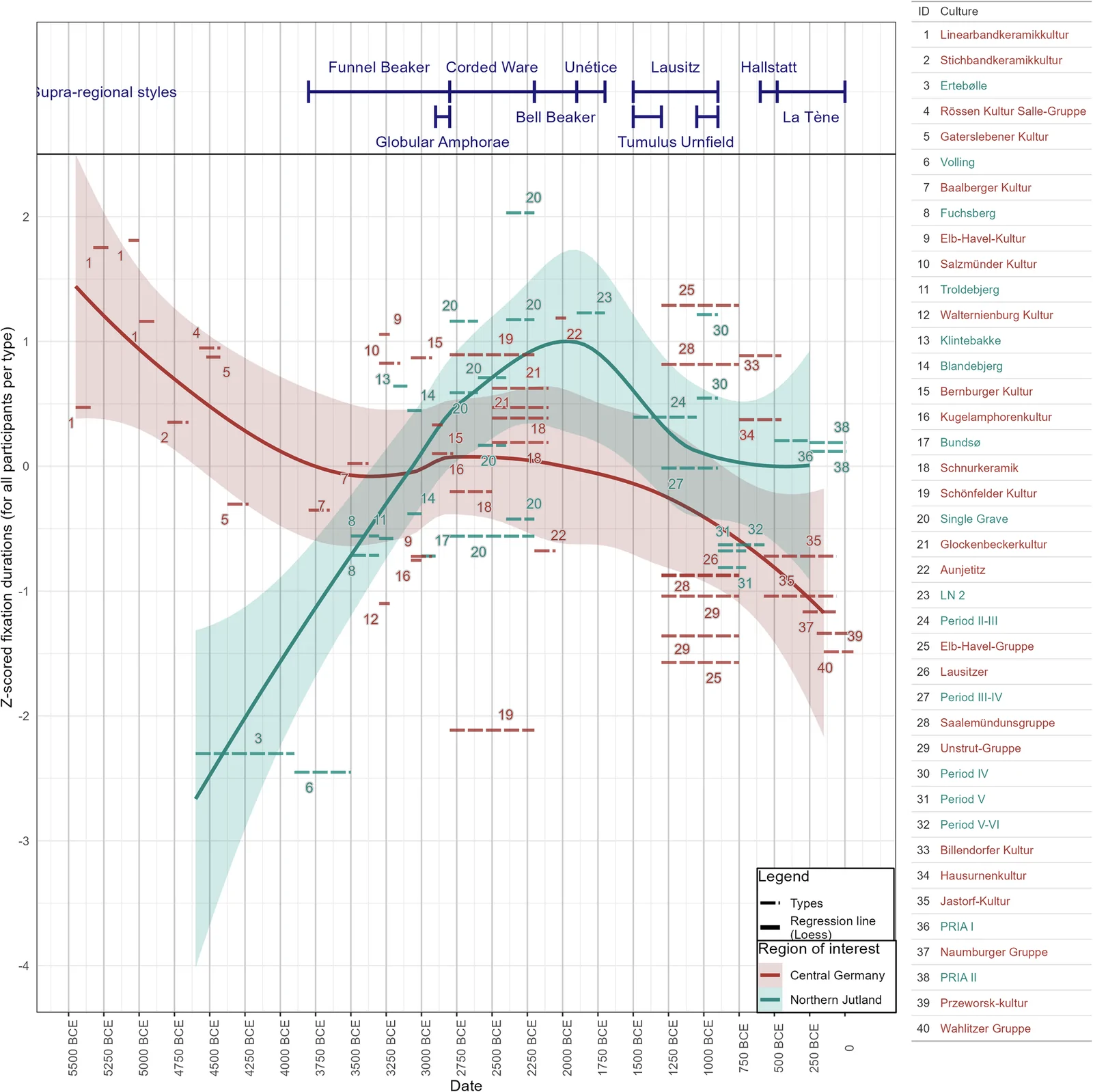
Compared z-scored fixation durations of the ceramic styles from central Germany (red) and Jutland (green) in absolute chronology.

However, when fixation duration values calculated for individual objects were plotted according to the chronological attribution of their associated ceramic styles on a continuous temporal scale (Fig. 10), similar long-term trajectories again became visible between the two regions. Figure 11 displays the mean fixation duration associated with each ceramic style, with Jutland represented in green and central Germany in red. LOESS regression curves calculated independently for each region highlight the broader temporal trends underlying the variability between individual objects. Although the distributions remain partially asynchronous, both regional trajectories begin to converge around 3000 BCE and subsequently display comparable large-scale variations through time.

Taken together, these results suggest that similarities between the Jutland and central Germany datasets are expressed more consistently within an absolute chronological framework than through regionally defined archaeological sequences. Rather than aligning with discrete cultural-historical transitions, the observed variations in fixation duration appear to reflect more gradual and partially asynchronous long-term developments.

These results should nevertheless be interpreted with caution. While fixation duration does not provide direct access to symbolic meaning or cognition in its broadest sense, it remains a robust indicator of overt visual attention and attentional engagement. The observed patterns therefore support the interpretation that long-term transformations in ceramic visual organisation were associated with comparable patterns of visual attention across both regions.

## Discussion: visual attention and long-term stylistic transformations

In the following discussion, variations in fixation duration are examined in relation to long-term transformations in pottery production, decorative organisation, and broader socio-historical developments in Jutland. Rather than treating eye-tracking measurements as direct evidence for cultural cognition or symbolic meaning, the objective is to explore whether recurrent changes in visual attention patterns may correspond to broader shifts in material practices, supra-regional interactions, and the importance attributed to decorative expression through time.

Figure 10 presents mean fixation duration values for all participant per pottery type, based on the stylistic sequence detailed in Fig. 2 (see also Supplementary Information: Table). By plotting individual ceramic styles on a continuous chronological axis, the figure provides a finer temporal resolution and allows comparison with major social, economic, funerary, and crafts-related transformations. Based on these observations, together with the data presented in Fig. 6, four broad phases in the organisation of visual attention can be distinguished within the Jutland sequence.

Figure 11 compares the standardised fixation duration values associated with individual pottery styles in Jutland and central Germany. The comparison highlights long-term similarities and divergences in visual attention trajectories between the two regions and provides a framework for discussing how broader transformations in material culture may have structured stylistic investment through time.

The first phase (4600–3500 BCE) encompasses the end of the Final Mesolithic and Early Neolithic I (ENI), and is characterised by the lowest mean fixation duration values of the entire sequence. As noted earlier, this pattern may partly reflect vessel size, since the largest pots in the dataset belong to this period. However, these dimensions also correspond to the dominant ceramic forms documented archaeologically at the time. For instance, Ertebølle assemblages are dominated by larger vessels, while only a minority of vessels, including the so-called ‘blubber lamps’, are smaller.

Pottery from this phase was also mostly undecorated, as observed for Ertebølle vessels throughout Jutland and for the Wangels groups in southern Jutland. When decorated, it was most often limited to a few decorative patterns below the vessel’s rims (Müller and Peterson, 2015, 576), similarly to the earliest Neolithic pottery documented elsewhere in southern Scandinavia (Koch, 1998). The combination of large vessel size and limited decoration organisation may explain the relatively low fixation duration values observed during this phase. Some variability nevertheless appears during the later part of the first phase in the geographically restricted Volling style of northern and central Jutland, which included vessels with highly complex decorative patterns. However, the vessel sampled in the present study displayed only limited decorative elements.

From a socio-historical perspective, this phase corresponds to the introduction of pottery among hunter-gatherer-fisher communities, most likely influenced by eastern Baltic networks rather than those of central Europe. Pottery appears to have been used primarily for food processing (Craig et al., 2007; Craig et al., 2011; Papakosta et al., 2019). At the same time, ceramic vessels remain largely absent from mortuary contexts and are generally only sparsely decorated. In contrast, stylistic investment during this period appears more visible in other media, including bone, antler, wood or amber objects (Andersen, 2013; Jensen, 2013, 108; Skriver et al., 2017; Andersen, 2023).

By the time FBC pottery emerged in Jutland, a gradual shift in stylistic investment towards ceramic production was likely already underway. This process could be interpreted through the concept of media displacement, understood as a transfer of stylistic complexity from one material medium to another (Roe, 1995, 56). This reorganisation of stylistic investment may have been associated with the arrival of populations displaying stronger affinities with central European Neolithic traditions (Mittnik et al., 2017; Allentoft et al., 2024). However, given the limited number of vessels available for this early phase, these transformations remain only partially visible in the present dataset and become clearer in the subsequent period.

In central Germany, a different trajectory can be observed during the same broad chronological interval. Highly captivating Linearbandkeramik, Stichbandkeramik, and Early Rössen styles were replaced by largely undecorated asssemblages such as Late Rössen, Gatersleben and Baalberg (Müller, 2001, 92–94; Kaufmann, 2017). This reduction in decorative complexity corresponds to lower fixation duration values in the central German dataset. However, although both regions display comparatively low attentional measures, the underlying archaeological processes appear distinct. In Jutland, stylistic investment may have remained concentrated in other media, whereas in central Germany ceramics continued to function as a major medium of stylistic and symbolic expression, with increasing emphasis placed on vessel morphology rather than surface decoration.

The second phase (3500–2000 BCE) spans a long interval between the second part of the Early Neolithic (ENII) to the Younger Neolithic, during which we observed a progressive increase in mean fixation duration, accompanied by increasing variability in pottery forms and decorative organisation. This trend may have continued into the Late Neolithic, particularly LN I, but unfortunately no vessels from this period met our sampling criteria. The rise in variation and fixation duration is especially notable in the Fuchsberg style, characterised by chevron and ladder-like impressions covering larger portions of the vessels. Subsequent Middle Neolithic styles such as Klintebakke, Troldebjerg, Blandebjerg and Bundsø display further increases in complexity, not only in the number of motifs but also in their orientation and structuring across vessel surfaces (Madsen, 2024, 467; Bloch and Johannsen, 2025). A particularly striking development is the introduction of chevron patterns with different orientations, some inverted and others rotated (e.g. Asset 62, Blandebjerg style), which are known to increase visual processing demands under experimental viewing conditions (i.e. an inversion cost; Beitner et al., 2022; Hayes and Henderson, 2022). In the Younger Neolithic, the highly decorated flat-bottomed cylindrical vessels from the late Single Grave Culture (SGC) adopt a similar strategy and associated with similarly elevated fixation duration values, producing the highest mean fixation durations observed in the sequence.

Socio-historically, this second phase is characterised by the expansion of pottery style into supra-regional networks, such as Funnel Beaker Culture (FBC) and SGC (with Corded Ware and later Bell Beaker influences). This expansion of supra-regional interactions may have contributed to the increasing diversity of pottery designs in the Jutland region and may account for the higher fixation duration values observed during this phase. The Fuchsberg style marks the beginning of supra-regional styles within the FBC, which continued into the Middle Neolithic. During this period, specific vessel types, such as bowls and lugged beakers, were deposited in highly symbolic contexts, notably tombs and causewayed enclosures (Madsen, 1997, 79). At the same time, regionalisation of ceramic traditions became important for expressing cultural affiliation and identity (Müller and Peterson, 2015, 580). While flint and amber retained strong symbolic and economic importance (Ramstad et al., 2015; Earle et al., 2023), and copper artefacts were introduced (Klassen, 2000; Gebauer et al., 2021; Brozio et al., 2023) pottery appears to have become a new medium of symbolic expression in both funerary and domestic contexts (as illustrated, for instance, at Sarup (Andersen, 1997). This period also saw the peak in frequency and importance of megalithic graves (Ebbesen, 1985, 40; Jensen, 2013, 185), with passage graves associated with FBC pottery becoming widespread, contexts within which pottery may have acquired increasing symbolic and stylistic importance.

This trend may have continued into the Younger Neolithic, across the different phases of the SGC, despite socio-political transformations such as the shift towards individualisation within certain central family lineages (Müller, 2010; Madsen, 2024, 409) and the substantial genetic and cultural influence of Corded Ware groups (Kristiansen, 1989; Nielsen and Johannsen, 2023; Allentoft et al., 2024). Mortuary practices also diversified with the introduction of single graves placed in earthen mounds around 2800 BCE, including wooden coffins within ring ditches (Madsen, 2024, 426–427), i.e. mortuary palisades (Nielsen and Johannsen, 2023), alongside the reuse of dolmens and passage graves from around 2600 BCE (Hübner, 2005). While these were significant socio-historical transformations with potential implications for pottery designs, they left little trace in our dataset other than slightly longer mean fixation durations. This is also likely linked to the absence of vessels from the preceding MN V Store Valby style in our sample, characterised by less decorated, thicker and coarser-grained wares (Müller, 1918; Brozio et al., 2019; Madsen, 2024, 470), which may well have been associated with lower responses and, consequently, highlighted the well-known resurgence of the ceramic craft in the subsequent SGC. Interestingly, while no major impact on pottery style designs can be observed, the MN V phases does show evidence of influence from pastoralist Globular Amphora Culture in Jutland, such as cattle burials and the intensification of animal husbandry (Johannsen and Laursen, 2010; Johannsen et al., 2016; Madsen, 2024, 479).

In central Germany, a comparable development of supra-regional networks coincided with an increase in the variation and number of pottery styles. Globular Amphora, Corded Ware and Bell Beaker assemblages are characterised by a revitalisation of decorative pattern design, exemplified by hybridised decorative patterns such as those combining Bernburg and Globular Amphora elements (Müller, 2023). By the Early Aunjetitz (Unétice), around 2200 BCE, decorative motifs had become scarce and the number of ceramic types had declined (Großmann, 2016, 115). Funerary practices also underwent significant changes (Haak et al., 2008; Meyer et al., 2009; Spatzier and Bertemes, 2018), partly driven by increasing social differentiation, and may have encouraged more selective investment in decorative pottery associated with funerary contexts. In southern Scandinavia, these supra-regional networks similarly shaped social organisation, as reflected in mortuary practices and the pottery grave goods.

A third phase can be distinguished, corresponding to the ‘hiatus’ between LN II and Bronze Age Period I, and extending into the Younger Bronze Age (1700–500 BCE; Fig. 11). Pottery designs during this phase appear comparatively less dynamic: vessels were generally less decorated and tended towards simpler, more standardised forms. At the onset of the Bronze Age, general vessel types were maintained, although stylistic consistency is less clearly defined, as reflected in the variability of face urns in period IV. This contrasts with Bronze Age Periods V and VI, during which stylistic variation appears more restricted (Lolk, 2009, 81). As a result, aside from vessel shape asymmetries, which are known to require greater visual processing than symmetrical forms (Wagemans, 1995, 14), and occasionally handles, which tend to attract attention as manipulable features (Federico and Brandimonte, 2019), few stylistic elements appear to contribute strongly to fixation patterns. Although some variation is introduced through decorative elements inspired by Lusatian and Tumulus styles in Period II, mean fixation durations show a gradual decrease from the Older Bronze Age onwards and through to Period VI of the Younger Bronze Age.

In terms of socio-historical developments during this phase, pottery appears to have gradually lost its role as a primary medium for prestige and symbolic display, while other craft categories, such as metalwork, became increasingly important as an indicator of status from LN II onwards (Vandkilde, 1996, 305). This shift is consistent with broader changes in metal production and exchange networks (Vandkilde et al., 2024), which contributed to the consolidation of increasingly hierarchical structures of small- and medium-sized chiefdoms at the beginning of the Bronze Age (Nørgaard, 2018, 360). This hierarchy is, for example, reflected in the variation in barrow size, which has been interpreted as signalling differences in social affiliation and membership within ‘chiefly lineages’ (Kristensen, 2018, 119). Within this context, investment in pottery design appears to have been reduced relative to other material domains. In contrast, bronze objects,became predominant in grave goods, hoards and settlements assemblages during this period (Earle, 2004, 120). The development of metallurgy and specialised techniques enabled both an increased in production and greater typological and ornamental variability, especially for weapons and tools (Kristiansen, 1987; Vandkilde, 1996; Johannsen, 2015, 79). While pottery designs became less varied and less elaborated during this phase, bronze artefacts display substantial ornamental and typological diversity and occupy prominent positions within funerary and depositional contexts. This pattern is consistent with the hypothesis that part of the stylistic and symbolic investment previously associated with pottery became increasingly directed towards metal objects, which appear to have played a growing role in expressions of prestige and social differentiation. It is possible that this phenomenon may have begun earlier with the Early Dagger Groups in LN I (Vandkilde, 1996, 299, 305), when flint held a highly esteemed position, and that it may have continued into LN II with the production and import of bronze artefacts.

At the onset of the Early Bronze Age in central Germany (LN II in Jutland), the Aunjetitz group prevailed, establishing supra-regional networks focused on metallurgy and the exchange of metal objects, while ceramic production was reduced to a limited range of standardised forms (Meller, 2019). Following the collapse of this system, central Germany fragmented into several regional groups (Revert Francés, 2022) characterised by distinct settlement patterns and mortuary practices (e.g. cremations and burial mounds). Within this context, two main groups emerged: Lausitz (Lausatian) and Tumulus groups. While pottery within the former occasionally featured elaborate garland motifs, metal objects bore the most complex ornamentation and appear more frequently in highly symbolic contexts. This contrast may indicate a broader shift in stylistic investment towards metal artefacts as media associated with prestige and social distinction.

In sum, from LN II onwards, a core-periphery system developed between the *Únětice* groups in central Germany and the family lineages established in Jutland (Vandkilde, 1996, 299; Nørgaard et al., 2021), centred on the exchange of bronze (e.g. Skeldal hoard; Vandkilde, 1988). Within this framework, the reduced stylistic investment observed in pottery may correspond to a broader reorganisation of craft production and prestige media, in which metal artefacts occupied a more prominent role. These developments are also consistent with the reinforcement of hierarchical social structures that persisted beyond the collapse of the *Únětice* groups. Afterwards, the Nordic Bronze Age, shaped by influences from the Tumulus and Lusatian groups, appears to have further reinforced this process of media displacement.

The fourth and final phase extends throughout the Pre-Roman Iron Age (500–1 BCE; PRIA), during which a slight increase in fixation duration values is observed, accompanied by a marked reduction in variation between vessels. PRIA vessels were undecorated, with symmetrical shapes and homogenous texture, as exemplified by the burnished surfaces of Asset 74 and 79. These attributes correspond to comparatively reduced variation in visual responses. However, despite these relatively simplified visual characteristics, fixation duration values do not decrease as strongly as might be expected. This discrepancy may be related to multiple factors, such as the cracked condition of certain vessels (e.g. Asset 78) or the presence of handles (e.g. Asset 74), both of which may locally attract visual attention.

The socio-historical developments during this phase are mainly associated with the standardisation and specialisation of pottery production, and the growing importance of iron metallurgy (Nørbach, 1998), both within a context of intensified social differentiation. Pottery making becomes more specialised, as seen in the reduced variability of designs (and, consequently, with their visual effects), and was no longer confined to household production, as attested by the presence of pottery kilns in the Late PRIA (Jensen, 2013, 615), such as the dome-shaped kilns at Hodde (Hvass, 1975). Despite the expansion of iron production, metal objects remained rare in Jutland during this part of the period (Martens, 1992, 129).

In central Germany it’s the corresponding Late Iron Age developments involved major technological and organisational transformations in ceramic production, including the introduction of the potter’s wheel (Thér et al., 2017) and the emergence of specialised workshops within agglomerated settlements. Consequently, ceramic assemblages display more regularised forms and more homogeneous surface treatments compared to earlier periods. In the present study, such features tend to co-occur with comparatively limited variation in visual responses. Metal production also occupied an important place within exchange systems and was frequently involved in the circulation of objects associated with social status and symbolic value. In comparison, the potter’s wheel had not yet been introduced in Jutland, and the organisation of metal production and circulation differed substantially between the two regions. These differences may account for the remaining divergences observed in fixation duration trajectories (Fig. 11).

Our results are consistent with phases characterised by highly decorated pottery styles, which may be interpreted as periods in which ceramics served as a medium for conveying regional and social information. They are also consistent with phases marked by reduced investment in decorative patterning, during which attention may have shifted towards other media carrying comparable social or symbolic significance, such as amber and antler in the Mesolithic and metal in later periods.

Rather than representing isolated developments, these alternations appear to be embedded within broader supra-regional networks of interaction, which structured the circulation of materials, techniques, and stylistic practices across northern Europe. These networks may have contributed to shaping both the emergence and the decline of visually elaborate ceramic traditions.

These patterns are associated with the emergence of new materials and production techniques that reshaped material culture and became particularly evident when the Jutland sequence is compared with central Germany.

## Conclusion

This article set out to test whether the socio-historical and material culture transformations that occurred in both central Germany and Jutland over the last five-thousand years BCE are associated with comparable patterns of visual attention to pottery styles. In other words, our hypothesis was that fixation-based measures associated with different ceramic designs would follow similar long-term trajectories in both regions, in line with their shared history of stylistic designs since at least the Early Neolithic in northern Europe.

Our comparative results are broadly consistent with this hypothesis. From 3500 BCE onwards, pottery styles from both regions show partially parallel trajectories in measured fixation duration, particularly during periods associated with the expansion of supra-regional interaction networks such as the Funnel Beaker phenomenon. Comparable large-scale variations are observable during periods marked by the development of supra-regional interaction networks associated with Corded Ware, Bell Beaker, Únětice, Tumulus, Lusatian, and La Tène assemblages. In the case of the Funnel Beaker and Corded Ware networks, such parallels are also consistent with evidence for substantial interregional mobility and connectivity, as recently demonstrated by genetic studies (Mittnik et al., 2017; Allentoft et al., 2024).

Importantly, these correspondences are most evident when the datasets are aligned through absolute chronology rather than through traditional archaeological periodisation. This distinction is particularly important because both archaeological chronologies are themselves structured around comparable socio-historical developments. However, the trajectories become less closely aligned when the fixation duration data are organised according to these frameworks. The observed similarities appear to be less tied to equivalent developmental stages and more reflective of long-term processes of interaction, exchange, and stylistic diffusion between connected regions. In this perspective, supra-regional networks may have played a structuring role in the circulation and transformation of decorative practices across prehistoric Europe.

One notable exception is observed at the beginning of the sequence. Contrasting results between the Final Mesolithic in Jutland and the Early Neolithic in central Germany suggest markedly different forms of stylistic investment in pottery during the initial adoption of ceramic technology. While Early Neolithic pottery traditions in central Germany are characterised by highly elaborated decorative systems, which tend to be associated with comparatively higher fixation duration values, contemporaneous Ertebølle pottery in Jutland remained largely undecorated and appears to have fulfilled primarily functional purposes linked to food-processing. At the same time, symbolic and decorative investment in Mesolithic Jutland appears to have been directed towards other material categories such as amber, antler, bone, and wood. These differences suggest distinct early trajectories of ceramic development under different regional interaction spheres, including eastern Baltic influences, despite evidence for some degree of contact with central Europe (Klassen, 2004).

From the emergence of the Funnel Beaker complex onwards, however, pottery traditions in Jutland became increasingly integrated within broader supra-regional stylistic networks. This development is particularly visible in the Fuchsberg style from around 3500 BCE, plausibly also in the slightly earlier Volling style, whose decorative organisation displays stronger similarities with wider northern and central European traditions.

At the same time, the present study identifies periods characterised by reduced decorative investment in pottery and comparatively lower fixation duration values. Such phases often co-occur with the growing importance of other forms of material production, particularly metalwork during the Bronze and Iron Ages. However, because the present analysis focuses exclusively on ceramic material, these correspondences should be interpreted cautiously. The results suggest that changes in the visual organisation of ceramic styles may have formed part of broader reorganisations of material culture occurring alongside transformations in technological practices, exchange systems, and social differentiation.

More generally, this study demonstrates the utility of eye-tracking methods for investigating long-term variations in the visual properties of archaeological material culture. While fixation duration and fixation distribution do not provide direct access to prehistoric cognition, symbolic meaning, or cultural perception, they nevertheless offer measurable indicators of how different stylistic organisations are associated with contemporary visual exploration. Combined with archaeological contextualisation, such approaches make it possible to investigate large-scale changes in stylistic organisation and to explore how these changes may relate to broader patterns of interaction and cultural transformation across prehistoric Europe.

Future work integrating additional material categories, larger archaeological samples, and controlled comparative experiments will be necessary to evaluate more directly the extent to which these long-term variations in visual attention relate to broader transformations in prehistoric material culture and interaction networks.

## Supplementary information

Supplement Material 1

Supplement Material 2

## Data Availability

The datasets generated during and/or analysed during the current study are available in the Dataverse repository: [https://doi.org/10.7910/DVN/LOTR7C].
