# Supplementary material for: Prehistoric pottery styles of the Jutland Peninsula and their effect on human attention
Source: Humanit Soc Sci Commun. 2026 Aug 15;13(1):2027. doi: 10.1057/s41599-026-08601-1 (PMC13743490; doi:10.1057/s41599-026-08601-1)
Supplement: Supplementary file 1 [file 41599_2026_8601_MOESM1_ESM.pdf]

Catalog

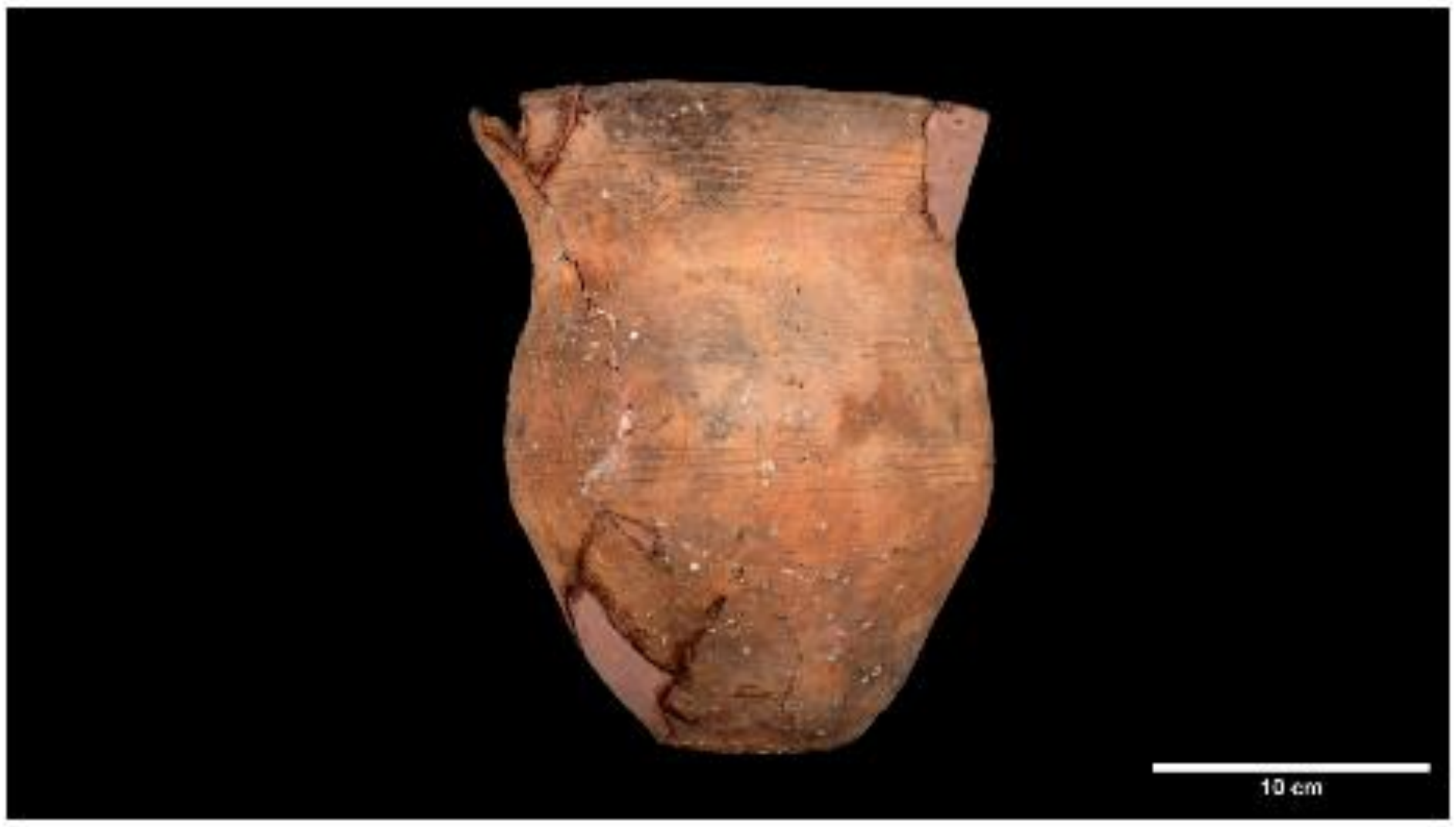

	Asset0059
**Museum Inventory N°**	FHM793-CC
**Site**	
**Context**	
**Chronology**	YN 1
**Absolute chronology**	2800 BCE - 2600 BCE
**Type**	Early Single Grave
**Phase**	11-12
**Phase absolute dates**	3000-2500 BCE

	Asset0059
**Decoration description**	Incised lines arranged in rows below the rim and the vessel body
**Shape description**	Beaker with flat base
**Height**	2.42
**Rim diameter**	19.8
**Base diameter**	8.2
**Reference for the typology**	Müller and Peterson 2014
**Reference for the asset**	

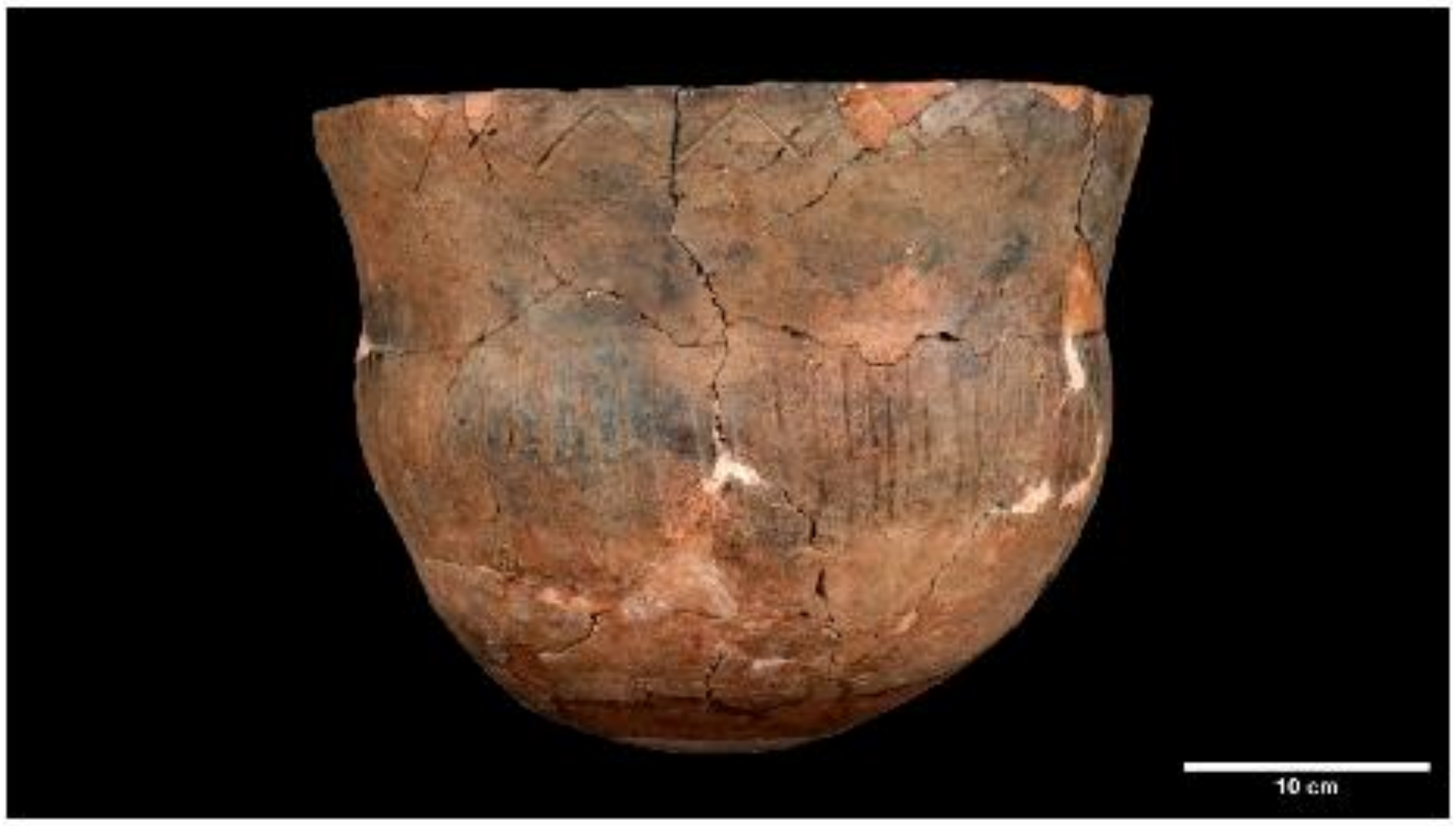

	Asset0060
**Museum Inventory N°**	1332
**Site**	Sarup, Denmark
**Context**	Ditch
**Chronology**	EN II
**Absolute chronology**	3500 BCE - 3300 BCE
**Type**	Sarup I - Fuchsberg
**Phase**	9
**Phase absolute dates**	3500-3250 BCE
**Decoration description**	Incised horizontally-oriented chevron pattern below the rim and incised vertical stripe patterns on the belly.
**Shape description**	Funnel-necked bowl with conical rim and rounded base
**Height**	27.2
**Rim diameter**	32.8
**Base diameter**	8.8
**Reference for the typology**	Müller and Peterson 2014; Andersen 1999; 1997
**Reference for the asset**	Müller and Peterson 2014; Andersen 1999; 1997

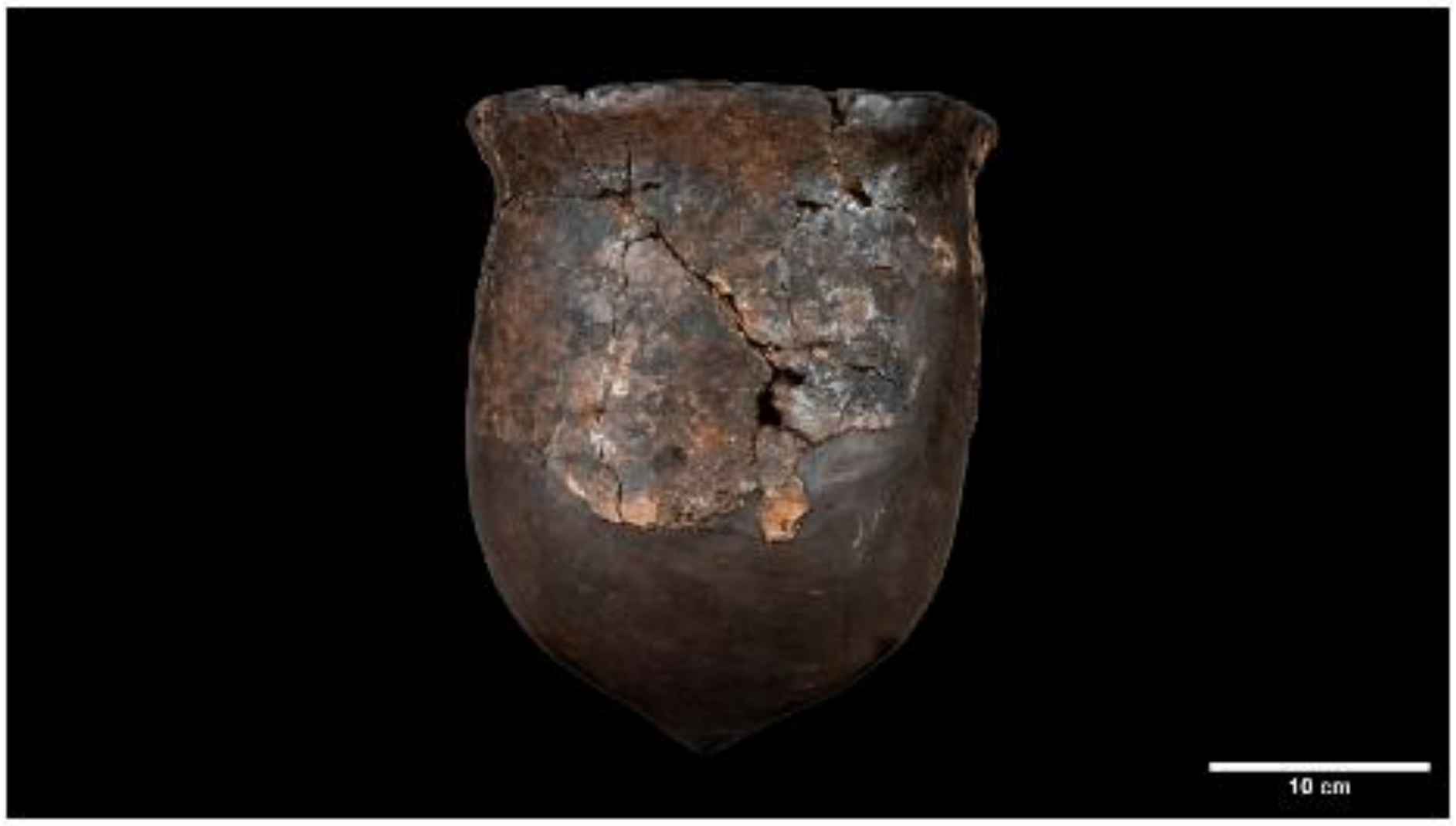

	Asset0061
**Museum Inventory N°**	1592BFMP
**Site**	
**Context**	
**Chronology**	Final Mesolithic
**Absolute chronology**	5400 BCE - 4000 BCE
**Type**	Ertebølle
**Phase**	4-7
**Phase absolute dates**	4750-3750 BCE

	Asset0061
**Decoration description**	Undecorated
**Shape description**	Funnel-necked vessel with pointed base
**Height**	30.5
**Rim diameter**	24.7
**Base diameter**	1.8
**Reference for the typology**	Sørensen 2015
**Reference for the asset**	Klassen 2004

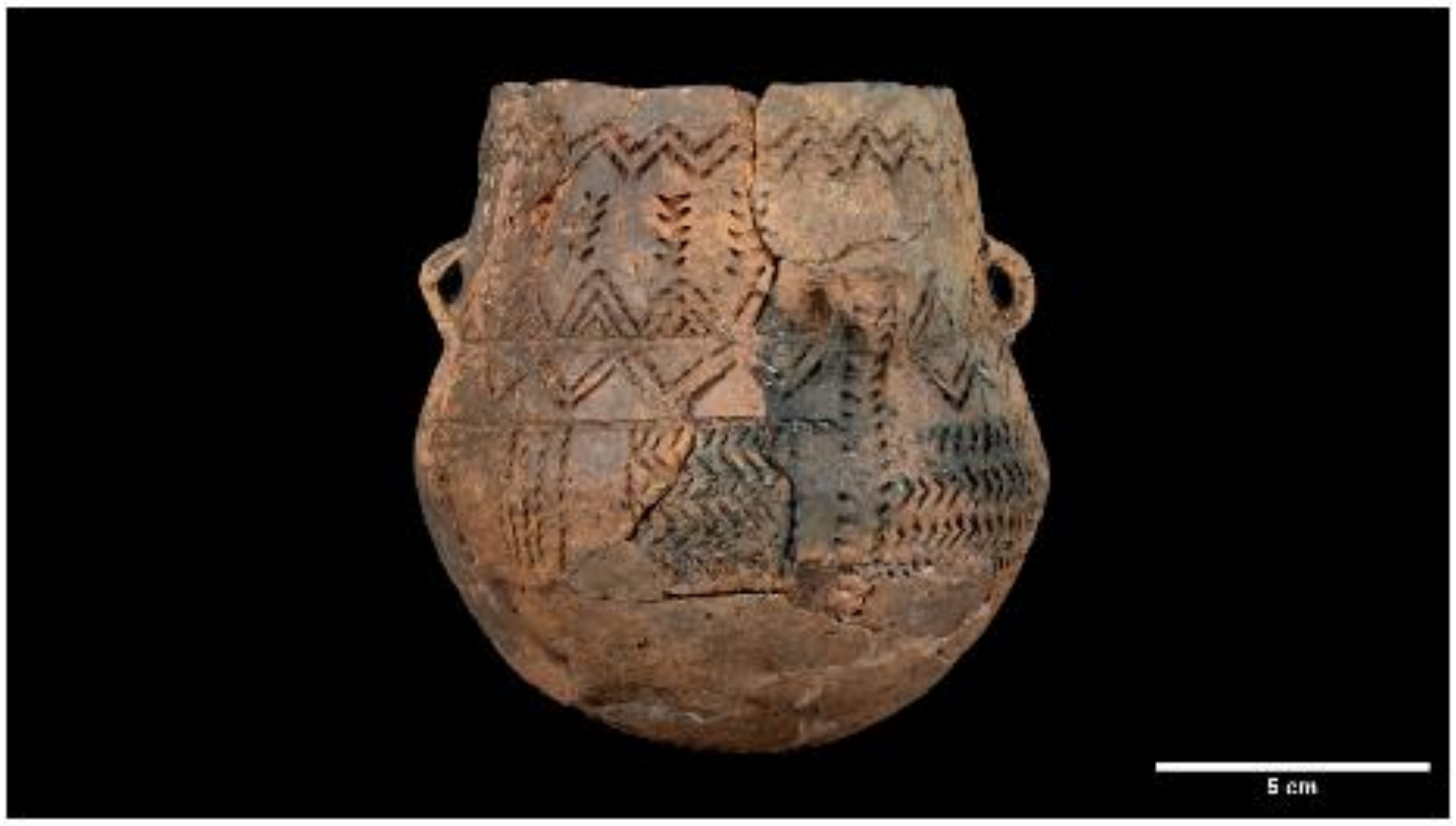

	Asset0062
**Museum Inventory N°**	X21712
**Site**	Sarup, Denmark
**Context**	
**Chronology**	MN II
**Absolute chronology**	3100 BCE - 3000 BCE
**Type**	Sarup III - Blandebjerg
**Phase**	10
**Phase absolute dates**	3250-3000 BCE
**Decoration description**	Several rows of stitched chevron bands below the vessel’s rim and neck, as well as a series of stitched angled stripes on the vessel’s neck and body. Several small vertically-oriented handles on the vessel’s neck are found decorated with stitched band patterns.
**Shape description**	Two-part jug with a closed conical neck and globular body
**Height**	12.2
**Rim diameter**	8.8
**Base diameter**	4.4
**Reference for the typology**	Müller and Peterson 2014; Andersen 1999; 1997
**Reference for the asset**	Andersen 1999

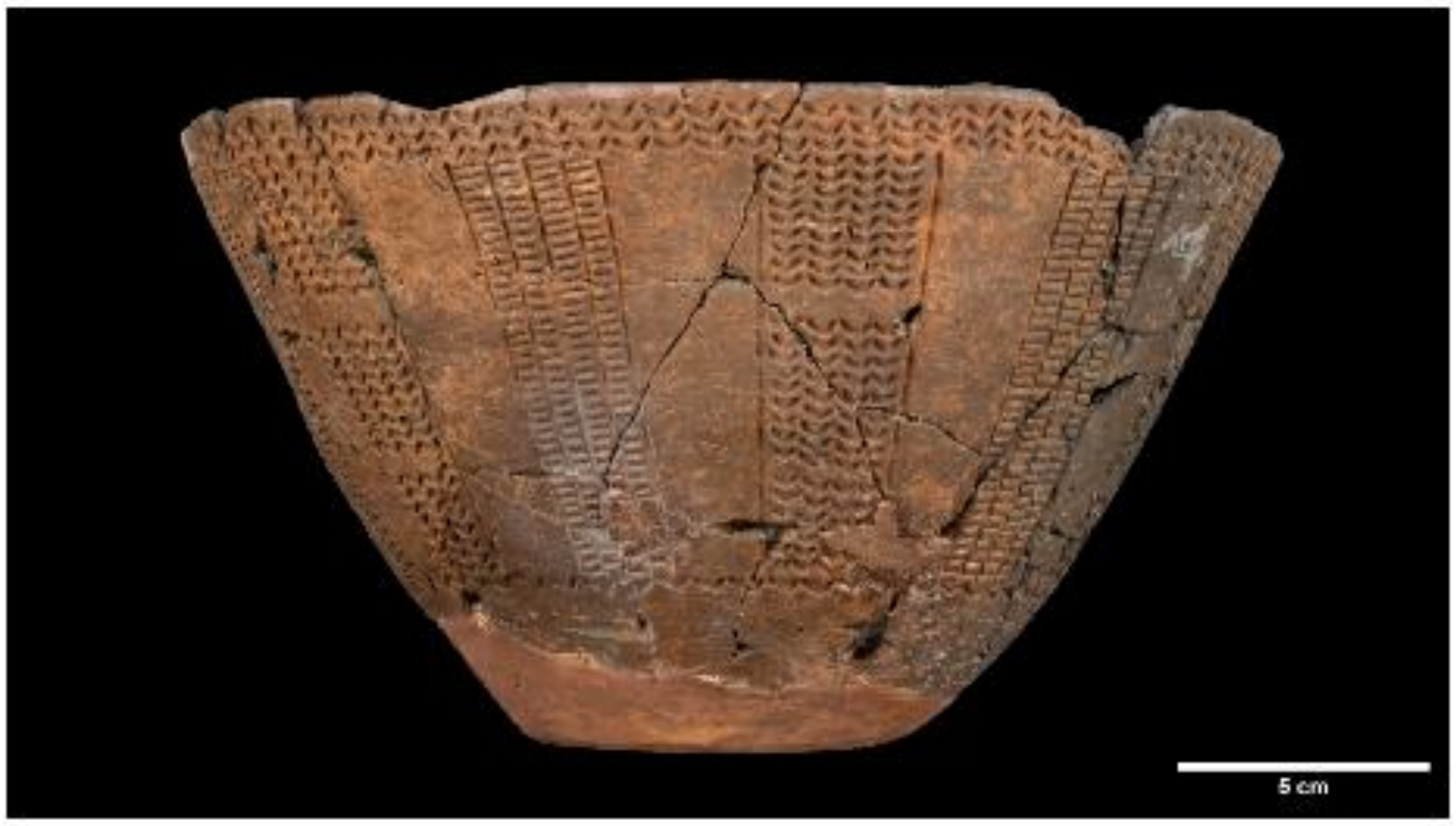

	Asset0063
**Museum Inventory N°**	X19279
**Site**	Sarup, Denmark
**Context**	
**Chronology**	MN II
**Absolute chronology**	3100 BCE - 3000 BCE
**Type**	Sarup III - Blandebjerg
**Phase**	10
**Phase absolute dates**	3250-3000 BCE
**Decoration description**	Three rows of stitched angled stripes below the vessel's rim perpendicular with a series of vertically oriented incised bands filled with horizontal or angled stitched stripes in the vessel's body.
**Shape description**	Funnel-shaped bowl with flat base
**Height**	13.4
**Rim diameter**	24
**Base diameter**	7.8
**Reference for the typology**	Müller and Peterson 2014; Andersen 1999; 1997
**Reference for the asset**	Andersen 1999

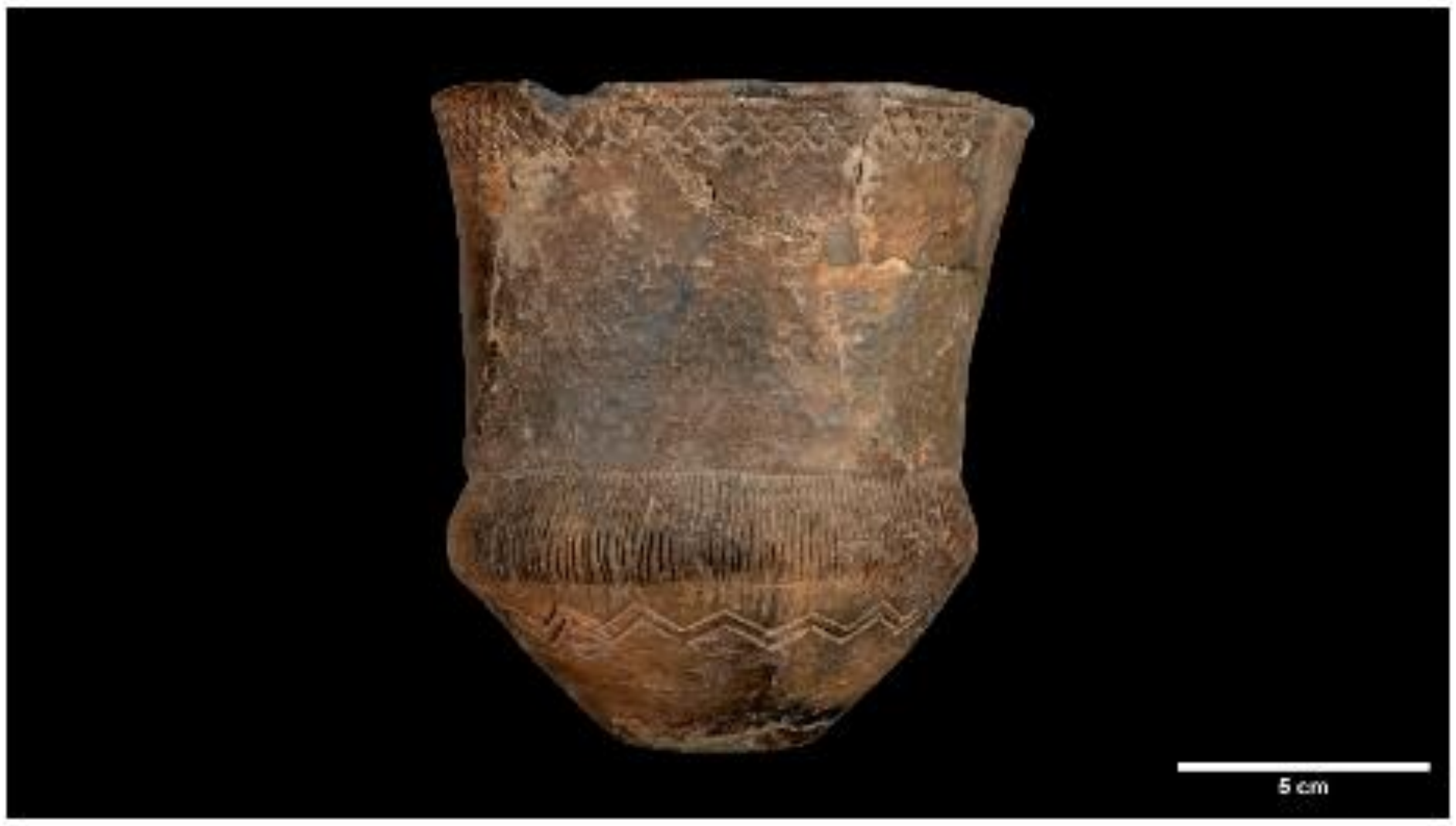

	Asset0064
**Museum Inventory N°**	AM2781
**Site**	Finderup, Northern Jutland
**Context**	
**Chronology**	MN Ib
**Absolute chronology**	3200 BCE - 3100 BCE
**Type**	Sarup II - Klintebakke
**Phase**	10
**Phase absolute dates**	3250-3000 BCE
**Decoration description**	Horizontal line and cross-hatched metopic pattern below the vessel’s rim, and a series of vertically-oriented incised stripes perpendicular with a chevron band on the vessel’s body.
**Shape description**	Funnel-necked beaker with flat base
**Height**	13.4
**Rim diameter**	12.4
**Base diameter**	3.9
**Reference for the typology**	Müller and Peterson 2014; Andersen 1999; 1997
**Reference for the asset**	Andersen 1999

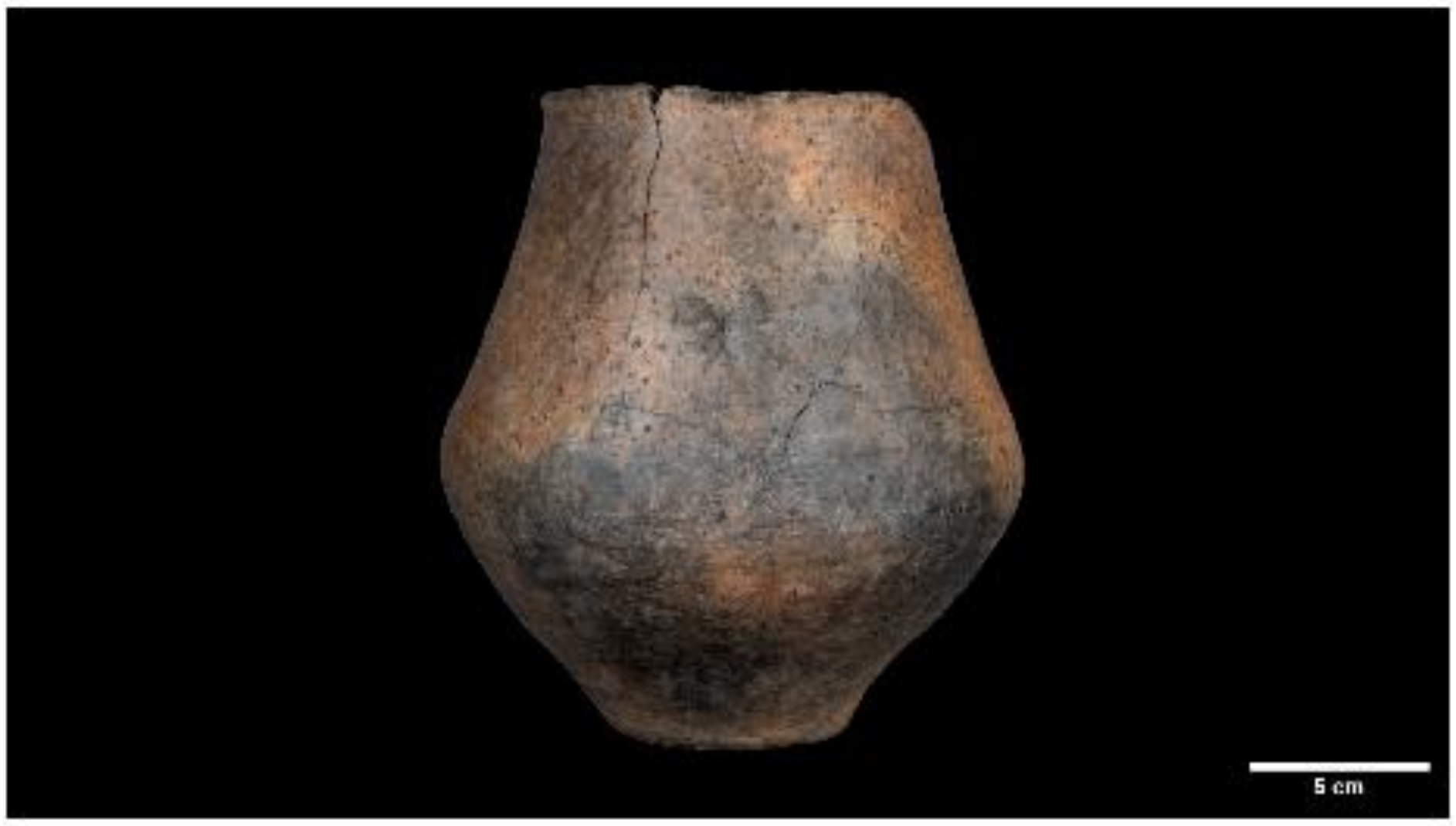

	Asset0065
**Museum Inventory N°**	AM0730
**Site**	
**Context**	Grave
**Chronology**	Period IV
**Absolute chronology**	1050 BCE - 900 BCE
**Type**	
**Phase**	18-19
**Phase absolute dates**	1250-750 BCE
**Decoration**	Face pattern formed through

	Asset0065
**description**	finger-pressing and pinching
**Shape description**	Two-part biconical urn
**Height**	18.1
**Rim diameter**	11.6
**Base diameter**	6.4
**Reference for the typology**	Jensen 2005; Lolk 2009
**Reference for the asset**	

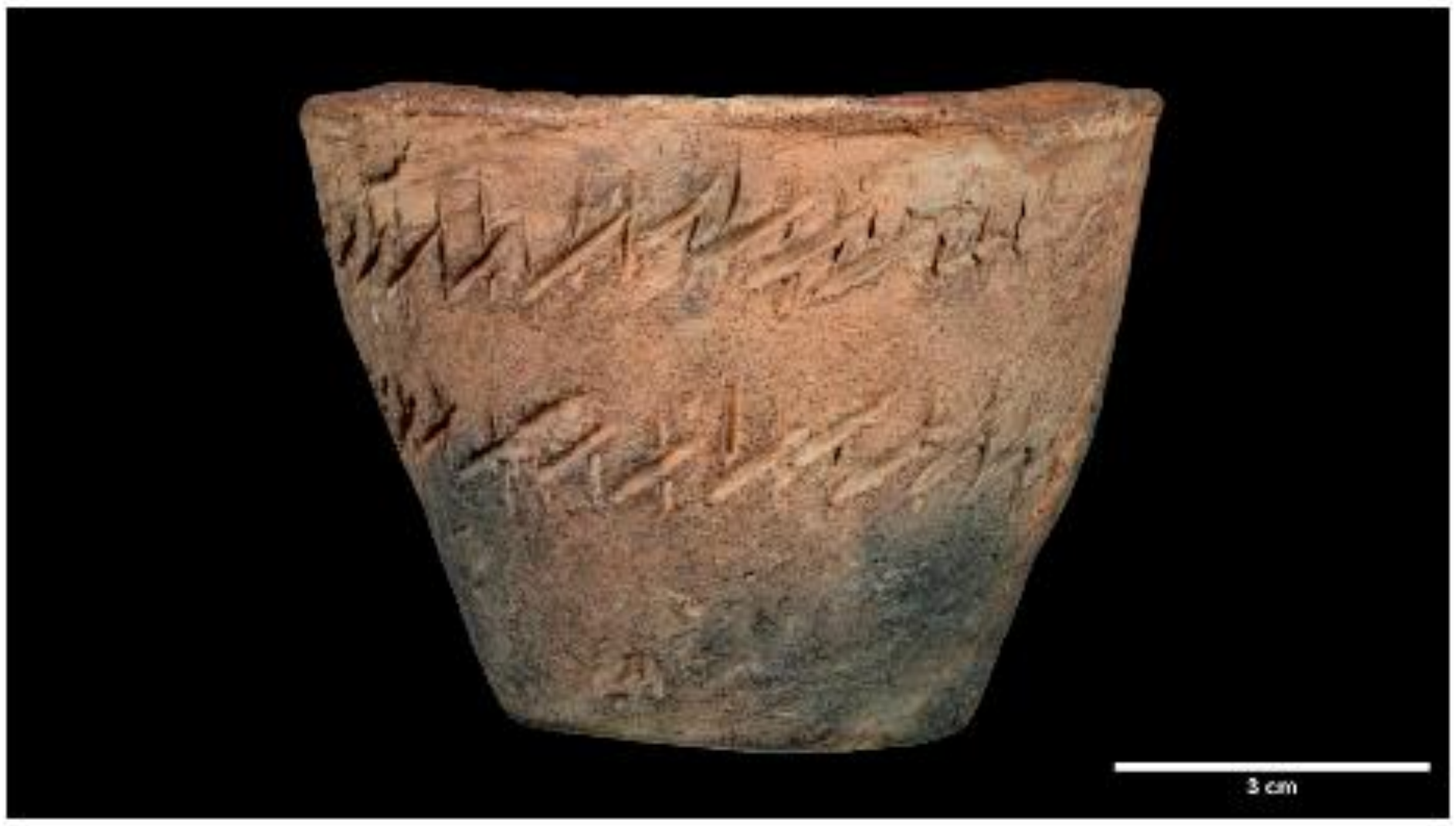

	Asset0066
**Museum Inventory N°**	AM2029
**Site**	Vorhøj Hammel sogn, Northern Jutland
**Context**	Burial Mound|Grave
**Chronology**	LN 2
**Absolute chronology**	1900 BCE - 1700 BCE
**Type**	
**Phase**	15-16
**Phase absolute dates**	2000-1500 BCE
**Decoration description**	Two horizontal incised cross-hatched bands in the vessel’s body
**Shape description**	V-shaped bowl with flat base
**Height**	6.2
**Rim diameter**	8.8
**Base diameter**	4.4
**Reference for the typology**	Müller and Peterson 2014; Van Kilde 2005
**Reference for the asset**	

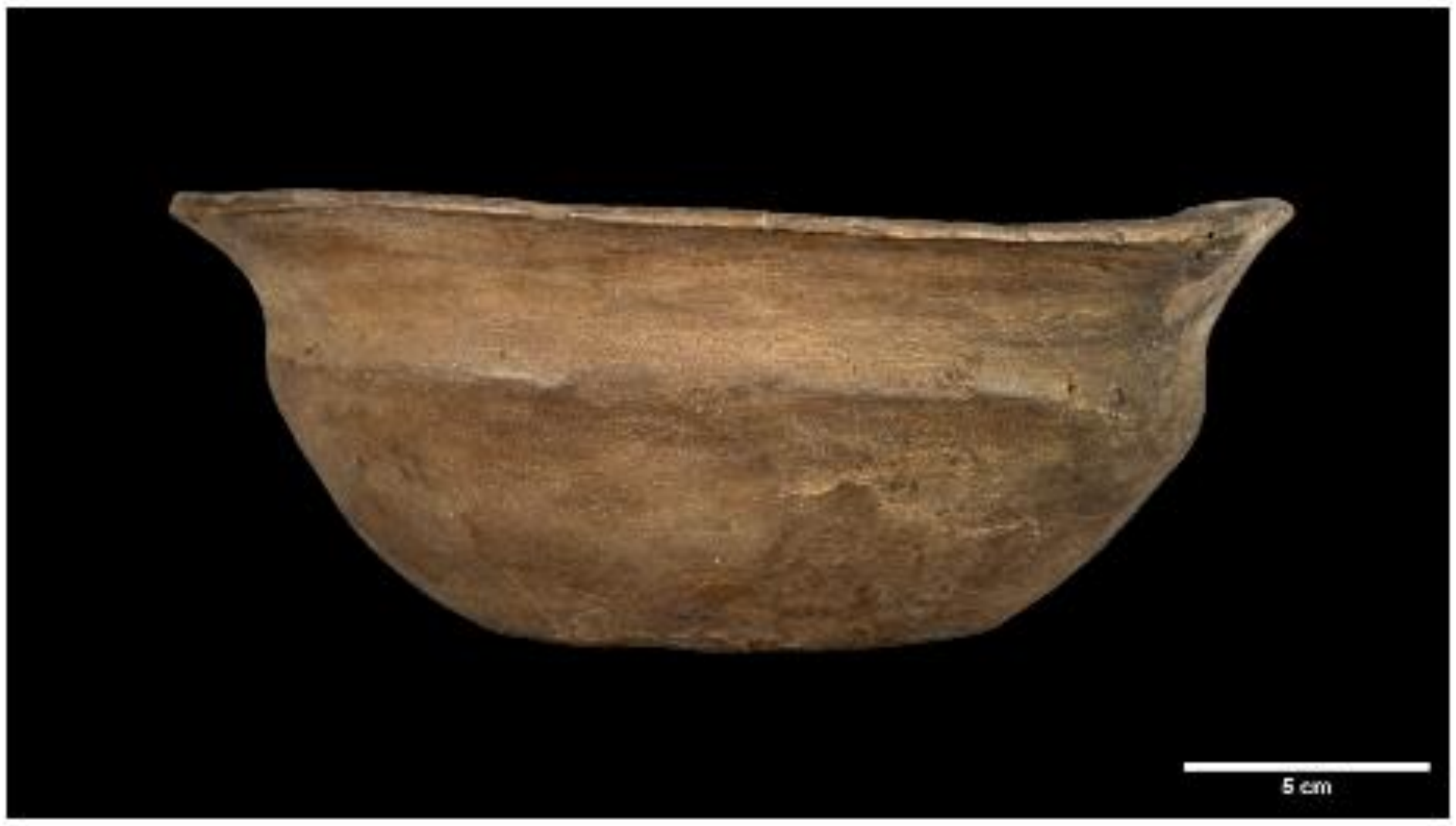

	Asset0067
**Museum Inventory N°**	AM2796
**Site**	Aabenrø, Northern Jutland
**Context**	Megalithic monument
**Chronology**	YN 2
**Absolute chronology**	2600 BCE - 2400 BCE
**Type**	Middle Single Grave
**Phase**	12-13
**Phase absolute dates**	2750-2250 BCE
**Decoration**	Undecorated

	Asset0067
**description**	
**Shape description**	Bowl with s-shaped profile and flat base
**Height**	9
**Rim diameter**	20
**Base diameter**	8.5
**Reference for the typology**	Müller and Peterson 2014
**Reference for the asset**	

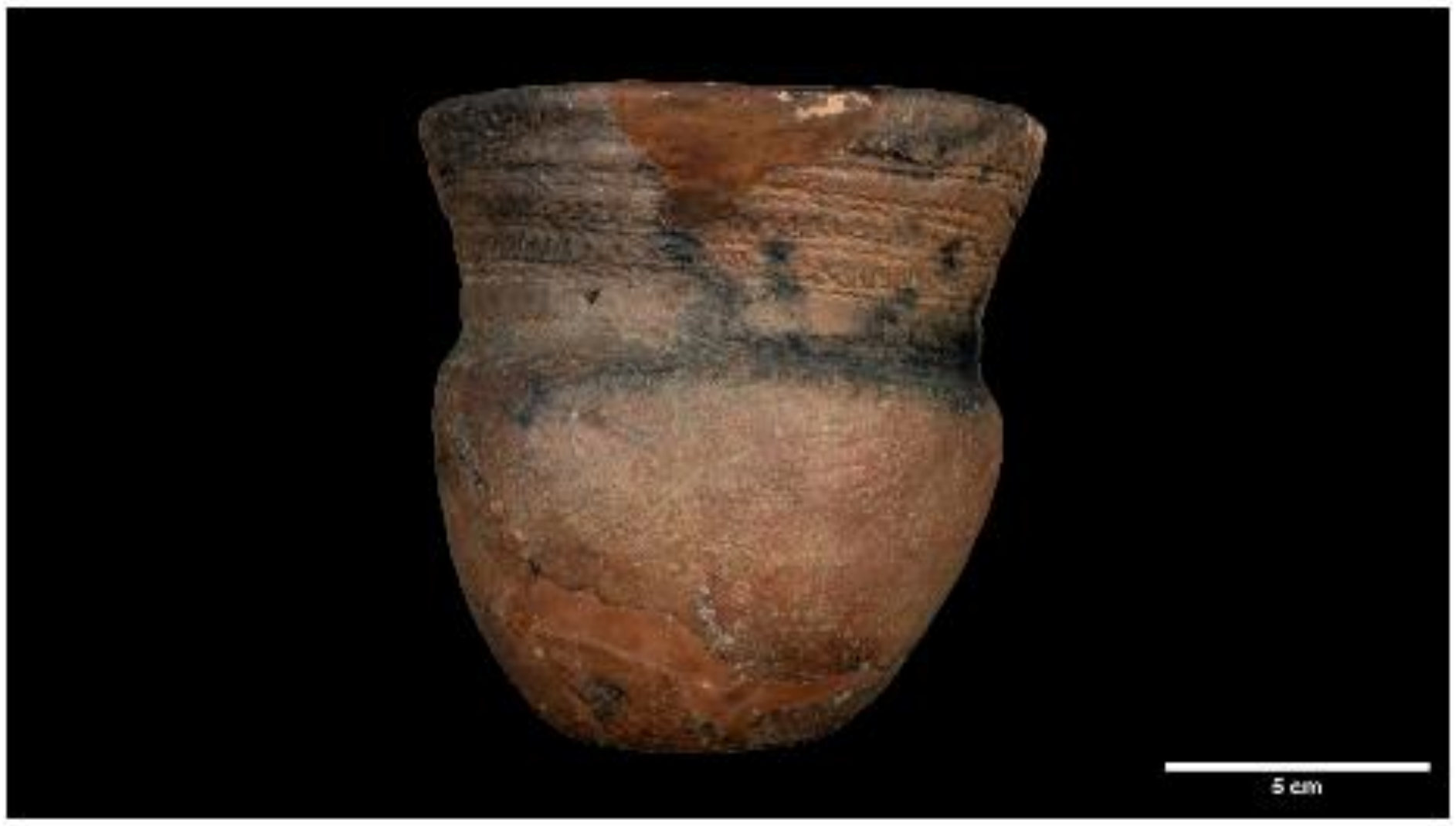

	Asset0068
**Museum Inventory N°**	AM2796
**Site**	
**Context**	Grave
**Chronology**	YN 1
**Absolute chronology**	2800 BCE - 2600 BCE
**Type**	Early Single Grave
**Phase**	11-12
**Phase absolute dates**	3000-2500 BCE

	Asset0068
**Decoration description**	Multiple rows of corded impressions on vessel's neck
**Shape description**	Beaker with open conical neck and flat base
**Height**	12.7
**Rim diameter**	12.4
**Base diameter**	4.9
**Reference for the typology**	Müller and Peterson 2014
**Reference for the asset**	

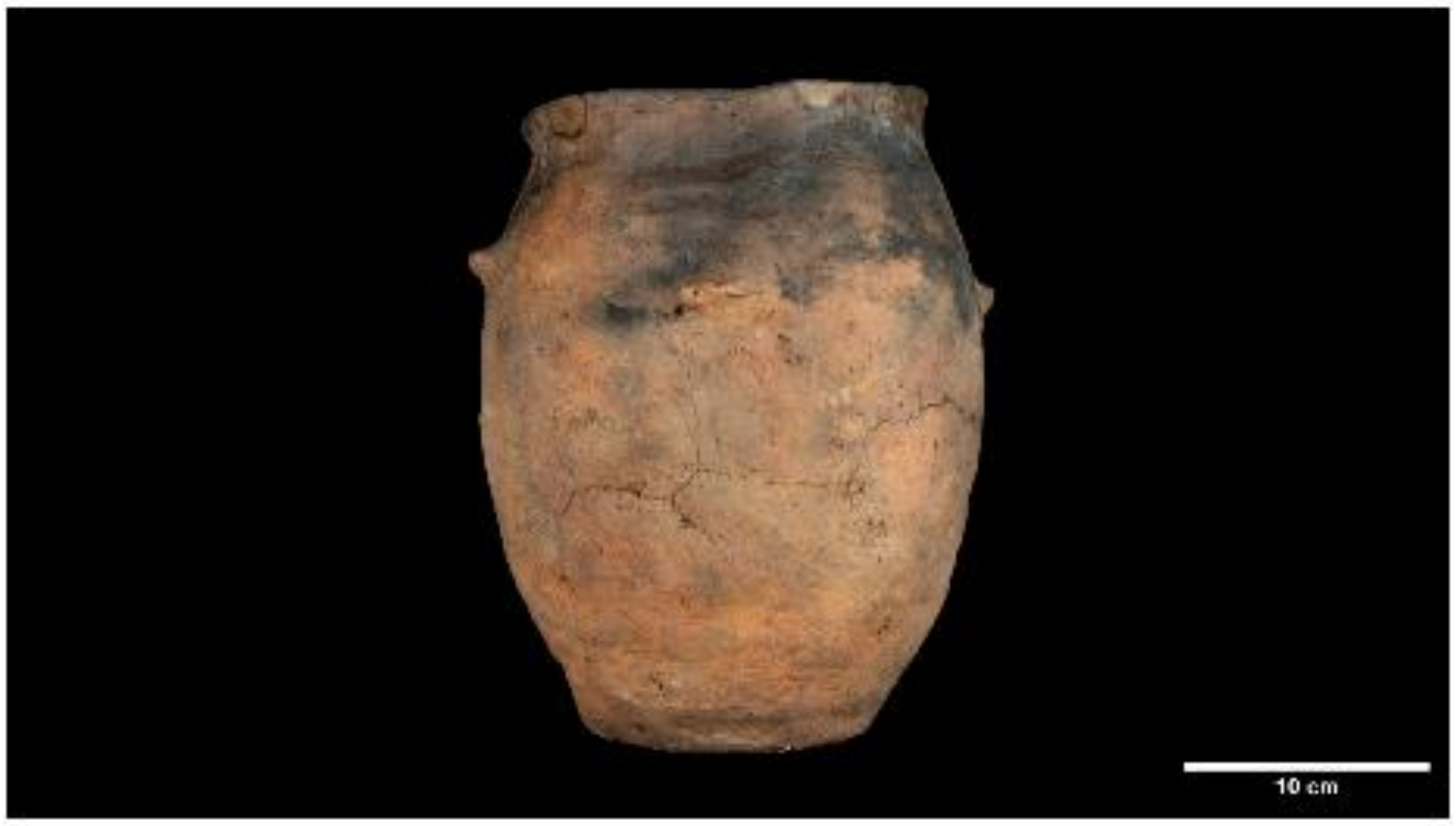

	Asset0069
**Museum Inventory N°**	AM3389
**Site**	
**Context**	Burial Mound|Grave
**Chronology**	Period V/VI
**Absolute chronology**	900 BCE - 700 BCE | 700 BCE - 570 BCE
**Type**	
**Phase**	19-20
**Phase absolute dates**	1000-500 BCE

	Asset0069
**Decoration description**	Multiple modelled flat handles at the vessel's upper body
**Shape description**	Tripartite slightly rounded vessel with outward rim
**Height**	27.1
**Rim diameter**	18.2
**Base diameter**	12
**Reference for the typology**	Jensen 2005; Lolk 2009
**Reference for the asset**	

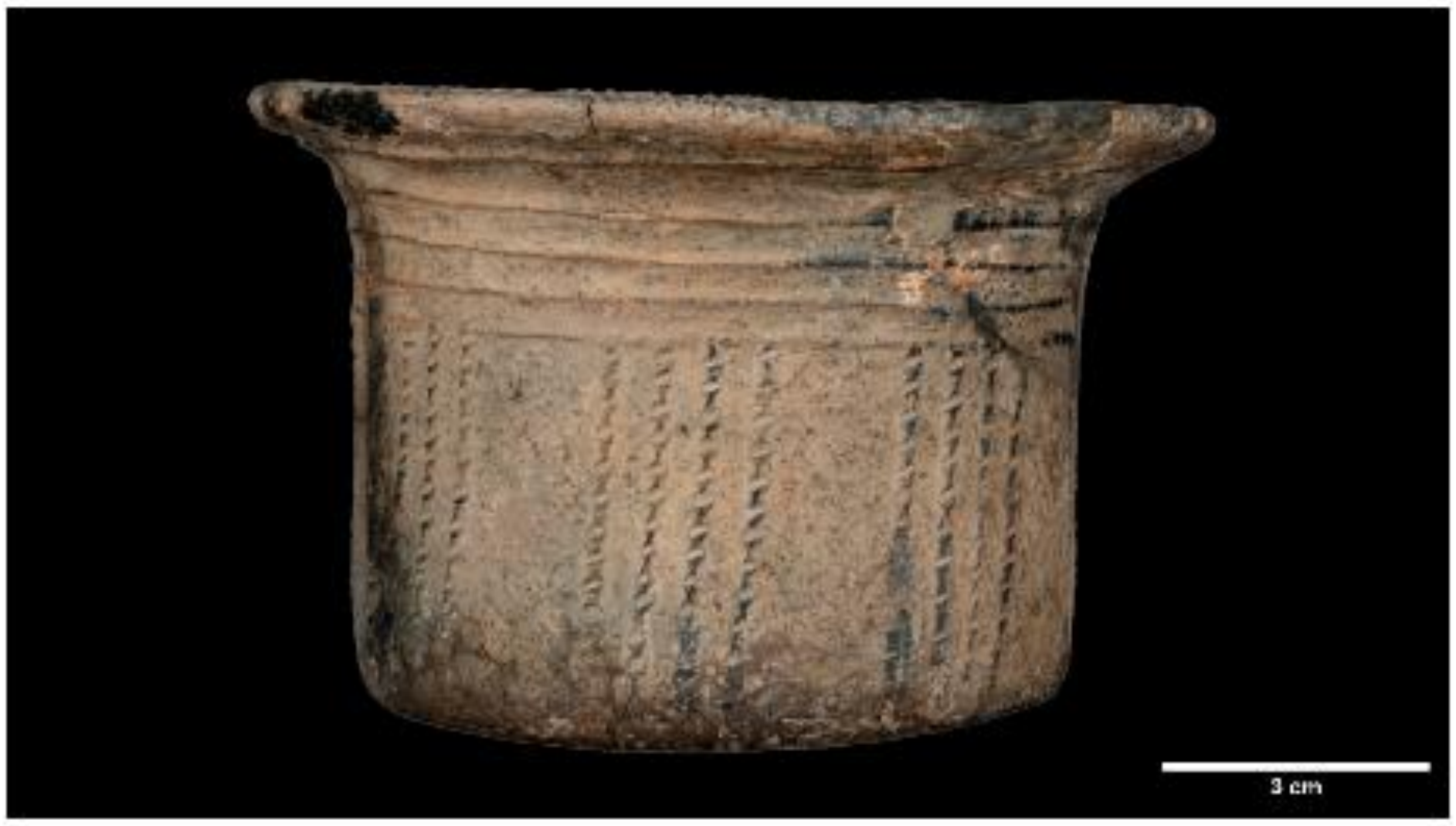

	Asset0070
**Museum Inventory N°**	AM3680
**Site**	
**Context**	Grave
**Chronology**	YN 3
**Absolute chronology**	2400 BCE - 2200 BCE
**Type**	Late Single Grave
**Phase**	13-14
**Phase absolute dates**	2500-2000 BCE
**Decoration description**	A series of moulded horizontal stripe patterns below the vessel’s rim and vertically-oriented waves
**Shape description**	Straight-walled beaker with everted rim and flat base
**Height**	7.4
**Rim diameter**	12.5
**Base diameter**	9
**Reference for the typology**	Müller and Peterson 2014
**Reference for the asset**	

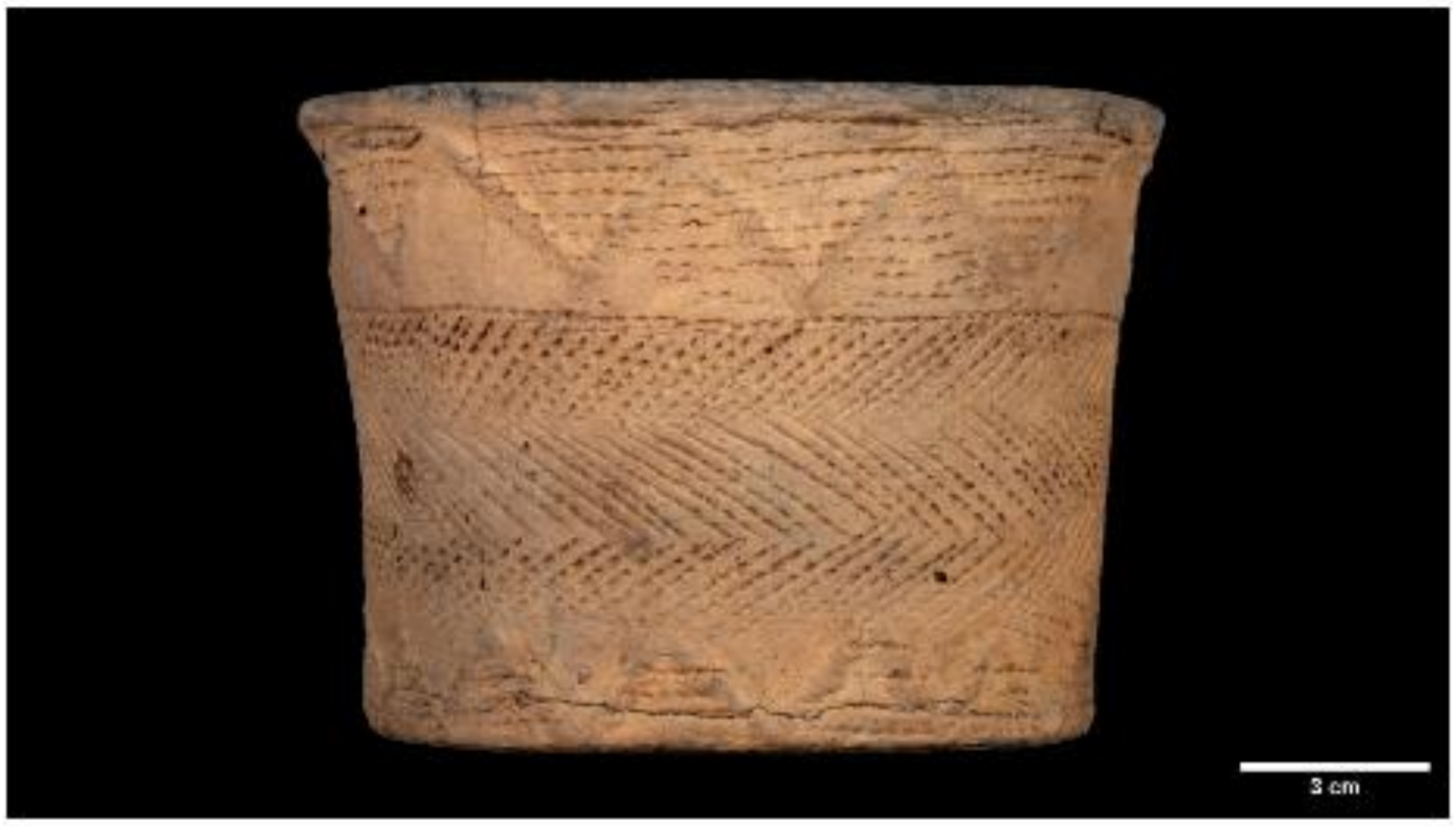

	Asset0071
**Museum Inventory N°**	AM5184
**Site**	
**Context**	Grave
**Chronology**	YN 3
**Absolute chronology**	2400 BCE - 2200 BCE
**Type**	Late Single Grave
**Phase**	13-14
**Phase absolute dates**	2500-2000 BCE
**Decoration description**	A series if moulded wave-like patterns superposed on moulded chevron impressions below the vessel's rim and above its base
**Shape description**	Straight-walled beaker with flat base
**Height**	10.7
**Rim diameter**	14.5
**Base diameter**	12.3
**Reference for the typology**	Müller and Peterson 2014
**Reference for the asset**	

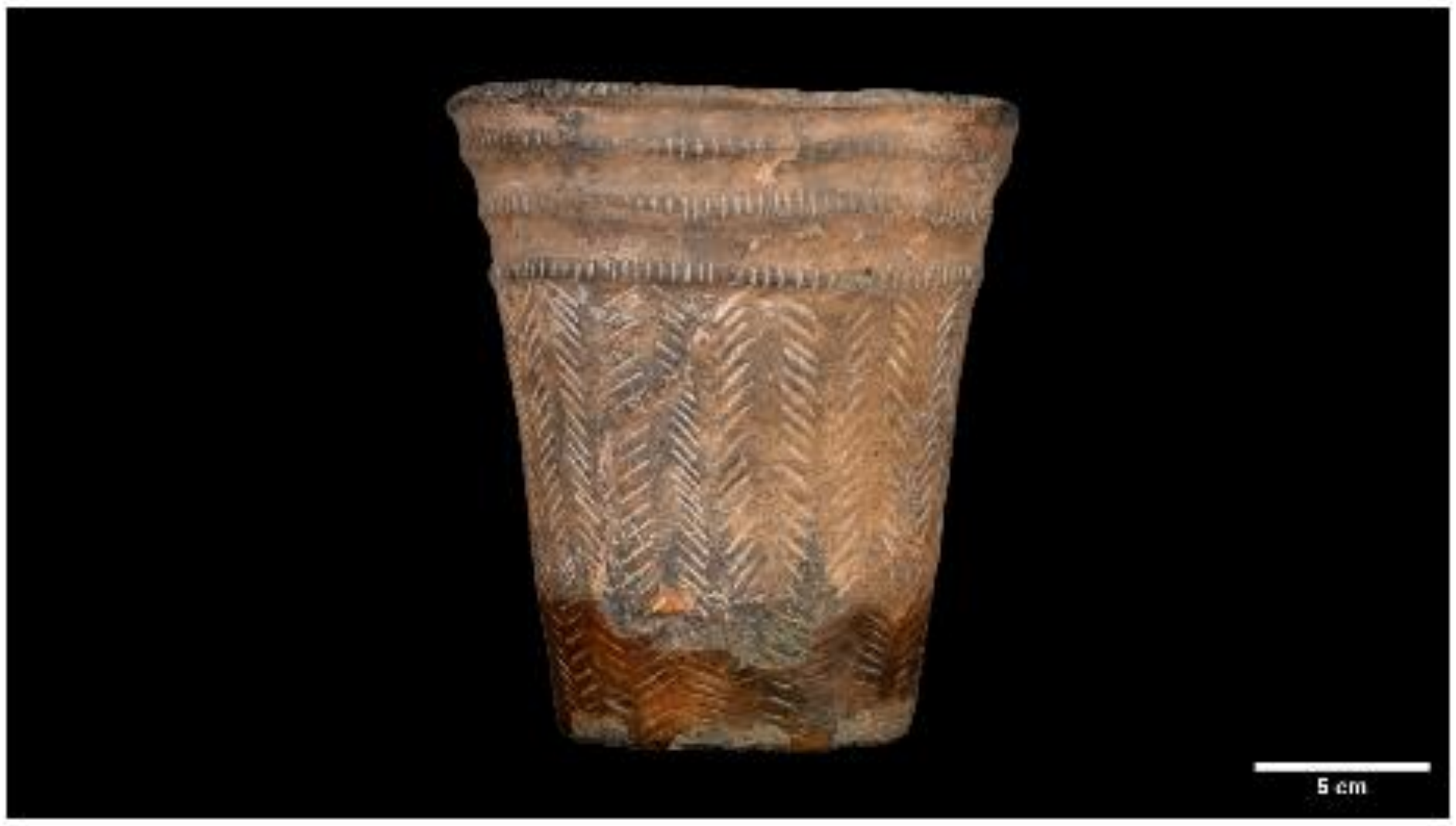

	Asset0072
**Museum Inventory N°**	AM5185
**Site**	
**Context**	Grave
**Chronology**	LN 1 | YN 3
**Absolute chronology**	2200 BCE - 1900 BCE | 2400 BCE - 2200 BCE
**Type**	Late Single Grave
**Phase**	13-14
**Phase absolute dates**	2500-2000 BCE
**Decoration description**	Vertical incised stripes in the vessel’s rim., three rows of vertical incised stripes, and a series of vertical bands composed of oblique incised stripes (fishbone-like pattern) in the vessel’s body.
**Shape description**	Slightly outwardly titled straight-walled beaker with flat base
**Height**	19.1
**Rim diameter**	17.3
**Base diameter**	10.8
**Reference for the typology**	Müller and Peterson 2014; Van Kilde 2005
**Reference for the asset**	

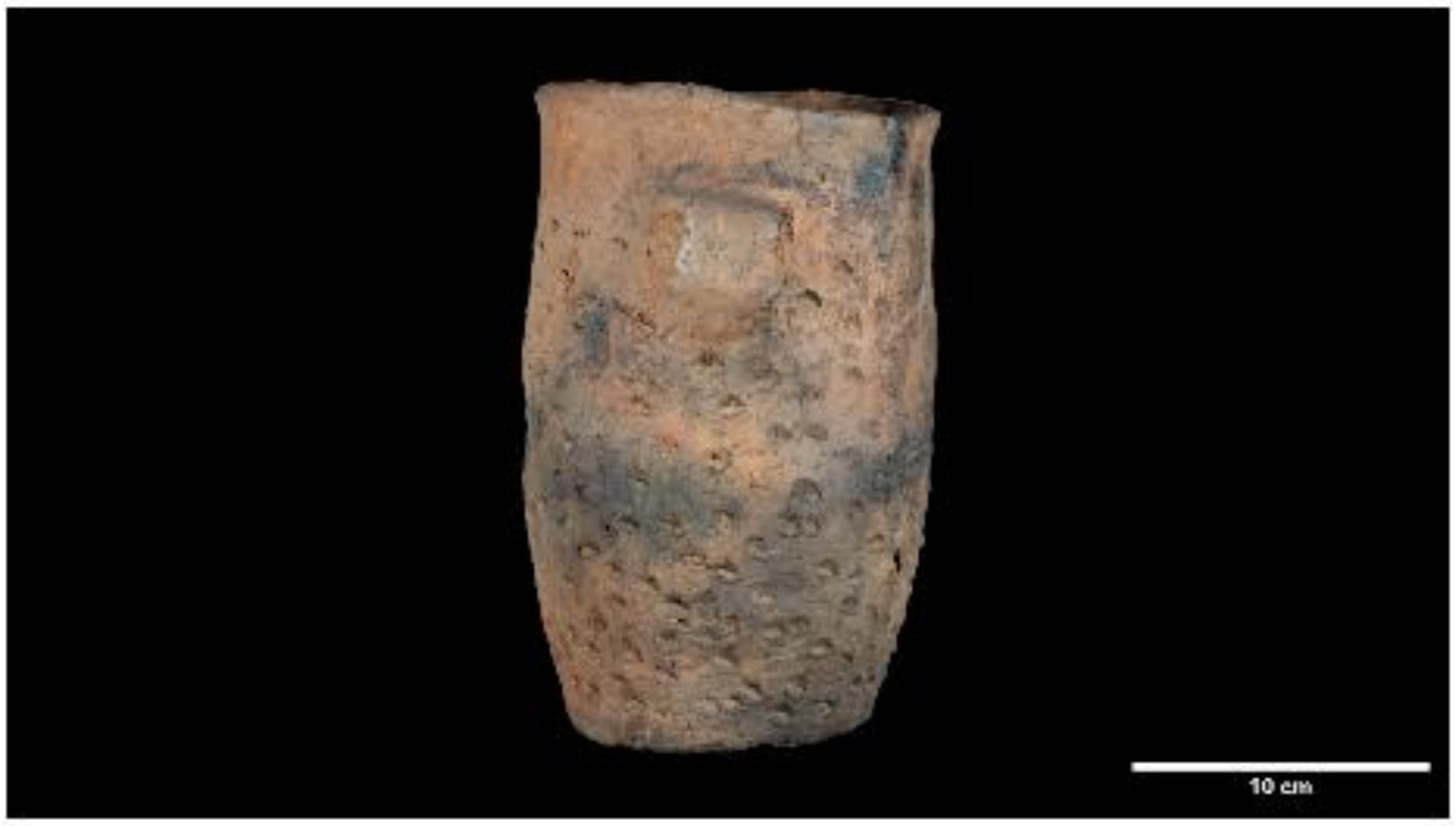

	Asset0073
**Museum Inventory N°**	AM6077
**Site**	
**Context**	Burial Mound|Grave
**Chronology**	Period IV
**Absolute chronology**	1050 BCE - 900 BCE
**Type**	
**Phase**	18-19
**Phase absolute dates**	1250-750 BCE
**Decoration description**	A scatter of fingernail impressions along the vessel's body, and a vertically oriented handle on the vessel's upper body.
**Shape description**	Cylindrical urn with flat base
**Height**	22.3
**Rim diameter**	13.1
**Base diameter**	10.5
**Reference for the typology**	Jensen 2005; Lolk 2009
**Reference for the asset**	

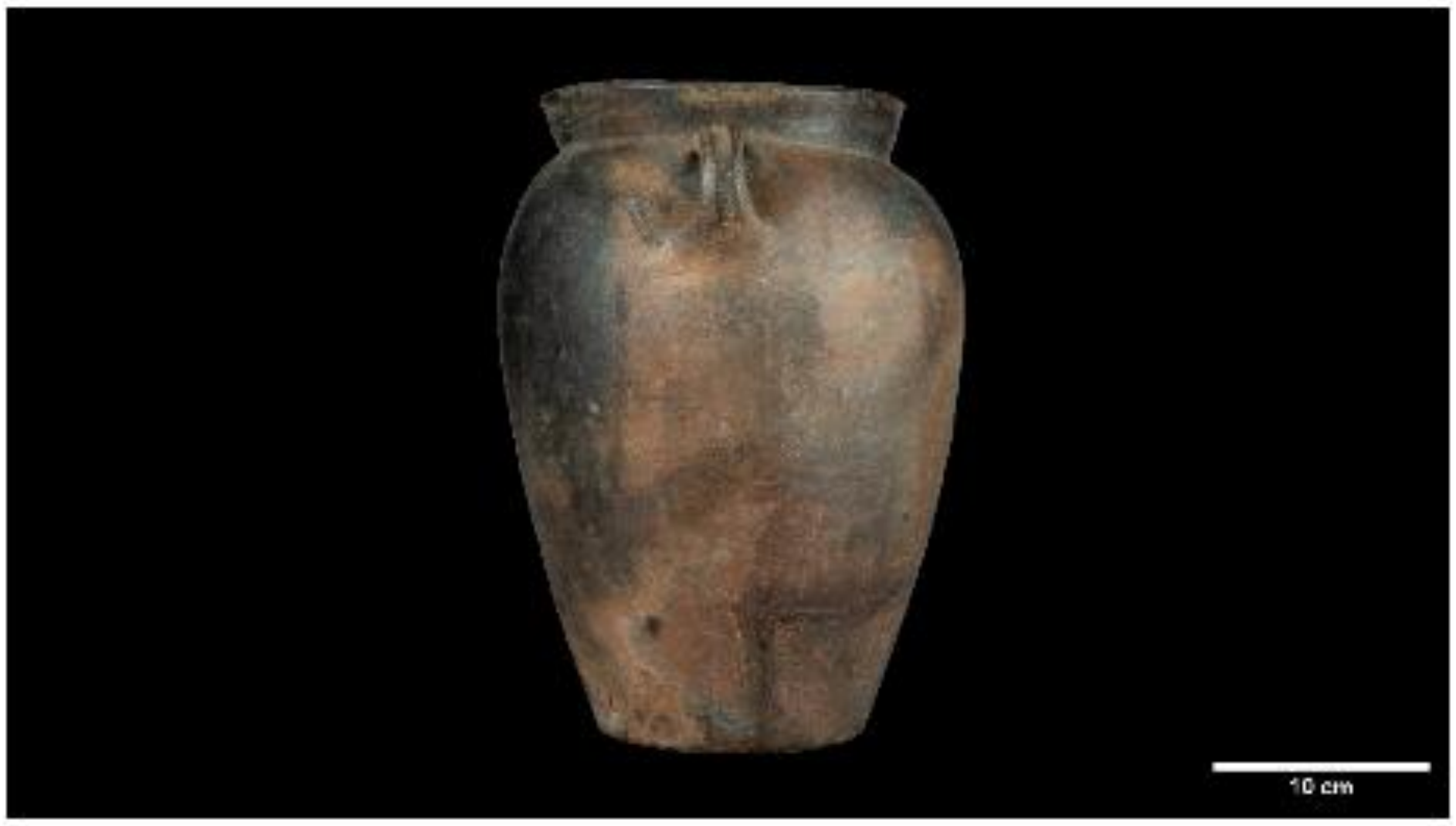

	Asset0074
**Museum Inventory N°**	AM6175
**Site**	
**Context**	
**Chronology**	Iron Age
**Absolute chronology**	570 BCE - 10
**Type**	PRIA II
**Phase**	22
**Phase absolute dates**	250-1 BCE
**Decoration**	Vertically oriented handle

	Asset0074
**description**	on the vessel's shoulder
**Shape description**	Elongated vessel with a collar-like rim.
**Height**	31.2
**Rim diameter**	17.5
**Base diameter**	13.7
**Reference for the typology**	Jensen 2005; Martens 1992
**Reference for the asset**	

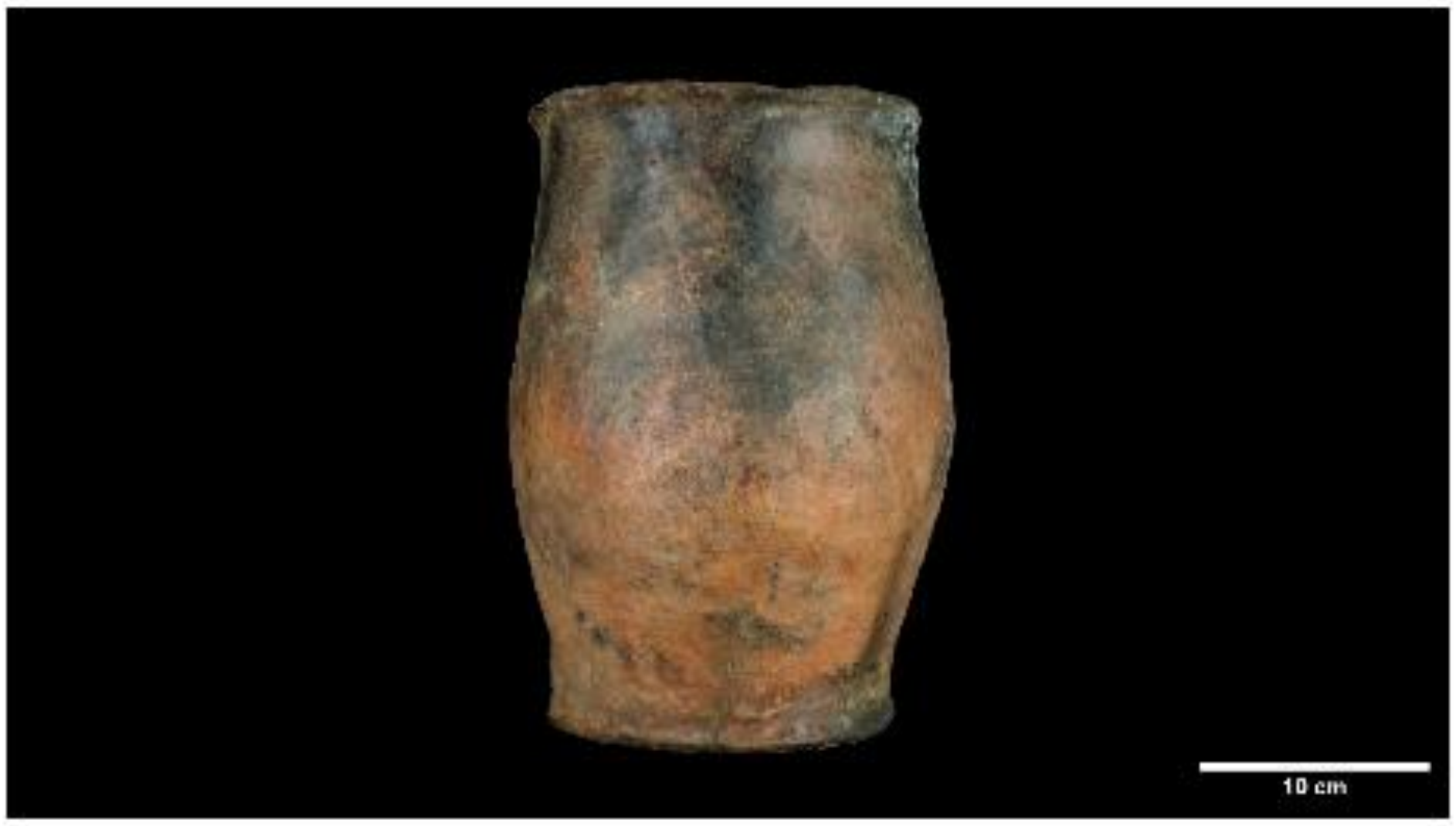

	Asset0075
**Museum Inventory N°**	AM6332a
**Site**	
**Context**	Burial Mound|Grave
**Chronology**	Period III | Period IV
**Absolute chronology**	1300 BCE - 1050 BCE | 1050 BCE - 900 BCE
**Type**	
**Phase**	17-19
**Phase absolute dates**	1500-750 BCE

	Asset0075
**Decoration description**	Undecorated
**Shape description**	Elongated urn with slightly-rounded body
**Height**	29.3
**Rim diameter**	18.6
**Base diameter**	15.2
**Reference for the typology**	Jensen 2005; Lolk 2009
**Reference for the asset**	Aner, Kersten, Willroth 2014, nr.6433

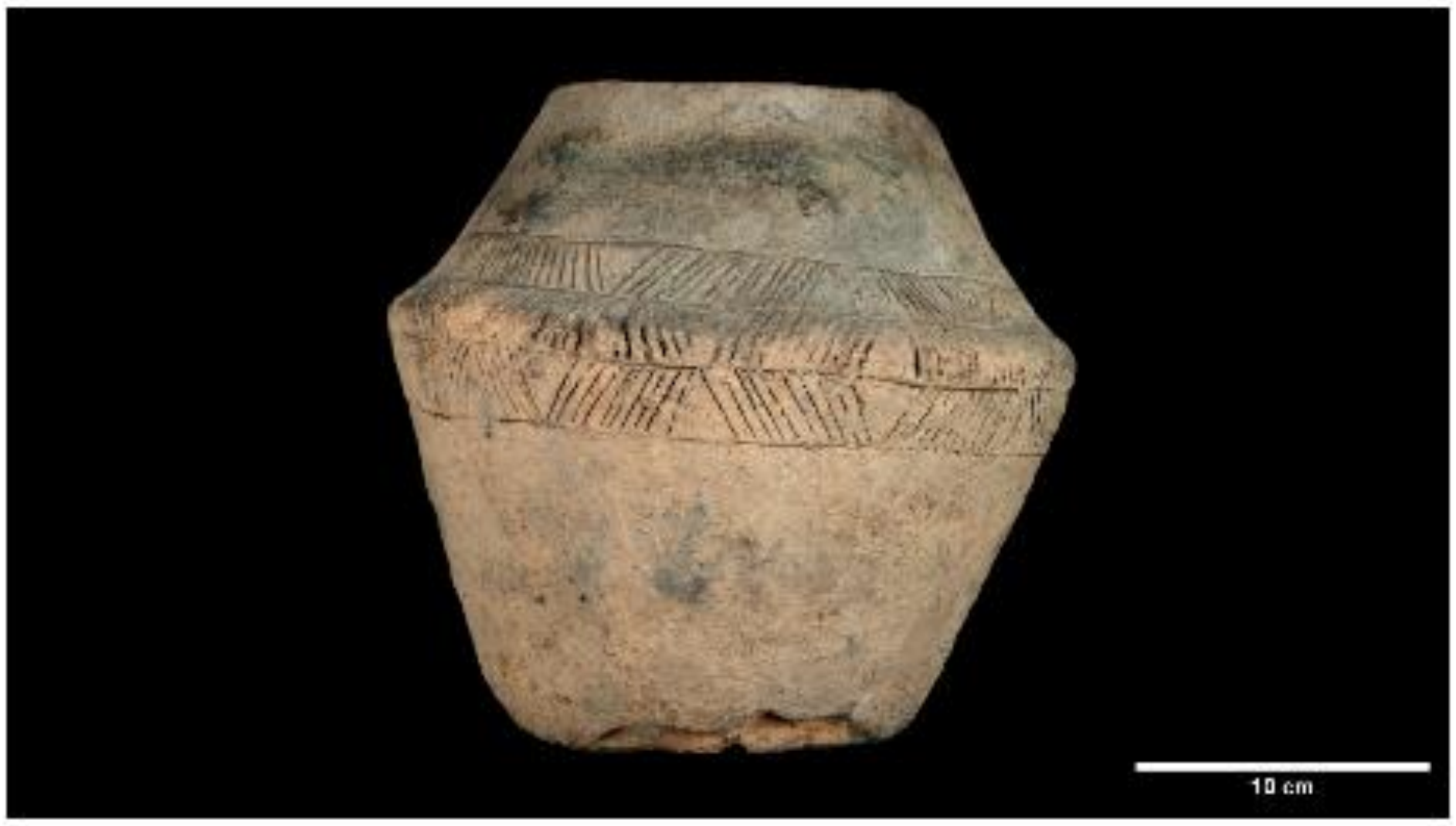

	Asset0076
**Museum Inventory N°**	AM6343
**Site**	
**Context**	
**Chronology**	Period V
**Absolute chronology**	900 BCE - 700 BCE
**Type**	
**Phase**	19-20
**Phase absolute dates**	1000-500 BCE
**Decoration description**	Three incised horizontal bands filled with angled stripe patterns at the inflexion point between vessel’s neck and body
**Shape description**	Two-part biconical vessel
**Height**	22.8
**Rim diameter**	14.2
**Base diameter**	14.3
**Reference for the typology**	Jensen 2005; Lolk 2009
**Reference for the asset**	

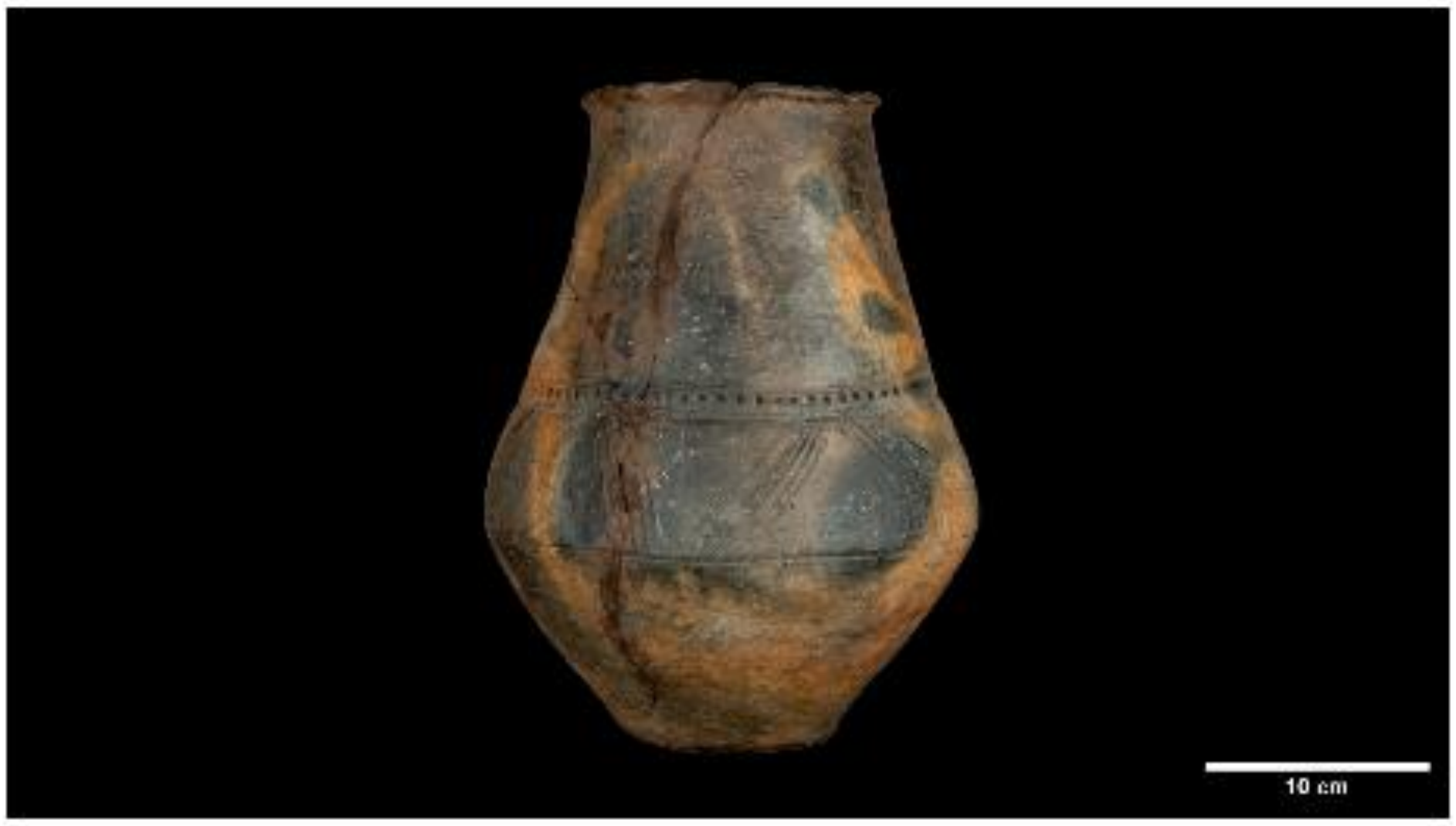

	Asset0077
**Museum Inventory N°**	AM6553
**Site**	
**Context**	Single find
**Chronology**	Period V
**Absolute chronology**	900 BCE - 700 BCE
**Type**	
**Phase**	19-20
**Phase absolute dates**	1000-500 BCE
**Decoration description**	Horizontal incised thin band filled with impressed dots and a large horizontal incised band

	Asset0077
	filled with an angled stripe pattern on the vessel's body.
**Shape description**	Elongated urn with long closed conical neck, a short rounded body and flat base
**Height**	29.5
**Rim diameter**	13.5
**Base diameter**	9.7
**Reference for the typology**	Jensen 2005; Lolk 2009
**Reference for the asset**	

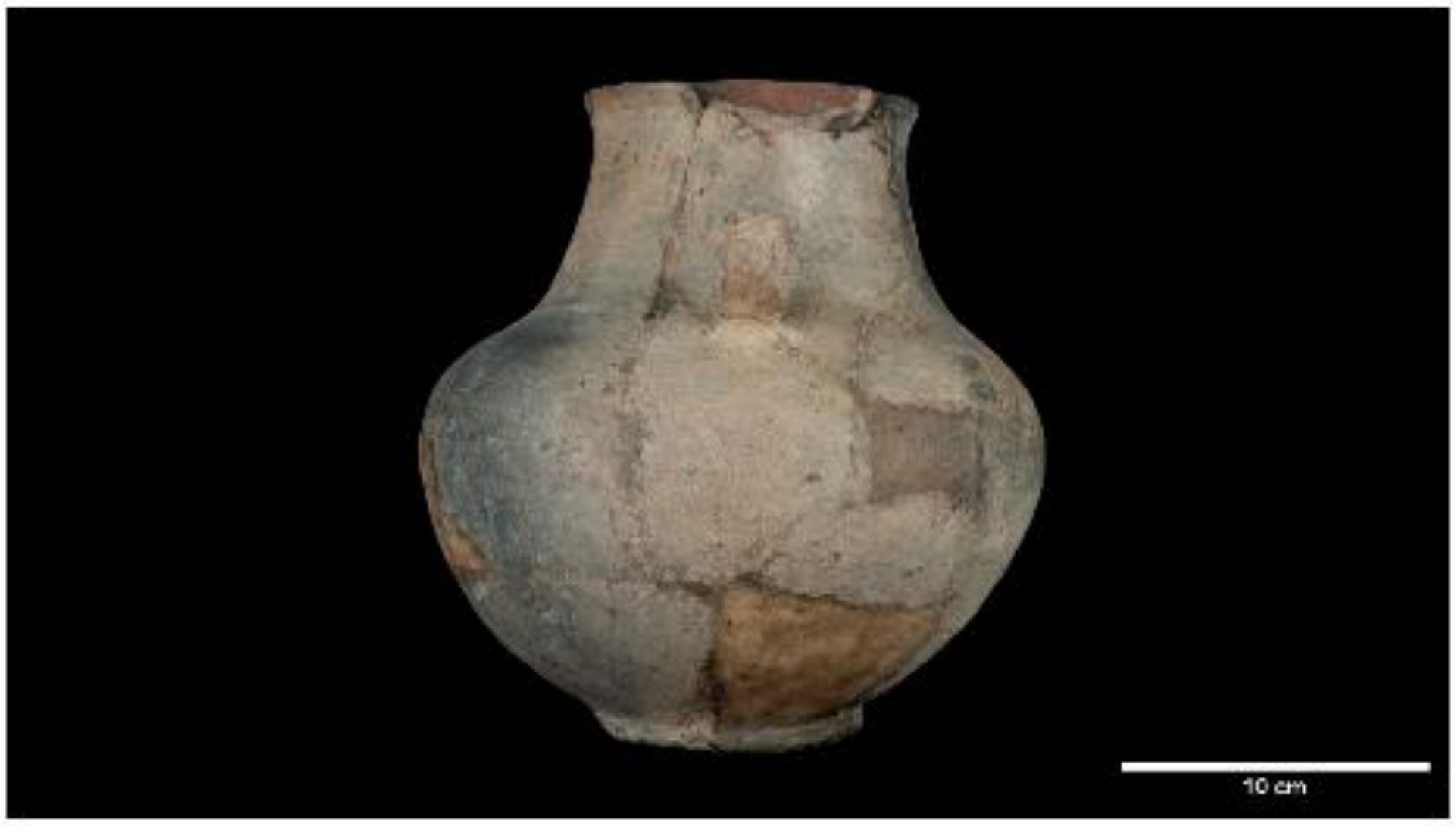

	Asset0078
**Museum Inventory N°**	AM7737
**Site**	
**Context**	Single find
**Chronology**	Iron Age II
**Absolute chronology**	250 BCE - 10
**Type**	PRIA II
**Phase**	22
**Phase absolute dates**	250-1 BCE
**Decoration description**	Vertically oriented handles at the inflexion point between vessel’s neck and body
**Shape description**	Three-part vessel with a broad, slightly kinked rim, globular body and flat base
**Height**	21.8
**Rim diameter**	11.9
**Base diameter**	8.9
**Reference for the typology**	Jensen 2005; Martens 1992
**Reference for the asset**	

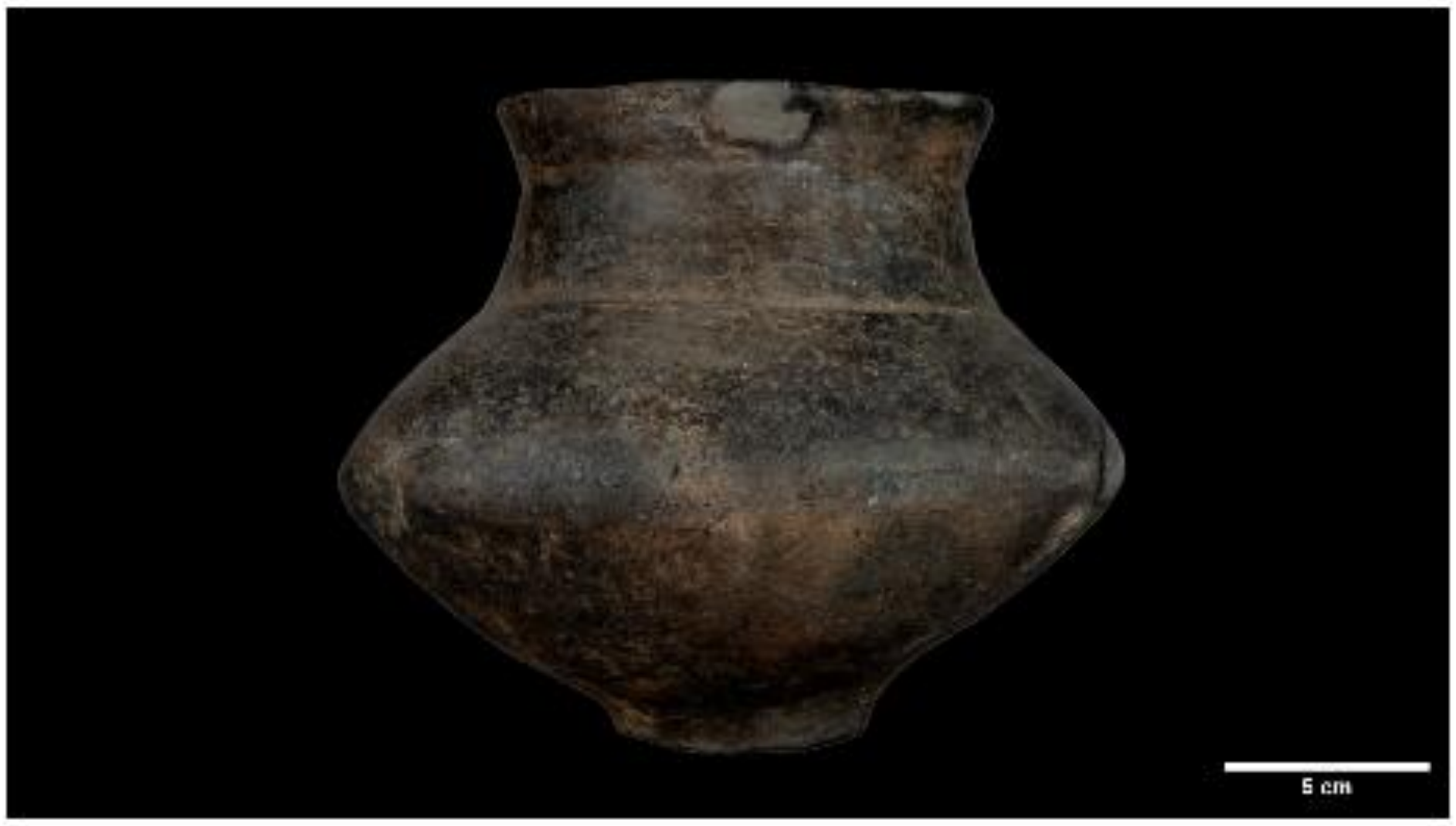

	Asset0079
**Museum Inventory N°**	AM8160
**Site**	
**Context**	Single find
**Chronology**	Iron Age I
**Absolute chronology**	500 BCE - 250 BCE
**Type**	PRIA I
**Phase**	21
**Phase absolute dates**	500-250 BCE
**Decoration**	Undecorated

	Asset0079
**description**	
**Shape description**	Three-part vessel with a sharply kinked everted open rim, pronounced straight neck, a biconical body and a flat base
**Height**	16.4
**Rim diameter**	11.7
**Base diameter**	6.3
**Reference for the typology**	Jensen 2005; Martens 1992
**Reference for the asset**	

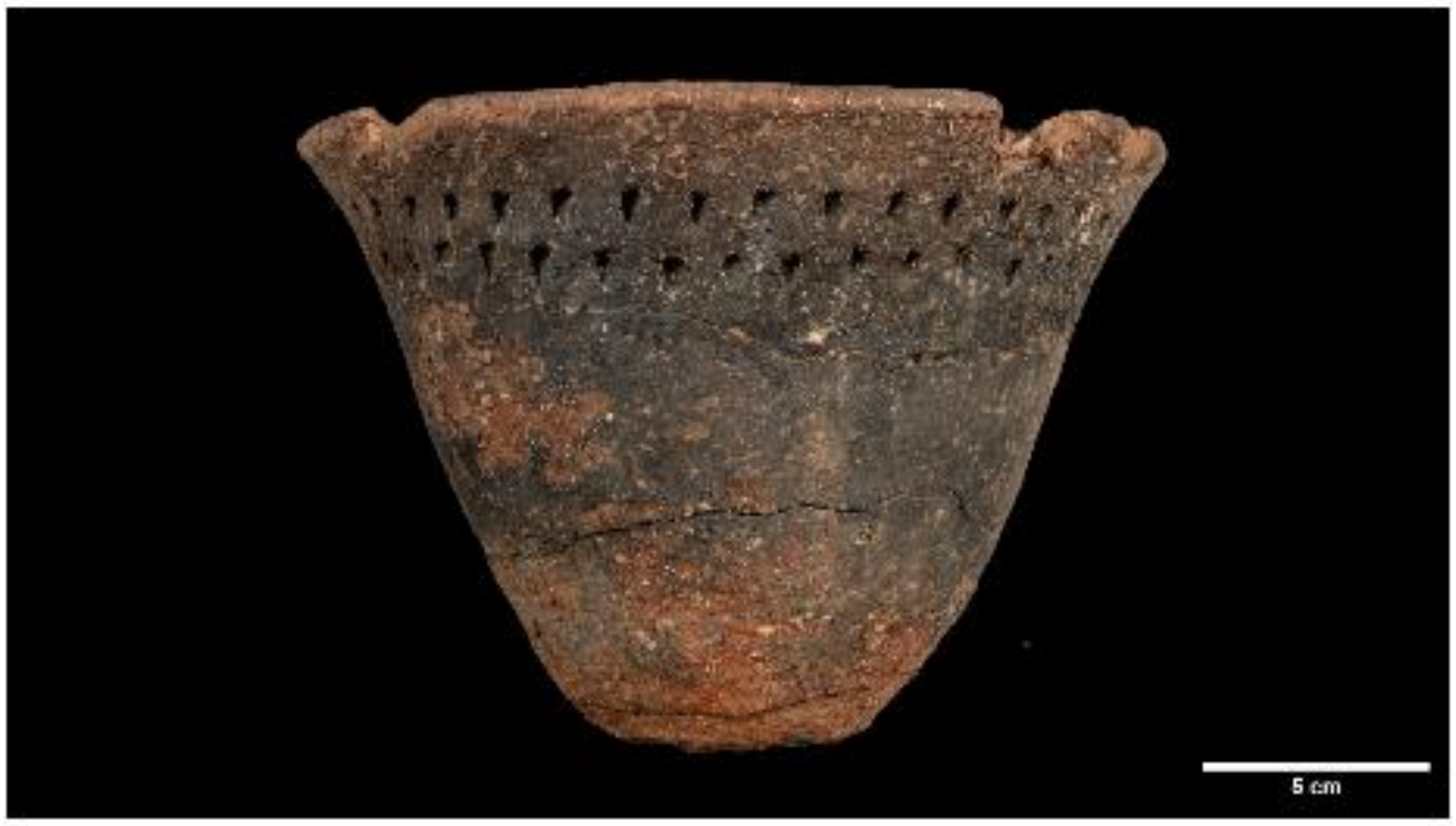

	Asset0080
**Museum Inventory N°**	FHM1616-O
**Site**	
**Context**	Megalithic monument
**Chronology**	MN V
**Absolute chronology**	2900 BCE - 2800 BCE
**Type**	
**Phase**	11
**Phase absolute dates**	3000-2750 BCE

	Asset0080
**Decoration description**	Two rows of triangular punchmarks below the vessels rim.
**Shape description**	V-shaped bowl with rounded base
**Height**	14.8
**Rim diameter**	20.6
**Base diameter**	6.3
**Reference for the typology**	Müller and Peterson 2014
**Reference for the asset**	

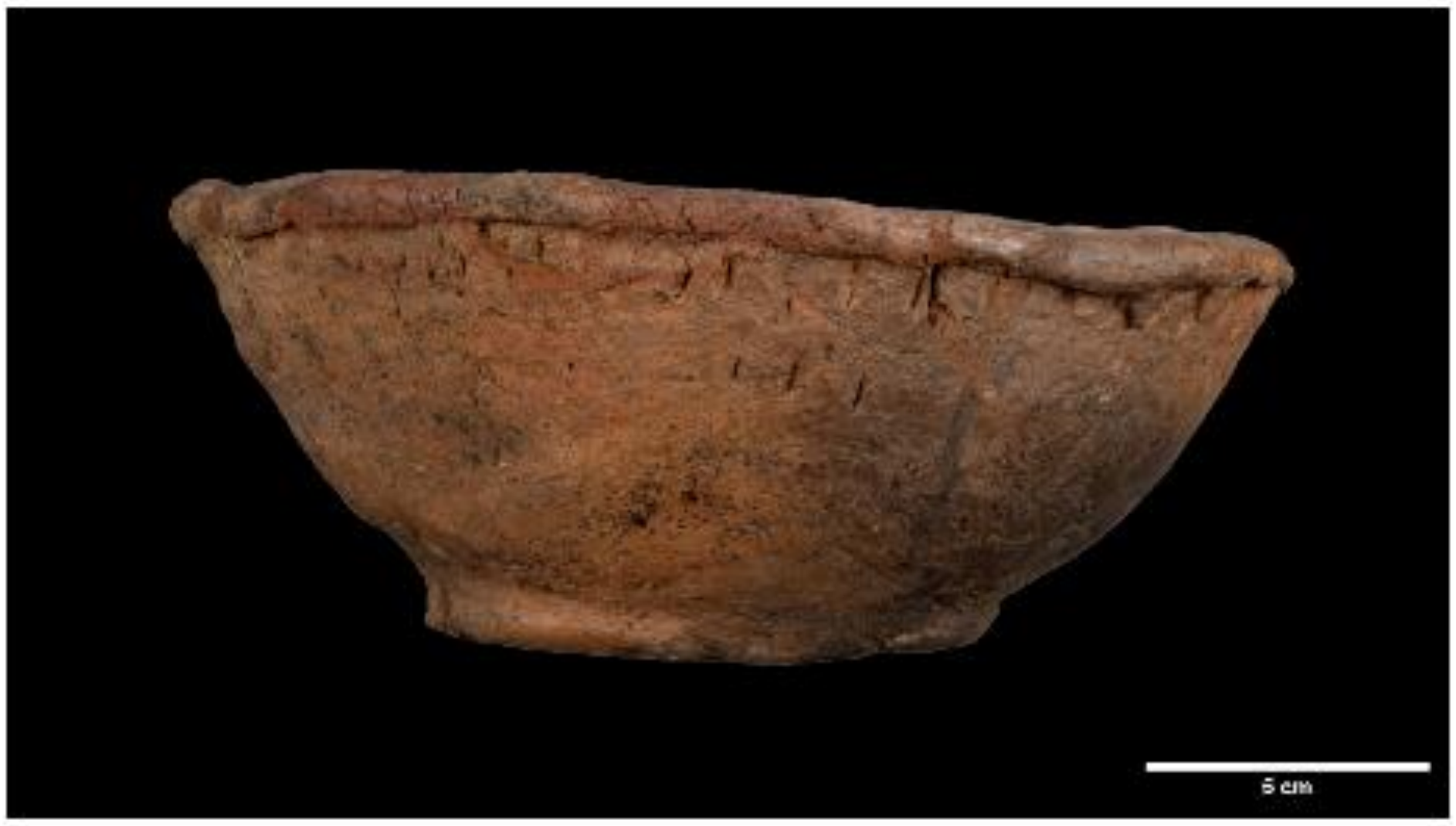

	Asset0081
**Museum Inventory N°**	FHM1656-fs
**Site**	Guldager, Denmark
**Context**	Grave
**Chronology**	YN 2
**Absolute chronology**	2600 BCE - 2400 BCE
**Type**	Middle Single Grave
**Phase**	12-13
**Phase absolute dates**	2750-2250 BCE
**Decoration**	Vertical stitches below the

	Asset0081
**description**	vessel’s rim
**Shape description**	Convex bowl with flat base
**Height**	8.3
**Rim diameter**	20.6
**Base diameter**	10.4
**Reference for the typology**	Müller and Peterson 2014
**Reference for the asset**	

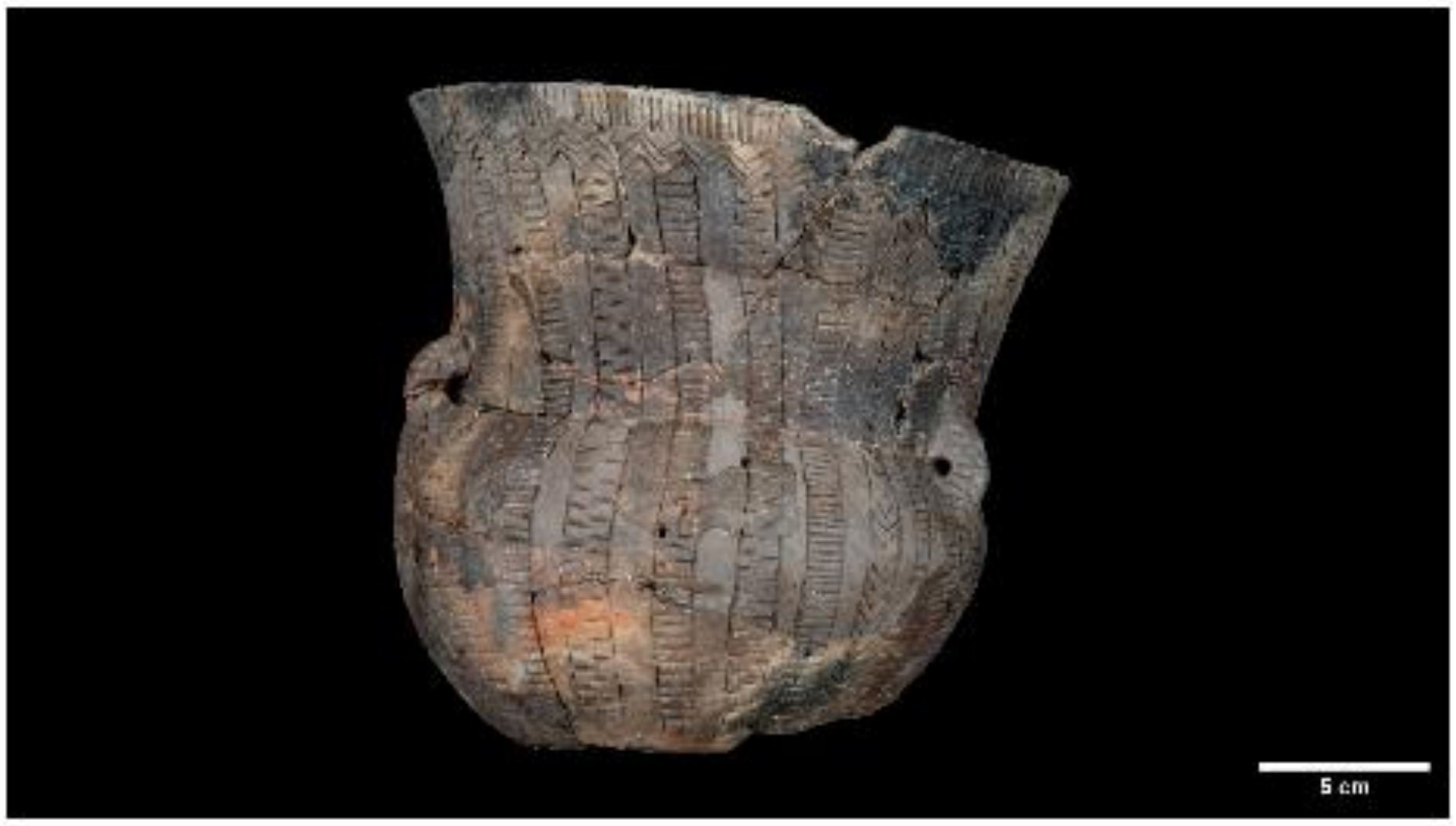

	Asset0082
**Museum Inventory N°**	FHM2314DK
**Site**	Bjerggård, Northern Jutland
**Context**	Ditch
**Chronology**	MN Ia
**Absolute chronology**	3300 BCE - 3200 BCE
**Type**	Troldebjerg
**Phase**	9-10
**Phase absolute dates**	3500-3000 BCE
**Decoration description**	Vertical incised line pattern below the vessel's rim followed by an incised chevron motif, and a series of vertical bands filled with stitches, horizontal (ladder-like) or angled incised stripes in the vessel's body. The decorated bands extend over two vertically oriented handles.
**Shape description**	Funnel-necked beaker
**Height**	19.6
**Rim diameter**	21.5
**Base diameter**	
**Reference for the typology**	Müller and Peterson 2014
**Reference for the asset**	Madsen 1988, Fig. 17.6b, p. 308

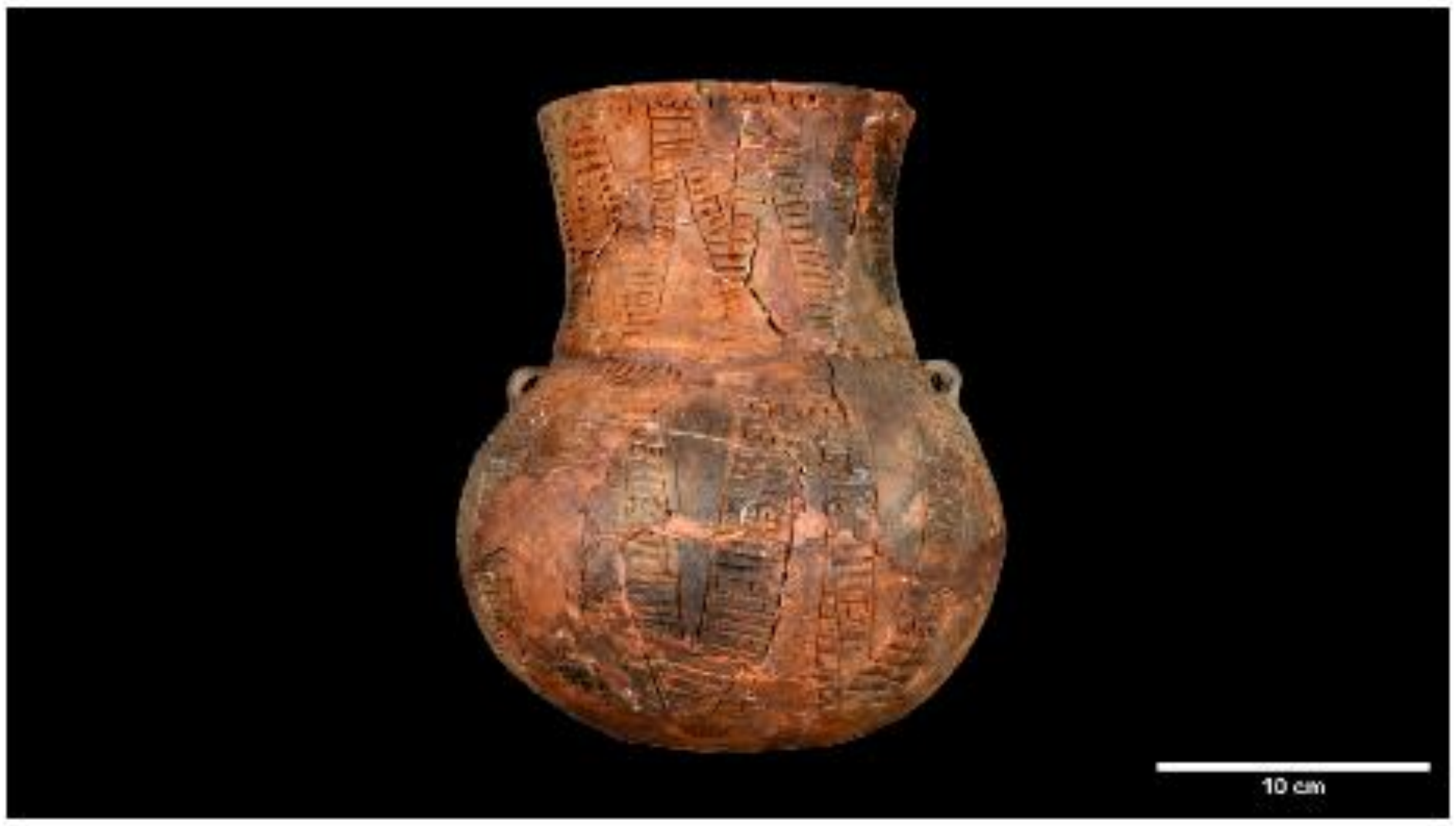

	Asset0083
**Museum Inventory N°**	FHM2314-DW
**Site**	Bjerggård, Northern Jutland
**Context**	Ditch
**Chronology**	EN II
**Absolute chronology**	3500 BCE - 3300 BCE
**Type**	Fuchsberg
**Phase**	9
**Phase absolute dates**	3500-3000 BCE
**Decoration description**	Vertical incised stripes extending below the vessel’s rim and shoulder followed by an incised zigzagged band filled with horizontal incisied lines and stitches. Two vertically oriented handles are placed joining the vessel’s neck and body.
**Shape description**	Flask with elongated neck and rounded base
**Height**	24.8
**Rim diameter**	14.3
**Base diameter**	8.8
**Reference for the typology**	Müller and Peterson 2014
**Reference for the asset**	Madsen 1988, Fig. 17.6a, p. 308

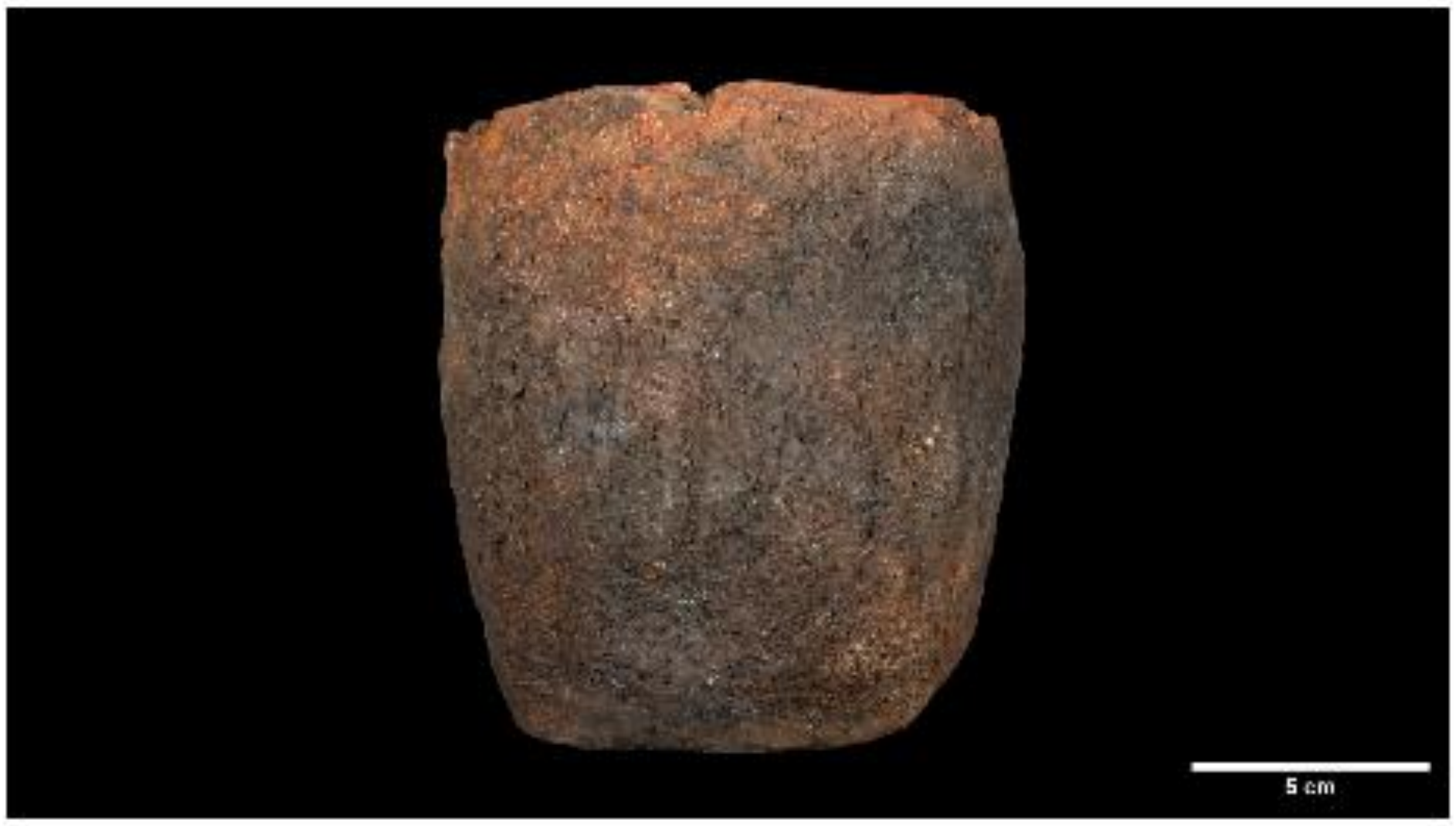

	Asset0084
**Museum Inventory N°**	FHM3341
**Site**	
**Context**	Burial Mound
**Chronology**	Period II/III
**Absolute chronology**	1500 BCE - 1300 BCE | 1300 BCE - 1050 BCE
**Type**	
**Phase**	17-18
**Phase absolute dates**	1500-1000 BCE

	Asset0084
**Decoration description**	Undecorated
**Shape description**	Cylindrical vessel with flat base
**Height**	14.1
**Rim diameter**	13.8
**Base diameter**	9.8
**Reference for the typology**	Jensen 2005; Lolk 2009
**Reference for the asset**	Schaefer-Di Maida 2020

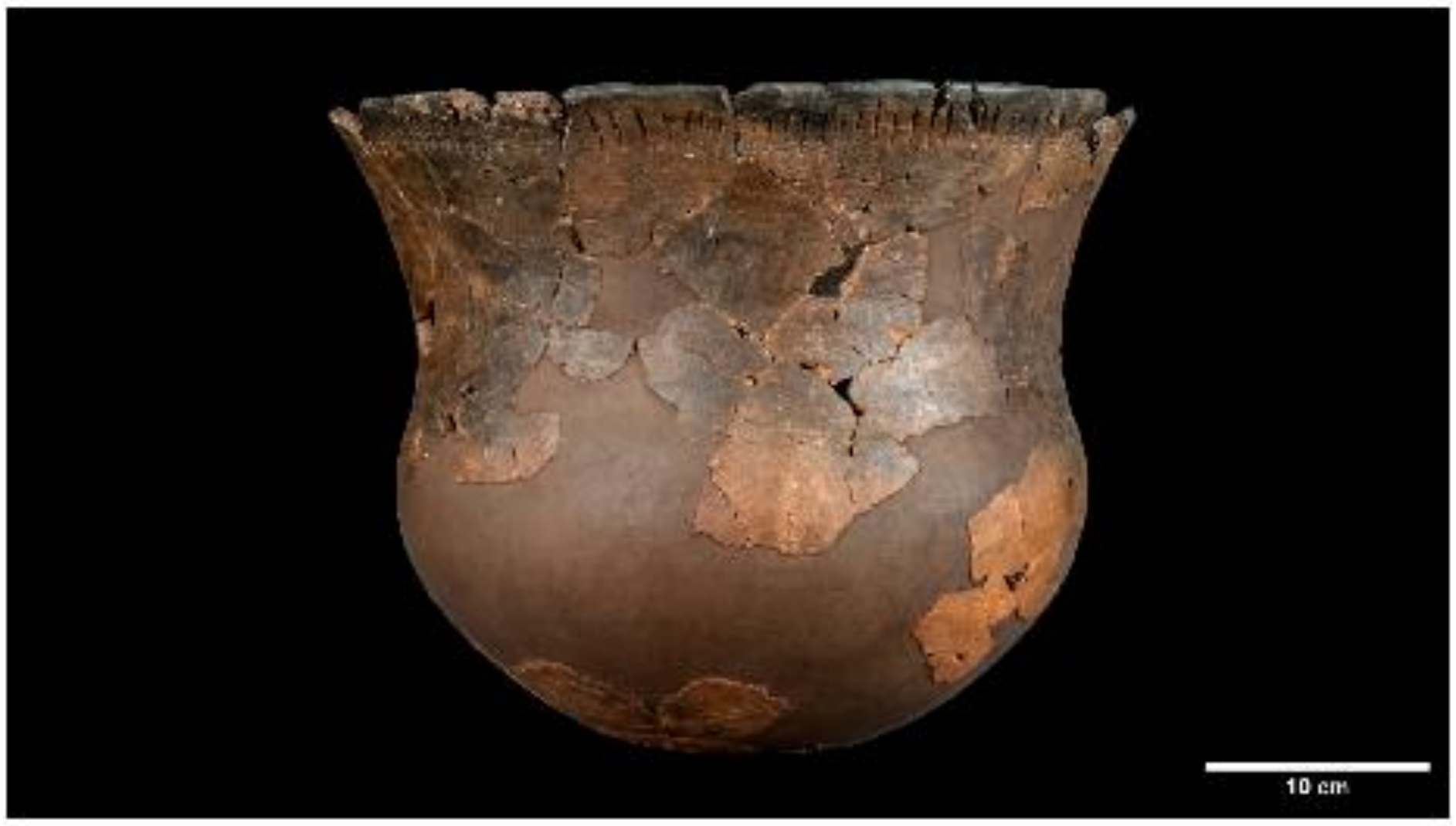

	Asset0085
**Museum Inventory N°**	FHM4183
**Site**	
**Context**	Pit/Settlement
**Chronology**	EN I
**Absolute chronology**	4000 BCE - 3500 BCE
**Type**	Volling
**Phase**	7-8
**Phase absolute dates**	4000-3500 BCE
**Decoration description**	Vertical stab-and-drag stitched stripe pattern below

	Asset0085
	the vessel’s rim.
**Shape description**	Globular vessel with funnel-shaped neck and rounded base
**Height**	29.8
**Rim diameter**	38.3
**Base diameter**	10
**Reference for the typology**	Müller and Peterson 2014; Sørensen 2015
**Reference for the asset**	Sørensen 2015

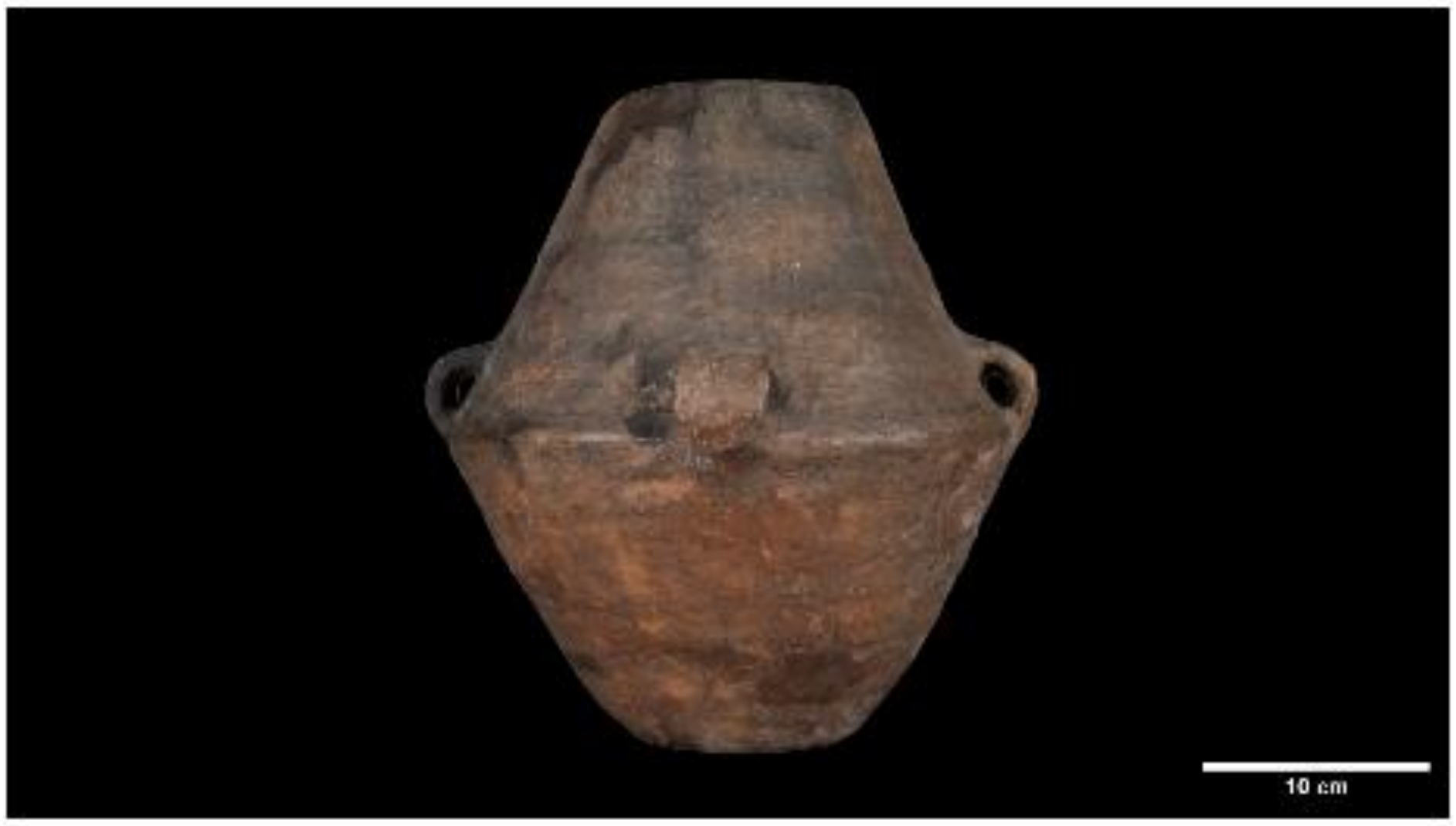

	Asset0086
**Museum Inventory N°**	33375
**Site**	Sarup, Denmark
**Context**	Settlement
**Chronology**	MN III-IV
**Absolute chronology**	3000 BCE - 2900 BCE
**Type**	Sarup IV – Bundsø
**Phase**	11
**Phase absolute dates**	3000-2750 BCE
**Decoration description**	Four vertically oriented large handles at the inflexion point between vessel's neck and body
**Shape description**	Two-part biconical jug
**Height**	29.8
**Rim diameter**	11.2
**Base diameter**	8.3
**Reference for the typology**	Müller and Peterson 2014
**Reference for the asset**	Andersen 1999

Experiment Report

Experiment 8

Composition

- **Type of objects:** Real
- **Presentation of the object:**
- **Object names:** Obj0230, Obj0232, Obj0234, Obj0236, Obj0238, Obj0240, Obj0242, Obj0244, Obj0246, Obj0248, Obj0250, Obj0252, Obj0254, Obj0256, Obj0258, Obj0260, Obj0262, Obj0264, Obj0266, Obj0268, Obj0270, Obj0272, Obj0274, Obj0276, Obj0278, Obj0280, Obj0282, Obj0284, Obj0292, Obj0293
- **Pictures:**
- **Control images names:**
- **Comments:**

Realisation

- **Location(s):** Aarhus
- **Date(s):** Feb 2024, Jan 2024
- **Number of participants:** 27

EQUATOR Network reporting (Dunn et al. (2024))

- **A1 Manufacturer and model**: The gaze data was collected using the Pupil Core mobile eye-tracking headset.
- **A2 Software and Firmware versions**: Pupil Capture software (version 3.5.1; Pupil Labs GmbH) running through the Psychopy® package. The recorded eye-tracking data were retrieved using the Pupil Player software (version 3.5.1; Pupil Labs GmbH).
- **A3 Eye tracking technology**: Video based eye-tracker.
- **A4 Sampling frequency**: maximum sampling rate of 200 Hz.
- **A5 Head movement restrictions**: There was no head restriction during the experiment.
- **A6 Eye(s) recorded**: Both eyes were recorded.
- **A7 Parameters recorded**: Gaze location, fixation location and duration, blinks and pupil diameter were recorded. Fixations were calculated with a threshold between 100-500 ms and a maximum dispersion of 1.5 degrees. The data were then processed by deleting fixations where pupil confidence detection was under 0.7.
- **A8 Environment lighting**: Experiments were always conducted in well-lit rooms with covered windows to minimise outdoor light.
- **A9 Calibration**: Screen calibration was performed at the beginning and end of each experiment. Post-hoc calibration was routinely performed to ensure calibration accuracy did not exceed three degrees of deviation. If despite post-hoc calibration, the accuracy was still above this threshold, the recording was not used.
- **A10 Measurement uncertainty**: The average accuracy accross participants is 1.85
- **A11 Data processing steps**: For projecting fixations into heat maps, world camera video frames were extracted and the coordinates and angle of the region of interest (ROI; i.e. the monitor screen) were calculated for each individual image frame using an automated ImageJ macro. The region of interest was defined according to values for the chroma of the green tape placed around the monitor frame. The extracted coordinates and angles for the region of interest were then used to recalculate and scale gaze positions and fixations so that these could be projected into the photograph shown during the experiment.
- **A12 Data loss**: the mean data loss accross participants is -13%, without counting non valid recordings (See details per participants below).
- **B2 Participant to display distance**: Participants were seated 0.65 m away from the display

Data quality

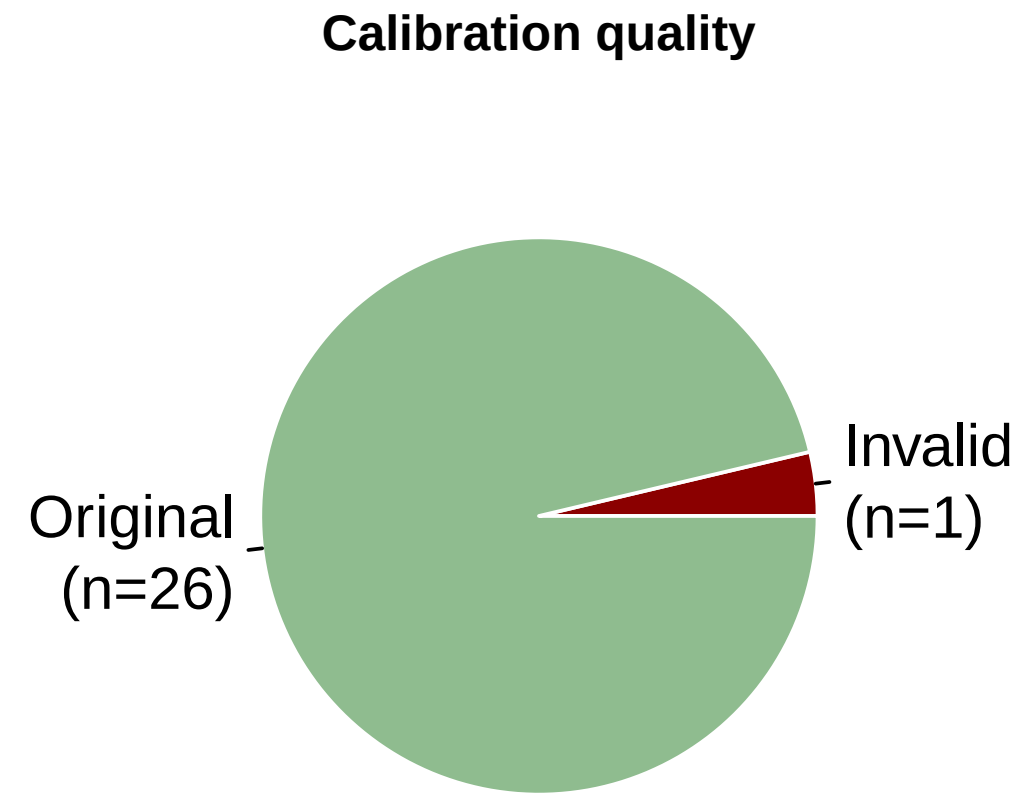

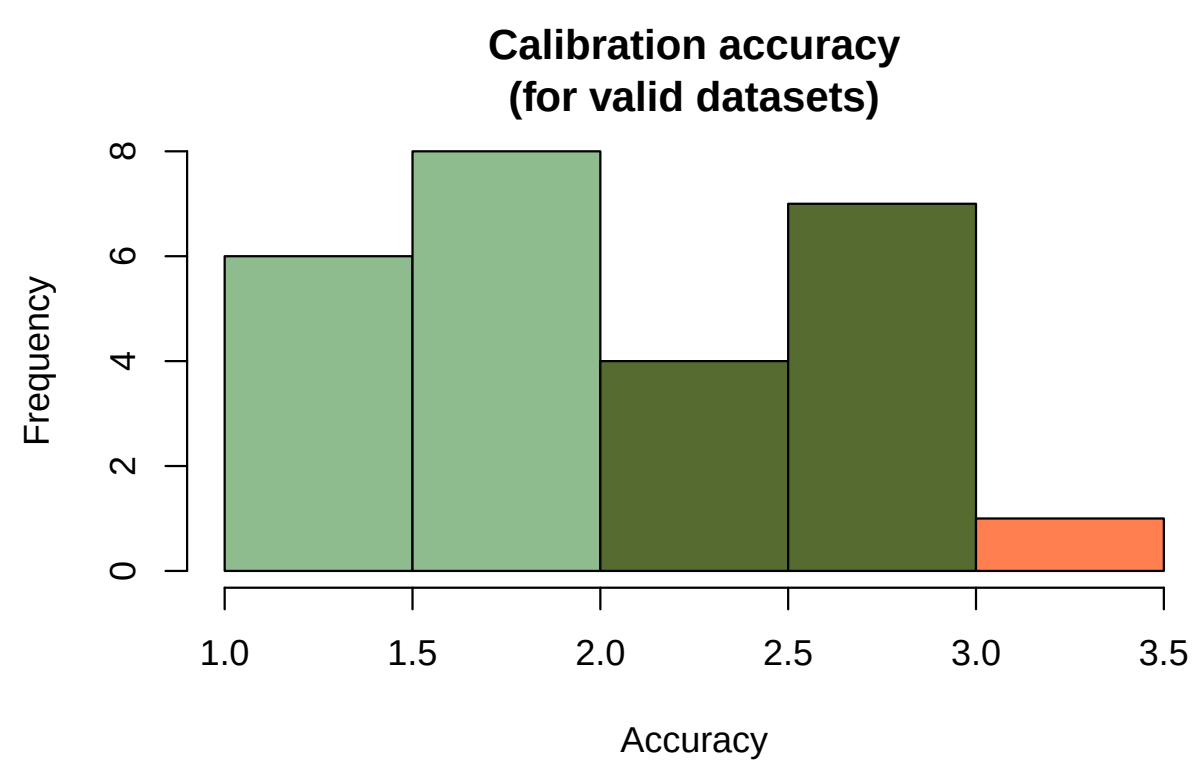

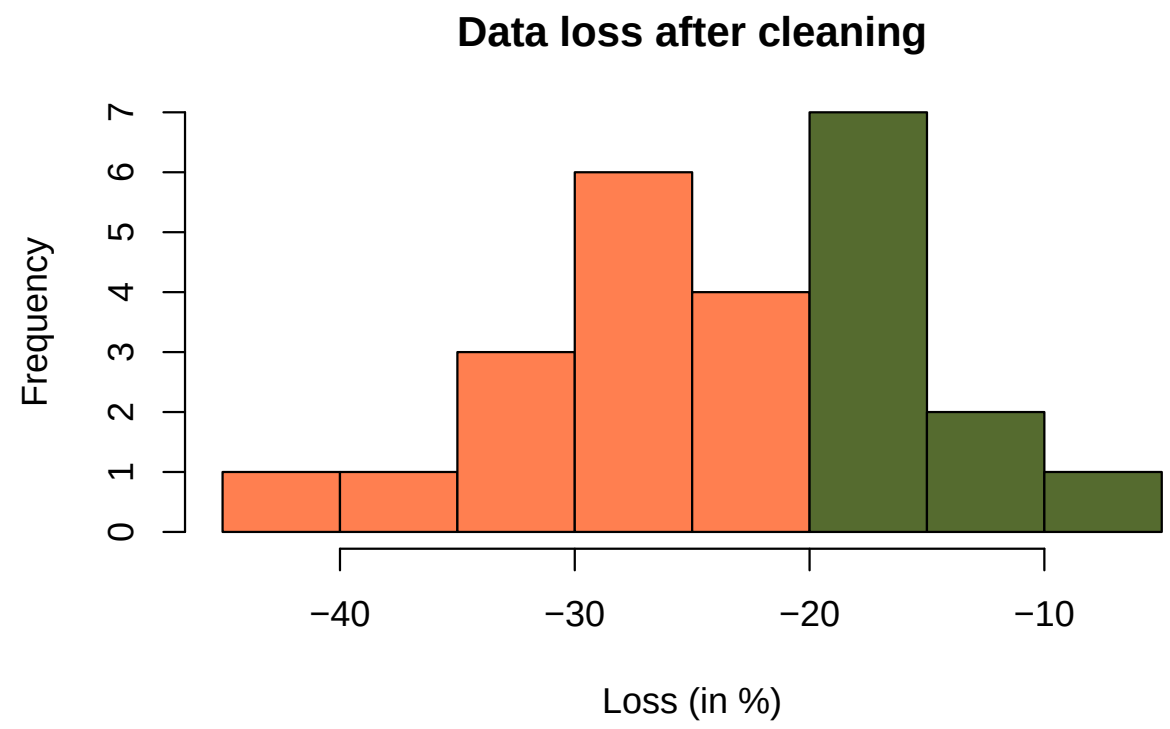

Analysed_Res_ID	Calibration.Accuracy	Data.loss	Blink.number
AnalysedRes0197	**1.841**	**-36.3**	**86**
AnalysedRes0198	**1.262**	**-28.5**	**106**
AnalysedRes0199	**2.118**	**-17.5**	**42**
AnalysedRes0200	**2.716**	**-30.9**	**168**
AnalysedRes0201	**1.580**	**-29.5**	**175**
AnalysedRes0202	**1.118**	**-31.6**	**233**

Analysed_Res_ID	Calibration.Accuracy	Data.loss	Blink.number
AnalysedRes0203	1.651	-19.2	44
AnalysedRes0204	1.976	-26.4	162
AnalysedRes0205	1.171	-28.2	66
AnalysedRes0206	2.541	-29.3	157
AnalysedRes0208	2.542	-13.3	36
AnalysedRes0209	2.521	-42.6	210
AnalysedRes0210	1.332	-22.4	138
AnalysedRes0211	2.014	-17.1	71
AnalysedRes0212	1.904	-15.0	51
AnalysedRes0213	2.188	-16.5	46
AnalysedRes0214	2.024	-22.1	136
AnalysedRes0215	1.251	-16.7	35
AnalysedRes0216	1.671	-19.0	106
AnalysedRes0217	1.521	-6.4	59
AnalysedRes0218	2.793	-27.1	0
AnalysedRes0219	2.816	-24.4	36
AnalysedRes0220	2.723	-22.1	130
AnalysedRes0221	1.208	-14.1	54
AnalysedRes0222	1.619	-30.0	136
AnalysedRes0299	3.100		

Socio-demographic info

Preselected groups

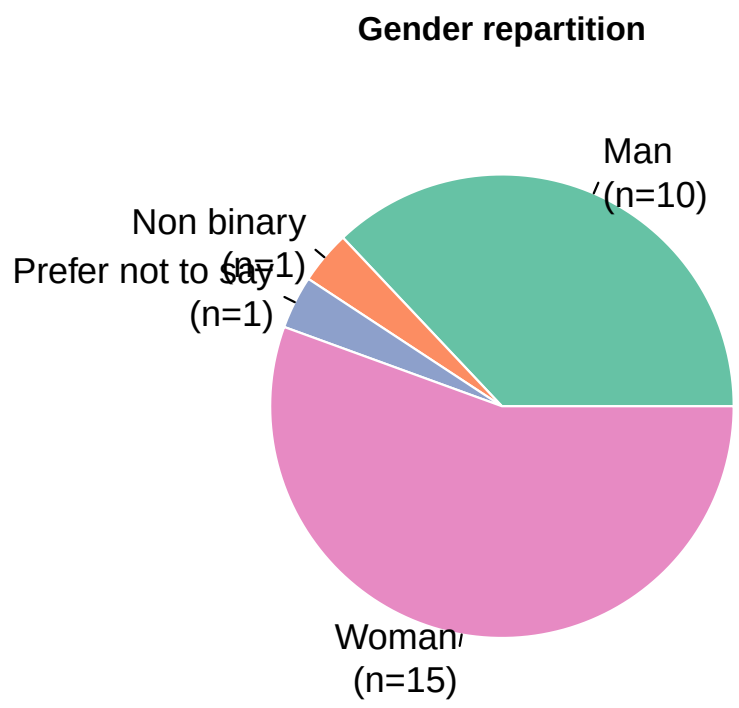

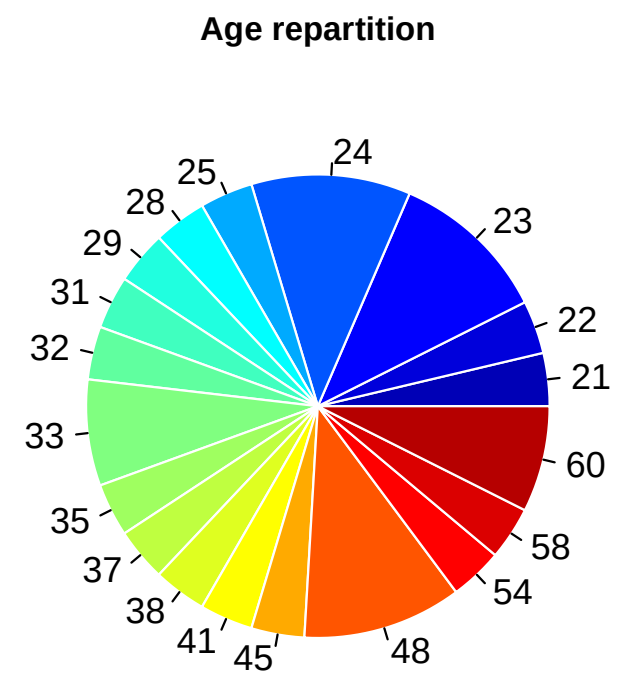

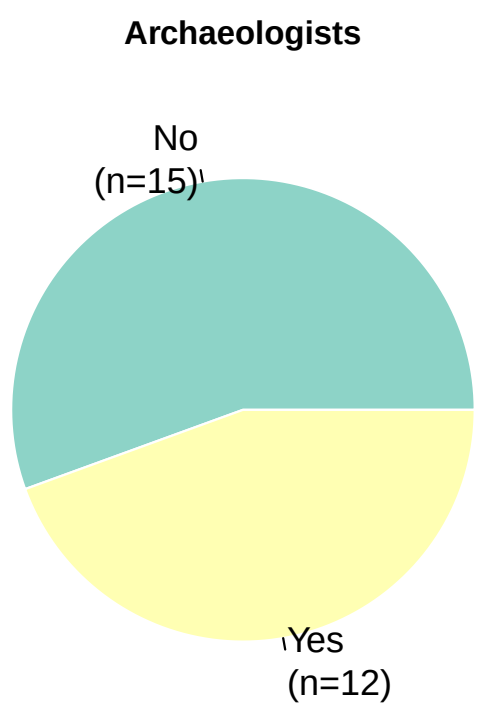

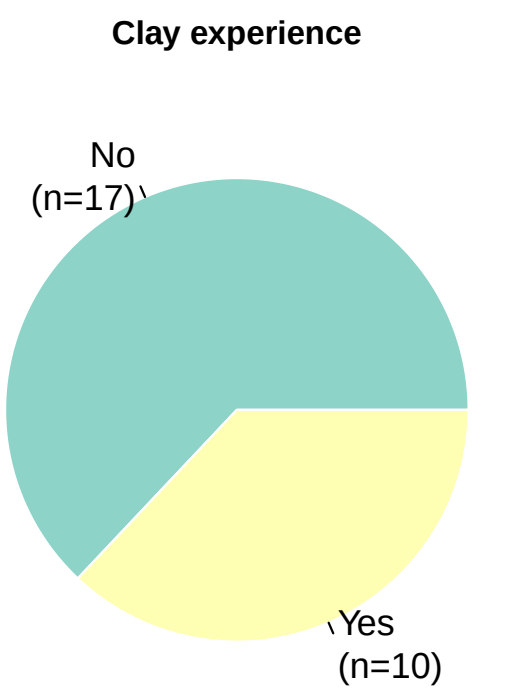

References

Dunn, Matt J., Robert G. Alexander, Onyekachukwu M. Amiebenomo, Gemma Arblaster, Denize Atan, Jonathan T. Erichsen, Ulrich Ettinger, et al. 2024. "Minimal Reporting Guideline for Research Involving Eye Tracking (2023 Edition)." *Behavior Research Methods* 56 (5): 4351–57. https://doi.org/10.3758/s13428-023-02187-1.

Appendix A.

Participant Information Form

Thank you for your interest in taking part of our [Anonymised]. Before continuing with our experiments, please take a moment to read the following form, which contains basic knowledge about our project and your rights as a participant in our experiments. The regulations listed follow the EU General Data Protection Regulation. This form is necessary due to legal regulations on ethical aspects of experiments.

1. Project information

[Anonymised] project investigating the various ways our settlements, buildings, roads, and artefacts (from pottery to smartphones) actively change our patterns of thought and attention, thereby shaping the modern mind. It attempts to put theoretical, practical, and computational explore the idea that the modern mind is in many ways our own invention – the product of a series of changes to our self-created environments. We will perform a series of perceptual experiments in various parts of the world. One of such experiments will be conducted now at the Moesgaard Museum (Højbjerg, Denmark), for which we very much appreciate your participation (details provided below).

1.1 What are our goals?

We consider that the information from our project may not only inform us about how the visual perception of past human population changed, but also allow us to understand how the objects we use on a daily basis might be influencing the way we behave today.

1.2 What do our experiments consist of?

On this occasion, we will be conducting two experiments that we expect will take around 30-45 minutes of your time. For both of these experiments you will be asked to wear our eye-tracking glasses and simply observe a series of objects (images and archaeological artefacts).

The eye-tracking glasses are simply composed of three cameras. Two are oriented towards each of your pupils and a third camera positioned near your forehead facing your field of vision. Together these cameras record where you fixate your gaze.

During the experiments we will record everything you observe and store this visual information (please see section 2 for more information on how we collect and process this data). Additionally, the experiments will be sound recorded, which is exclusively done to monitor environmental disturbances that may have an impact in our recording of your eye movement.

Please be advised that neither the equipment used nor the procedures conducted for these experiments pose any harm or risk to your mental or physical health.

For these experiments, we require obtaining your contact details, medical and socio-demographic information, as well as the participant data we obtain about your visual behaviour (see section 2). One of the reasons for this: it is possible that viewing behaviour is influenced by age and gender, for example - in order to research this, the age and gender data of the participants in the experiment are required. For this we require your informed consent.

Please be advised that:

- **Your consent is entirely voluntary**. You can revoke your consent to the further collection and use of your contact data, medical and sociodemographic information and participant data at any time, in whole or in part, without giving reasons and without reprisals. (Article 7(3)(a) of the EU General Data Protection Regulation).
- **You are not obliged to answer all the questions we ask you**.

2. Collection, processing and scientific use of your contact details, medical and socio-demographic information and participant data

For the experiments described above we will collect three types of data. Your **contact details** will simply include information that allows us to communicate with you at a later stage). You may choose any form of contact you prefer, including: your address and postcode, phone number or your email. Your **medical and socio-demographic data** is a combination of medical information related to your vision, such as your visual acuity and/or correction, and more general information including your age, gender, education level, etc. This data allows us to determine the factors that can explain your visual reaction to the objects shown. **Participant data** refers to the information gathered during your participation in the experiments, and exclusively refers to your visual behaviour, e.g. video recording from our eye-tracking device, measurements extracted from this video, among others.

We have taken a number of measures to protect your privacy, i.e. to protect your contact details as well as your medical and socio-demographic data:

- **Data segregation and restriction**: Your contact information will remain in paper form and will be stored separately from your medical, socio-demographic and participant data. This information is only processed by our data protection officer (contact information in Section 3*). **Please note that data identifying you will never be shared with researchers outside of our project or with any other third party.***
- **Pseudonymisation**: instead of using your real name, you will be provided an encoded participant internal identifier or ID, which will be the only link between the data we collect and all data that directly identify your person (name, telephone, address, etc.). In this way, this internal identifier and your associated contact details can no longer be directly traced back to you.

3. Further information and rights

The legal basis for processing your contact details, medical and socio-demographic information, and participant data is your consent (Article 9 (2) (a) and Article 6 (1) (a) of the EU General Data Protection Regulation). The institutions responsible for handling your data are the [Anonymised]. Contact details for these institutions are the following:

[Anonymised]	[Anonymised]
[Anonymised]	[Anonymised]

If you wish to withdraw or change your consent, please contact us at: [Anonymised]

The **data protection officer** for our project can be contacted at [Anonymised]. The supervisory authority for this institution is the [Anonymised].

Participant declaration of consent

Please complete this form after you have read the Information Sheet and/or listened to an explanation about the research.

Project: [Anonymised]
Experiment: Aarhus 29.01.2024-02.02.2024

Thank you for your interest in taking part in this research. Before you agree to take part, the person organising the research must explain the project to you.
If you have any questions arising from the Information Sheet or explanation already given to you, please ask the researcher before you to decide whether to join in. You will be given a copy of this Consent Form to keep and refer to at any time.

Participant's Statement

I agree that:

- I have read the notes written above and the Information Sheet, and understand what the study involves.
- I understand that my consent is entirely voluntary and if I decide at any time that I no longer wish to take part in this project, I can notify the researchers involved and withdraw immediately without giving reasons and without any reprisal.
- I have been informed about the use of my personal data and the associated risks, and give my consent within the framework described above.
- I understand that such information will be treated as strictly confidential and handled in accordance with the provisions of the EU General Data Protection Regulation.
- I have had sufficient time to properly consider the matter and all my questions have been answered to my satisfaction.
- I have been informed that I will receive a copy of the participant information sheet and a copy of the signed consent form.

Place, Date	Participant Full Name	Participant signature
I, in person, provided the participant with information and guidance.	Researcher/Employee Full Name	Researcher/Employee signature

Participant Legal Information

ID ____________

(To be completed by the organisers)

Please write readably or in capital letters

Name: ____

First name: ____

Please give us at least one way to contact you in the future

Email: ____

Phone: *(please put the country indicator)* +_____________________________________

Figure A1. Participant contact details form.

Participant Medical and Socio-demographic Information Form

ID ☐

(To be completed by the organisers)

Age: _____ **Gender:** ☐ Woman ☐ Man ☐ Non-binary ☐ Prefer not say

Country of residence: ______________________________

City: ____ **Postcode:** _____________________

If you are not living in your country of origin:

Country of origin: _________________________________

How long have you been in the country of residence: __________

Native language: ______________________ **Spoken language:** ___________________________

What was your environment growing up: ☐ Urban ☐ Rural

What is the environment you are living in now *(multiple responses possible)*:

☐ Urban ☐ Rural ☐ City center ☐ Suburb ☐ Flat ☐ House

Do you know how to **Read:** ☐ Yes ☐ No **Write:** ☐ Yes ☐ No

Education *(highest level reach)*: ____

Are you an archaeologist? ☐ Yes ☐ No

Do you have any experience with clay? ☐ Yes ☐ No

Do you have any prior knowledge about this project or experiment? ☐ Yes ☐ No

Dominant hand: ☐ Right ☐ Left

Dominant eye: ☐ Right ☐ Left *(If you don't know this information, the organisers will help you determine your dominant eye)*

Visual acuity, if known *(i.e. 10/10)*: __________

Do you have any visual diagnosis *(i.e. astigmatism, color blindness, myopia)?*:__________________

Visual aid: ☐ Glasses ☐ Contact Lenses ☐ None

Do you have any history of psychiatric diagnosis? ☐ Yes ☐ No

Are you under the influence of any substance or medication? ☐ Yes ☐ No

Are you wearing any makeup? ☐ Yes ☐ No

If you have any question while completed this form, don't hesitate to ask the organisers!

Figure A2. Medical and socio-demographic forms used for the experiments

Appendix B. Distribution of vessel sizes according to chronological phases

Measurements of vessel sizes according to their surface area were conducted using image analysis of scaled photographs used in experiment 8. Figure B1 highlights the distribution of vessel sizes according to chronological phases. As highlighted in the main text of this article, distribution of vessel sizes was uniform except for the first chronological phases (4-8), which contains only large-sized vessels. However, as also mentioned in the text, this is a representation of the vessel size distribution at the time, as mostly large-sized vessels occur in the Ertebølle and Volling styles.

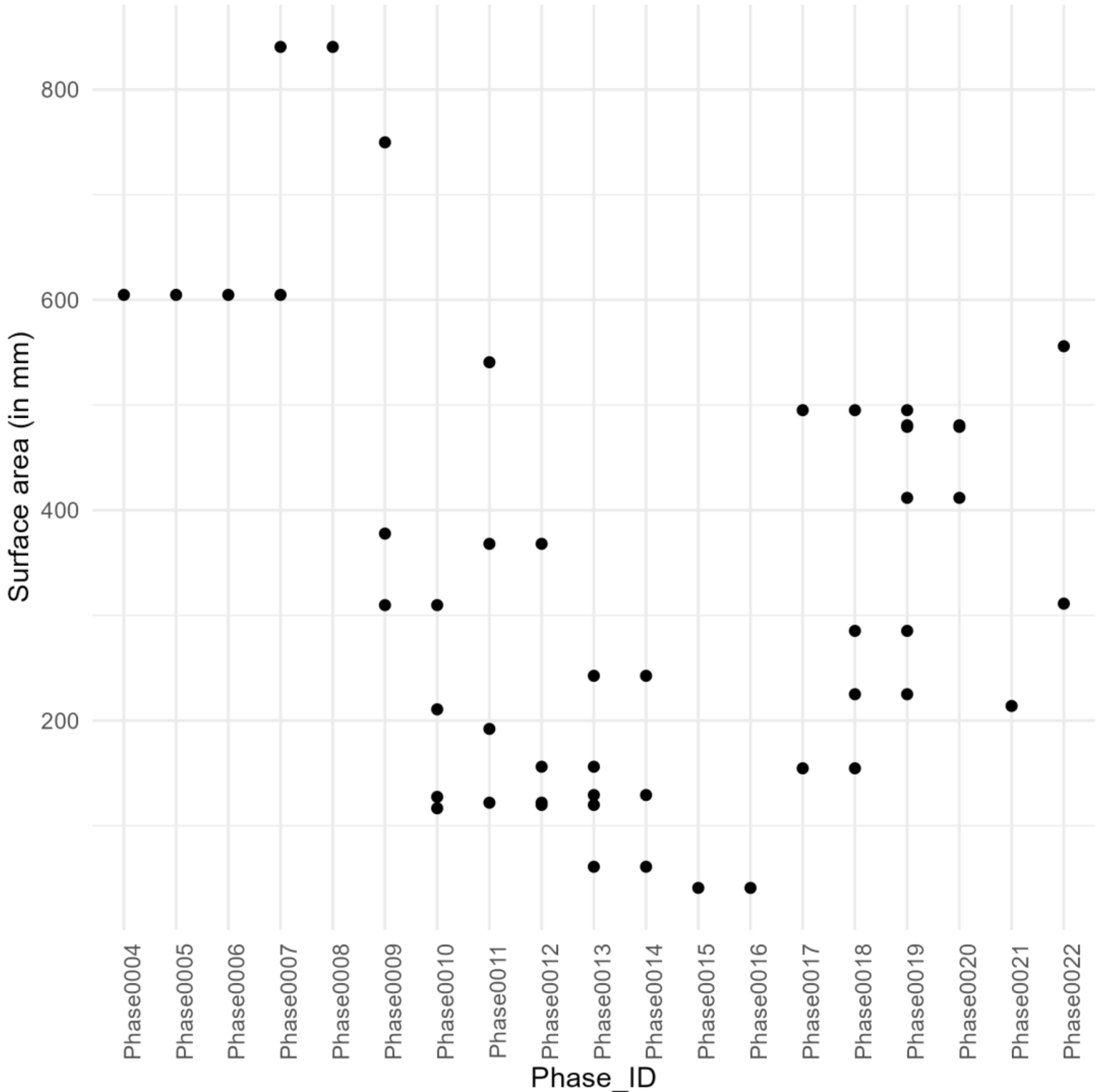

Figure B1. Distribution of vessel sizes per chronological phase.

Appendix C. Fixation durations distribution according to chronological periods

Compared z-scored fixation durations from central Germany (red) and Northern Jutland (green). The data are ordered by archaeological periods and the table below the graph explain how the two regions were merged in a single chronology.

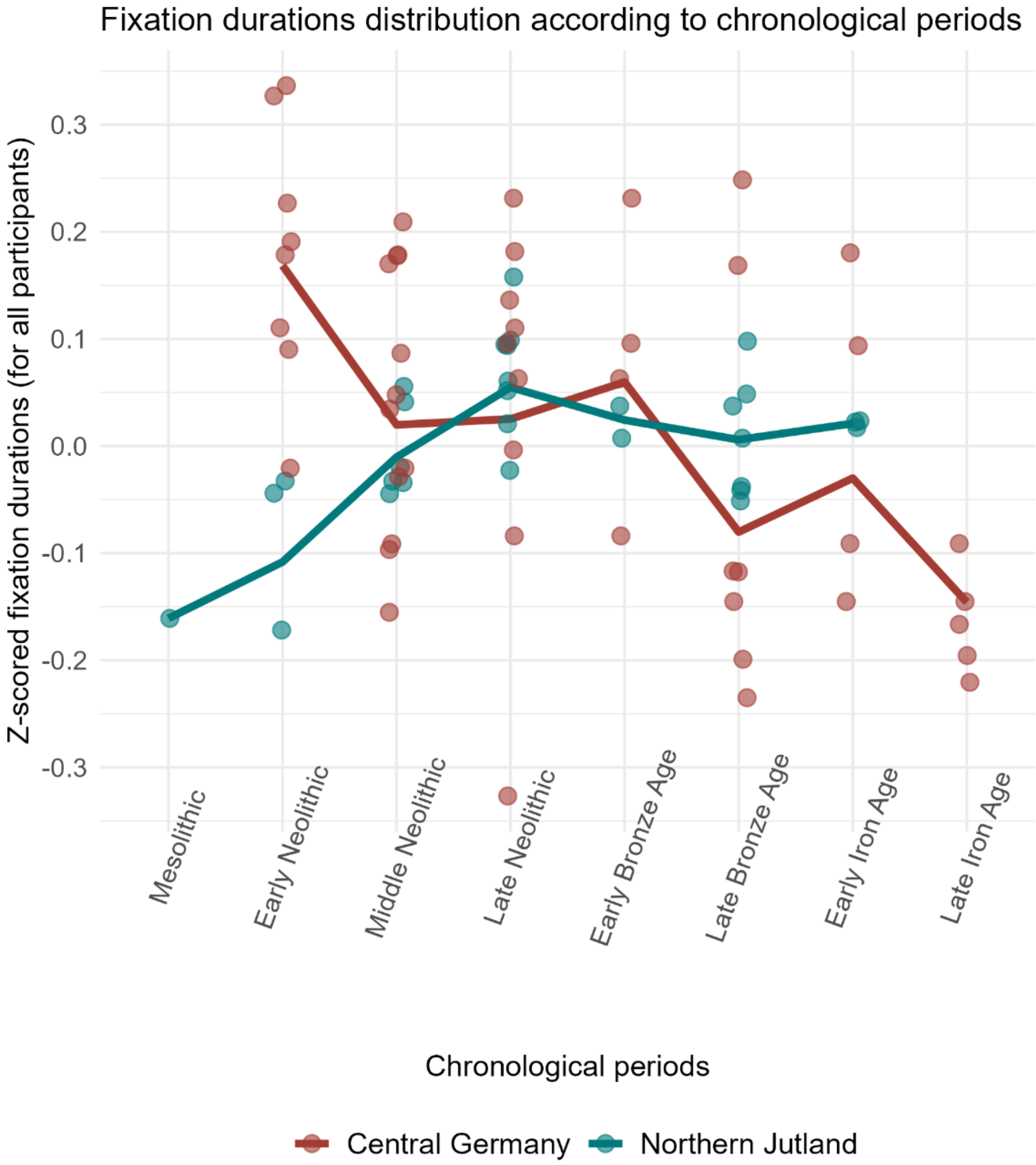

Chronological equivalences		
	Northern.Jutland	Central.Germany
Mesolithic	Final Mesolithic	
Early Neolithic	Early Neoltihic	Early and Middle Neolithic
Middle Neolithic	Middle Neolithic	Younger and Late Neolithic
Late Neolithic	Younger Neolithic	Final Neolithic
Early Bronze Age	Older Bronze Age	Early and Middle Bronze Age
Late Bronze Age	Younger Bronze Age	Late Bronze Age
Early Iron Age	Pre Roman Iron Age	Early Iron Age
Late Iron Age		Late Iron Age

Obj0230

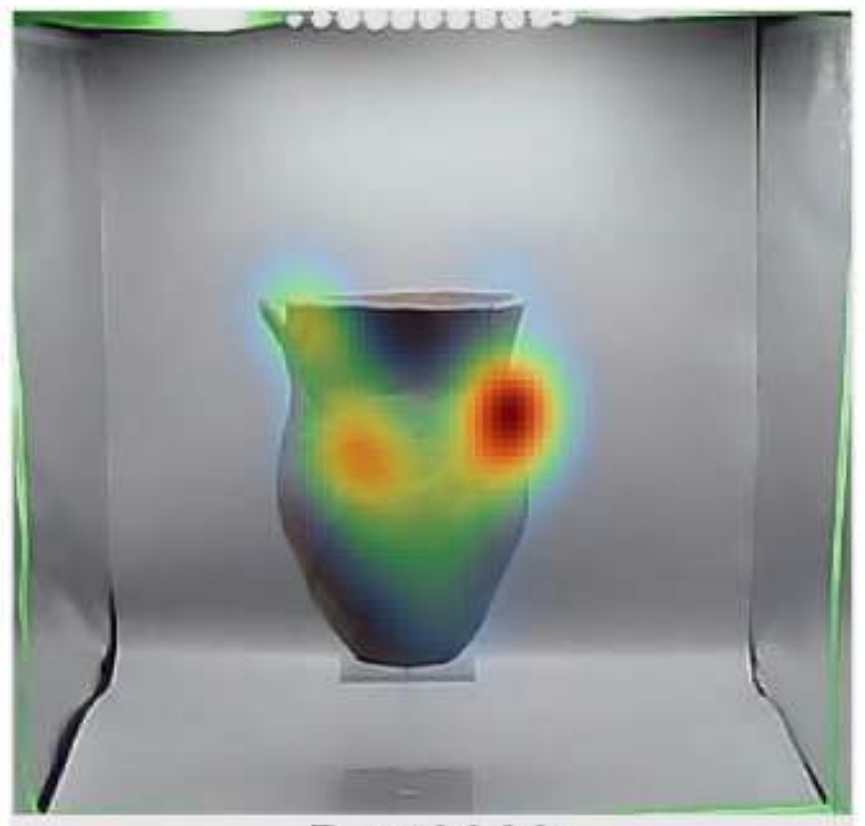
Part0089

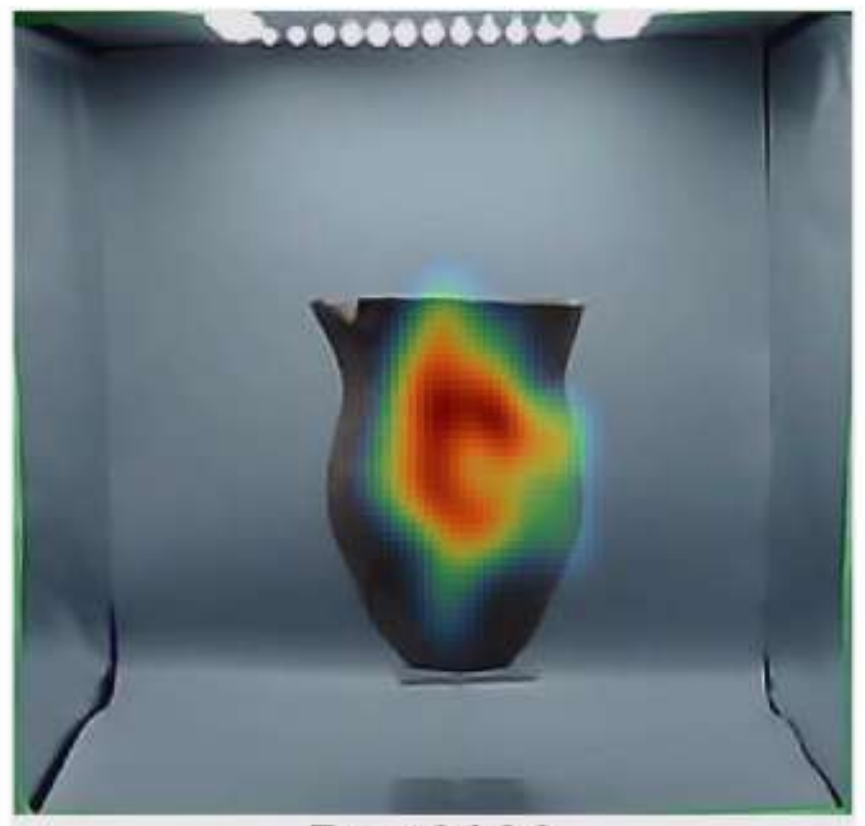
Part0090

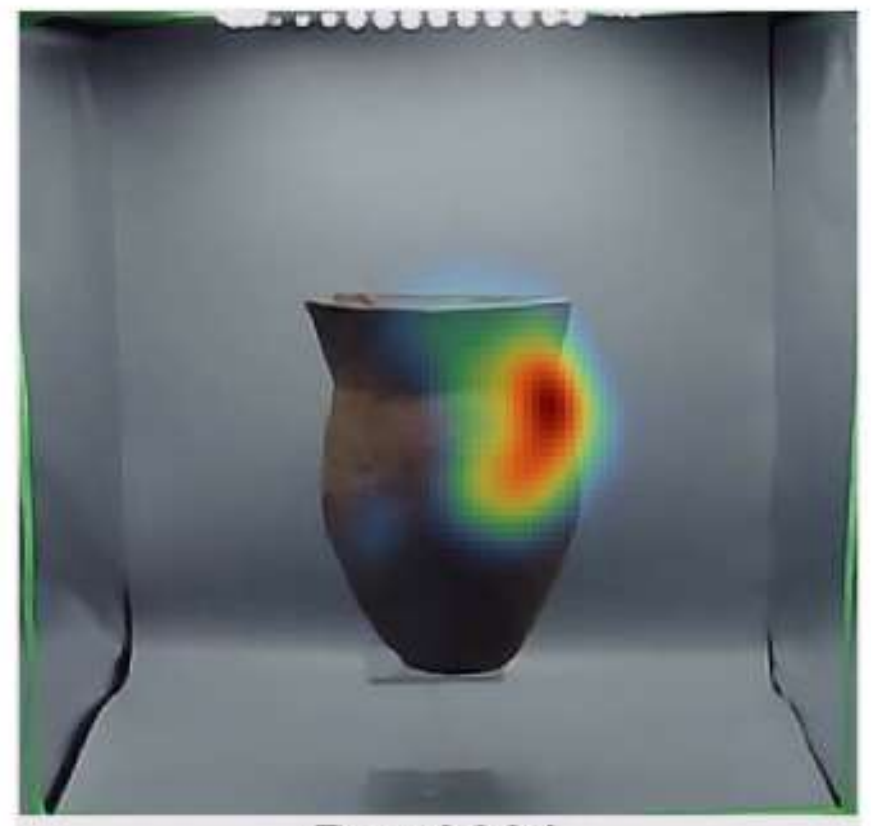
Part0091

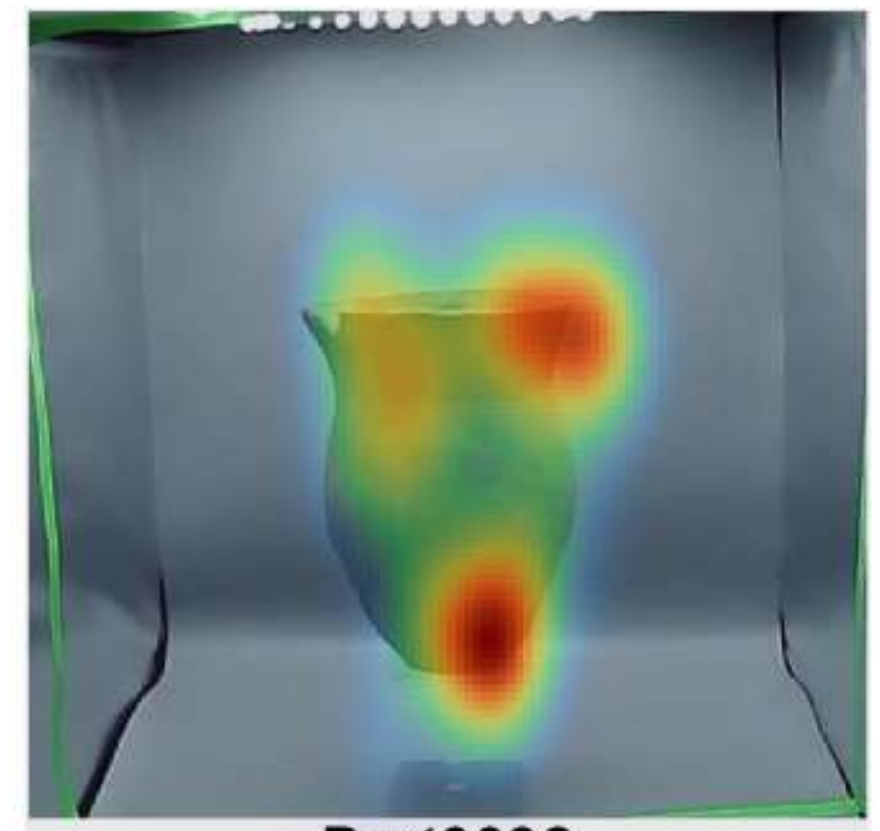
Part0092

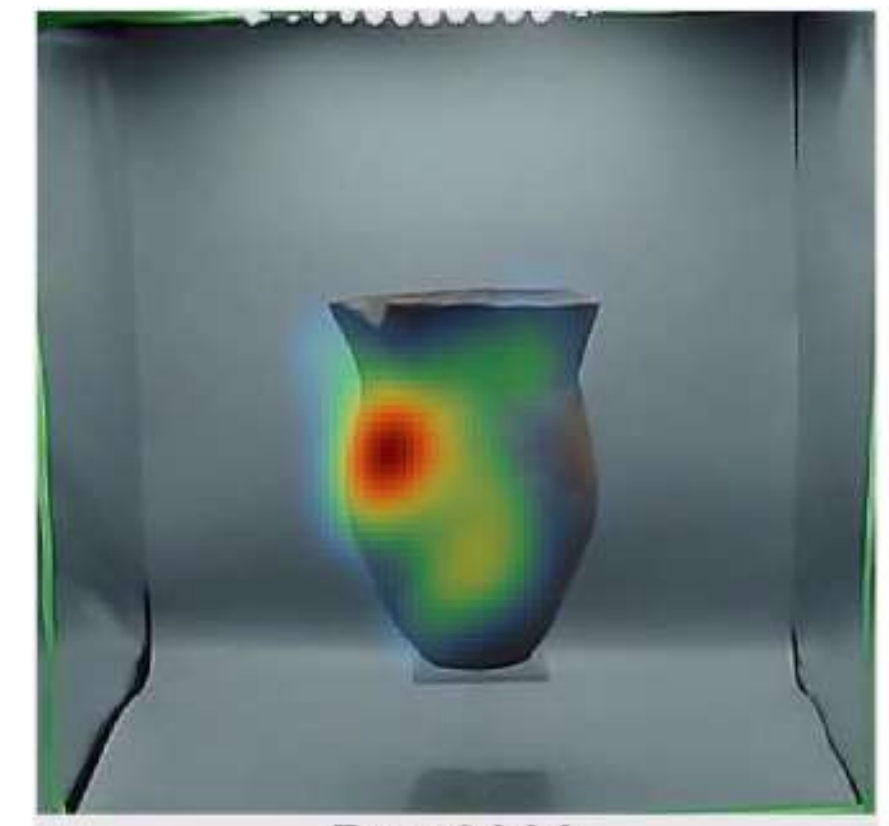
Part0093

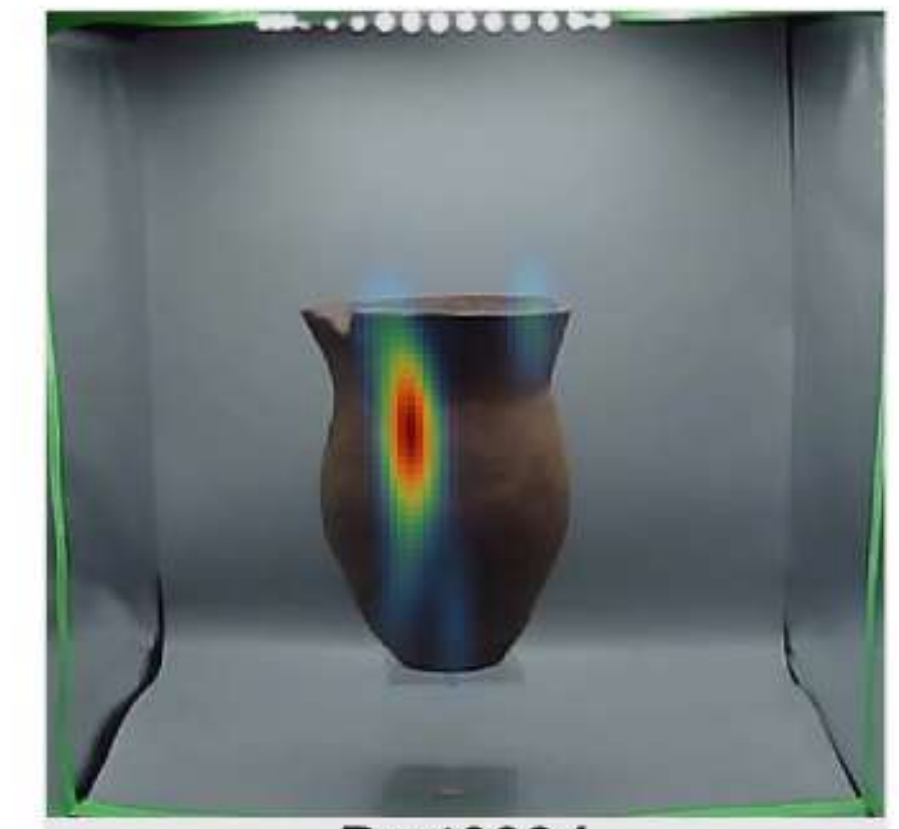
Part0094

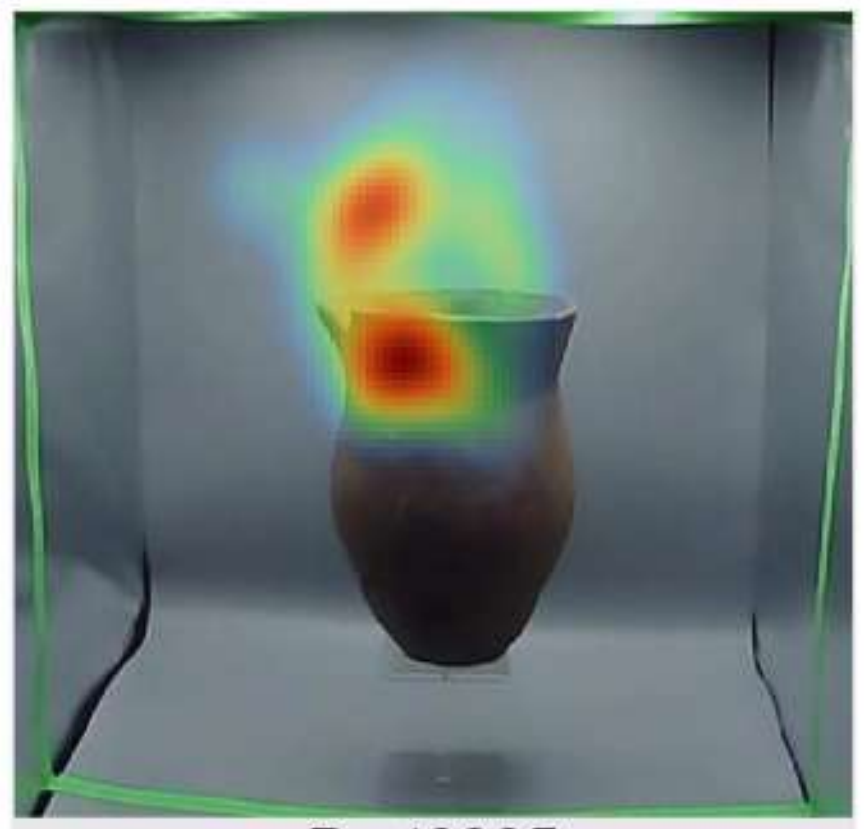
Part0095

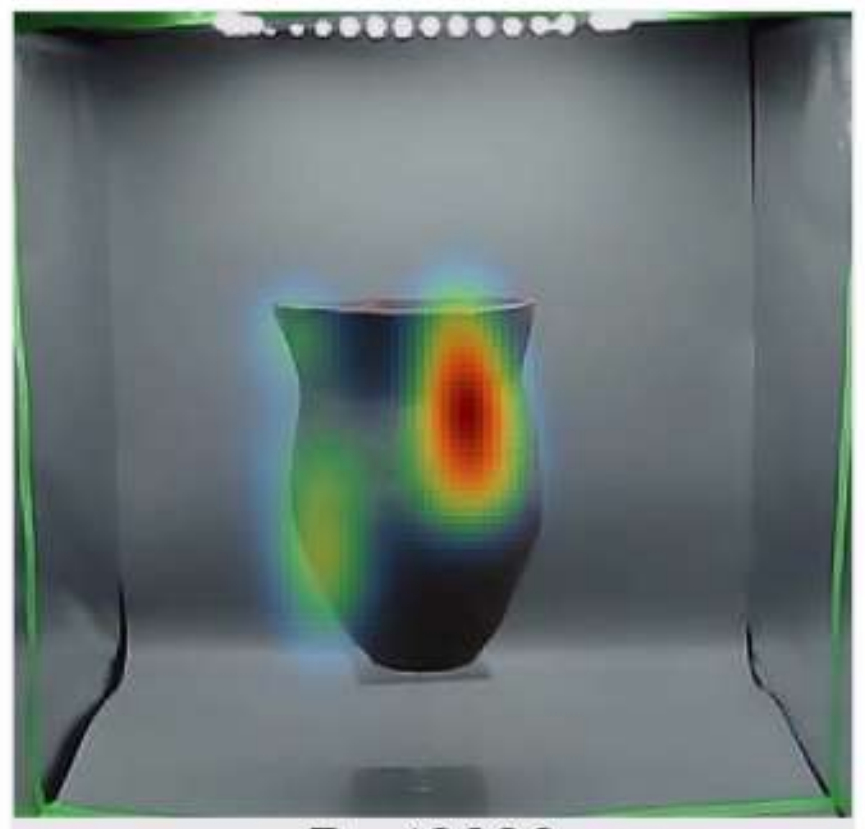
Part0096

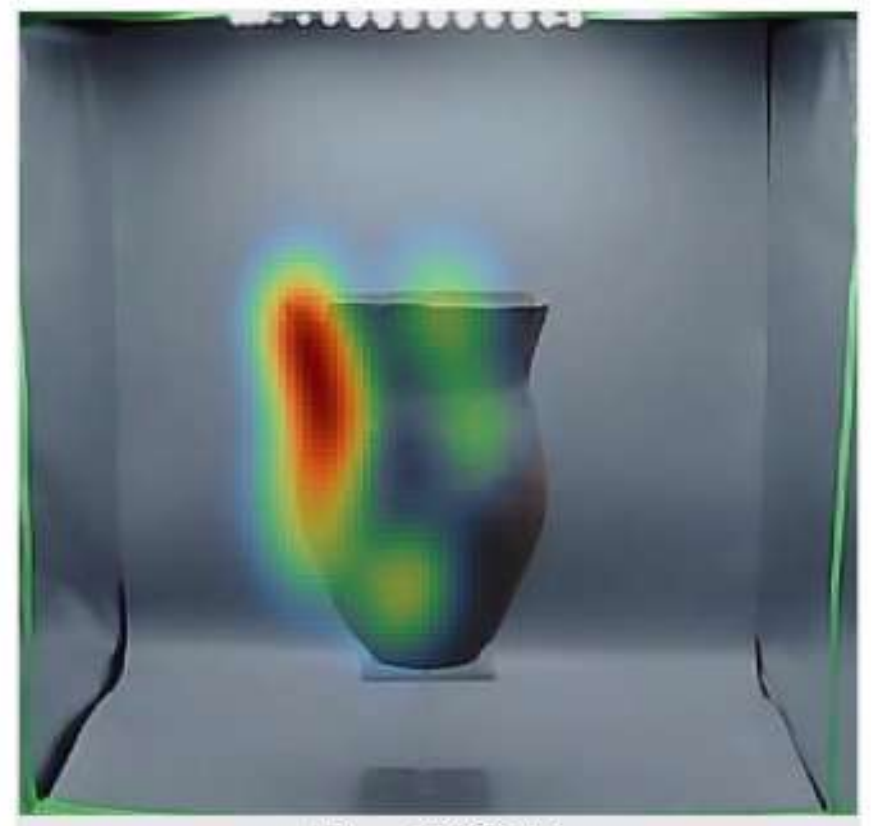
Part0097

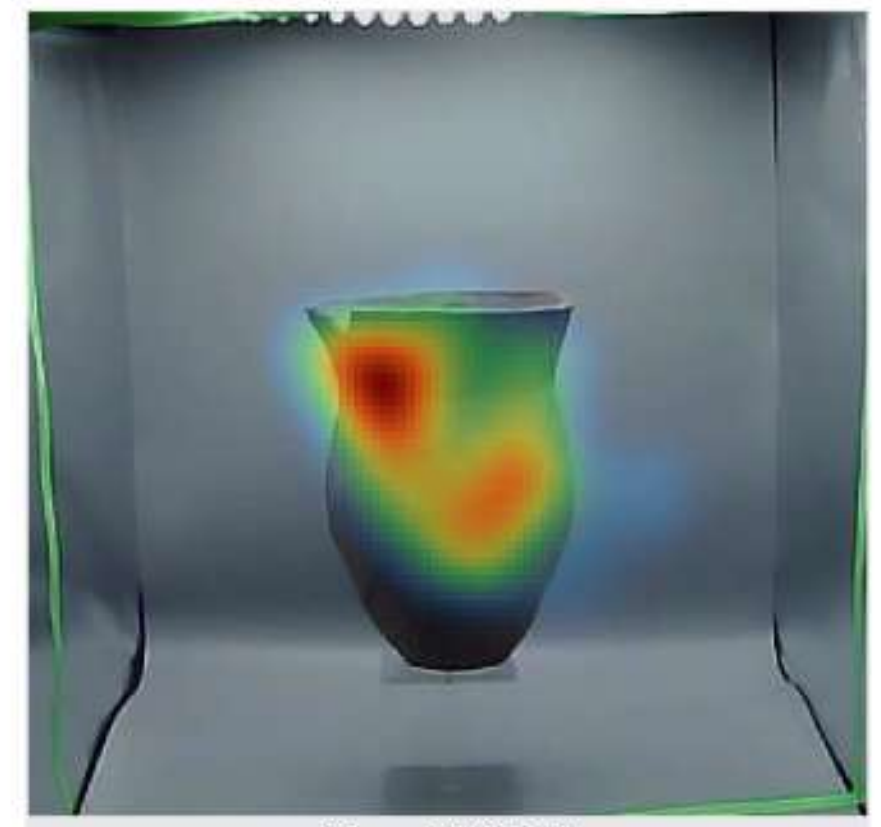
Part0098

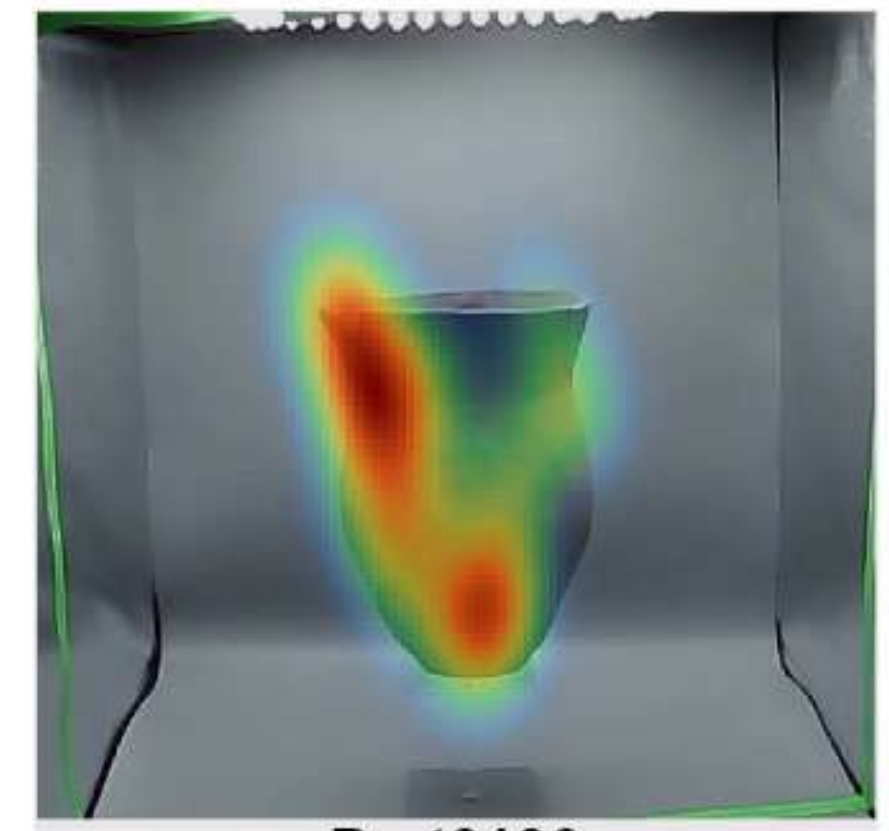
Part0100

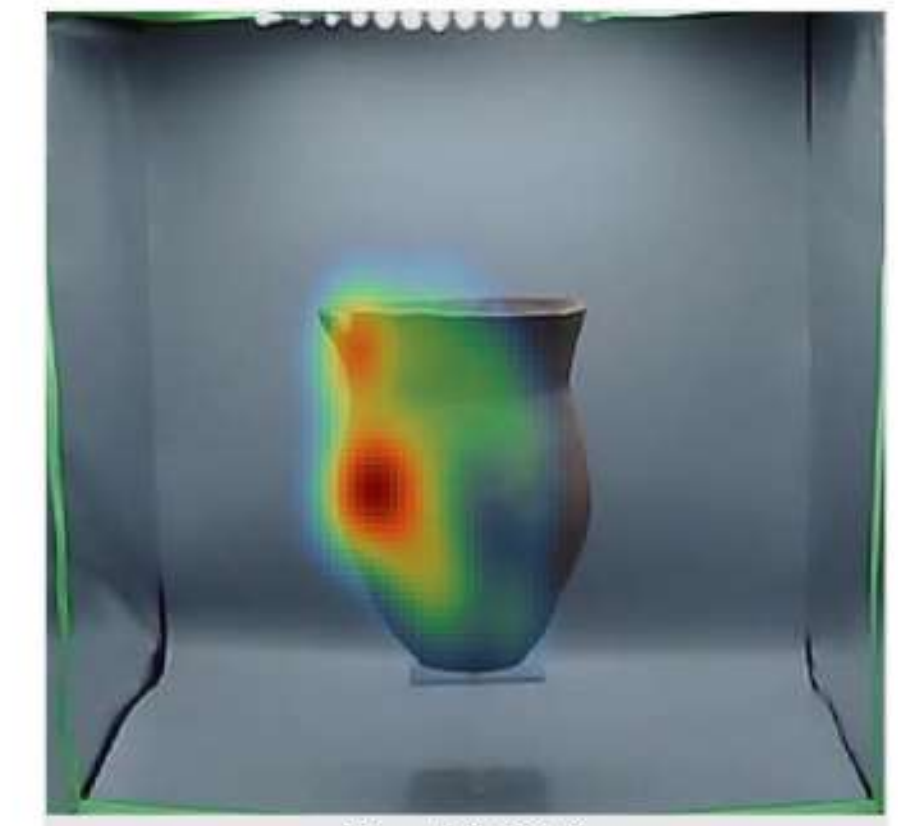
Part0101

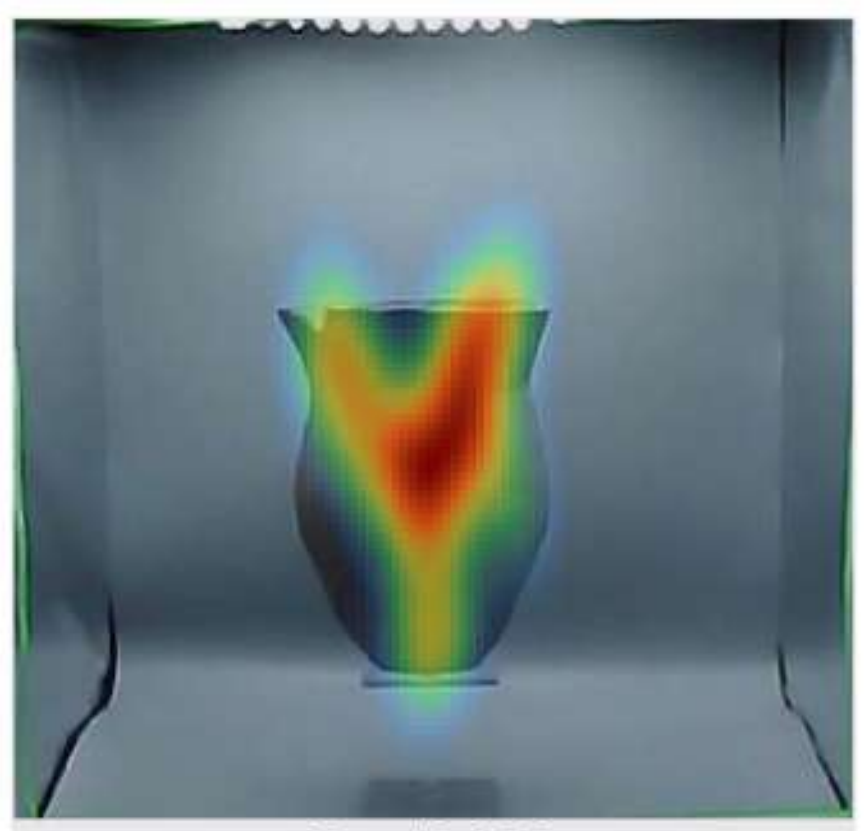
Part0102

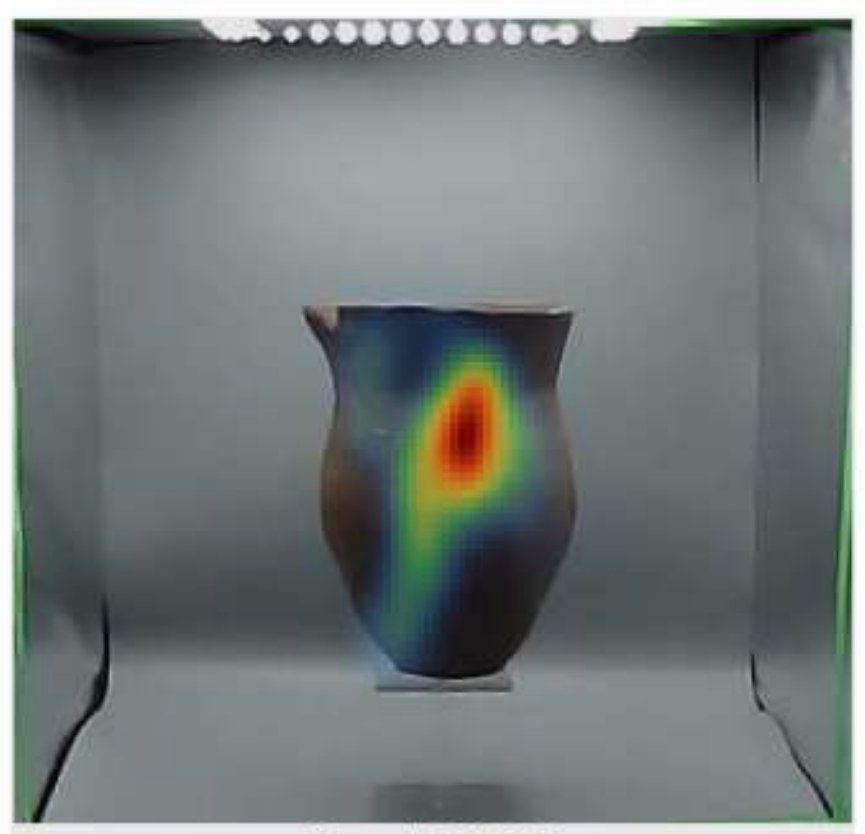
Part0103

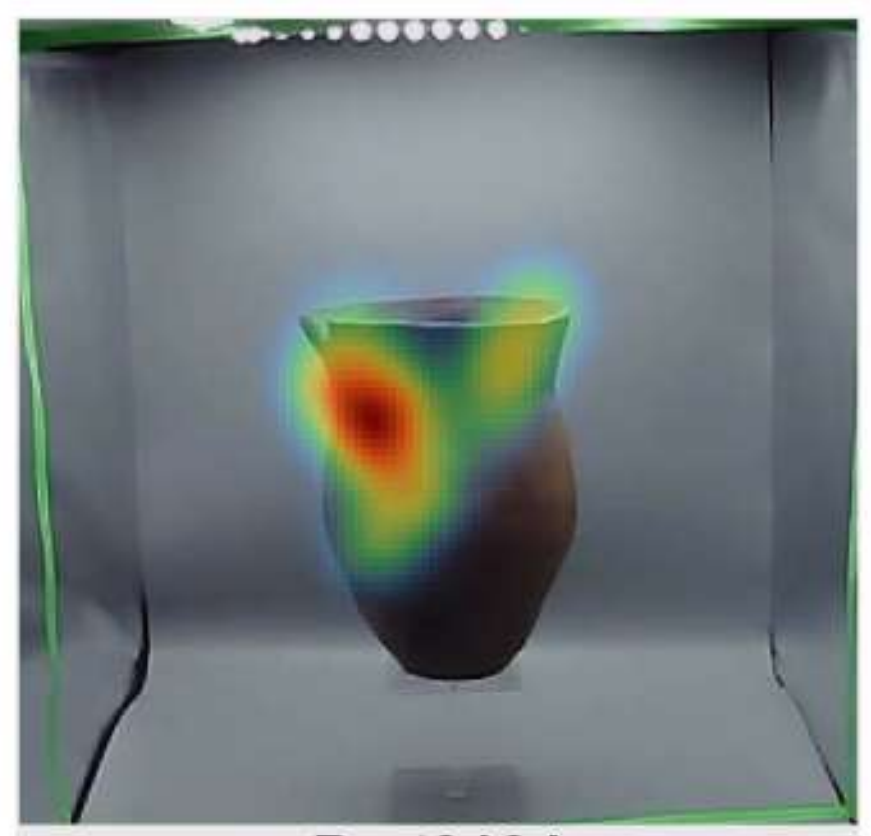
Part0104

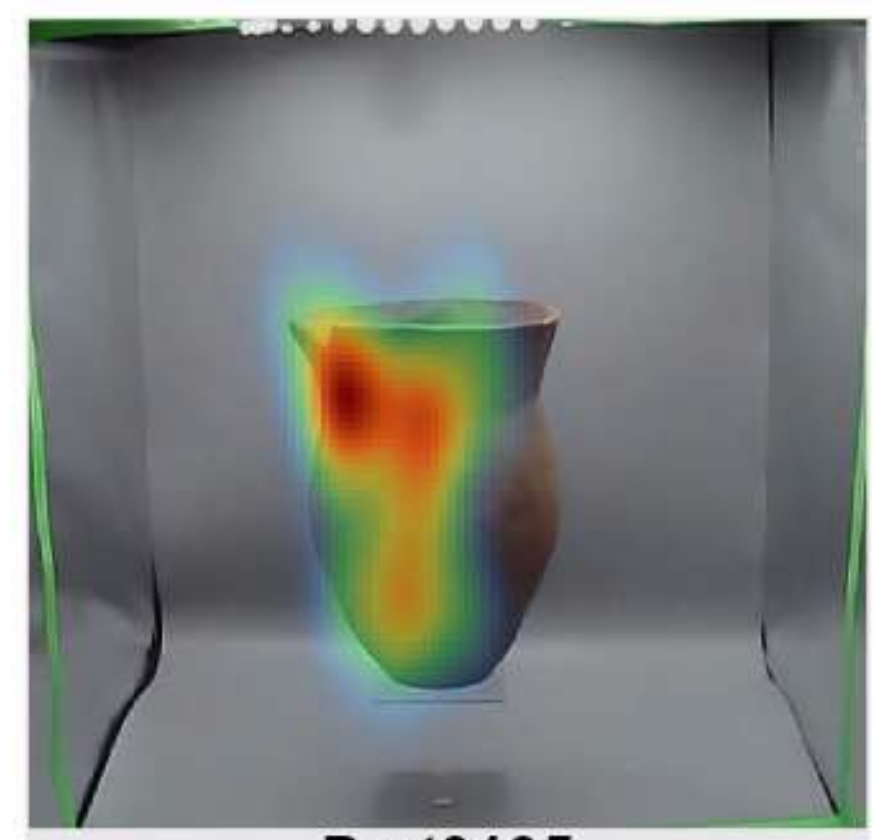
Part0105

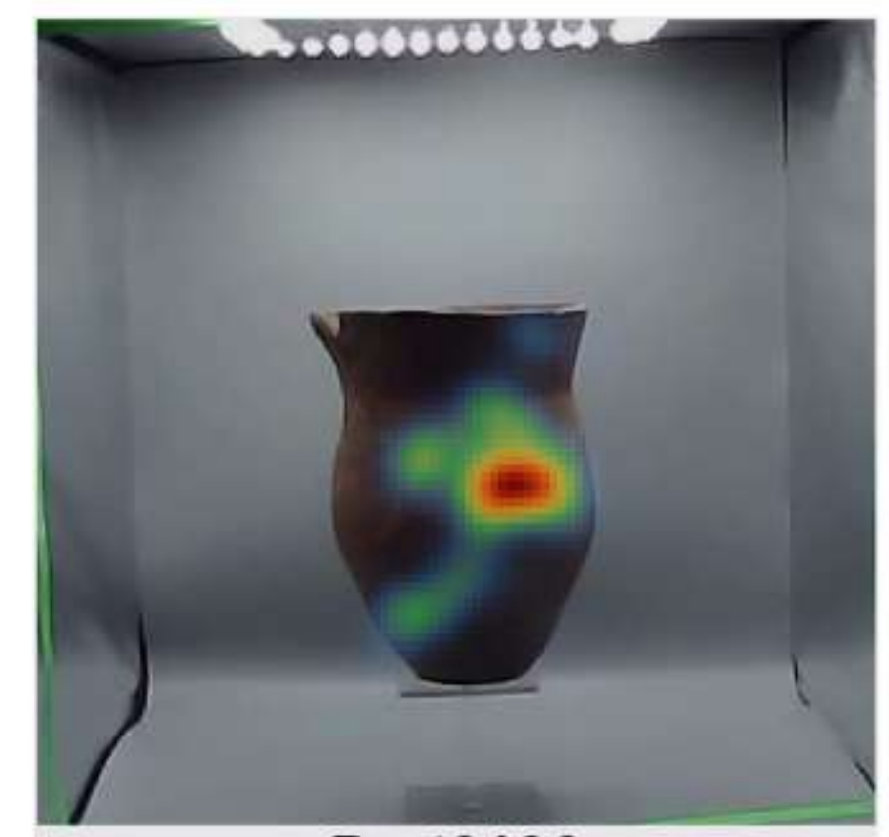
Part0106

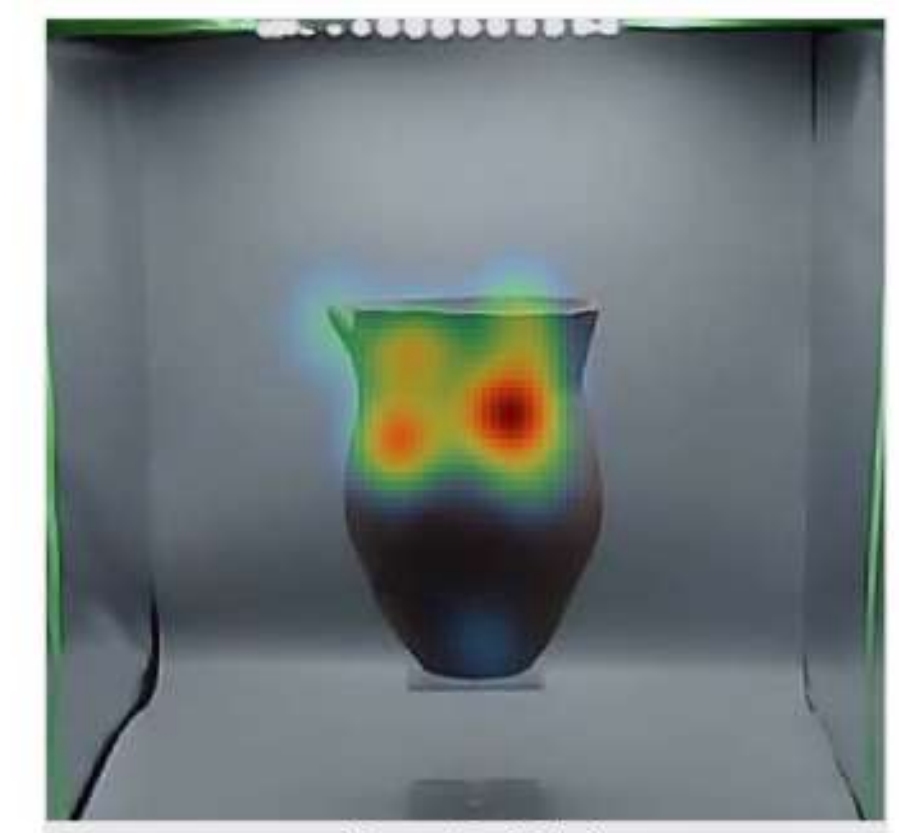
Part0107

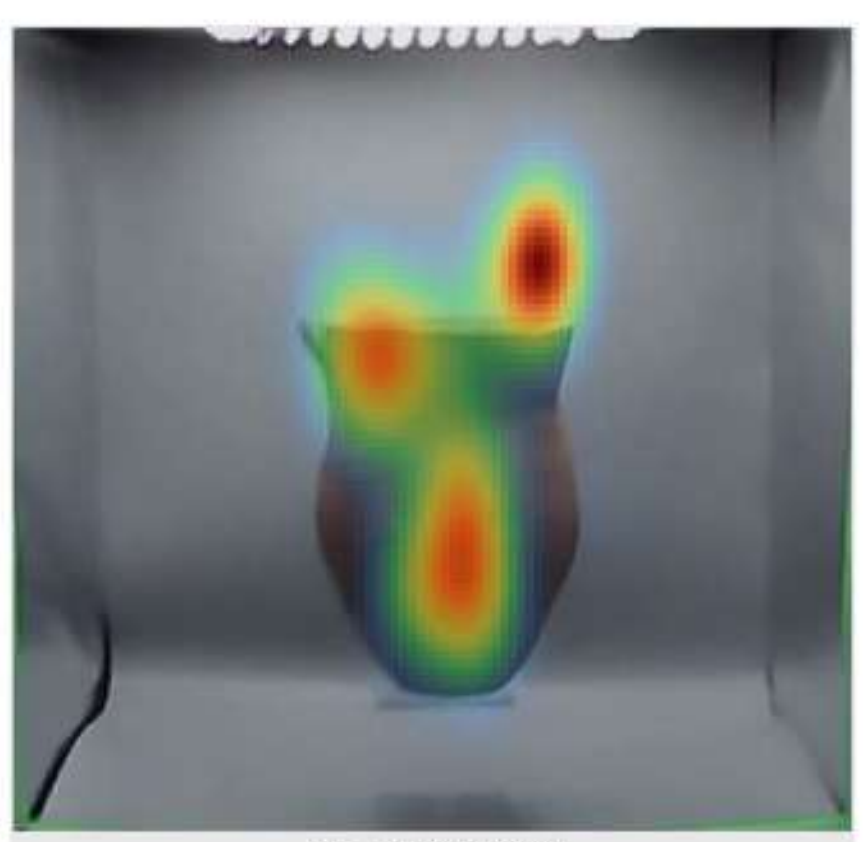
Part0108

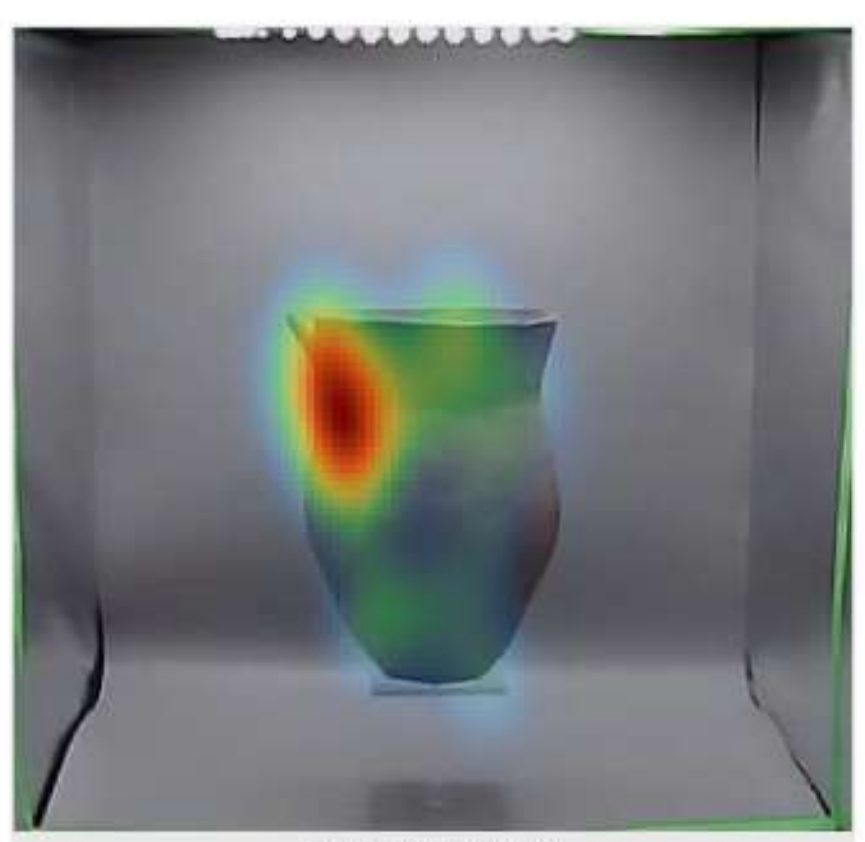
Part0109

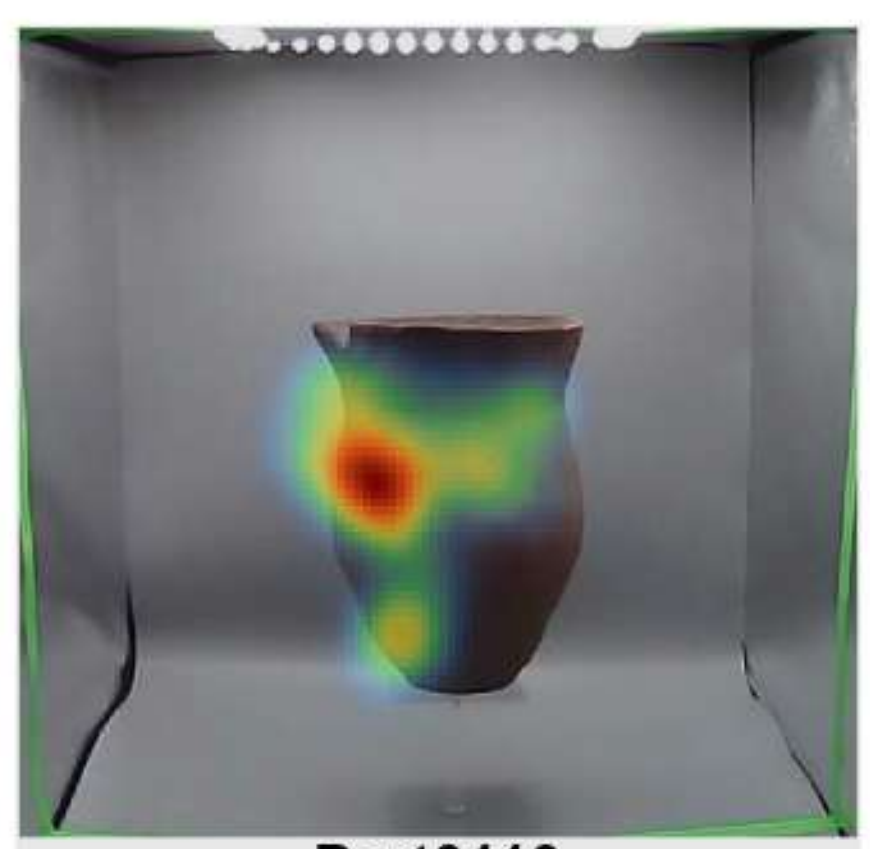
Part0110

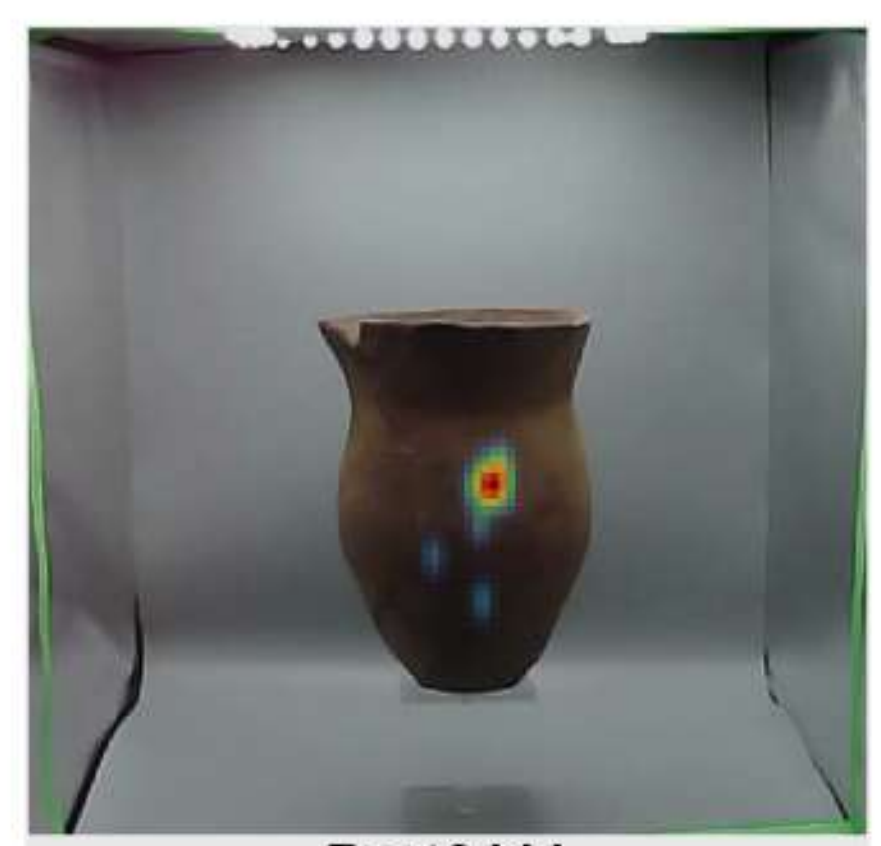
Part0111

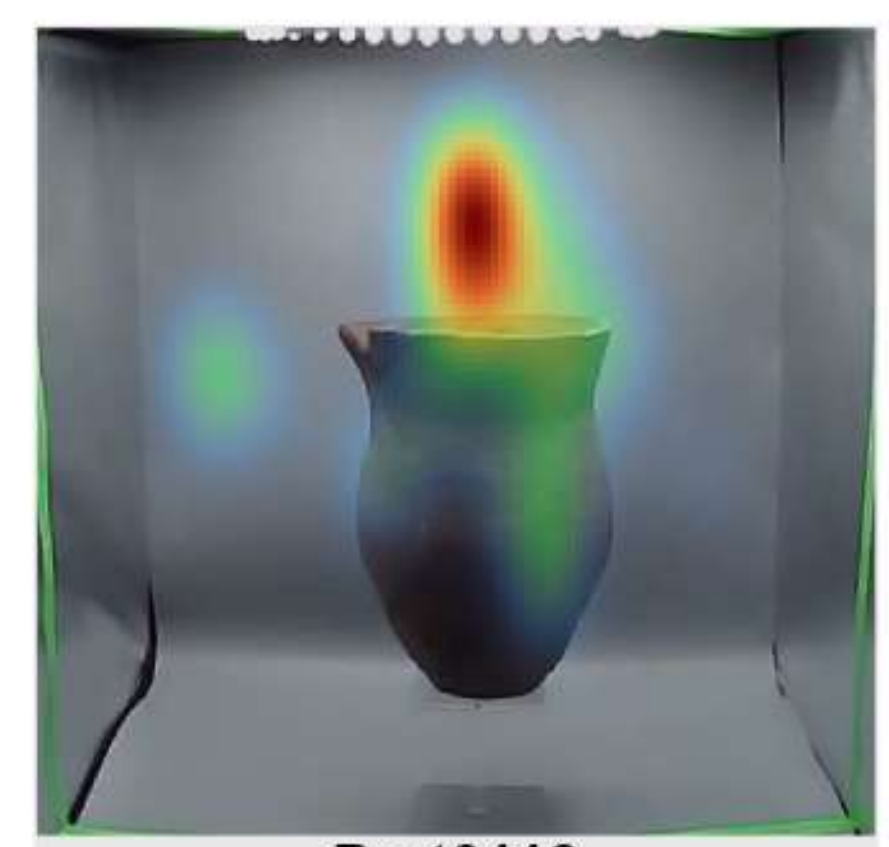
Part0112

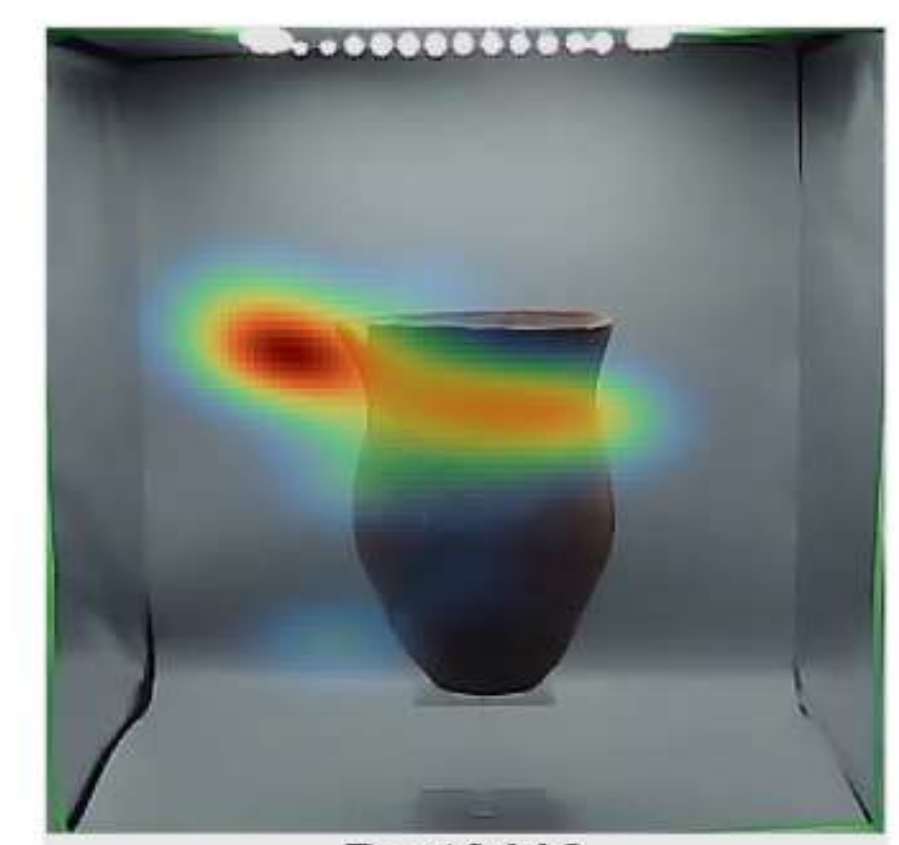
Part0113

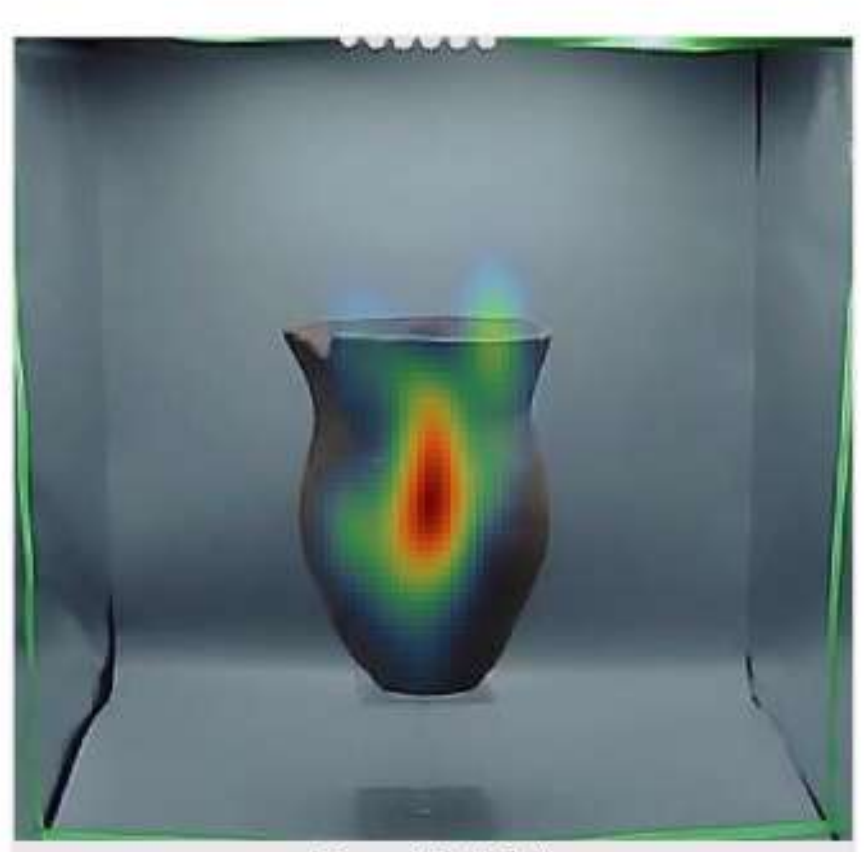
Part0114

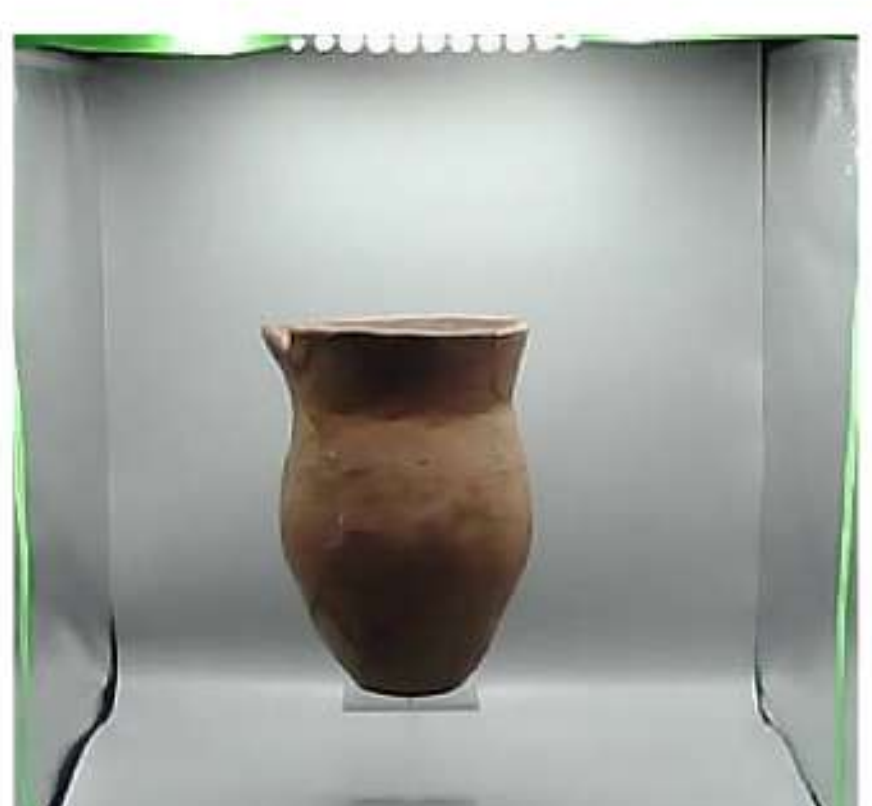
Part0115

Obj0232

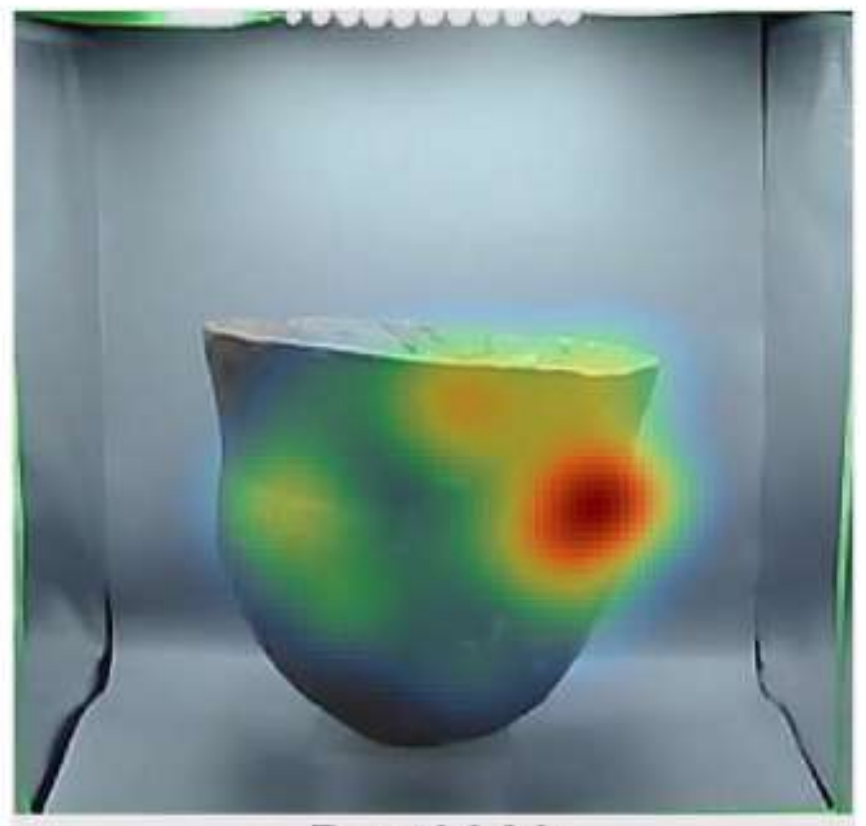
Part0089

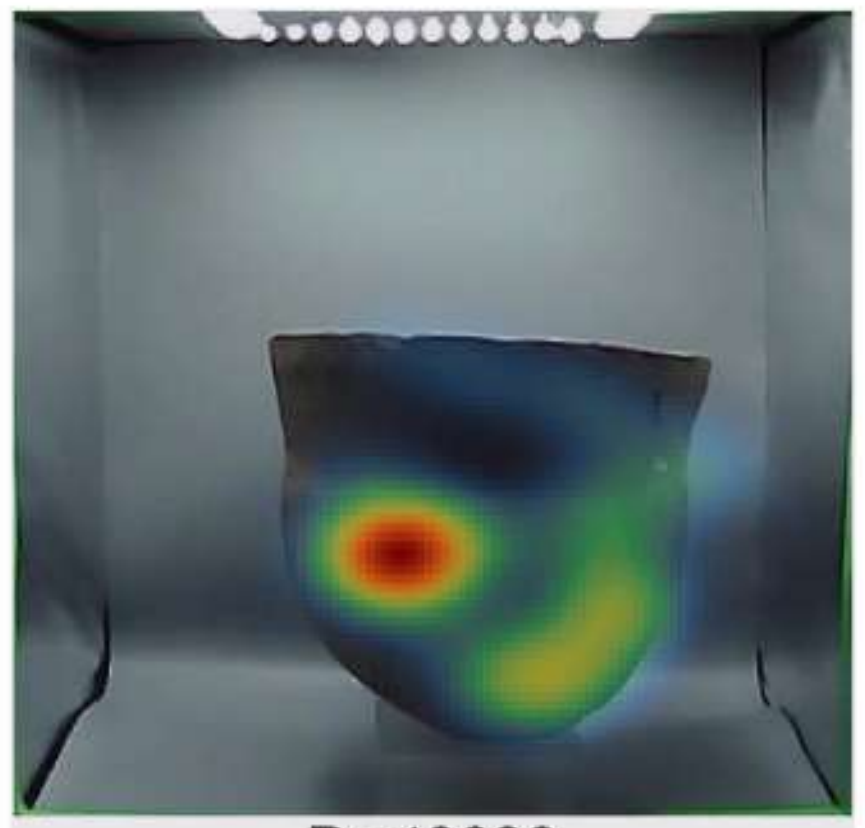
Part0090

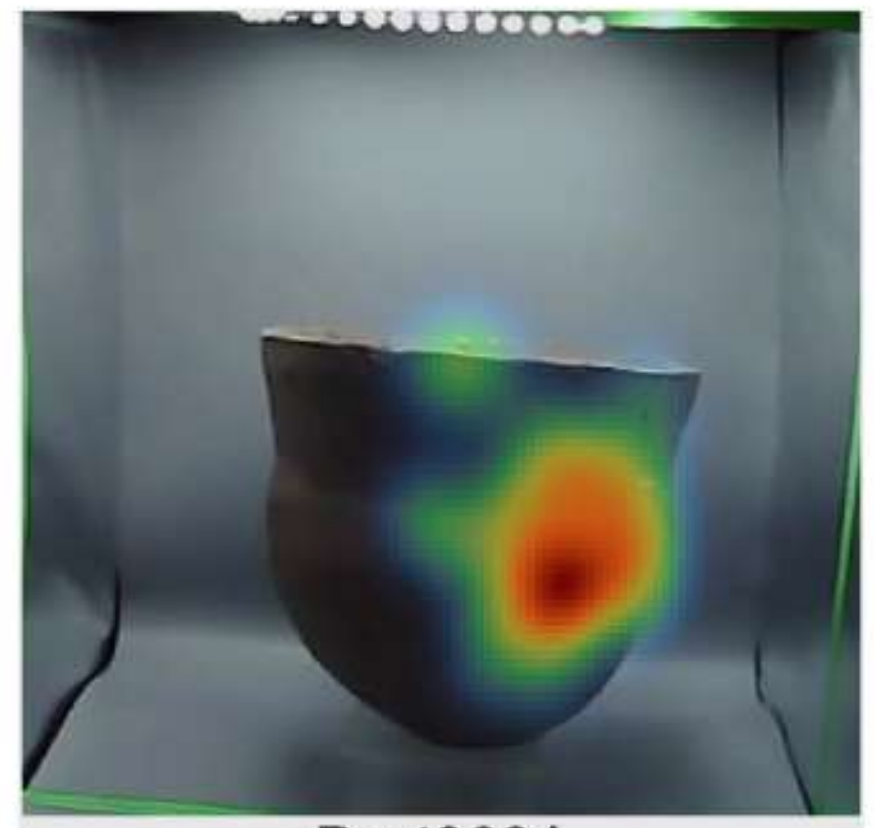
Part0091

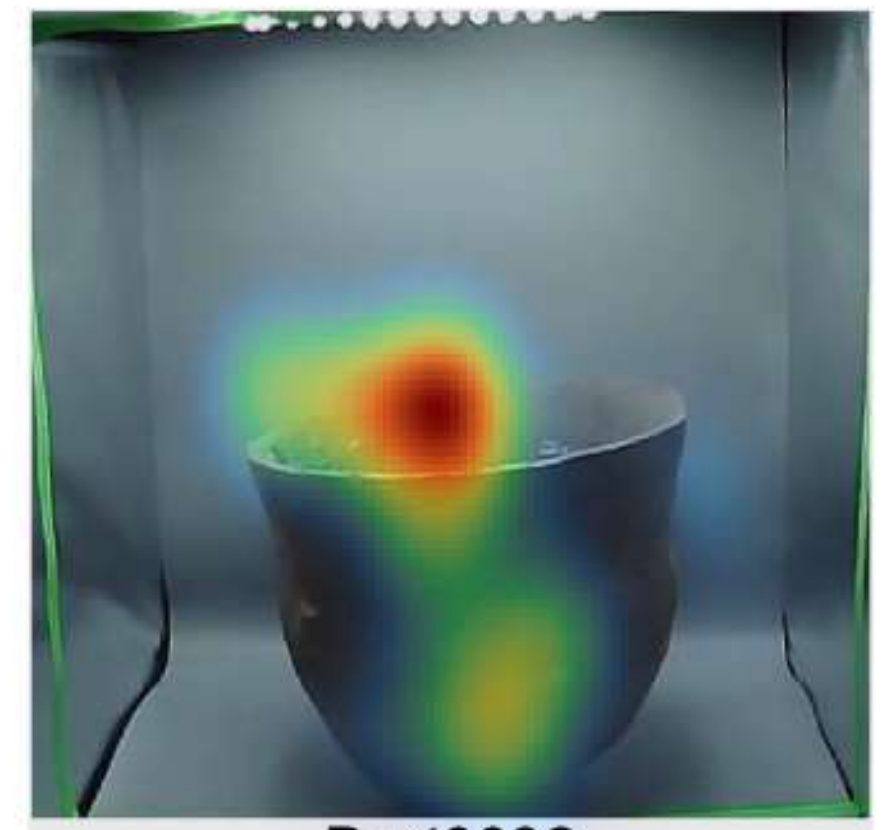
Part0092

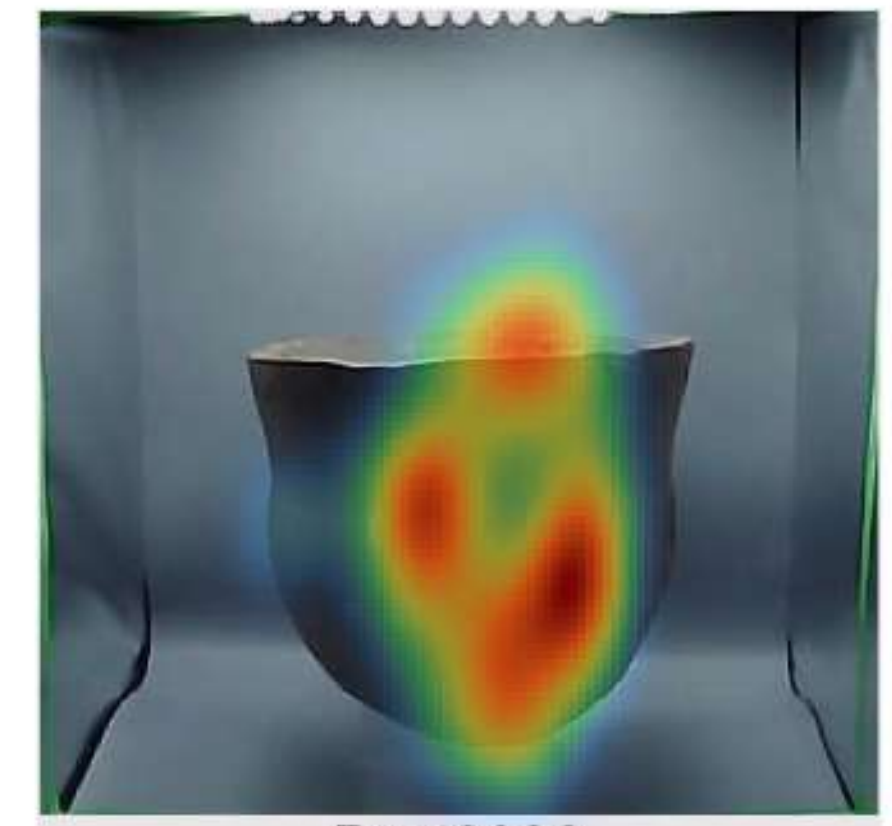
Part0093

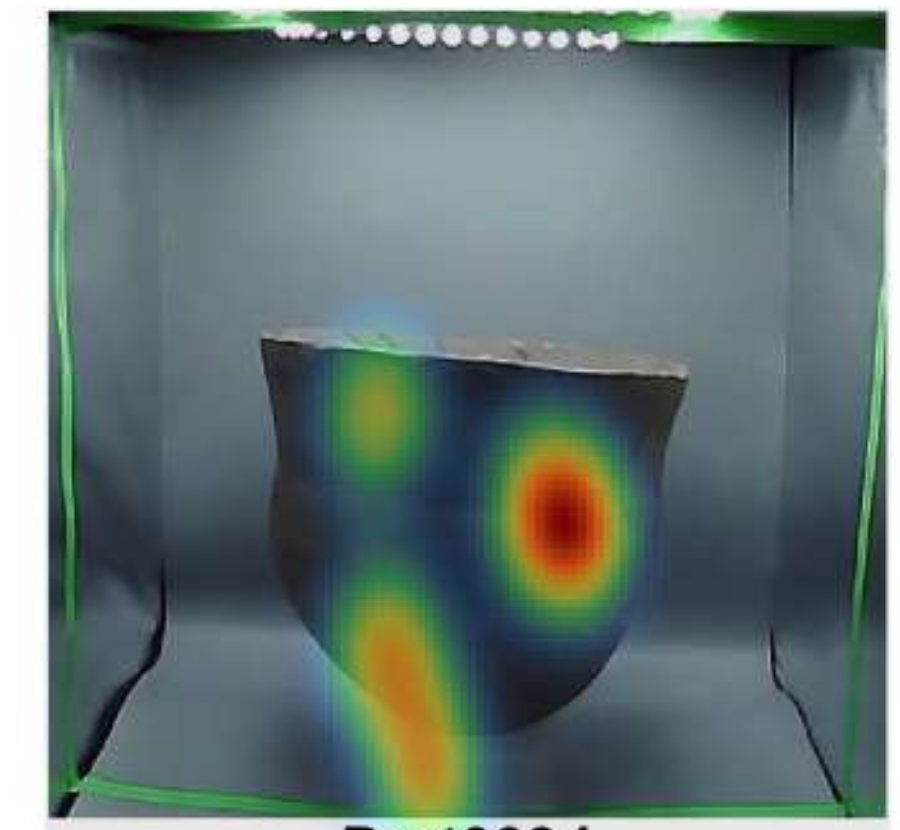
Part0094

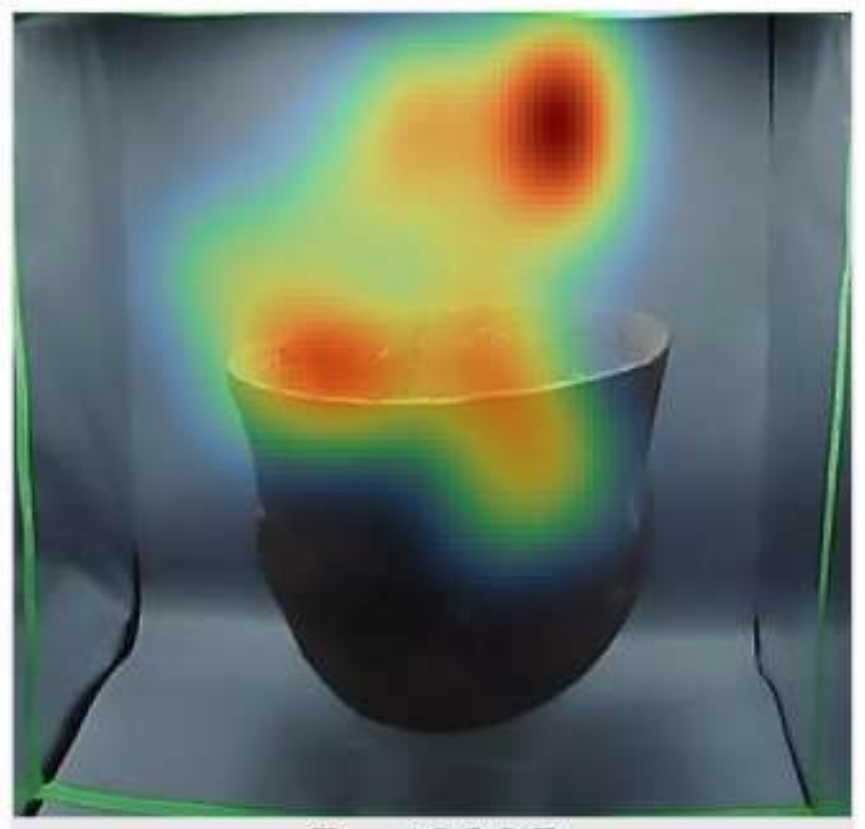
Part0095

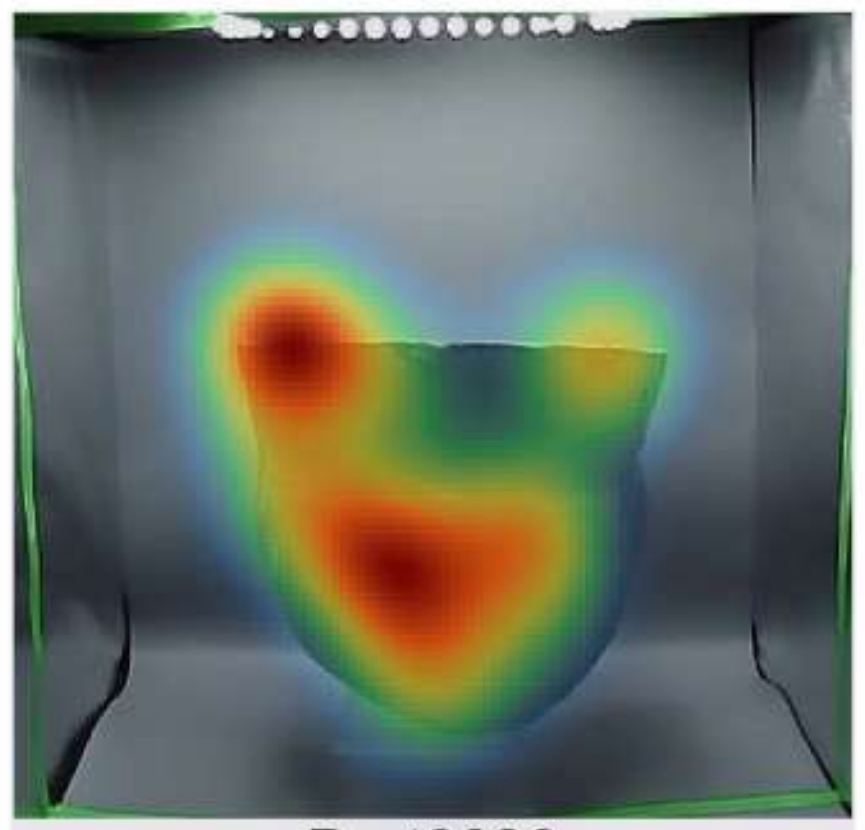
Part0096

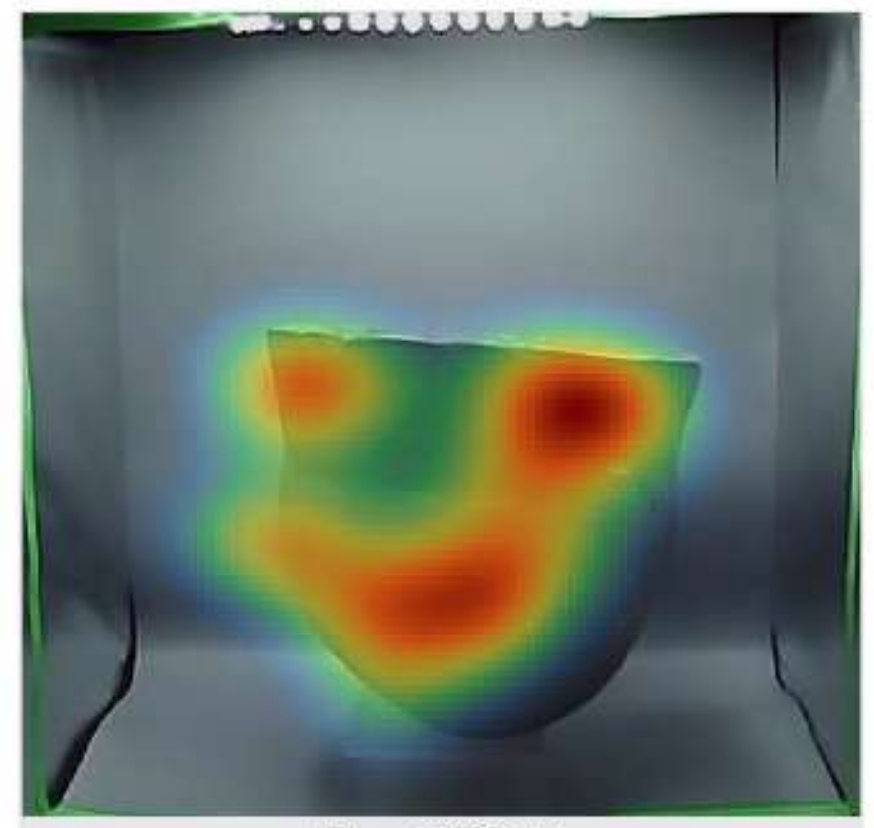
Part0097

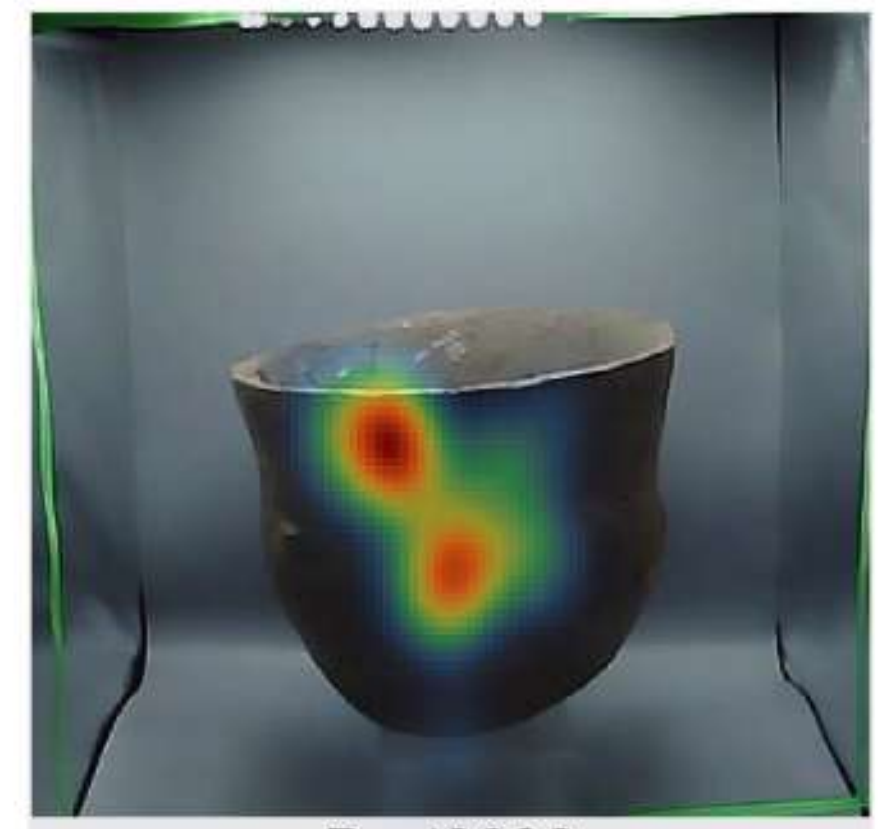
Part0098

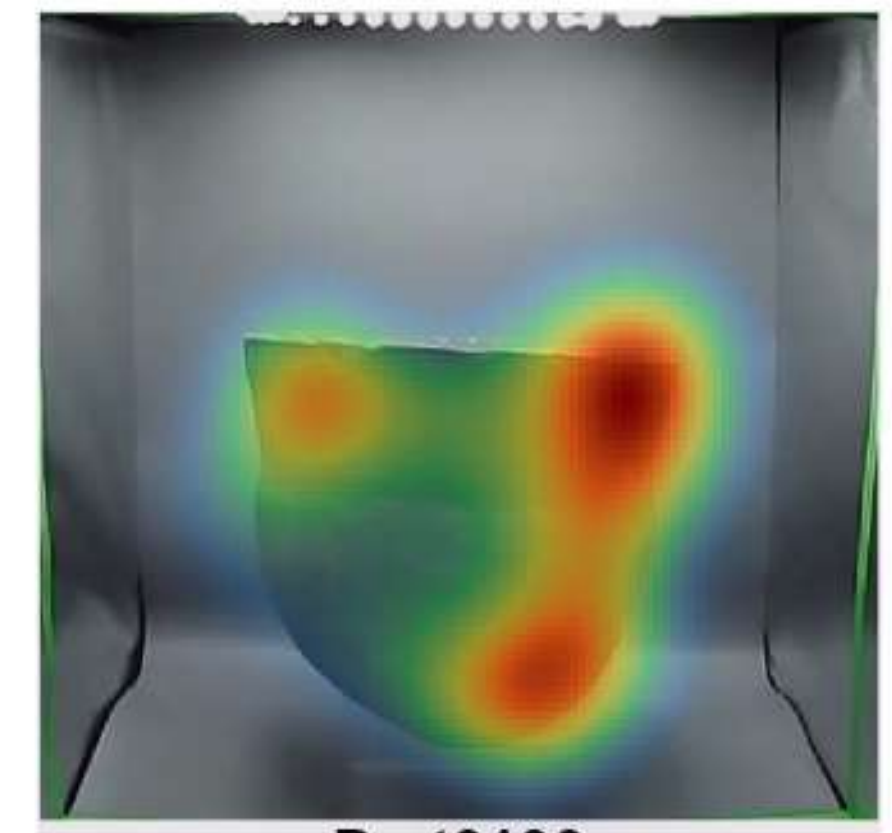
Part0100

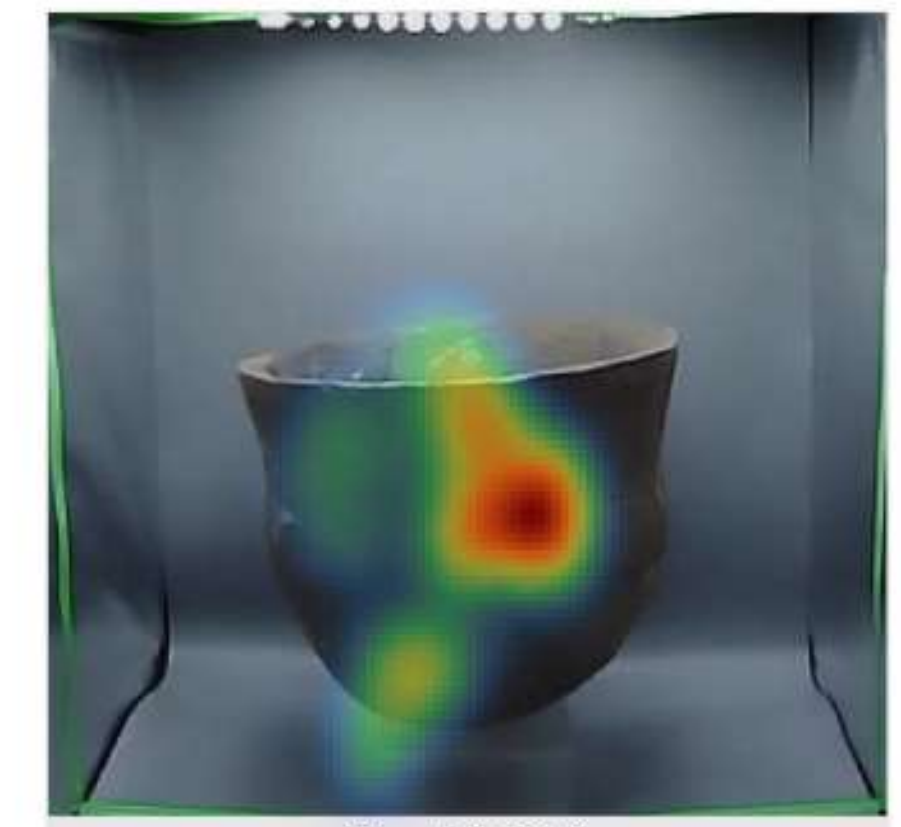
Part0101

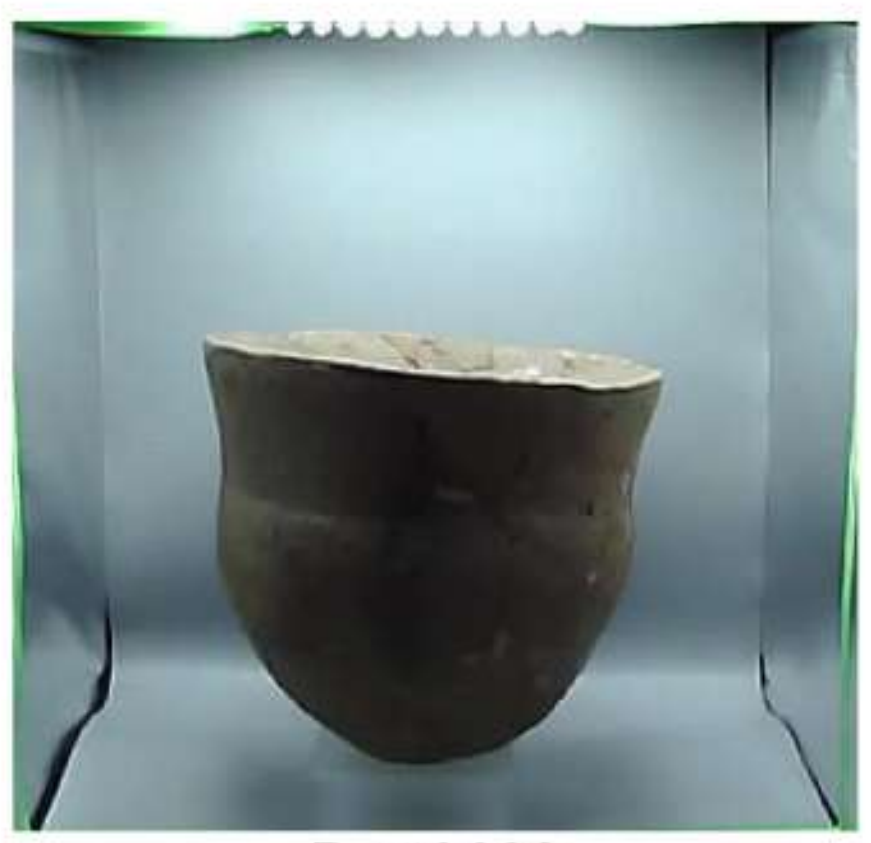
Part0102

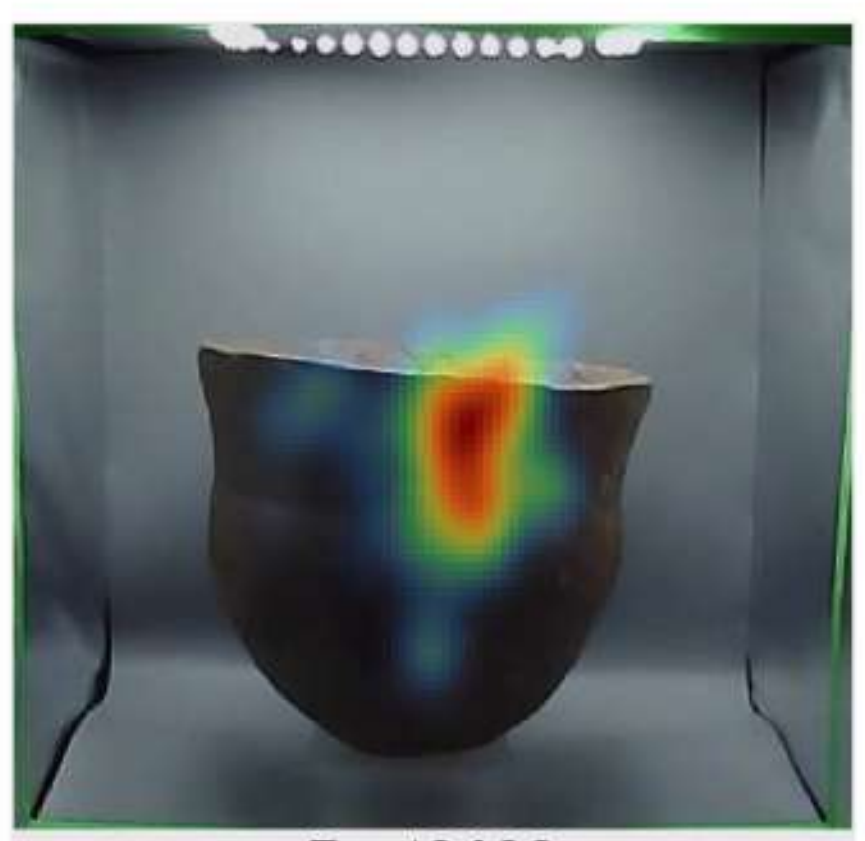
Part0103

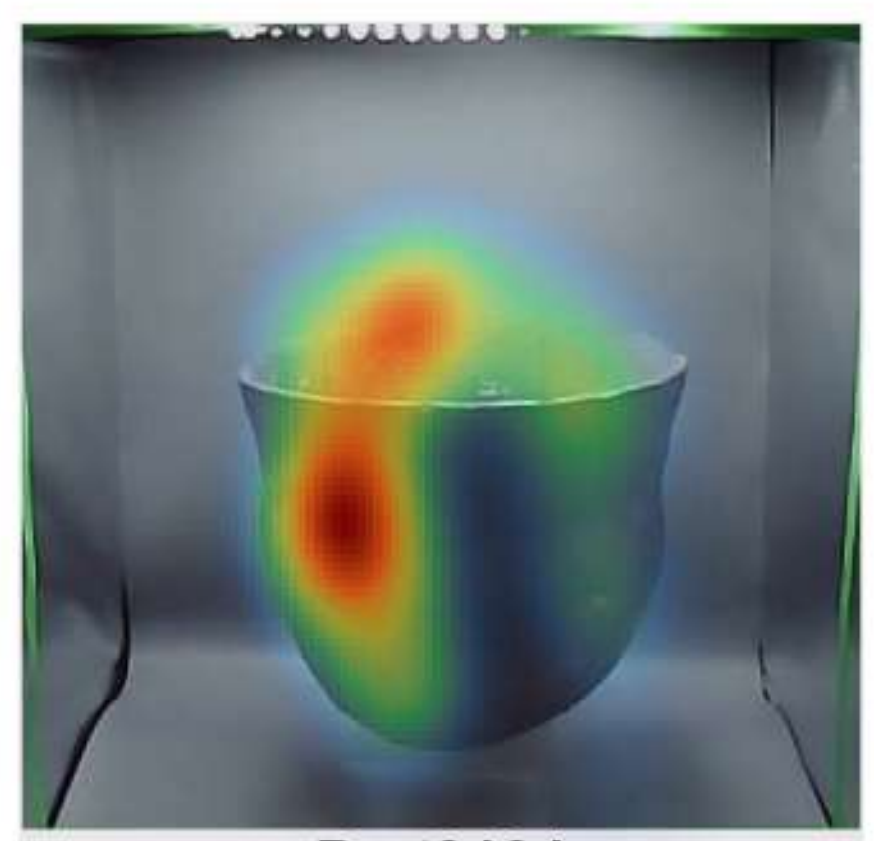
Part0104

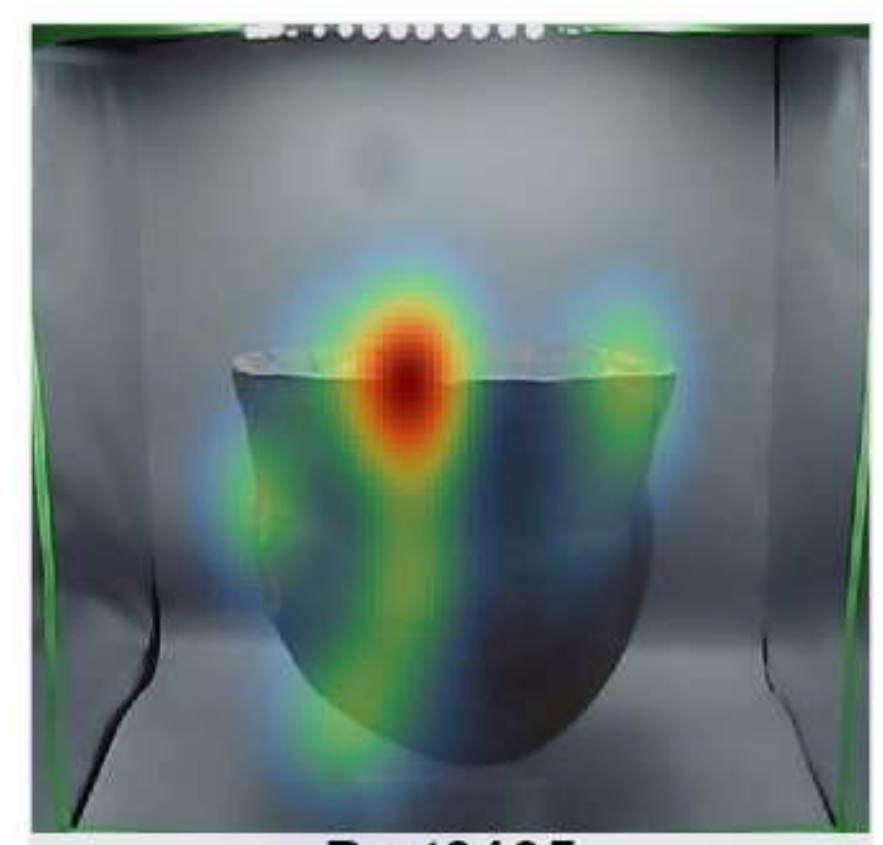
Part0105

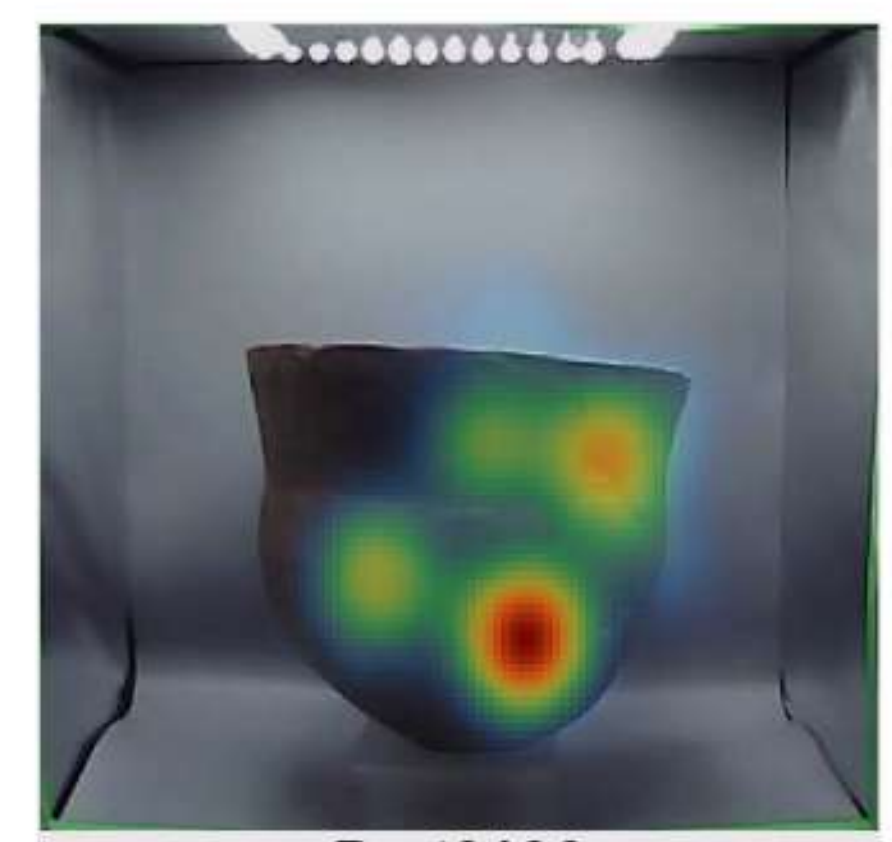
Part0106

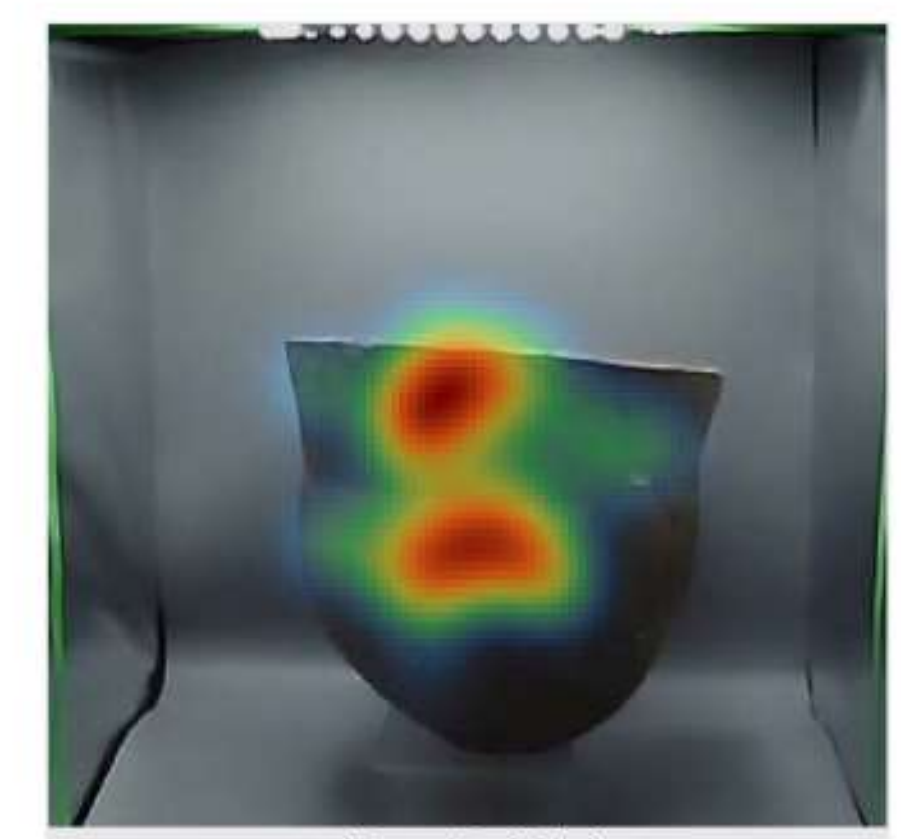
Part0107

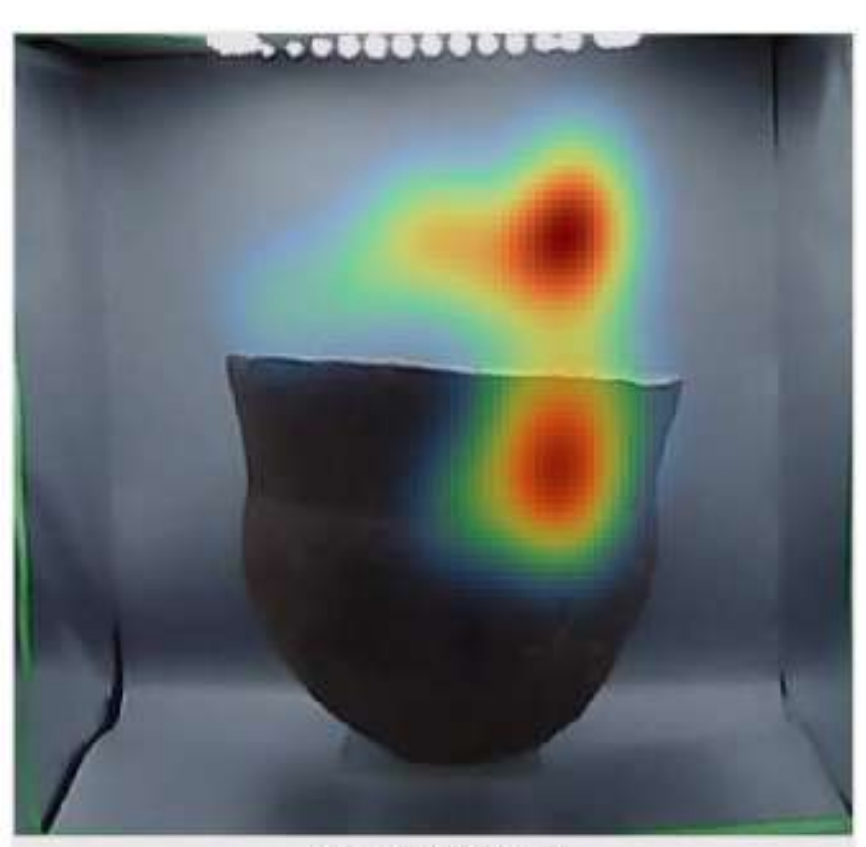
Part0108

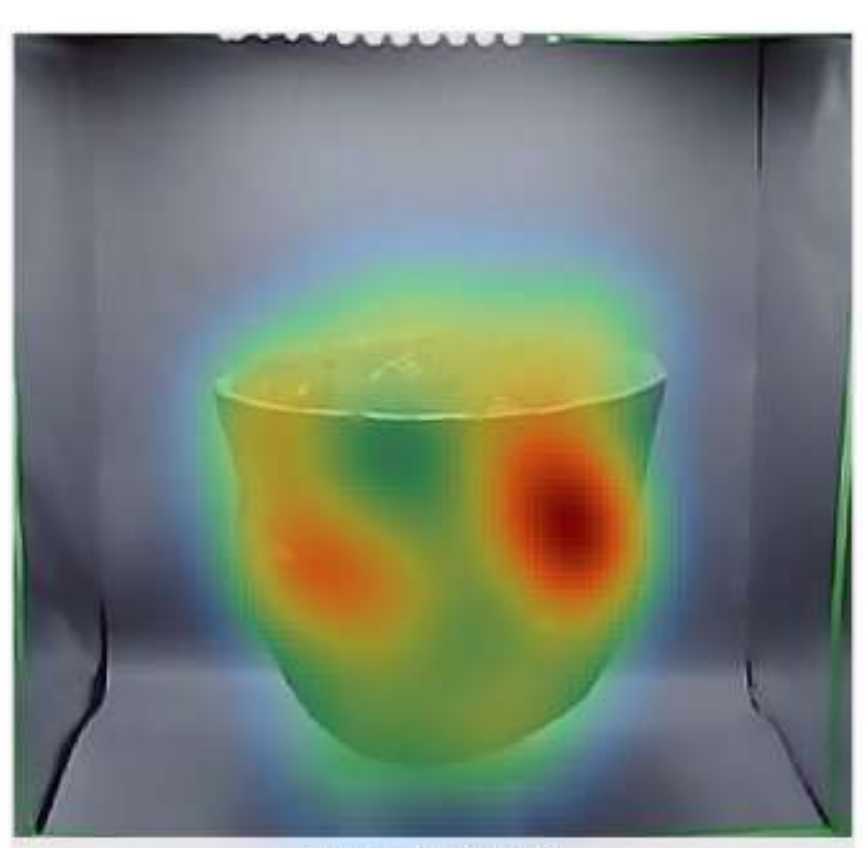
Part0109

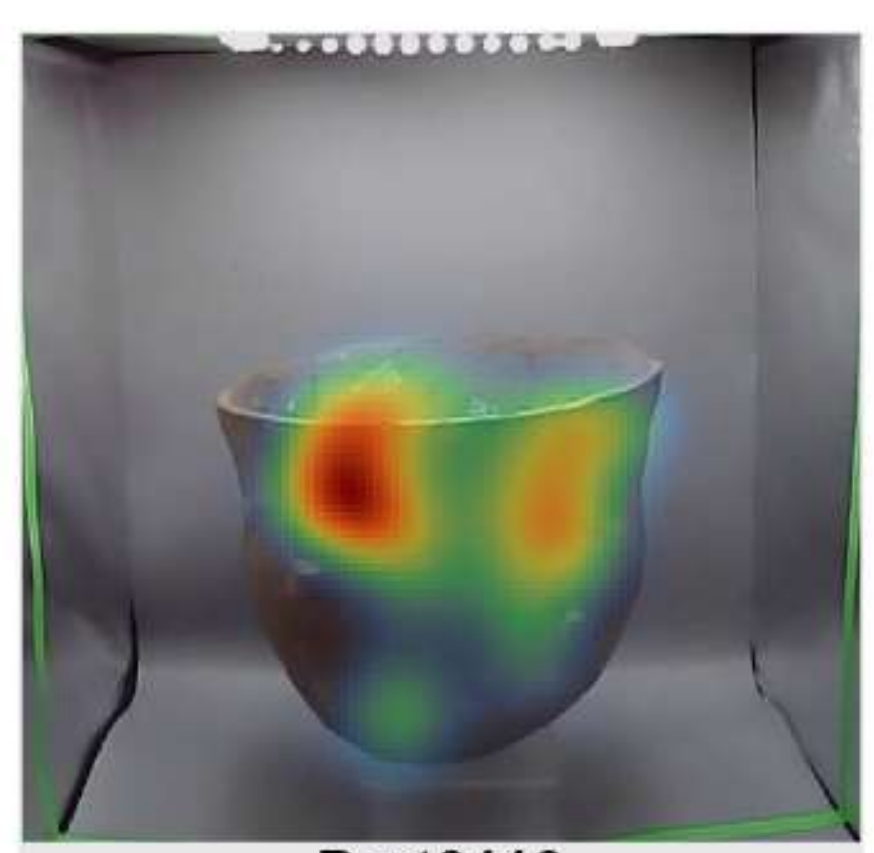
Part0110

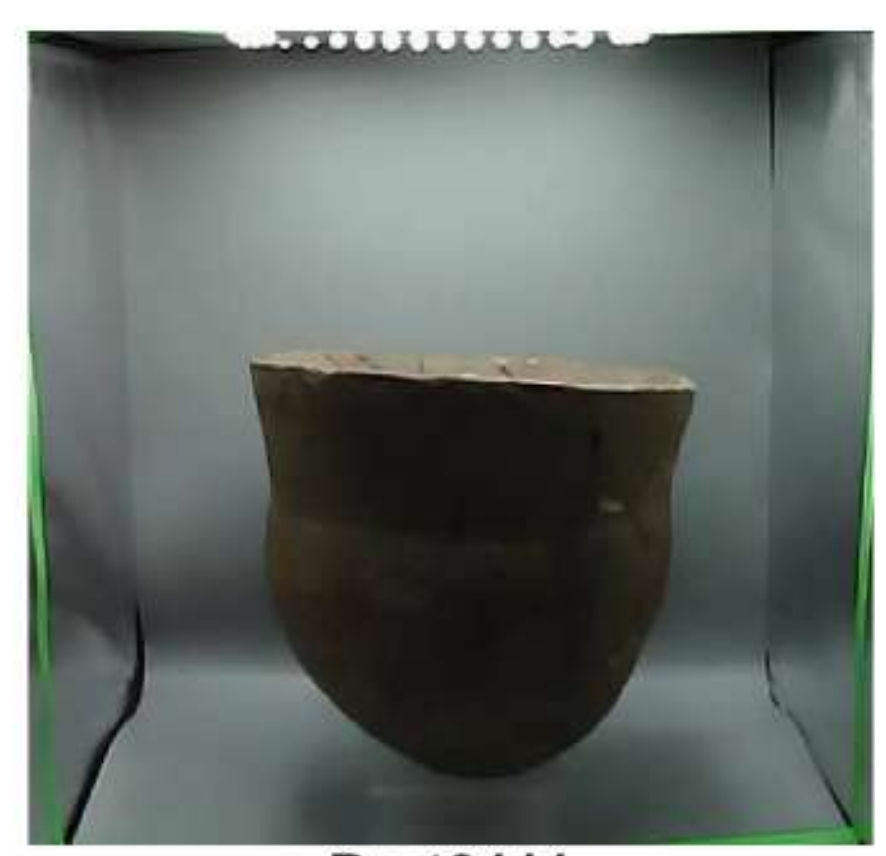
Part0111

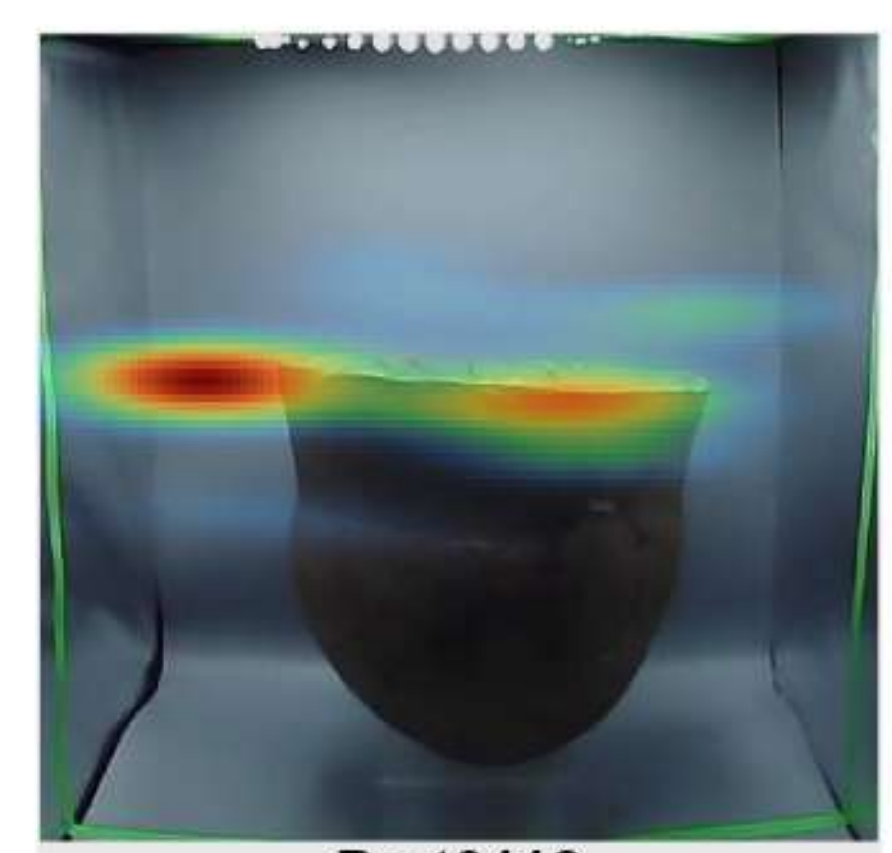
Part0112

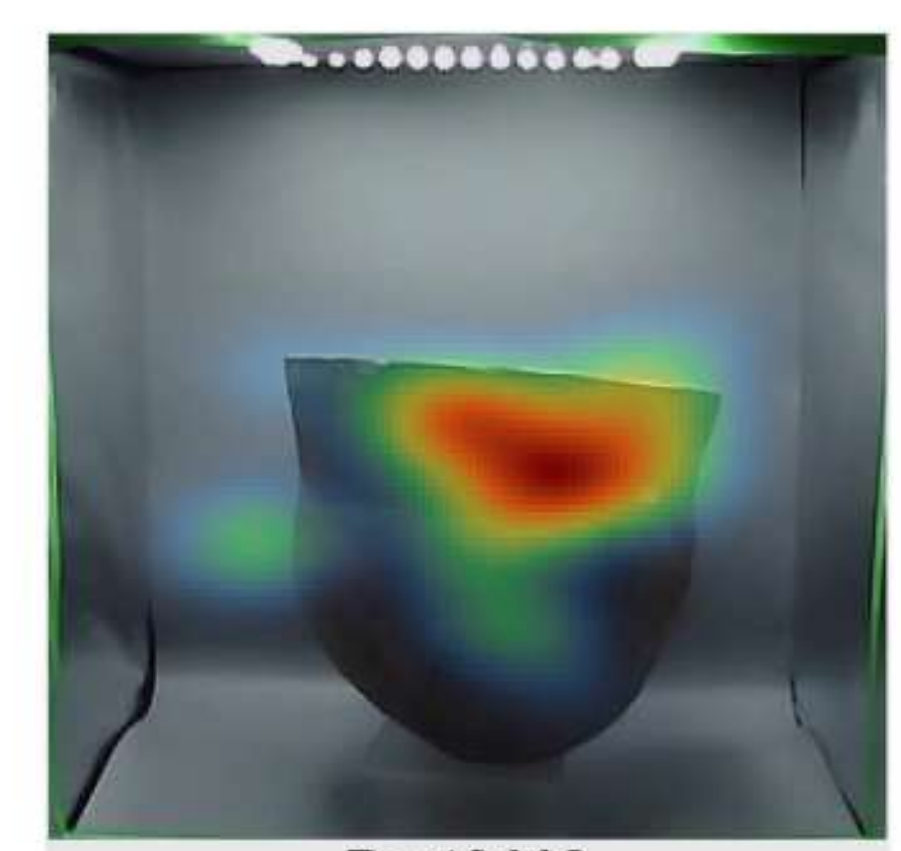
Part0113

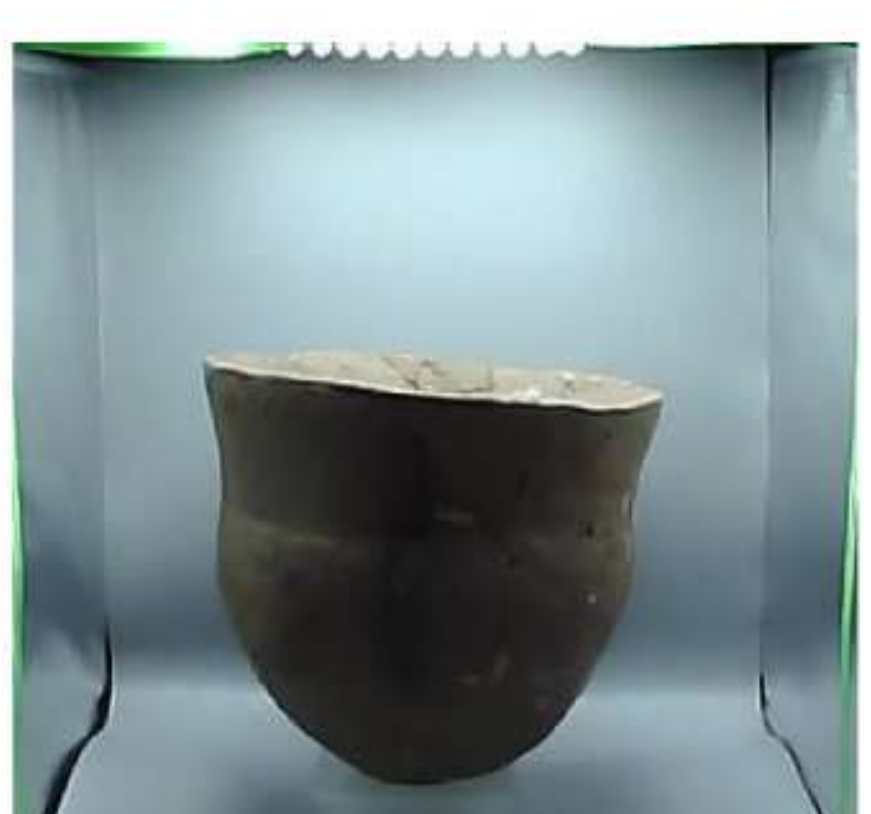
Part0114

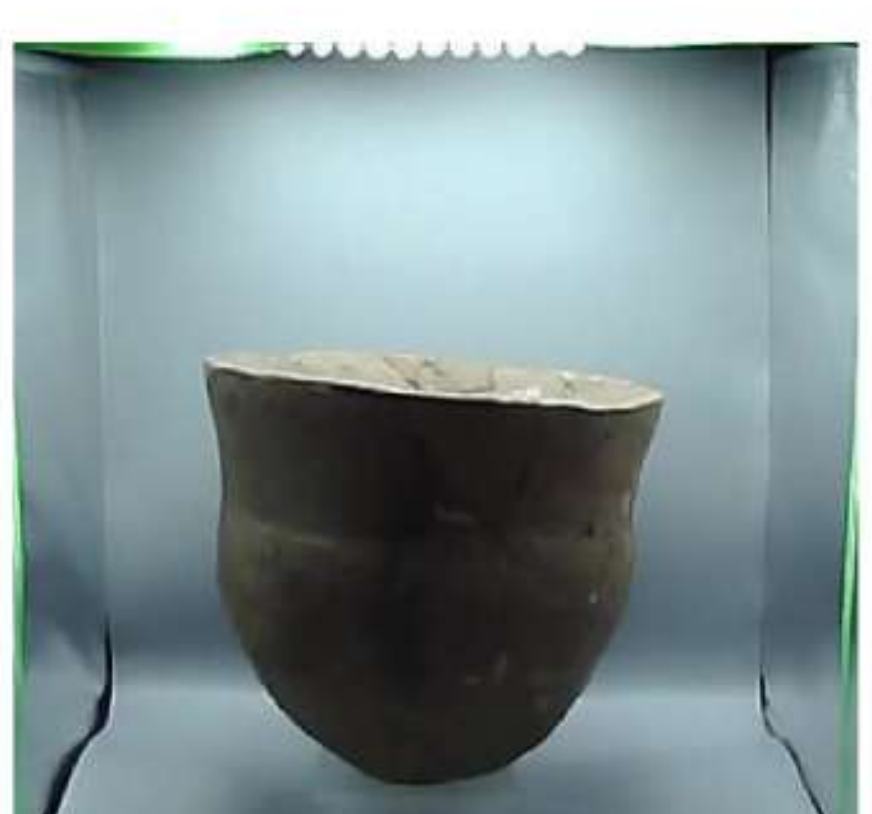
Part0115

Obj0234

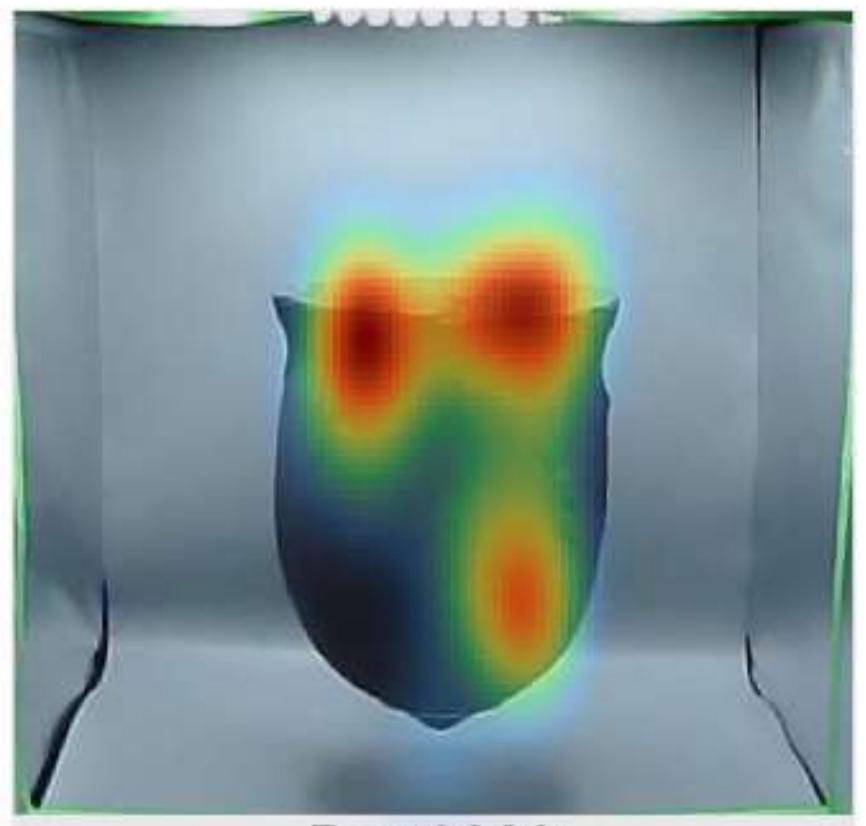
Part0089
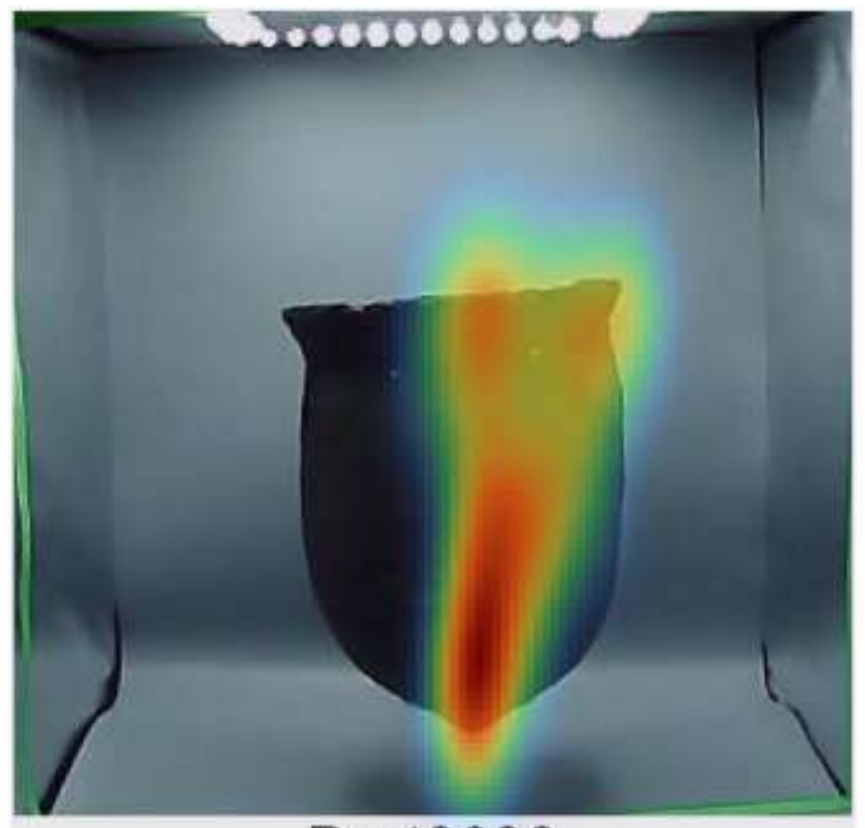
Part0090
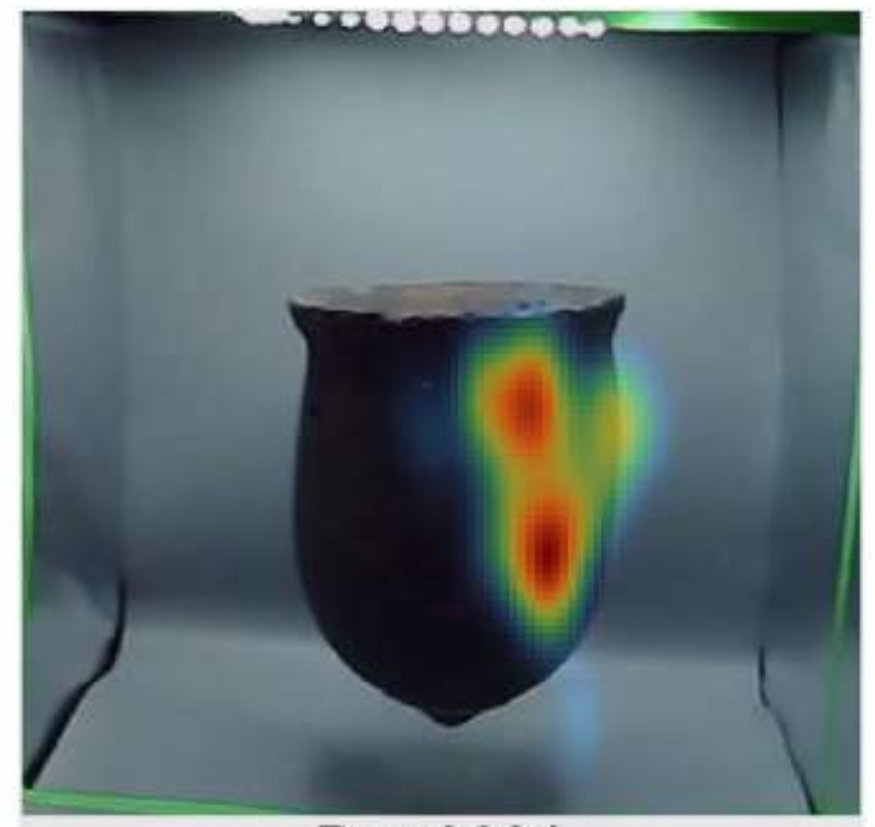
Part0091
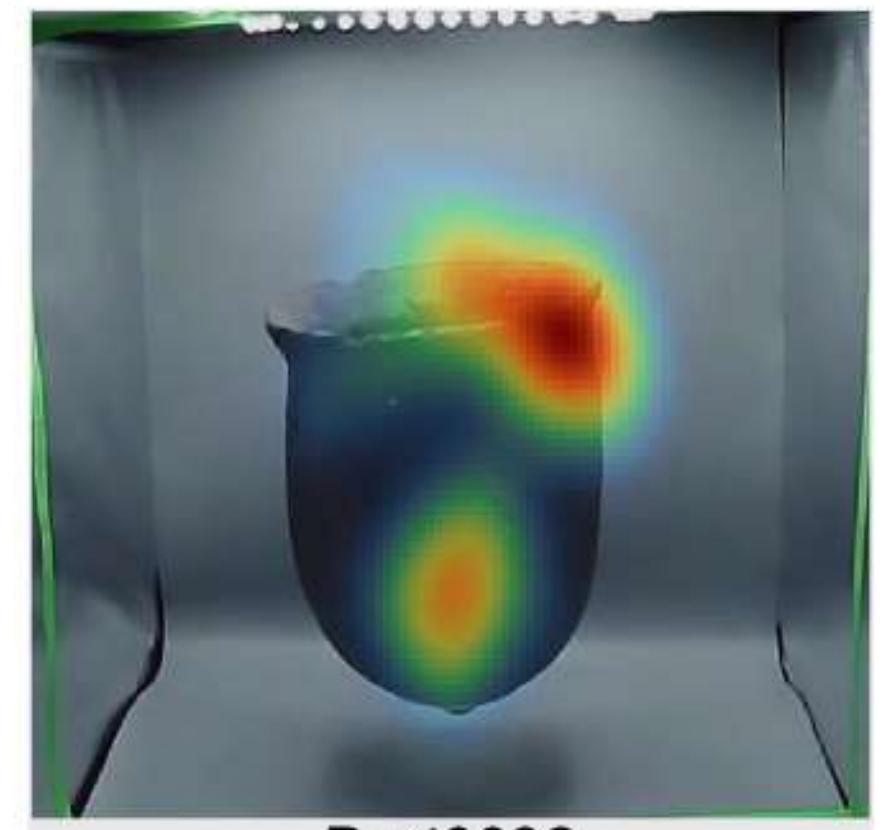
Part0092
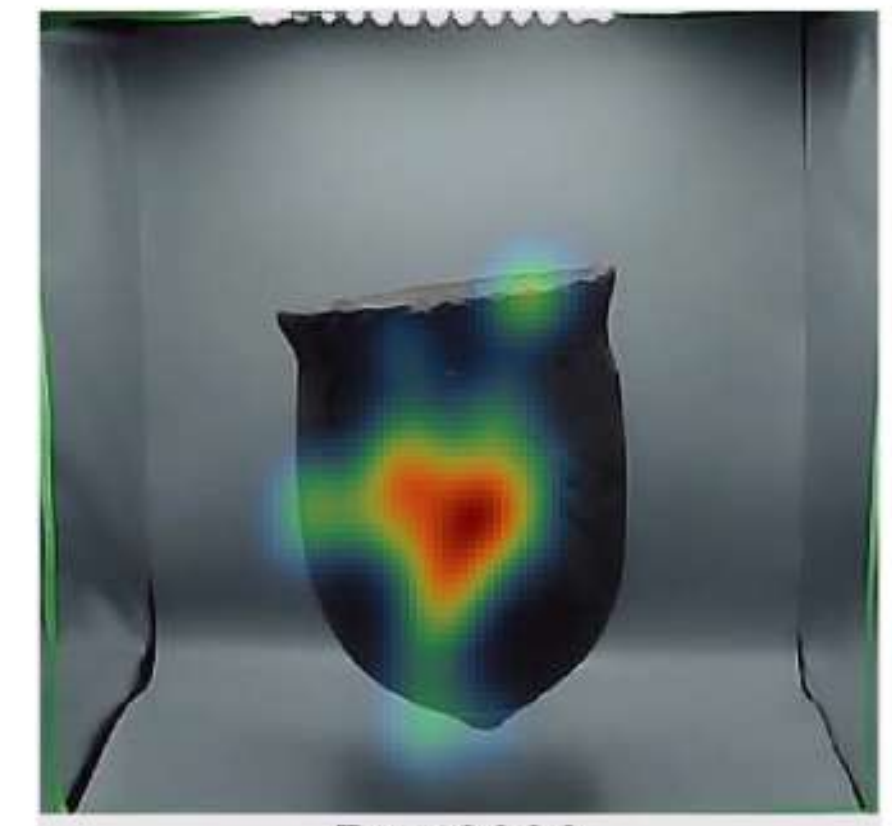
Part0093
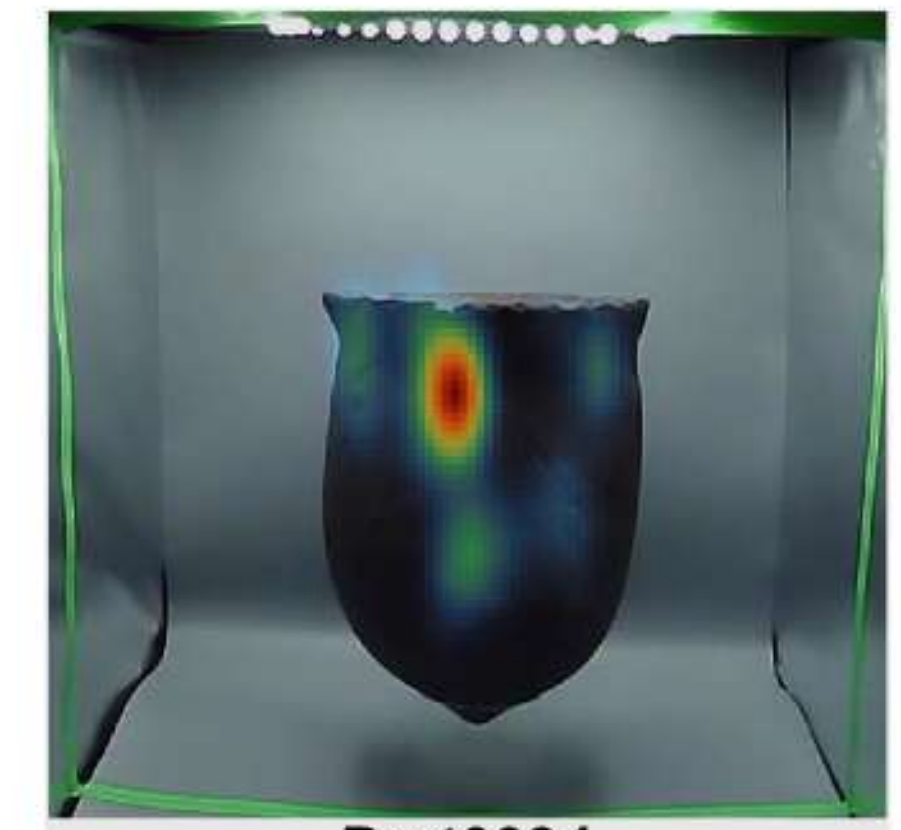
Part0094
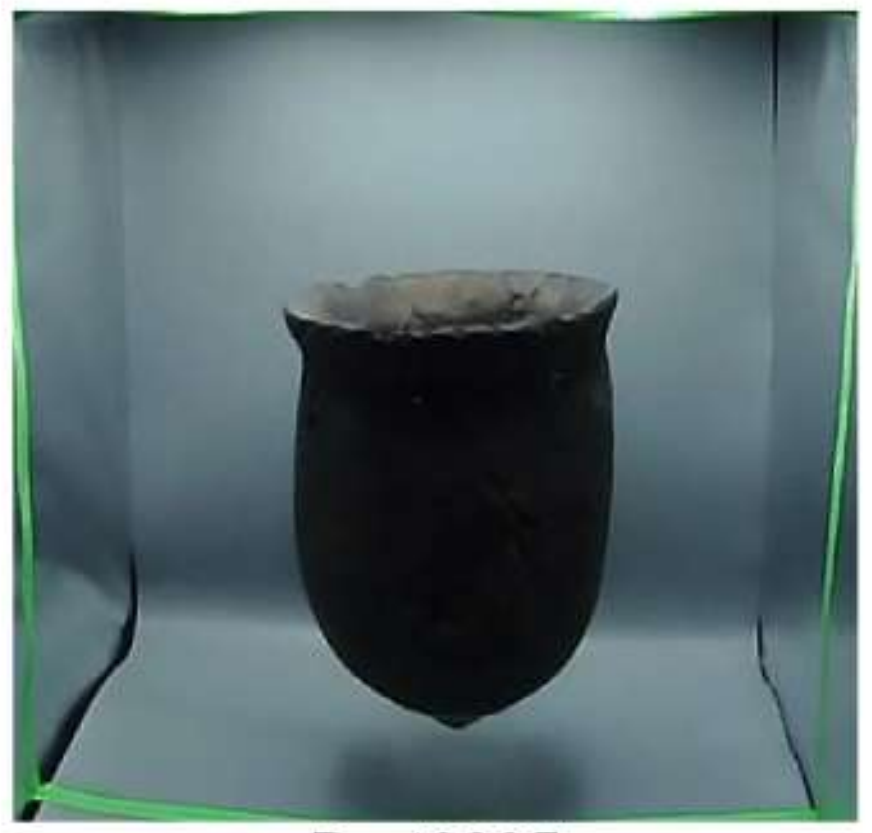
Part0095
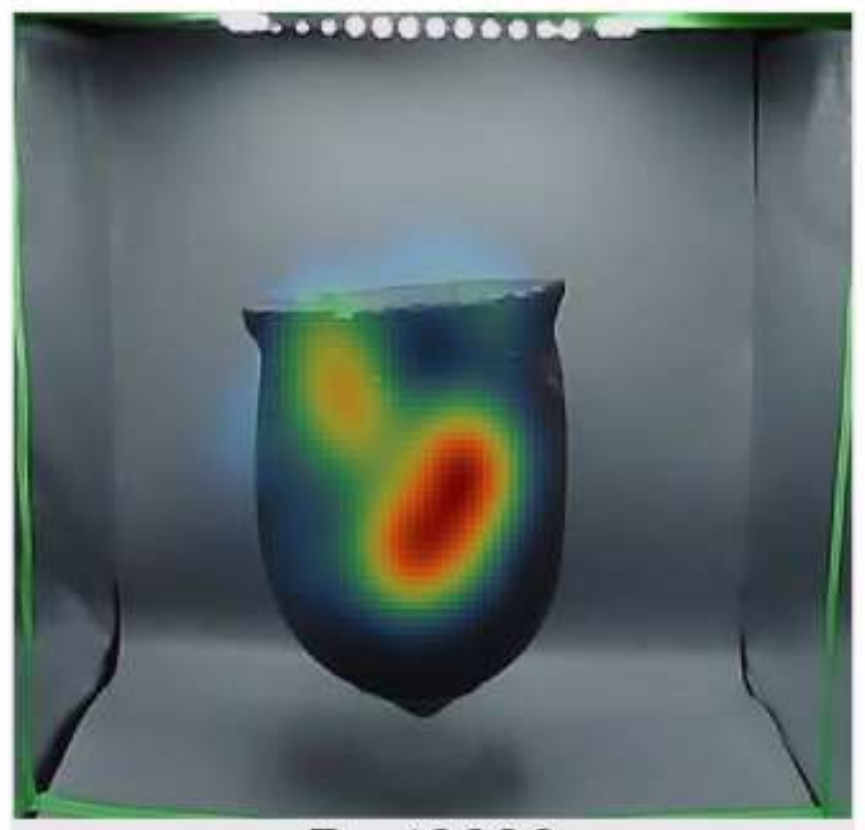
Part0096
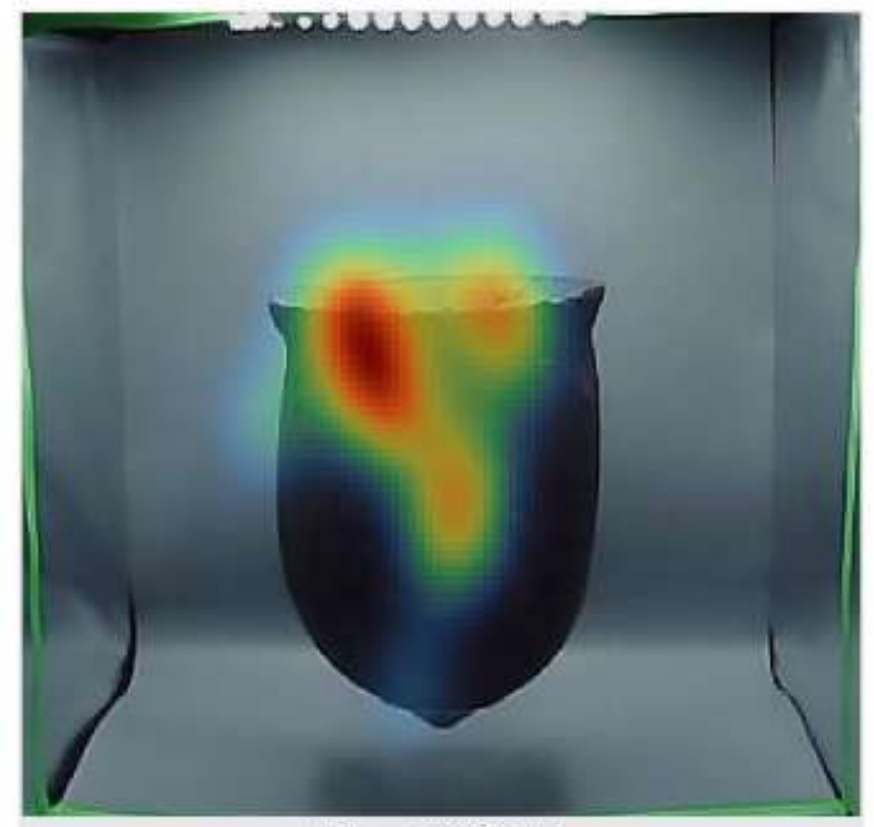
Part0097
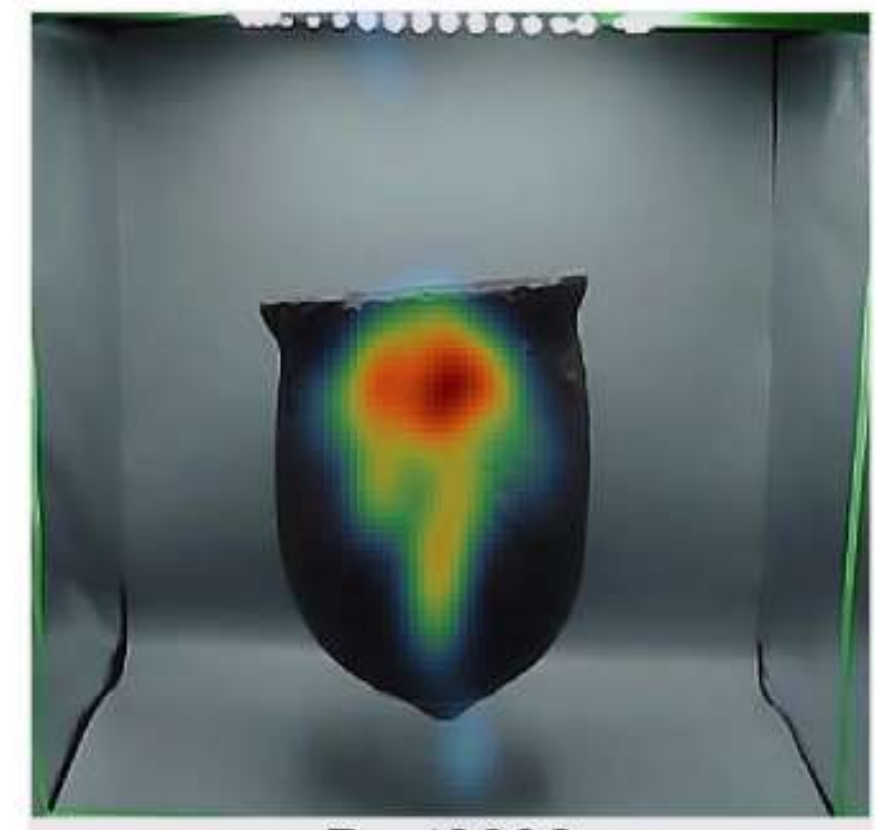
Part0098
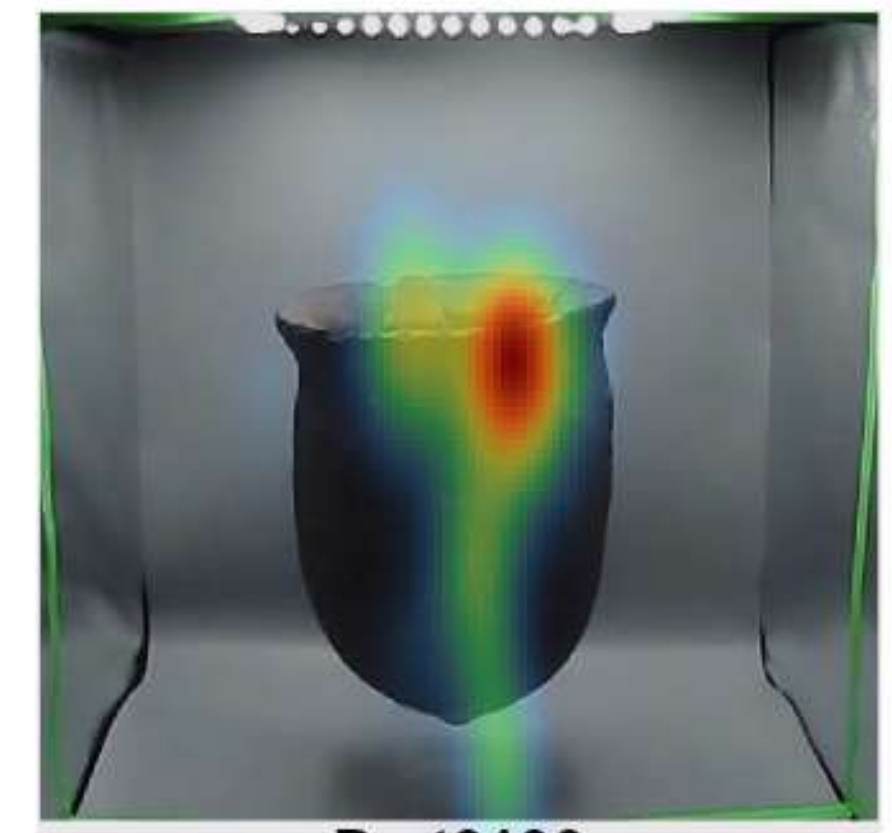
Part0100
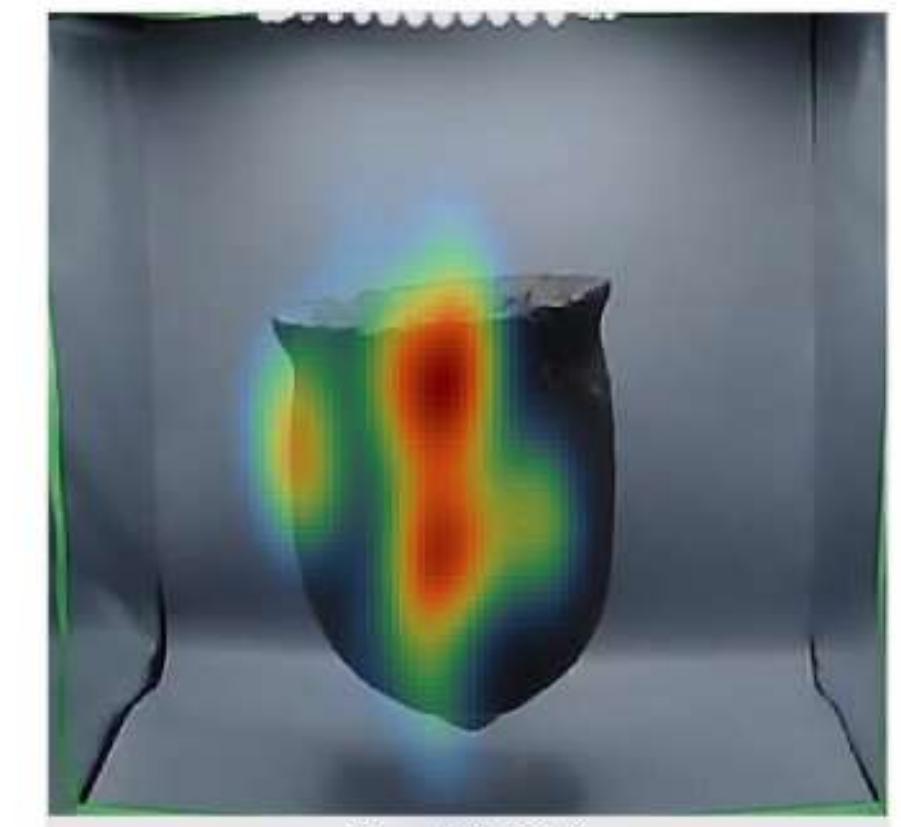
Part0101
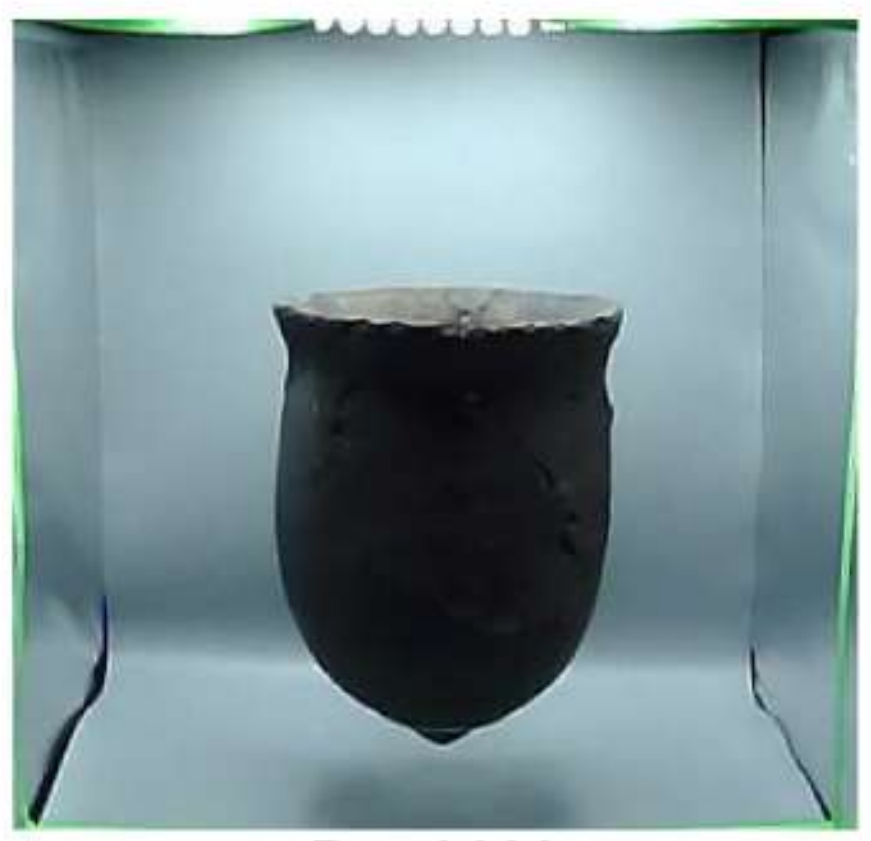
Part0102
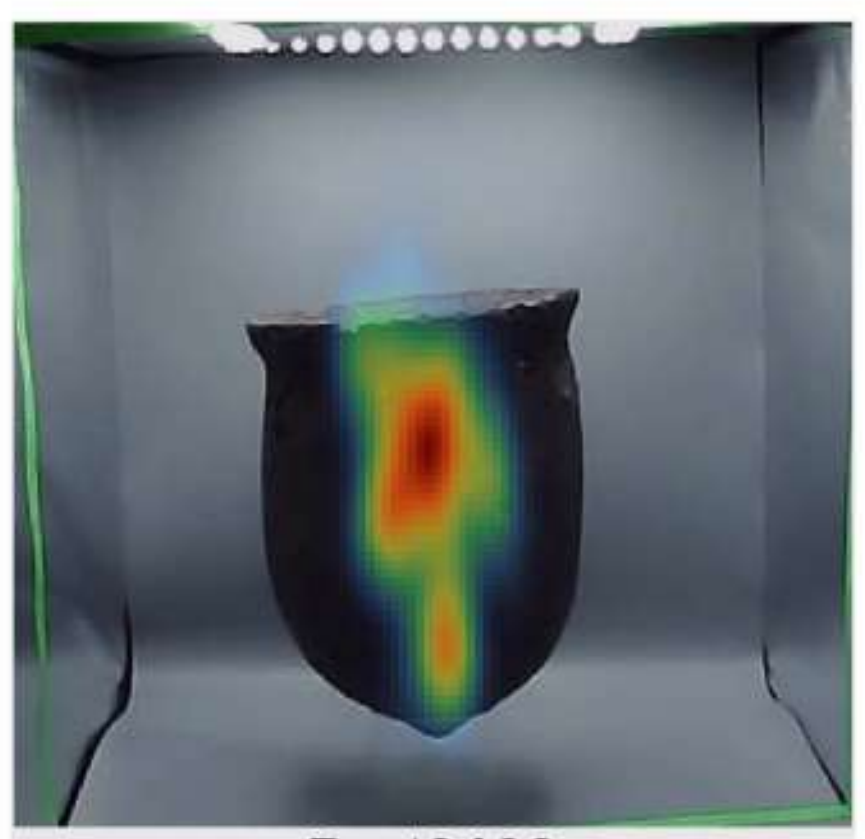
Part0103
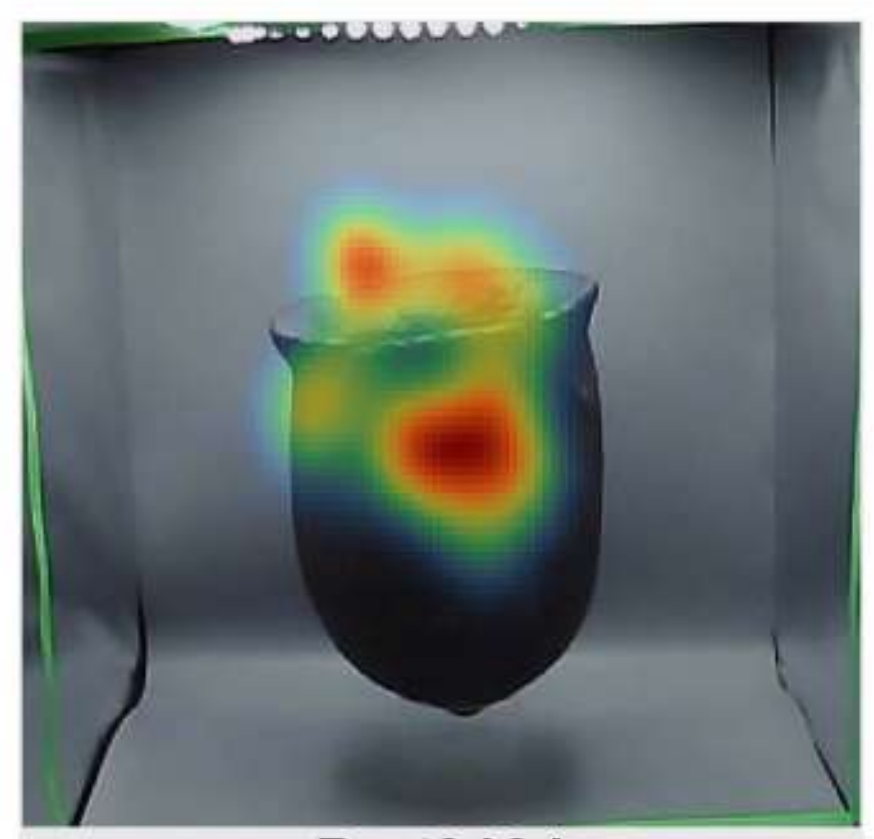
Part0104
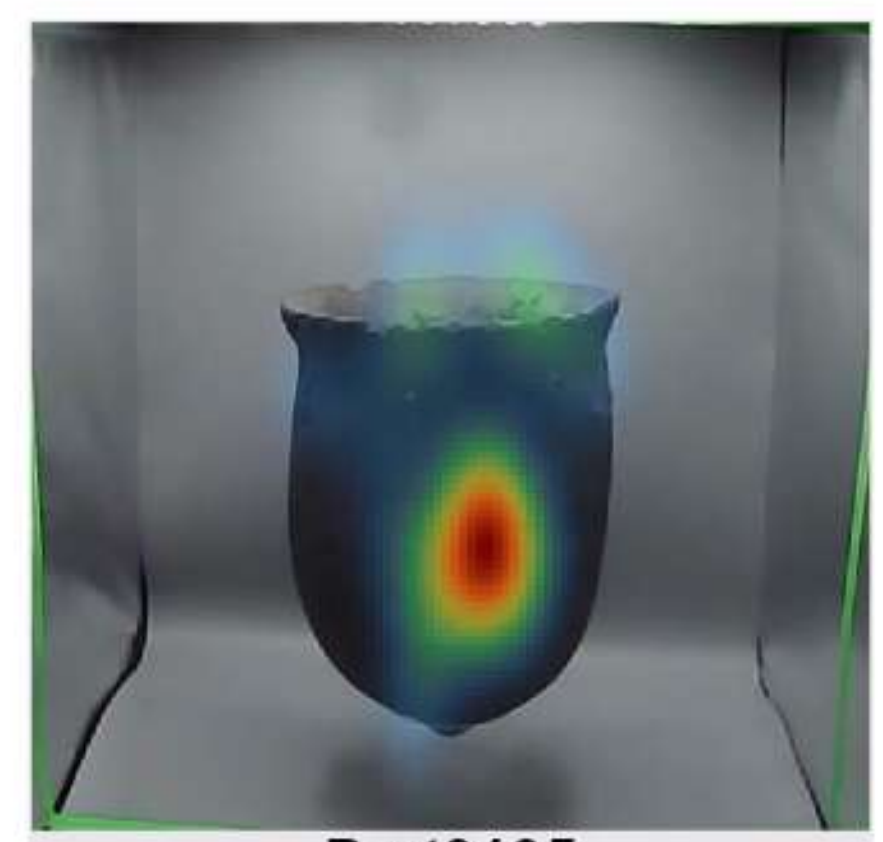
Part0105
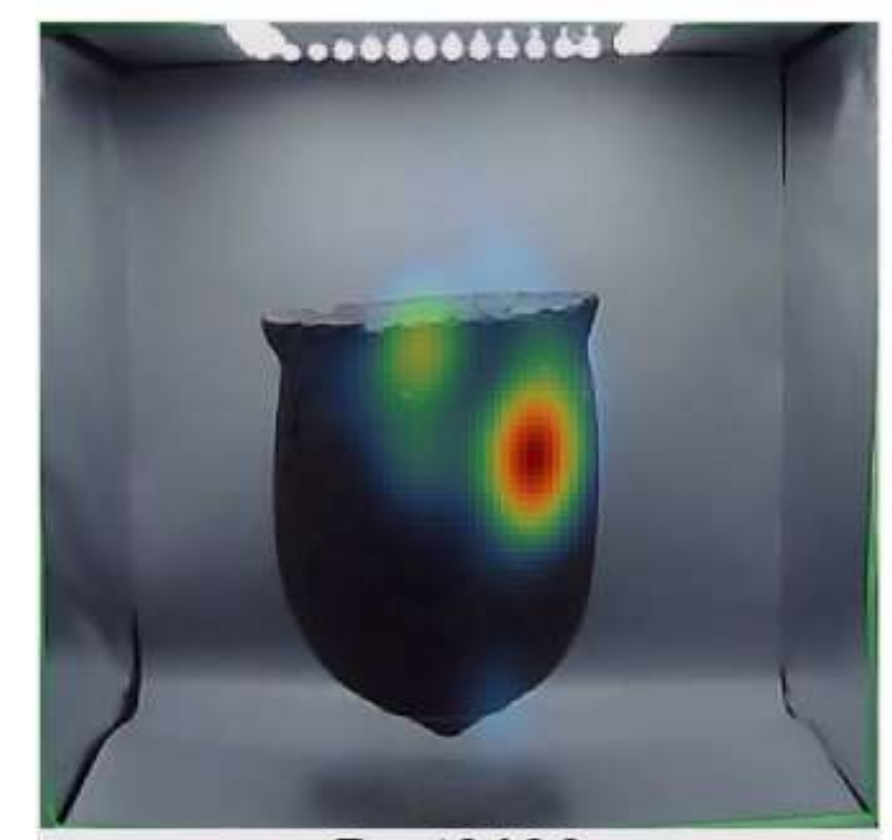
Part0106
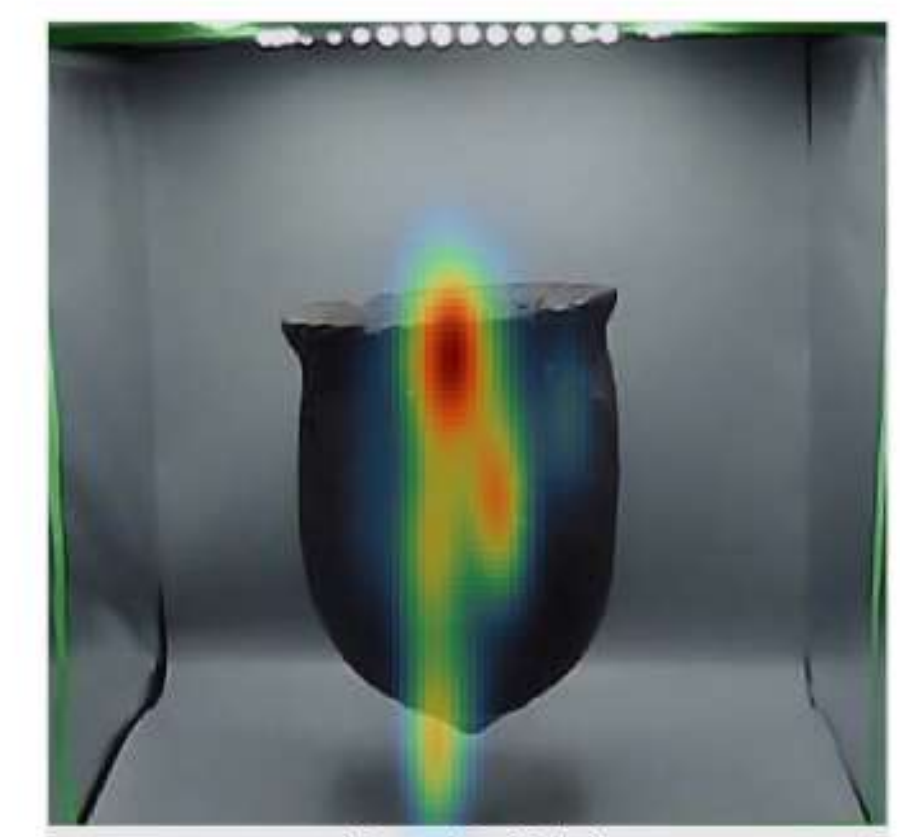
Part0107
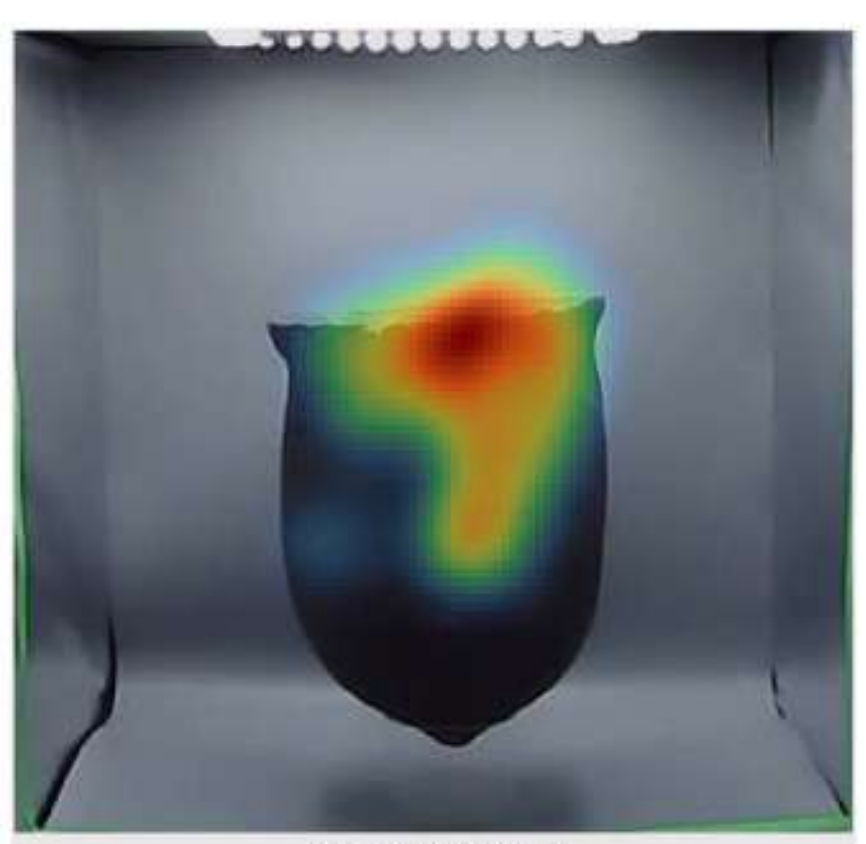
Part0108
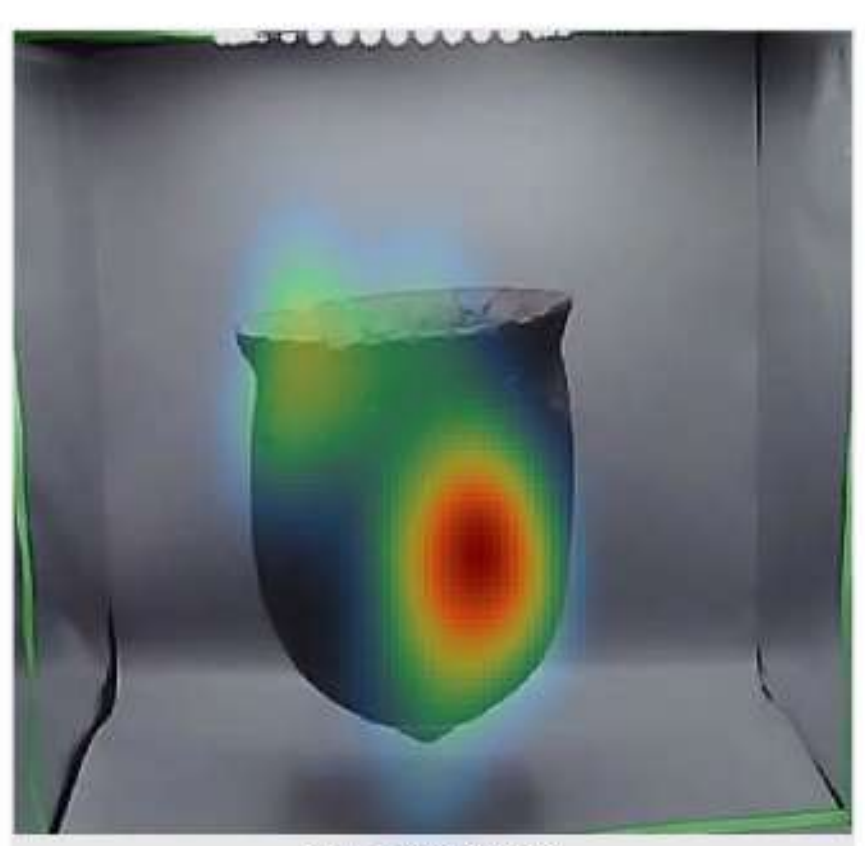
Part0109
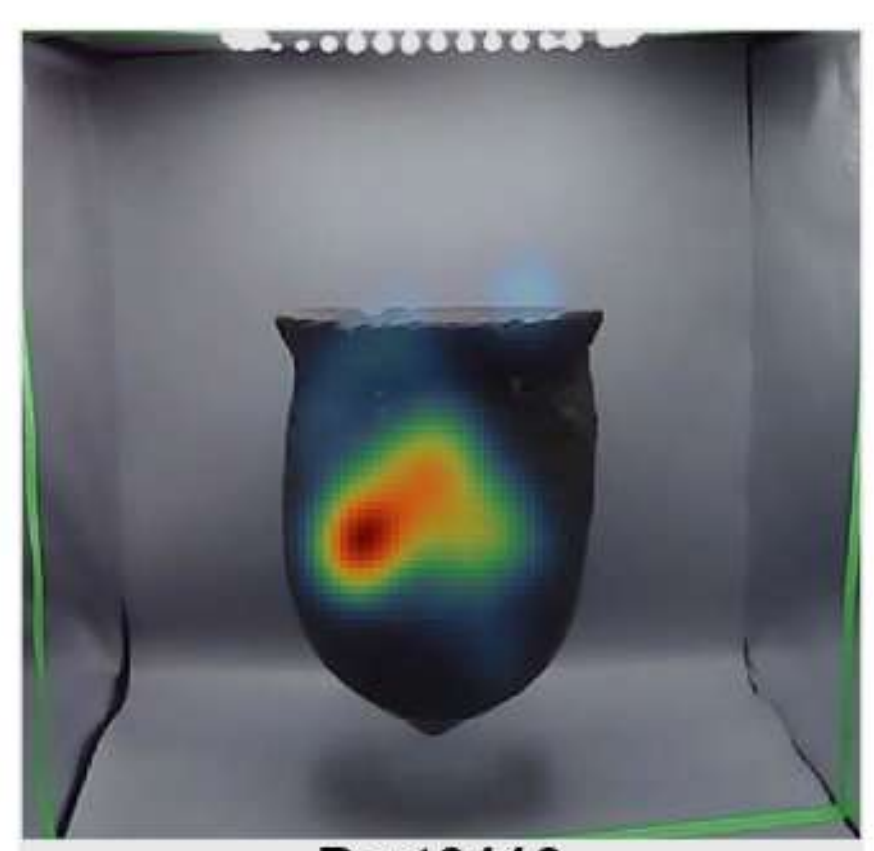
Part0110
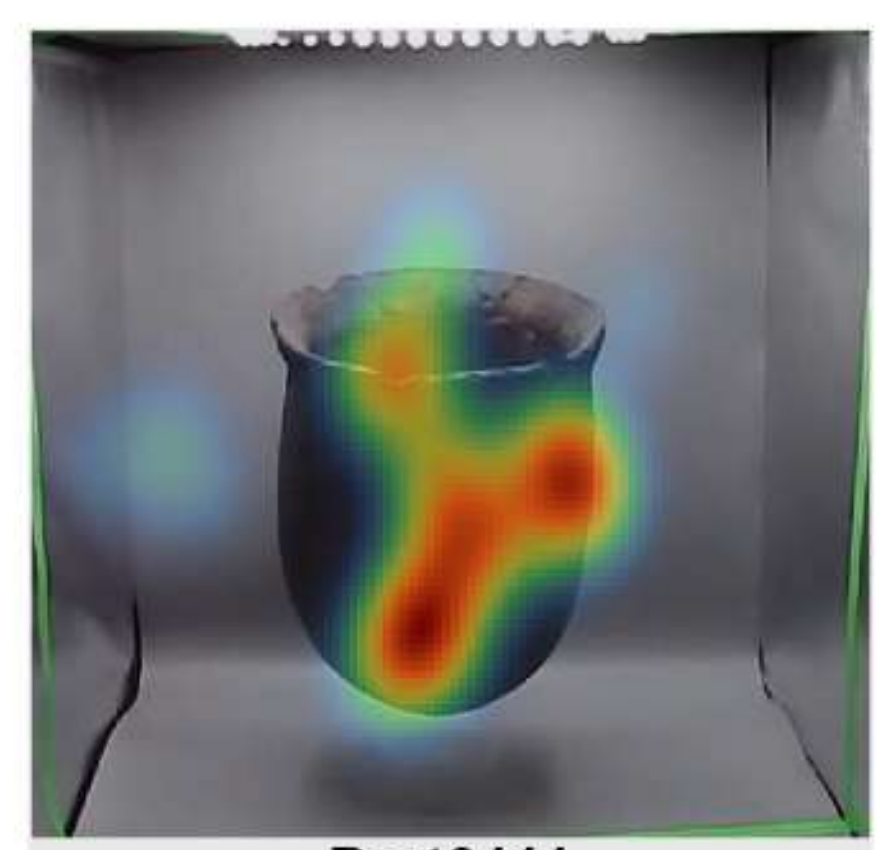
Part0111
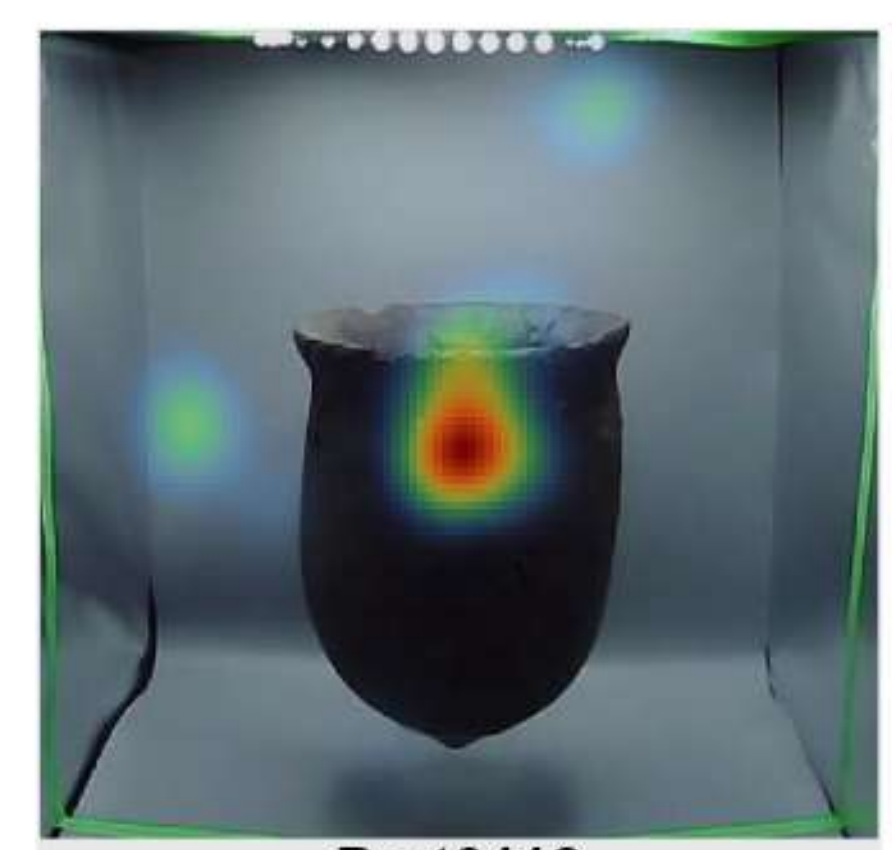
Part0112
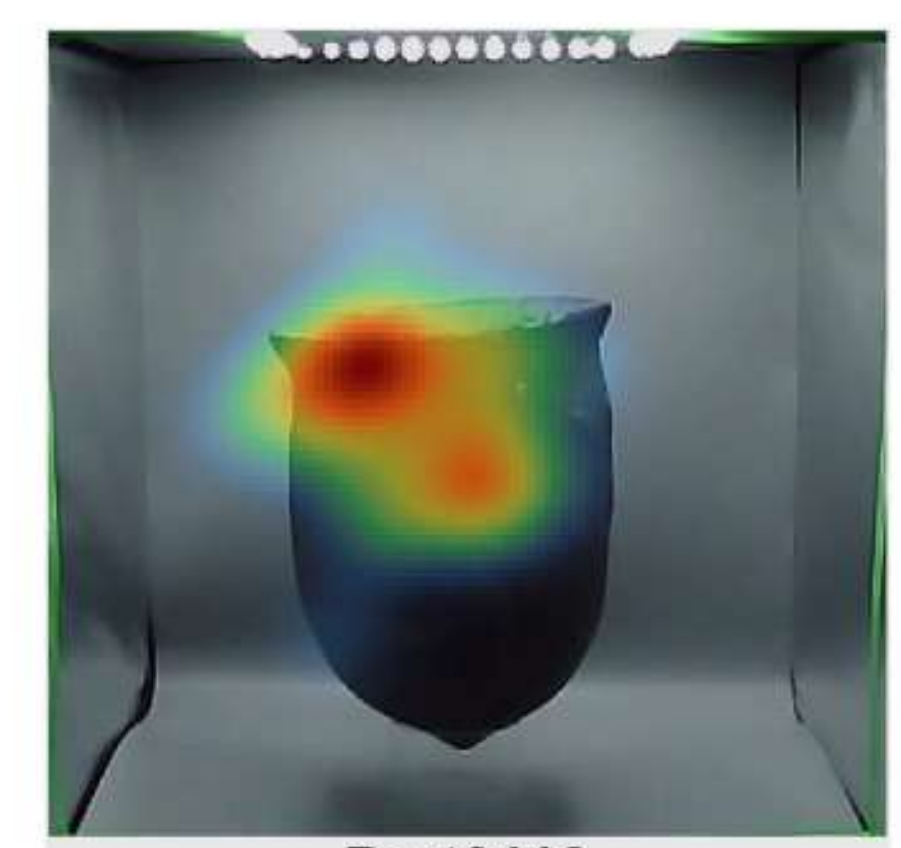
Part0113
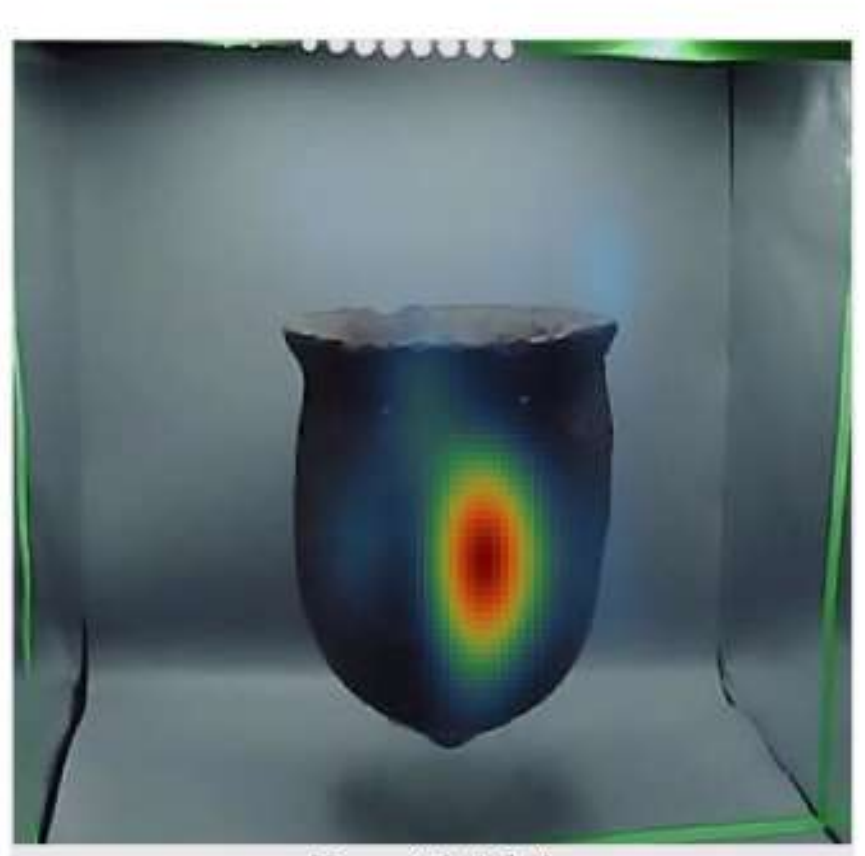
Part0114
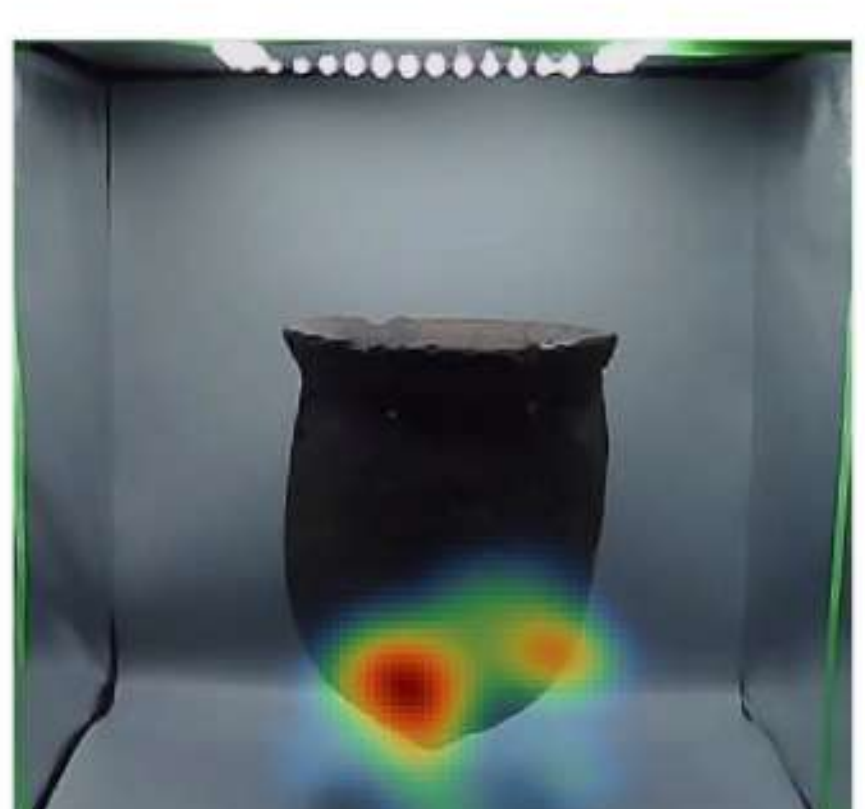
Part0115

Obj0236

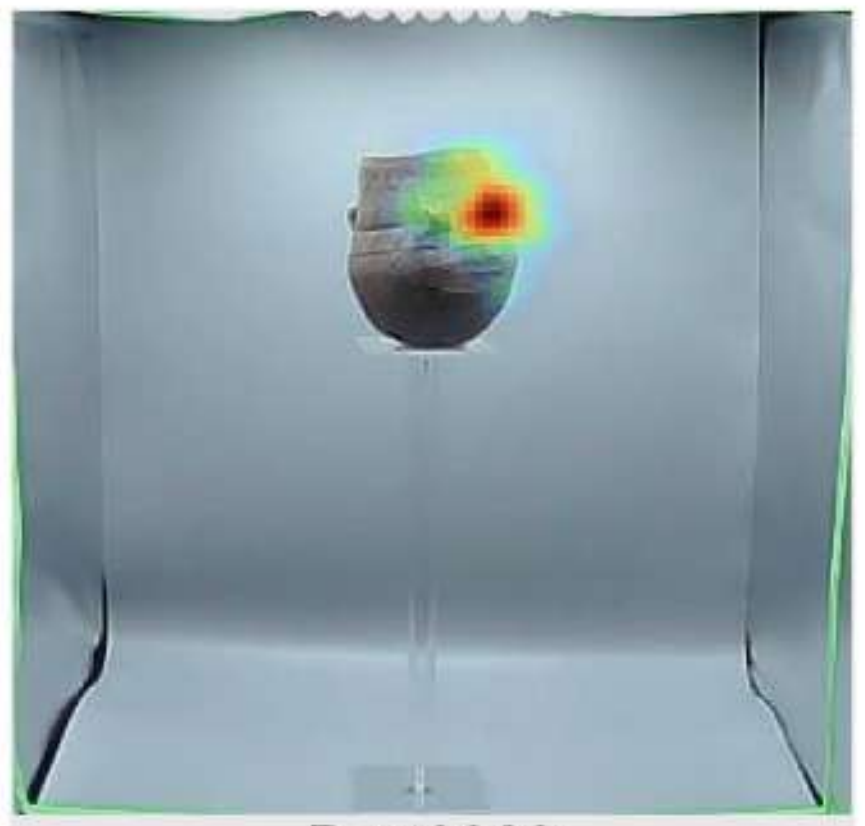
Part0089

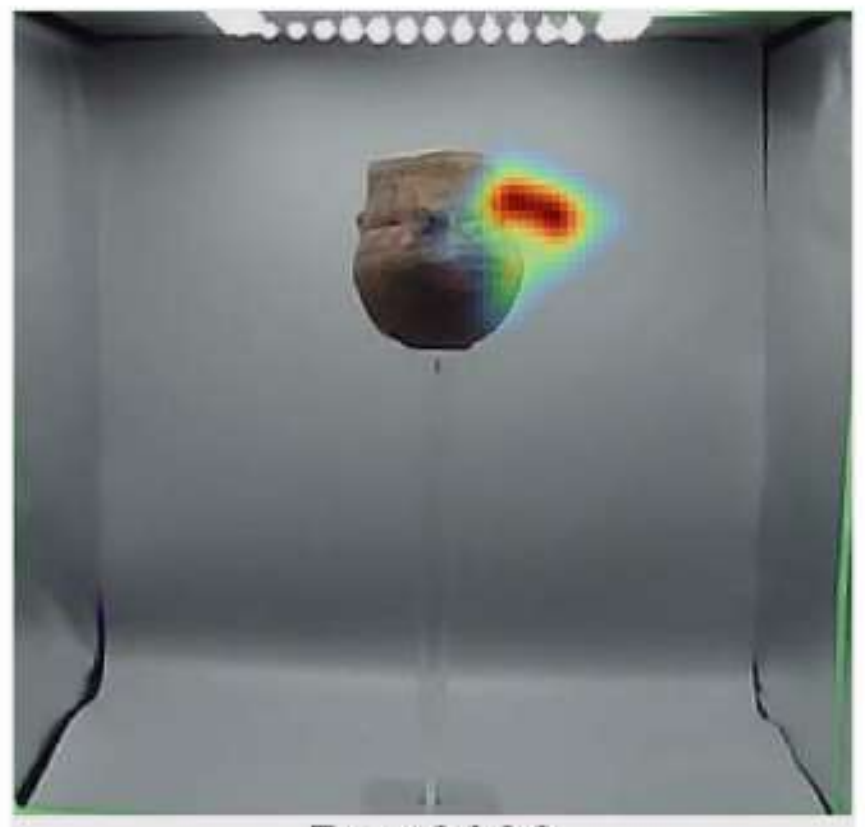
Part0090

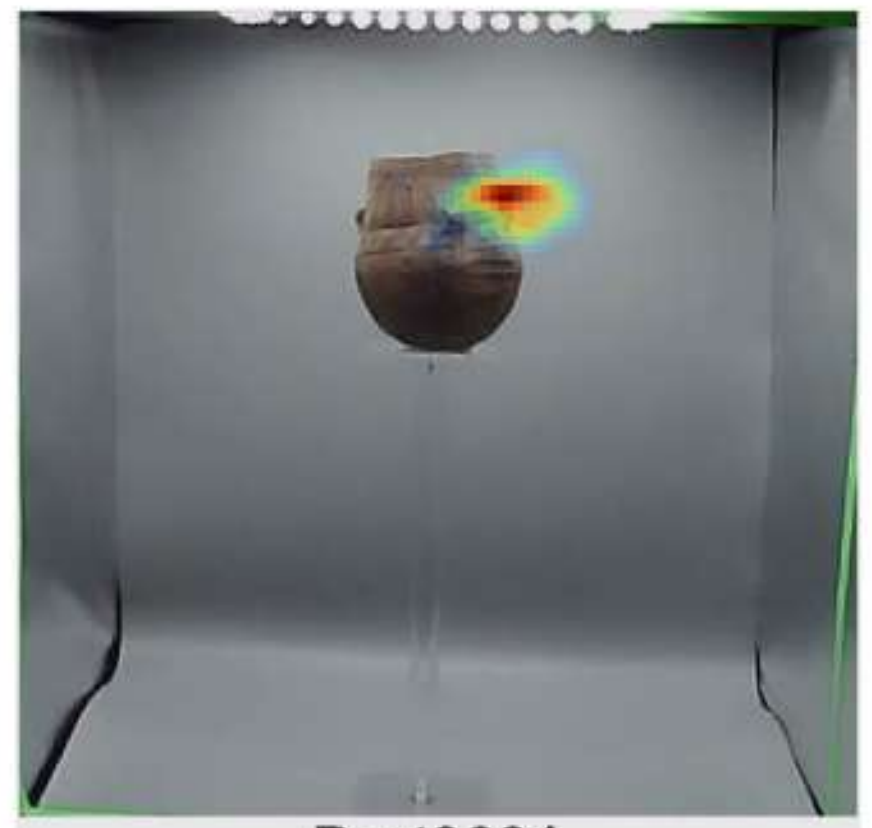
Part0091

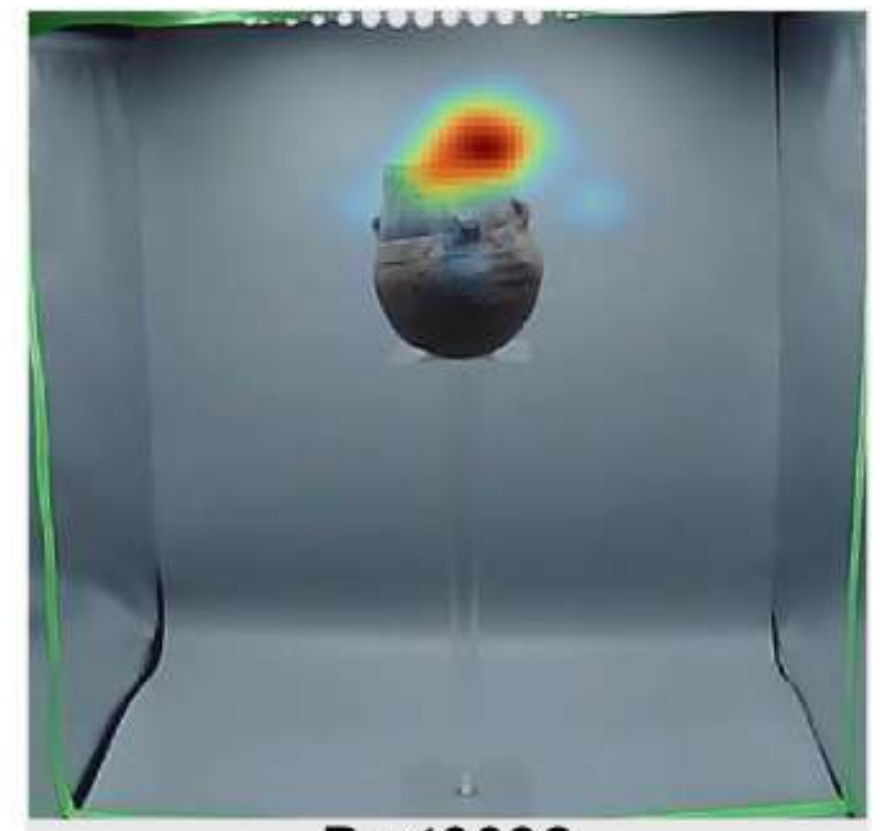
Part0092

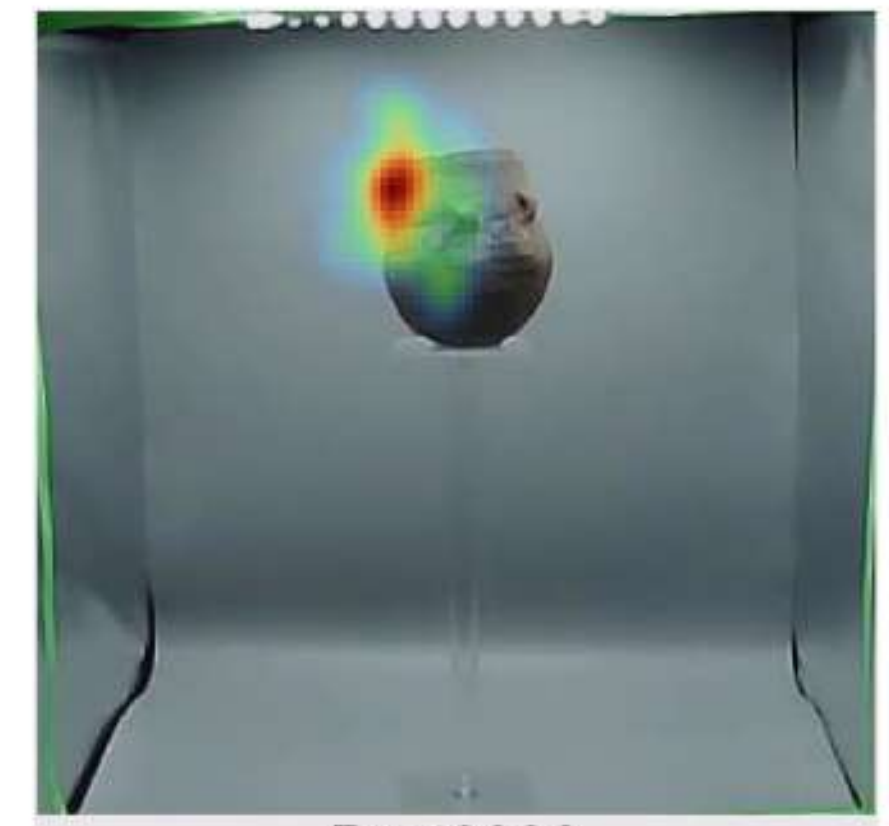
Part0093

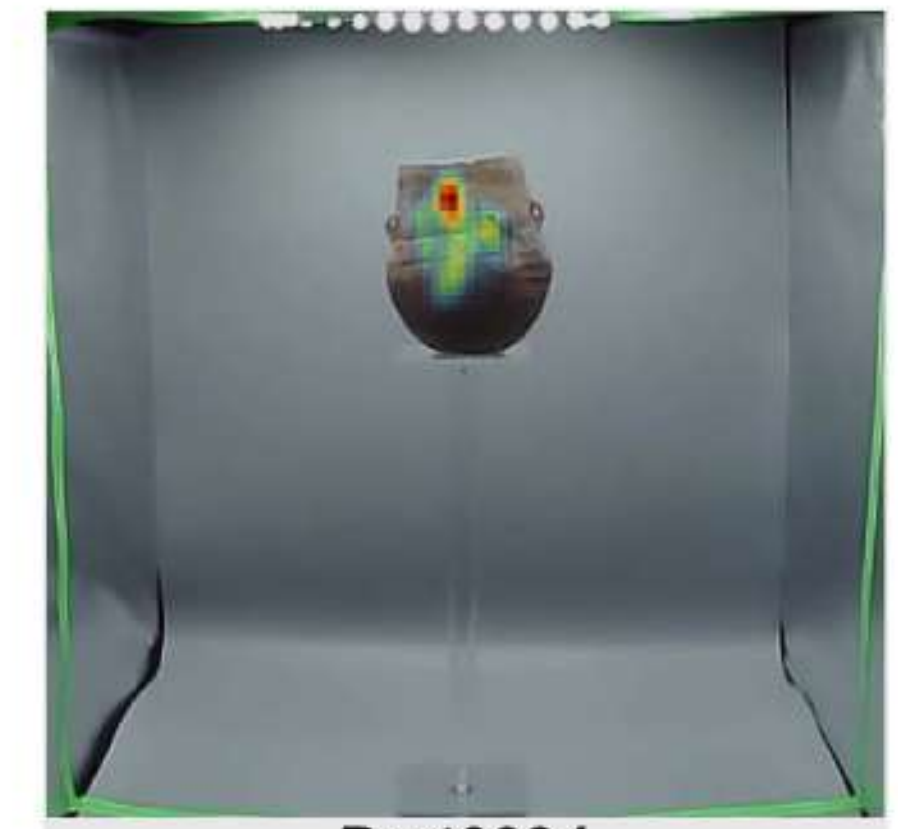
Part0094

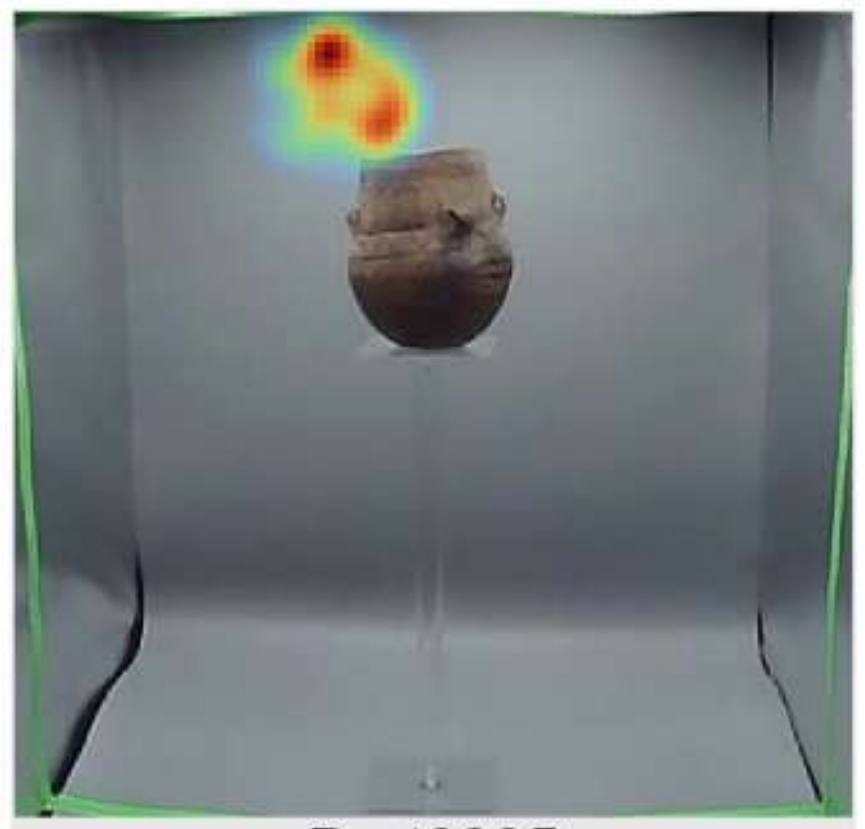
Part0095

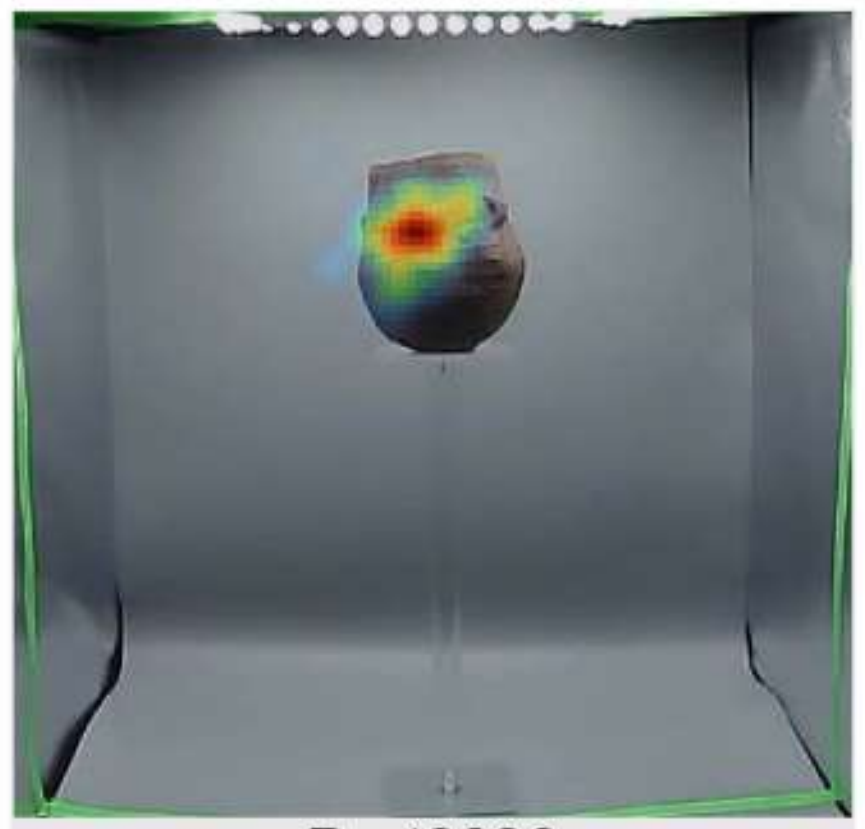
Part0096

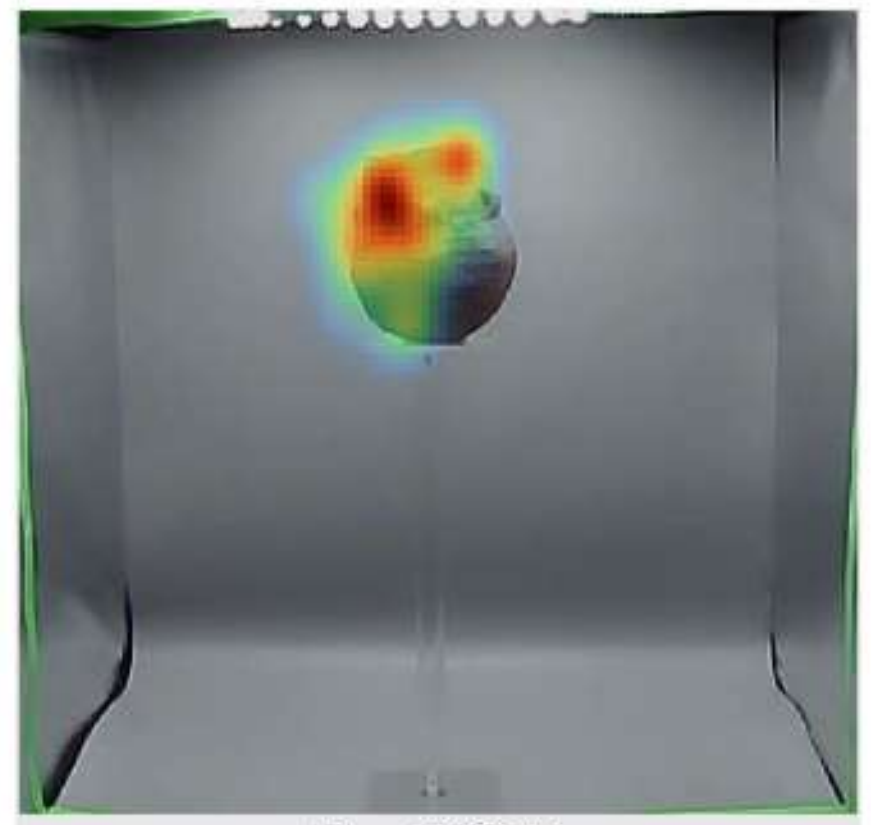
Part0097

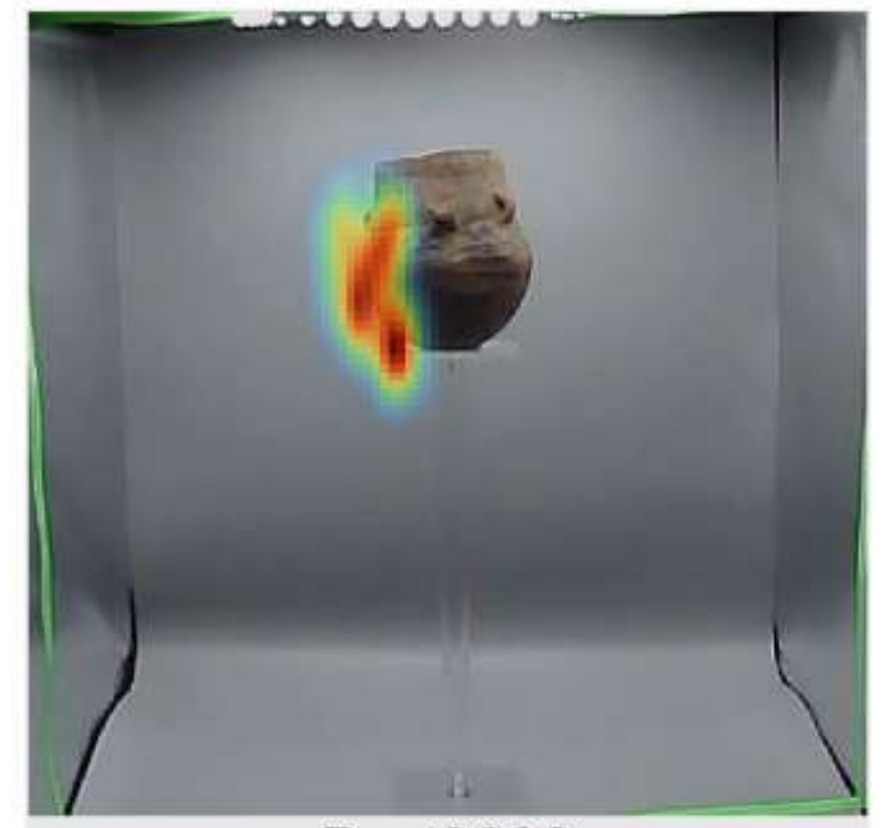
Part0098

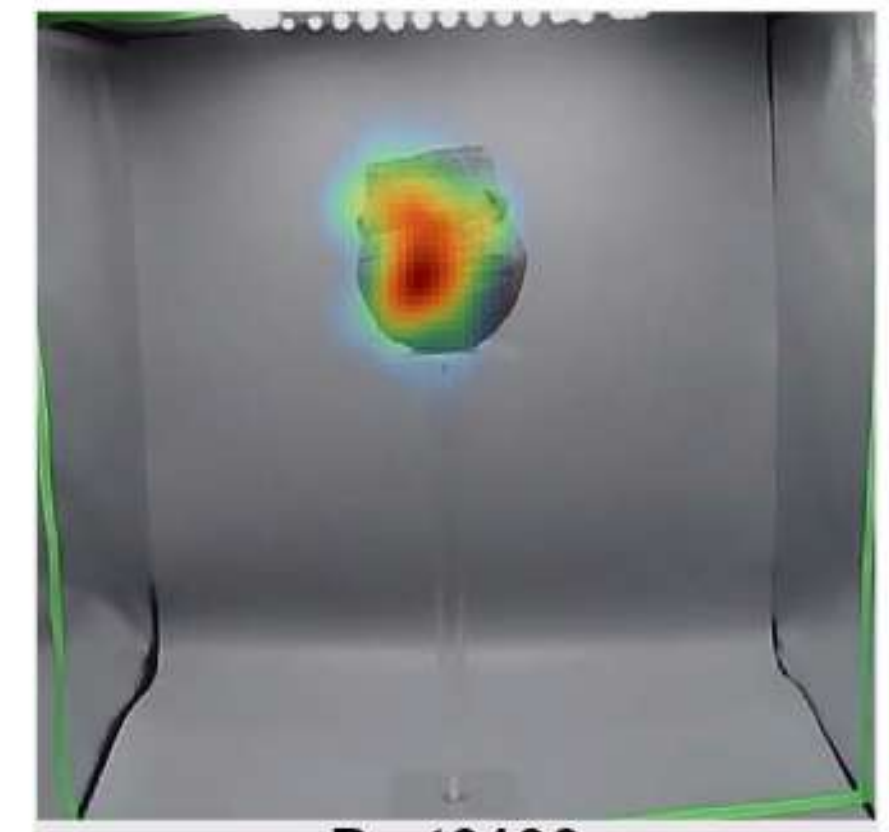
Part0100

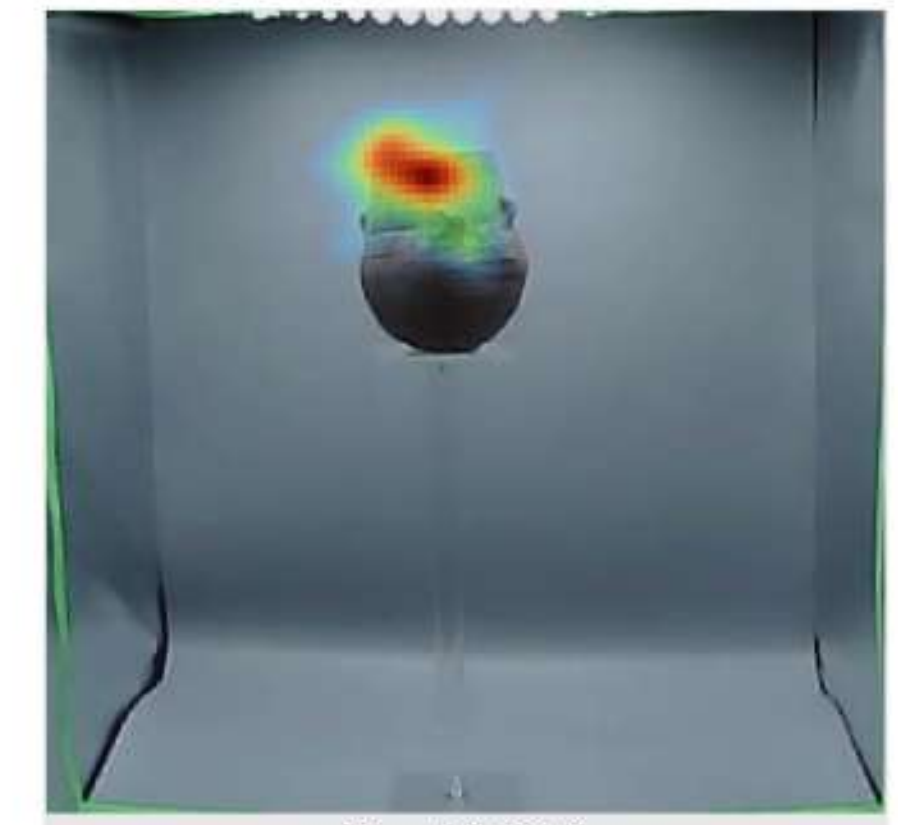
Part0101

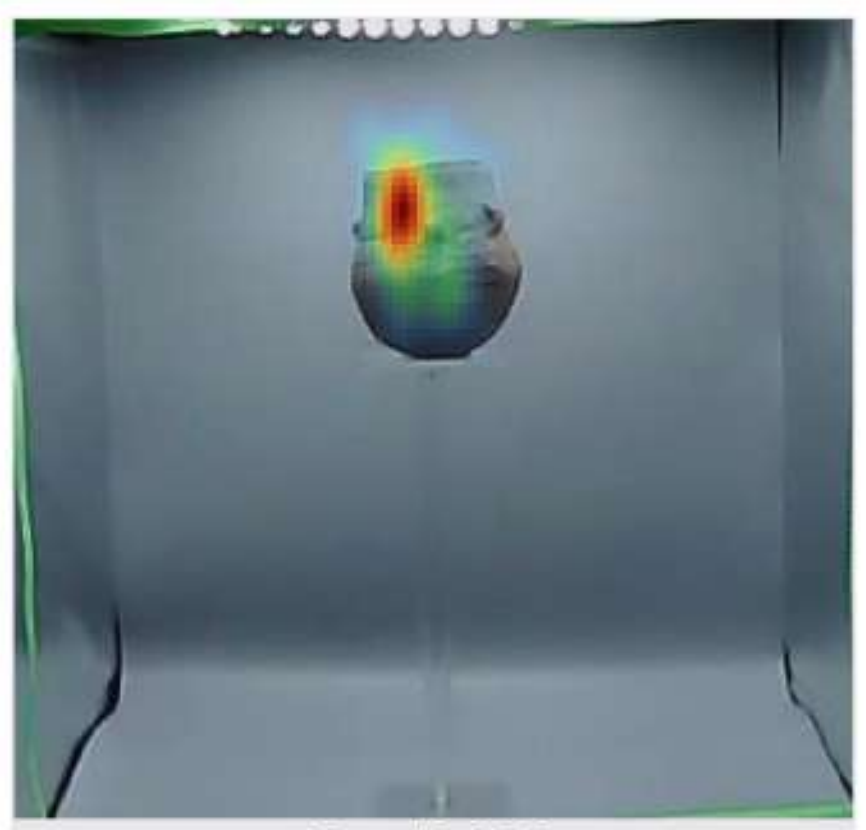
Part0102

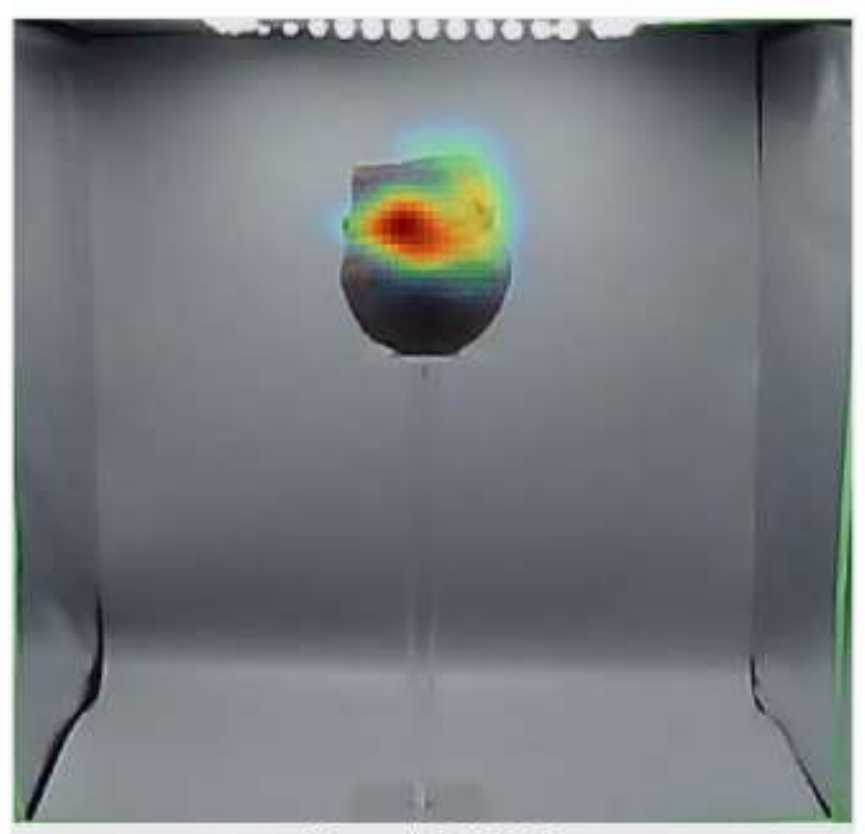
Part0103

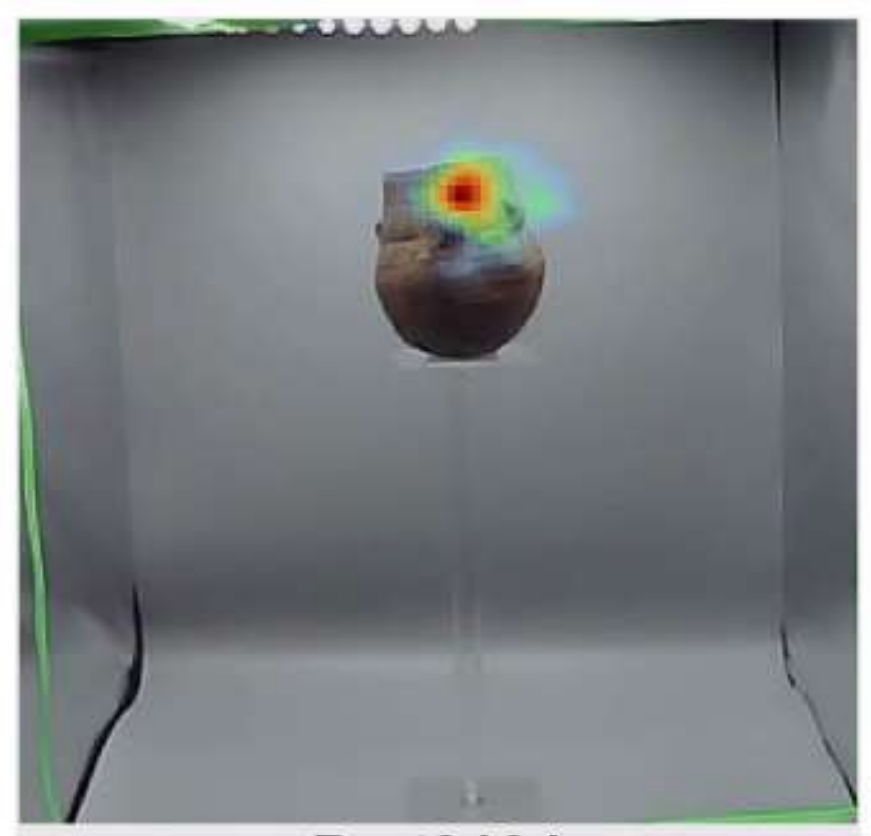
Part0104

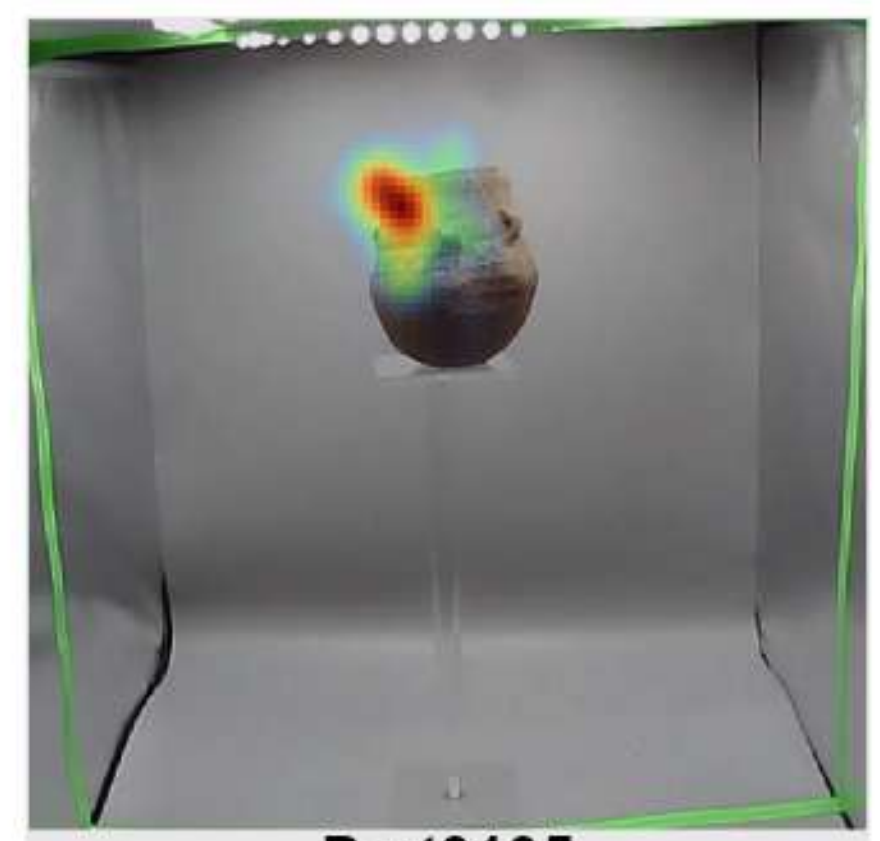
Part0105

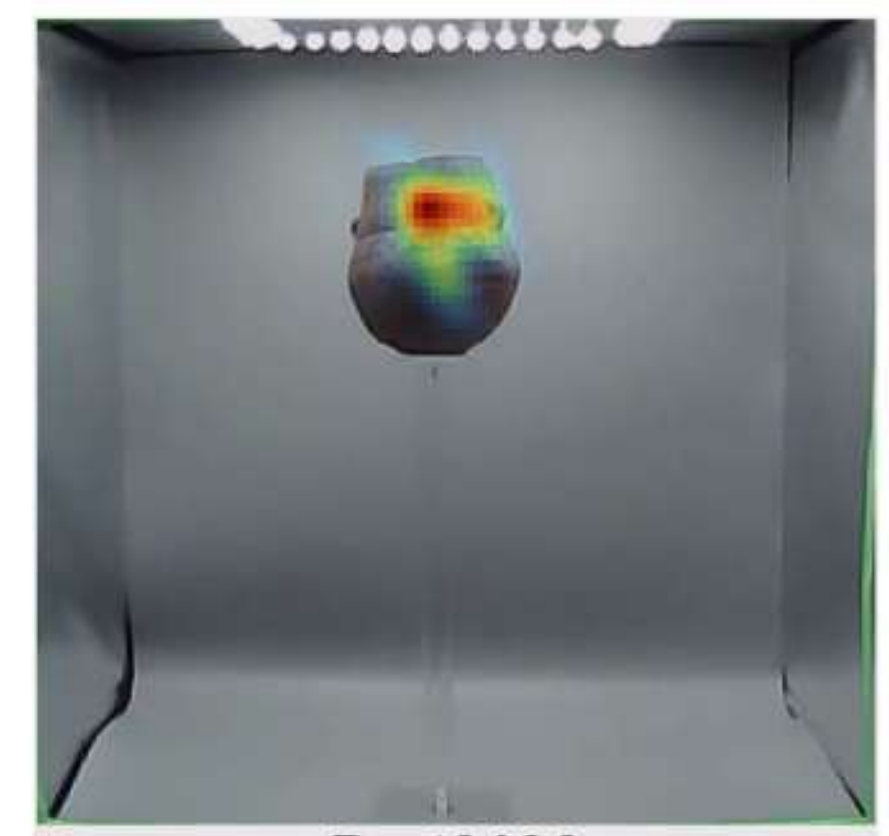
Part0106

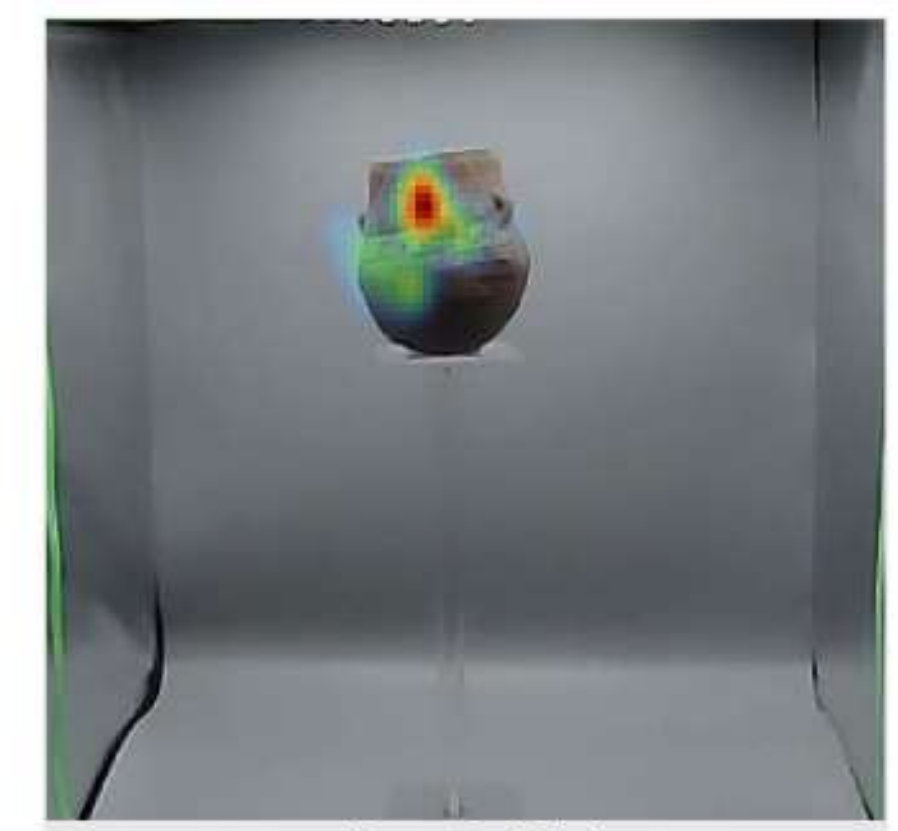
Part0107

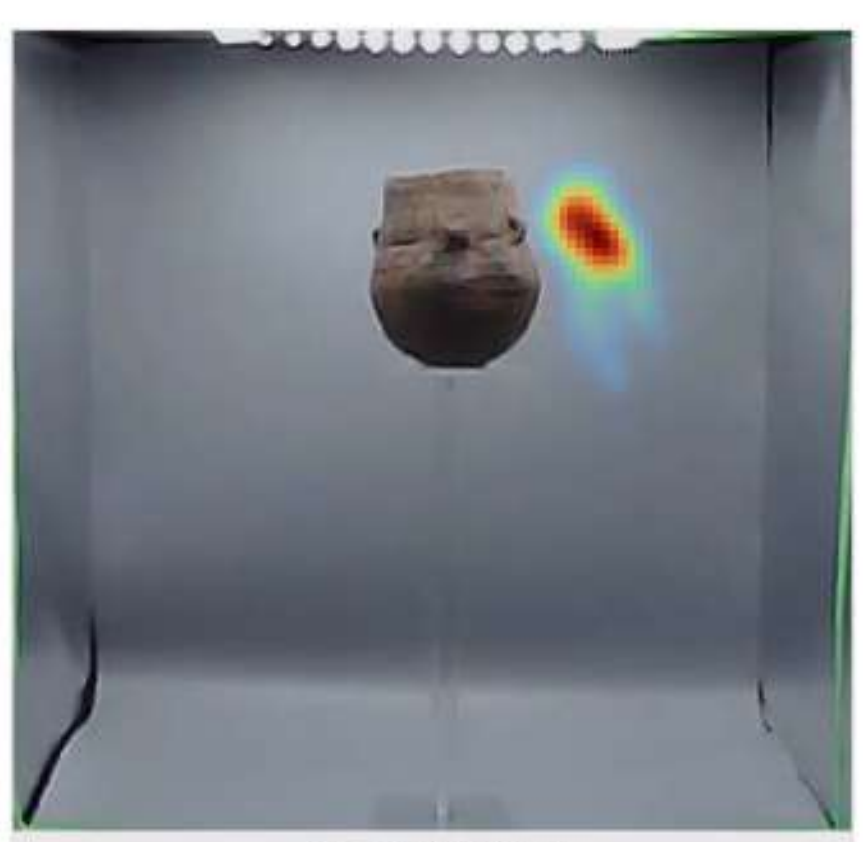
Part0108

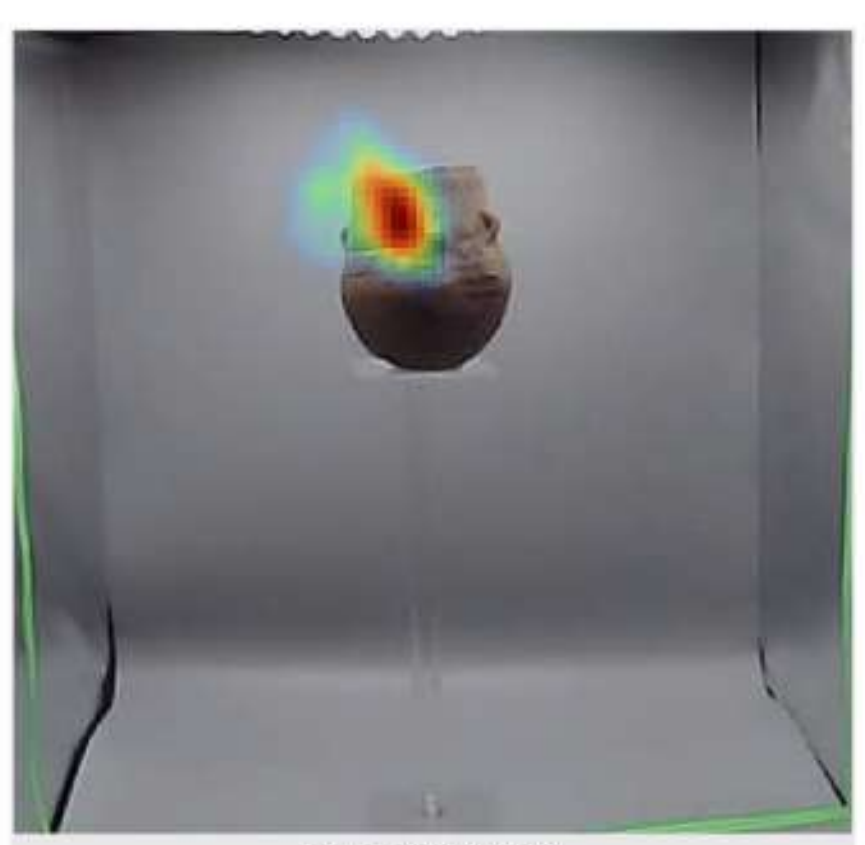
Part0109

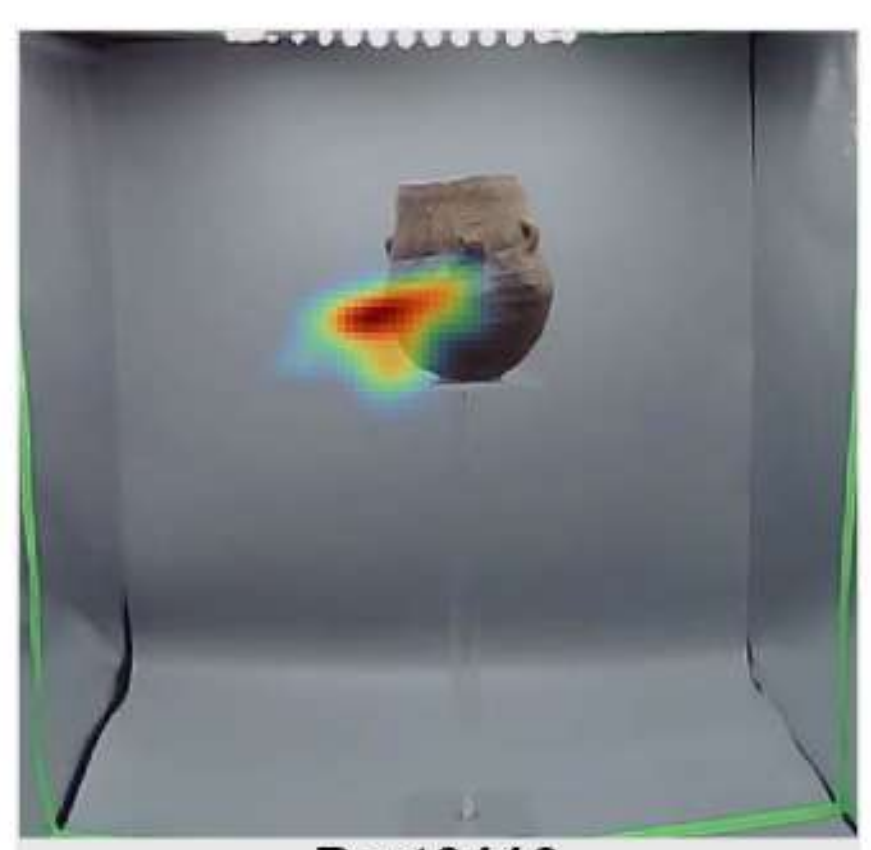
Part0110

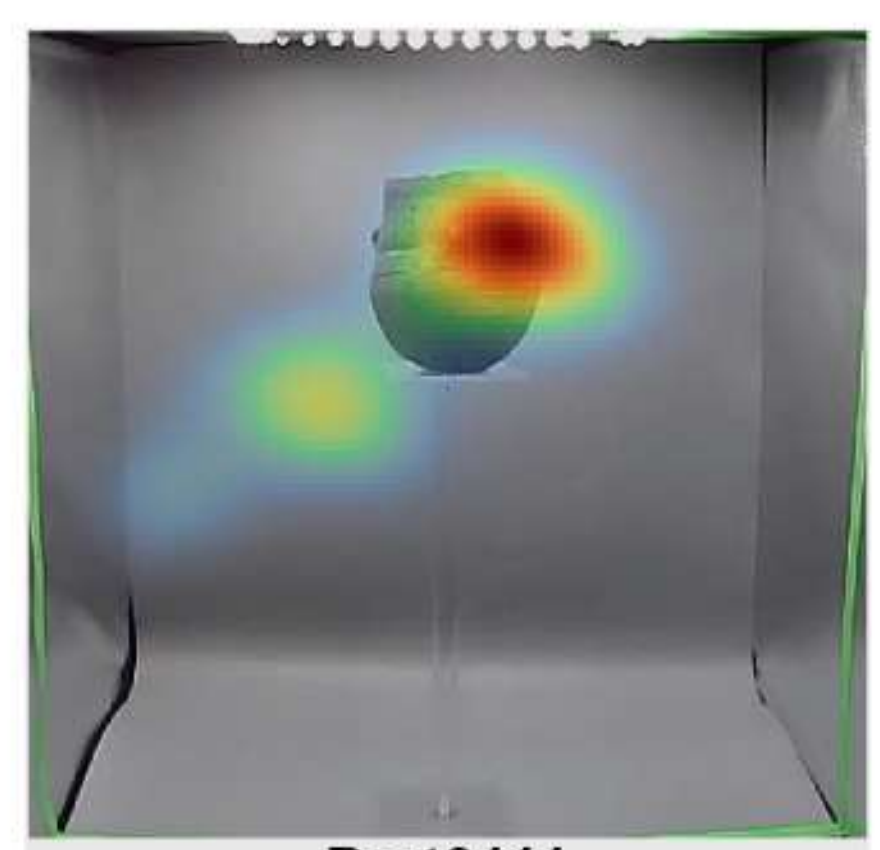
Part0111

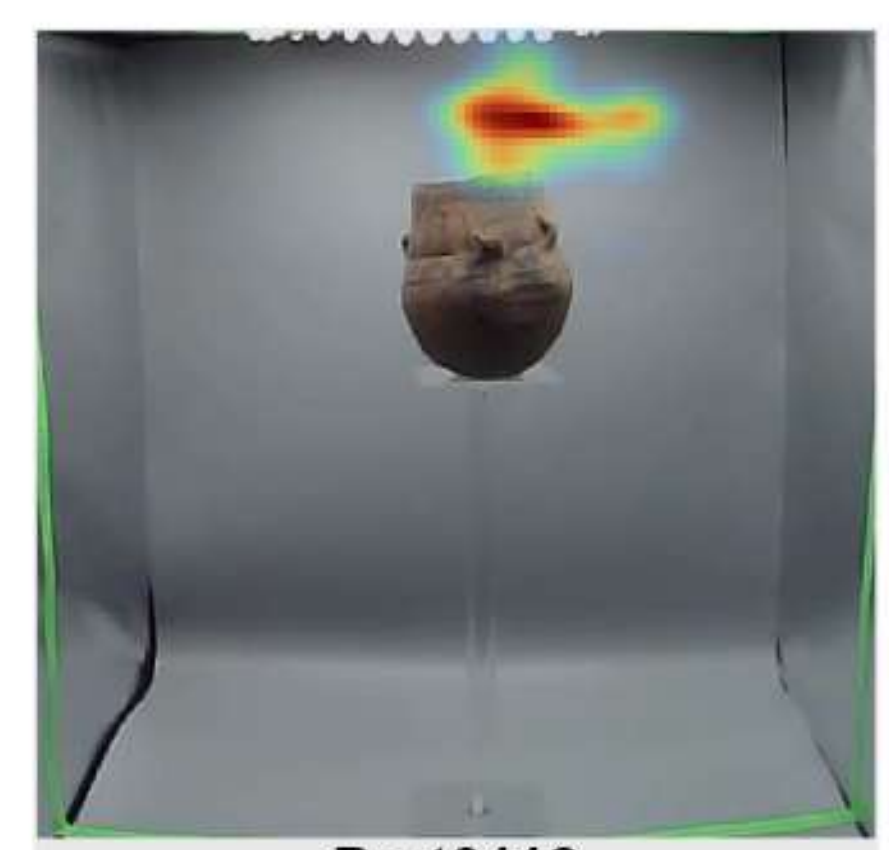
Part0112

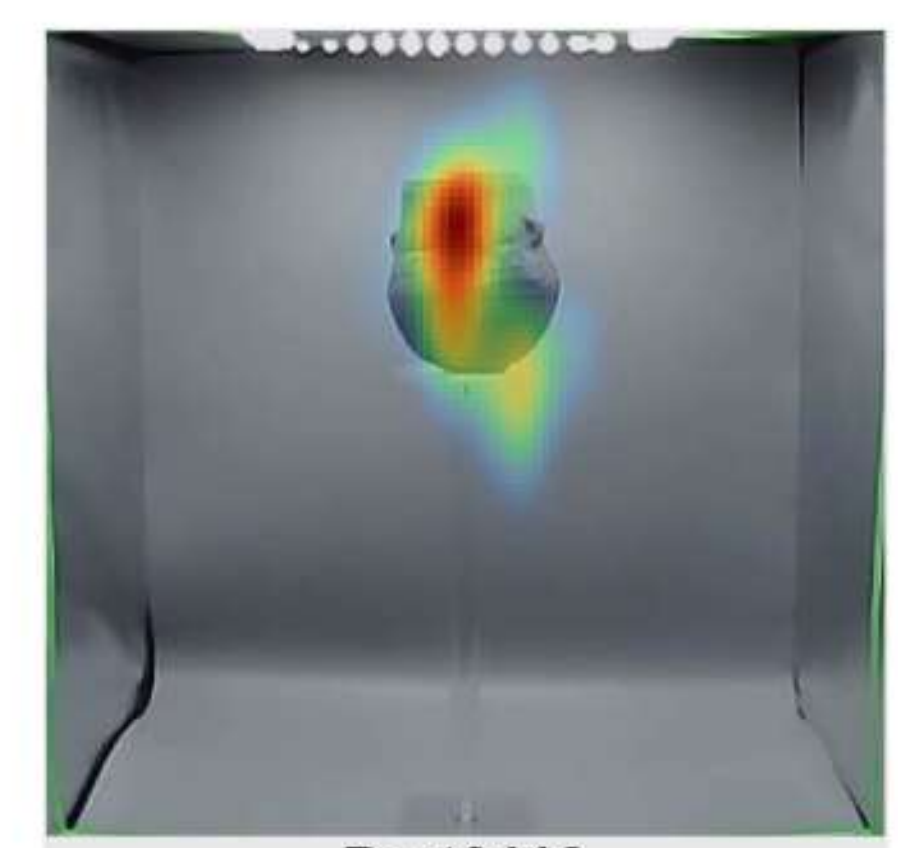
Part0113

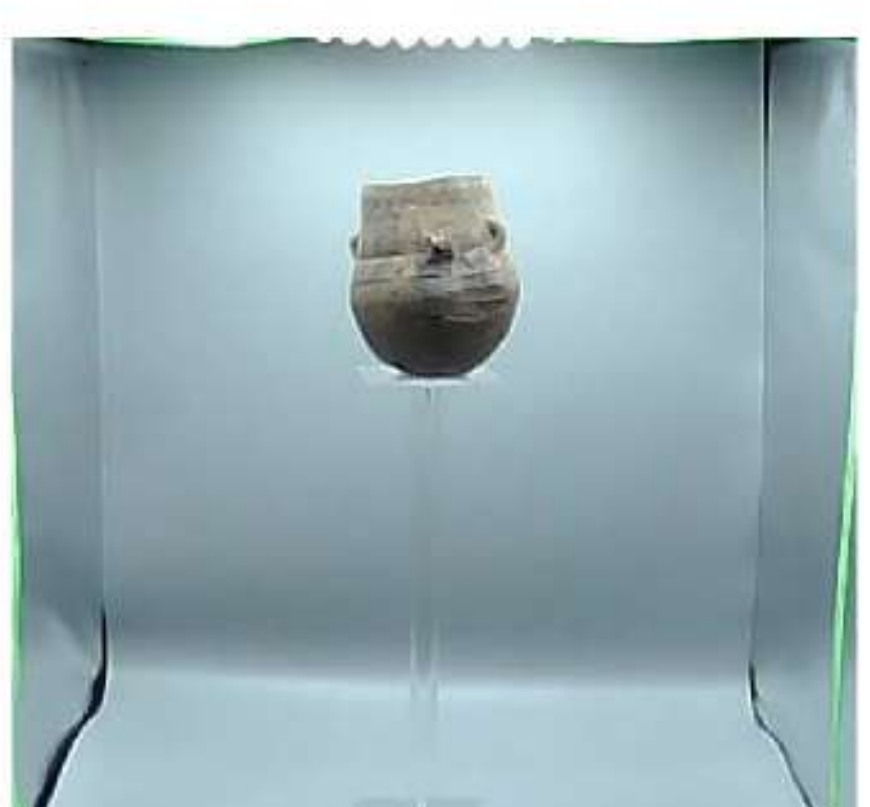
Part0114

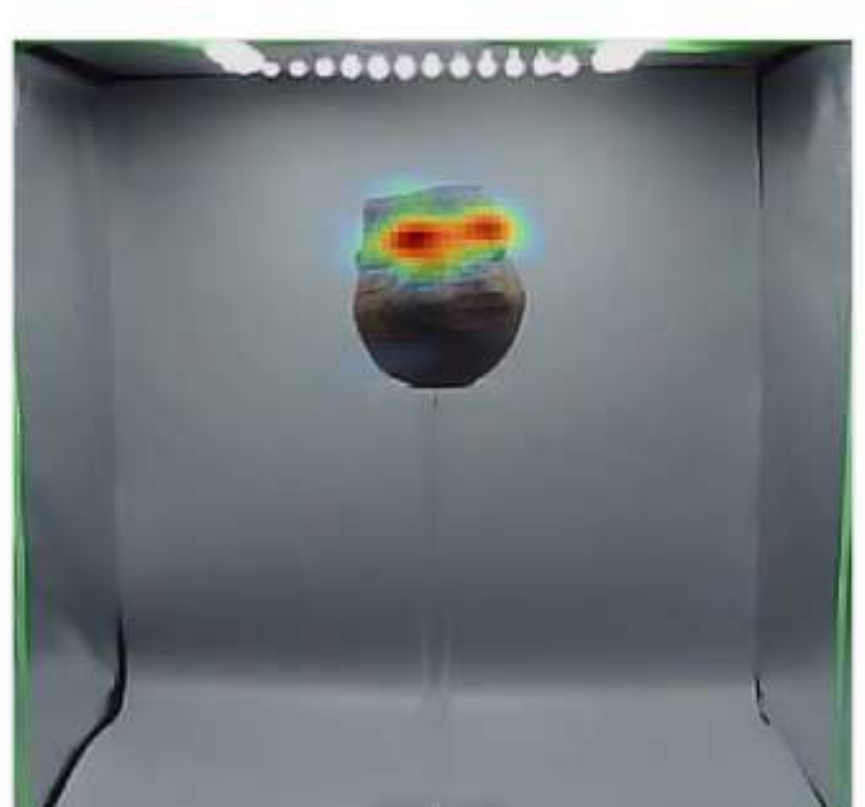
Part0115

Obj0238

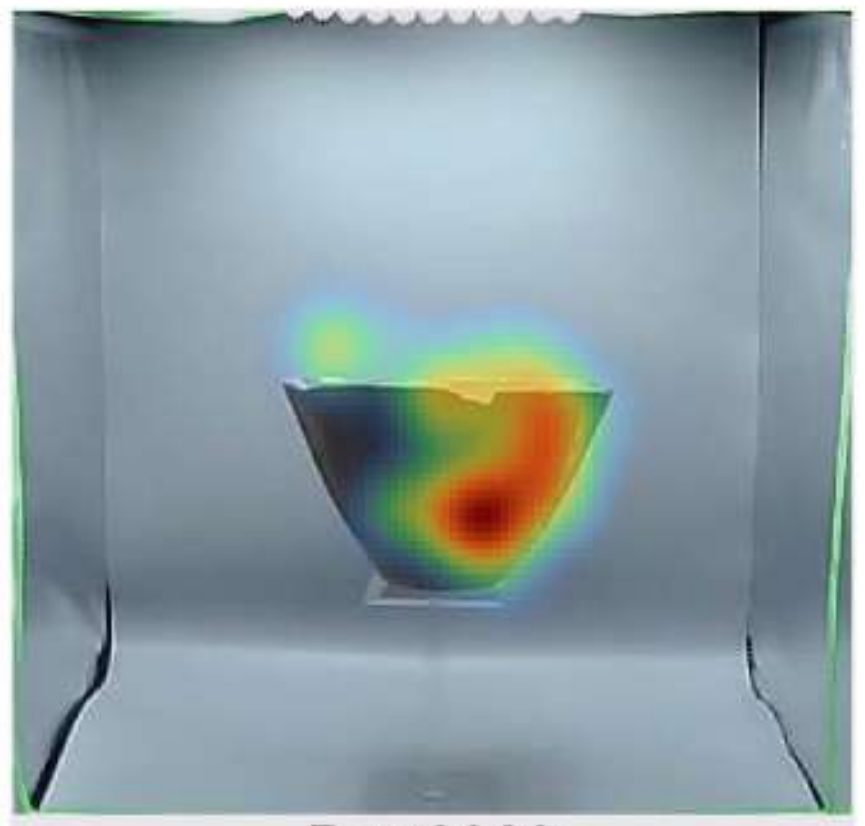
Part0089

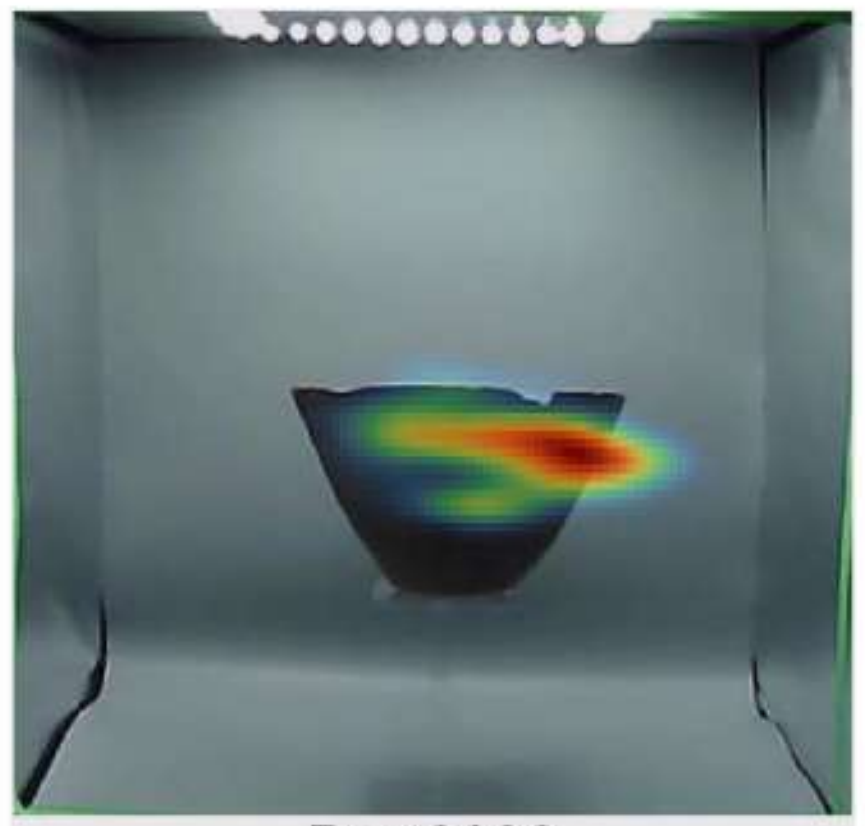
Part0090

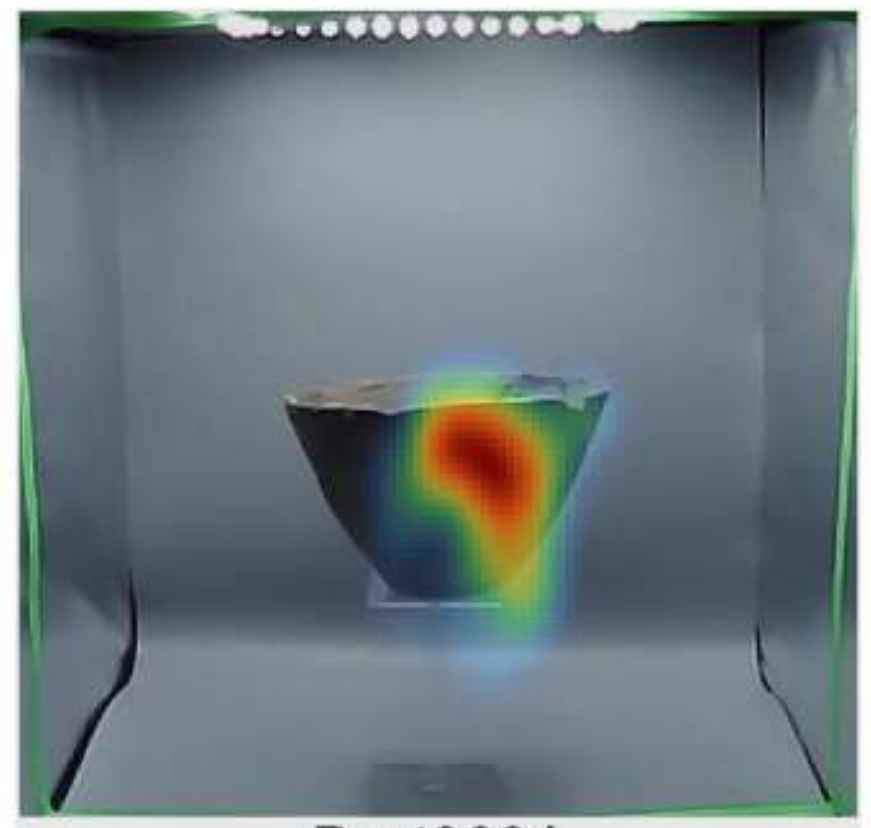
Part0091

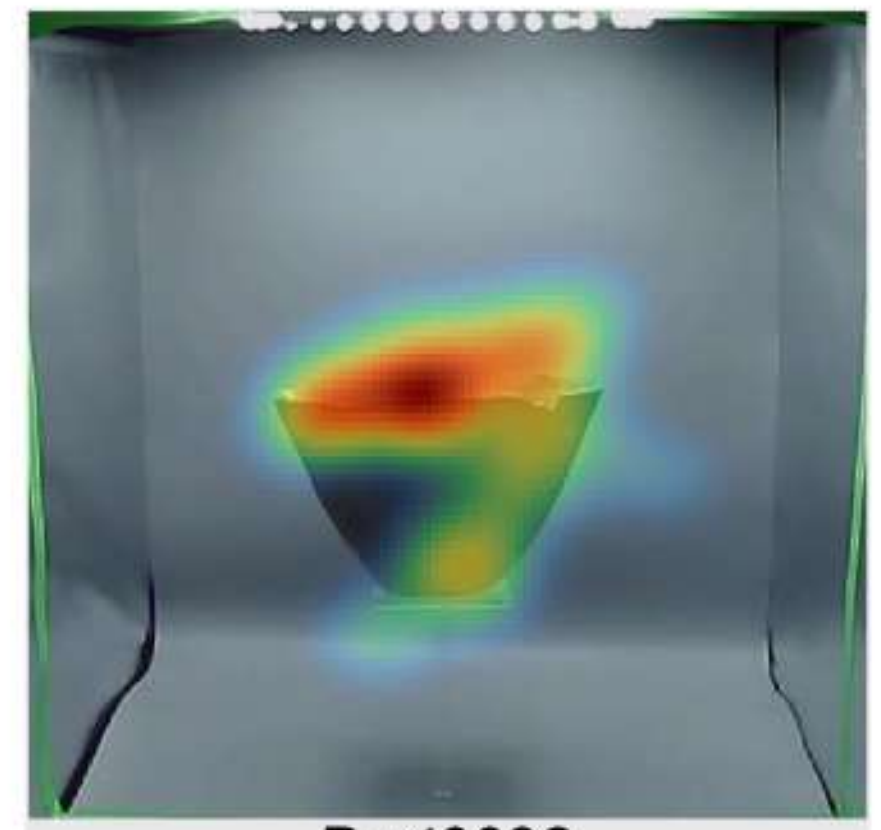
Part0092

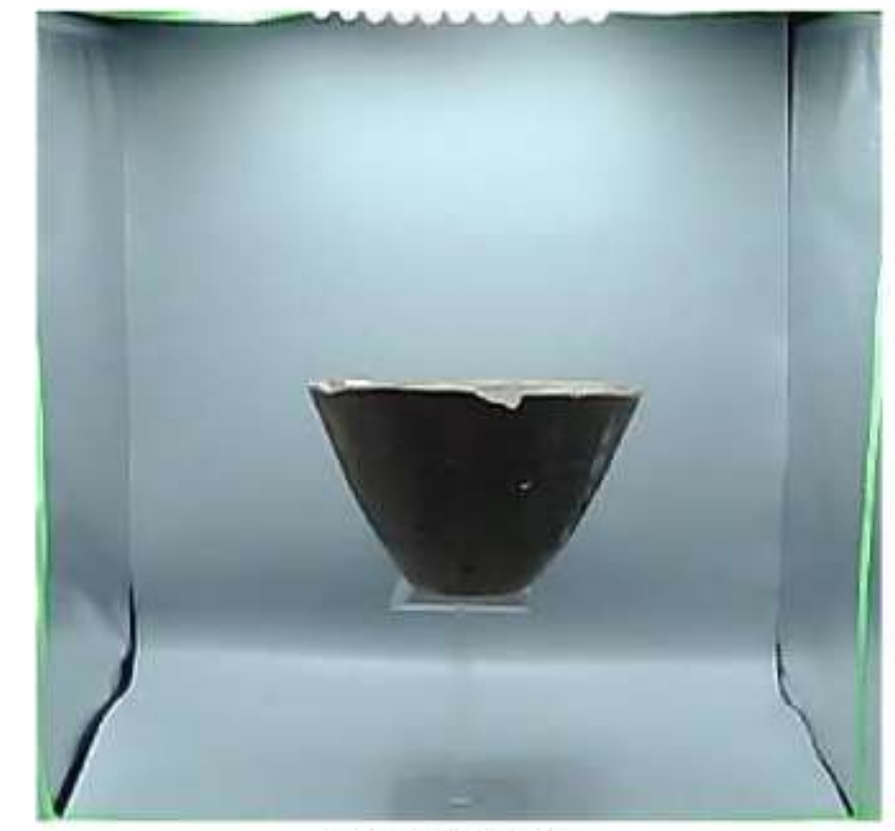
Part0093

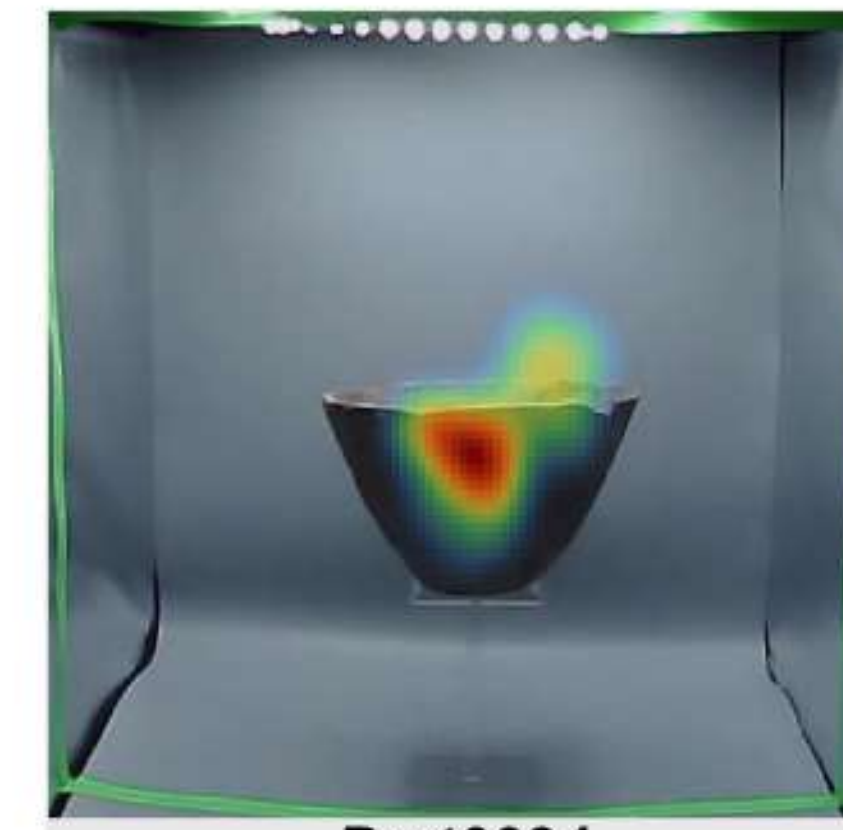
Part0094

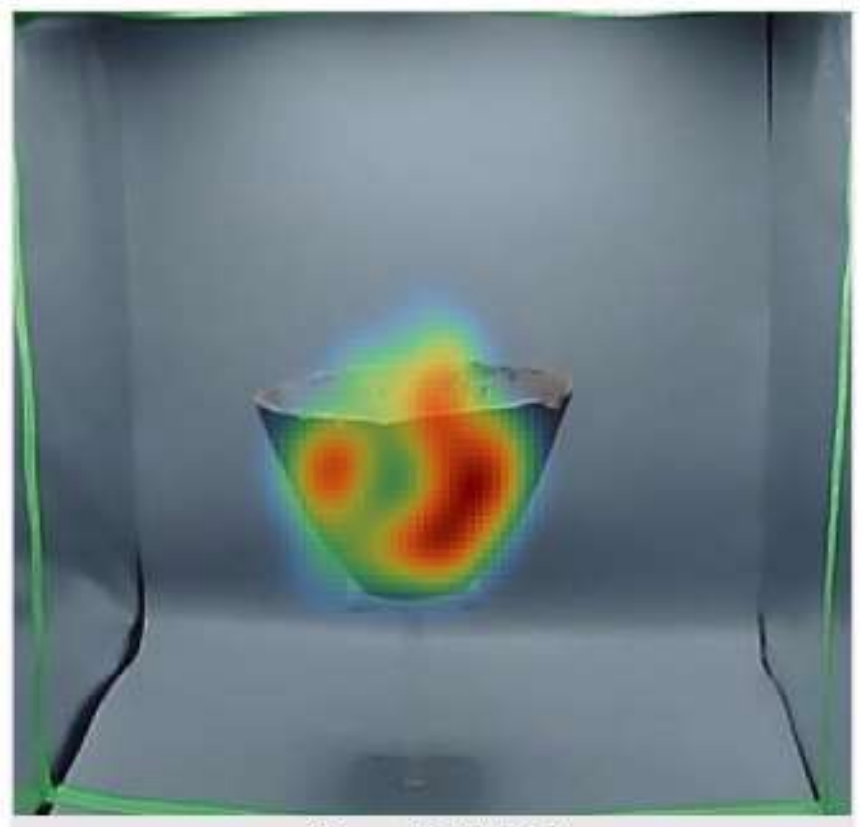
Part0095

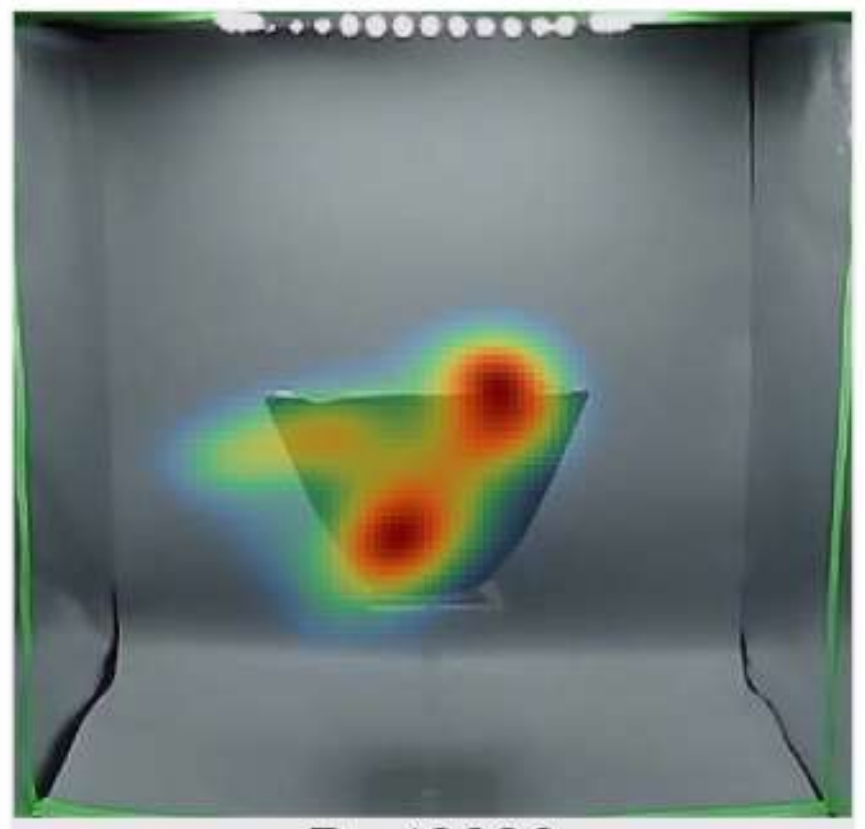
Part0096

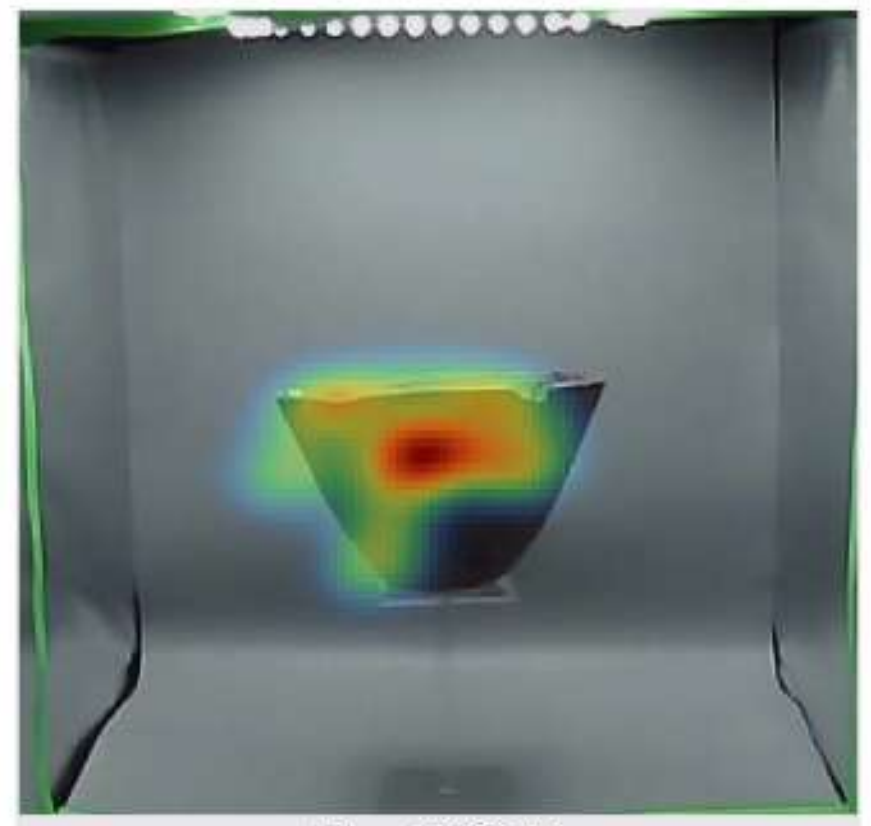
Part0097

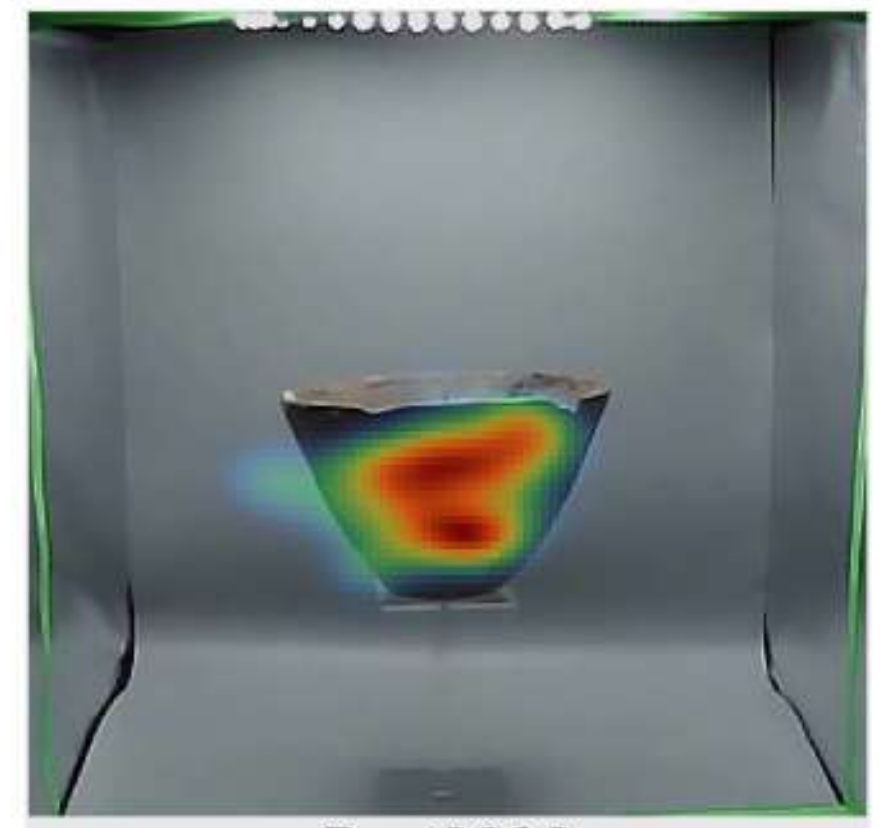
Part0098

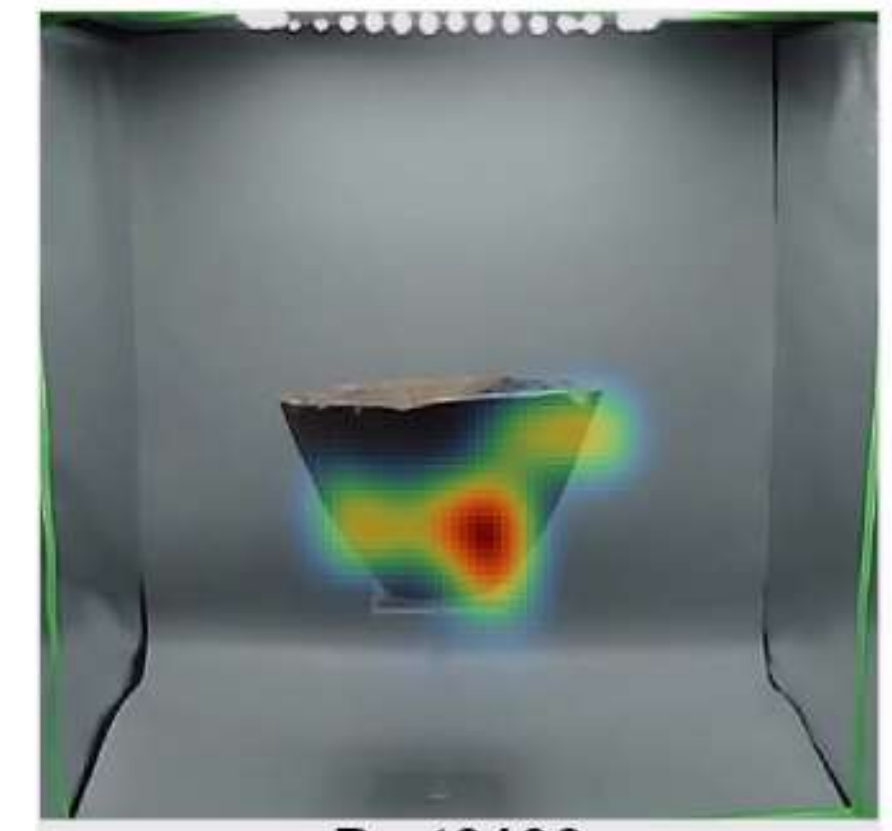
Part0100

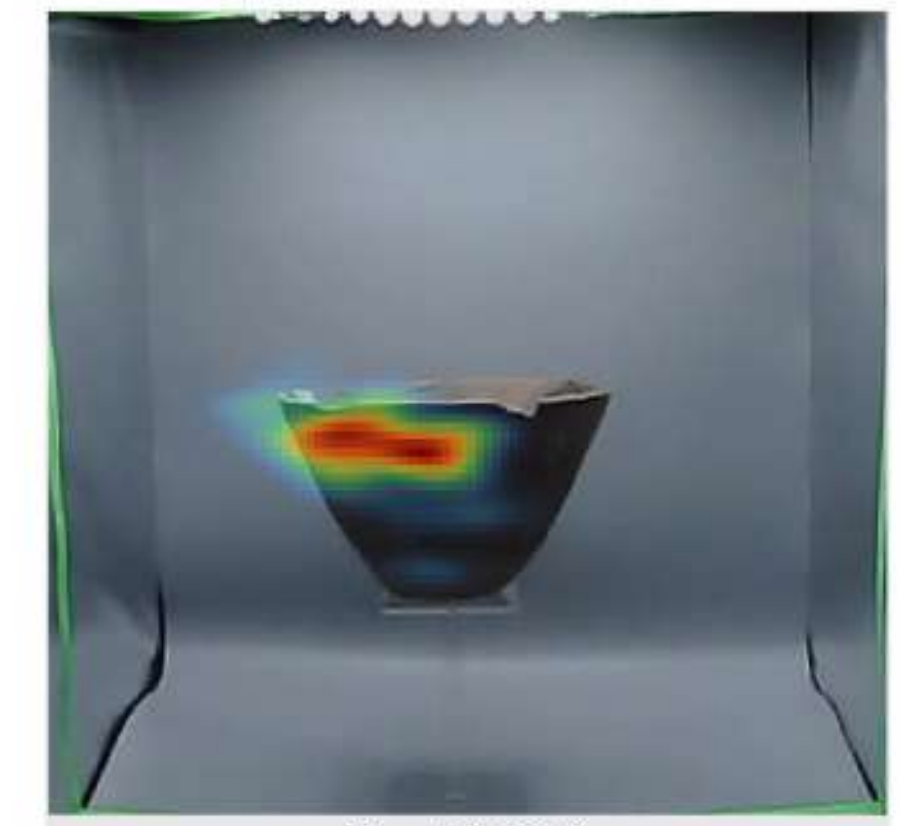
Part0101

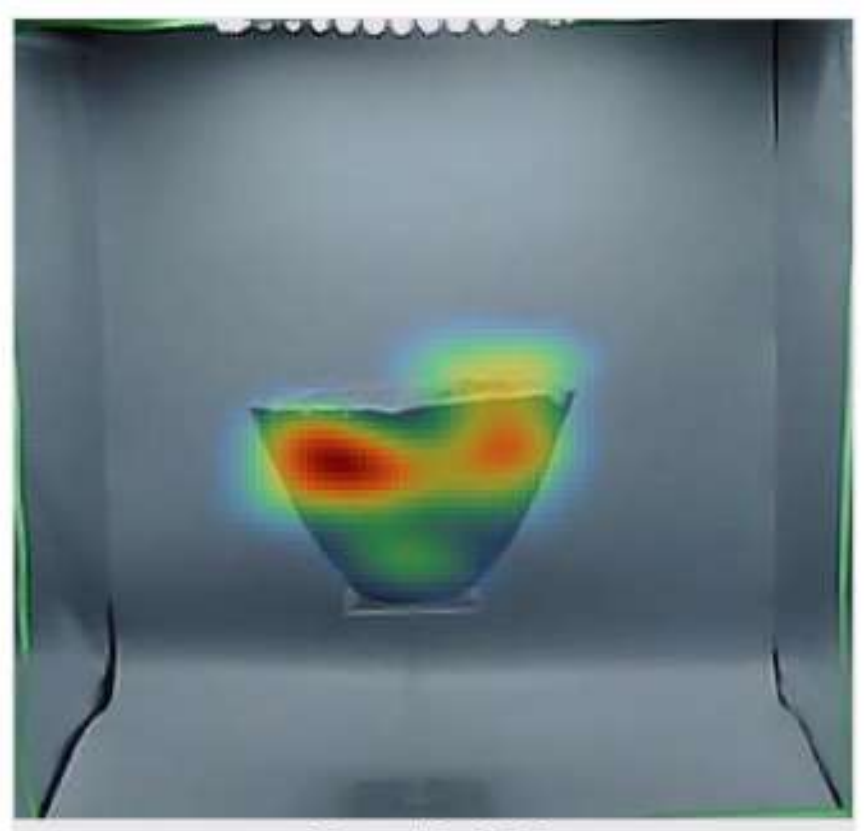
Part0102

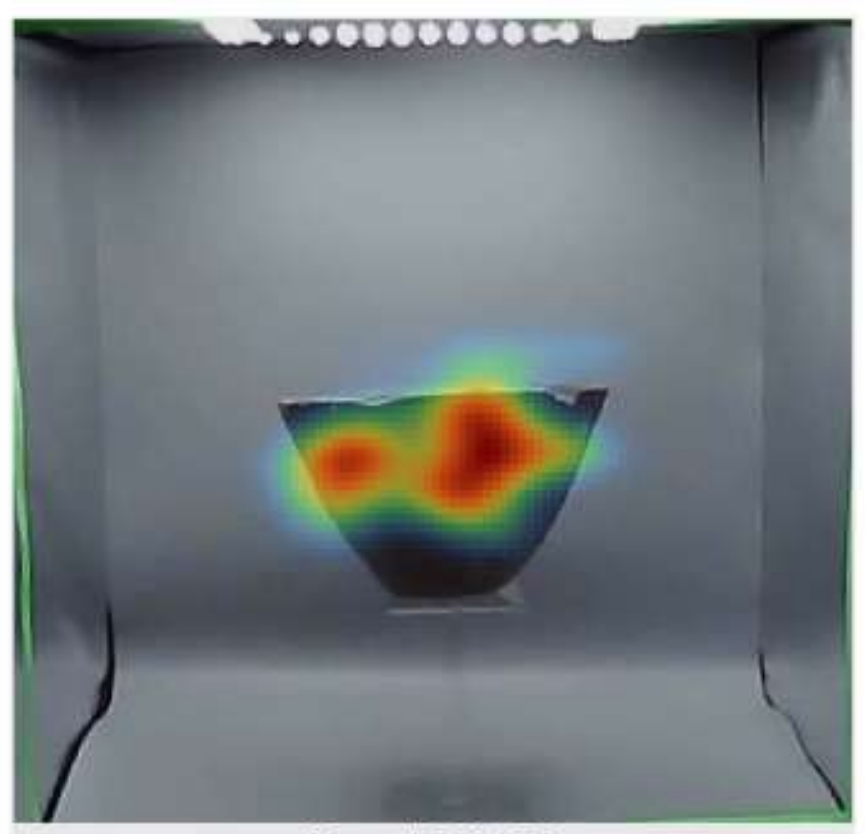
Part0103

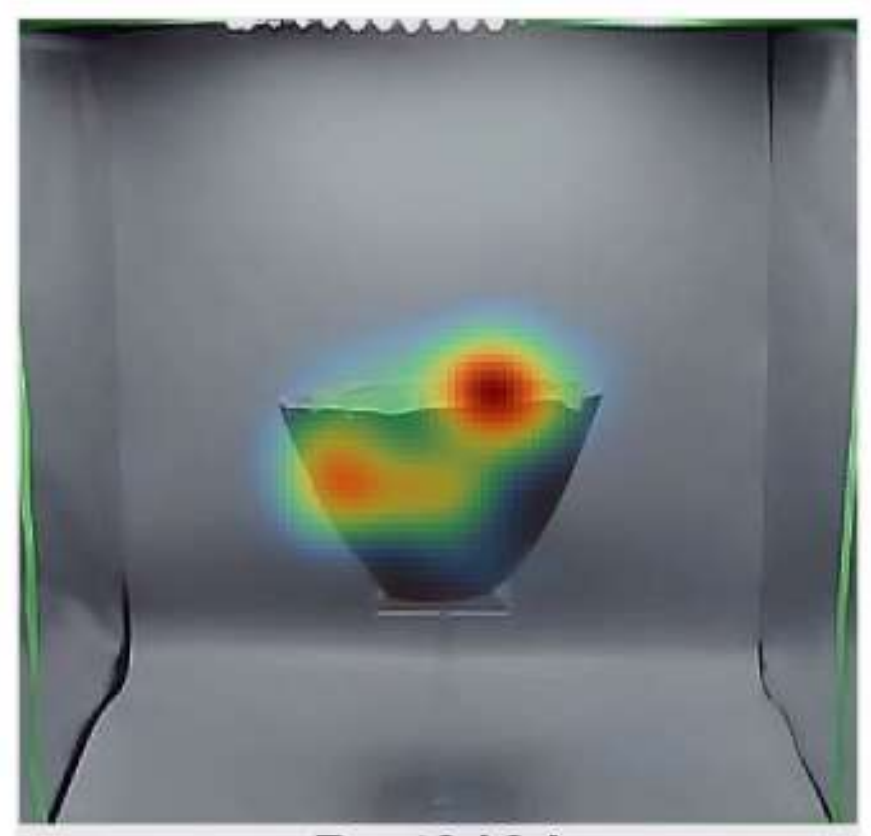
Part0104

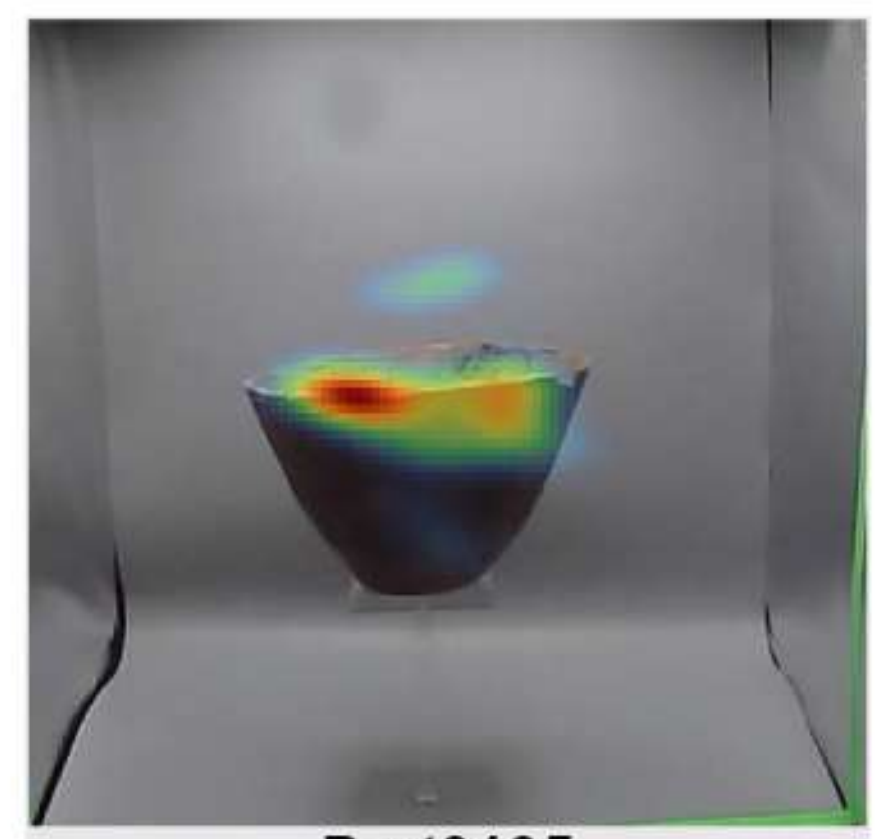
Part0105

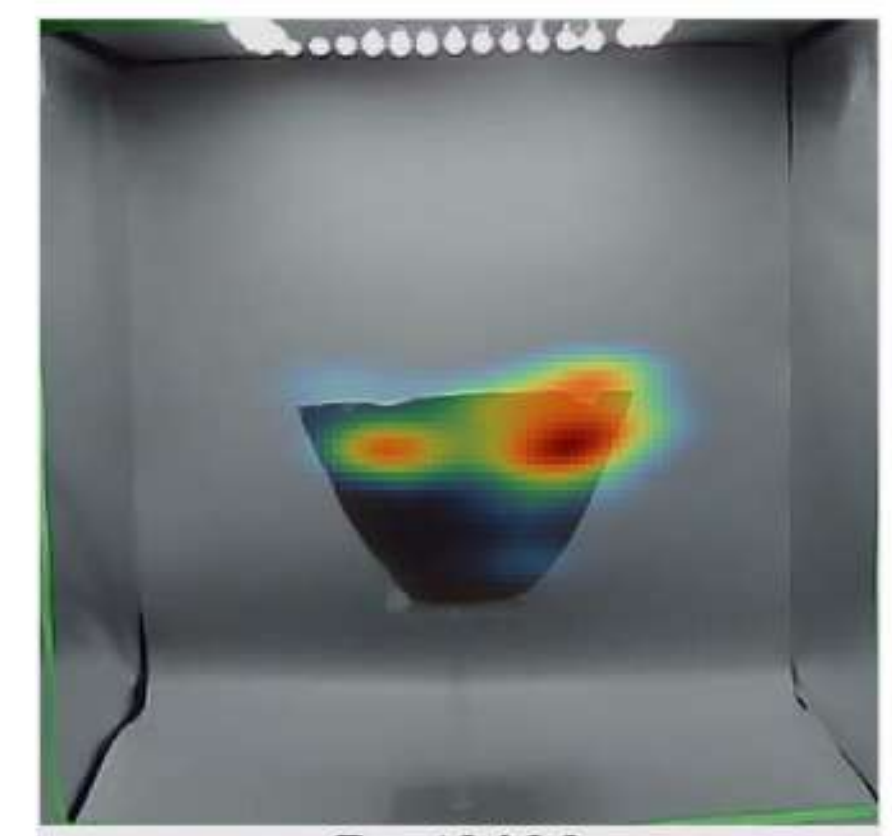
Part0106

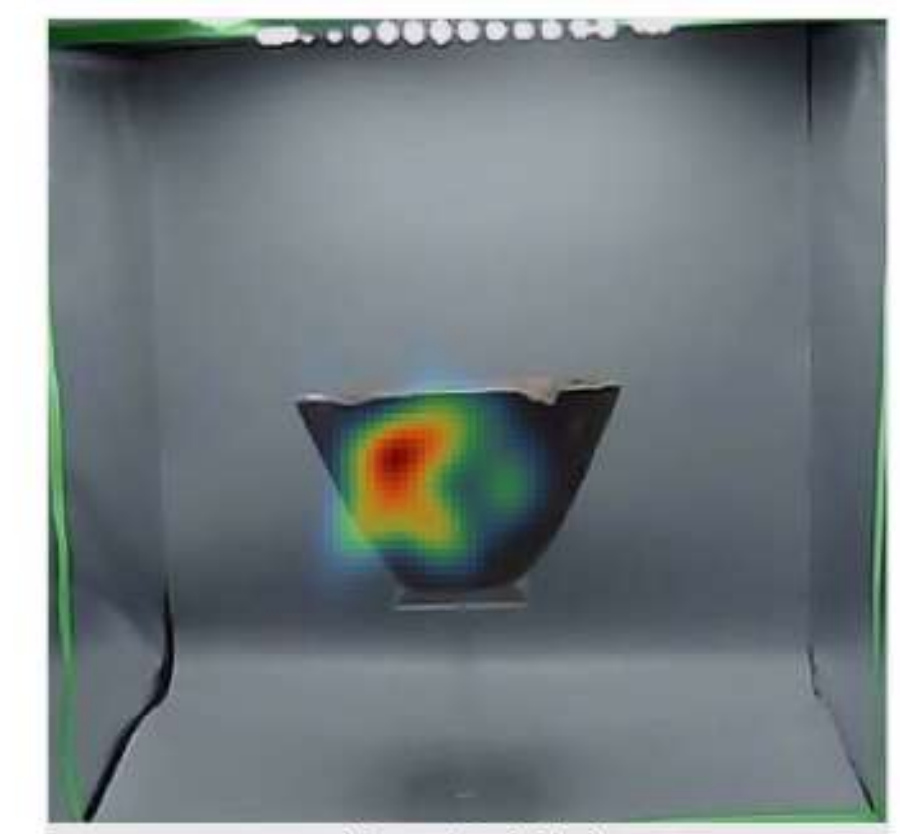
Part0107

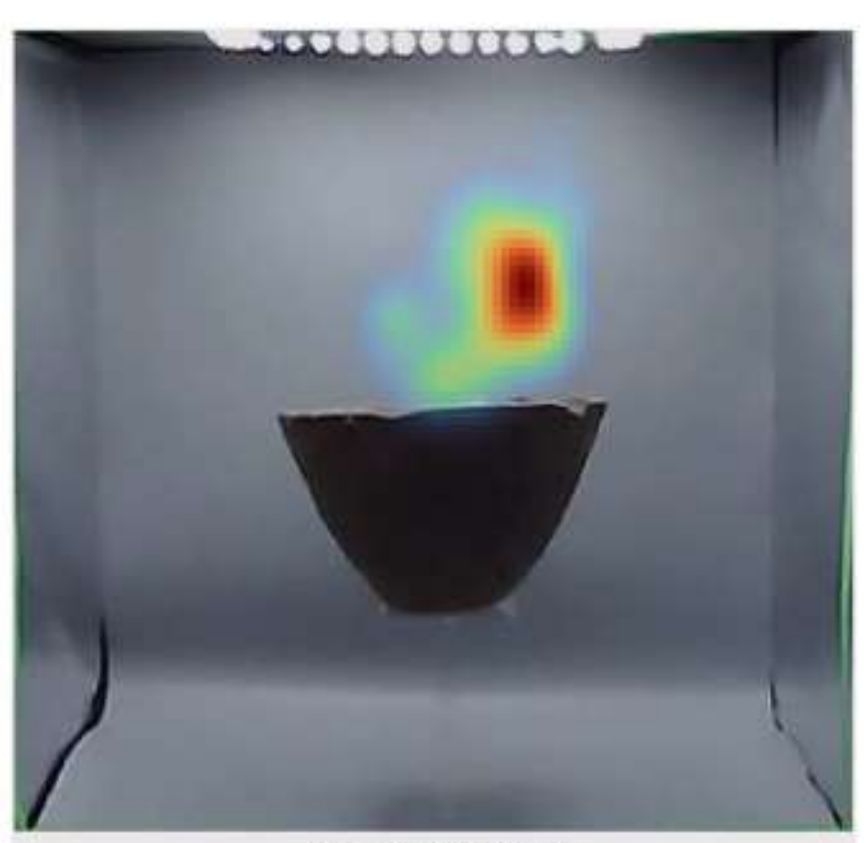
Part0108

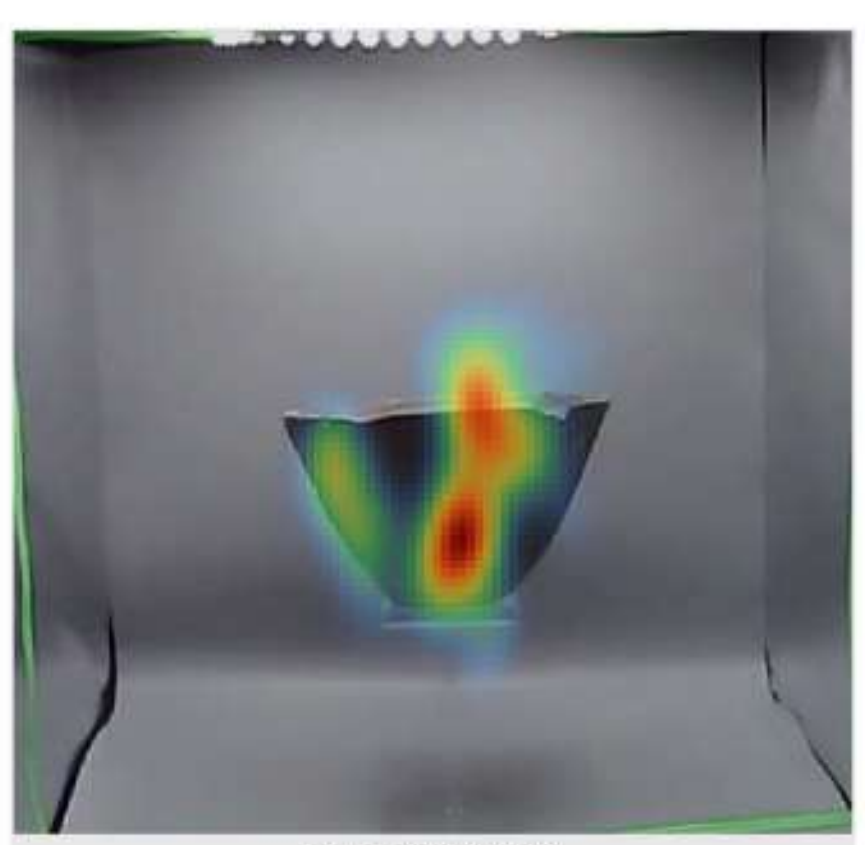
Part0109

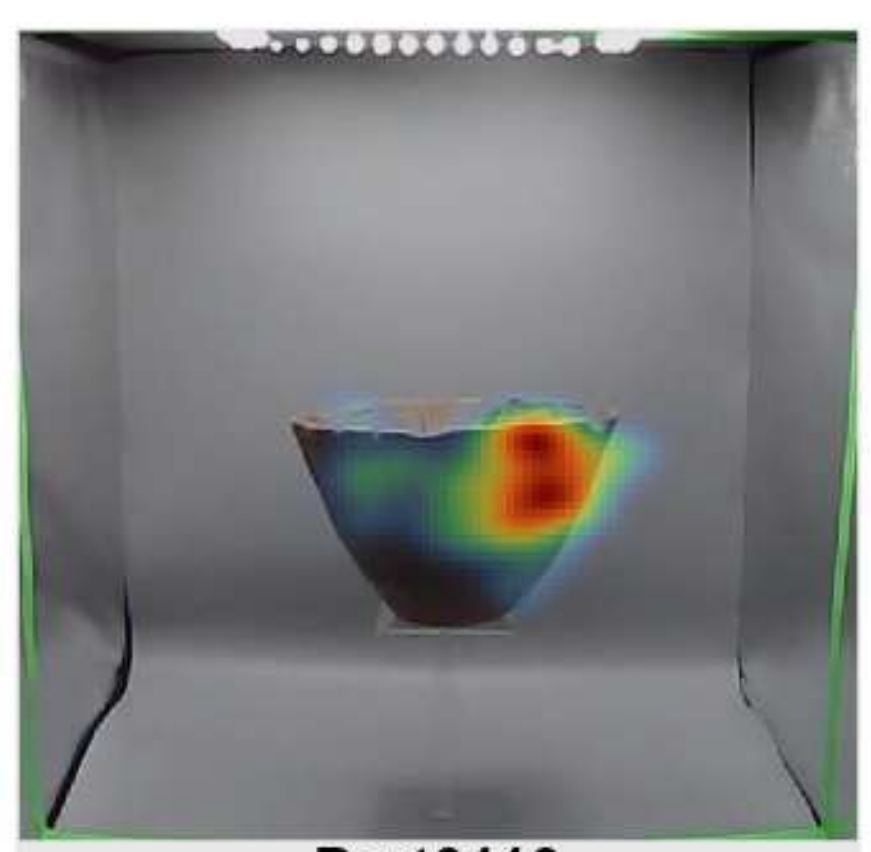
Part0110

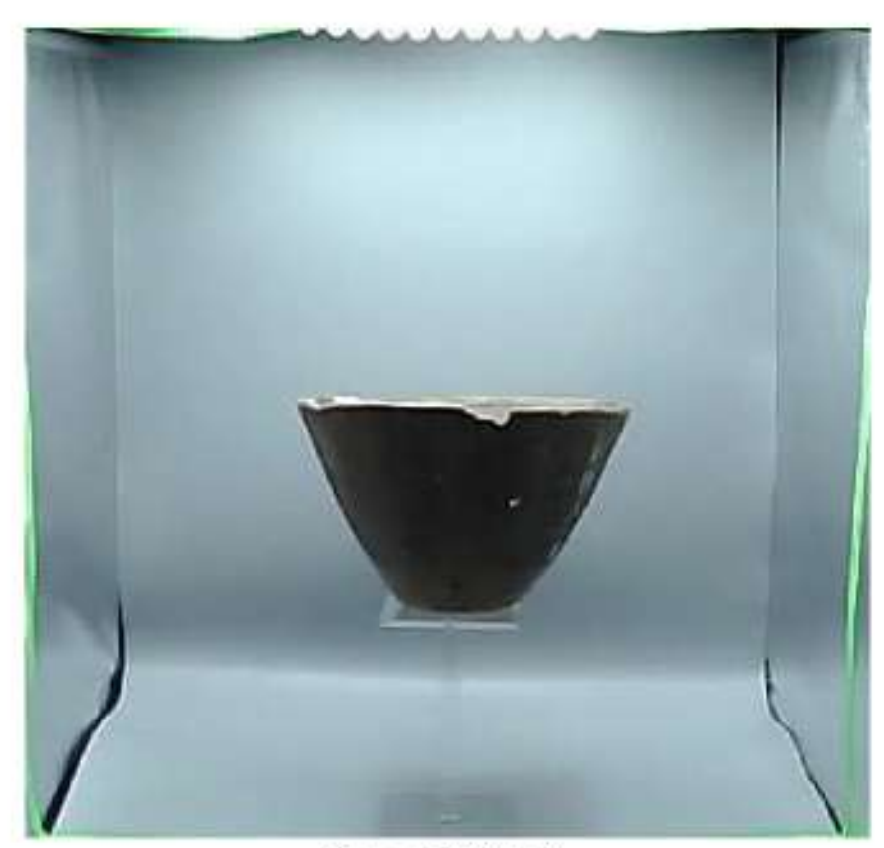
Part0111

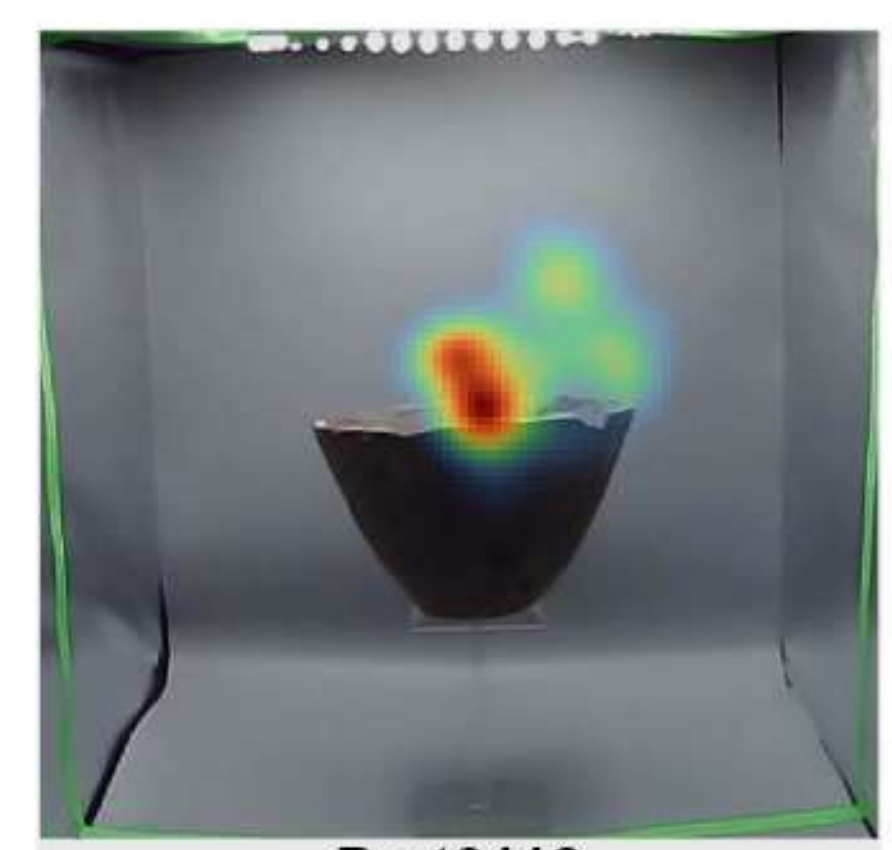
Part0112

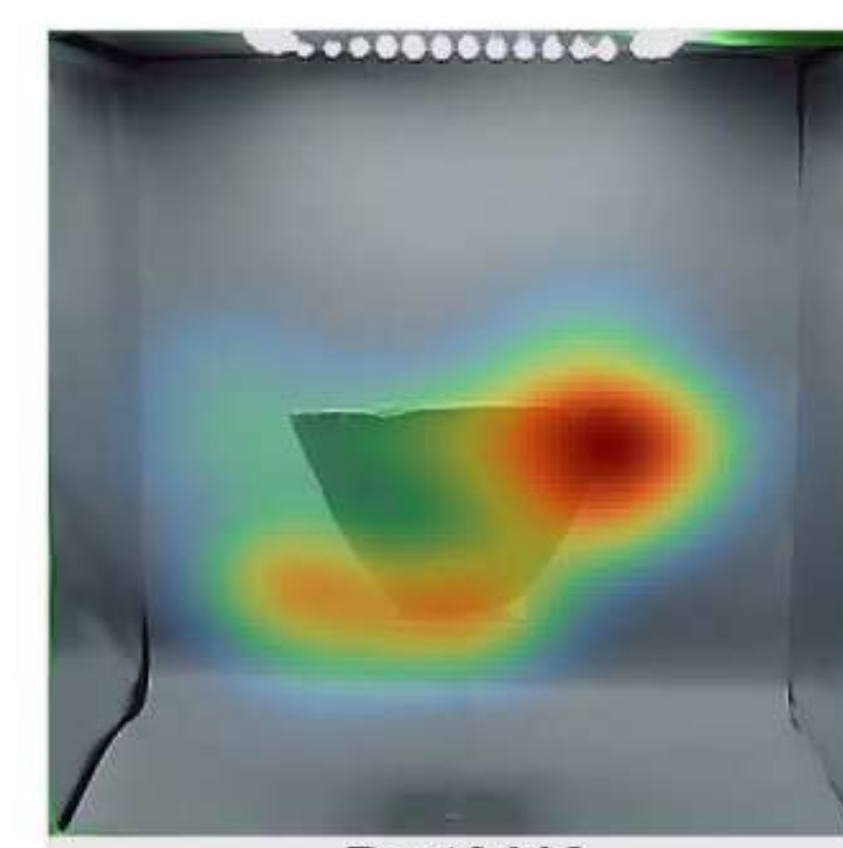
Part0113

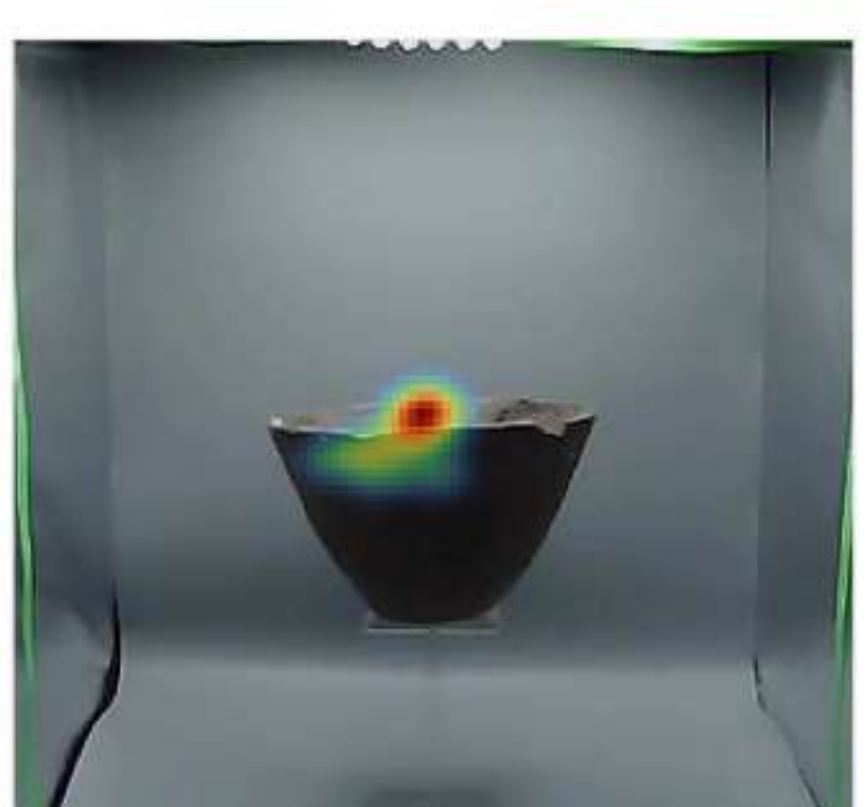
Part0114

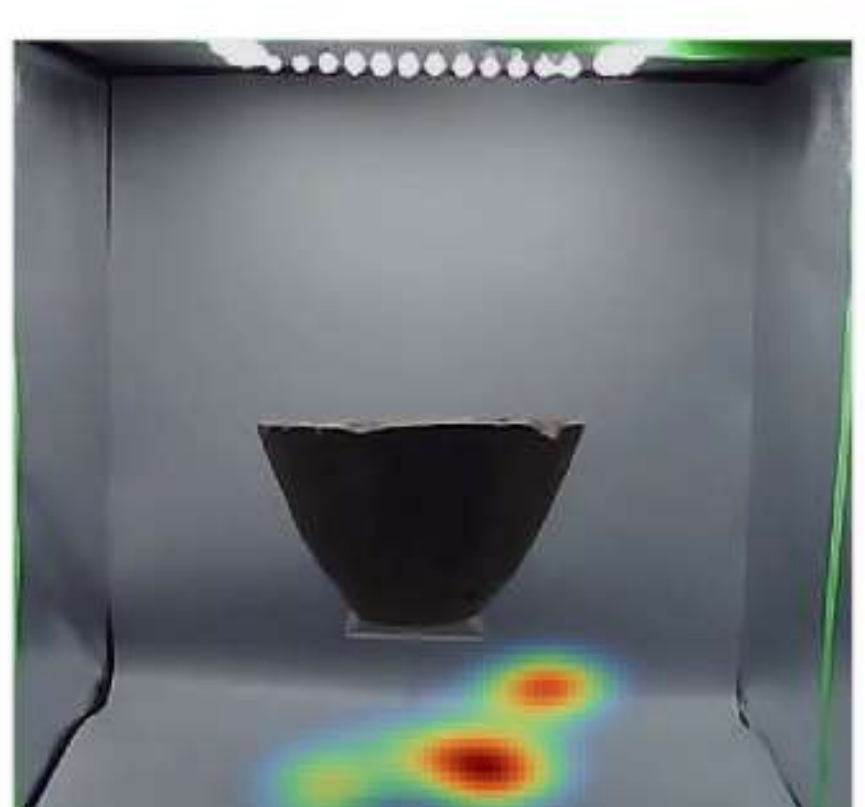
Part0115

Obj0240

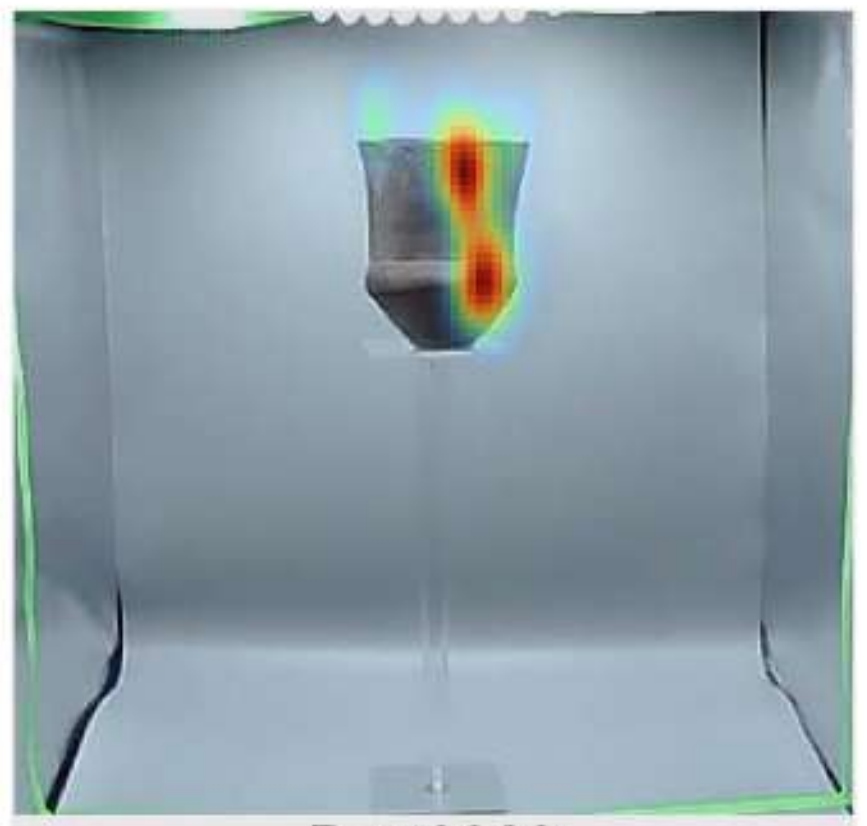
Part0089

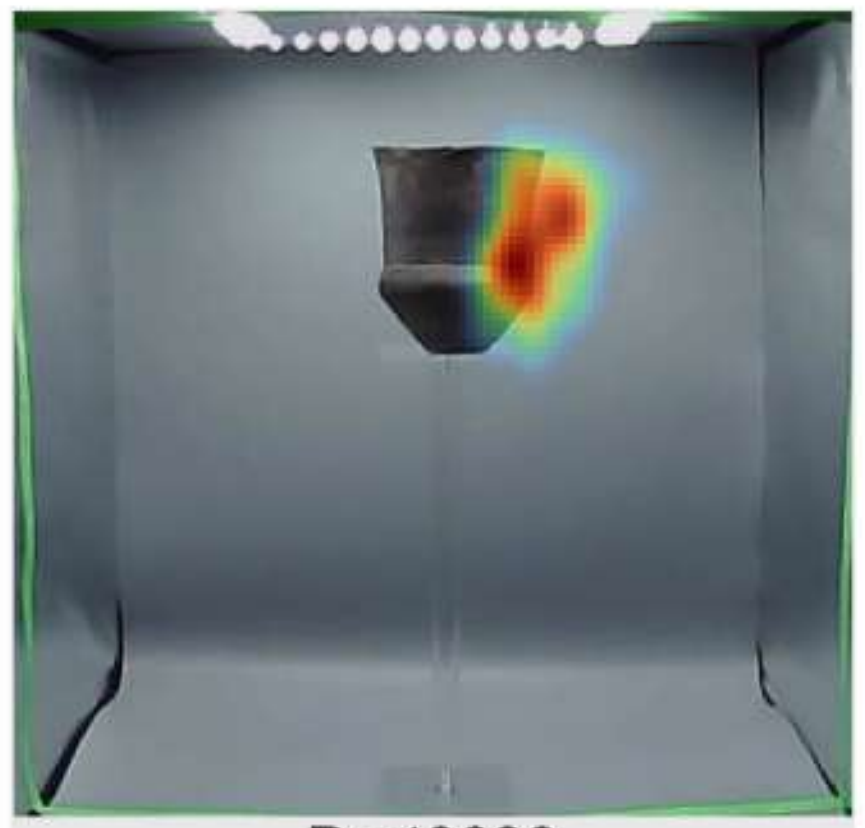
Part0090

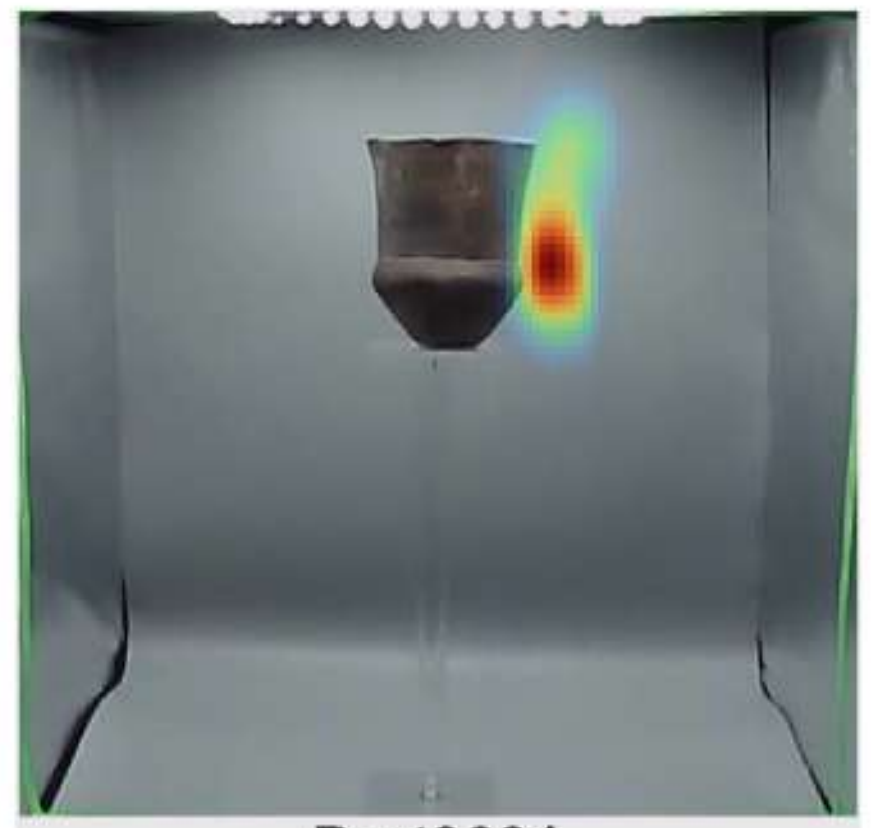
Part0091

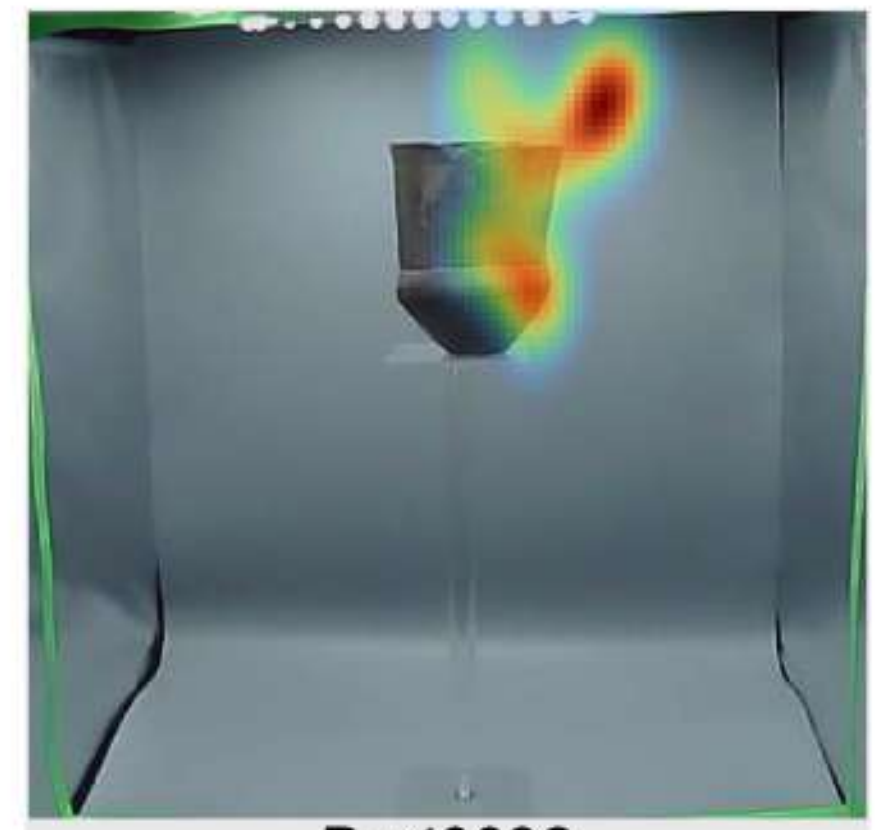
Part0092

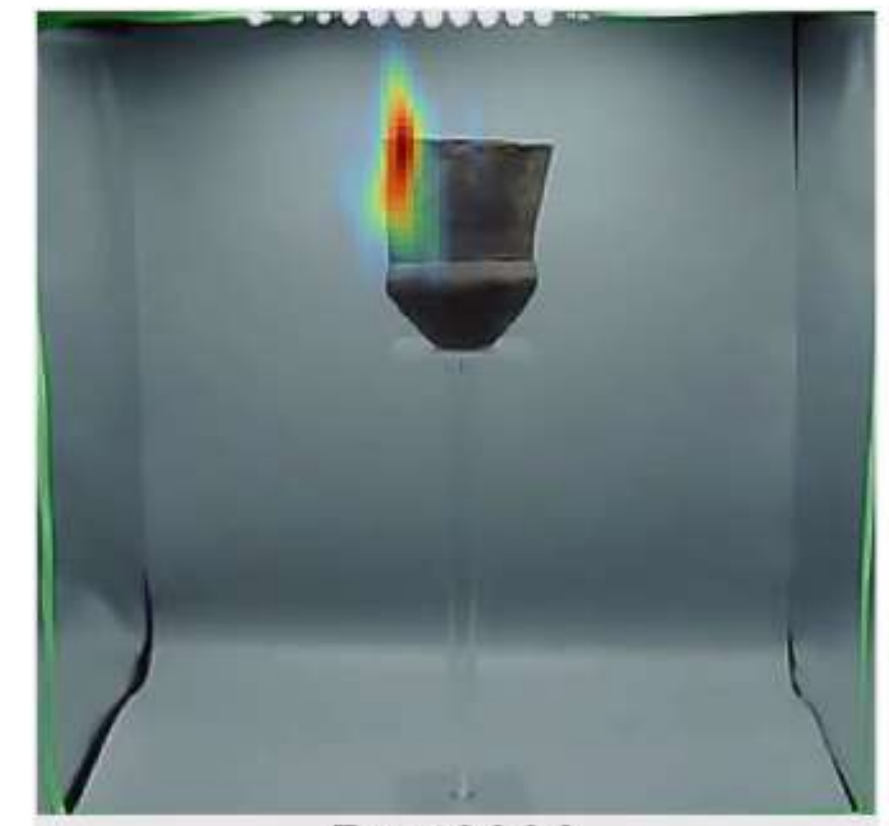
Part0093

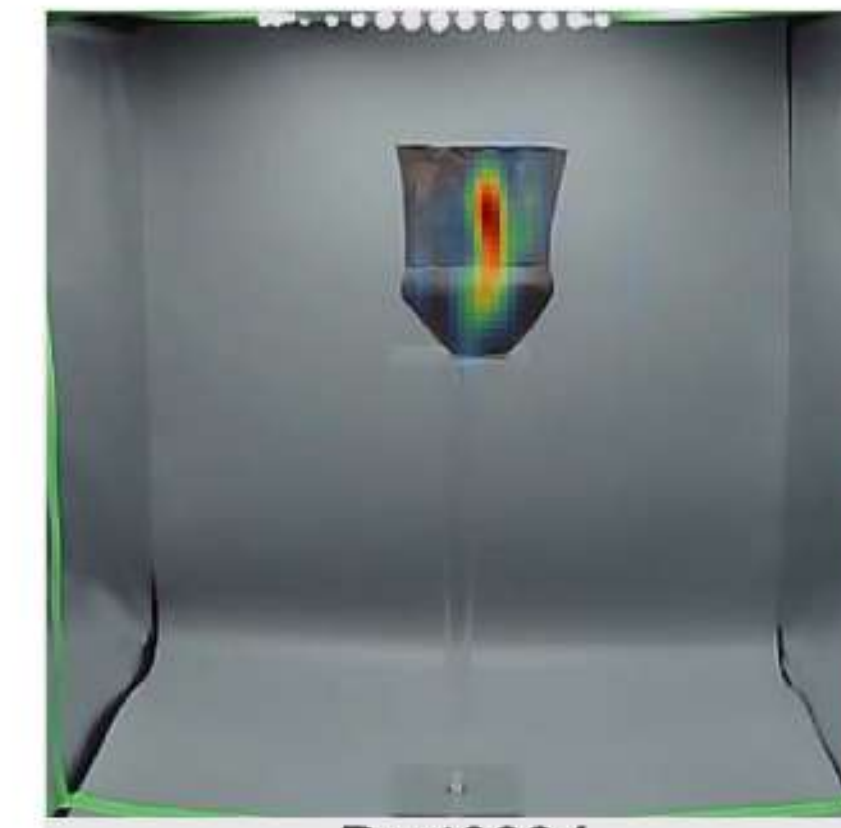
Part0094

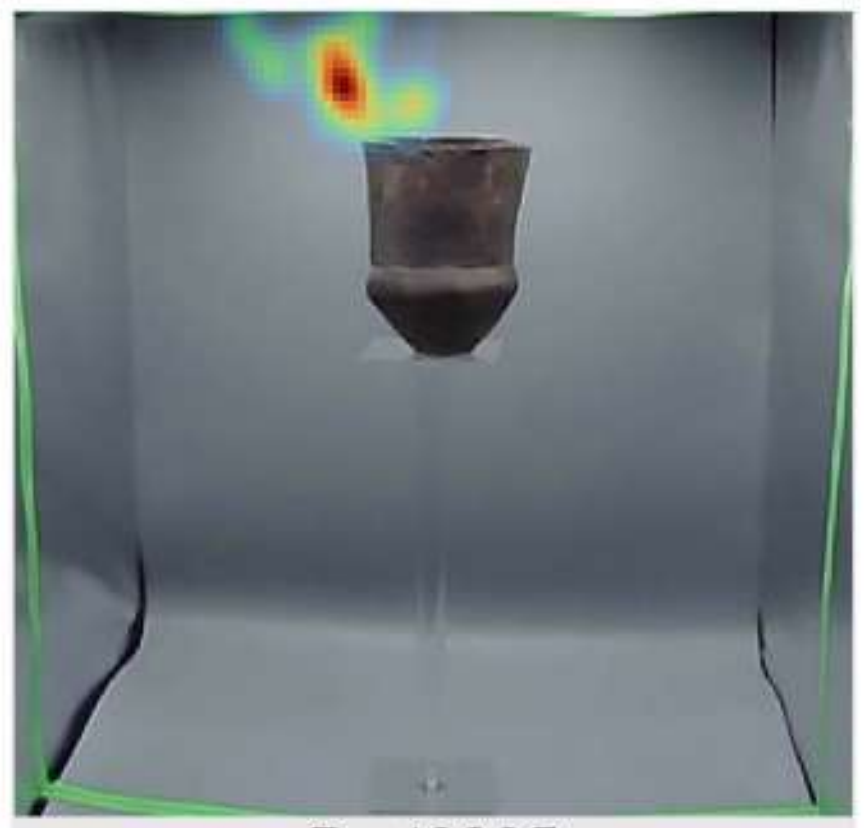
Part0095

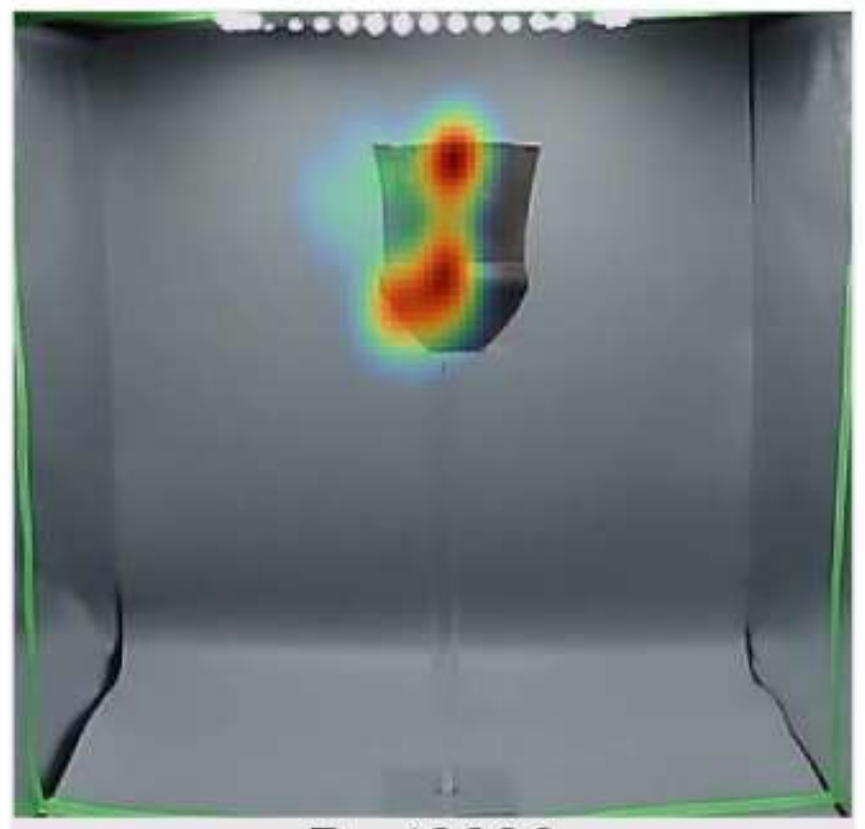
Part0096

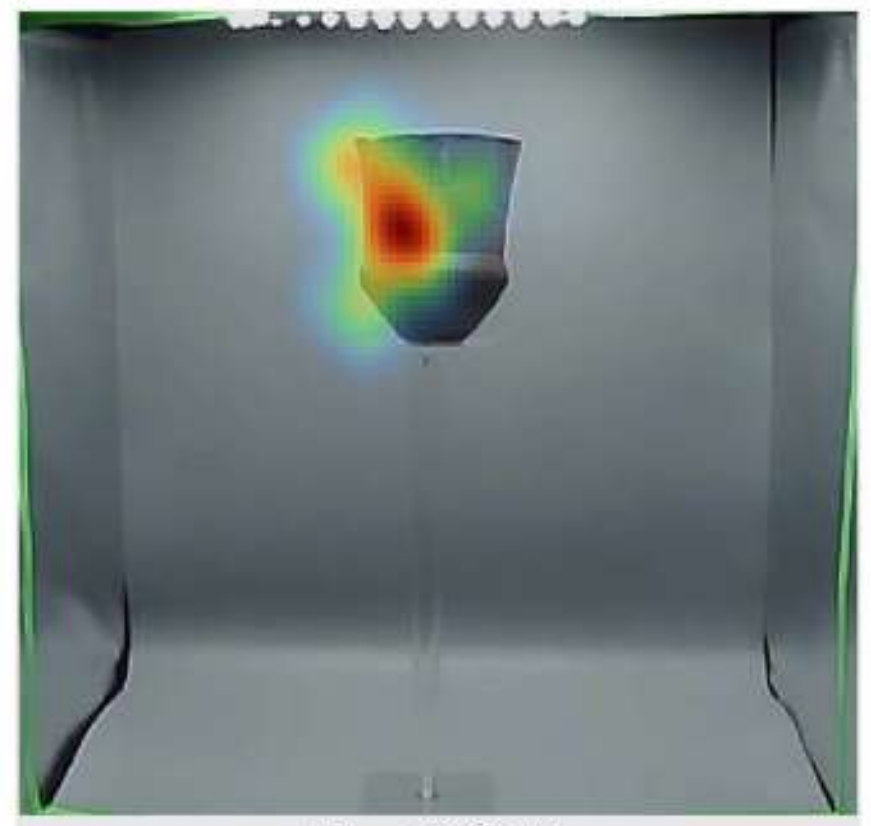
Part0097

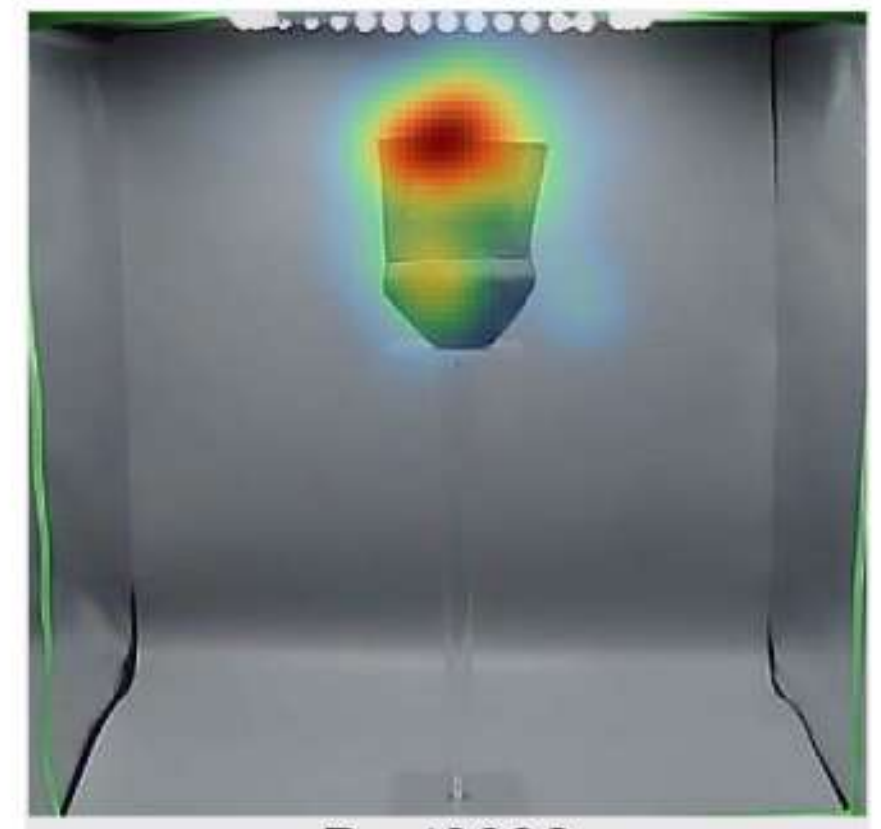
Part0098

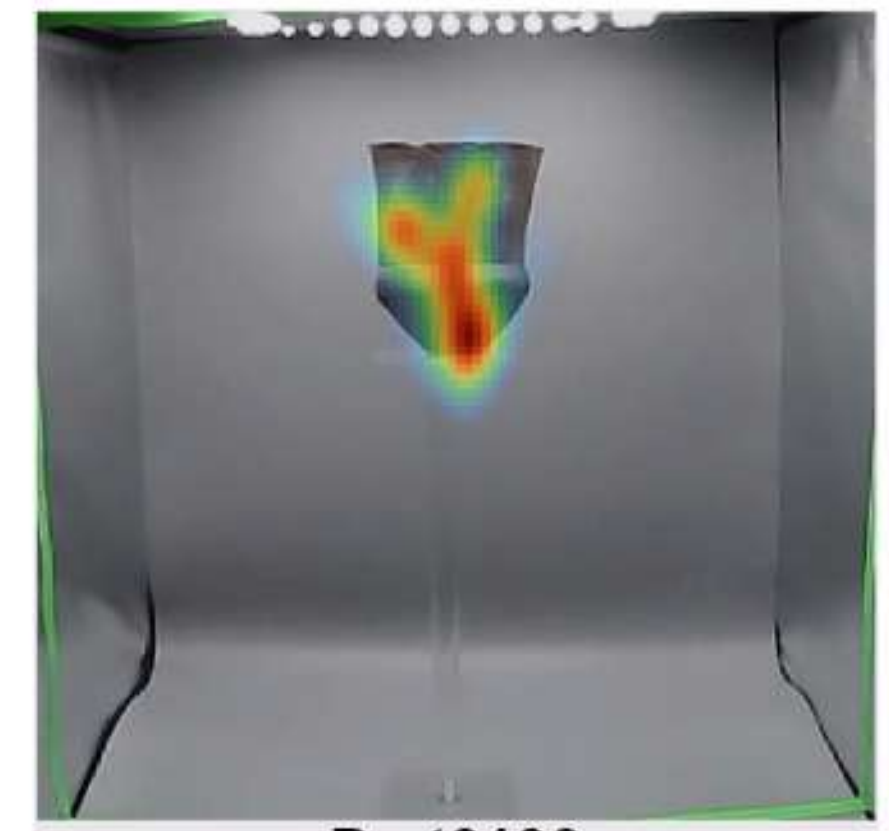
Part0100

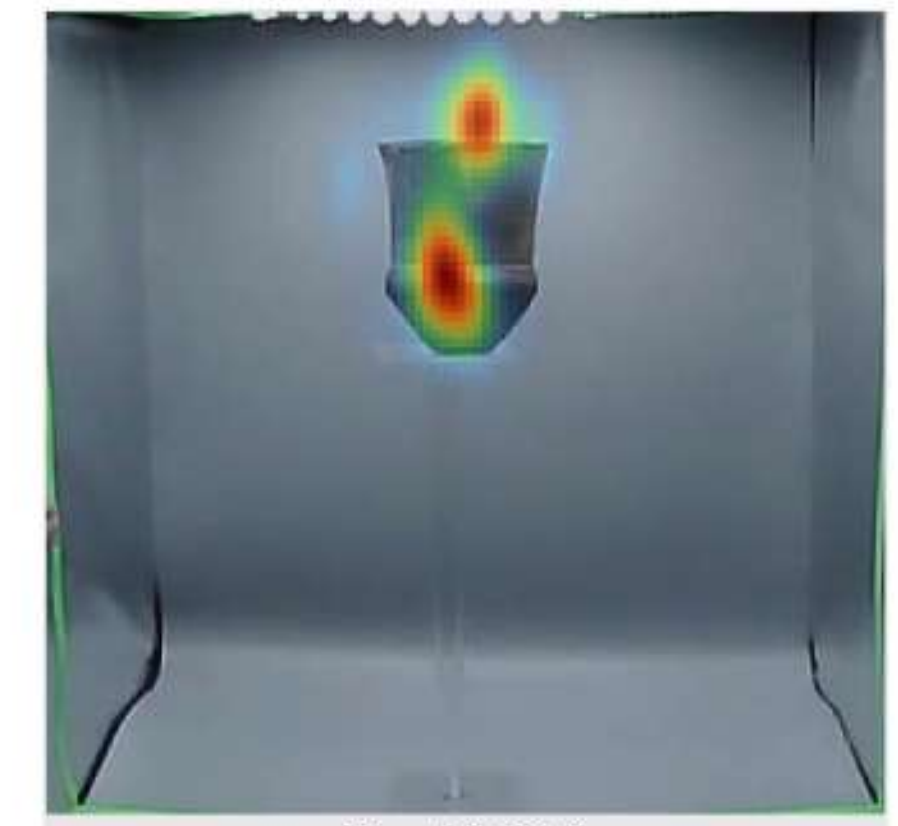
Part0101

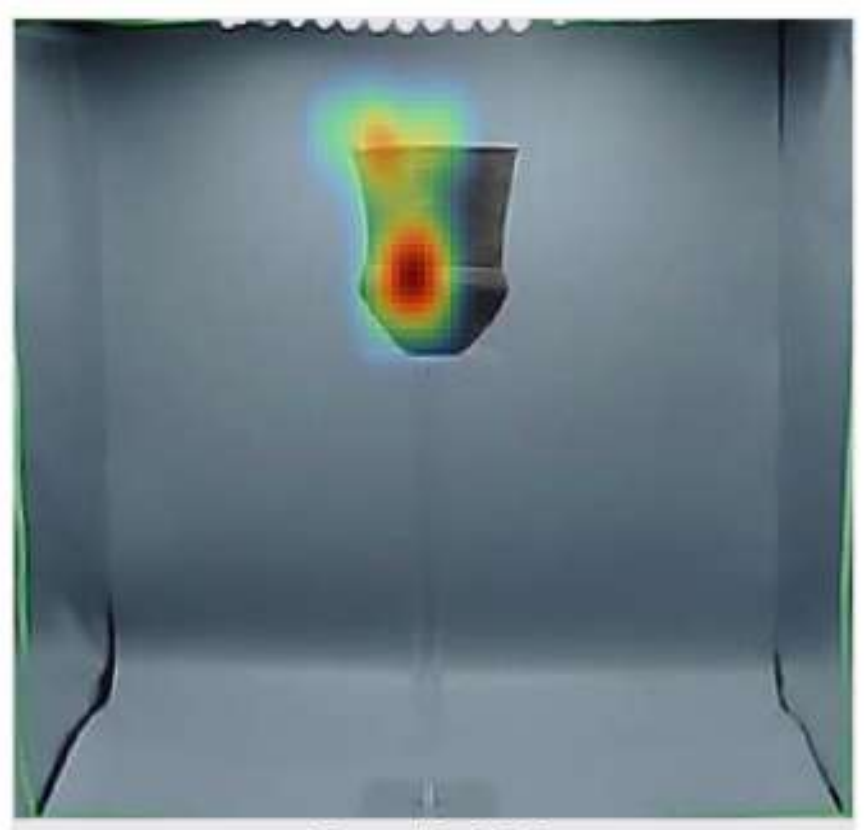
Part0102

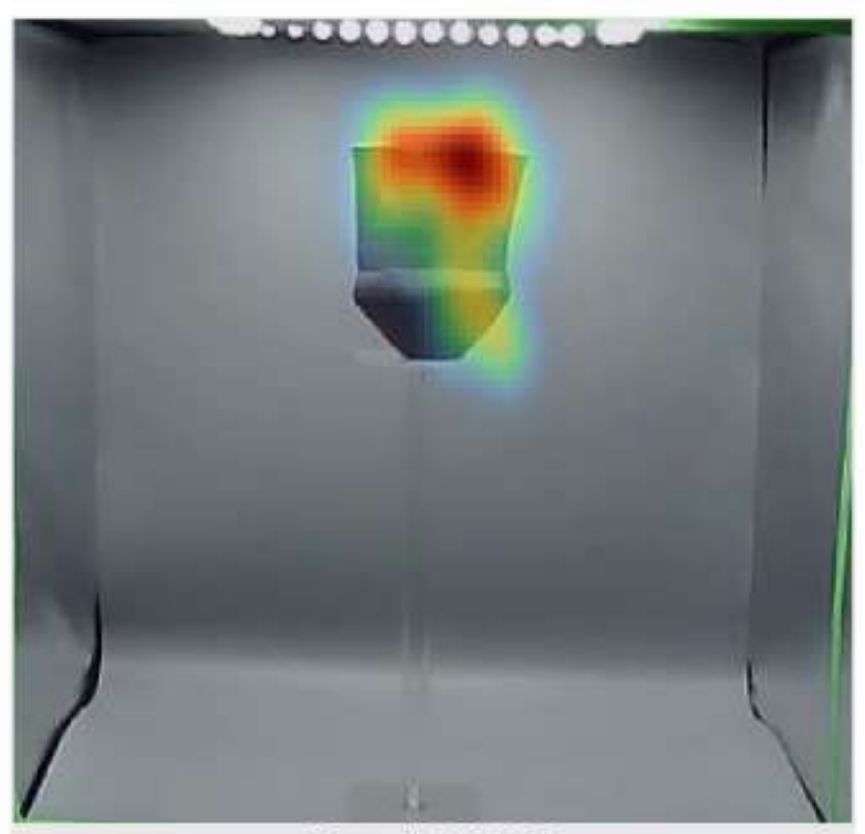
Part0103

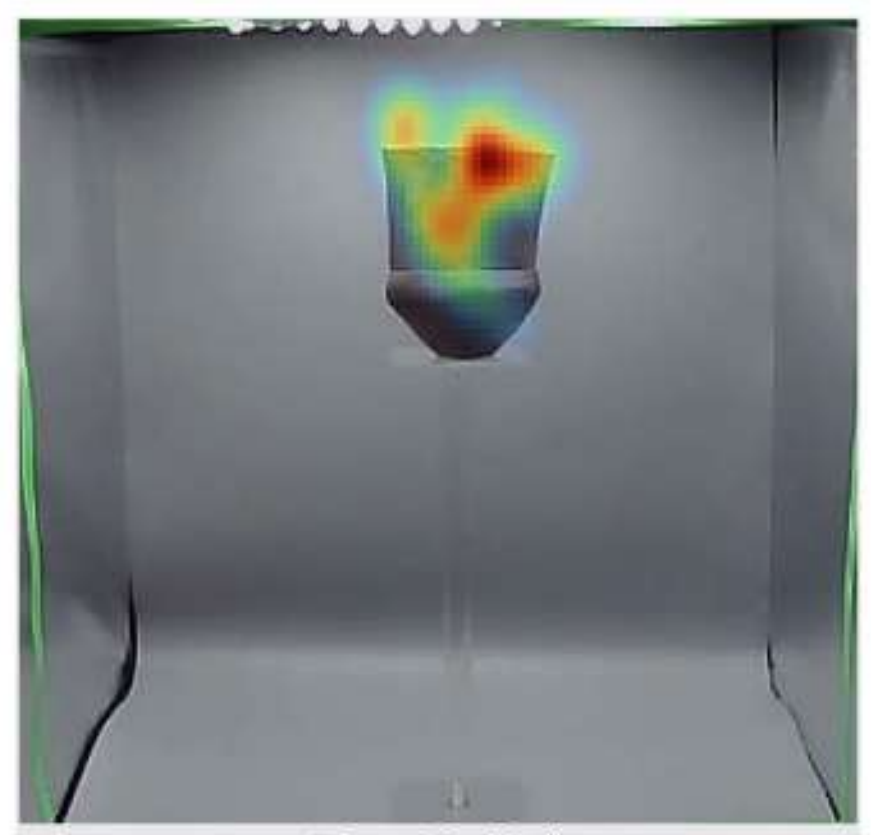
Part0104

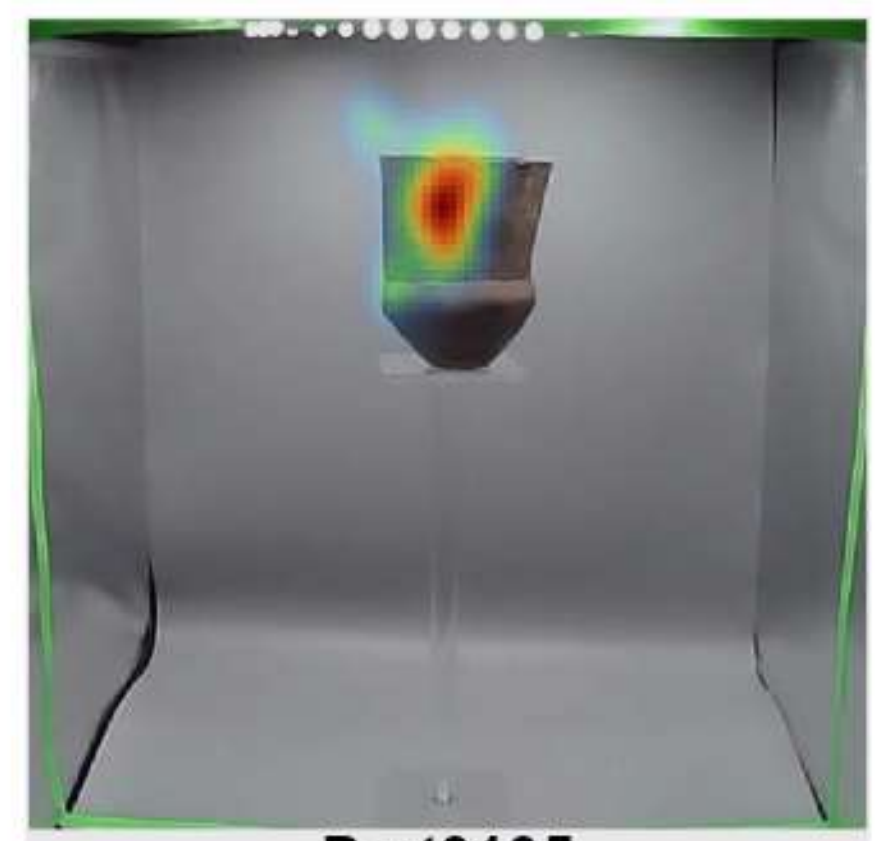
Part0105

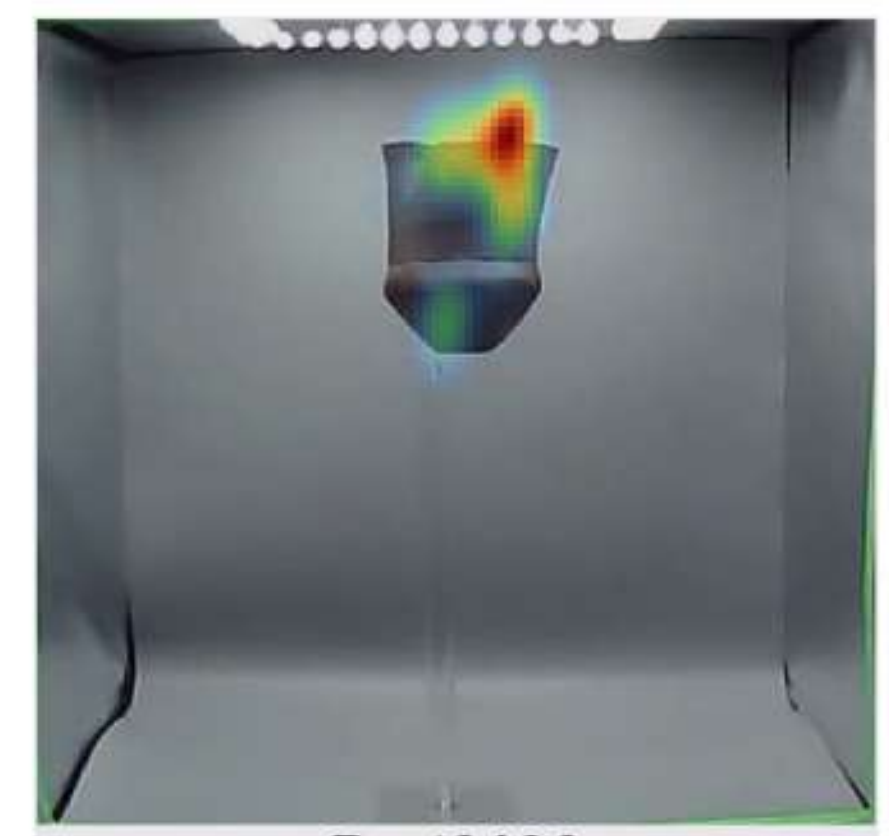
Part0106

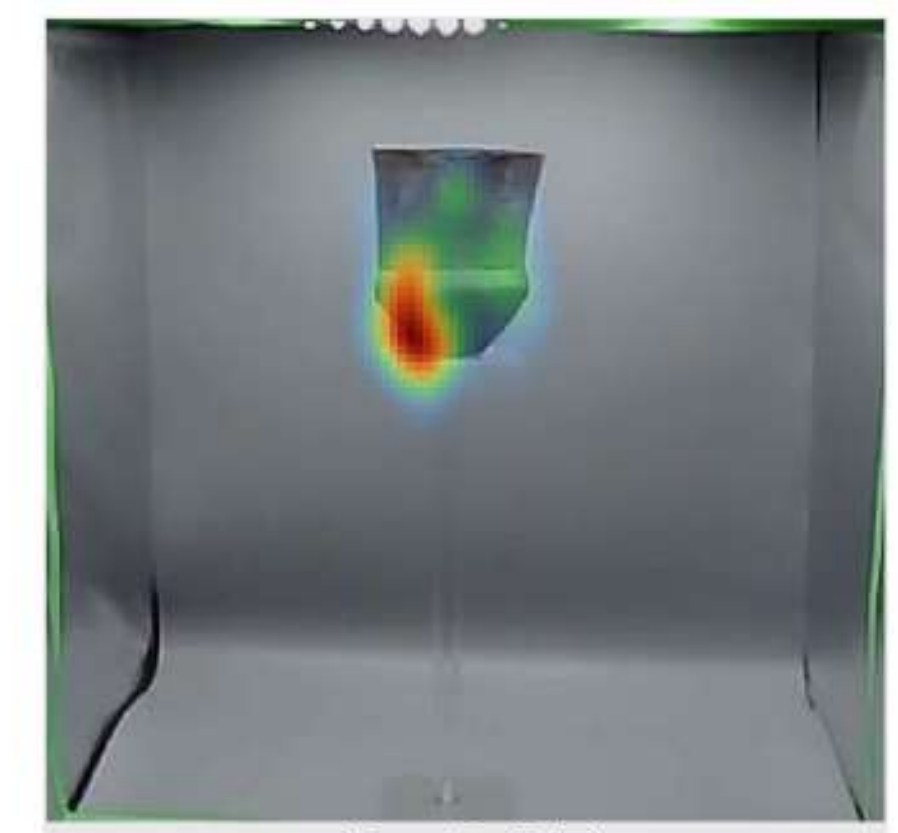
Part0107

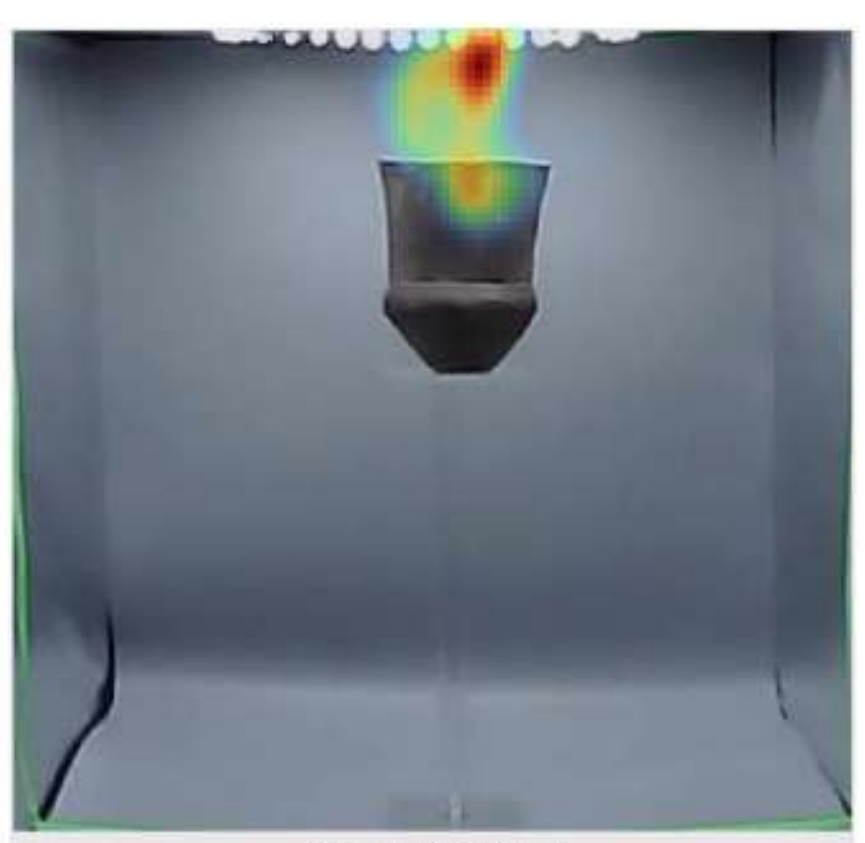
Part0108

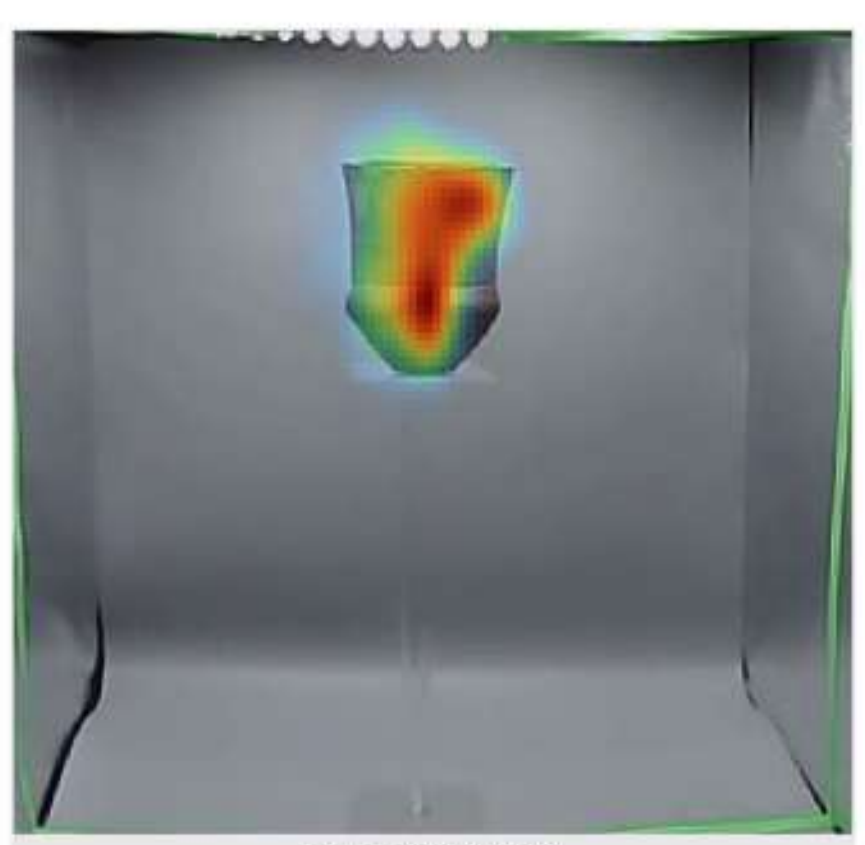
Part0109

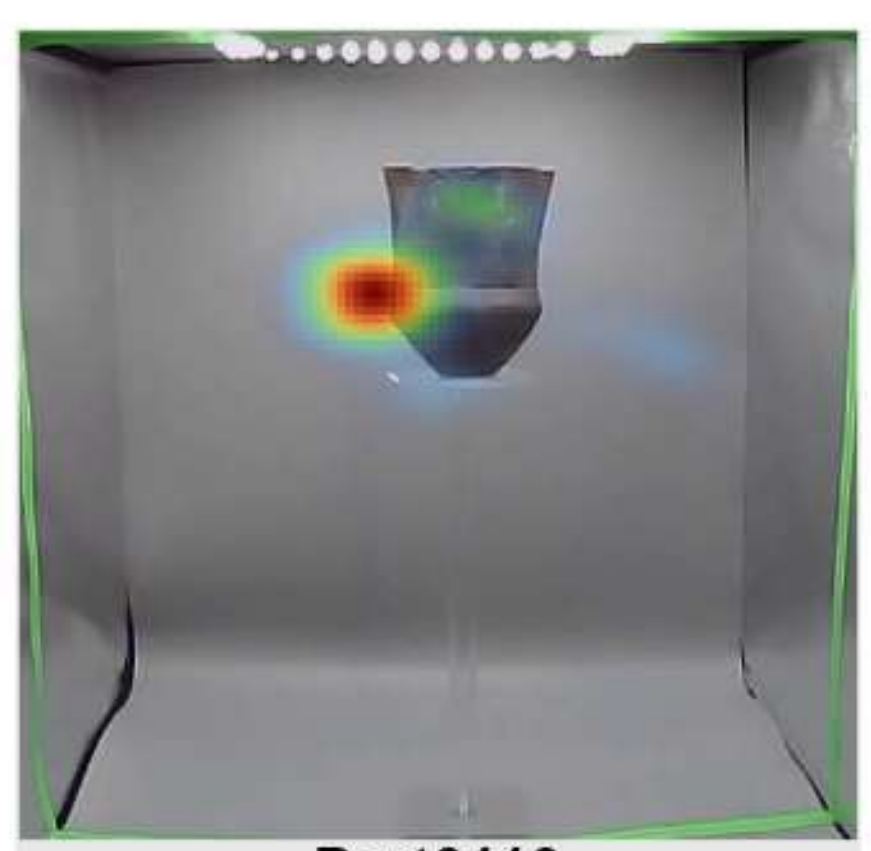
Part0110

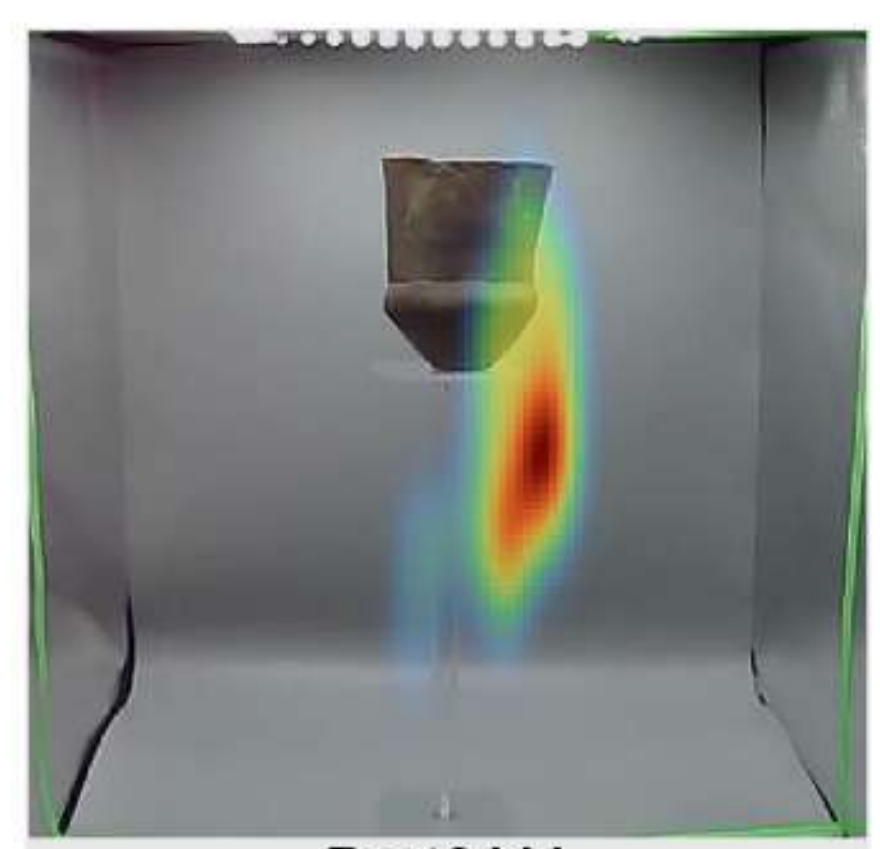
Part0111

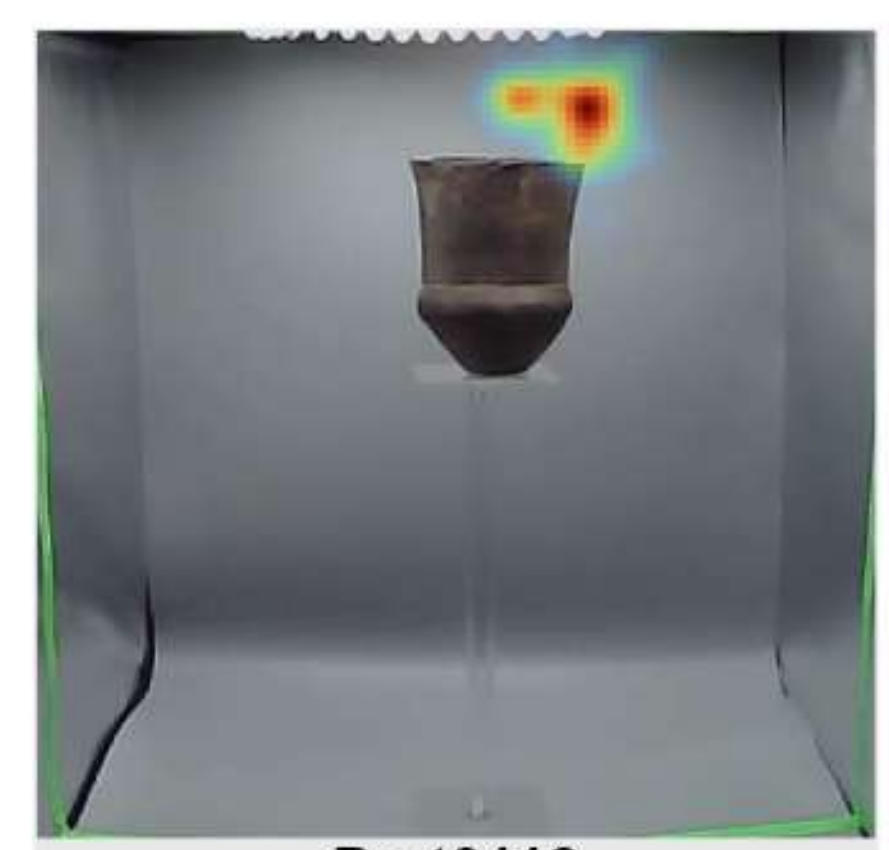
Part0112

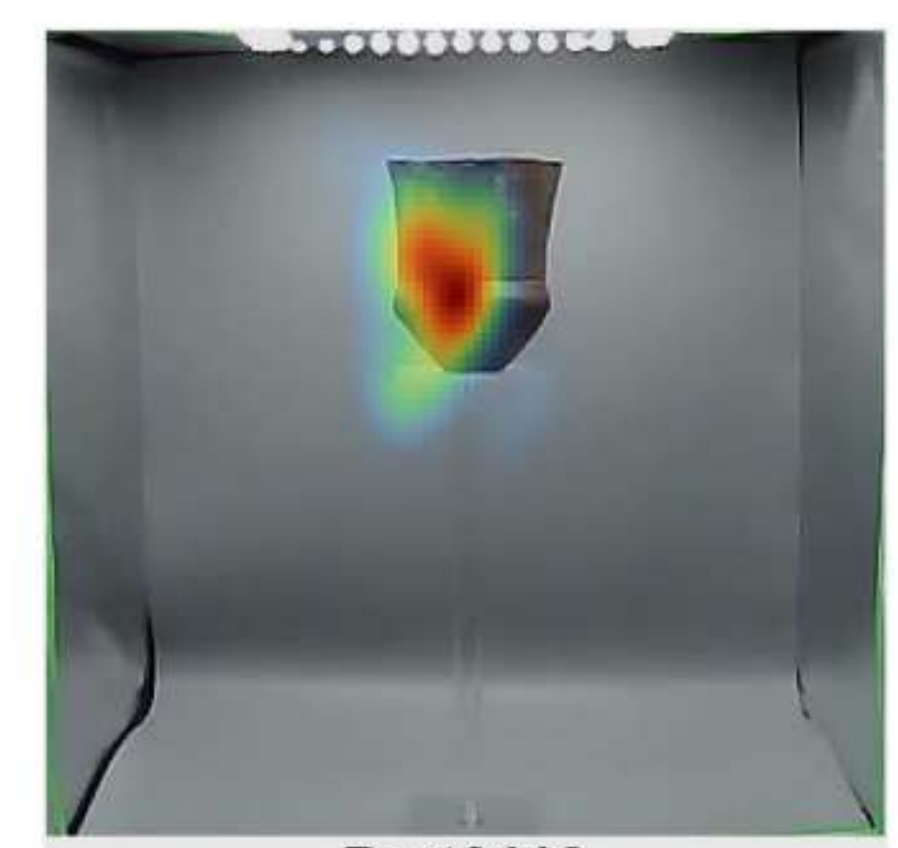
Part0113

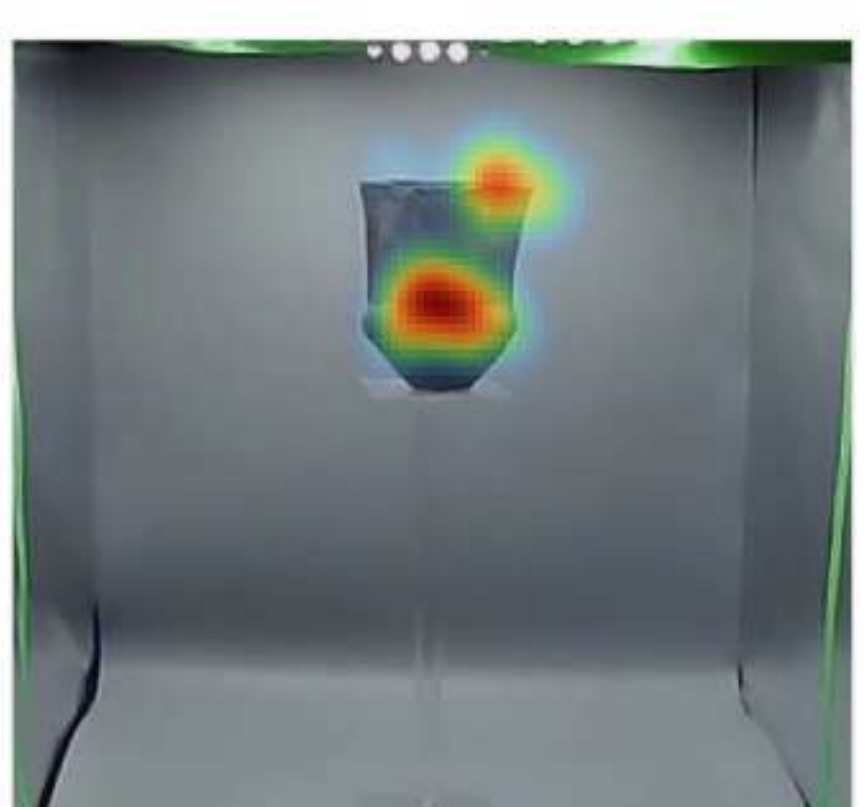
Part0114

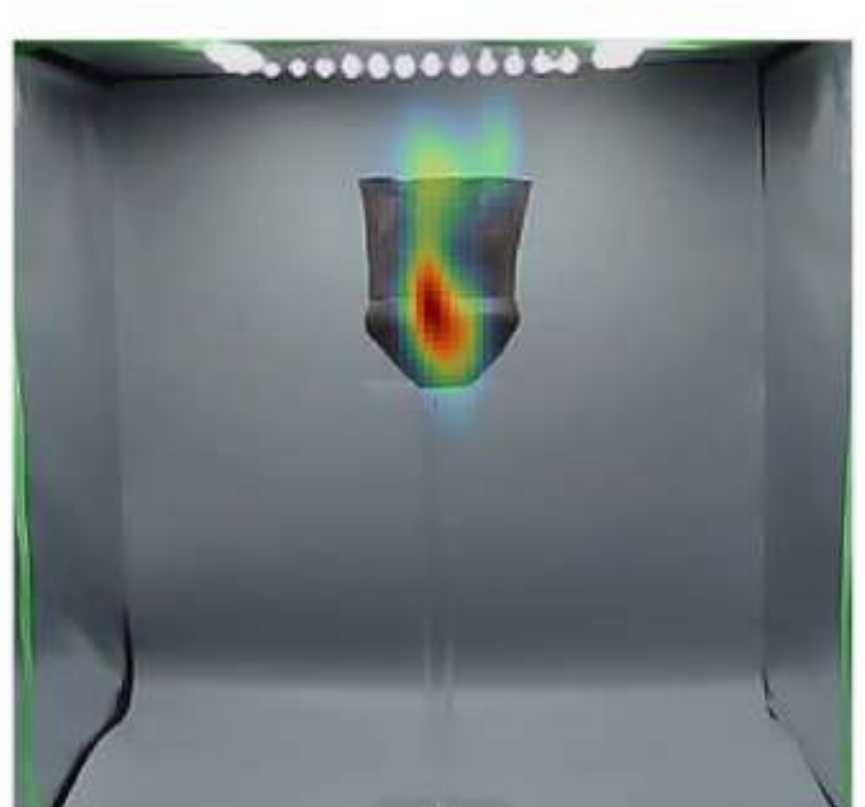
Part0115

Obj0242

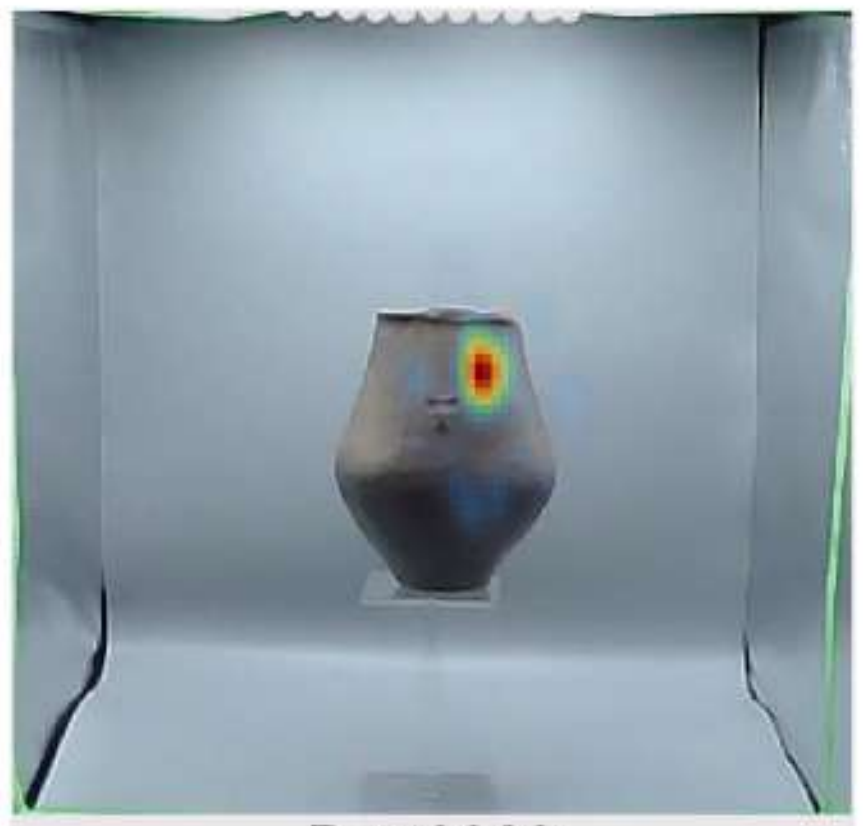
Part0089

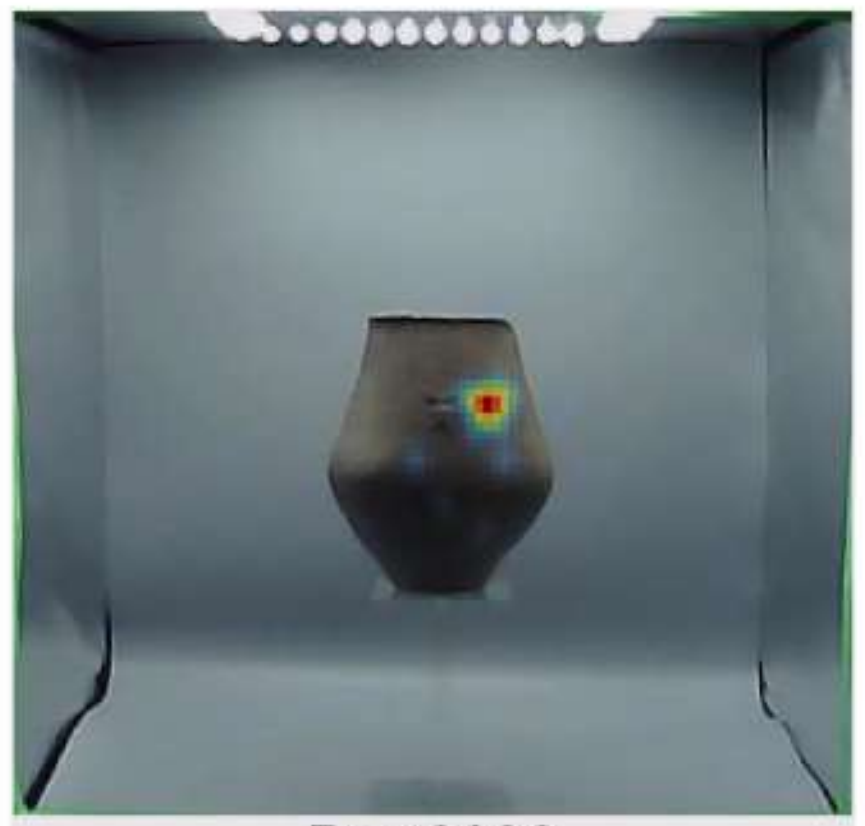
Part0090

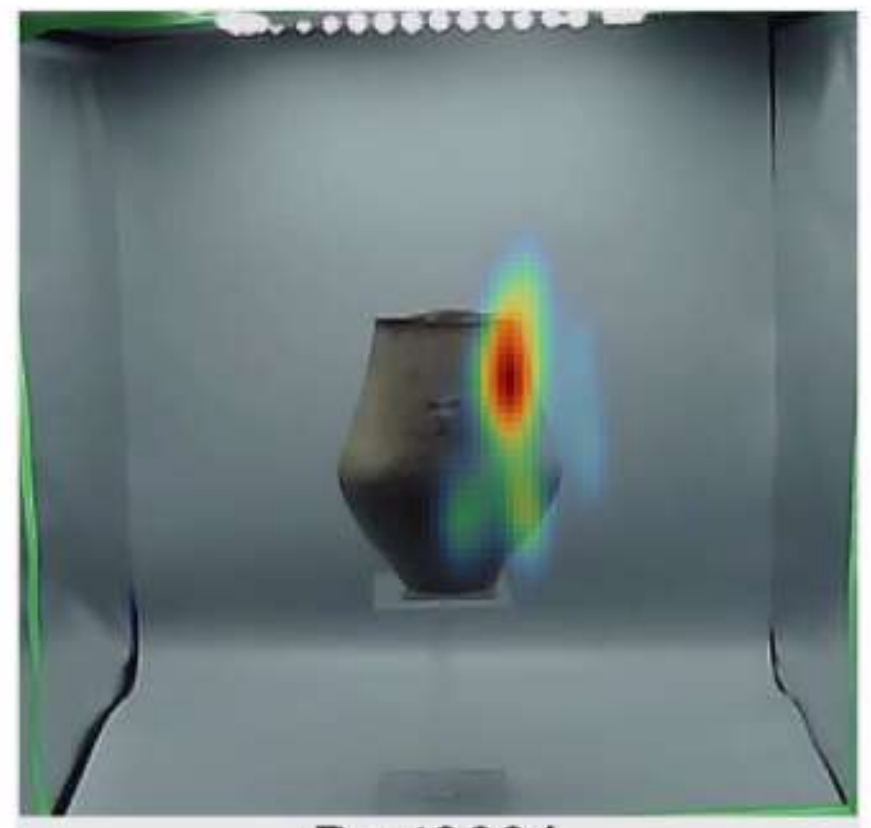
Part0091

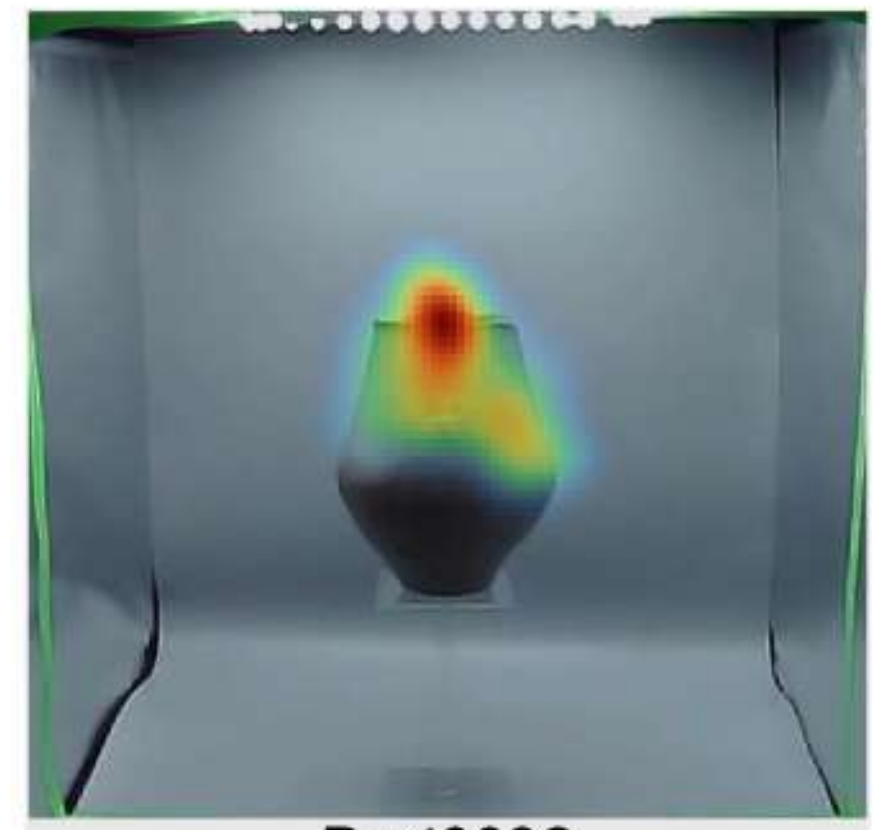
Part0092

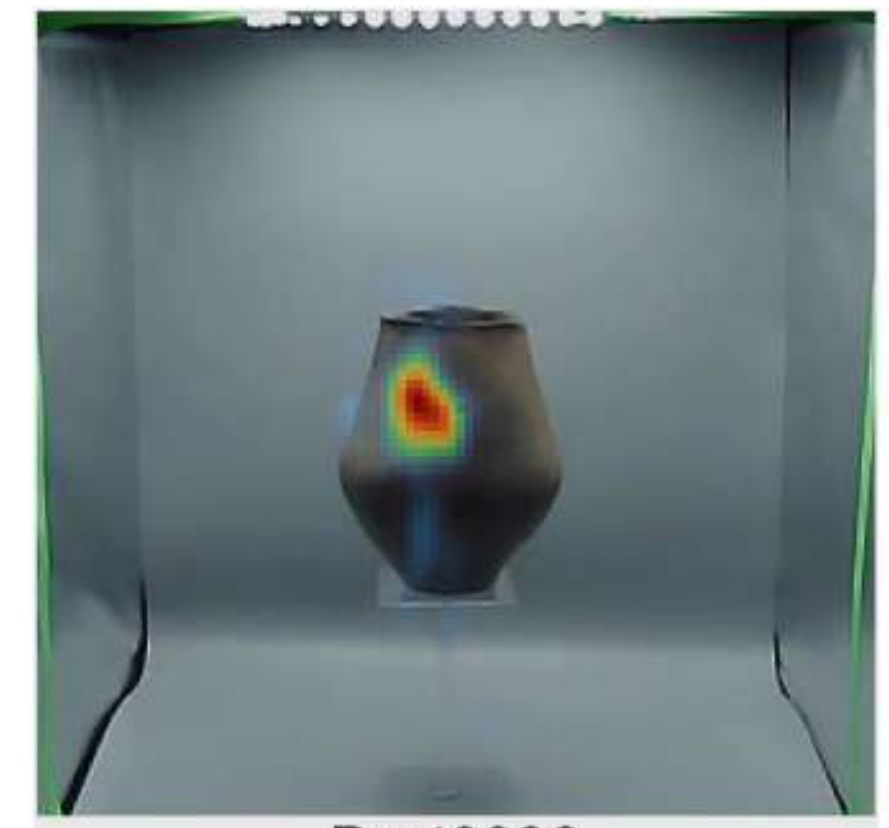
Part0093

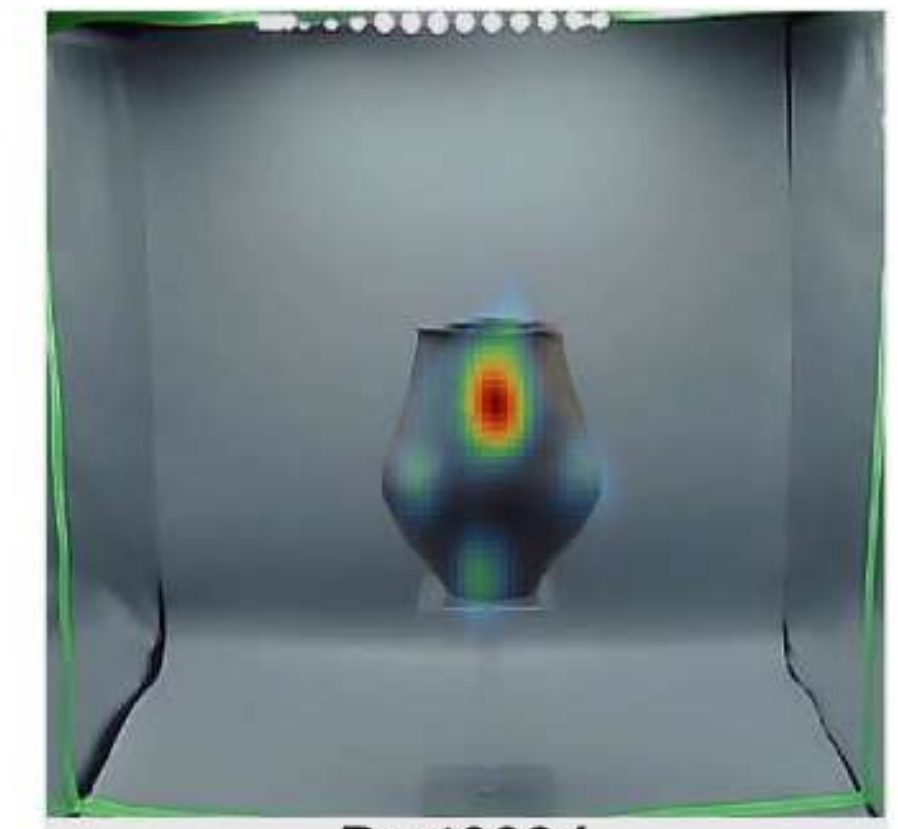
Part0094

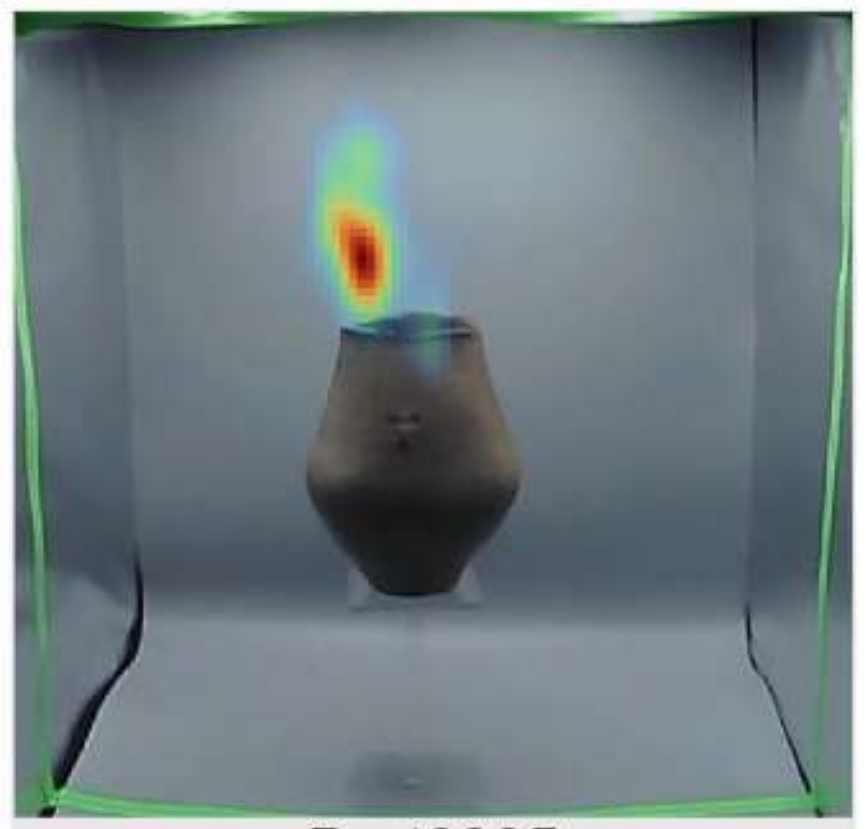
Part0095

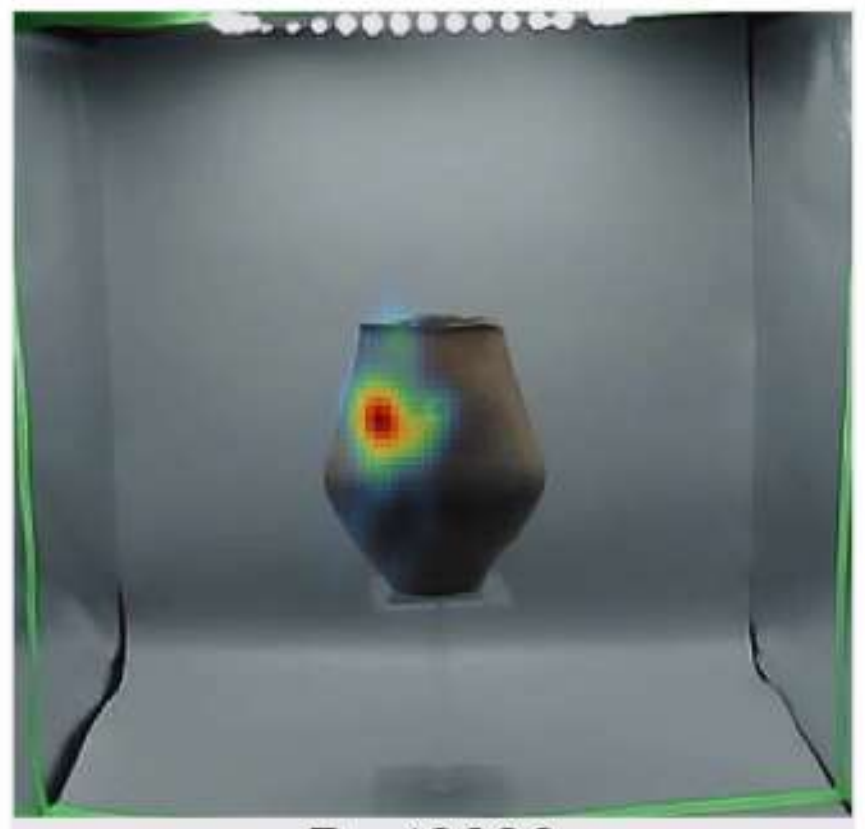
Part0096

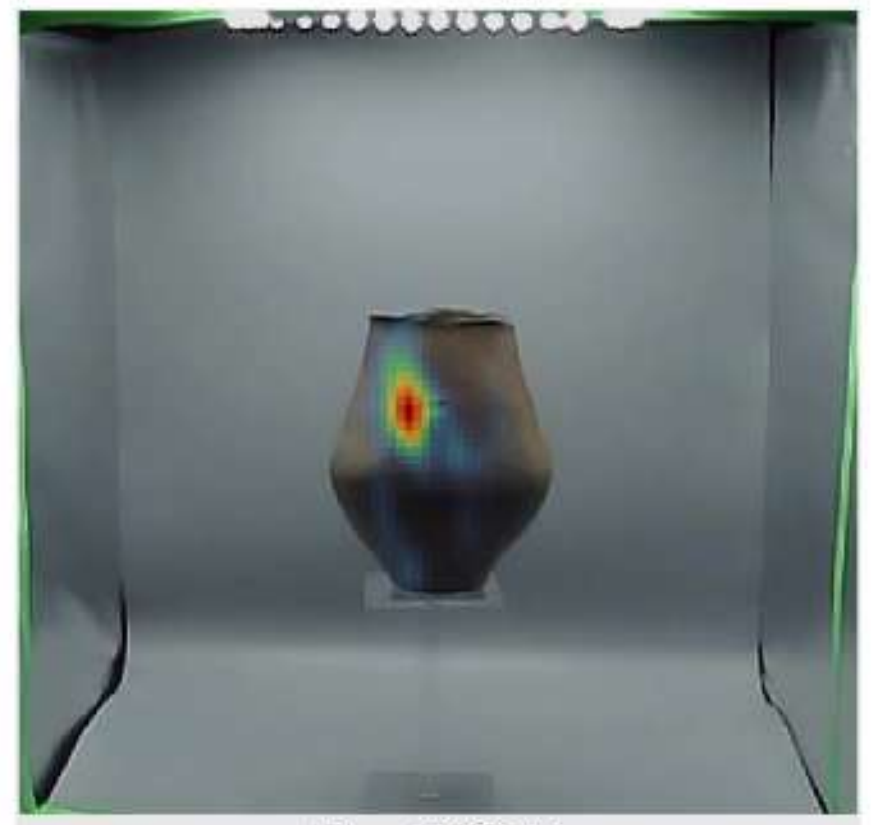
Part0097

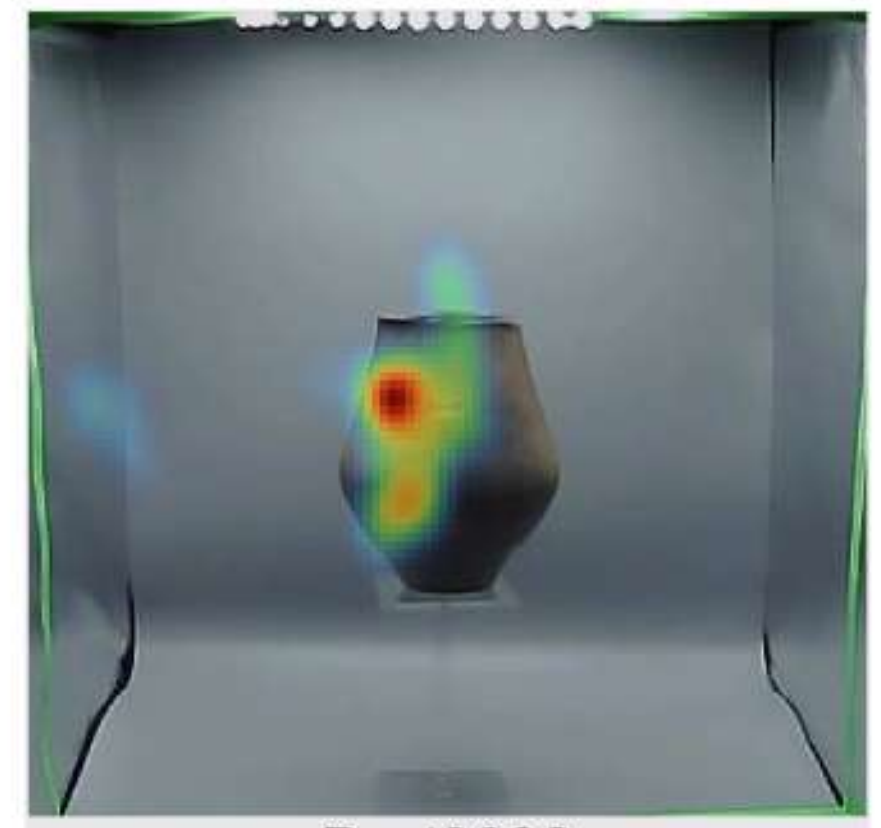
Part0098

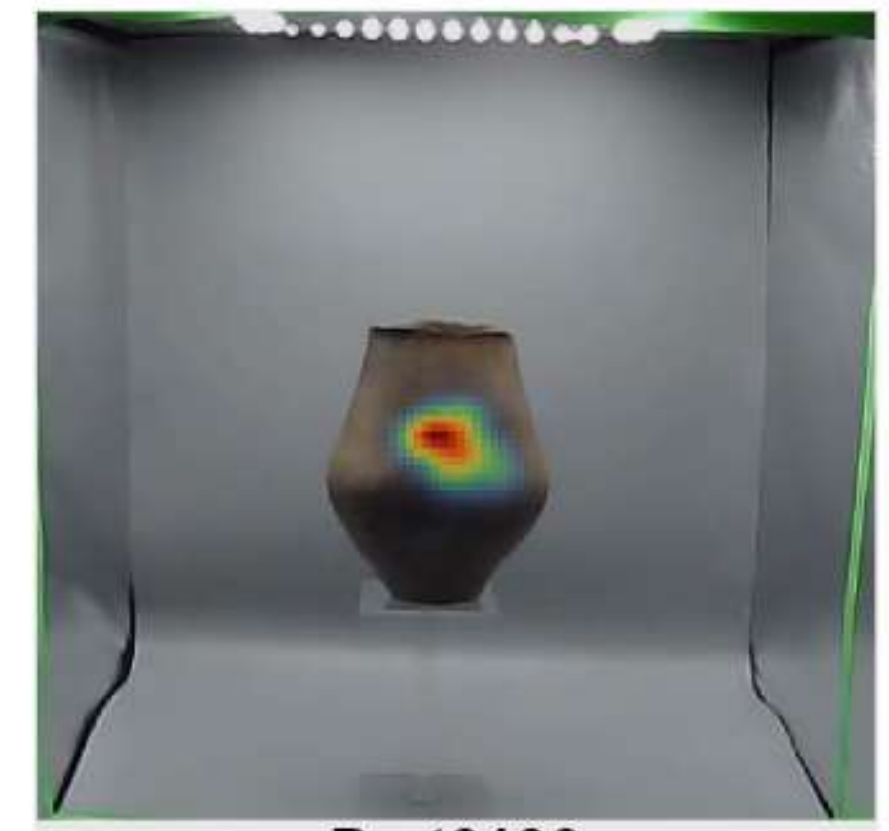
Part0100

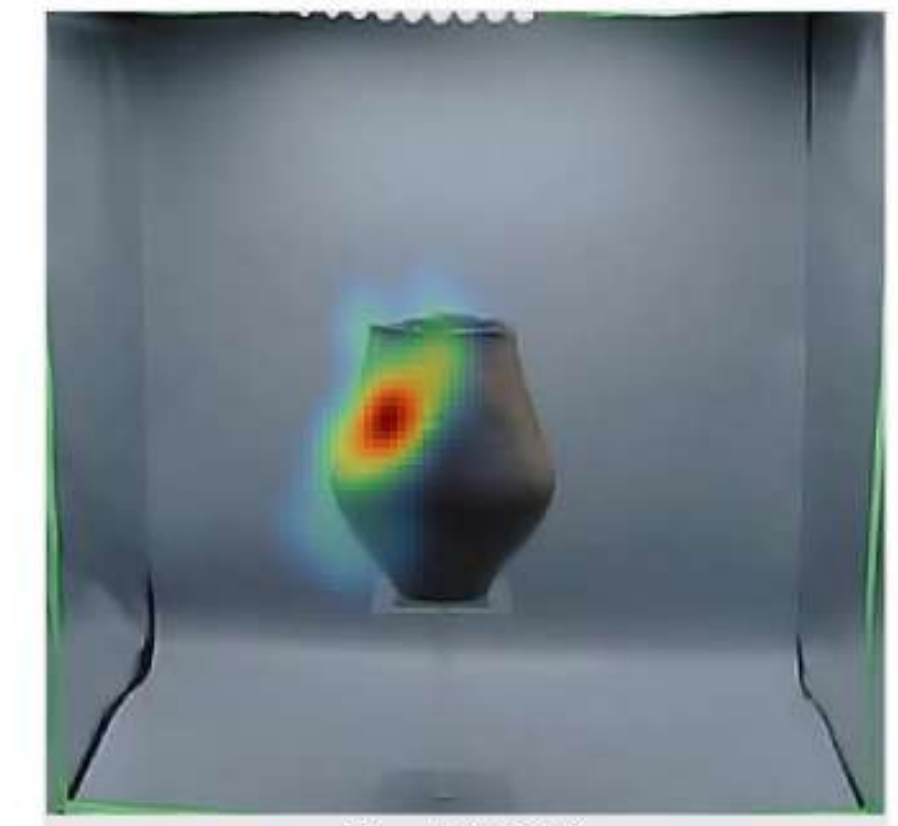
Part0101

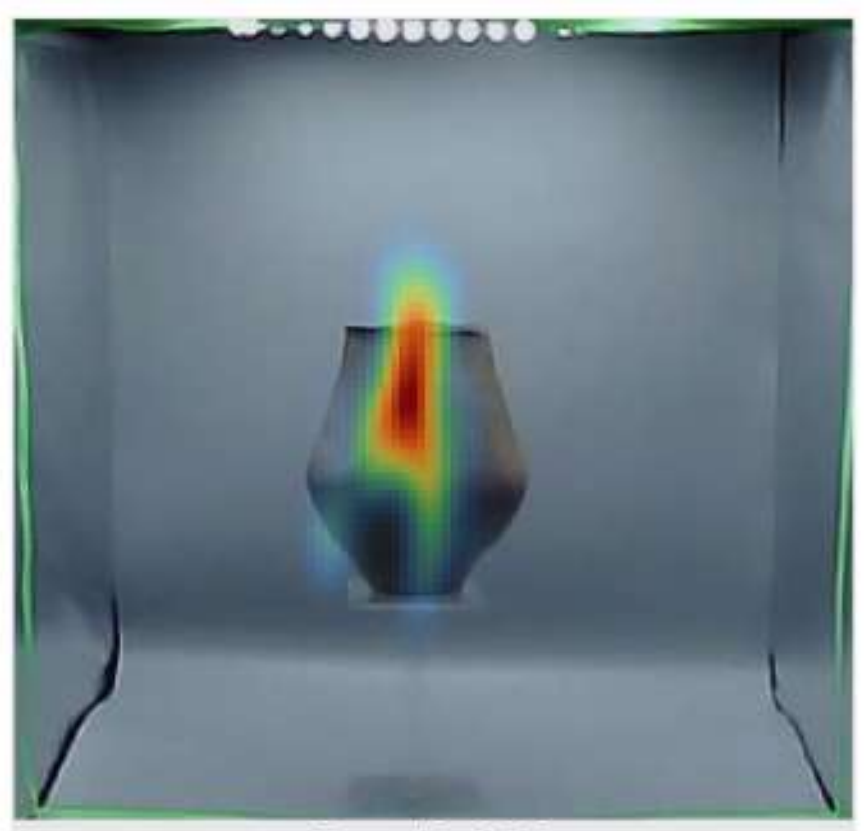
Part0102

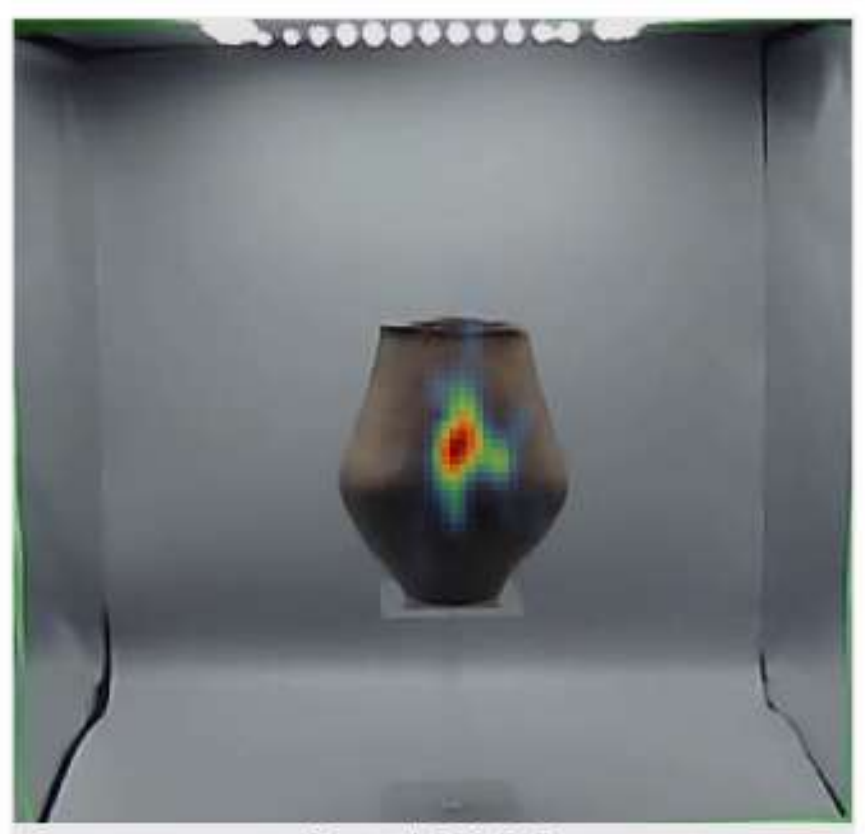
Part0103

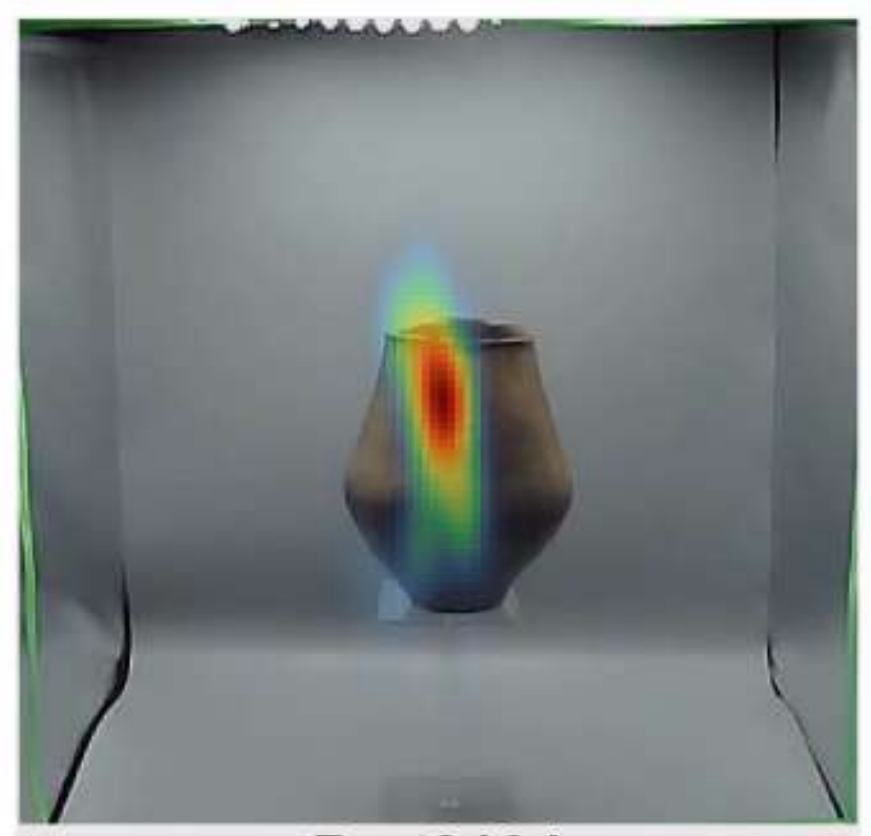
Part0104

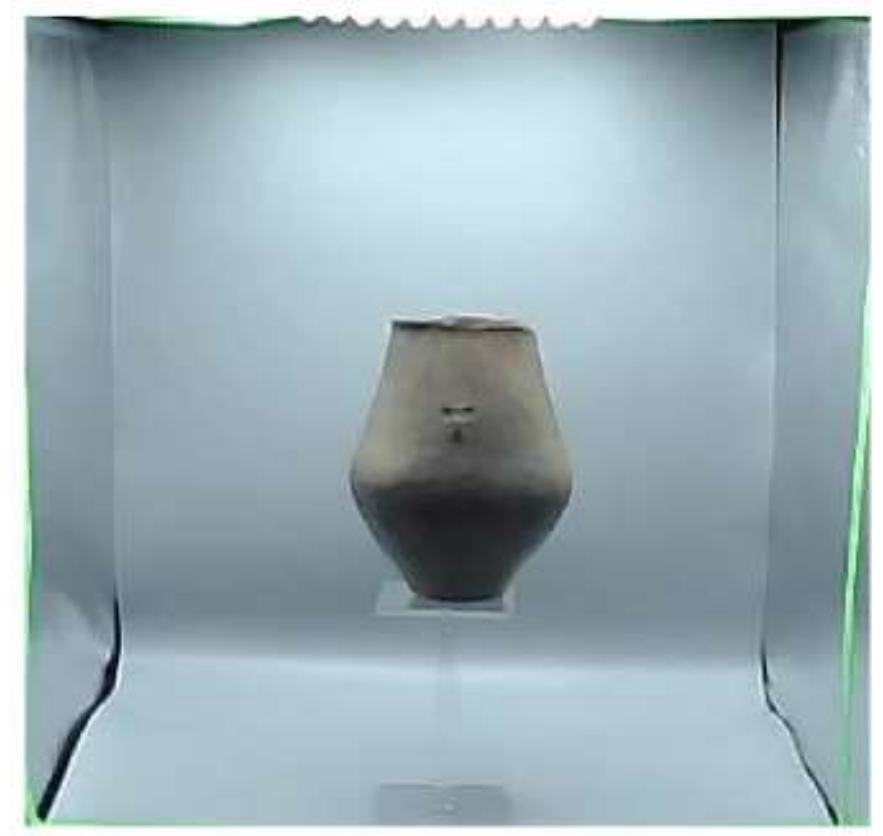
Part0105

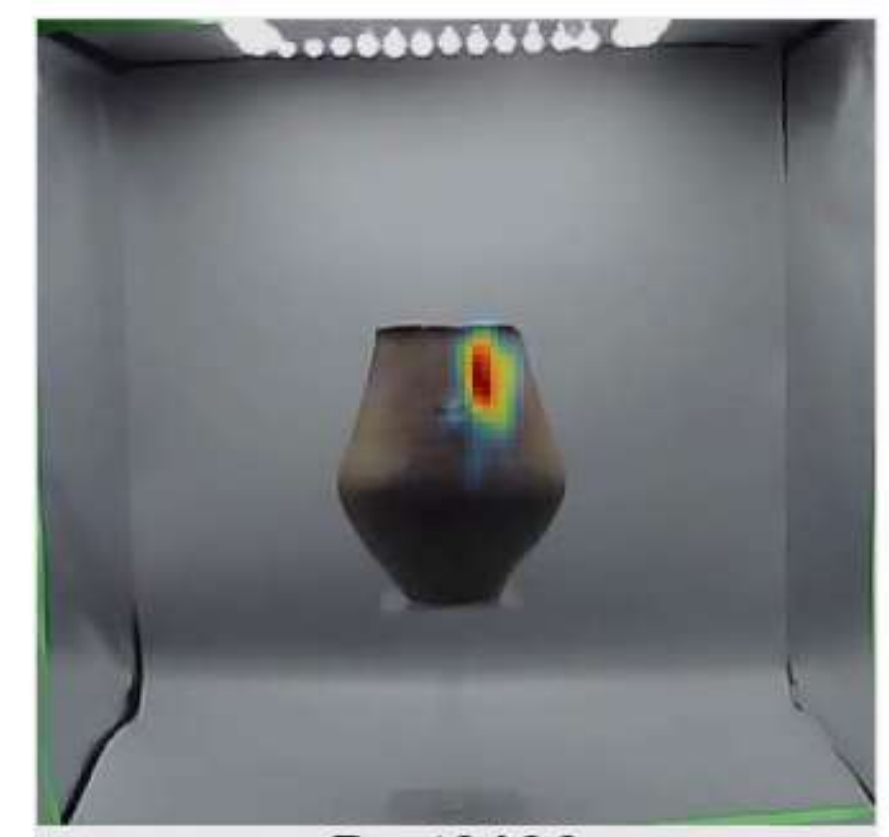
Part0106

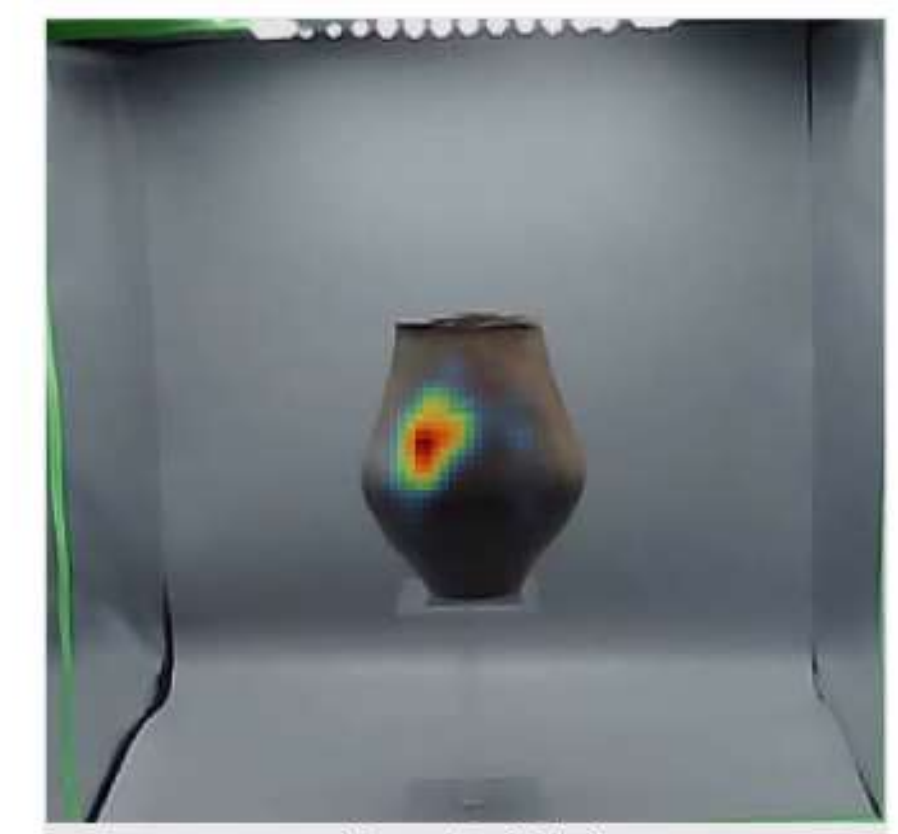
Part0107

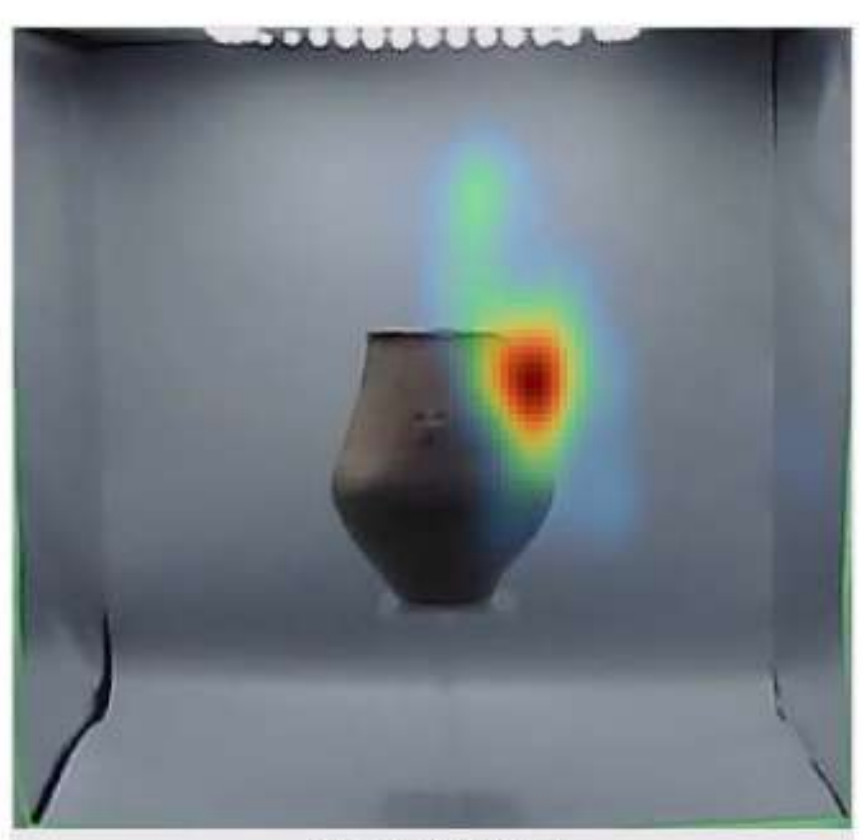
Part0108

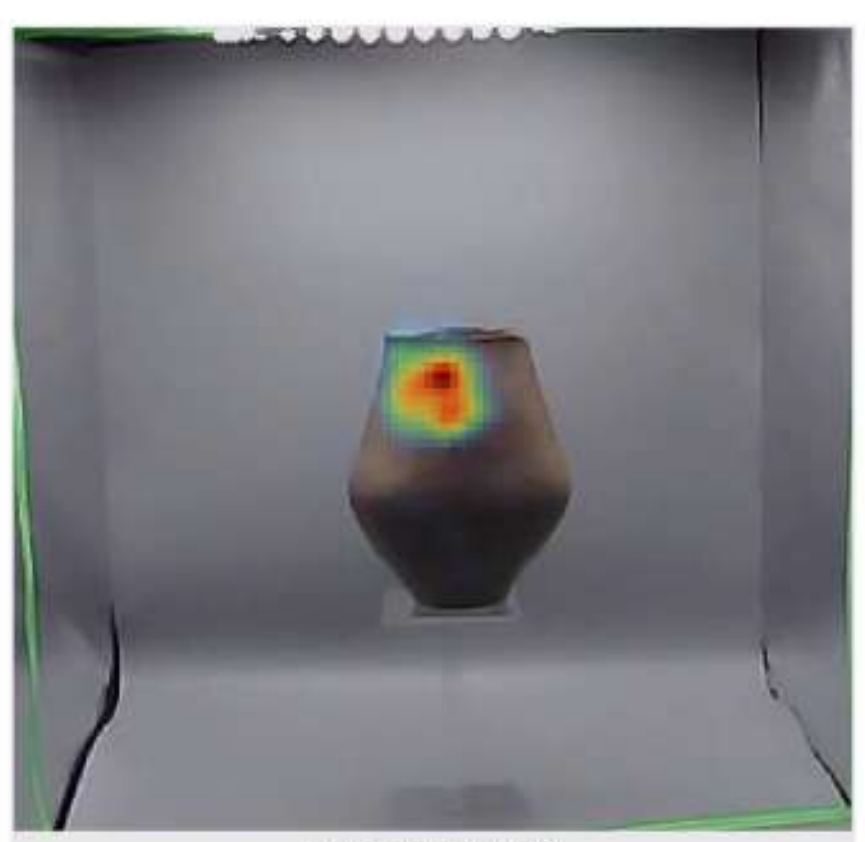
Part0109

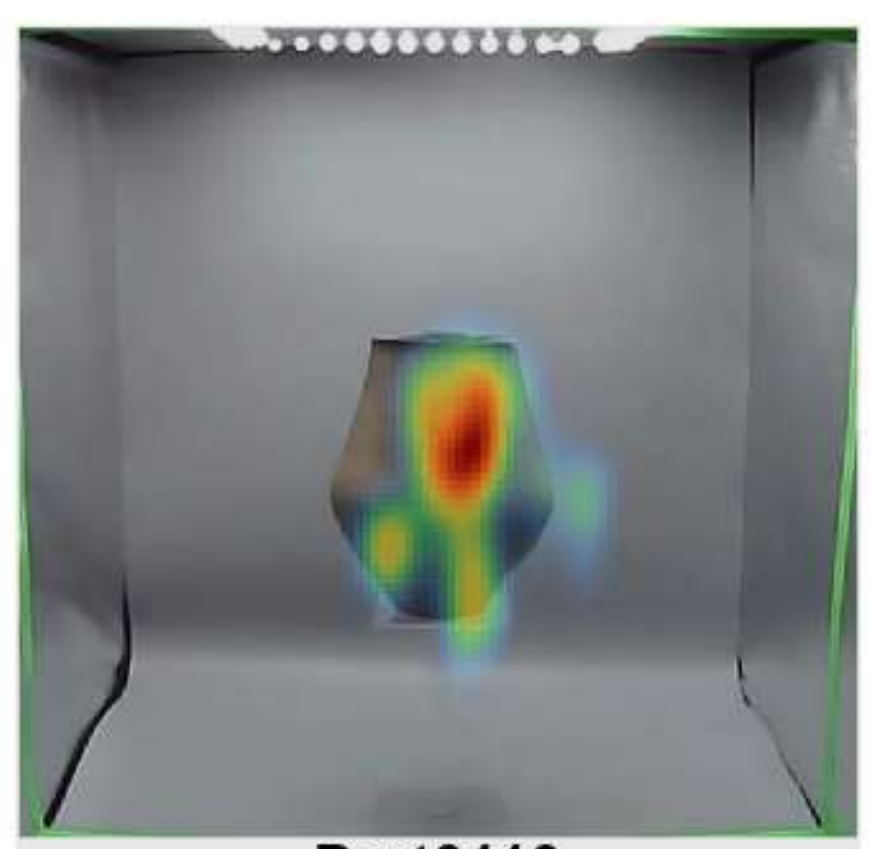
Part0110

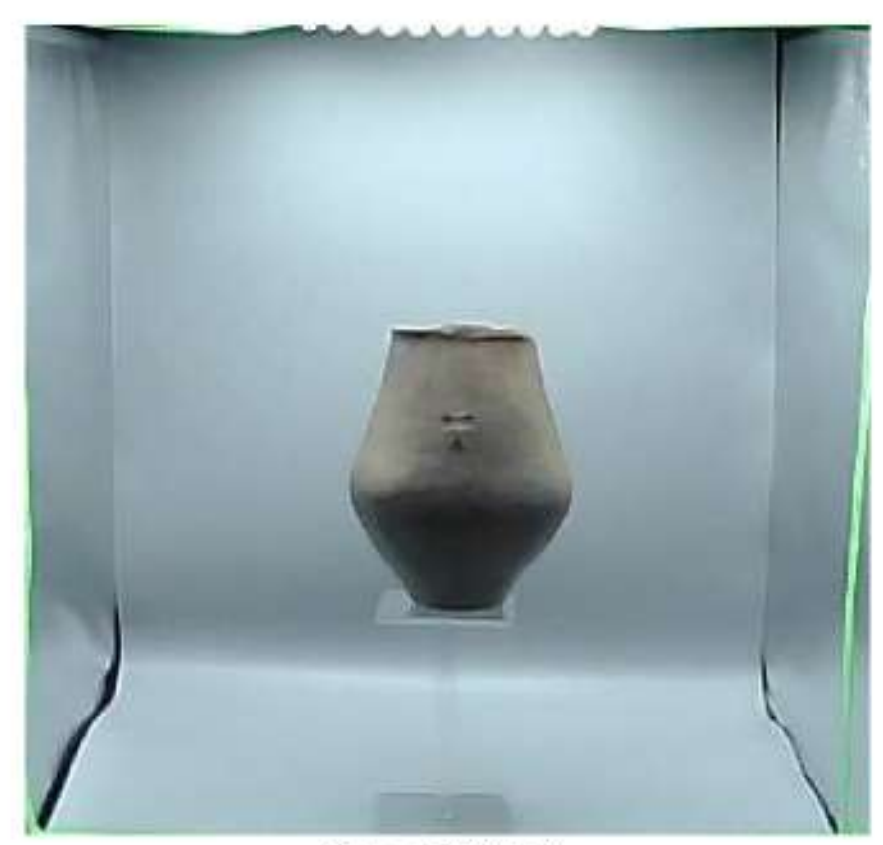
Part0111

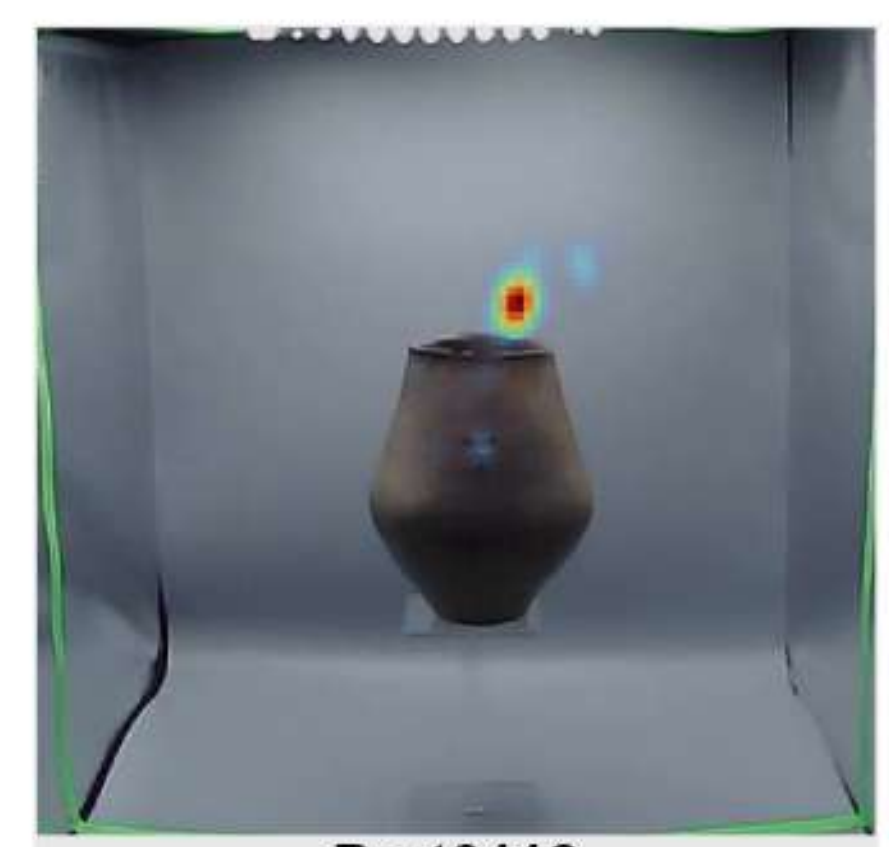
Part0112

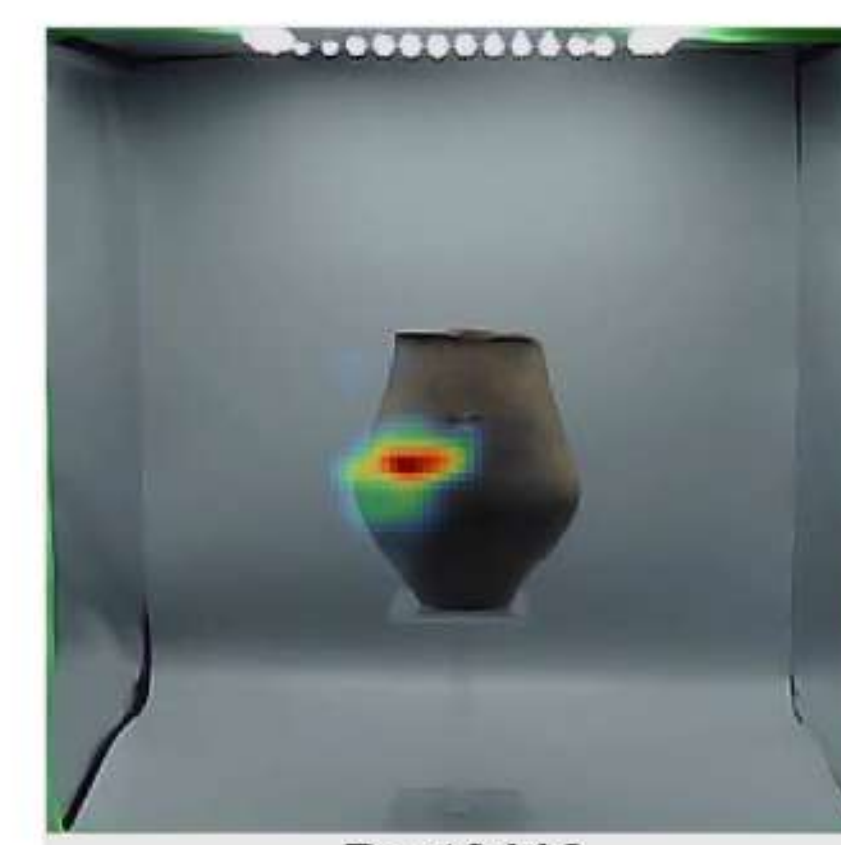
Part0113

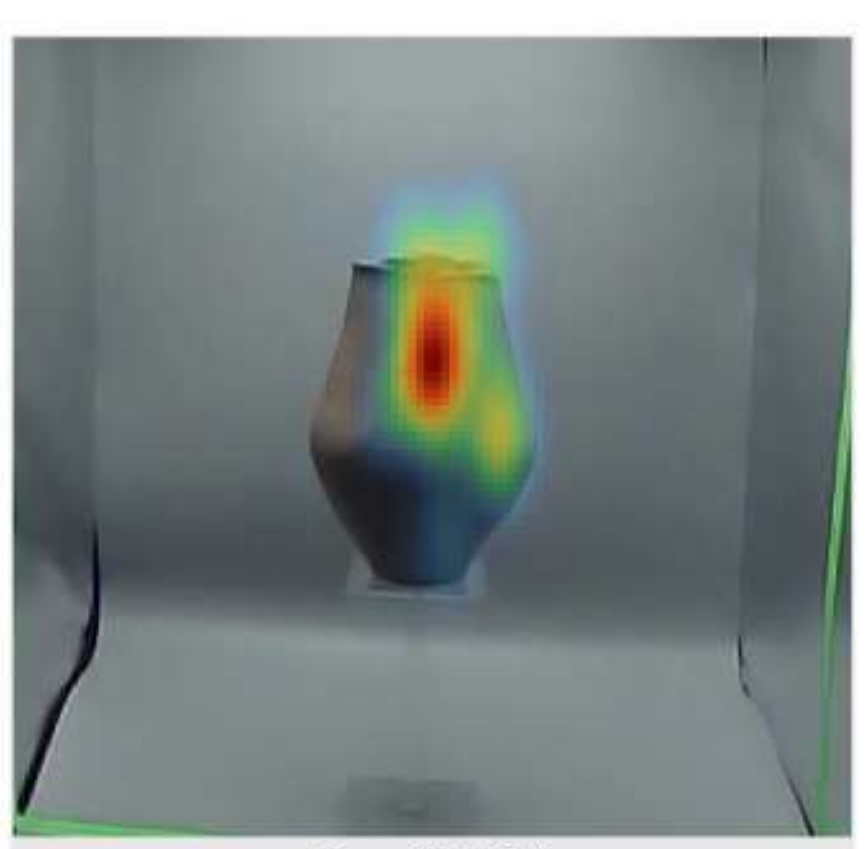
Part0114

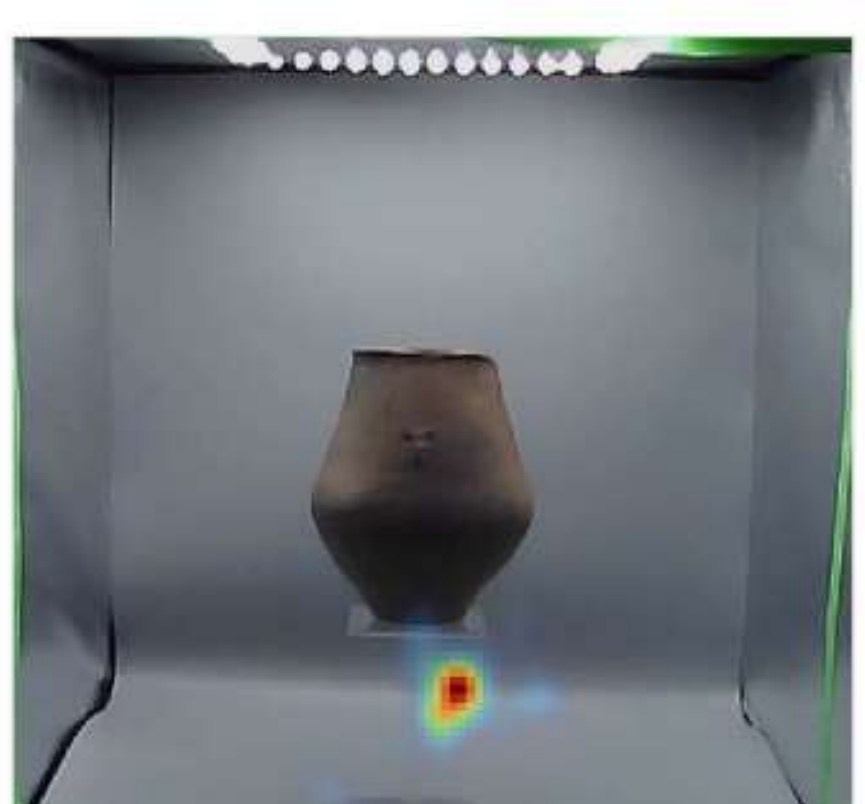
Part0115

Obj0244

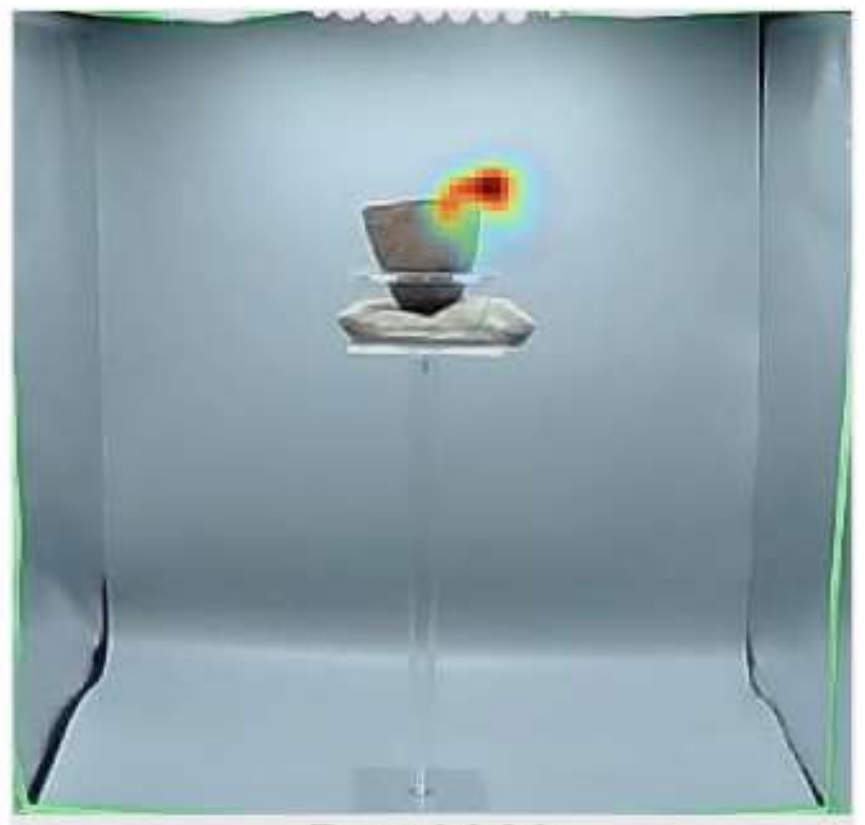
Part0089

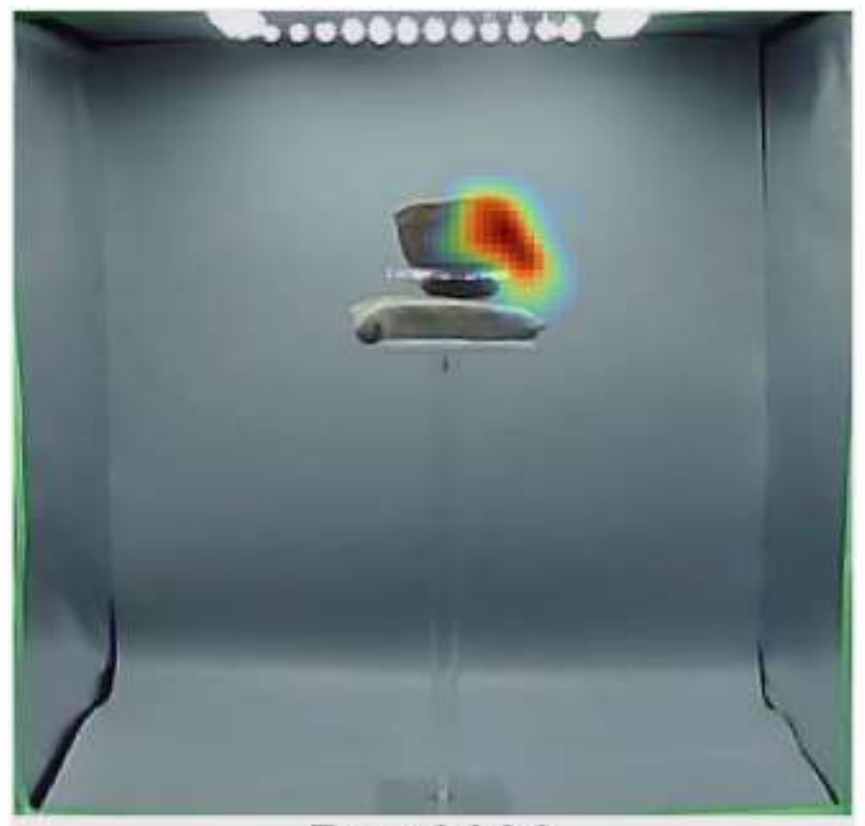
Part0090

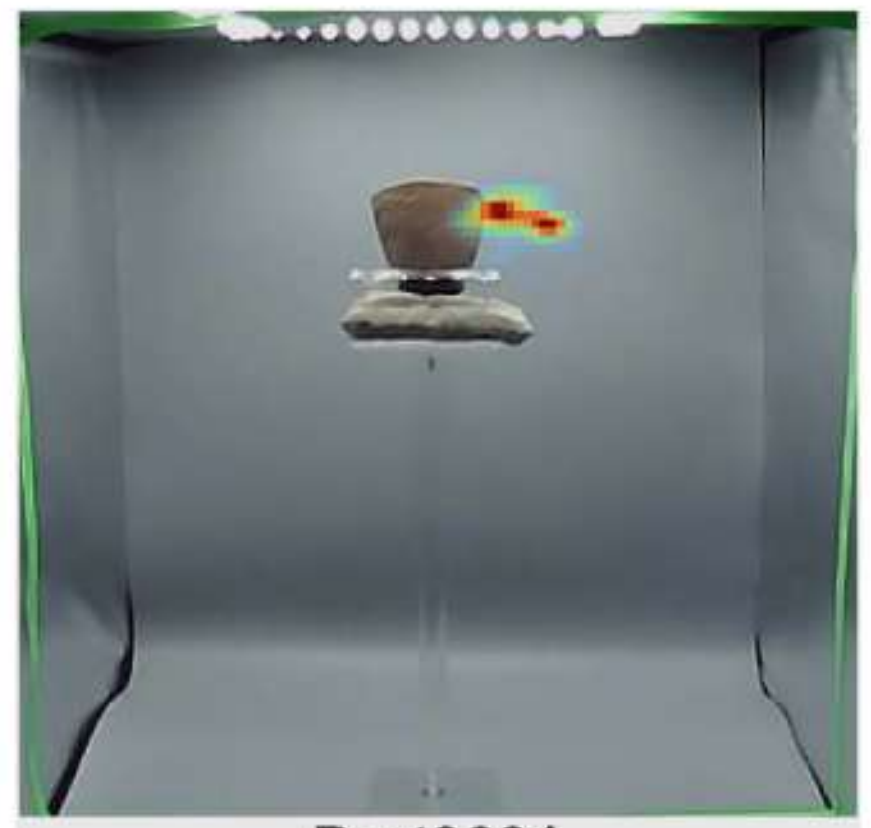
Part0091

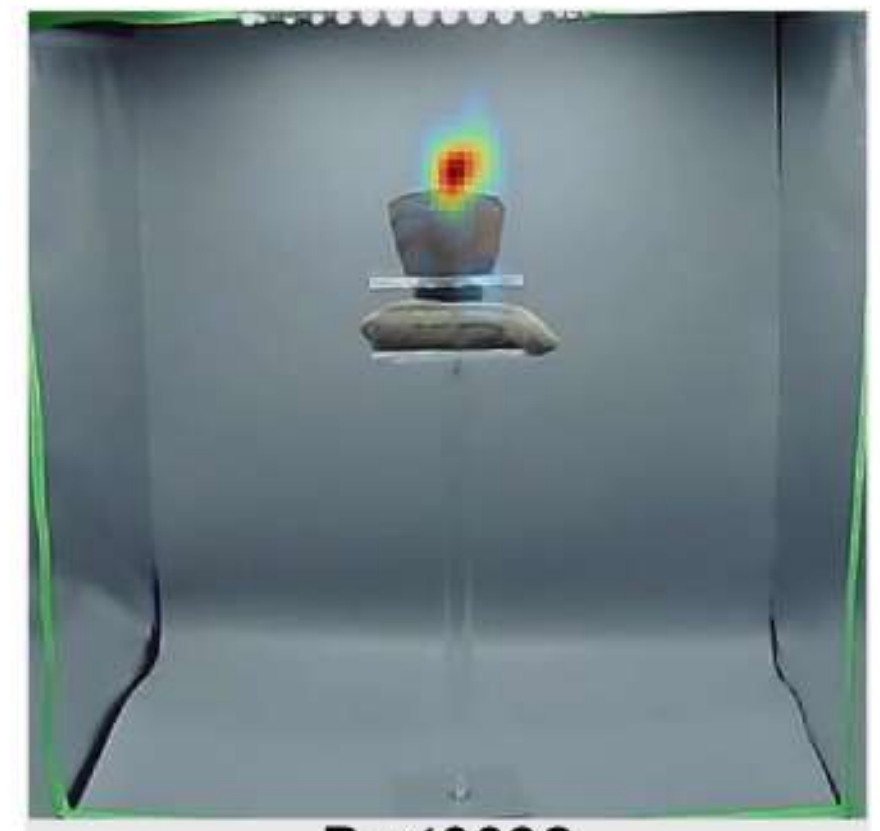
Part0092

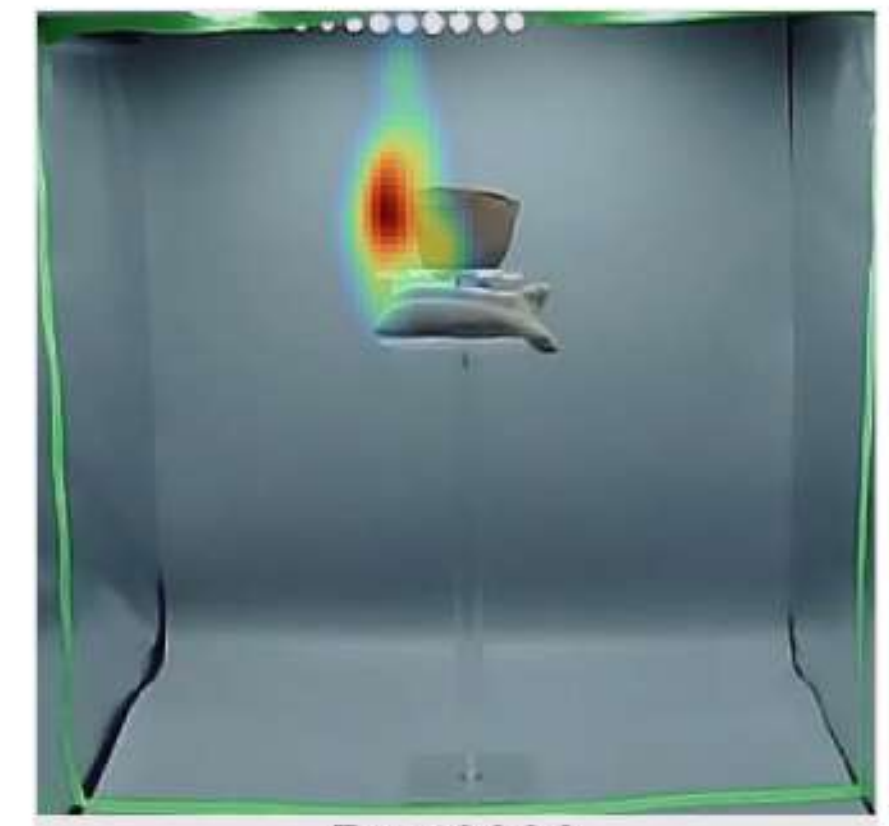
Part0093

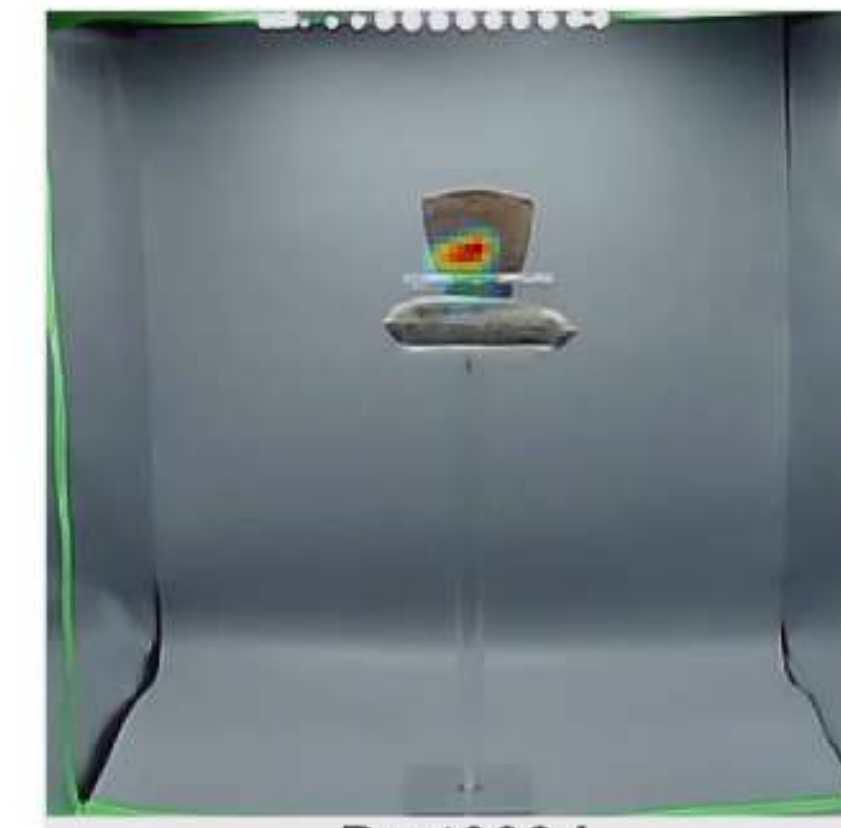
Part0094

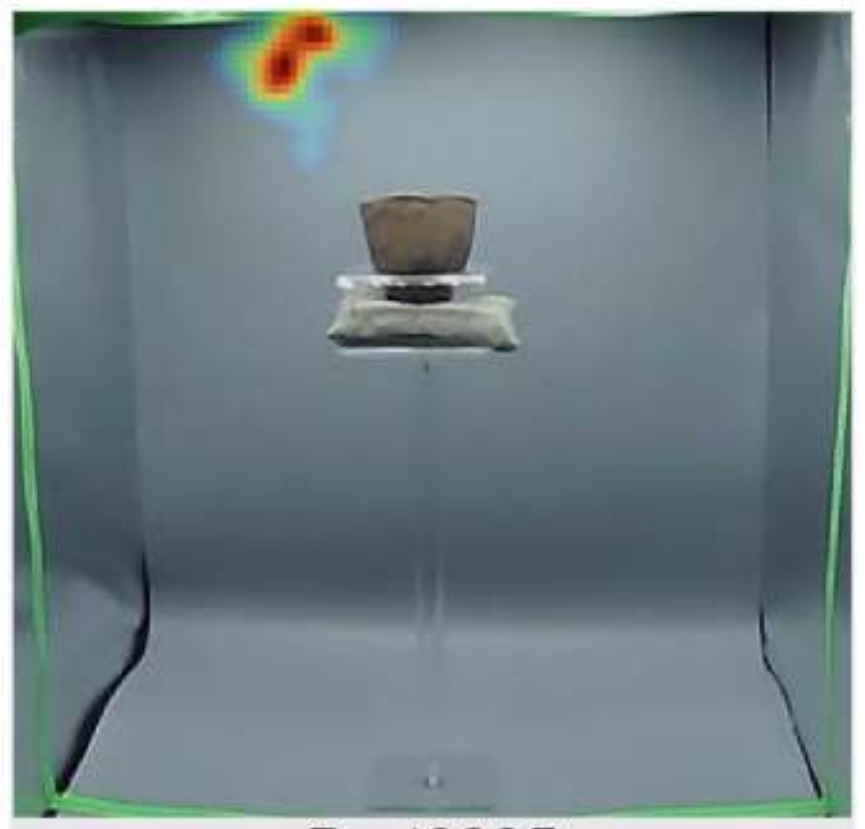
Part0095

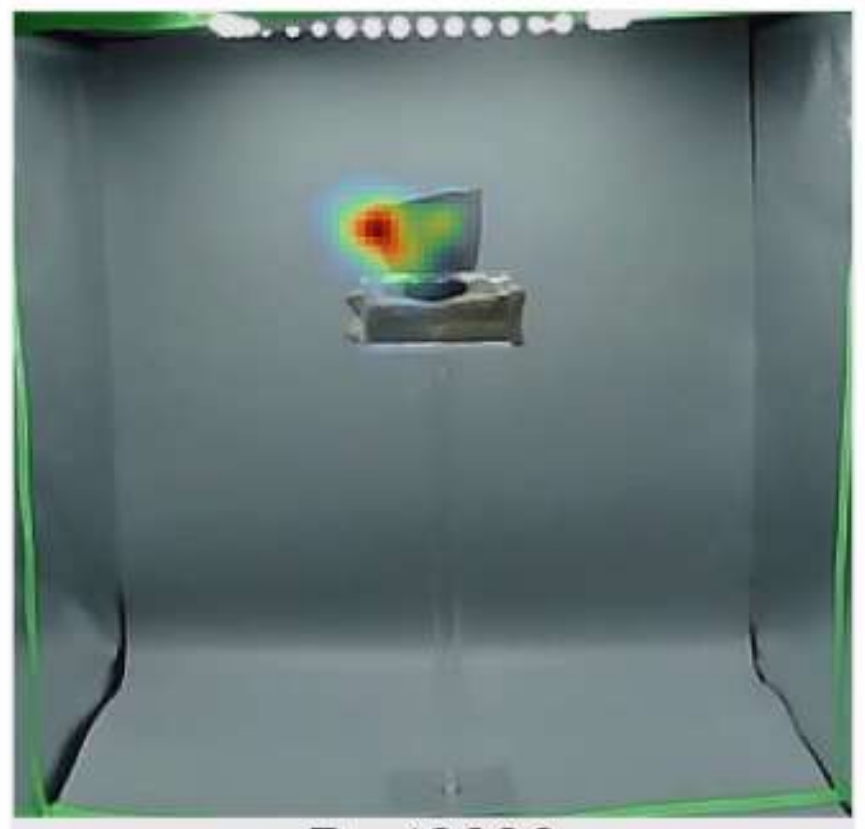
Part0096

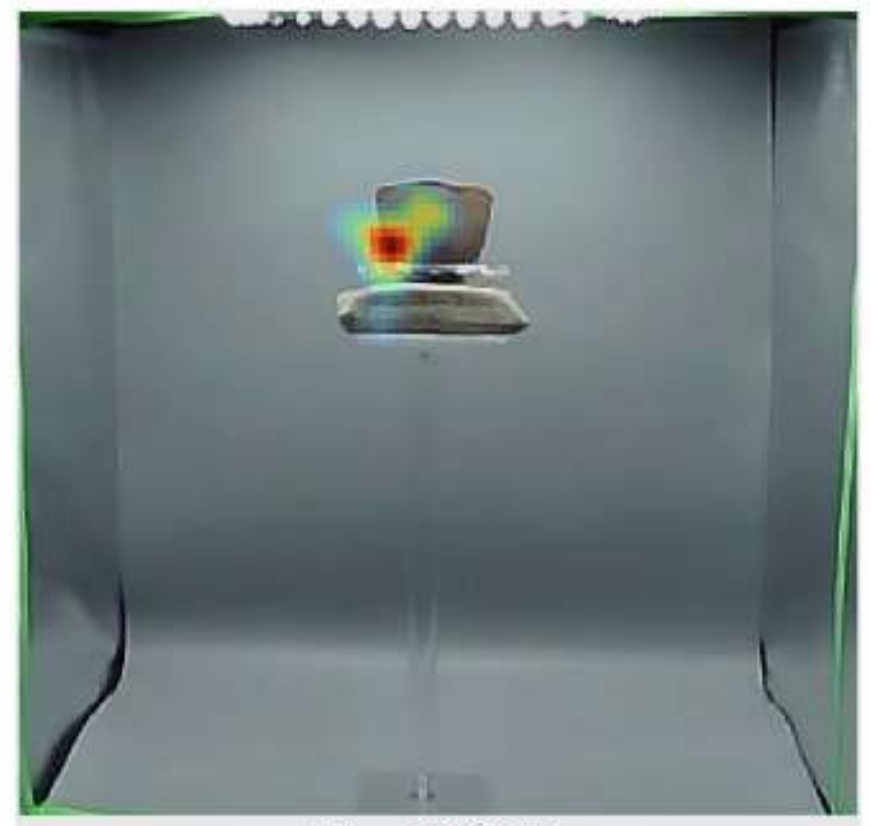
Part0097

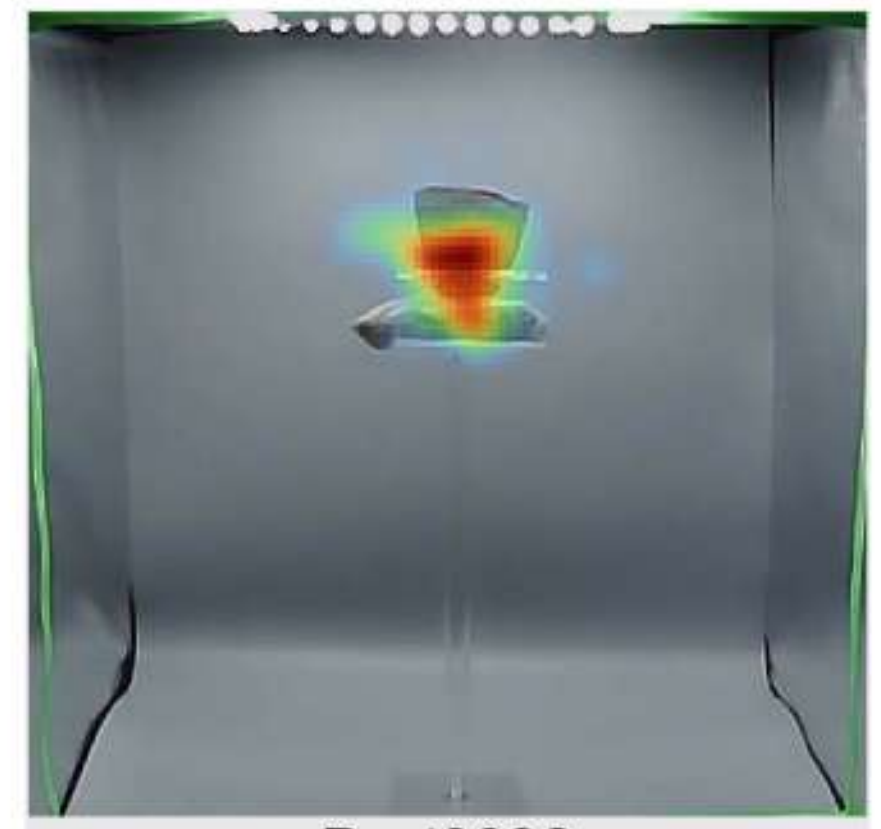
Part0098

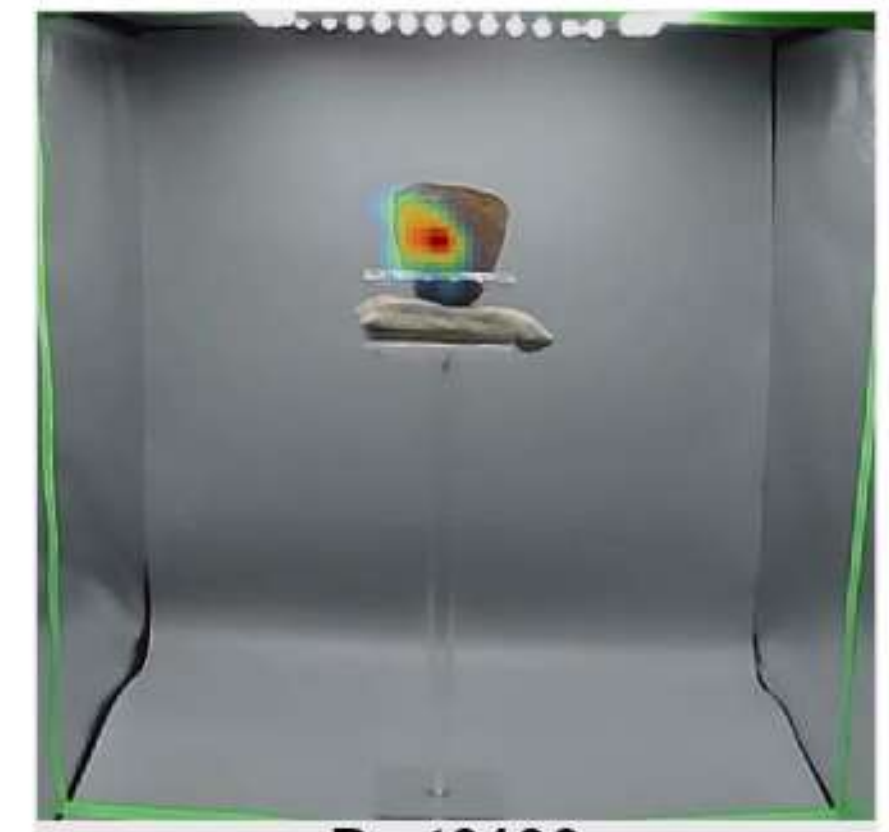
Part0100

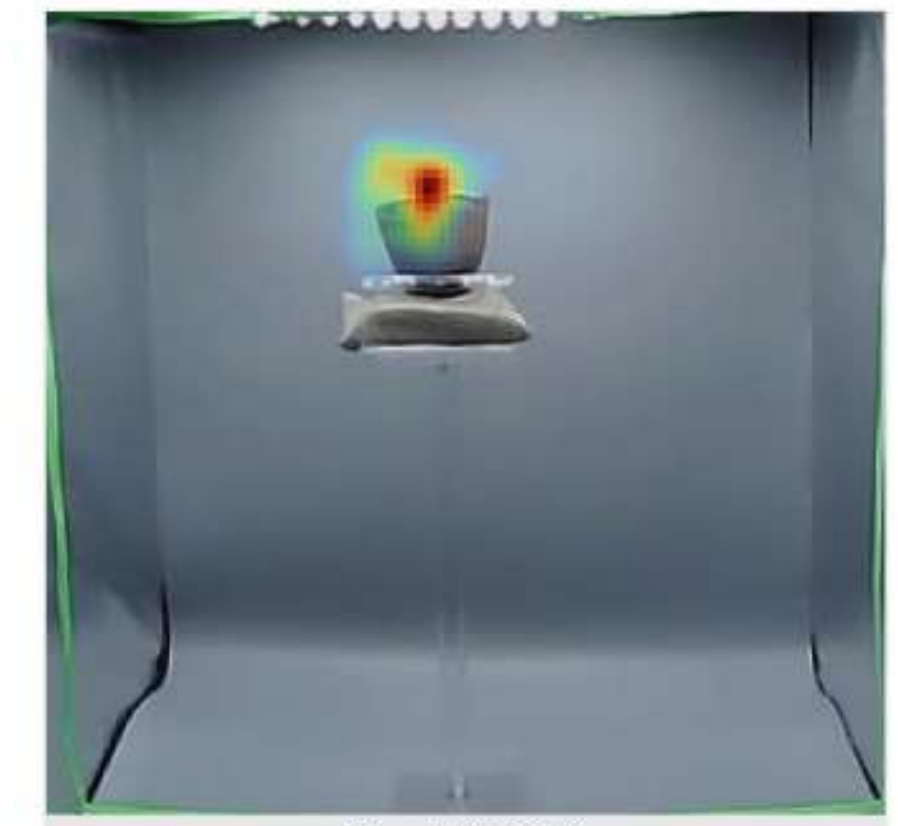
Part0101

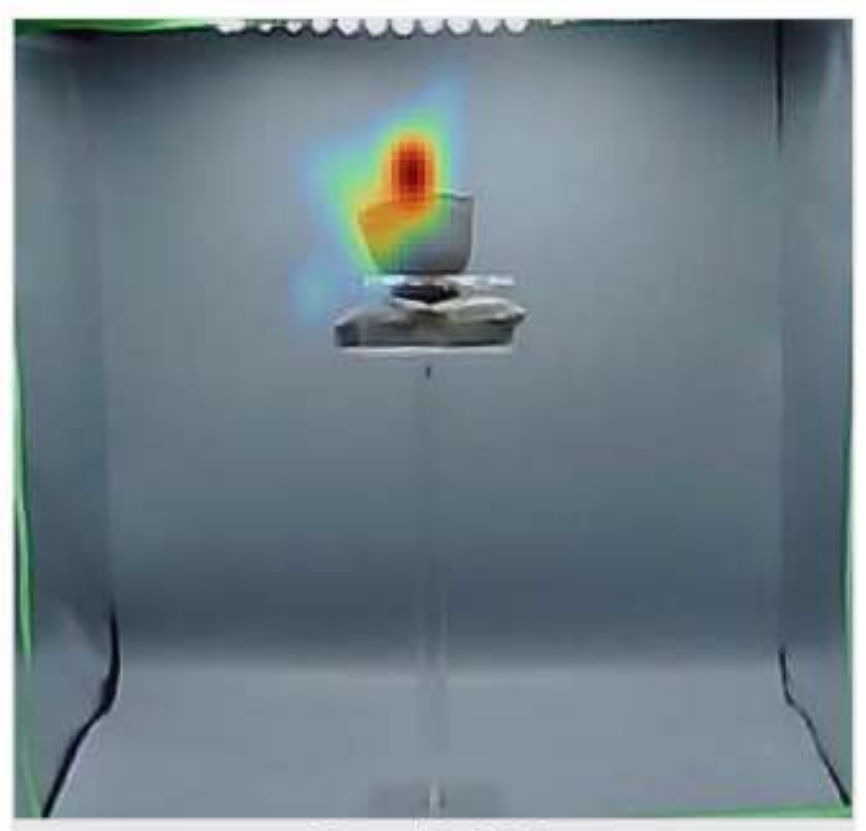
Part0102

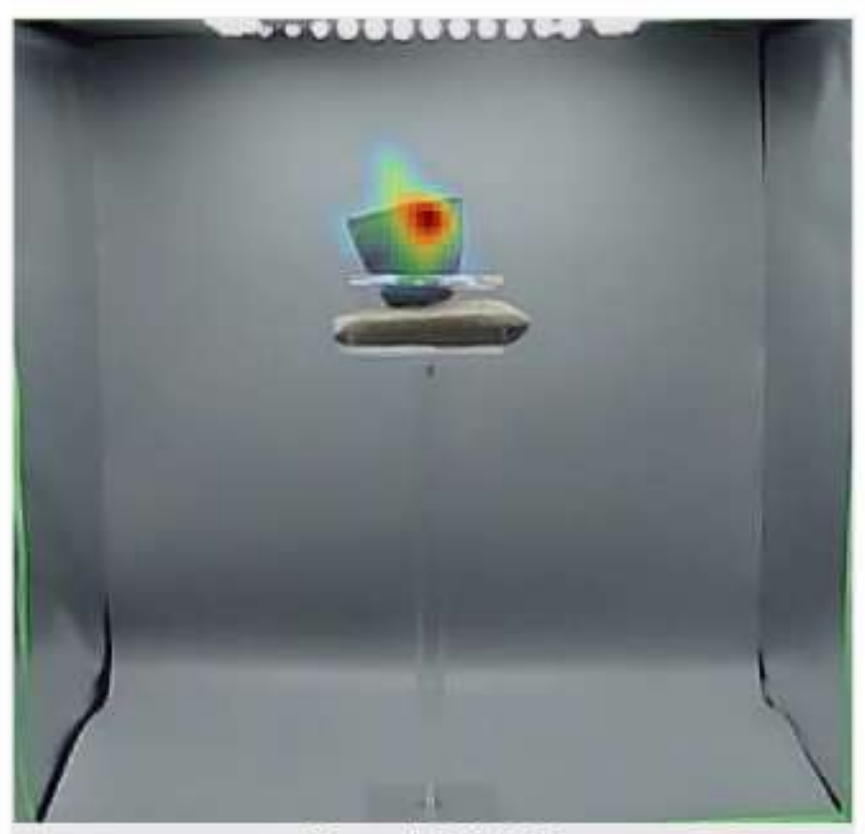
Part0103

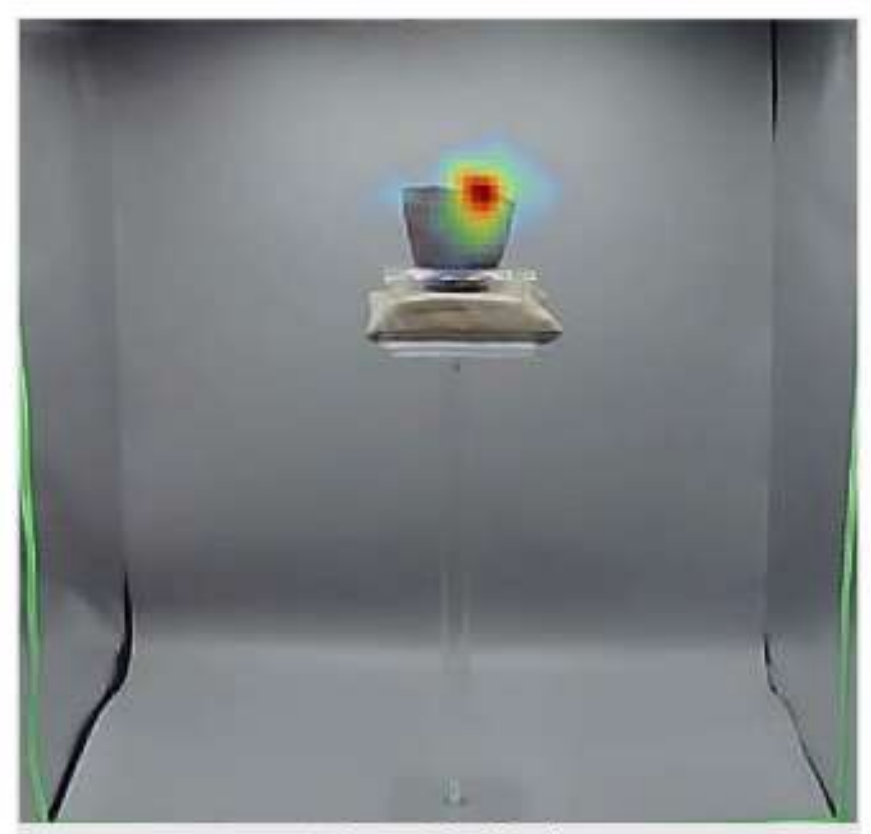
Part0104

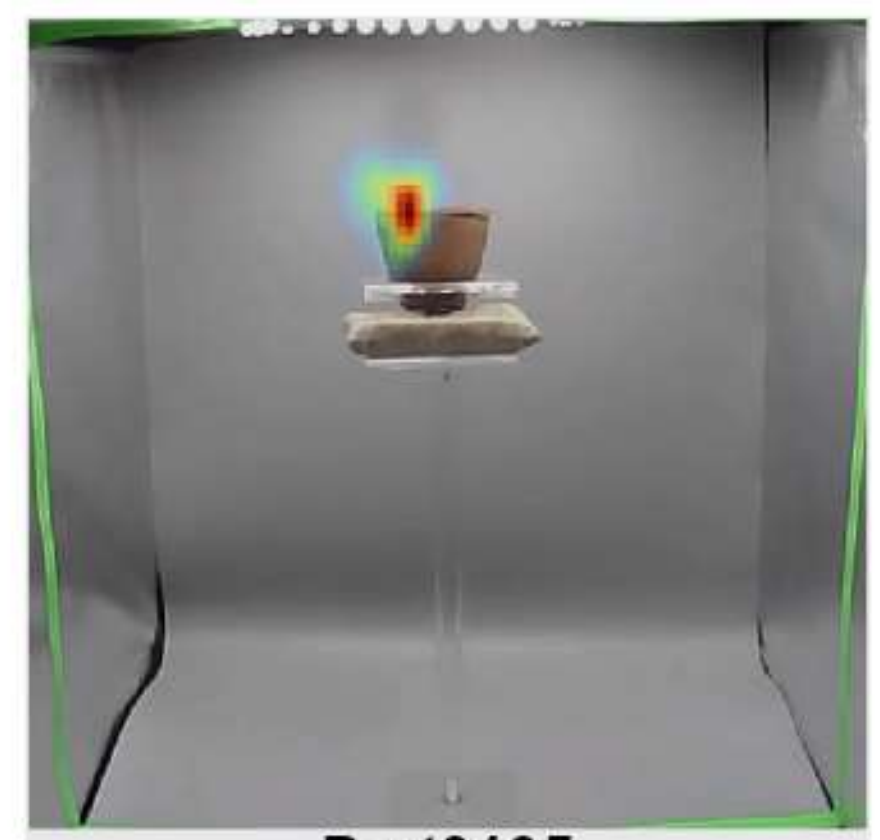
Part0105

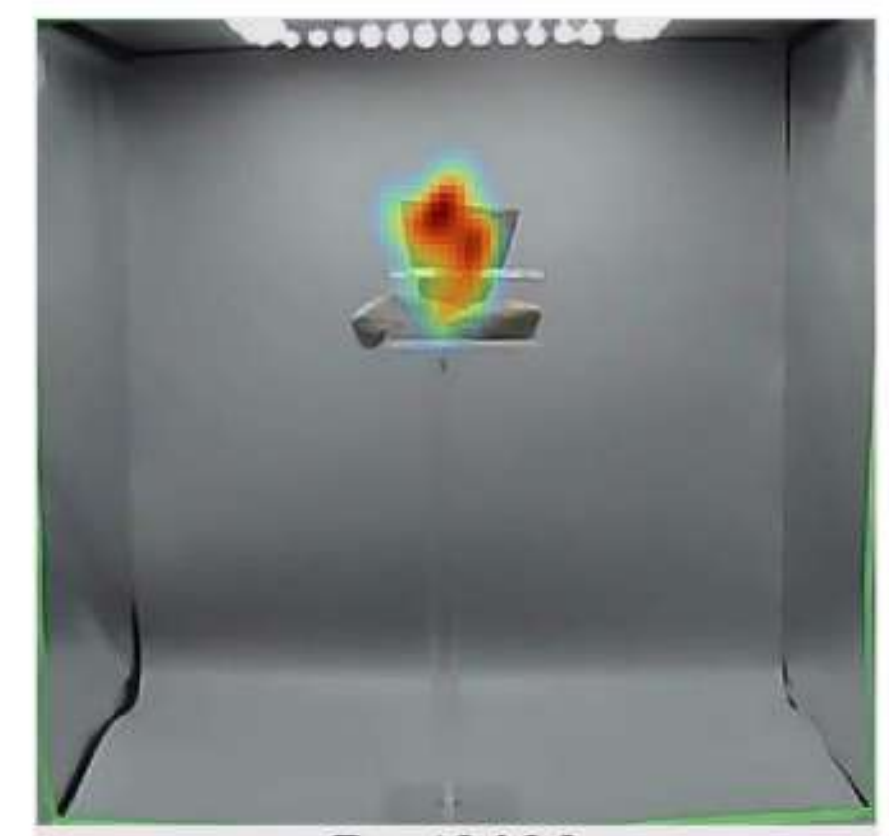
Part0106

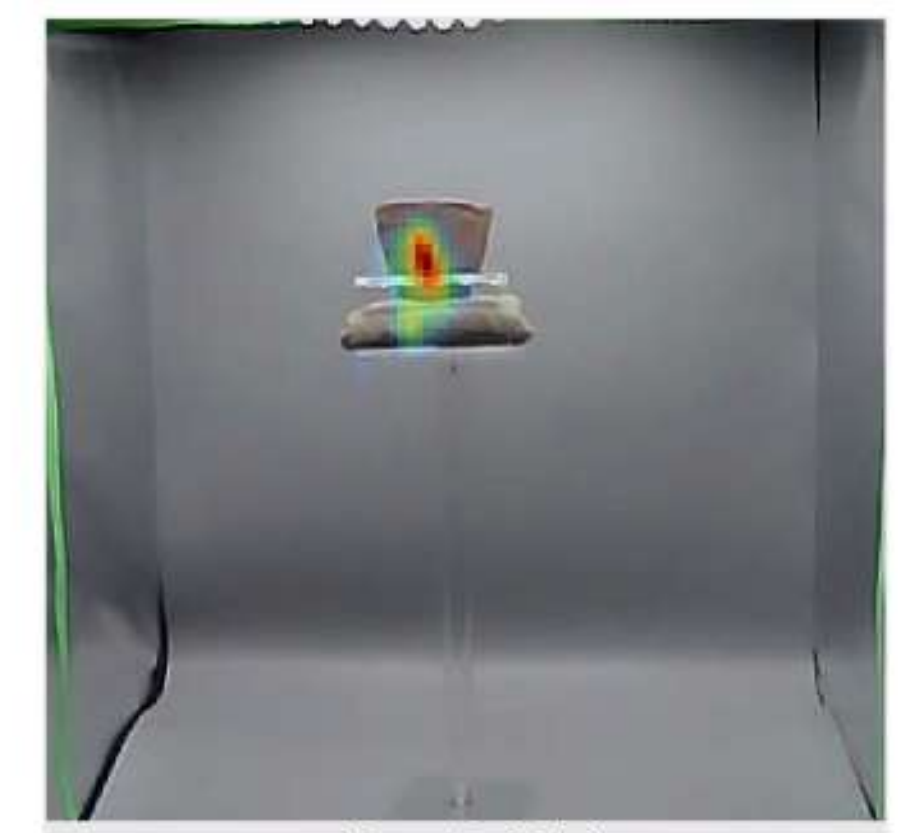
Part0107

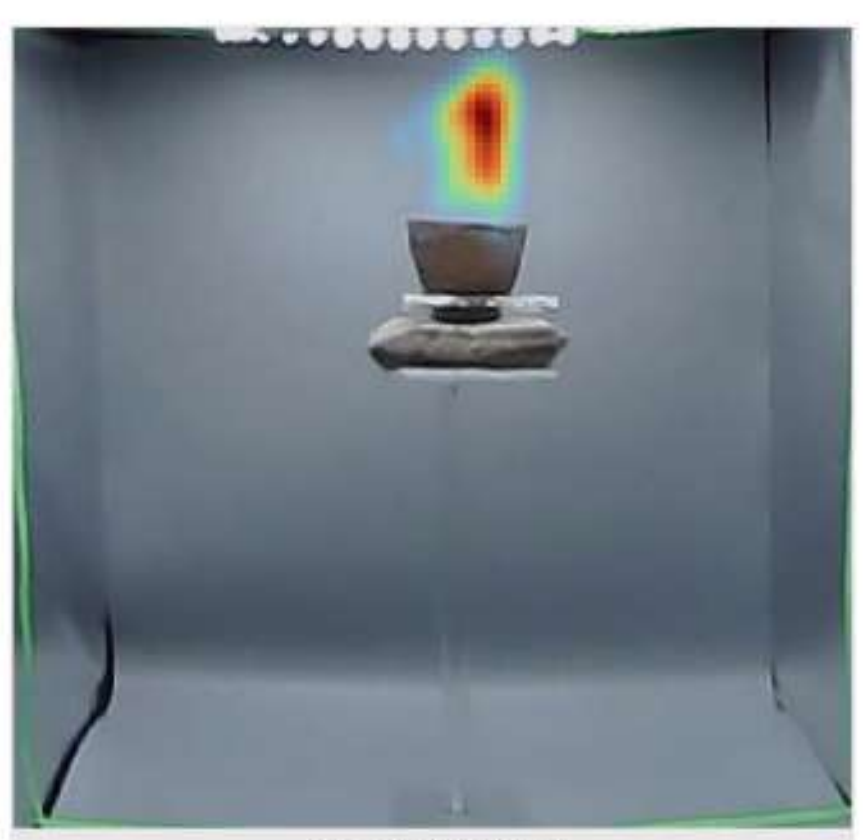
Part0108

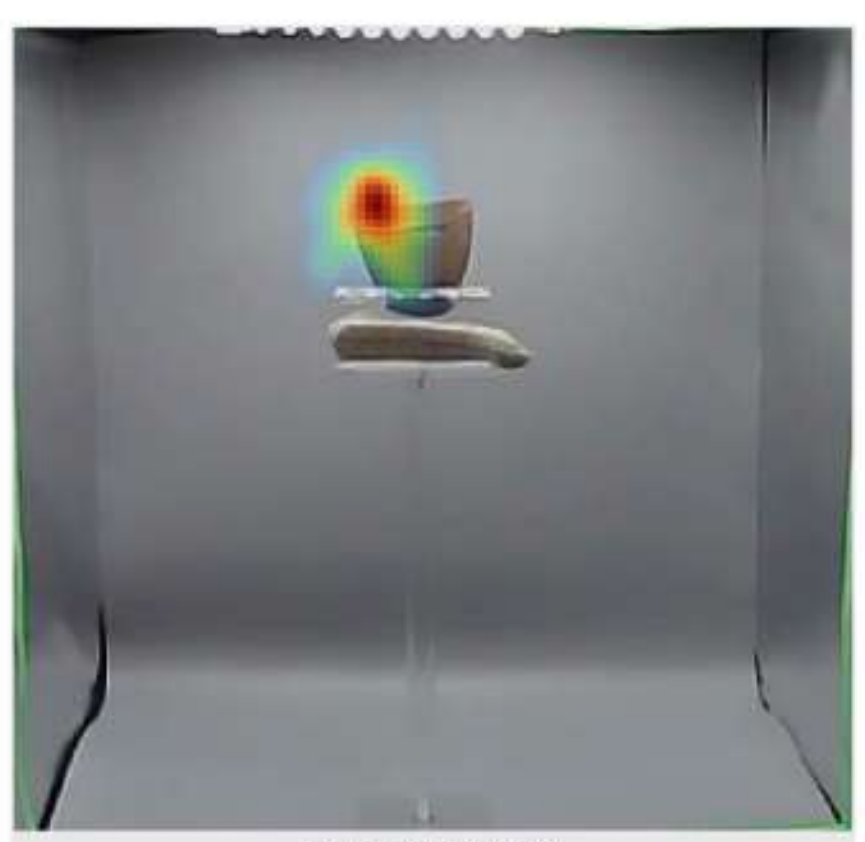
Part0109

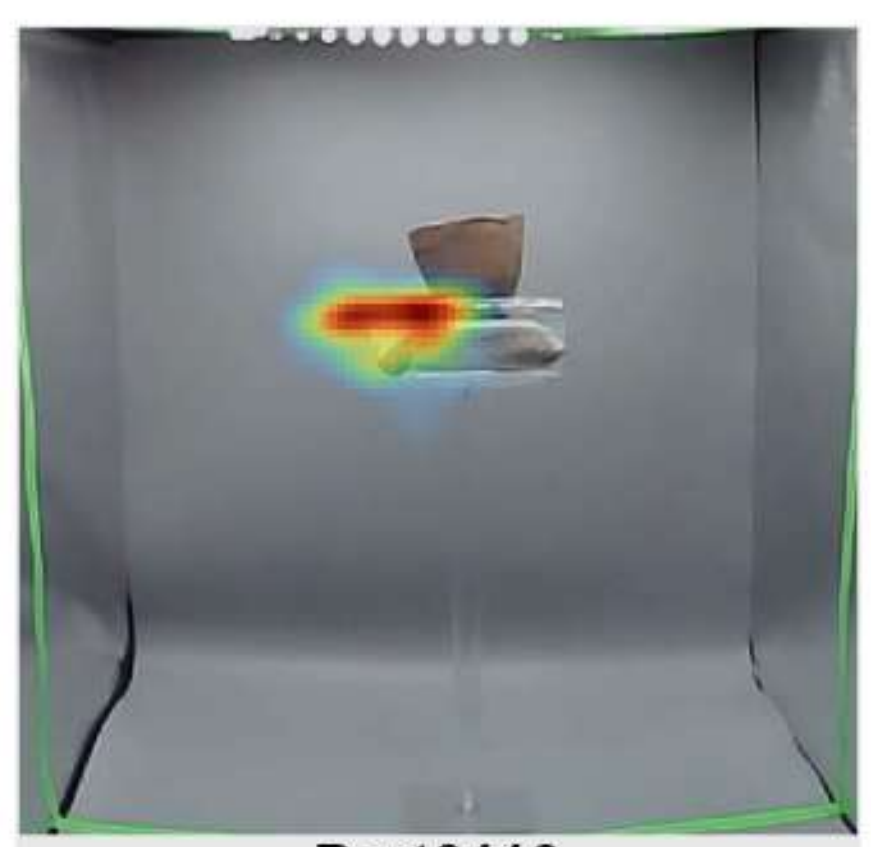
Part0110

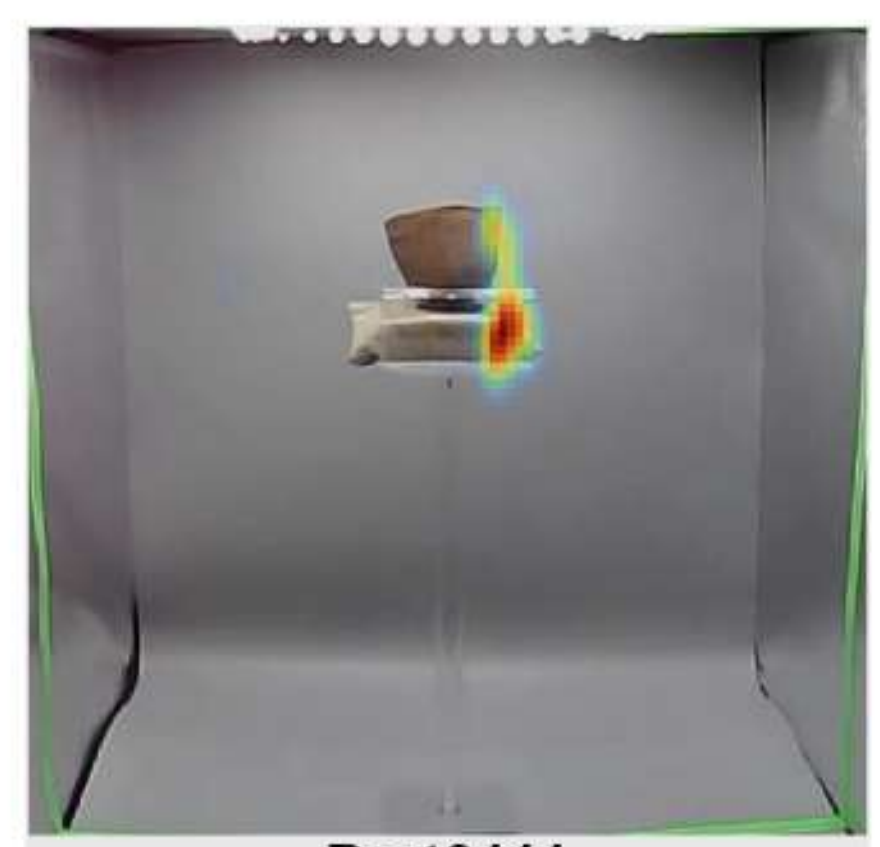
Part0111

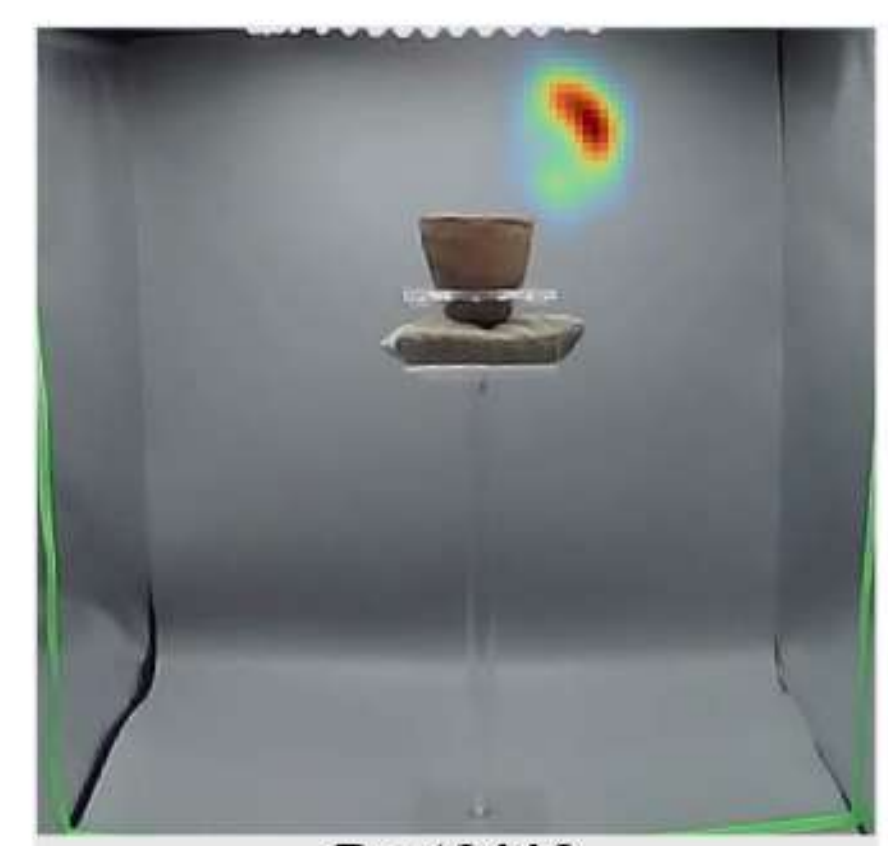
Part0112

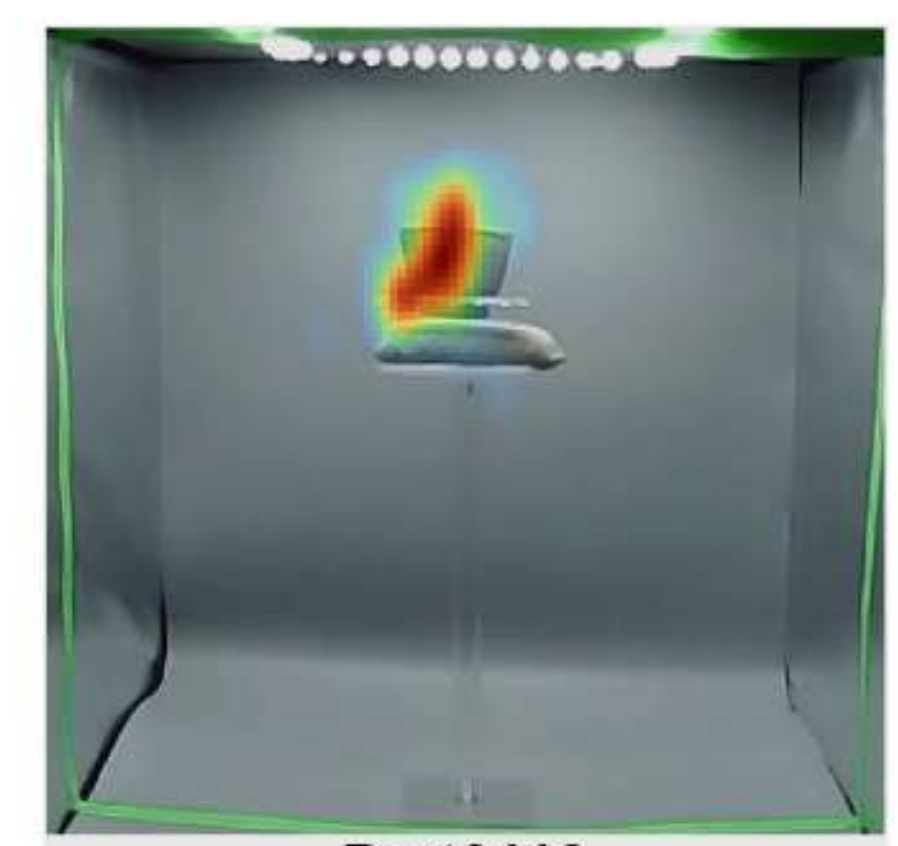
Part0113

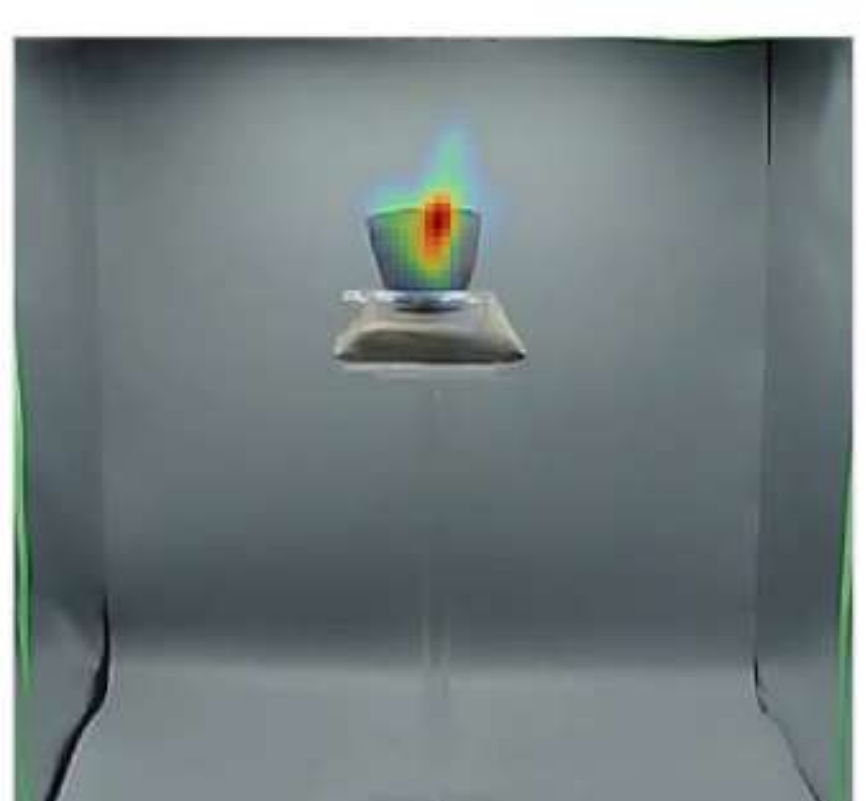
Part0114

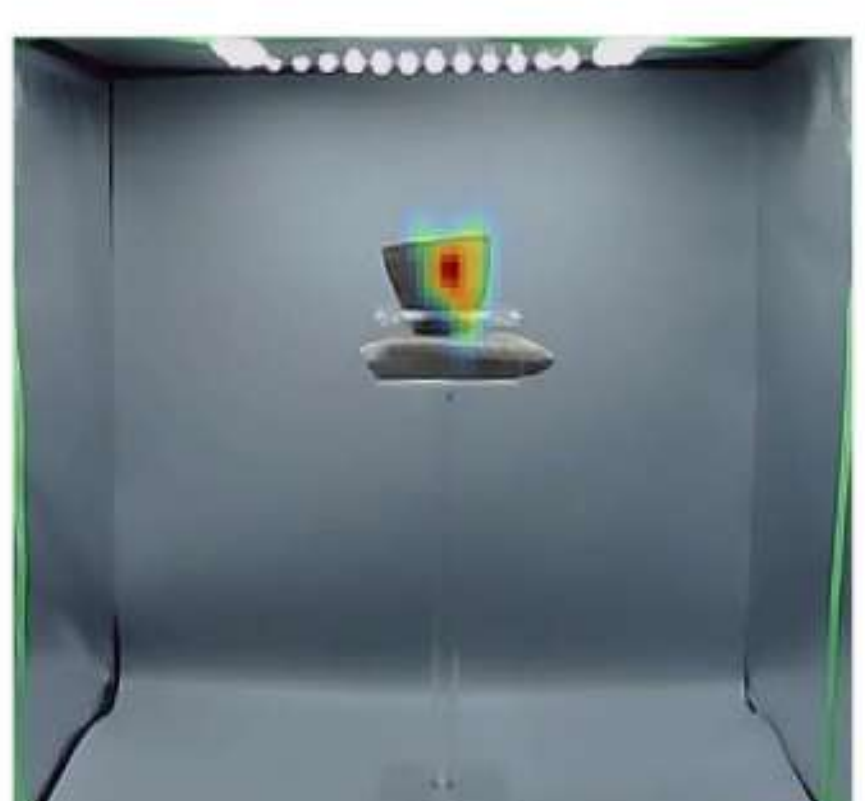
Part0115

Obj0246

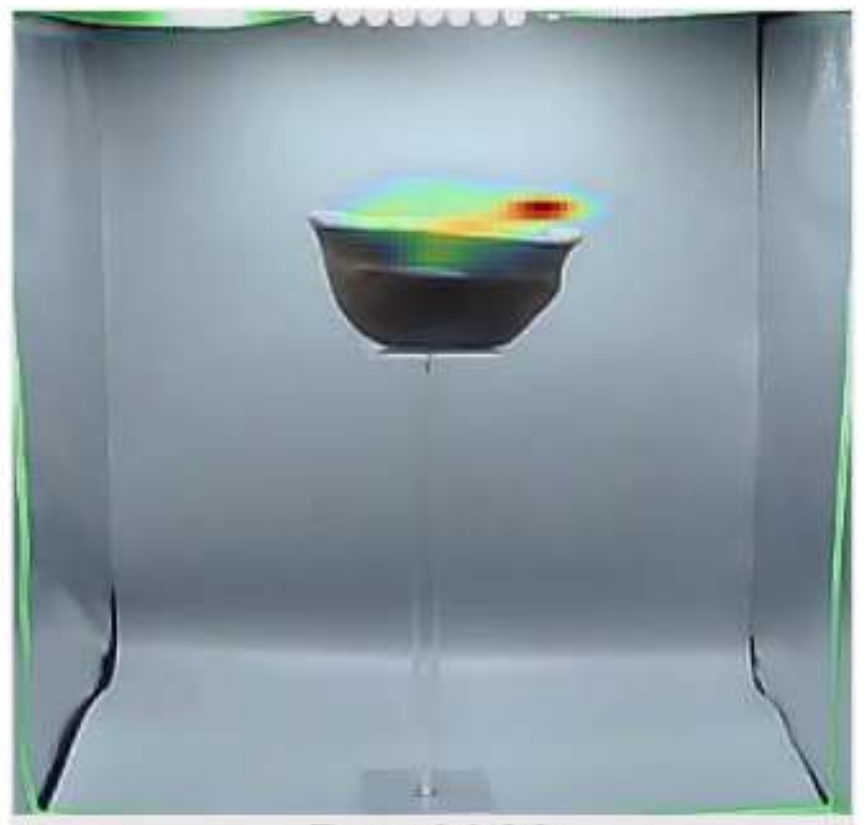
Part0089

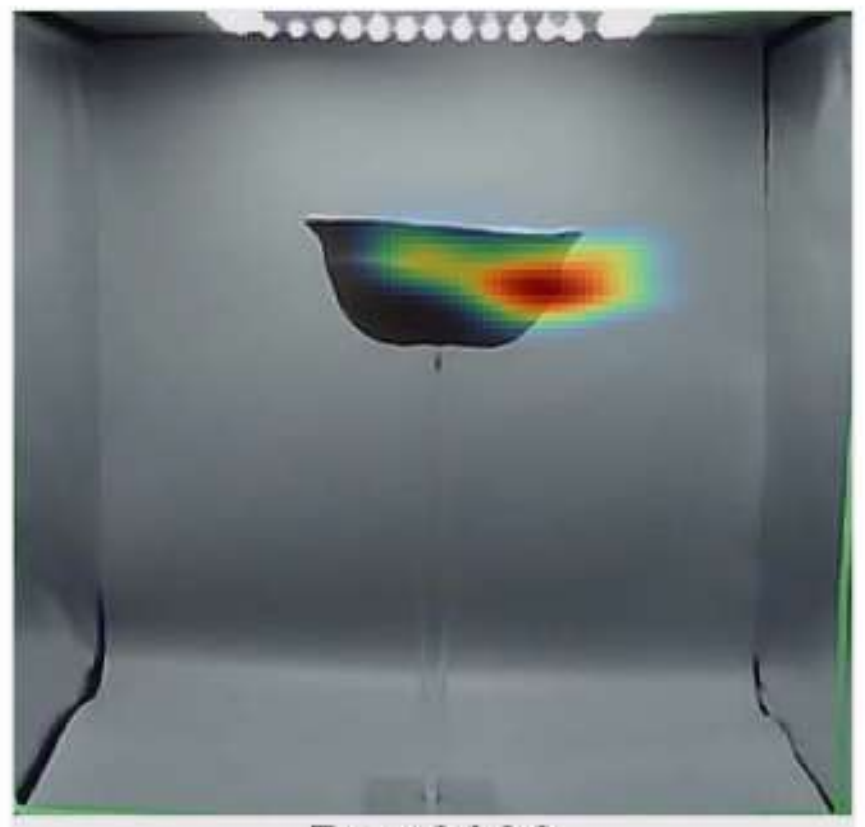
Part0090

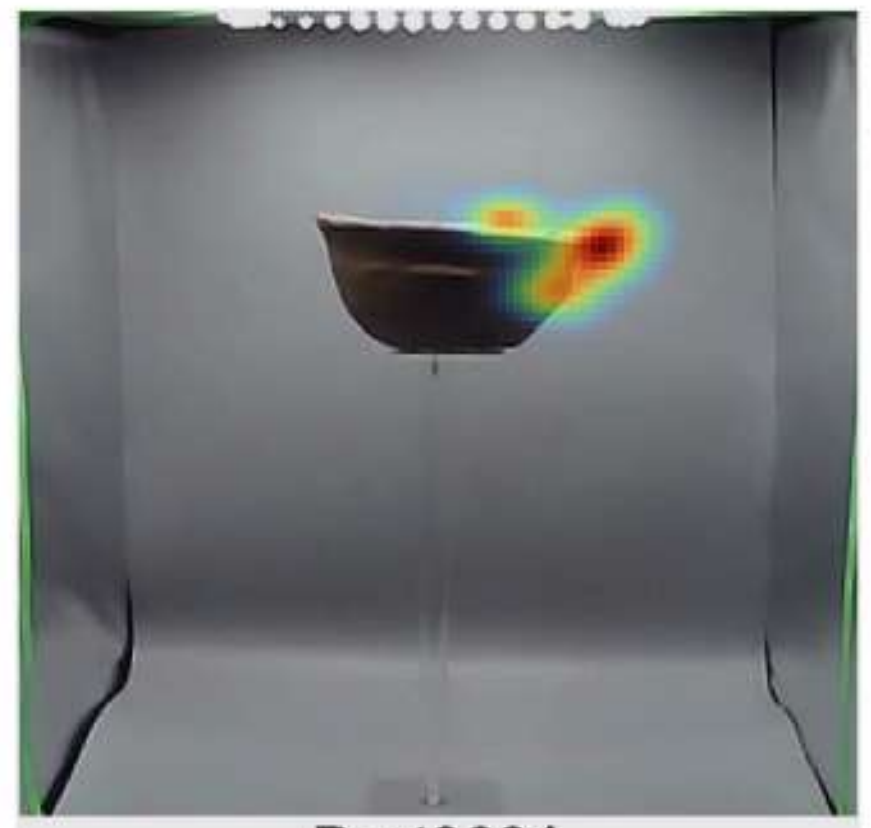
Part0091

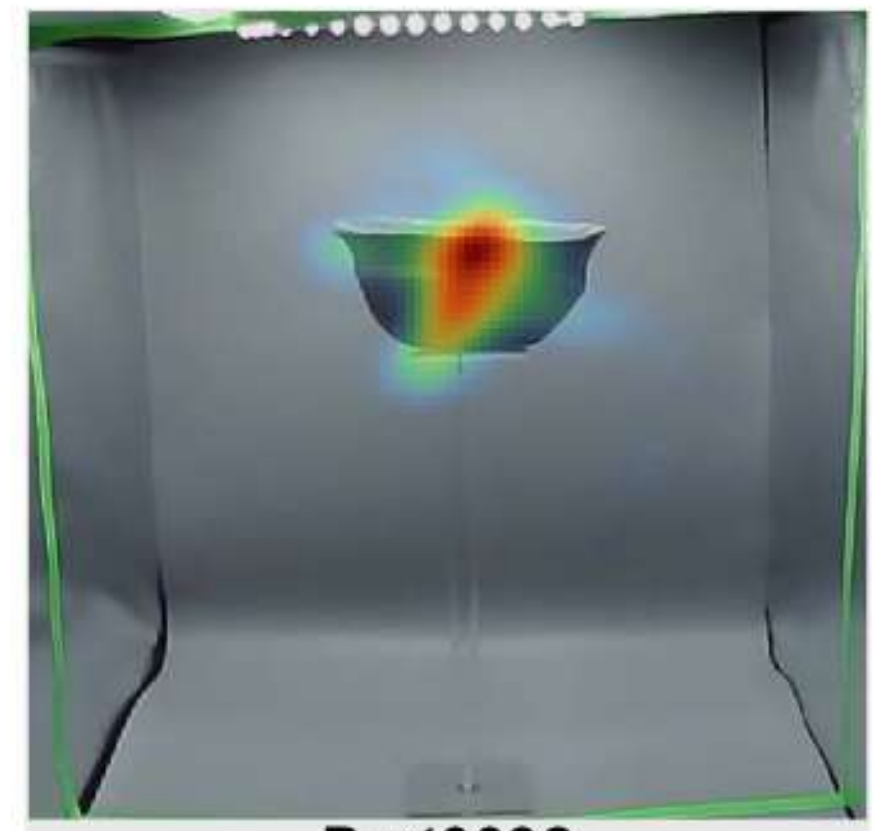
Part0092

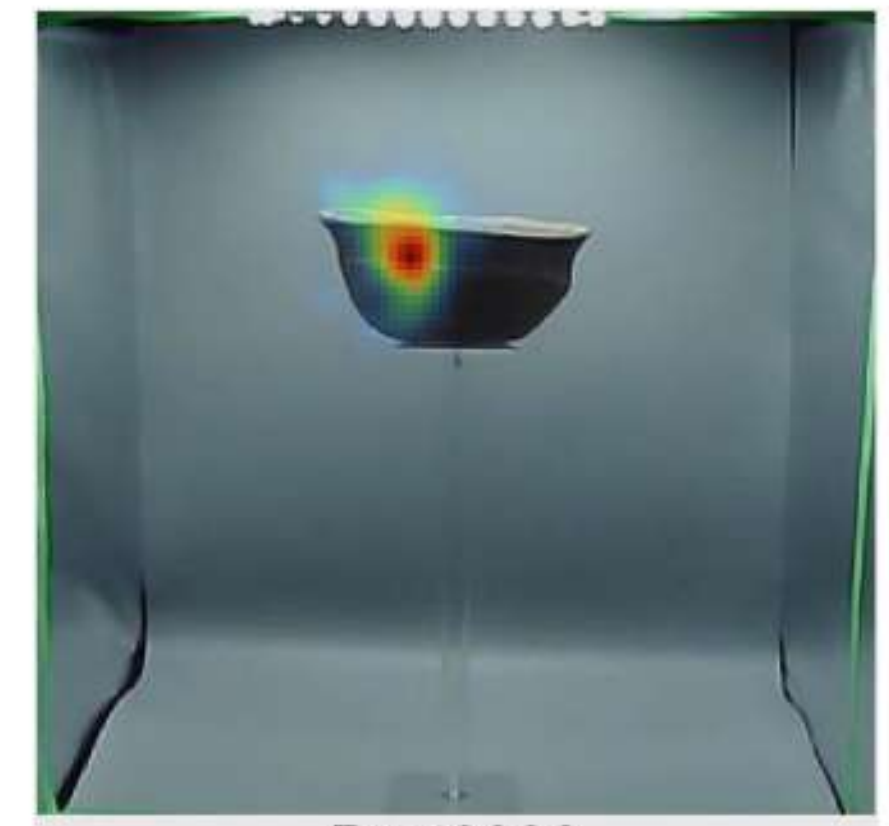
Part0093

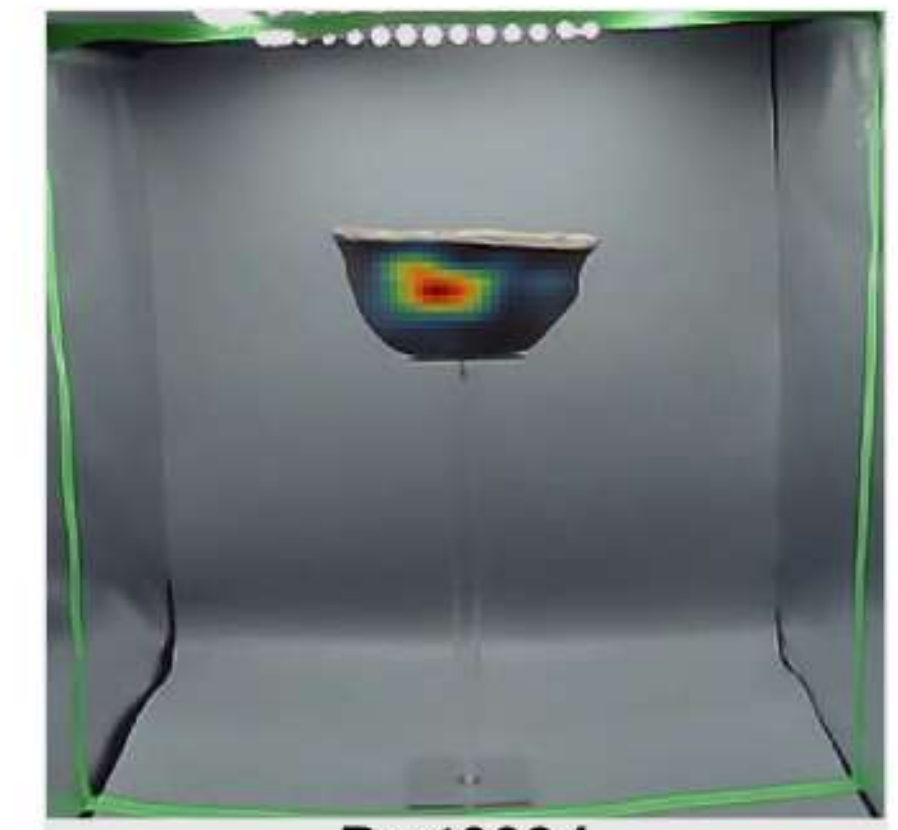
Part0094

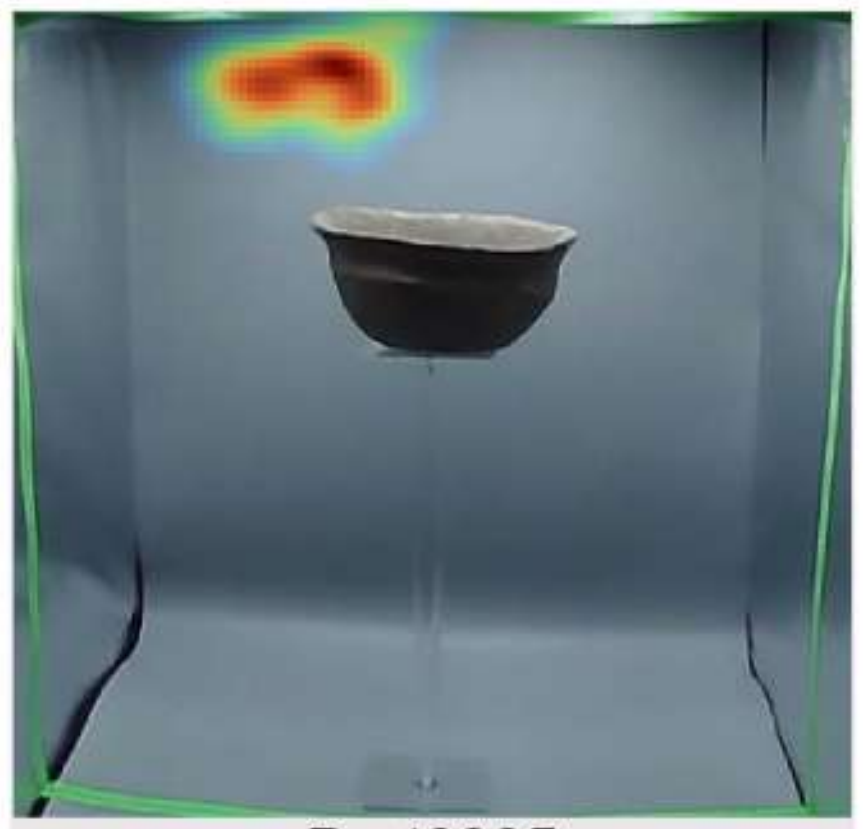
Part0095

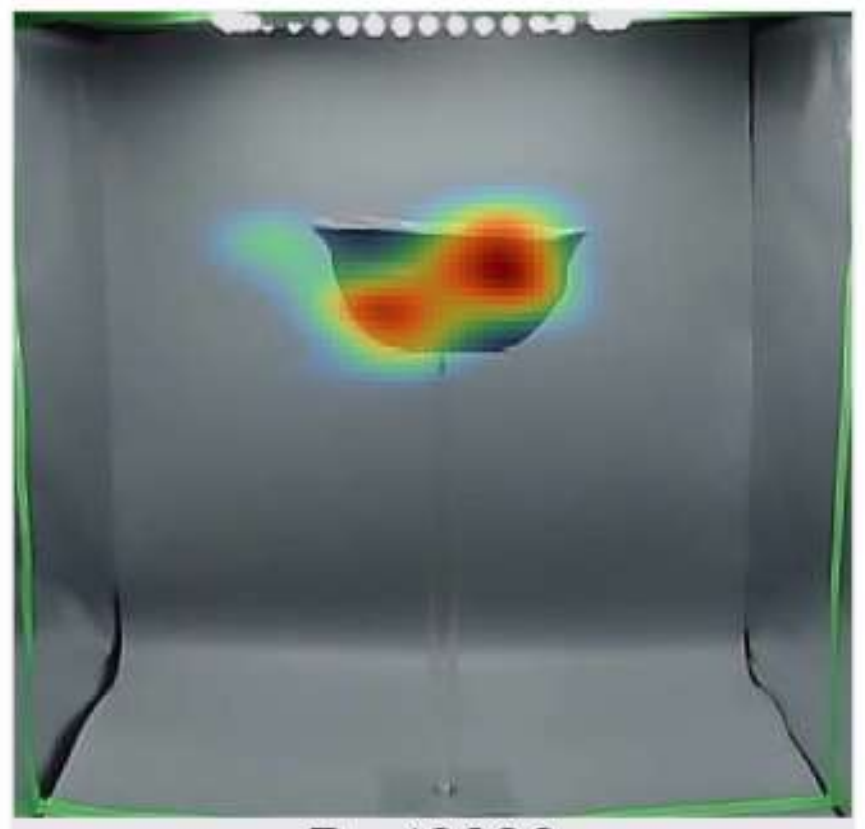
Part0096

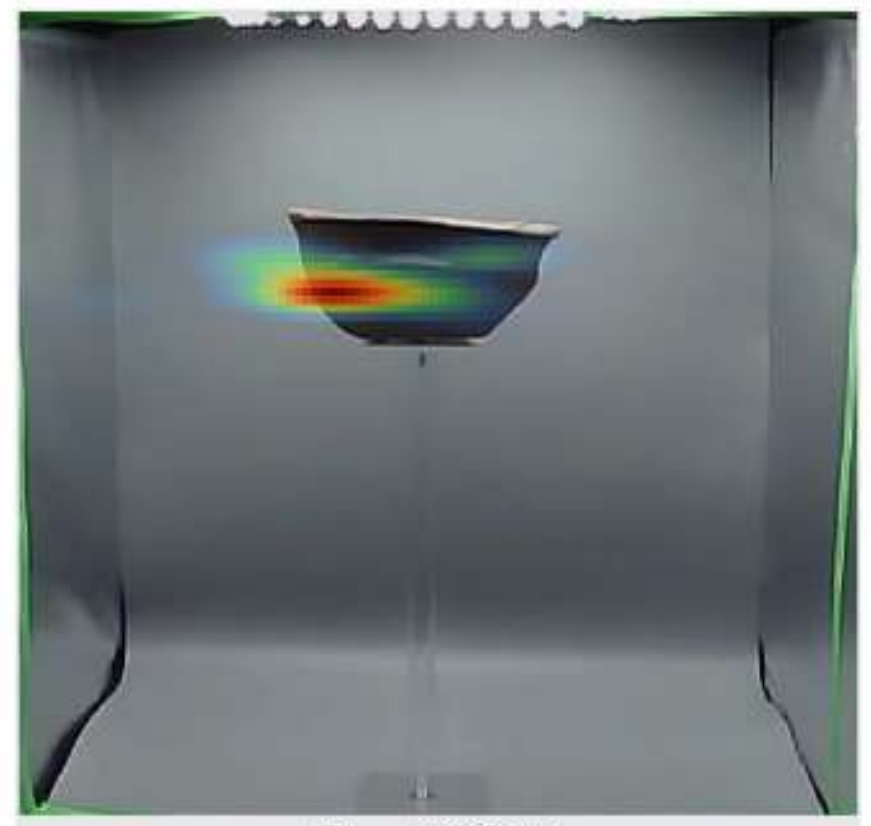
Part0097

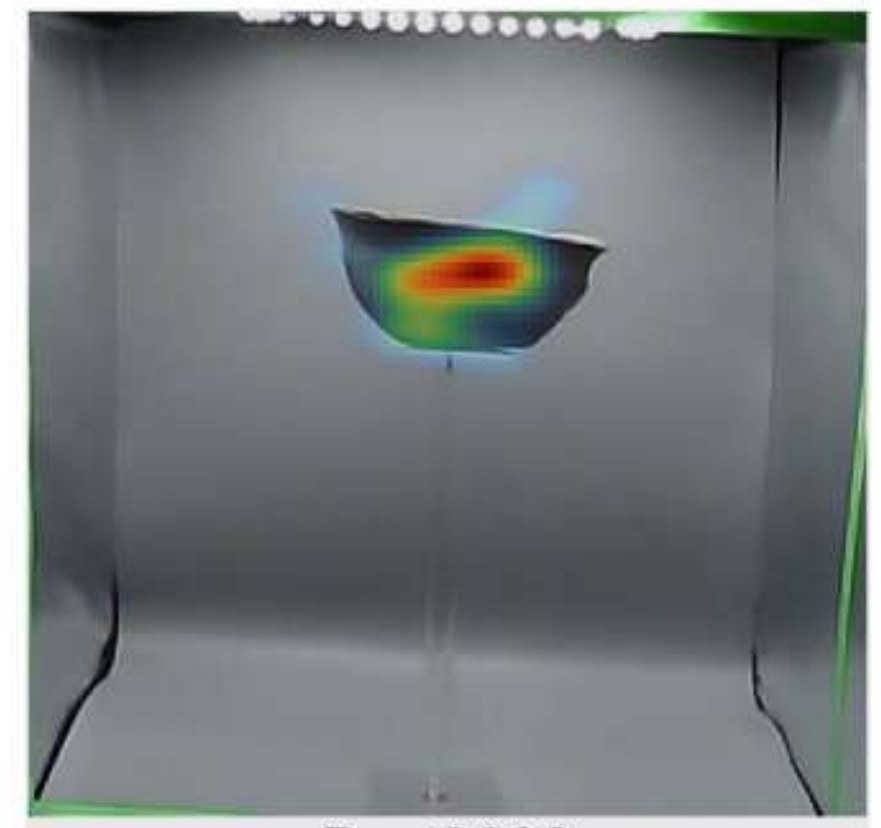
Part0098

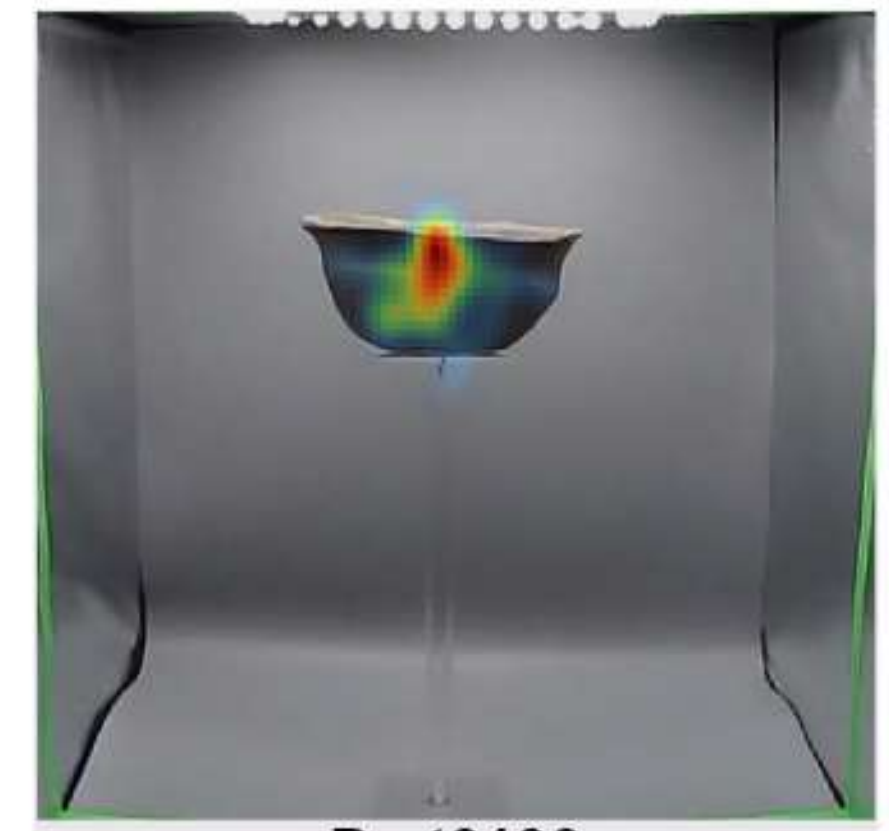
Part0100

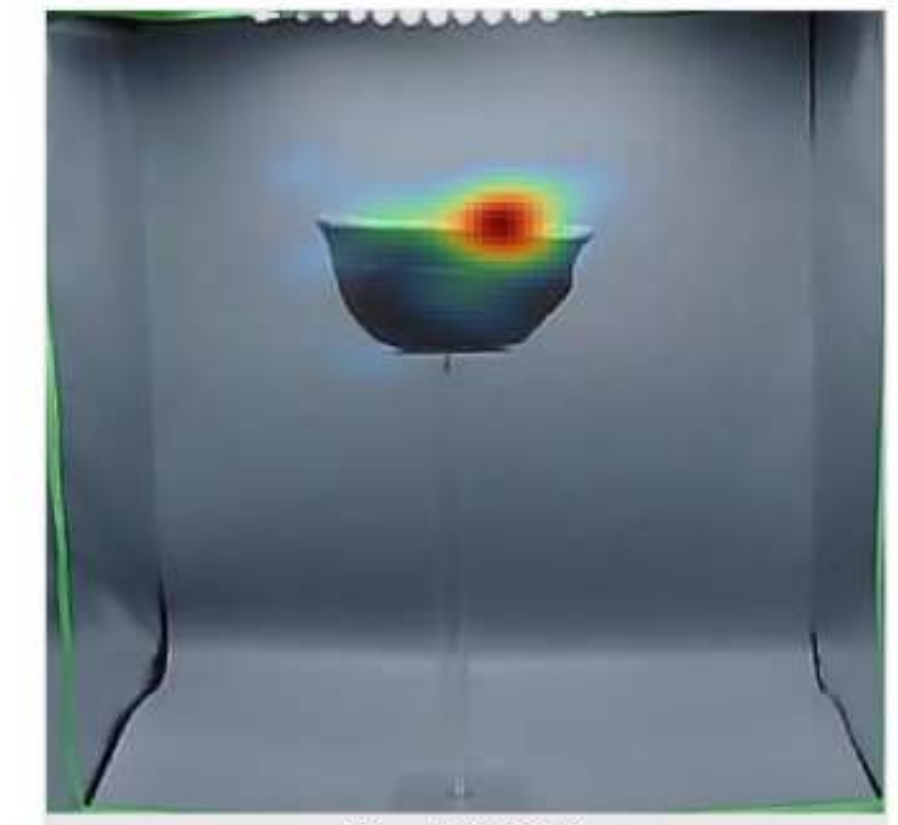
Part0101

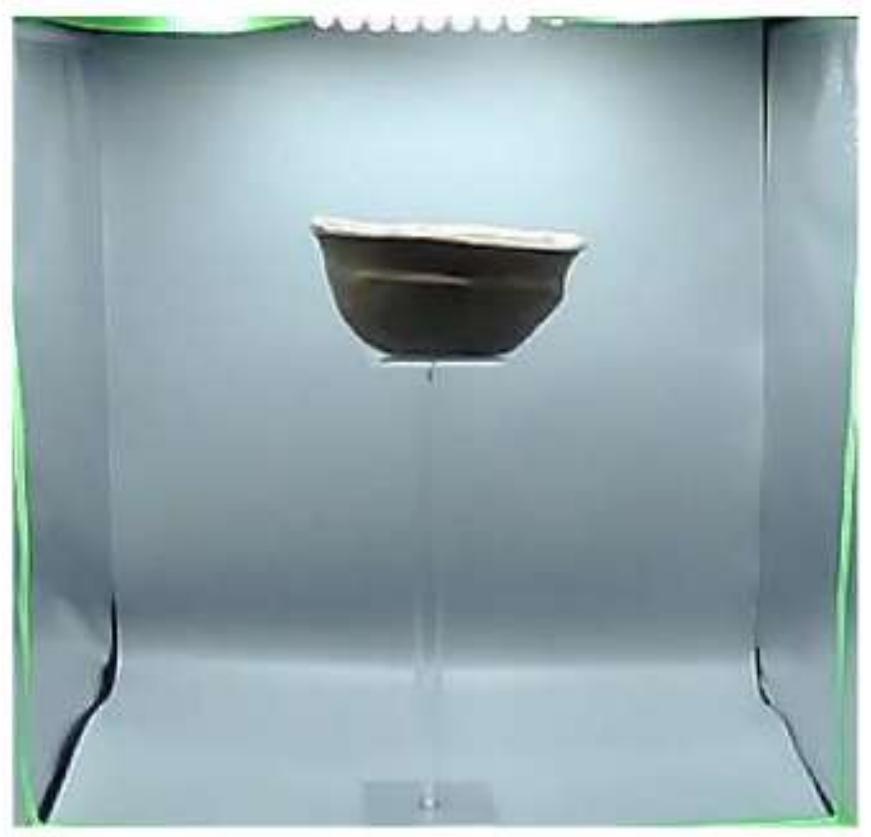
Part0102

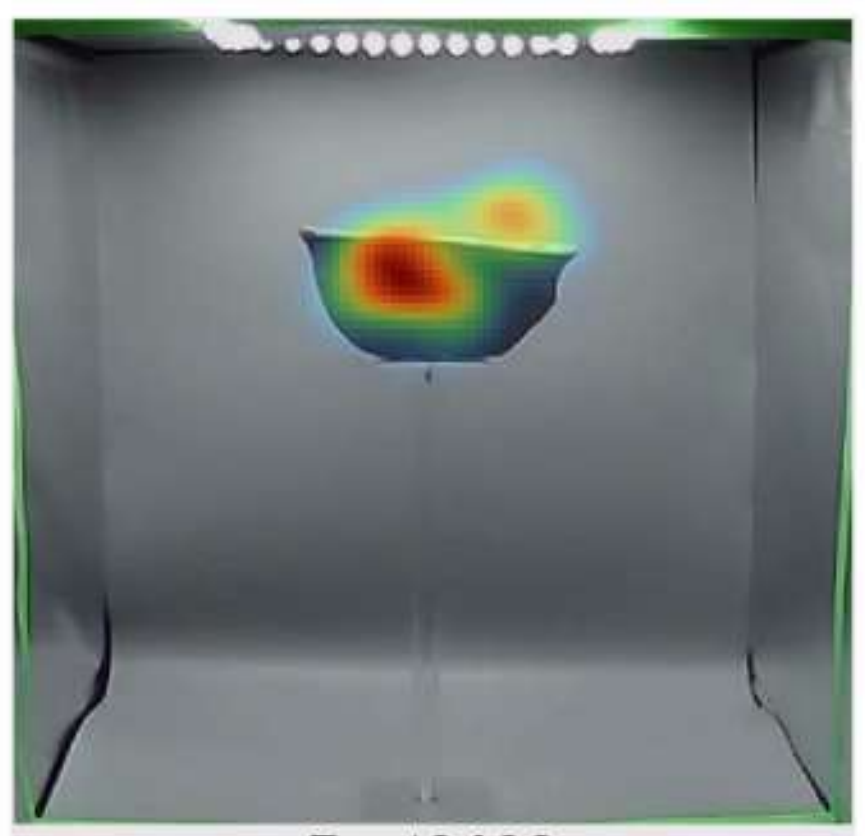
Part0103

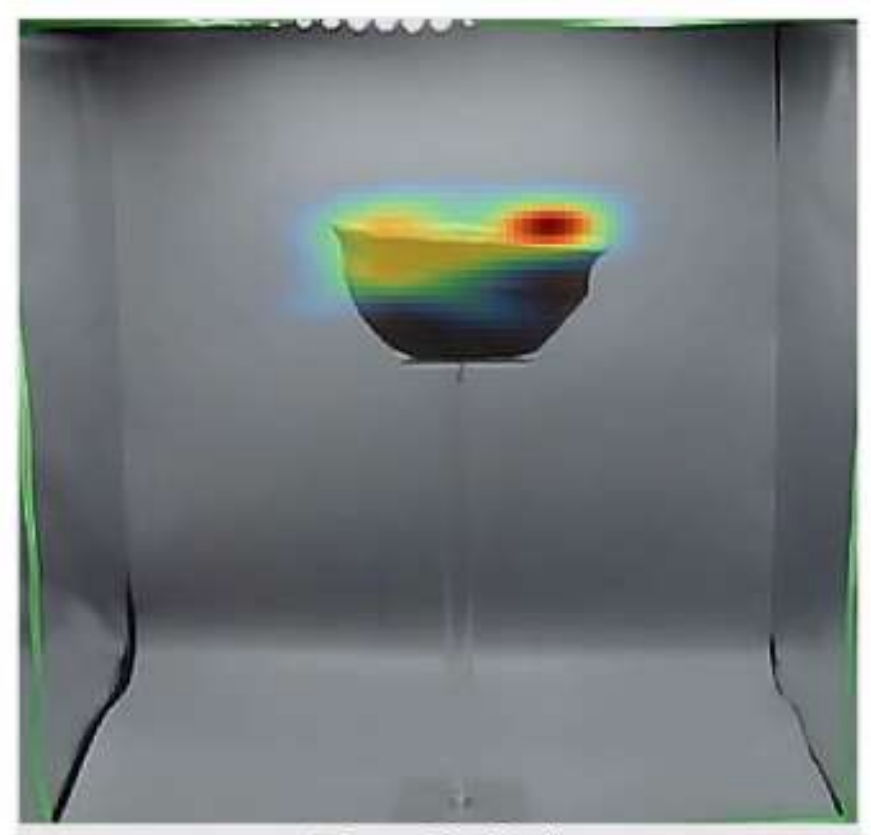
Part0104

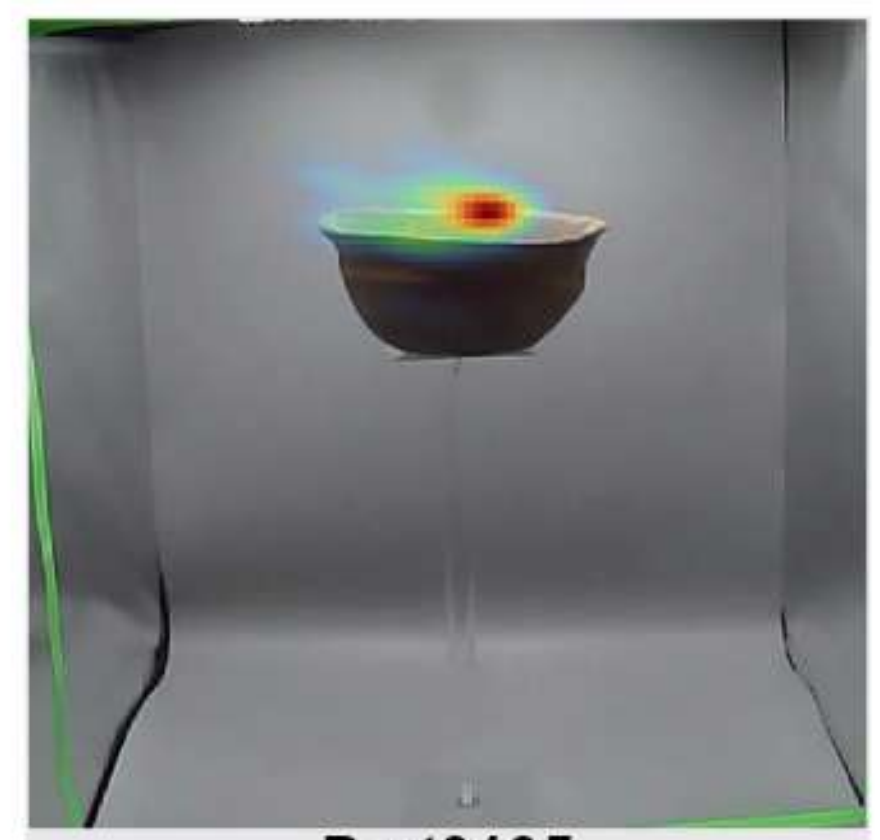
Part0105

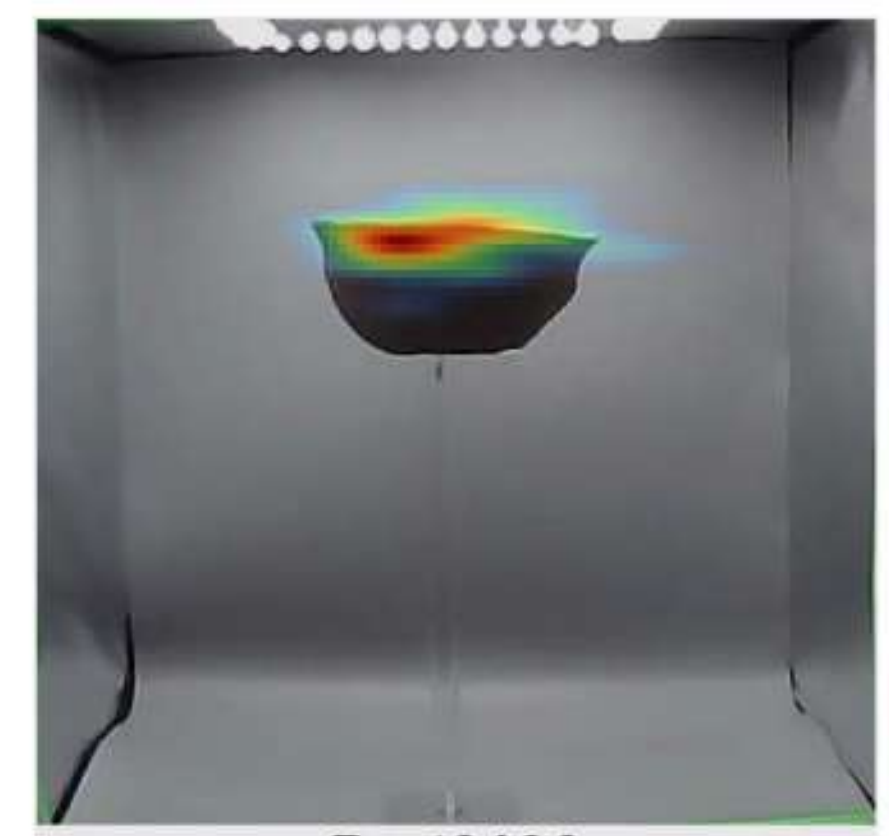
Part0106

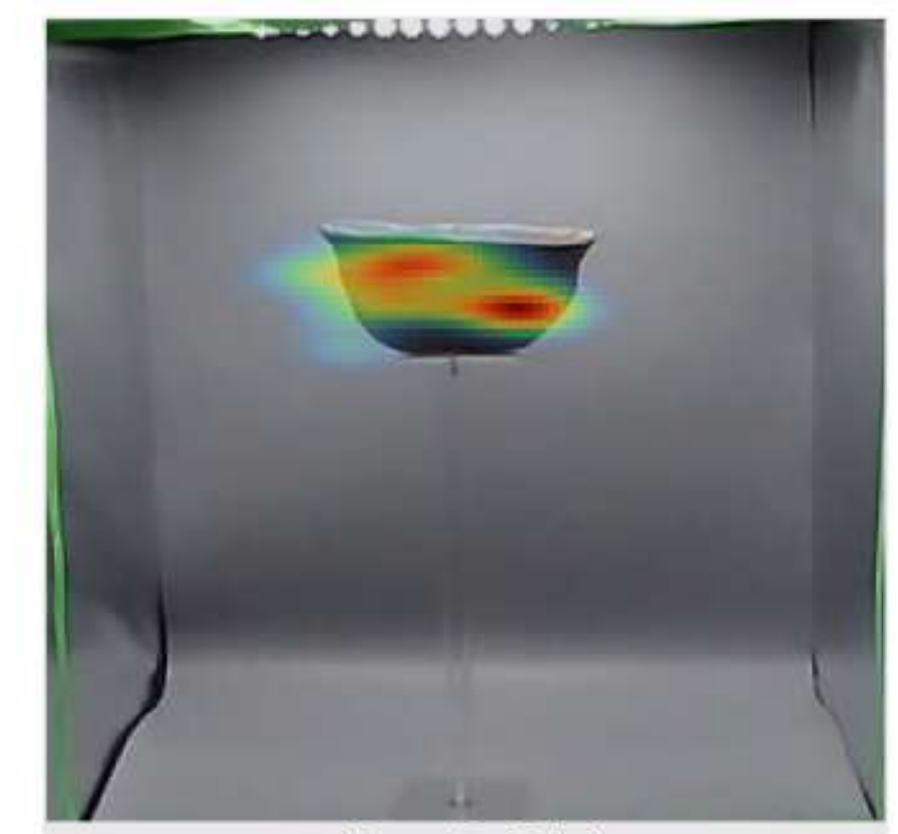
Part0107

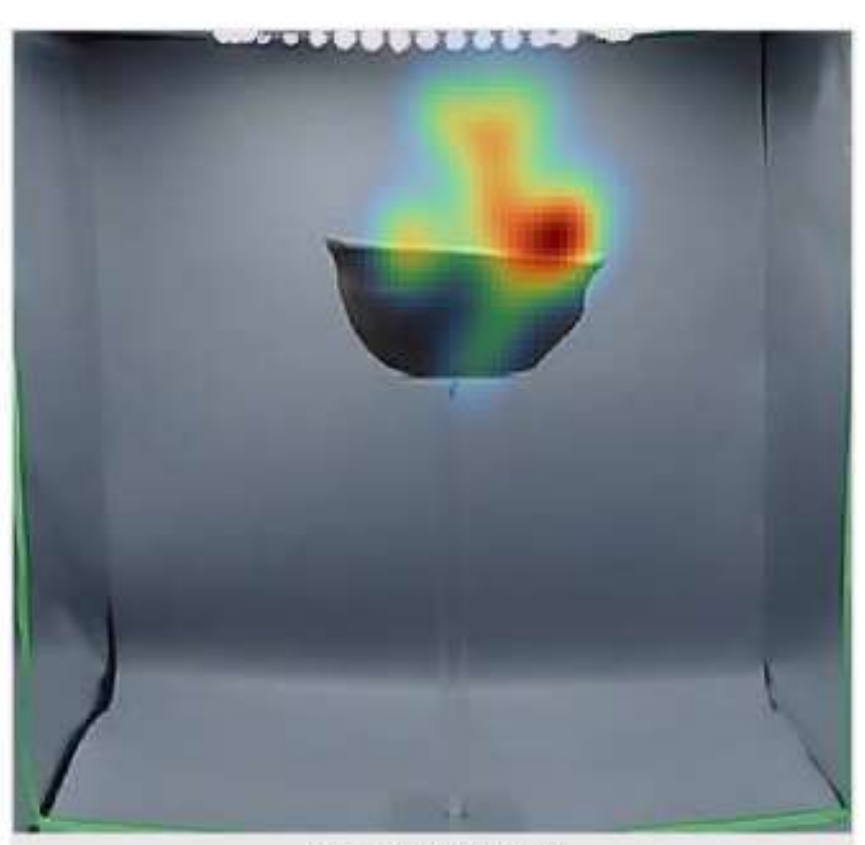
Part0108

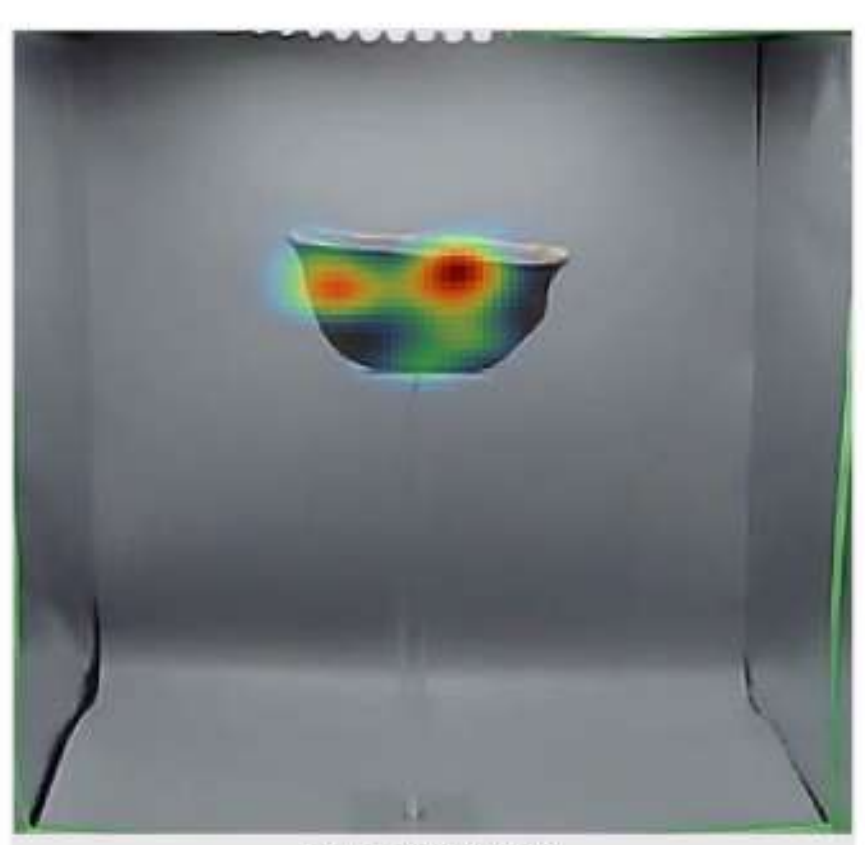
Part0109

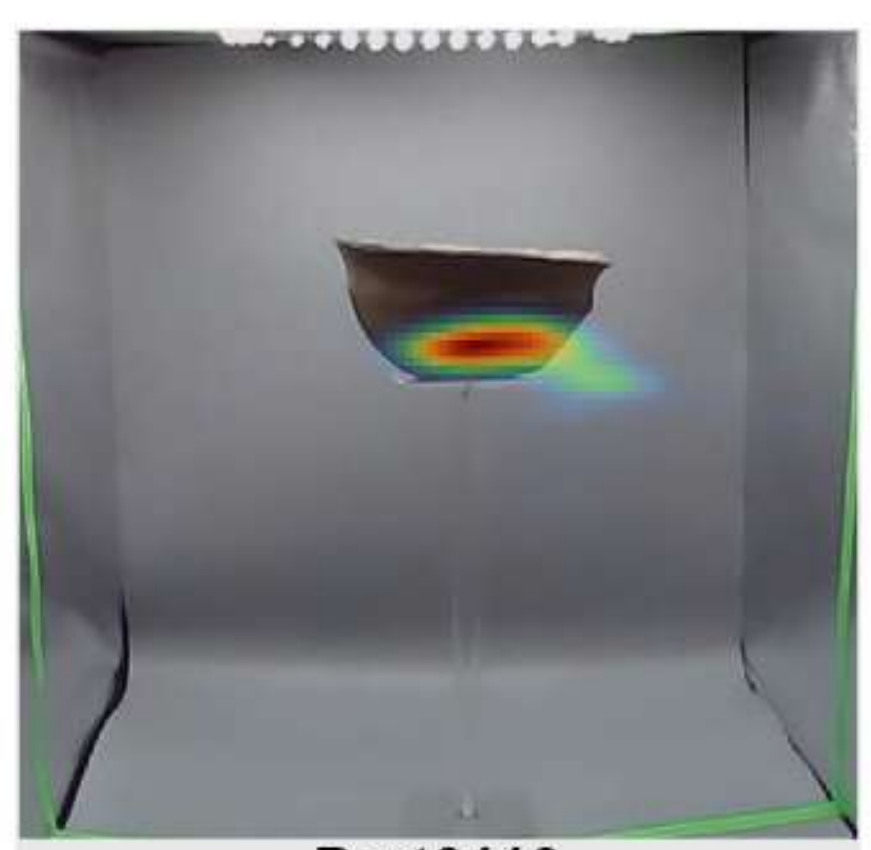
Part0110

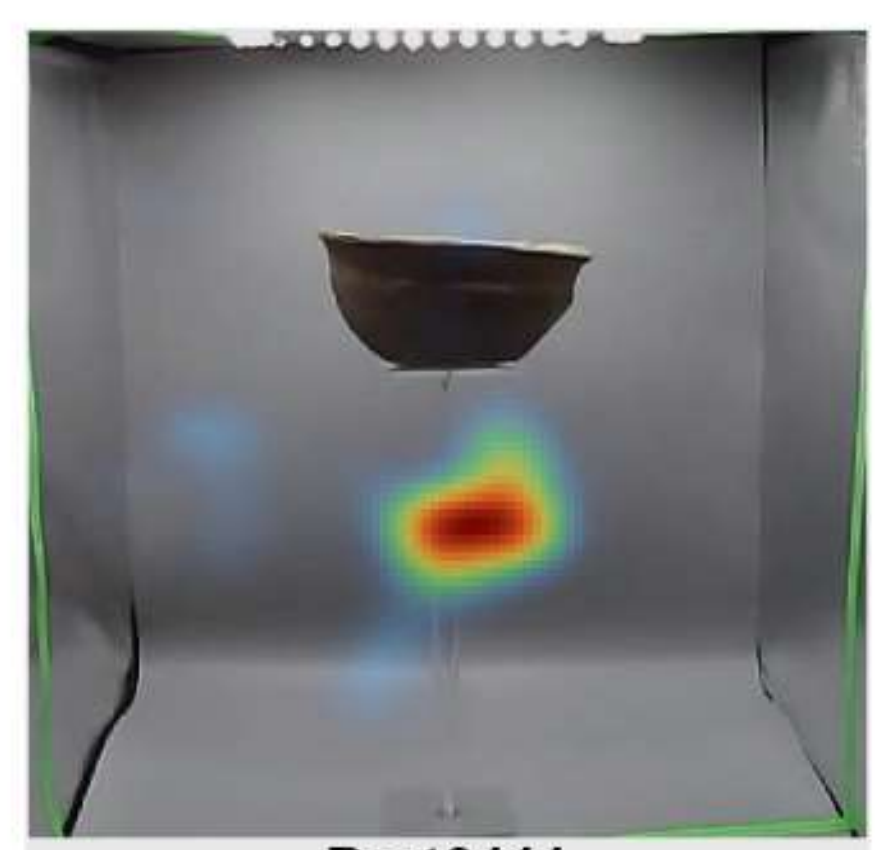
Part0111

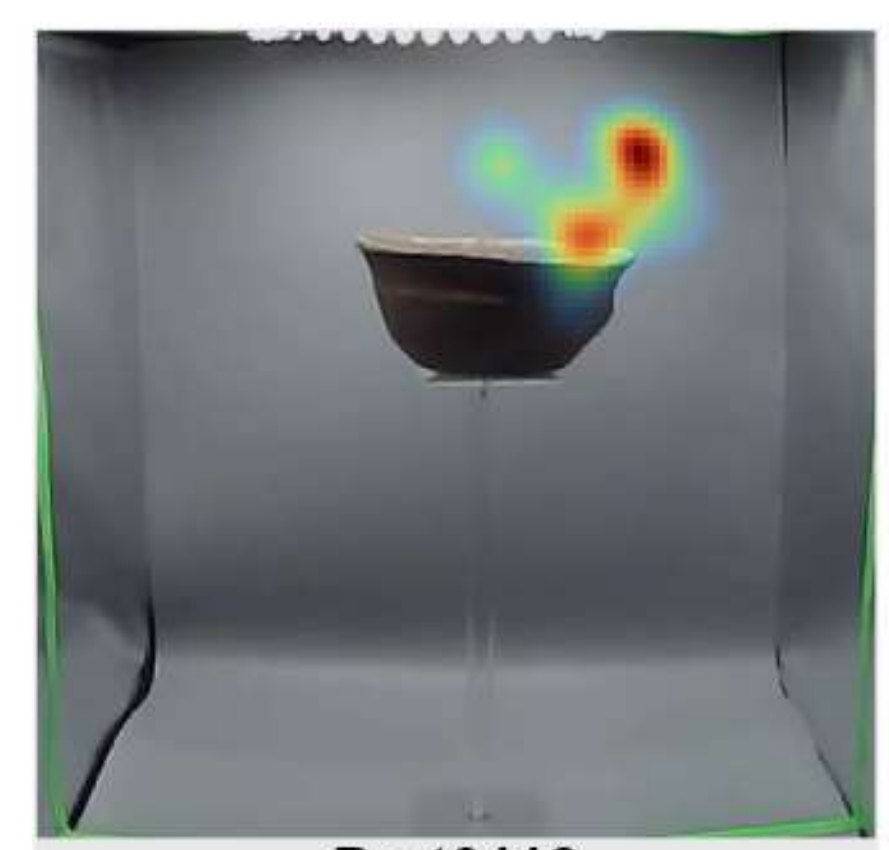
Part0112

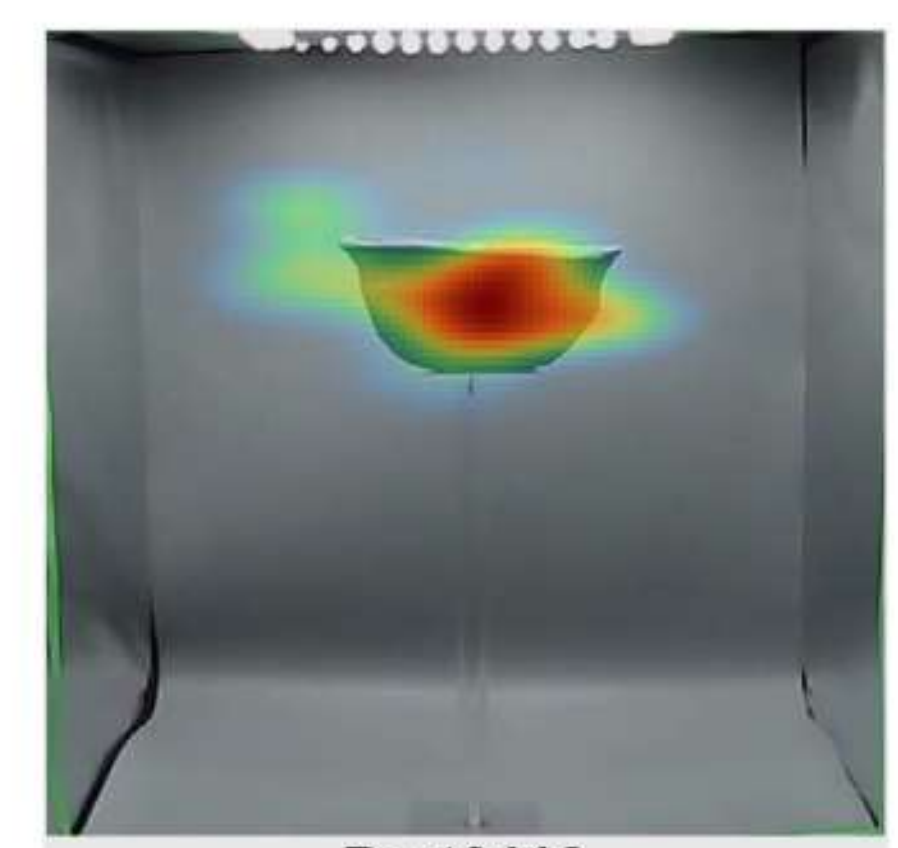
Part0113

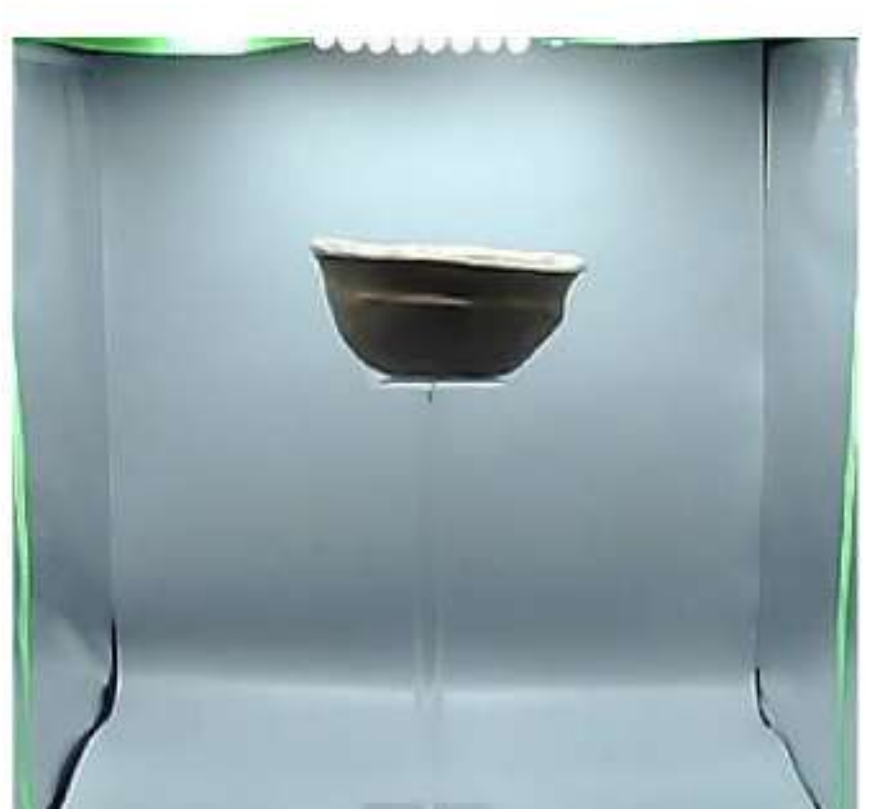
Part0114

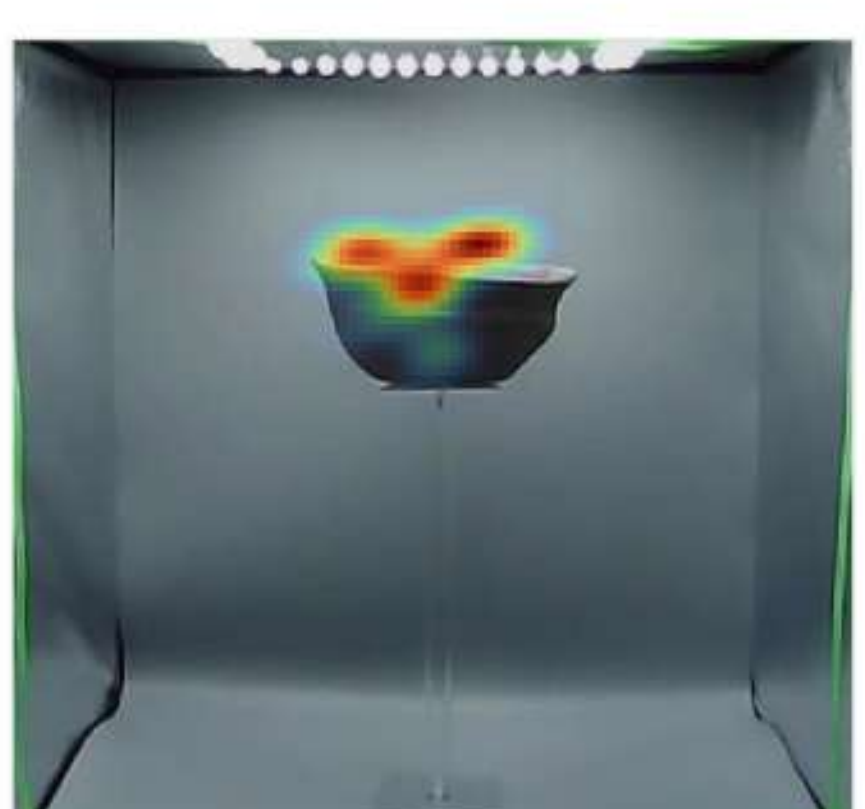
Part0115

Obj0248

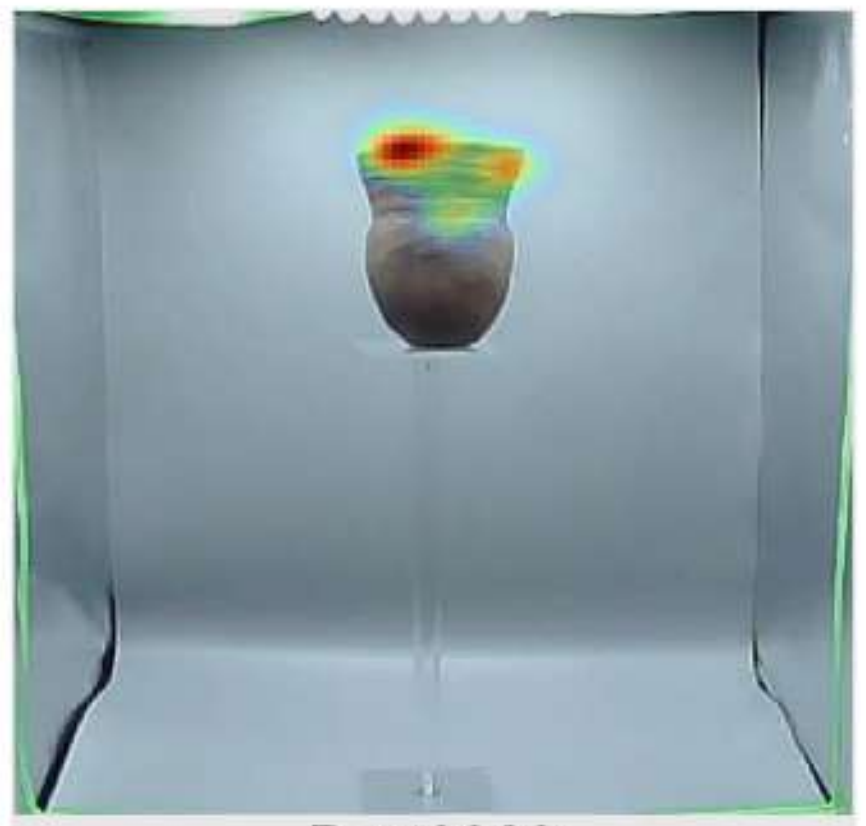
Part0089

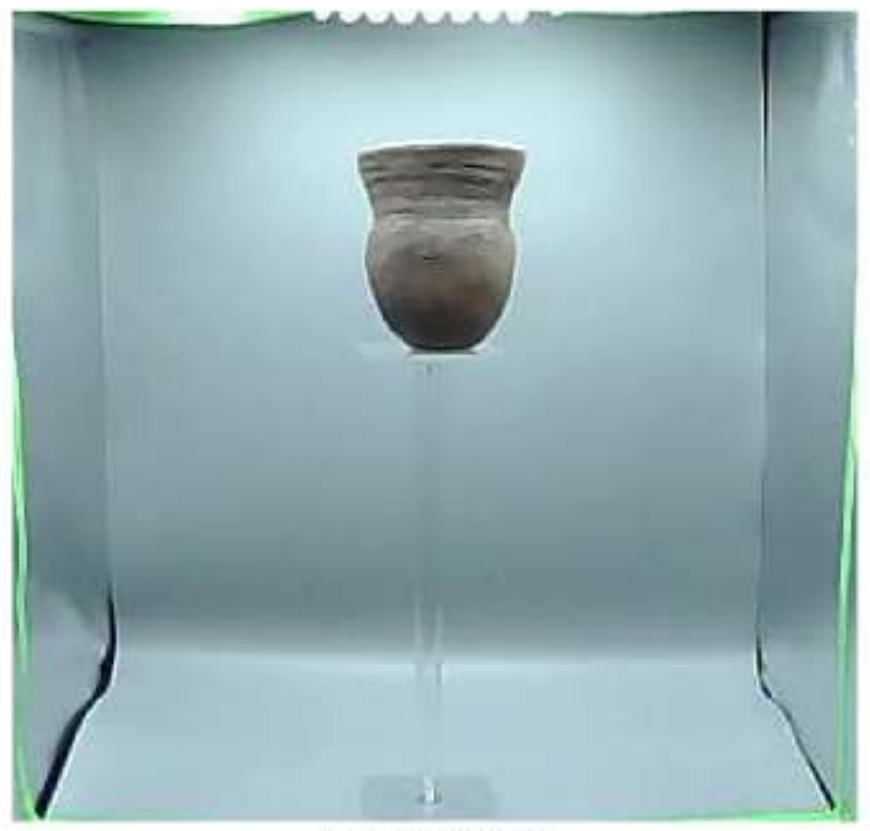
Part0090

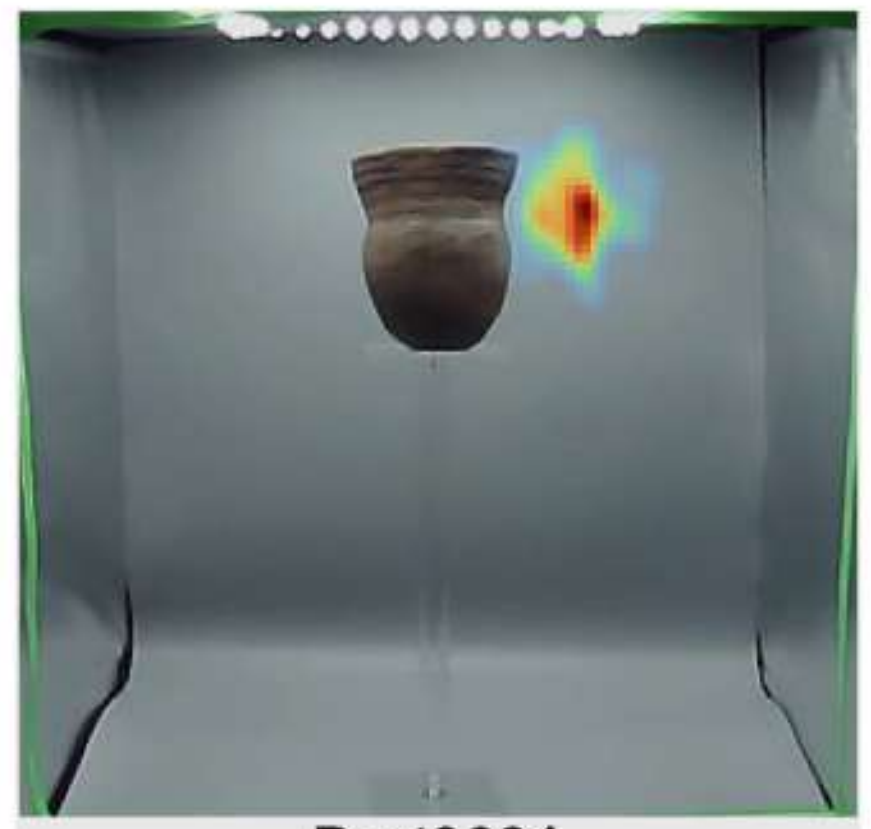
Part0091

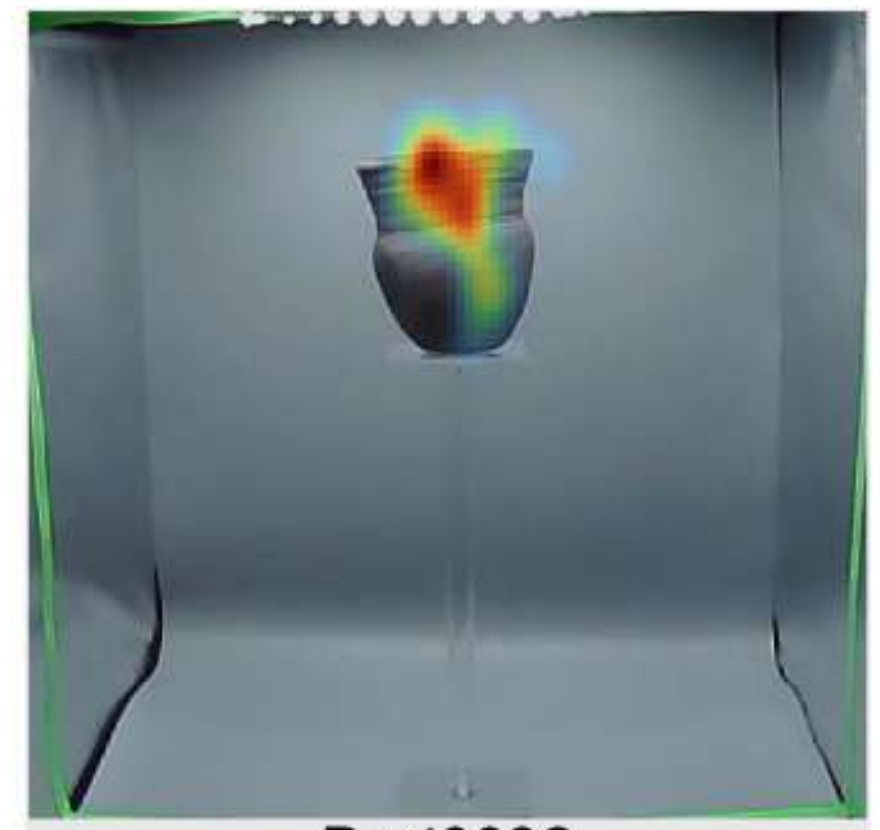
Part0092

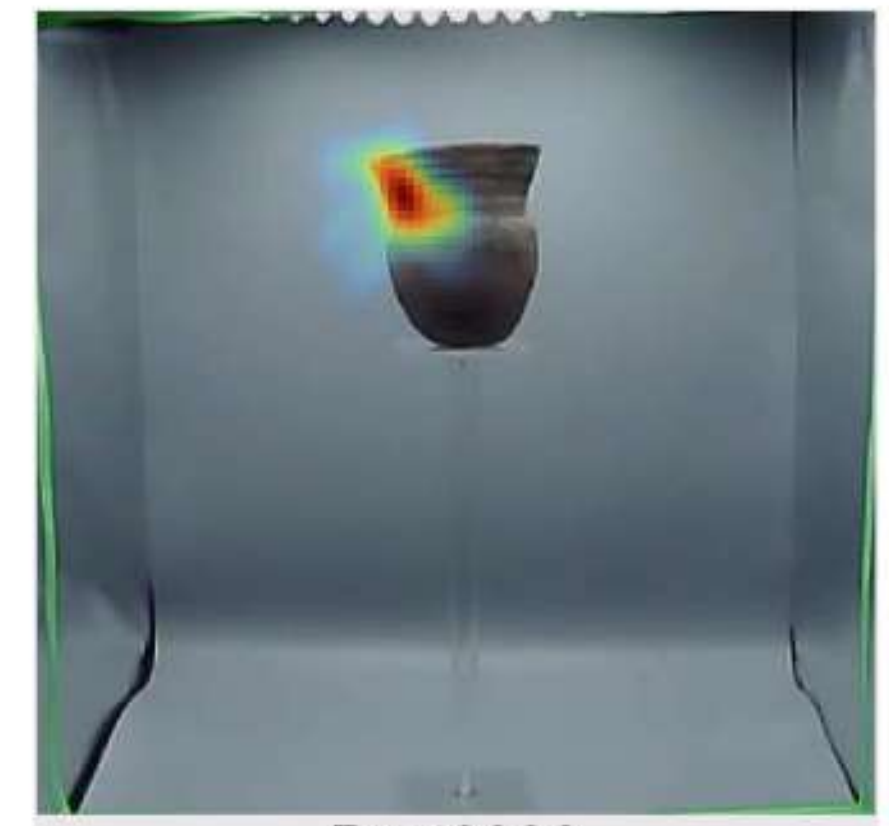
Part0093

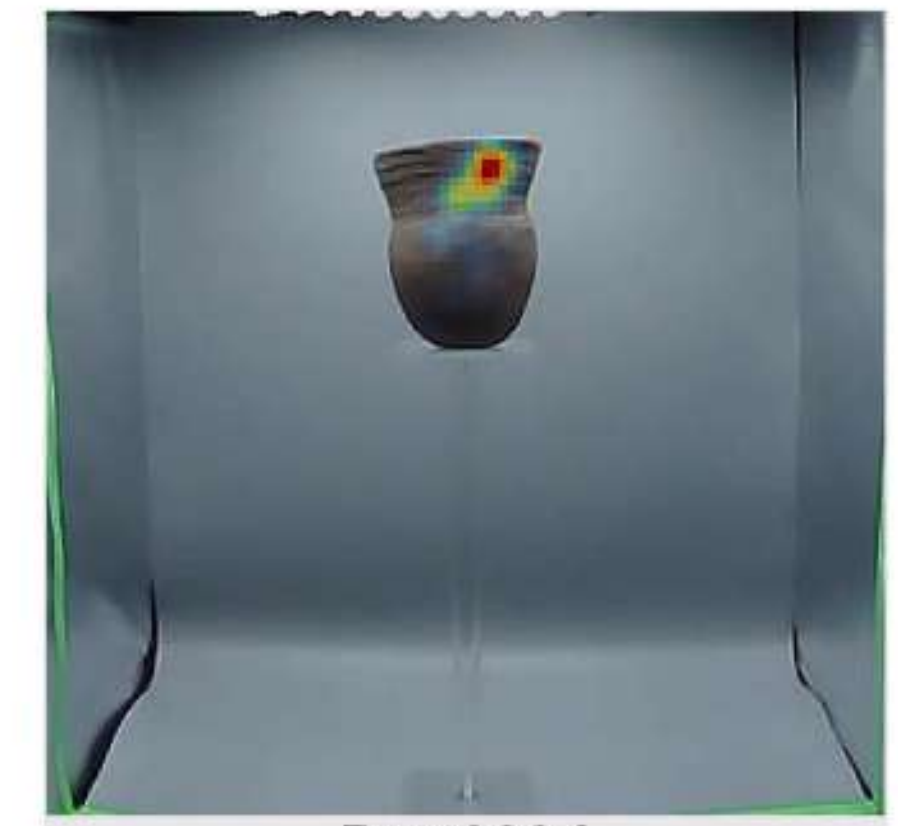
Part0094

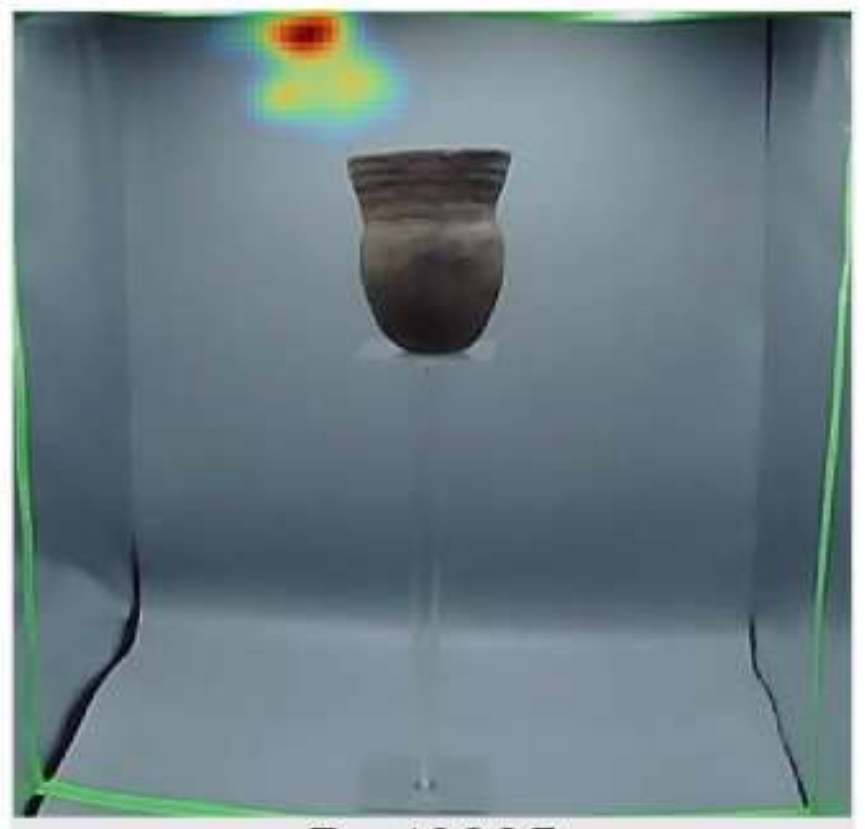
Part0095

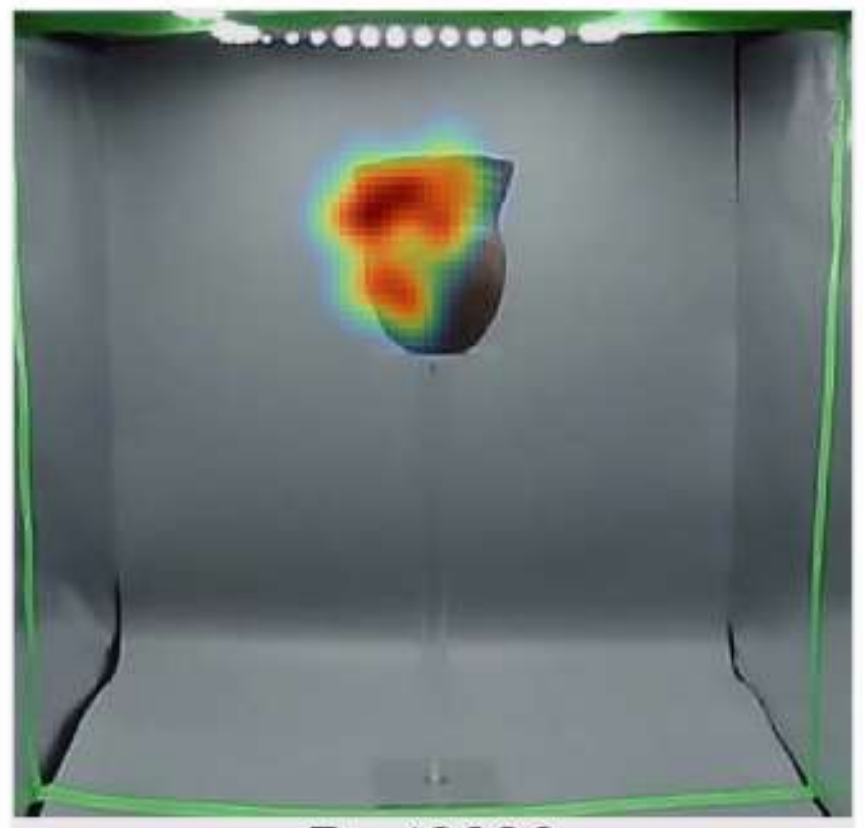
Part0096

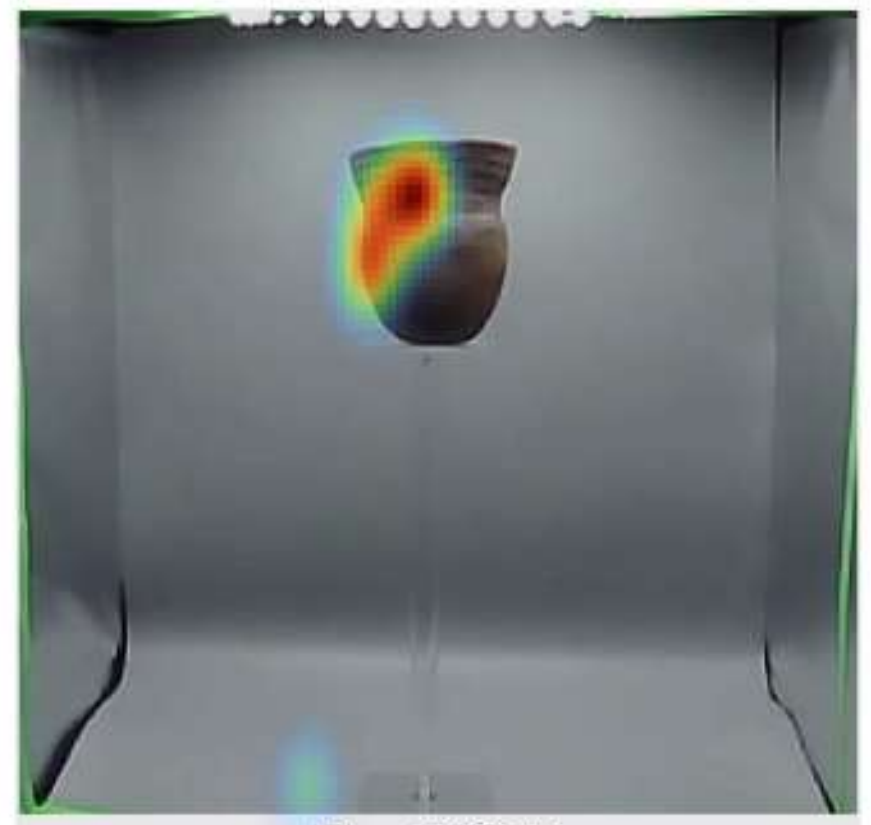
Part0097

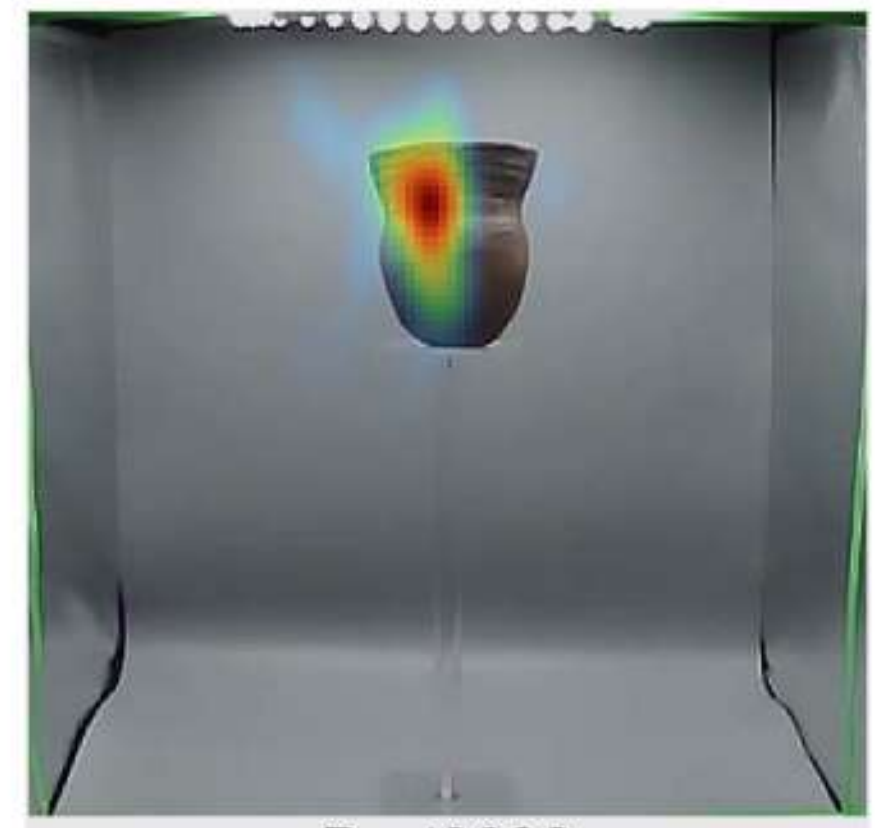
Part0098

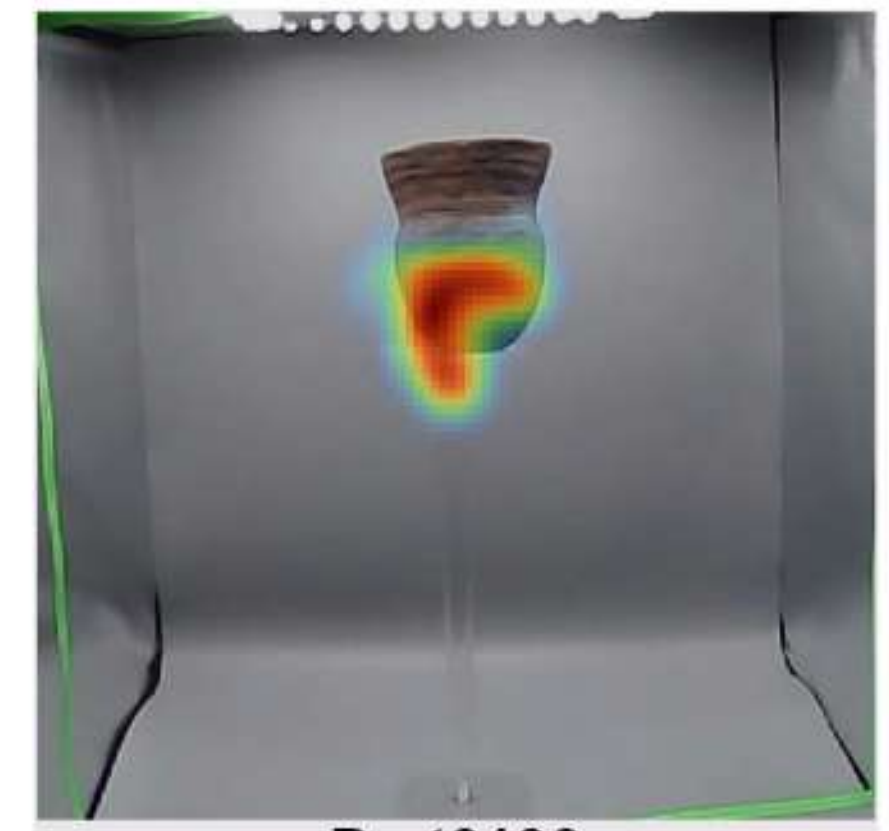
Part0100

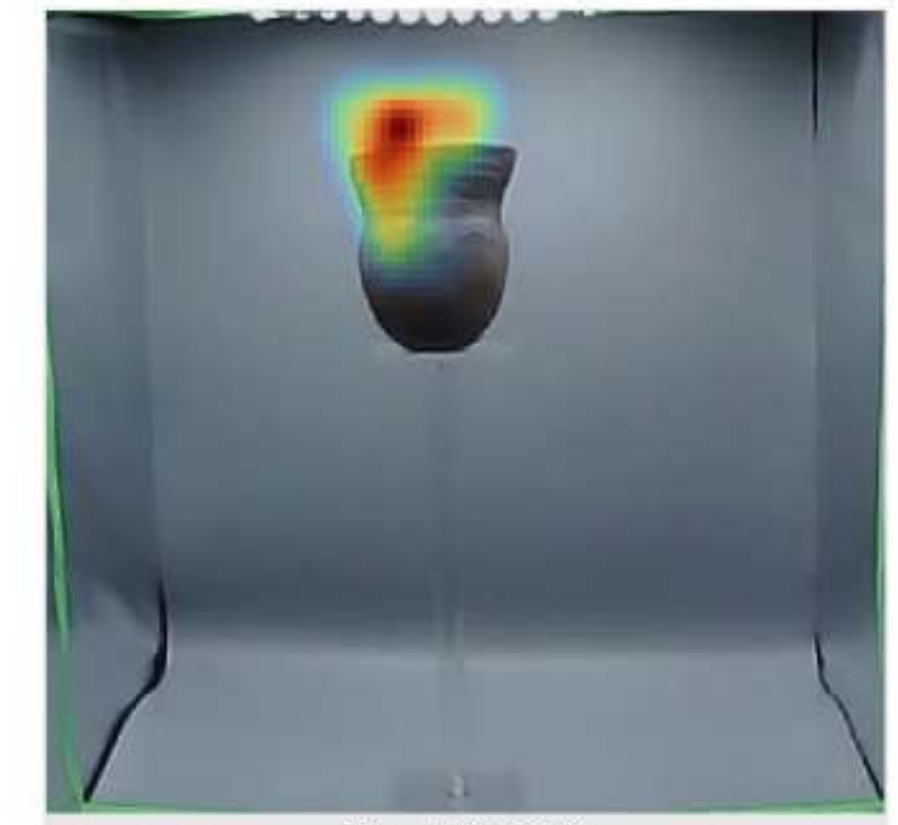
Part0101

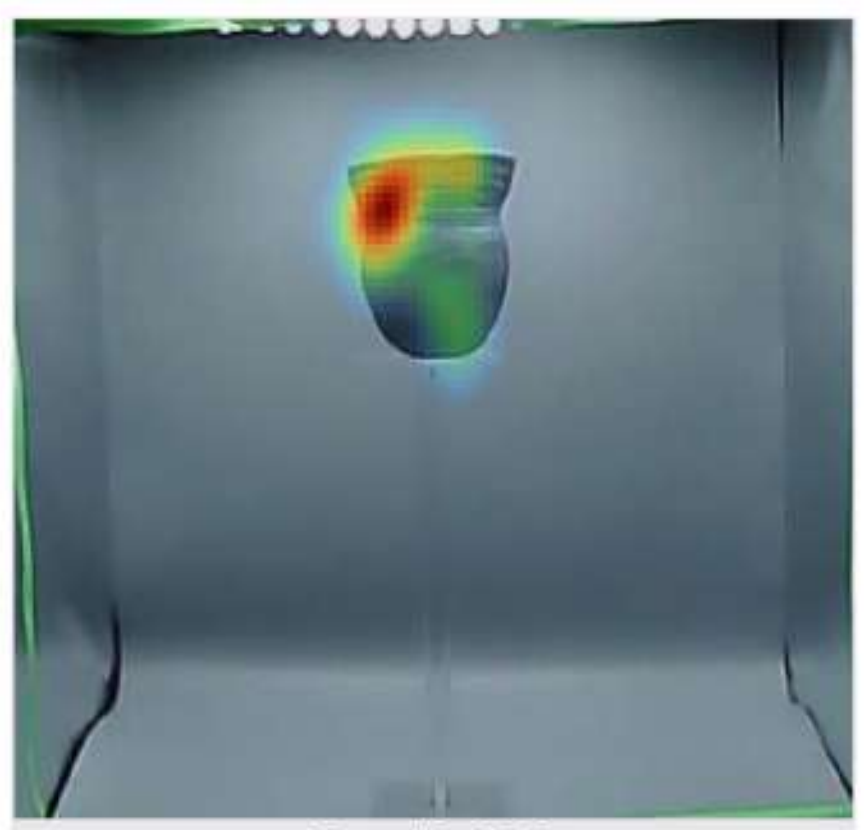
Part0102

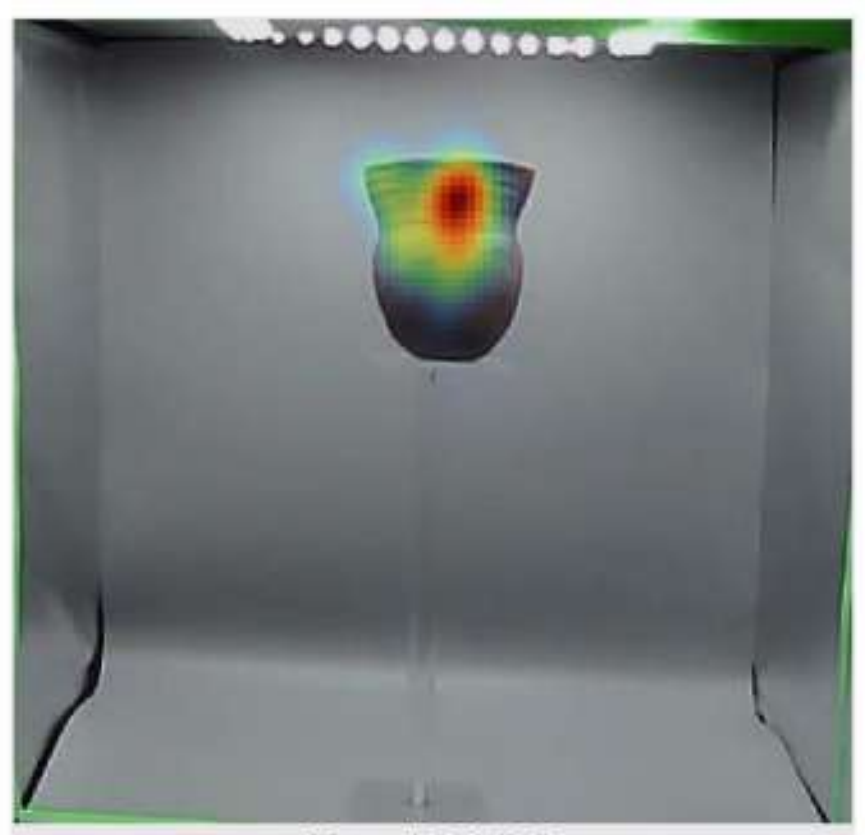
Part0103

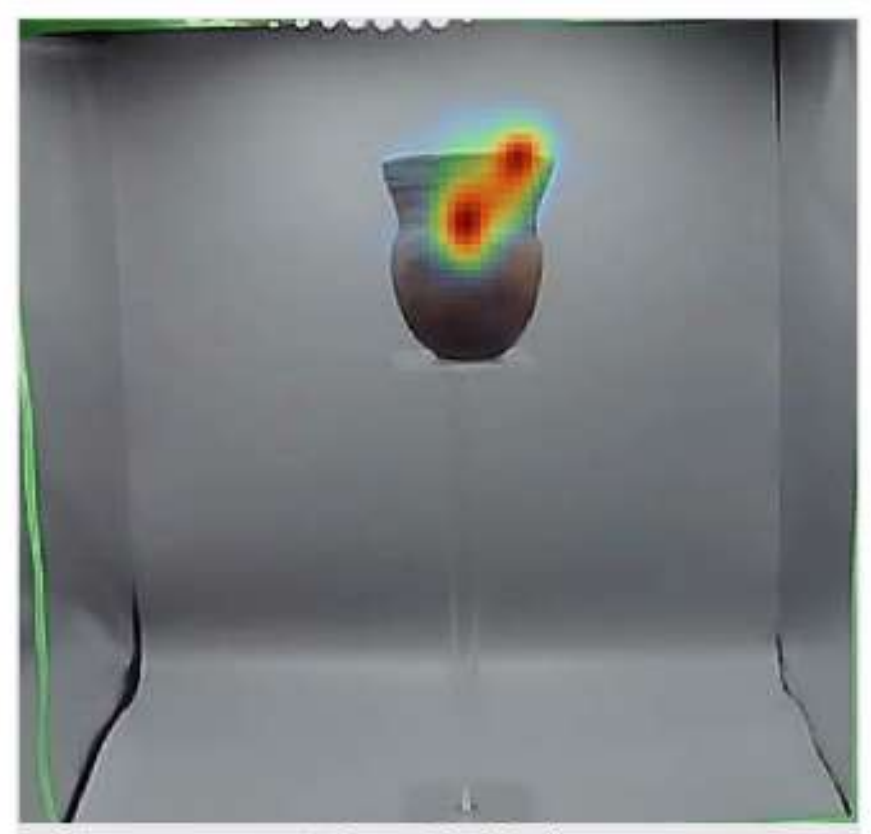
Part0104

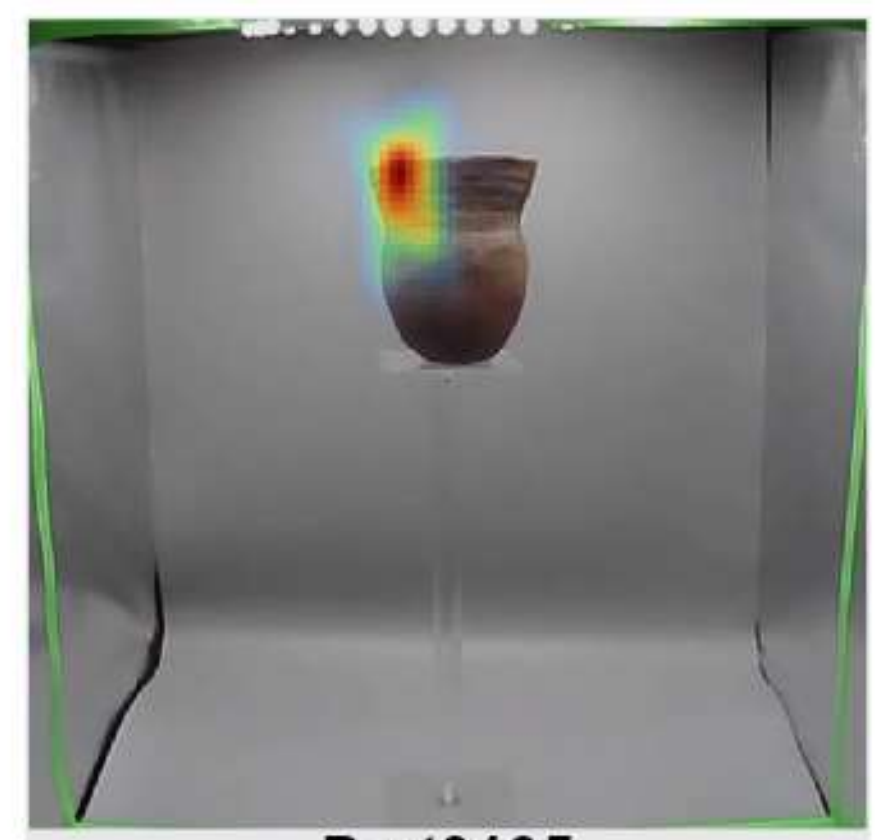
Part0105

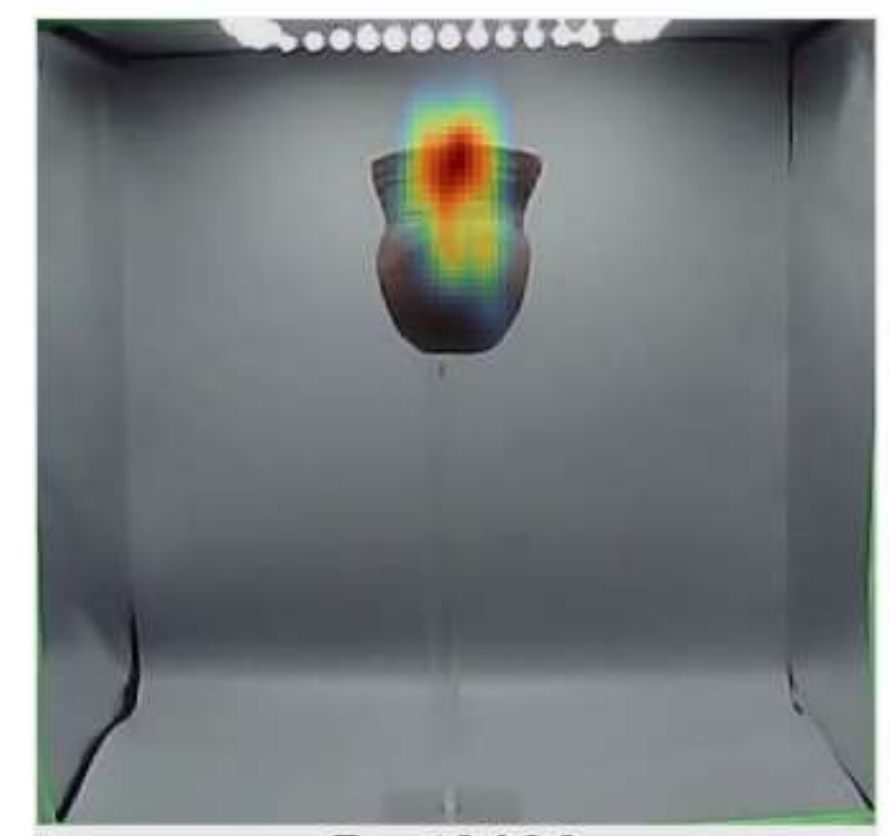
Part0106

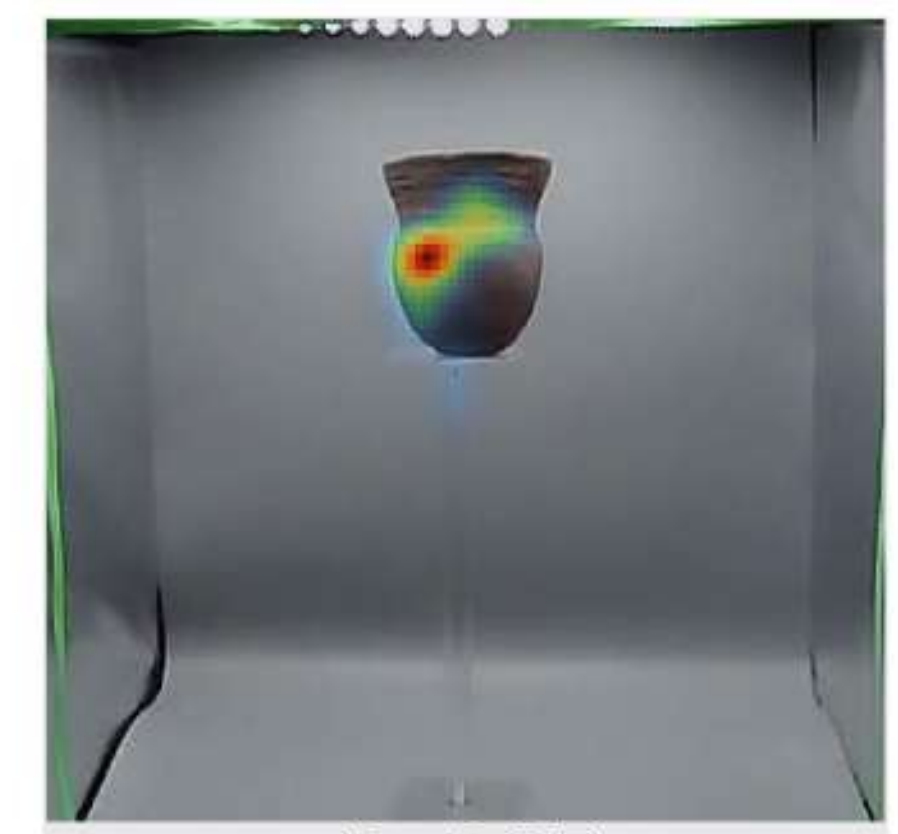
Part0107

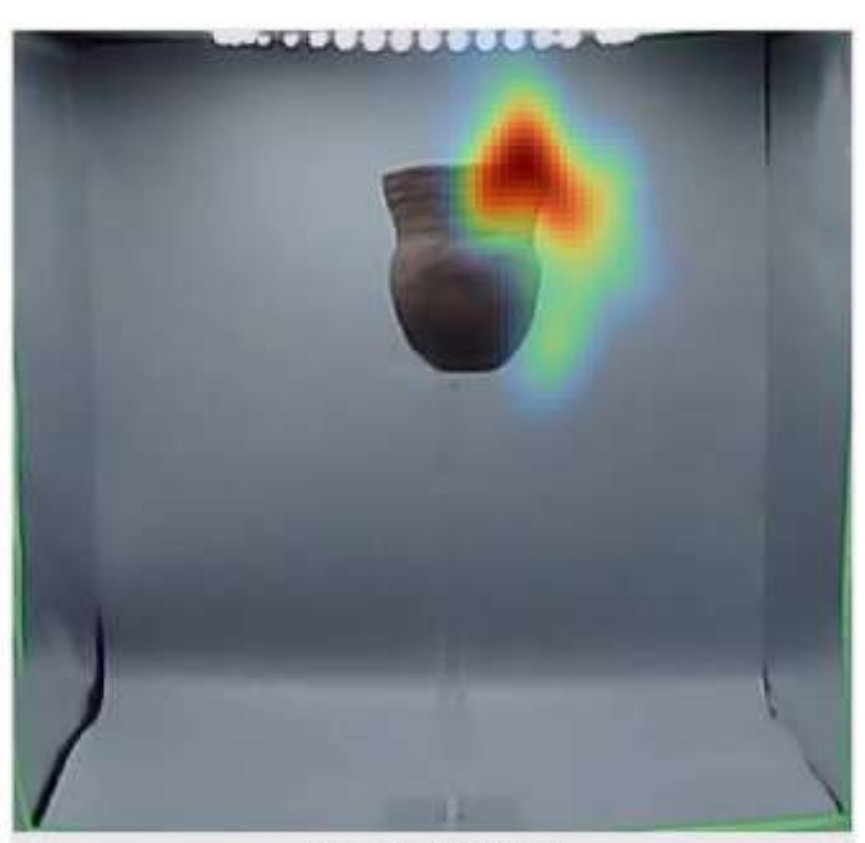
Part0108

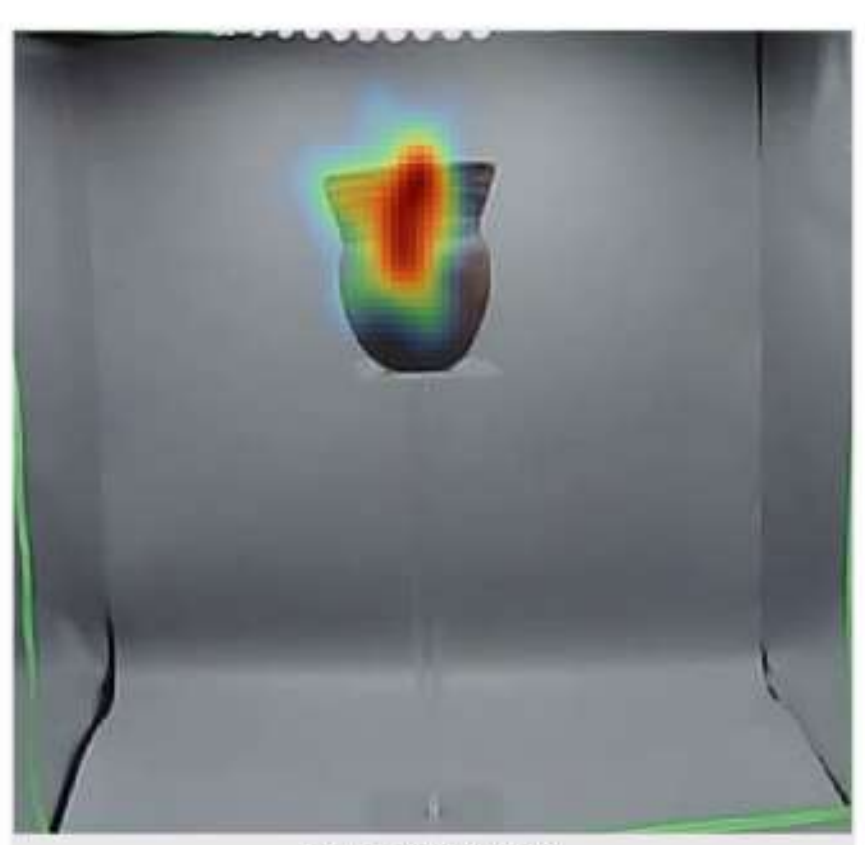
Part0109

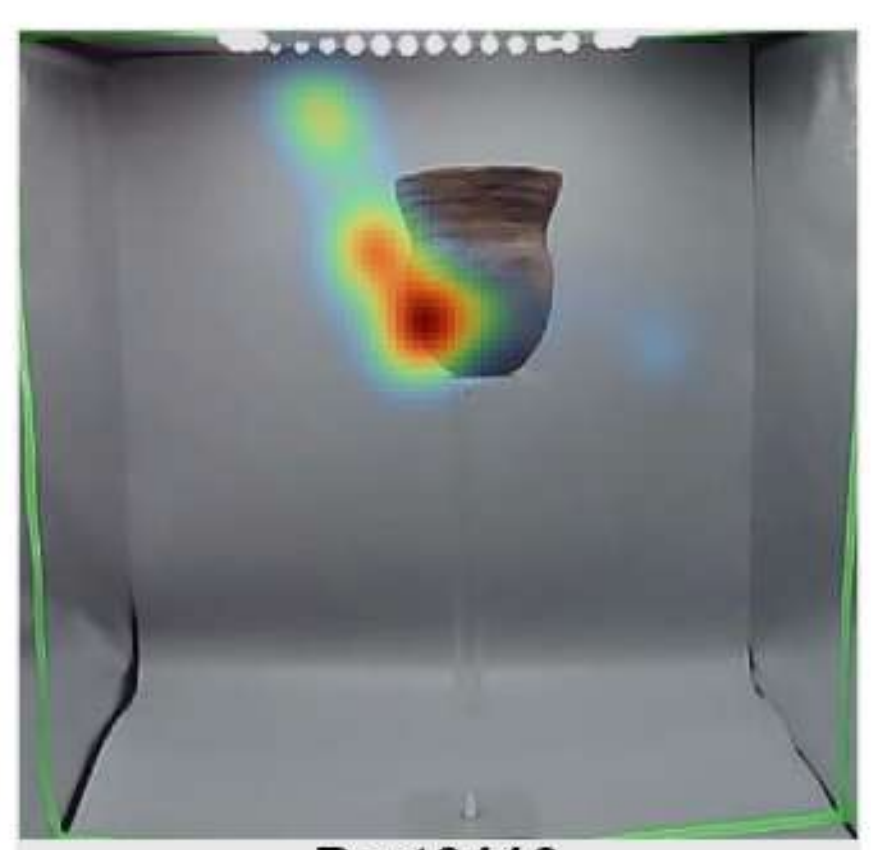
Part0110

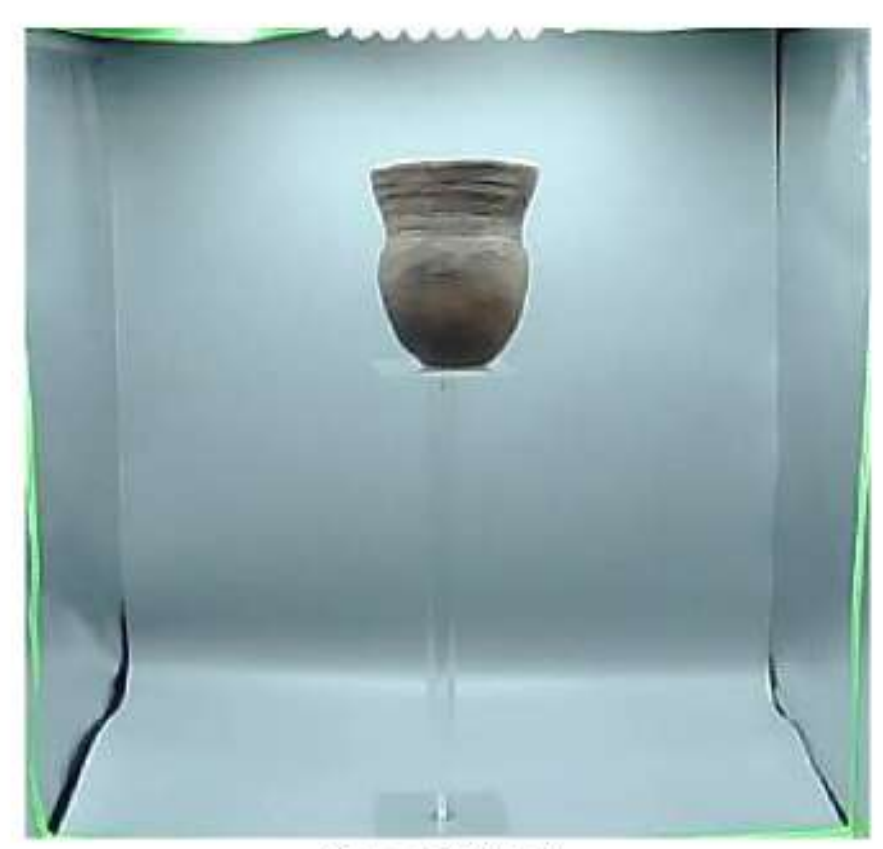
Part0111

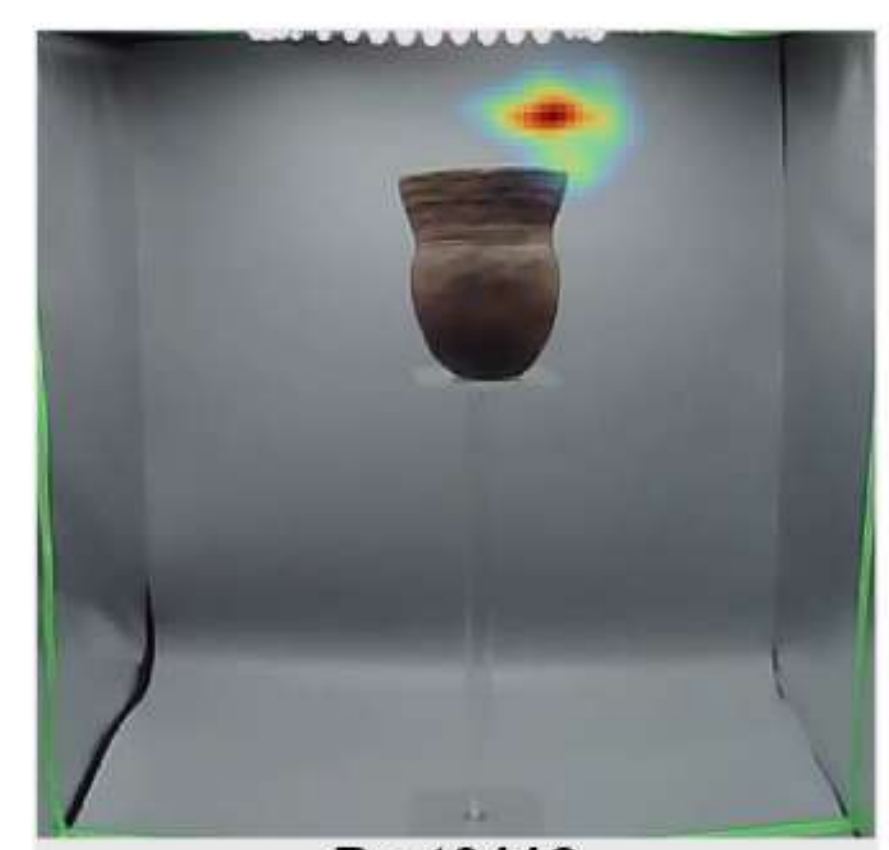
Part0112

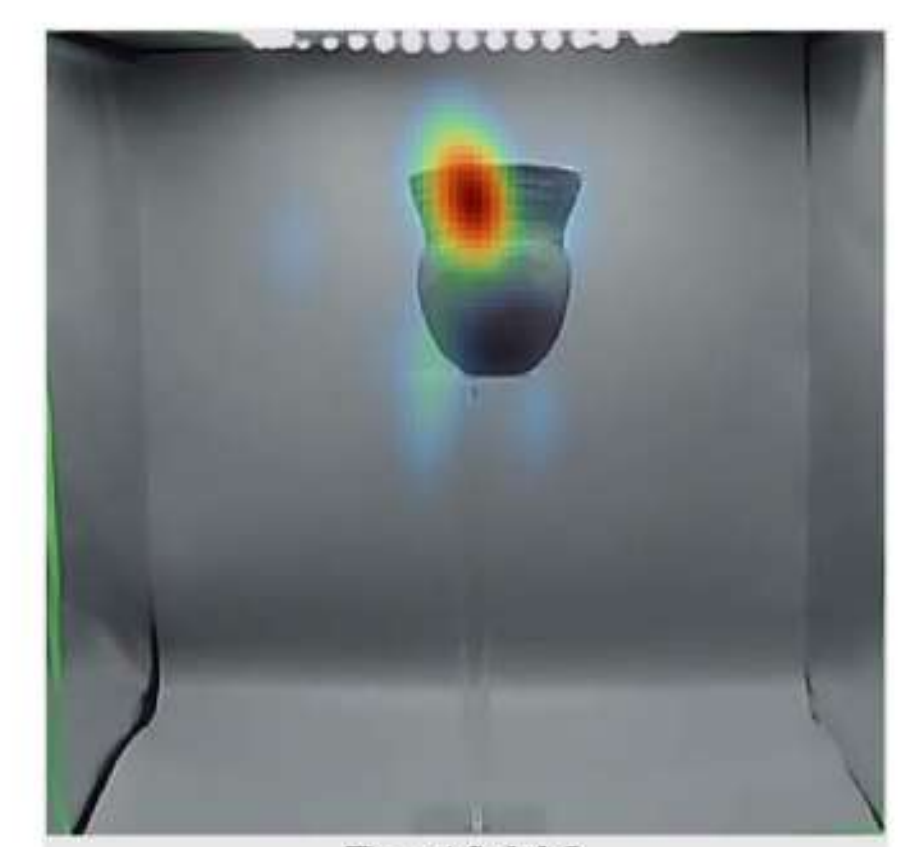
Part0113

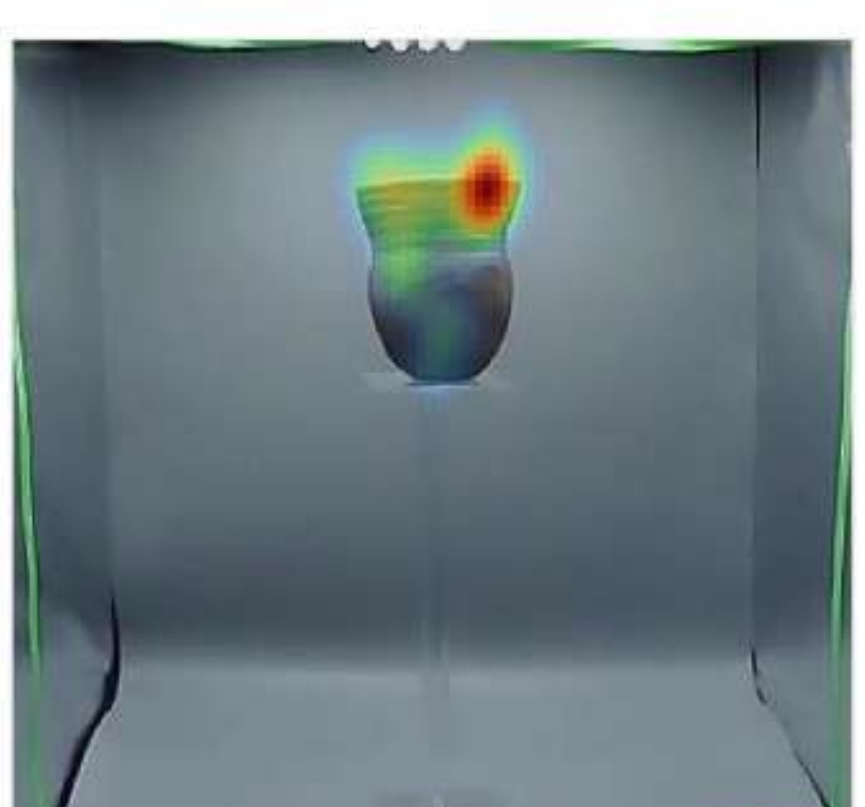
Part0114

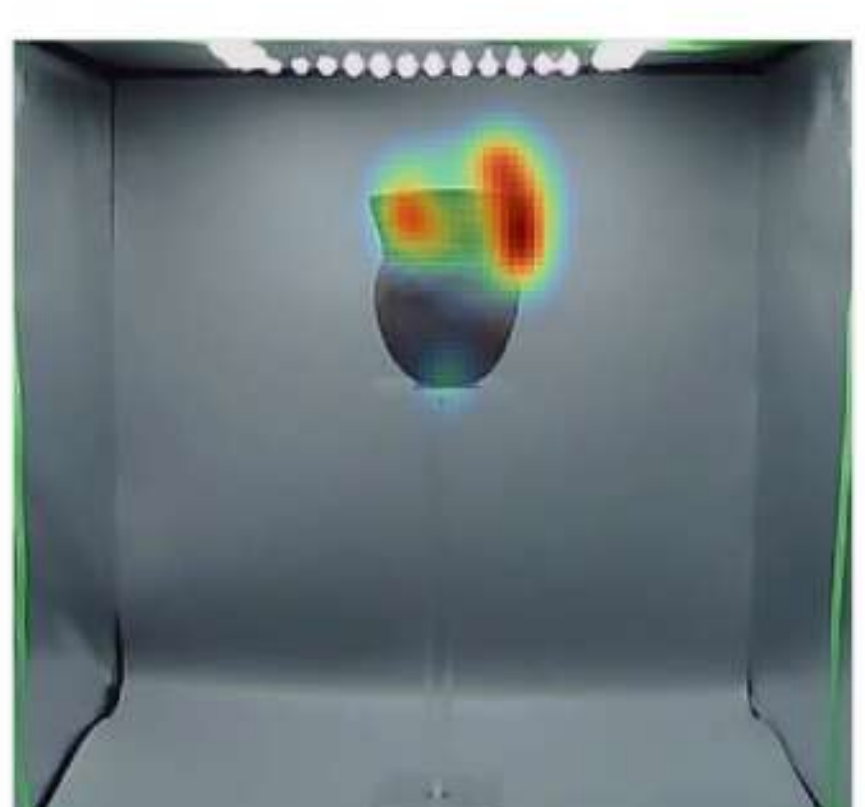
Part0115

Obj0250

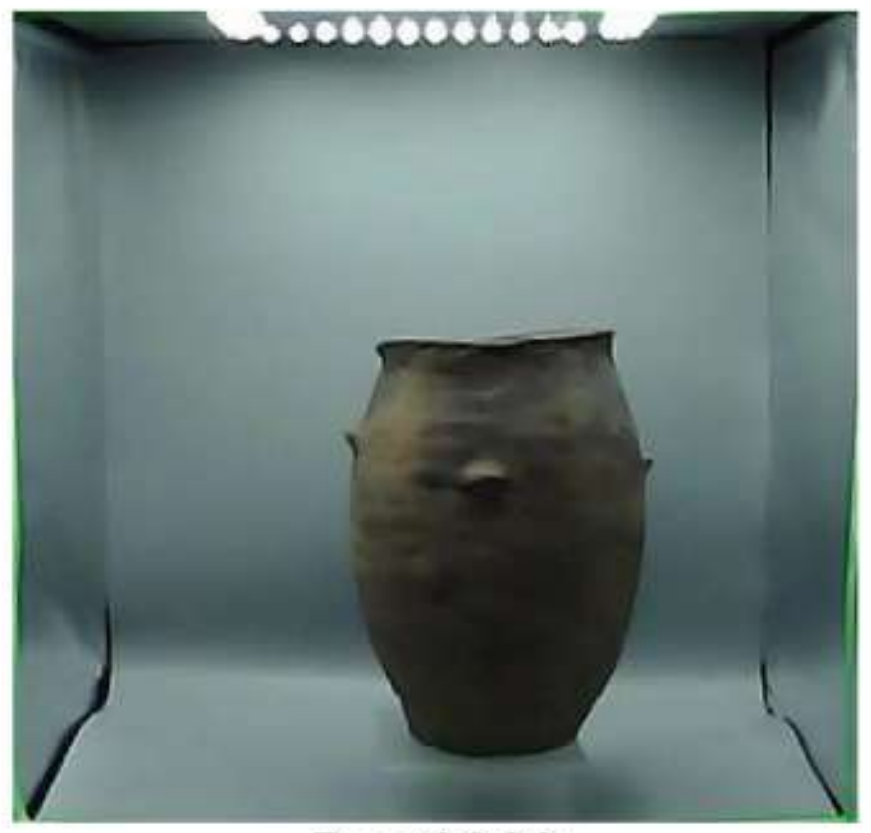
Part0089

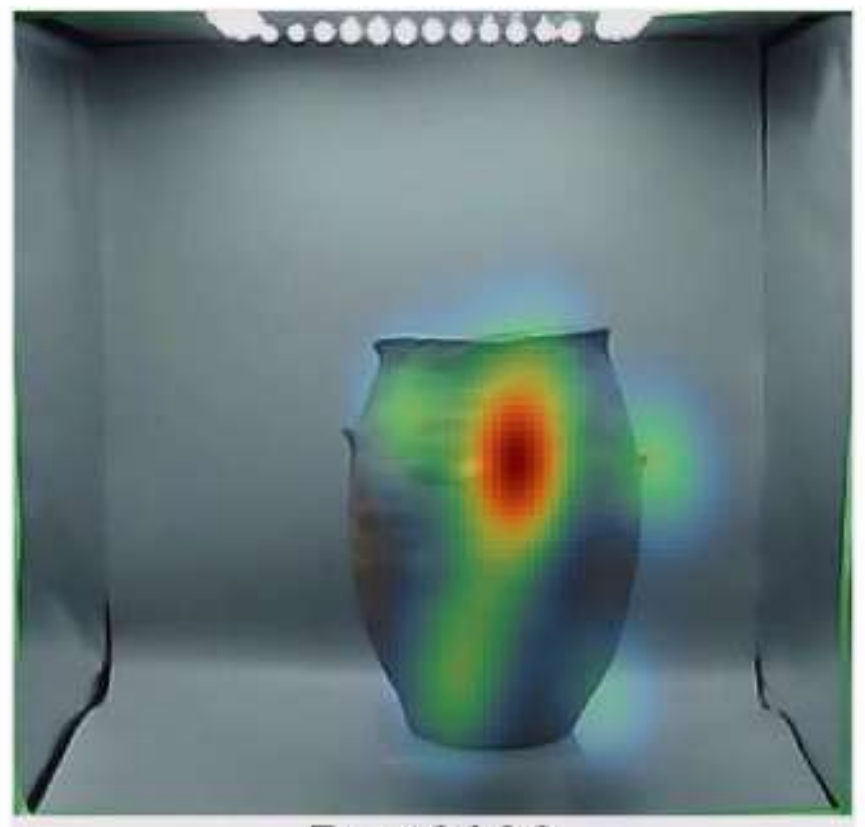
Part0090

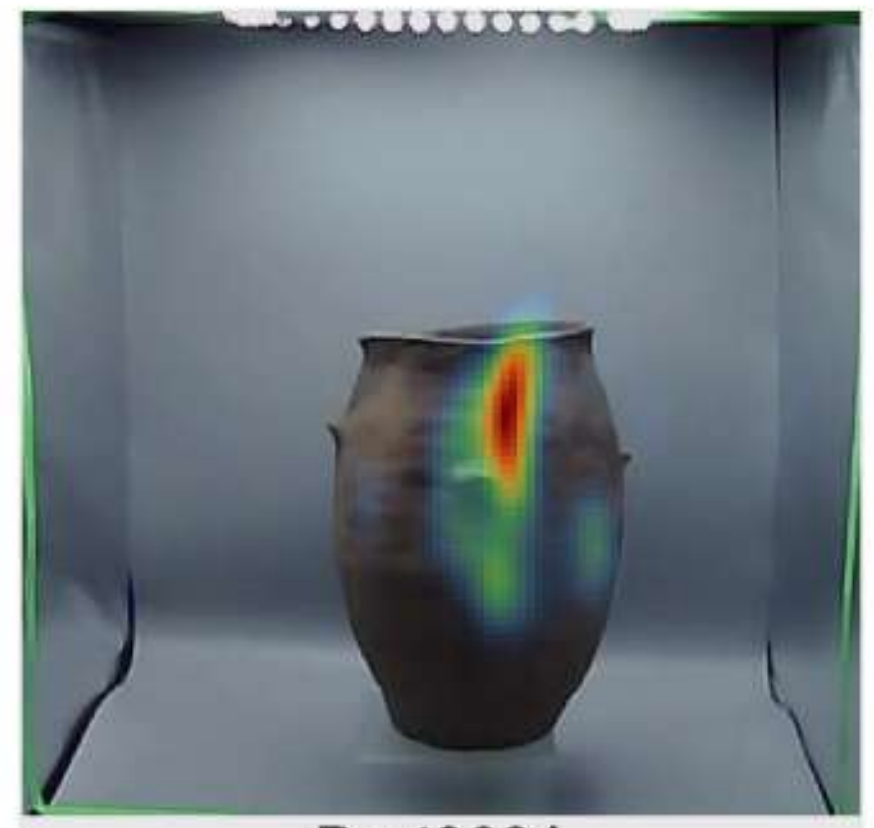
Part0091

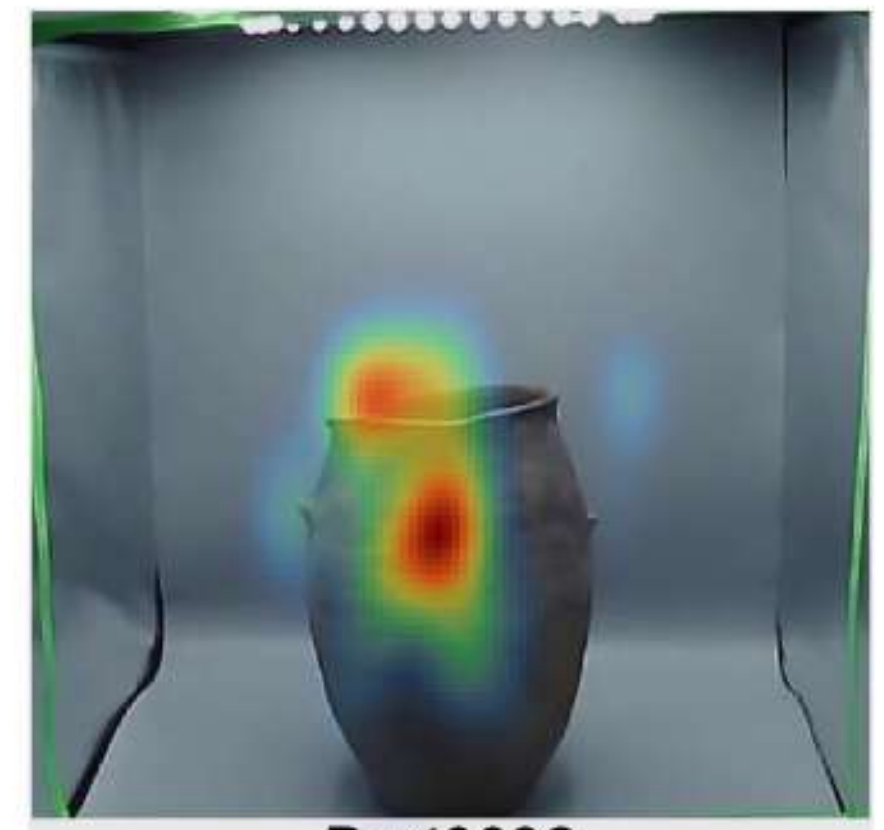
Part0092

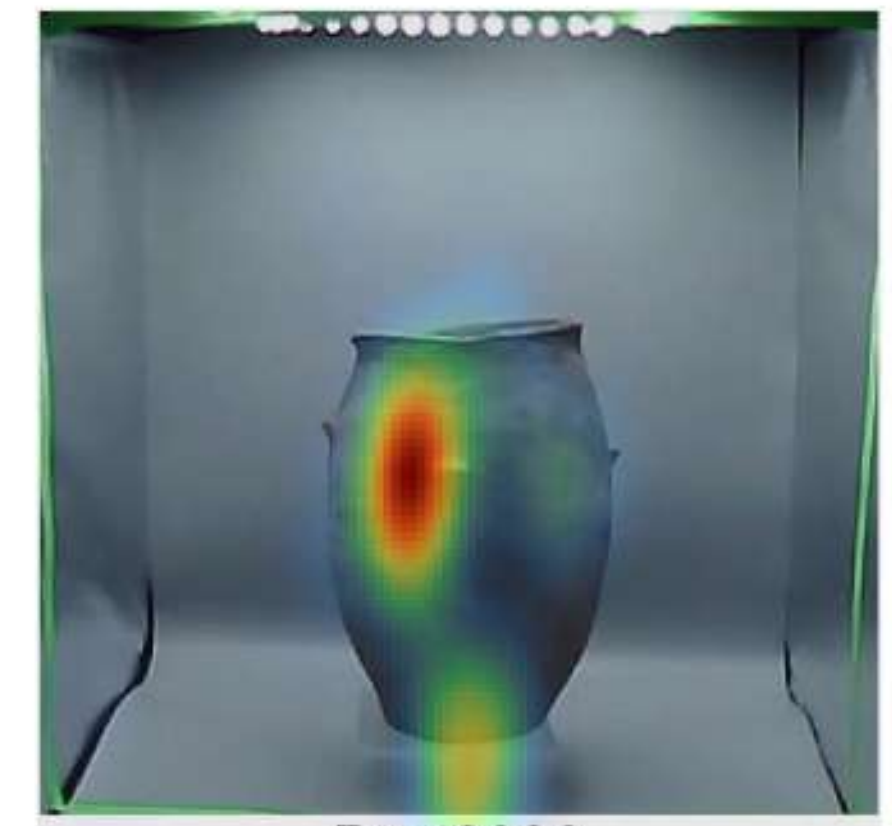
Part0093

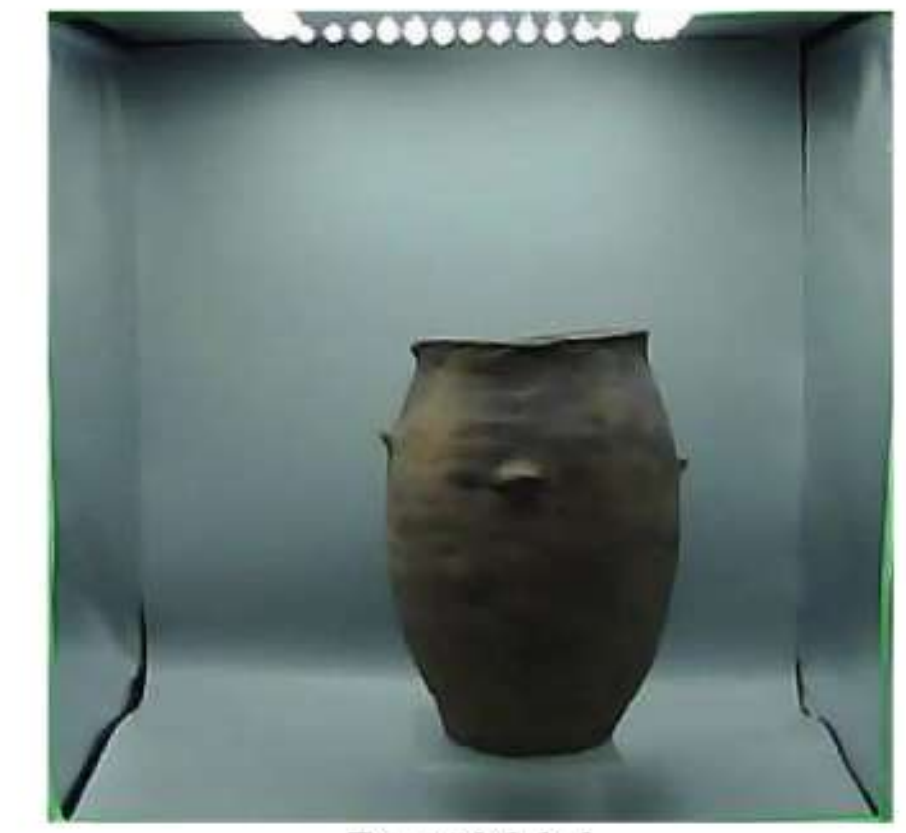
Part0094

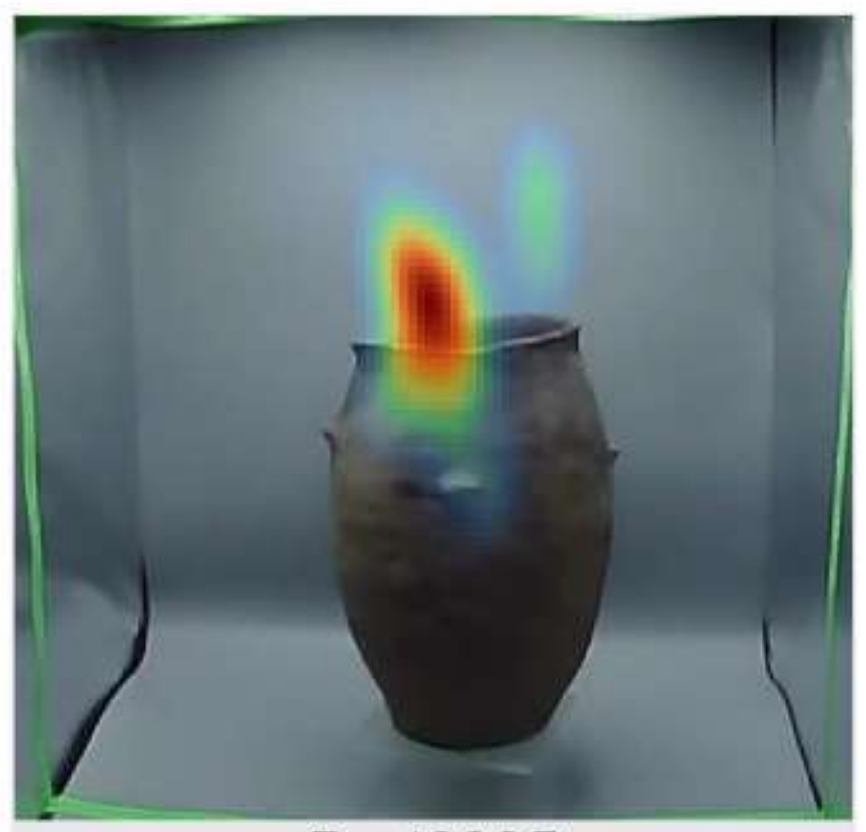
Part0095

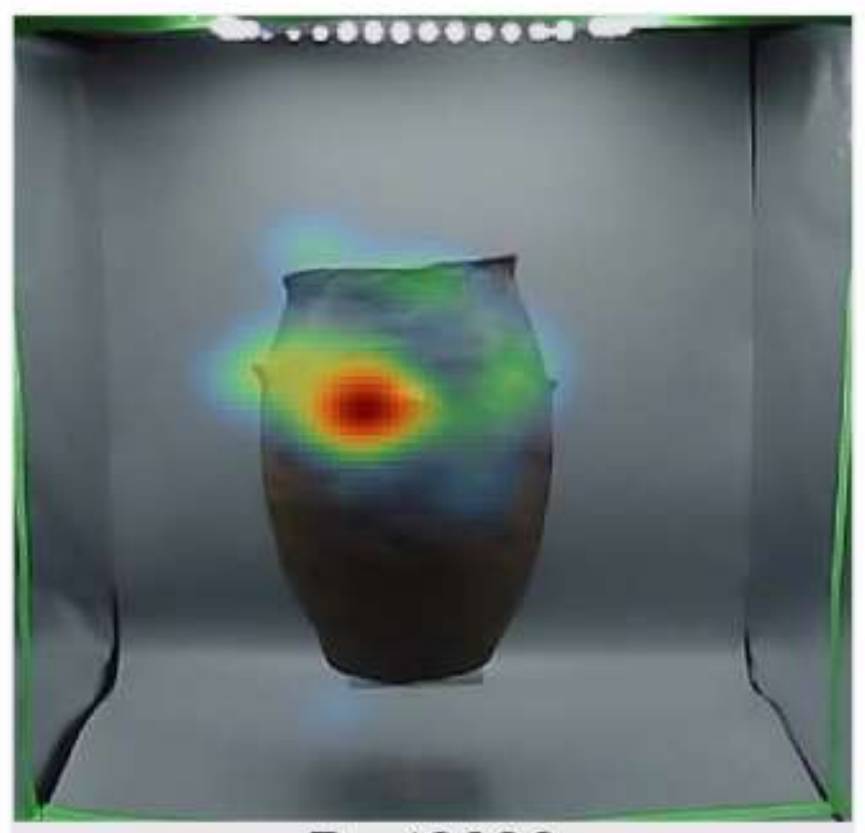
Part0096

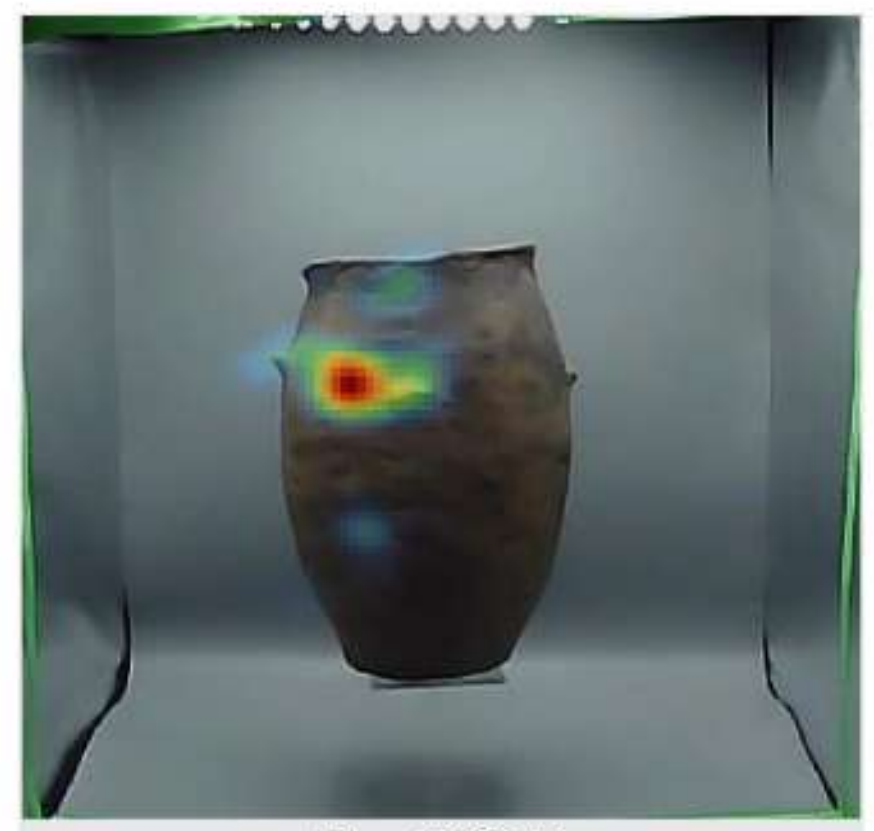
Part0097

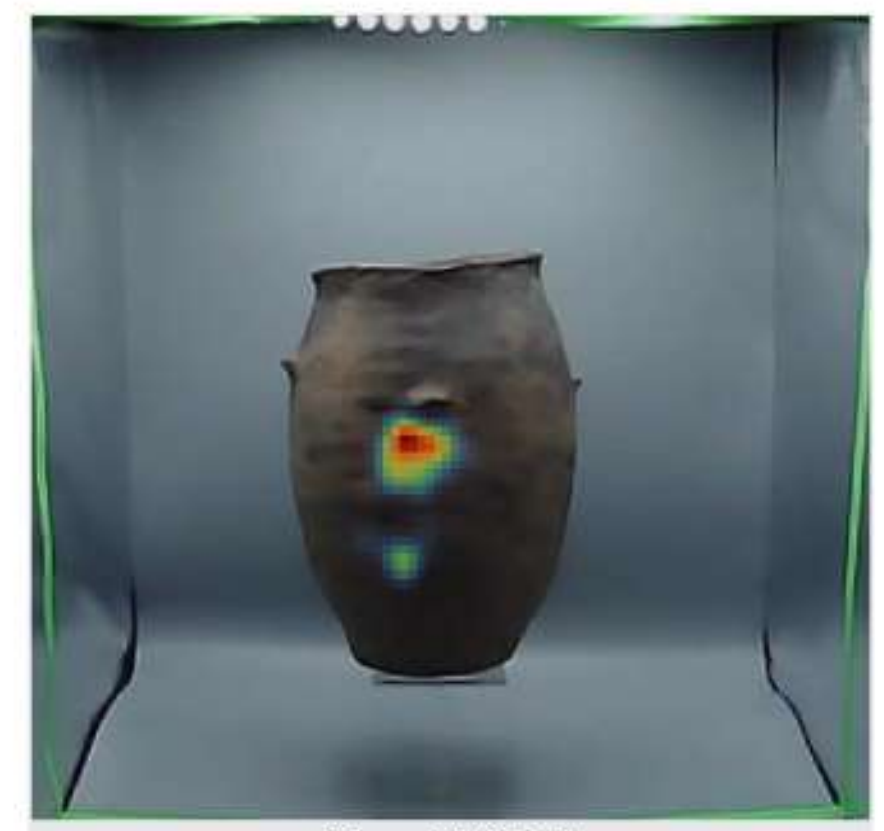
Part0098

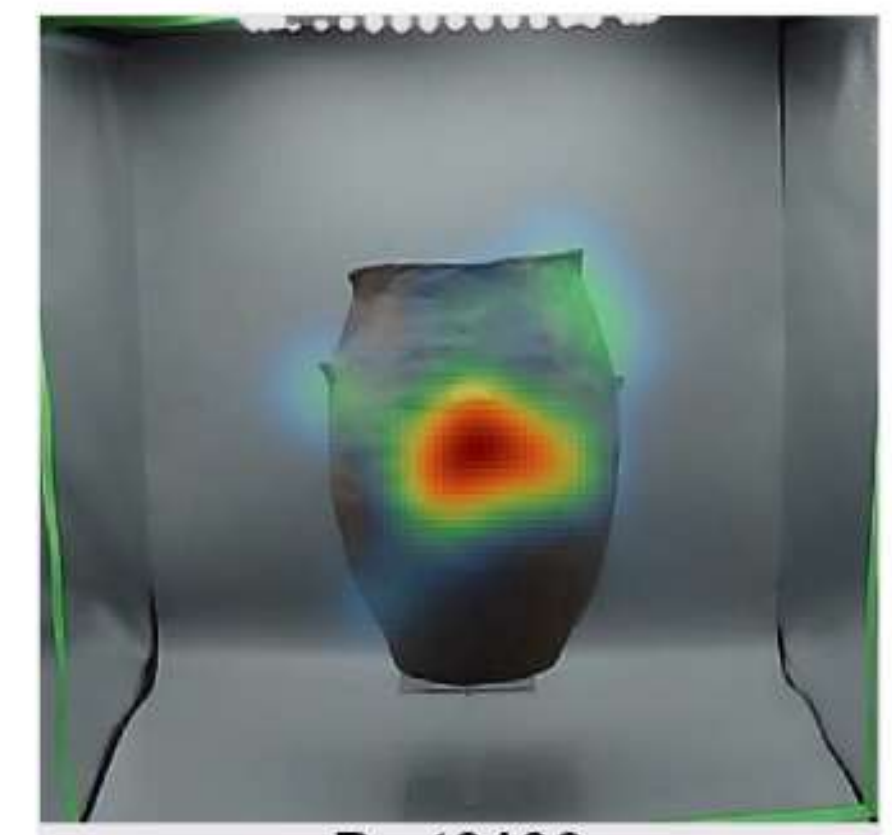
Part0100

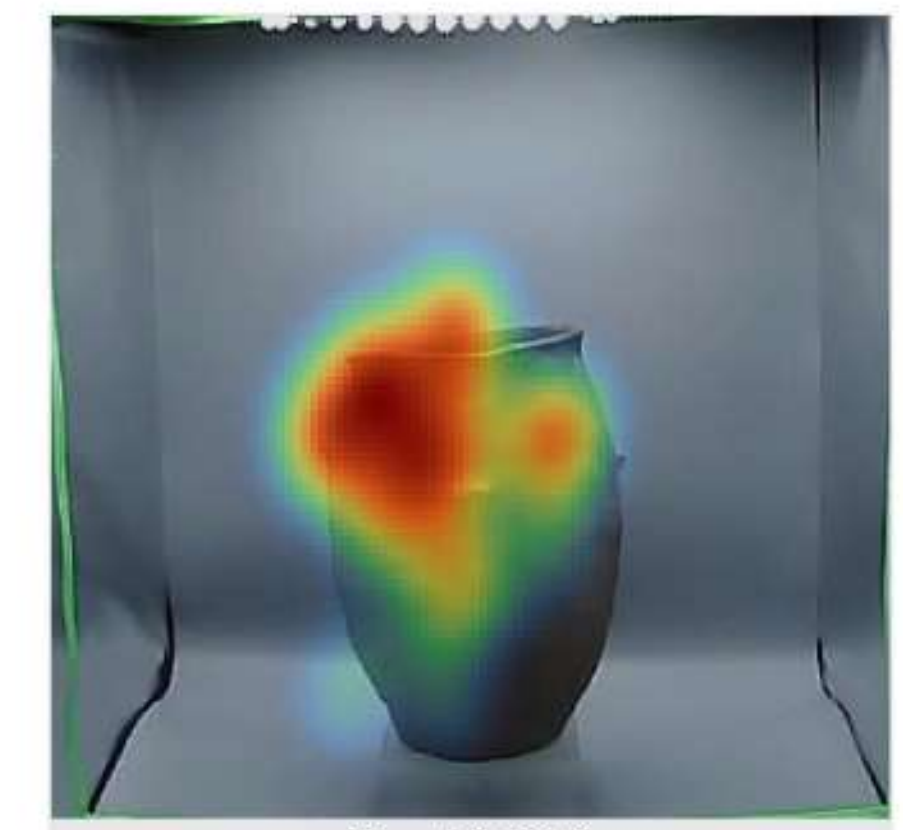
Part0101

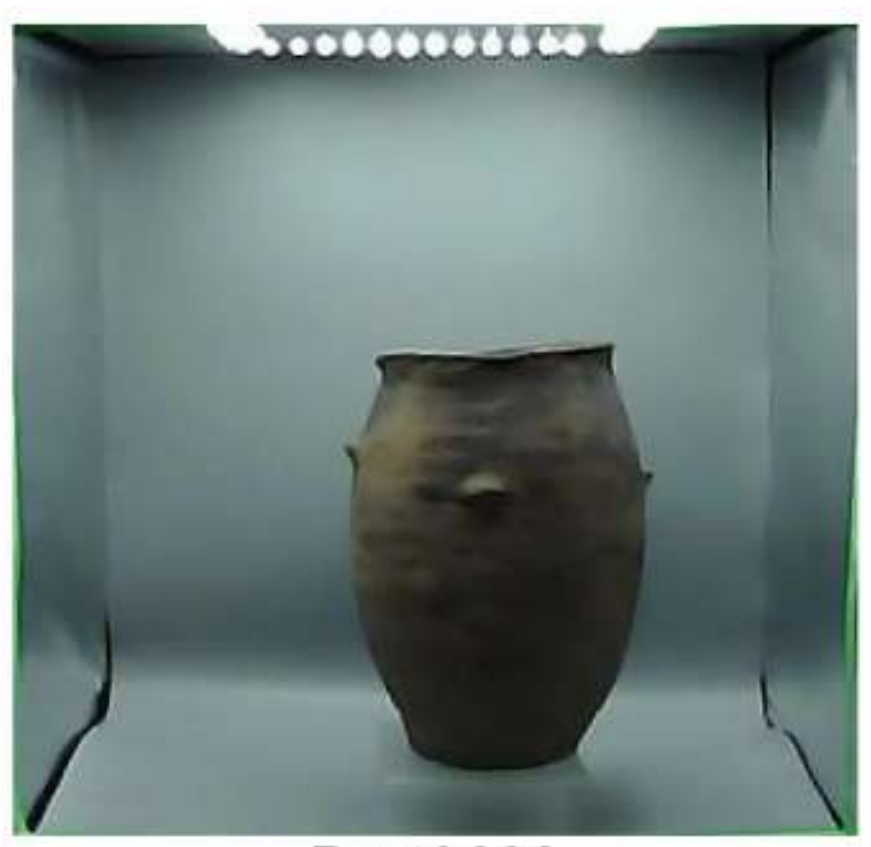
Part0102

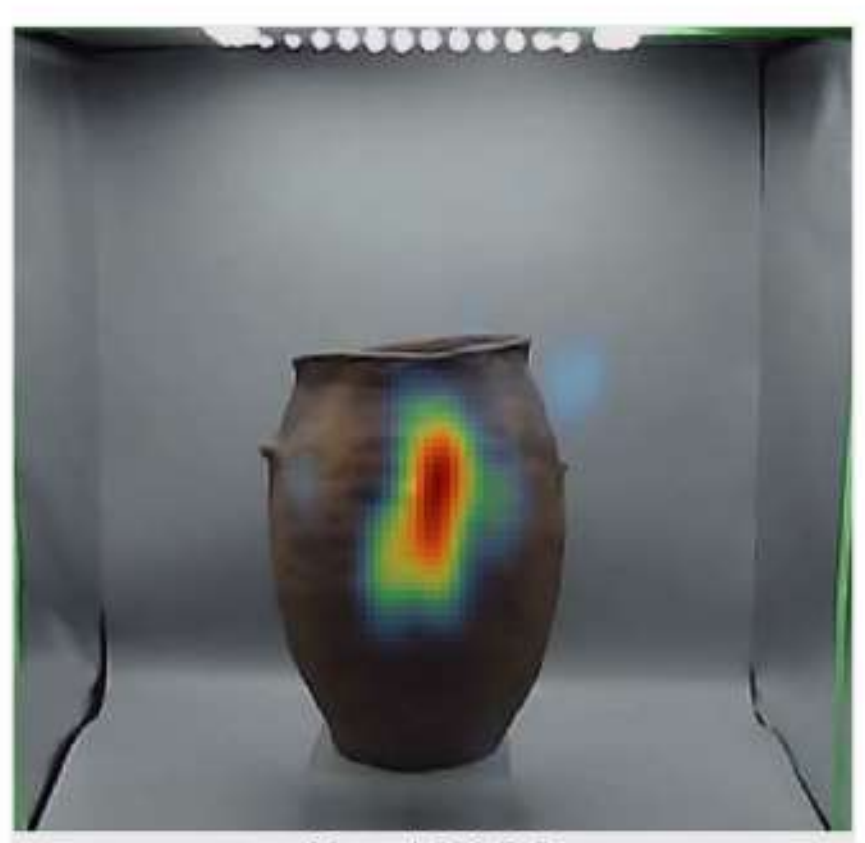
Part0103

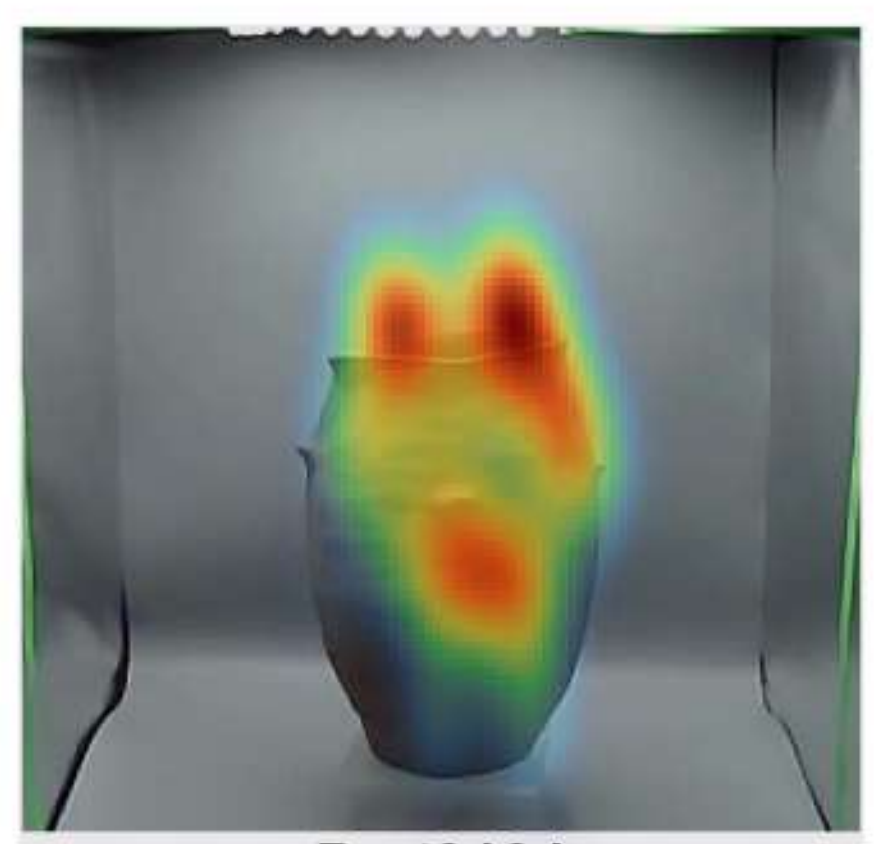
Part0104

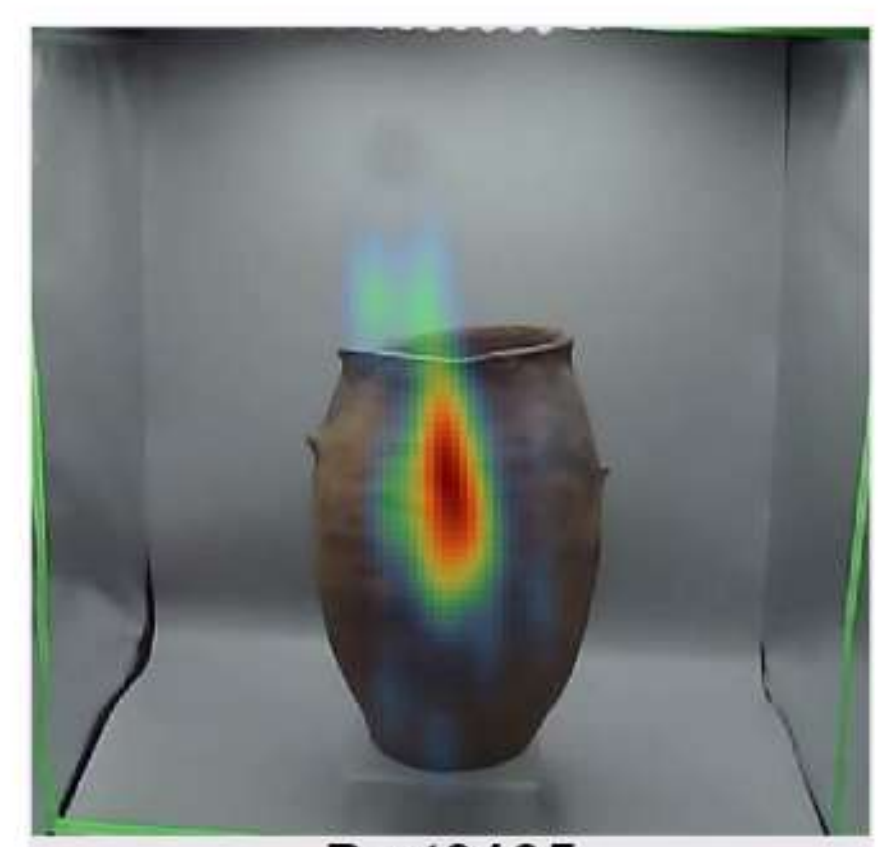
Part0105

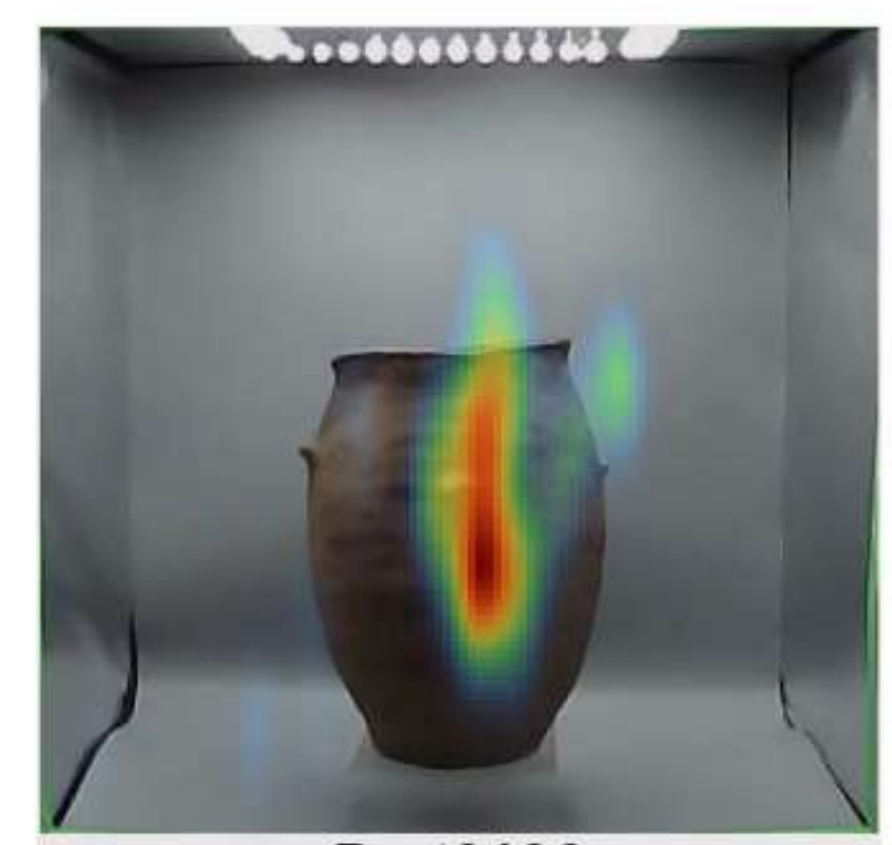
Part0106

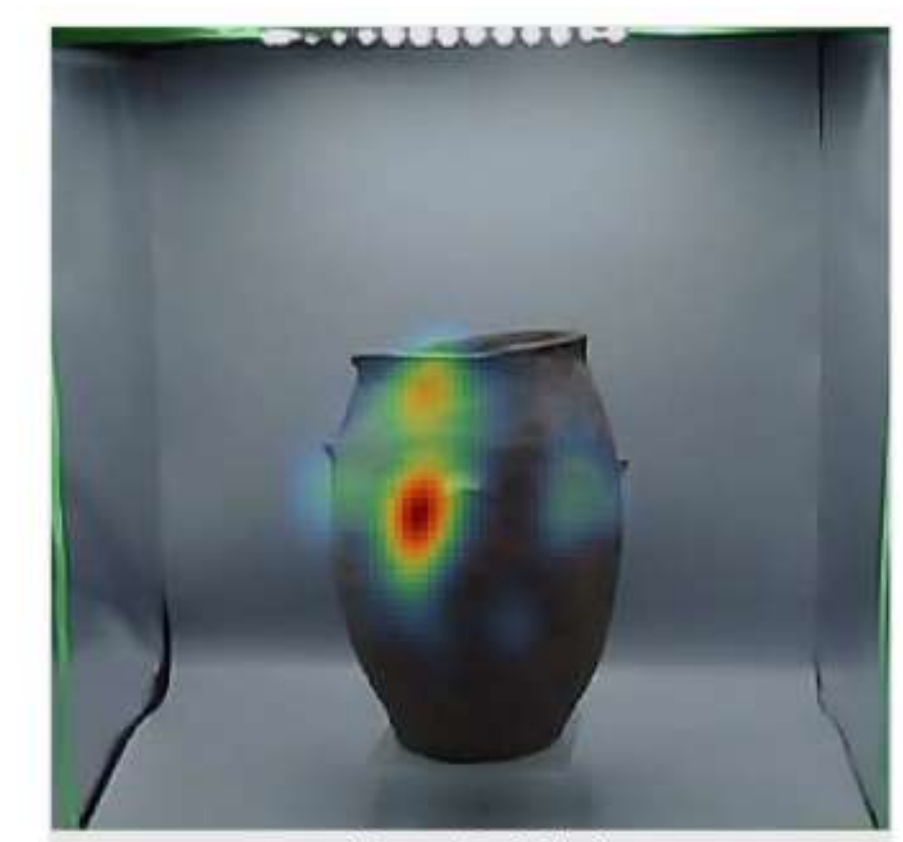
Part0107

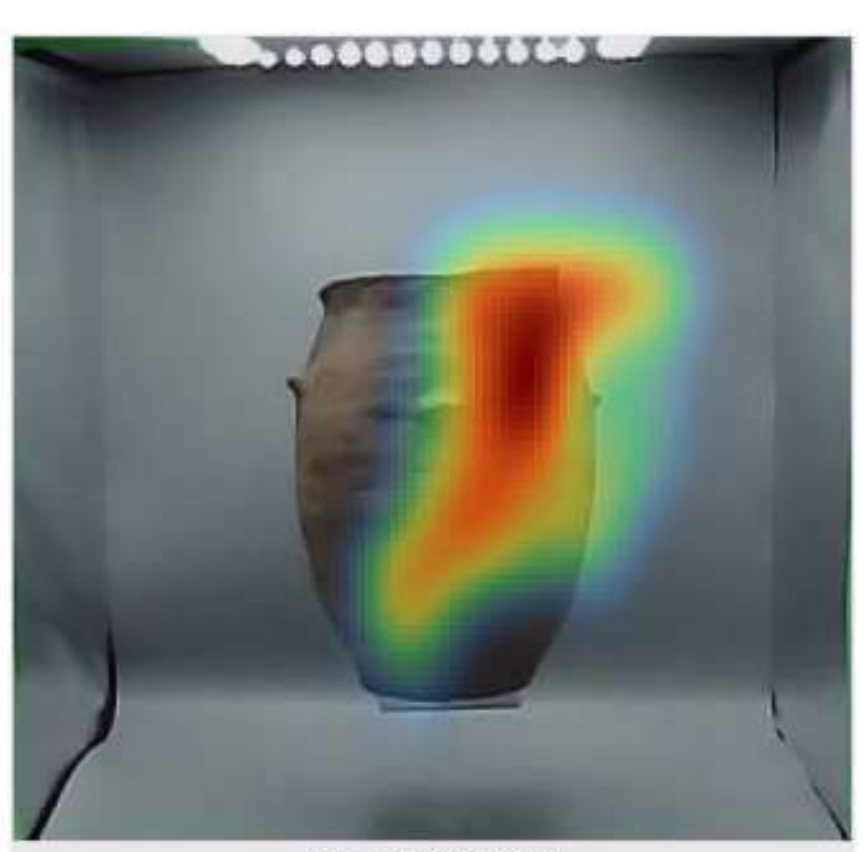
Part0108

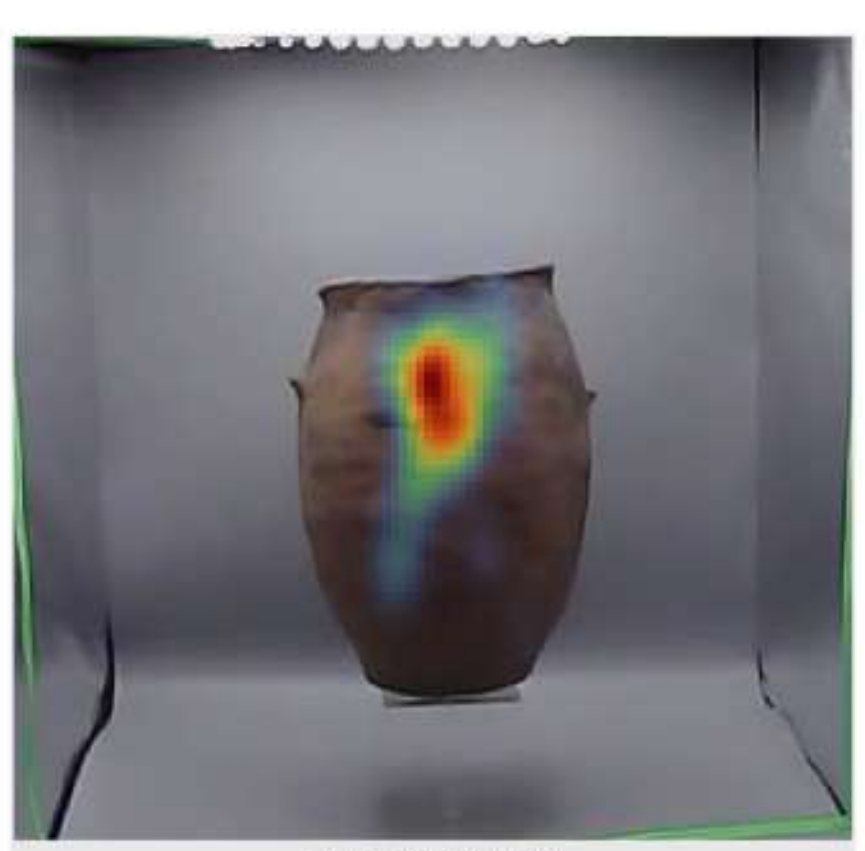
Part0109

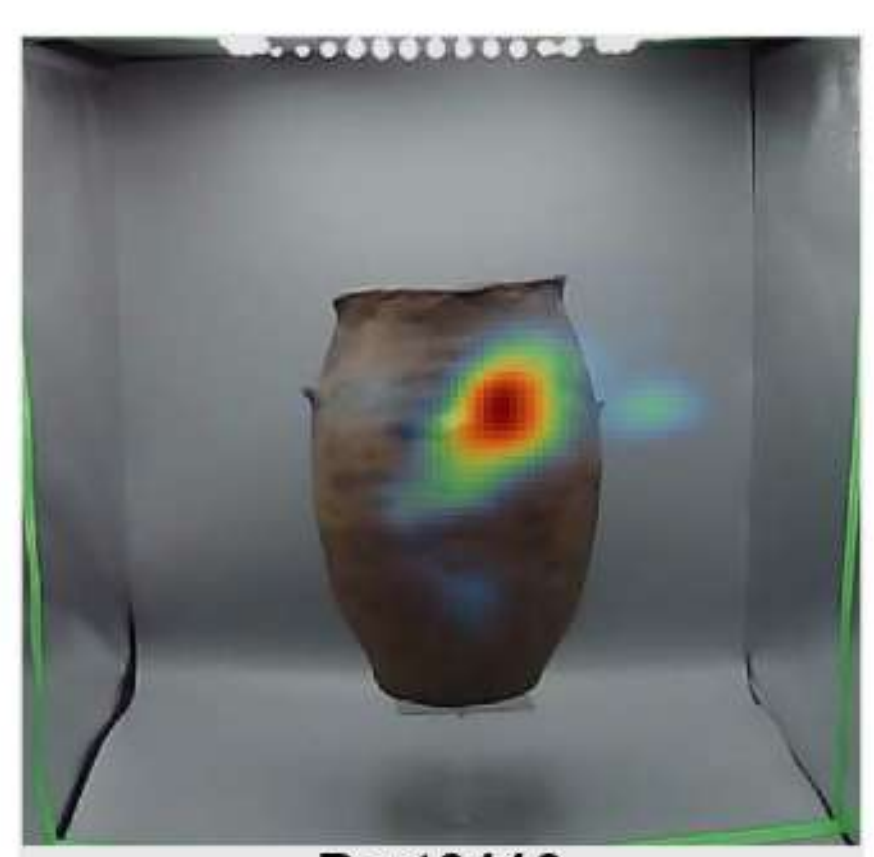
Part0110

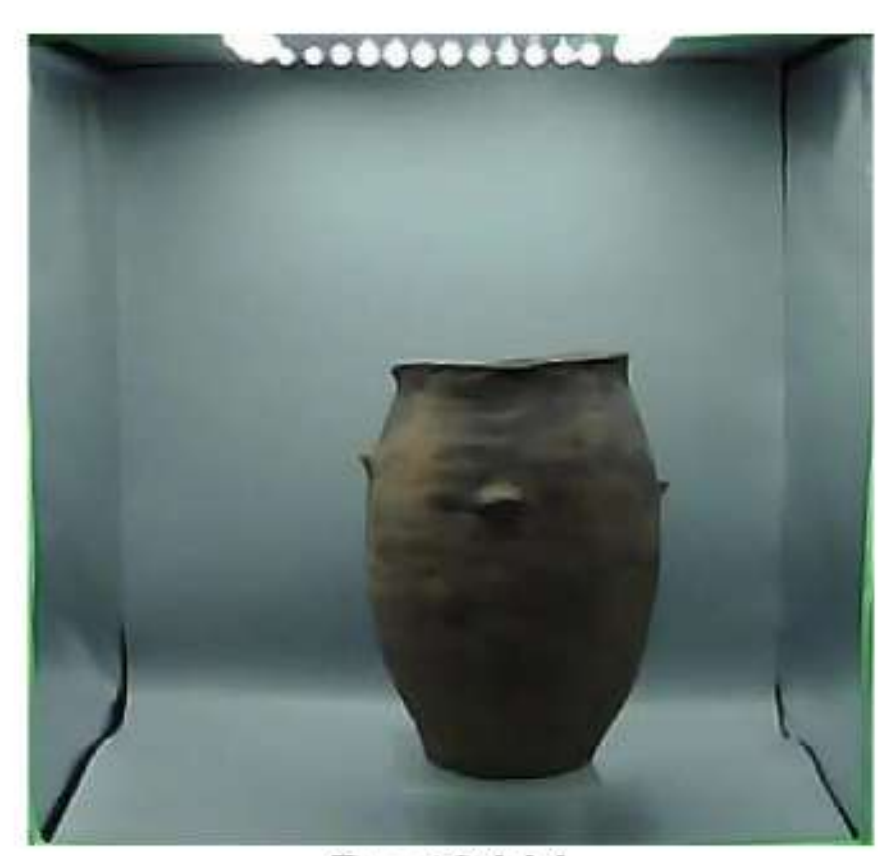
Part0111

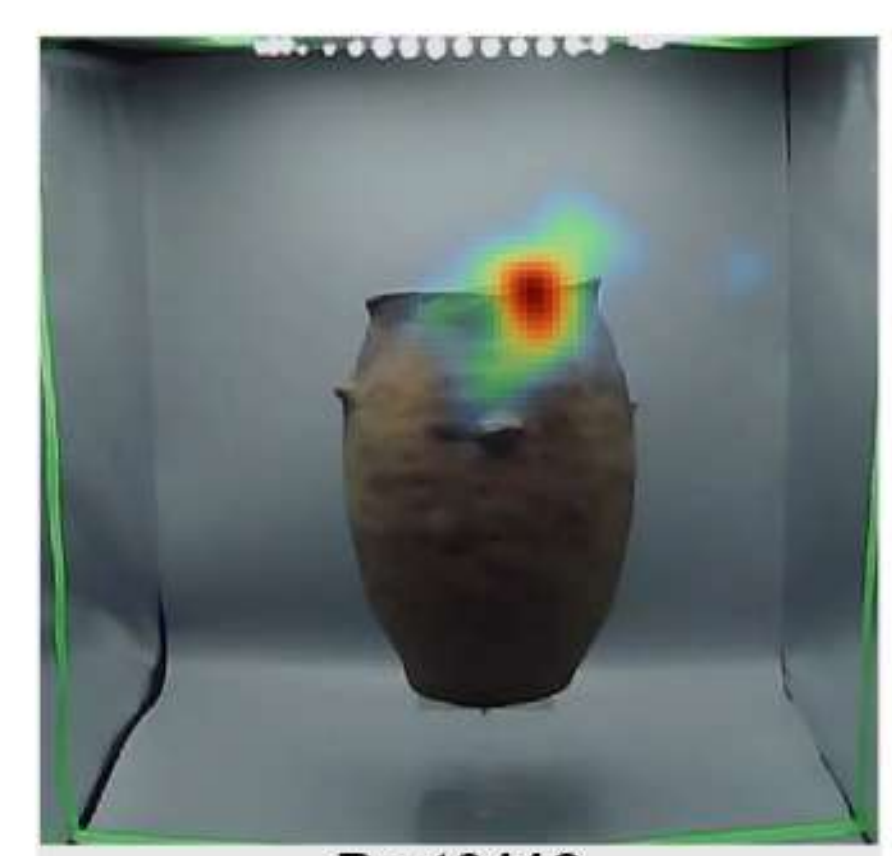
Part0112

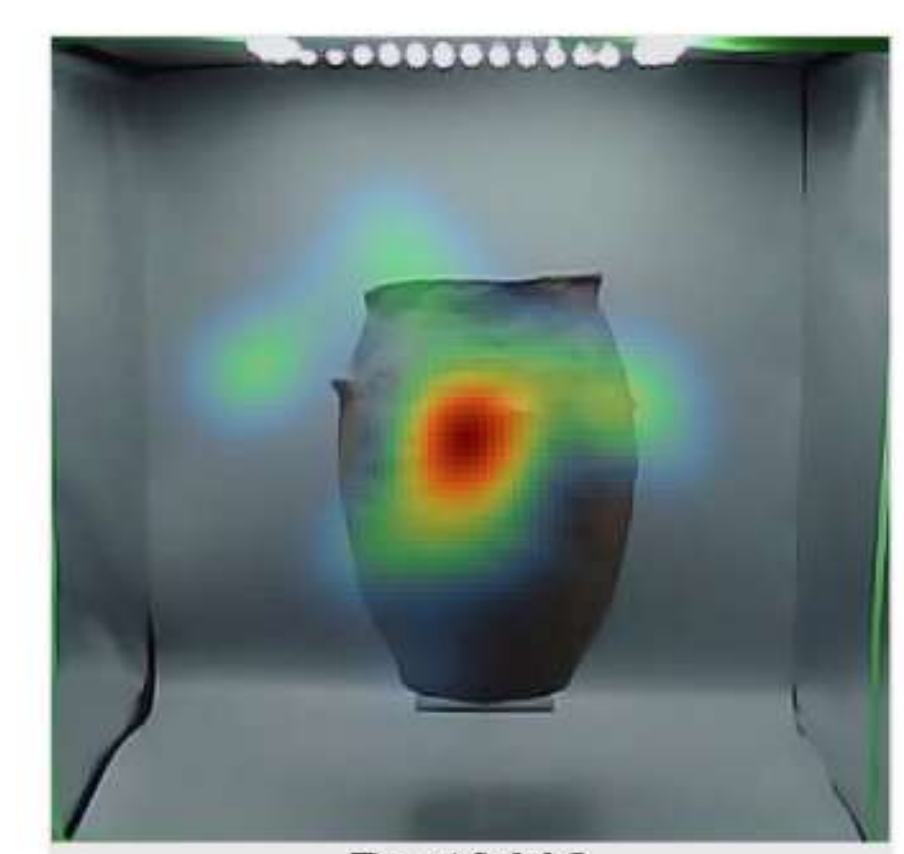
Part0113

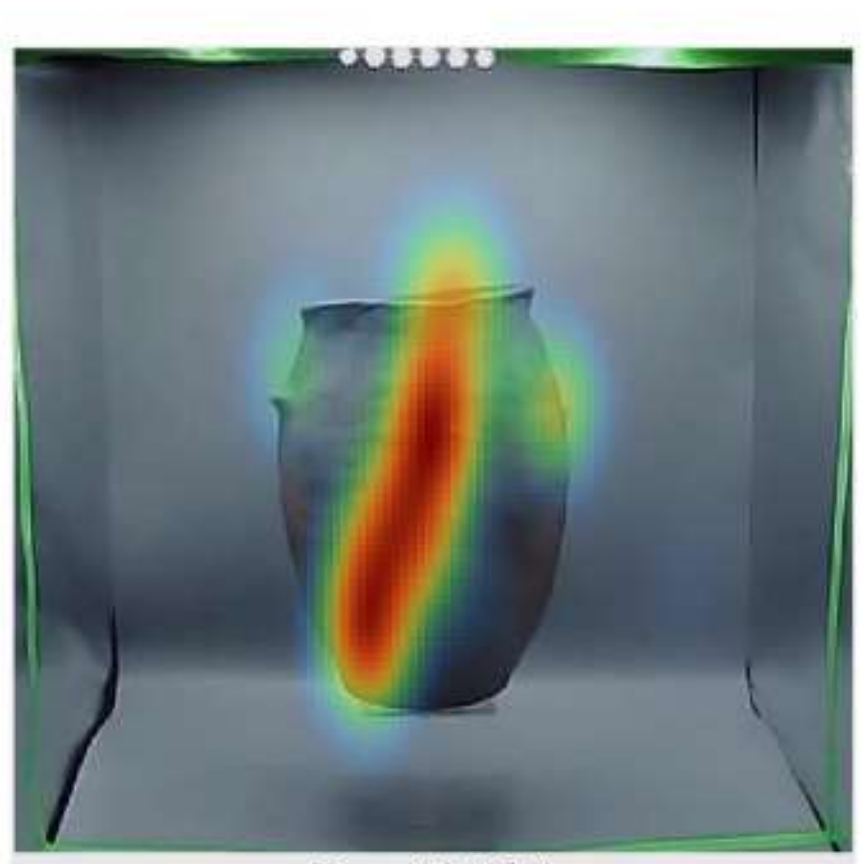
Part0114

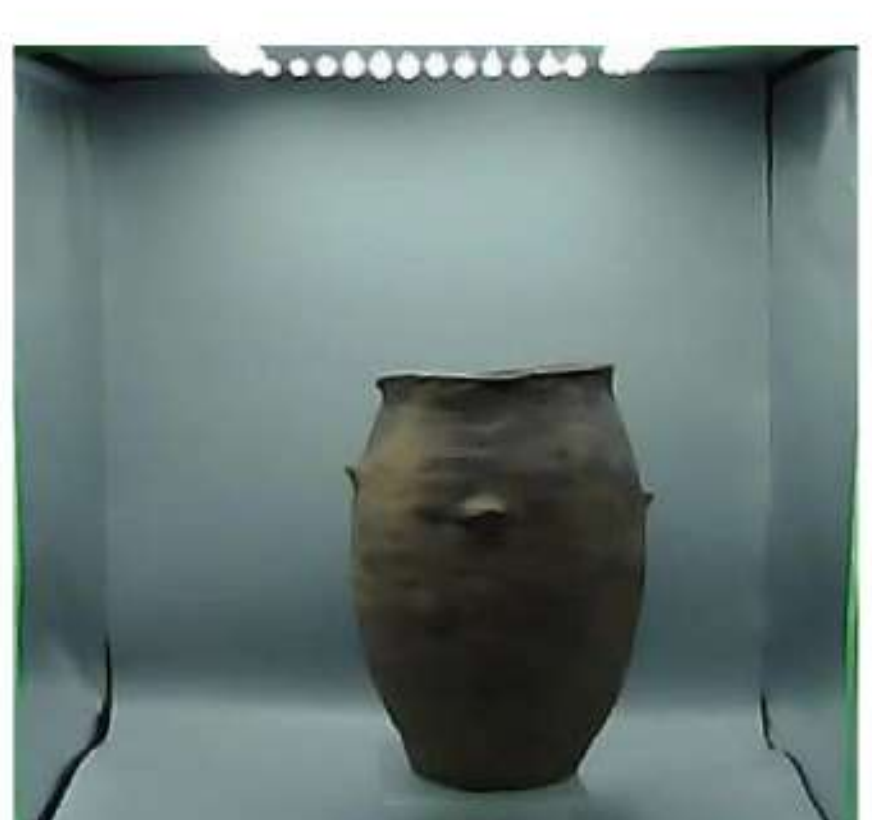
Part0115

Obj0252

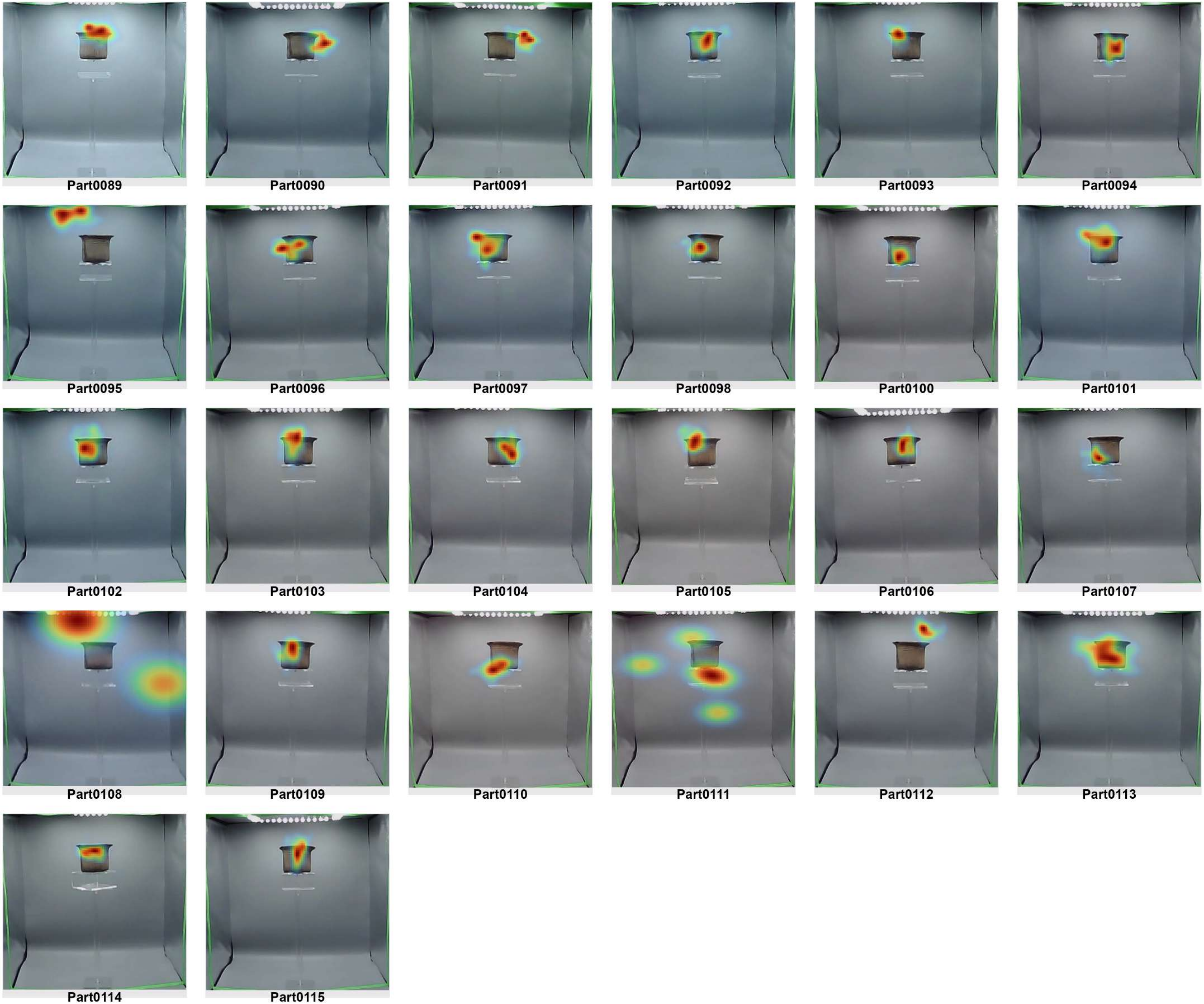
Part0089
Part0090
Part0091
Part0092
Part0093
Part0094
Part0095
Part0096
Part0097
Part0098
Part0100
Part0101
Part0102
Part0103
Part0104
Part0105
Part0106
Part0107
Part0108
Part0109
Part0110
Part0111
Part0112
Part0113
Part0114
Part0115

Obj0254

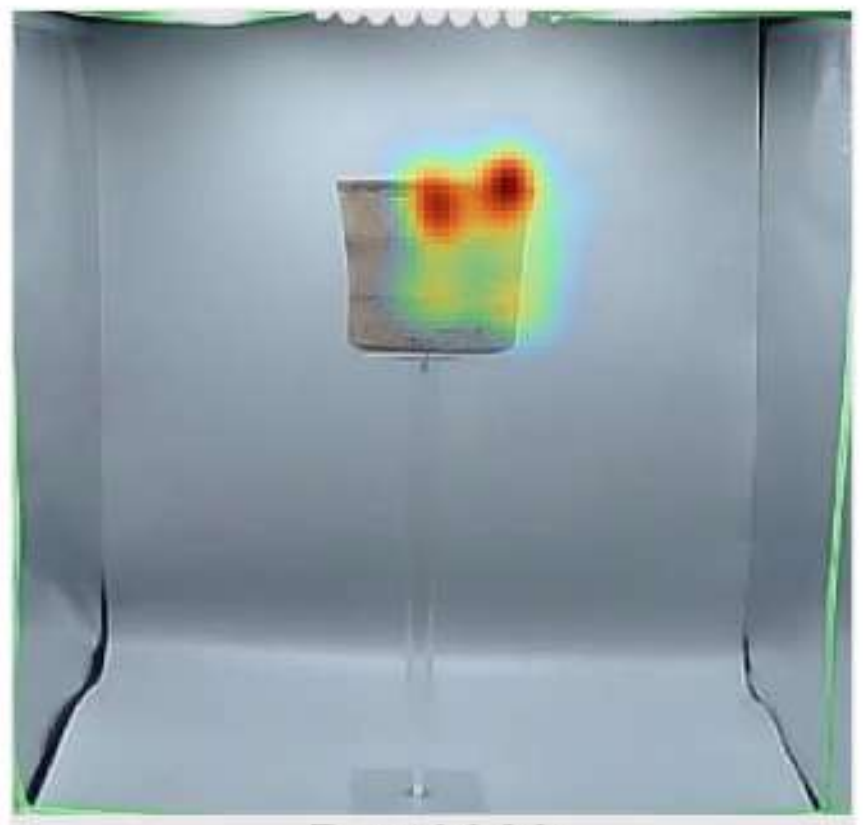
Part0089

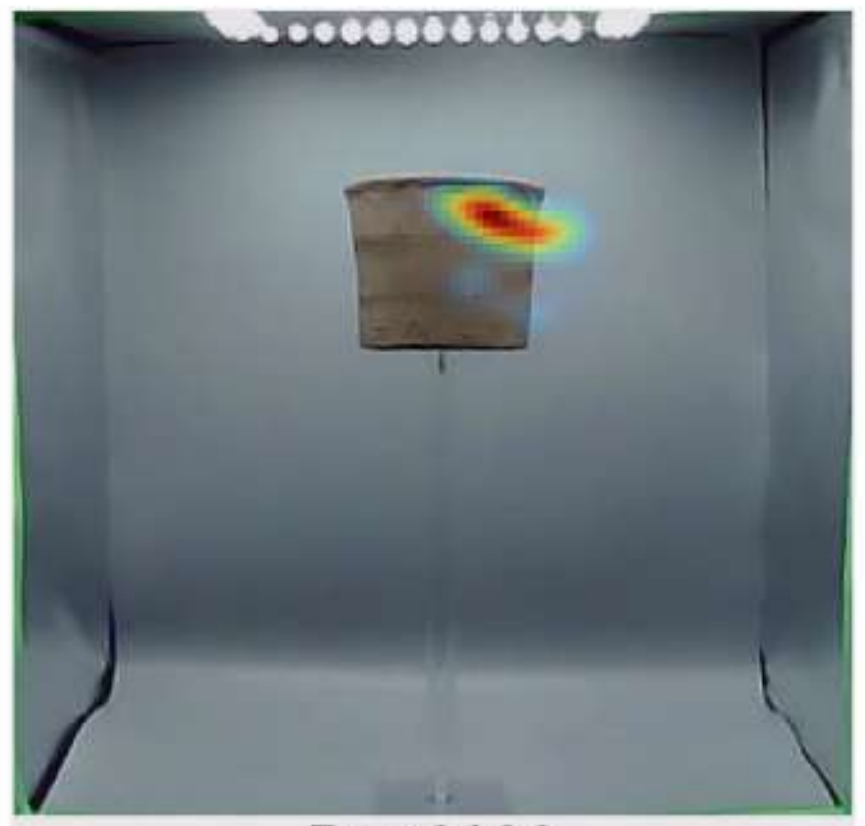
Part0090

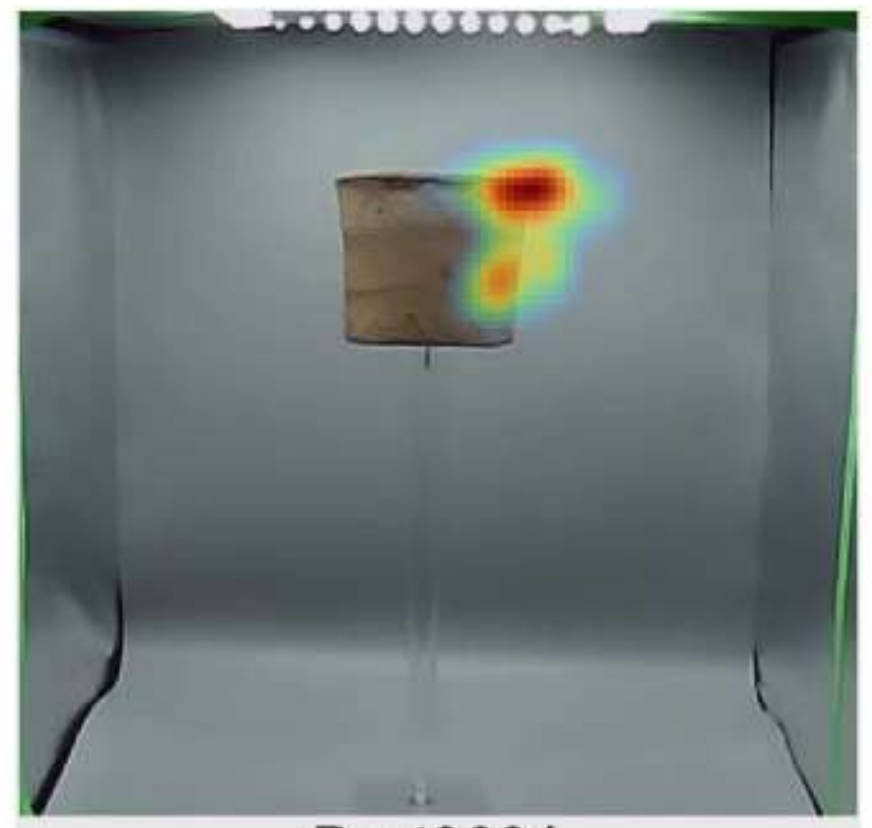
Part0091

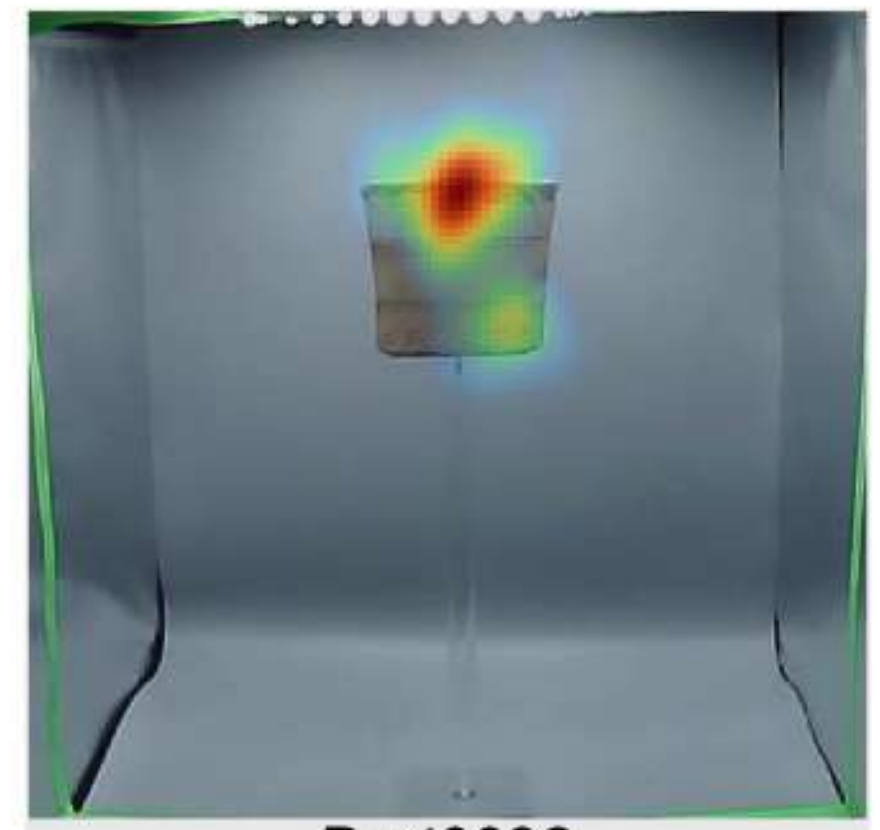
Part0092

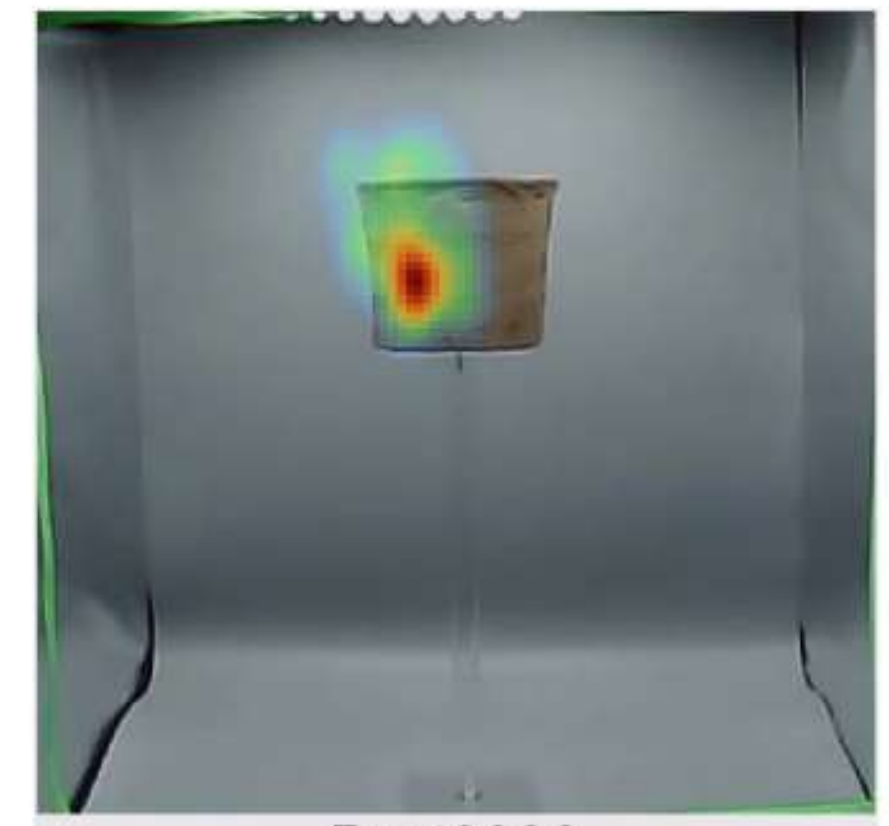
Part0093

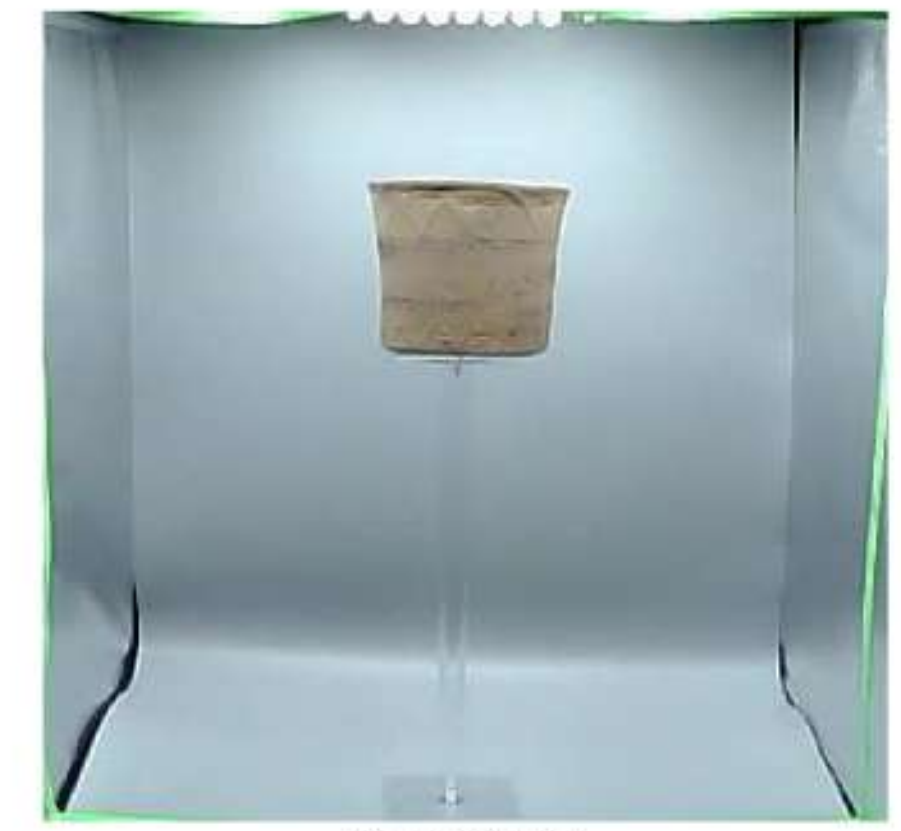
Part0094

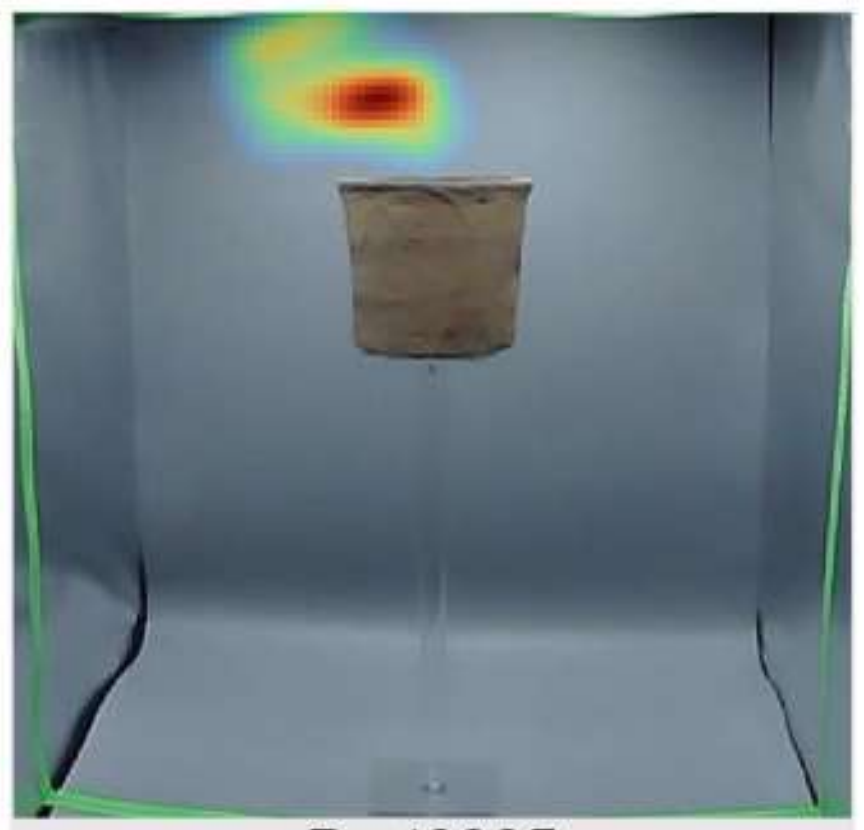
Part0095

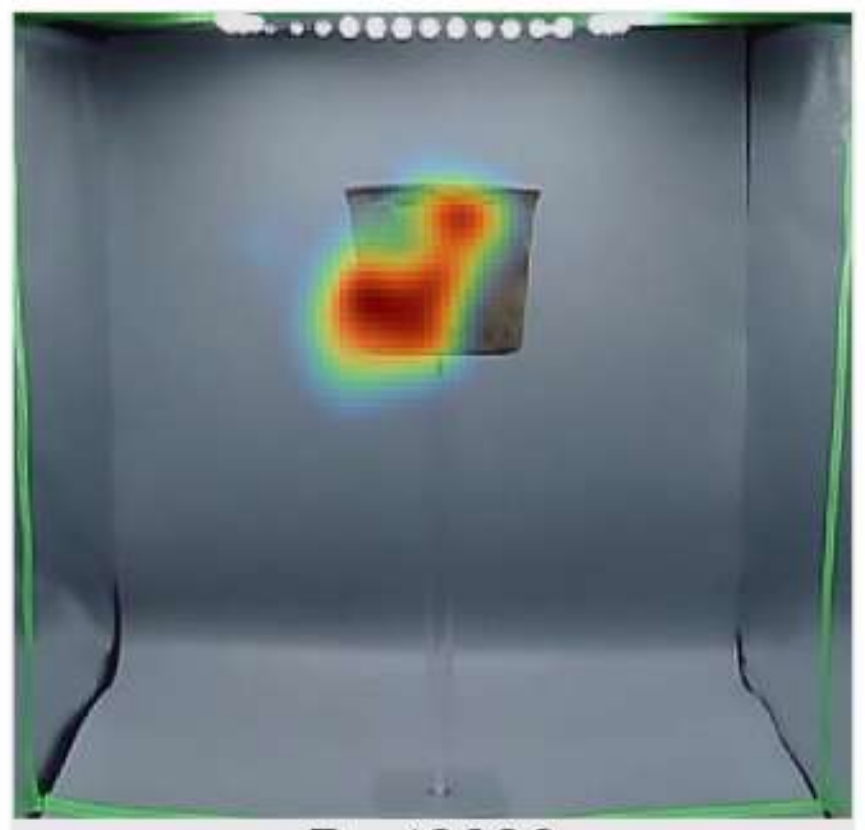
Part0096

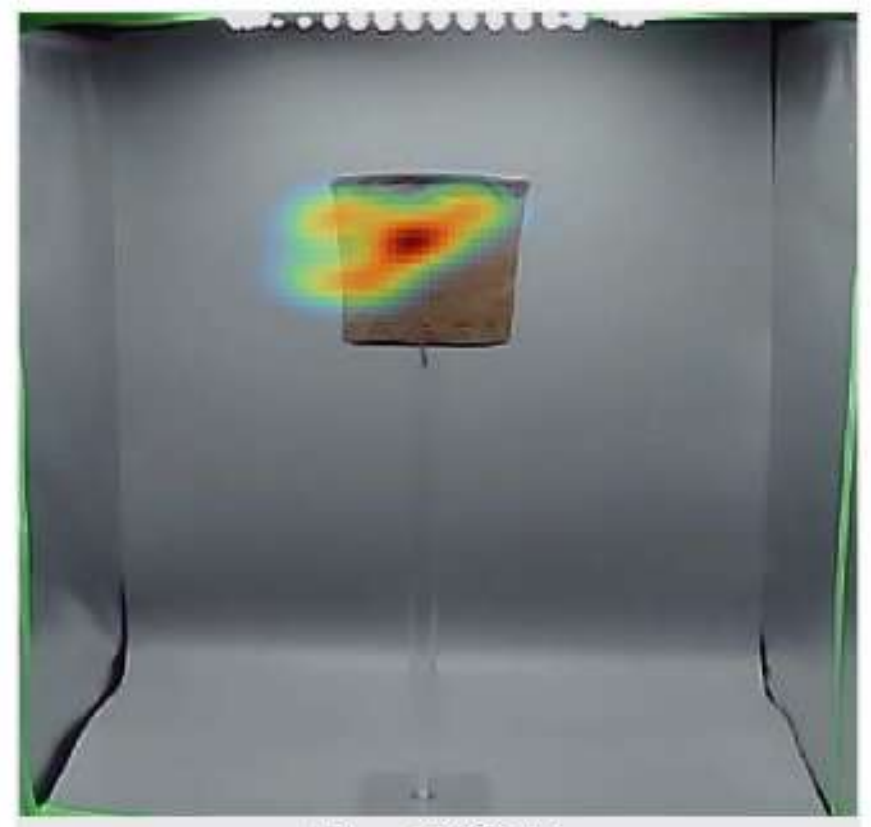
Part0097

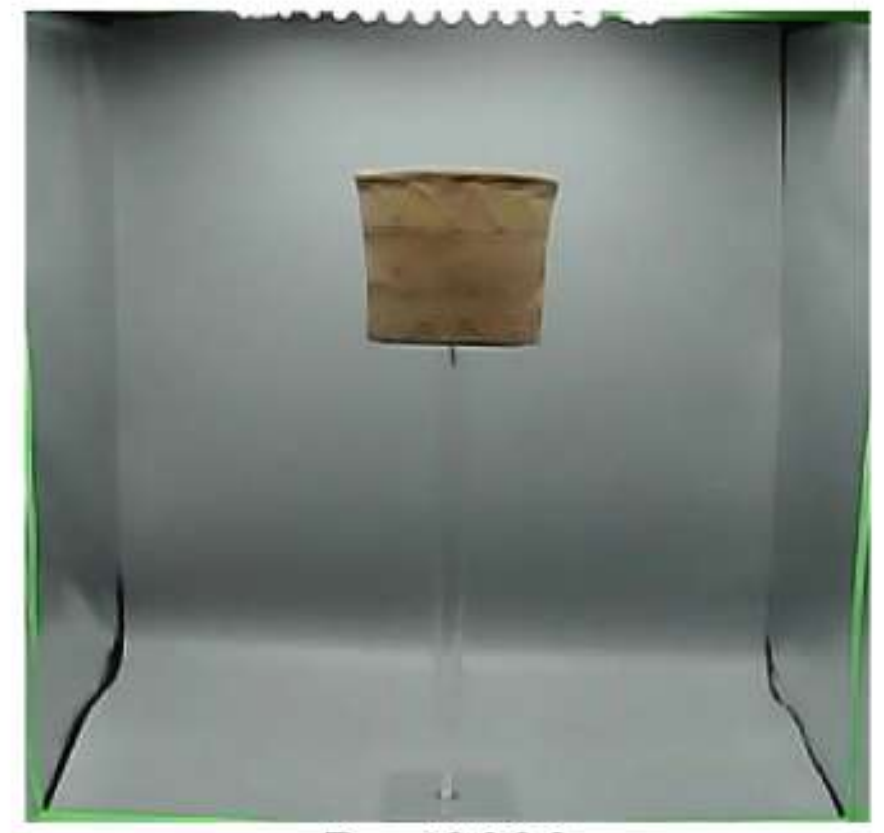
Part0098

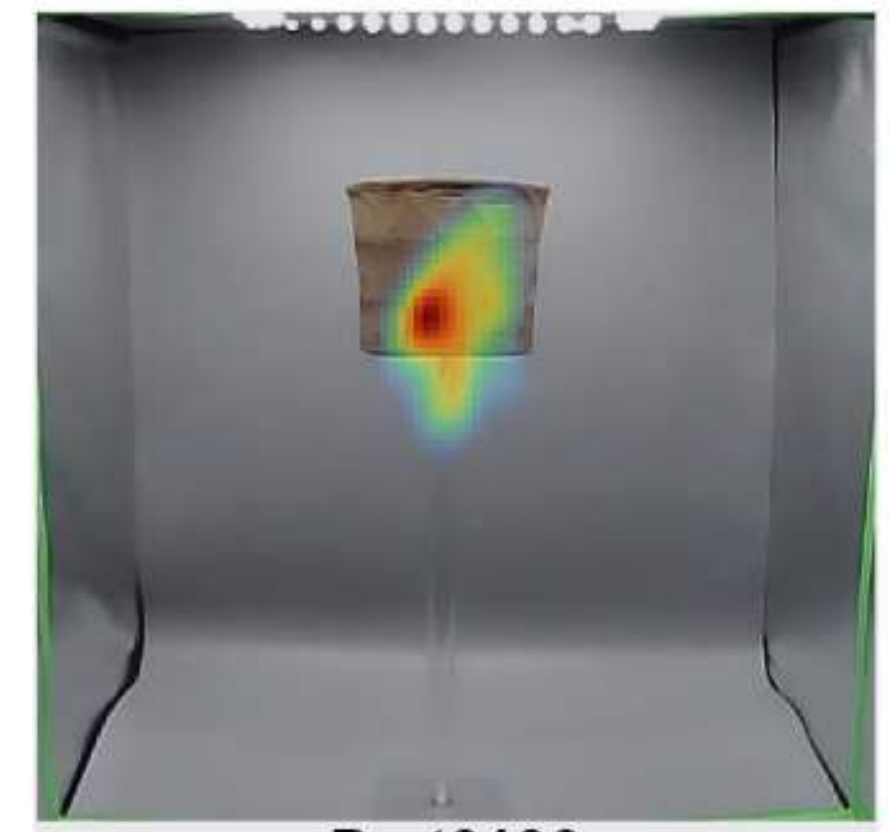
Part0100

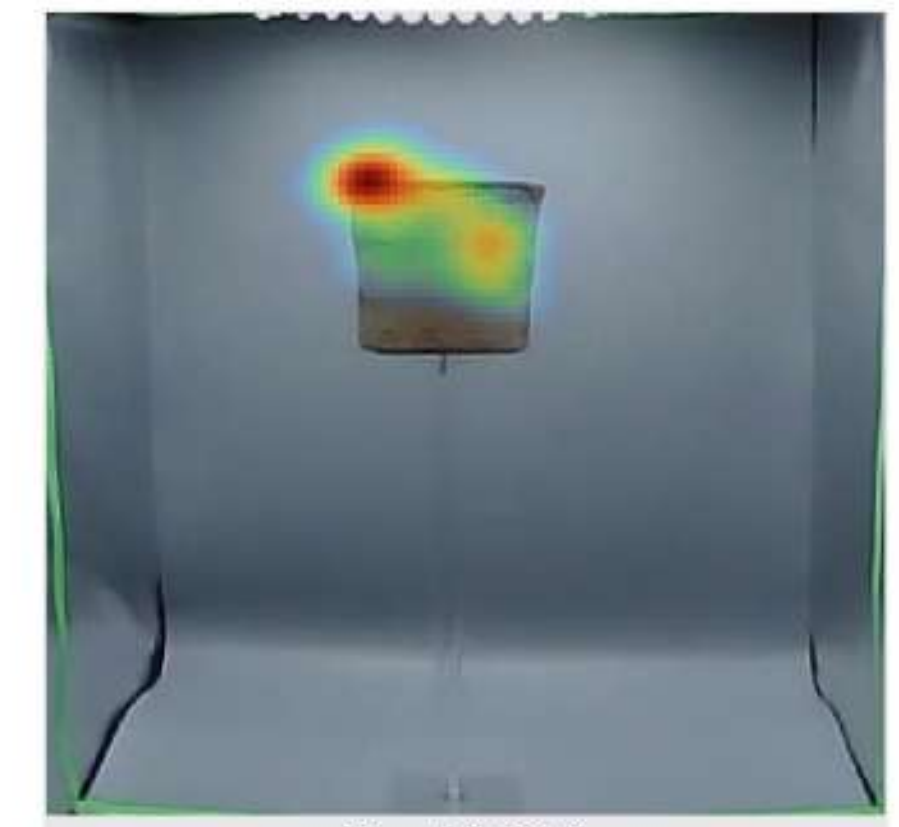
Part0101

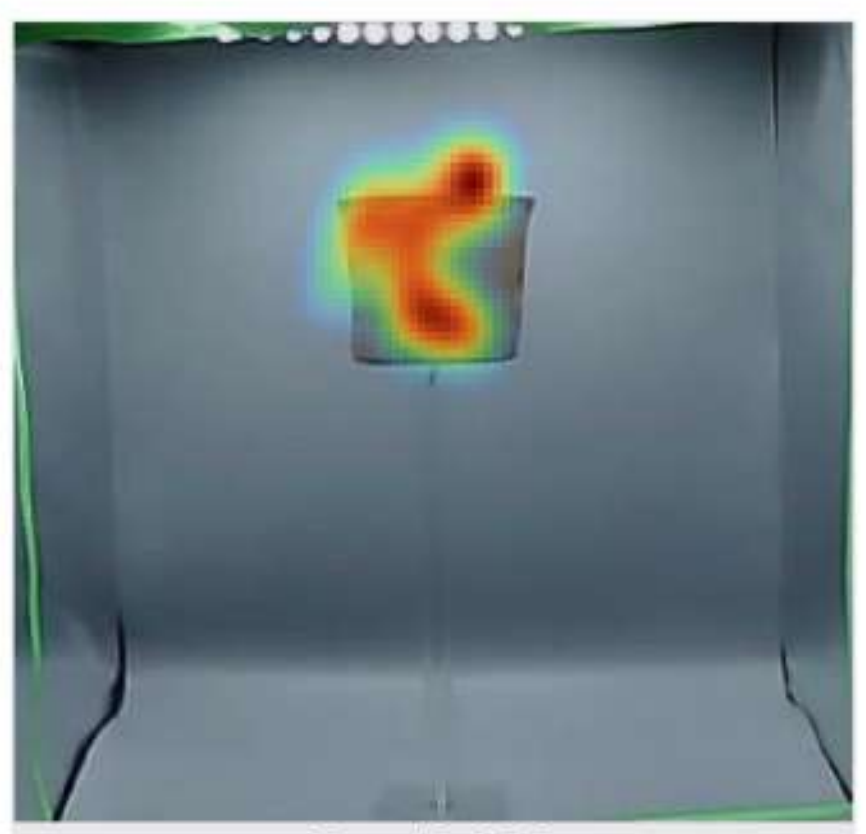
Part0102

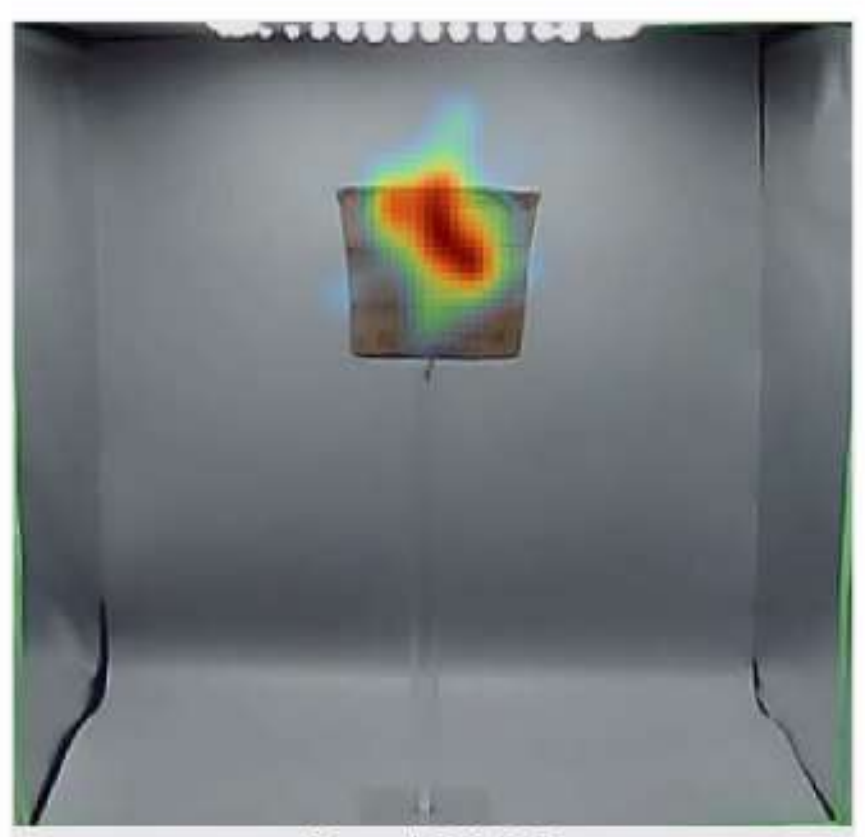
Part0103

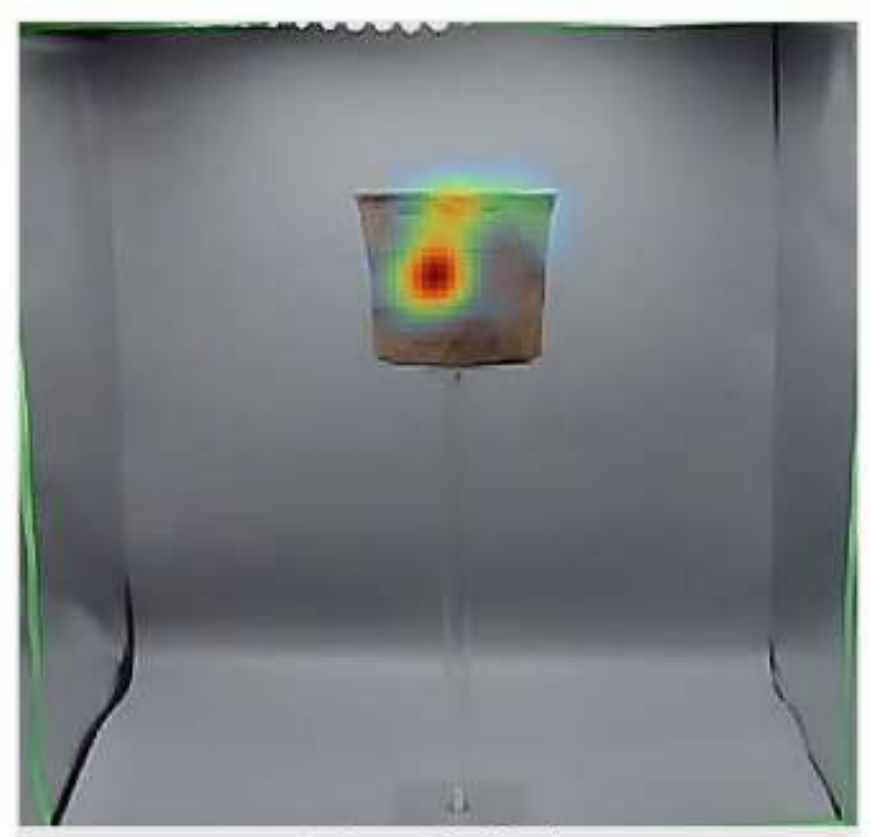
Part0104

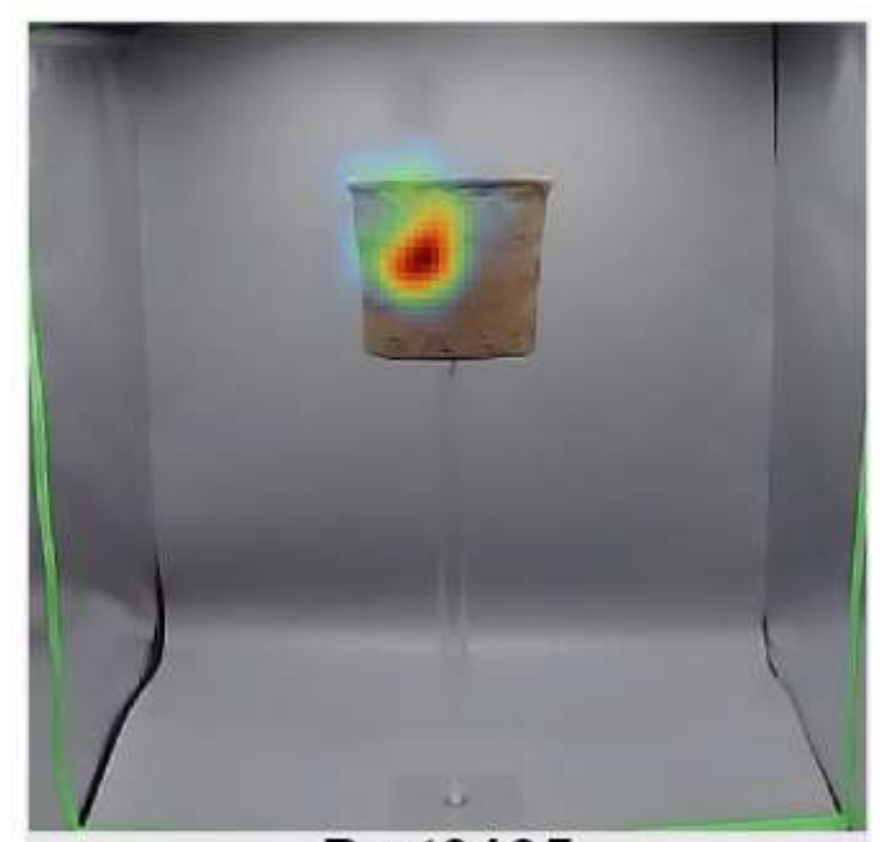
Part0105

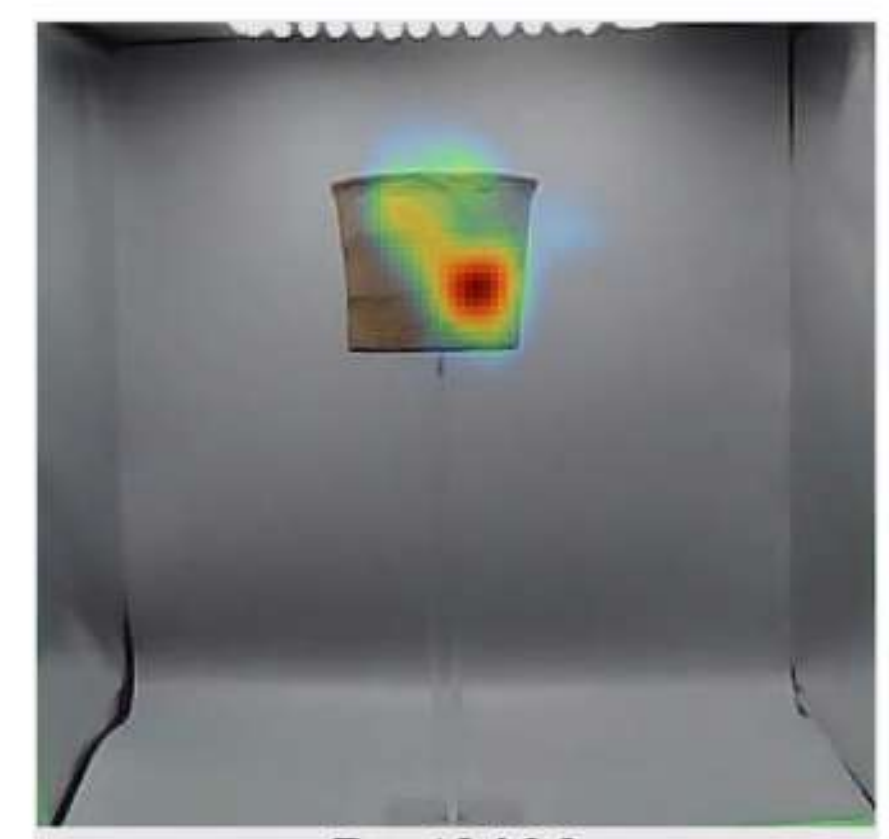
Part0106

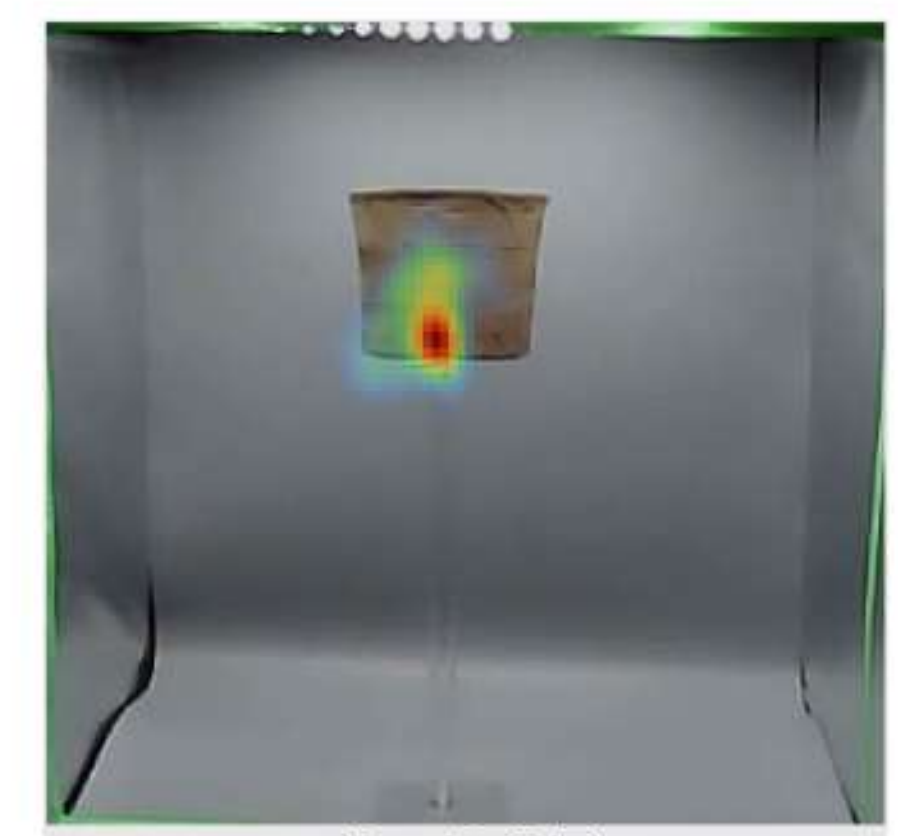
Part0107

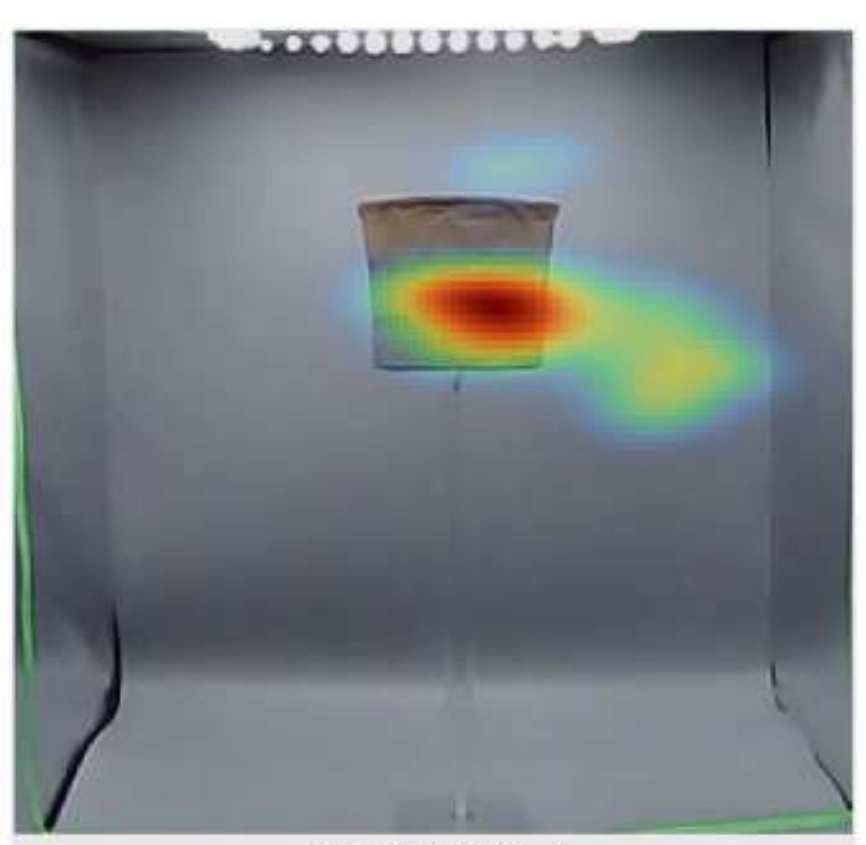
Part0108

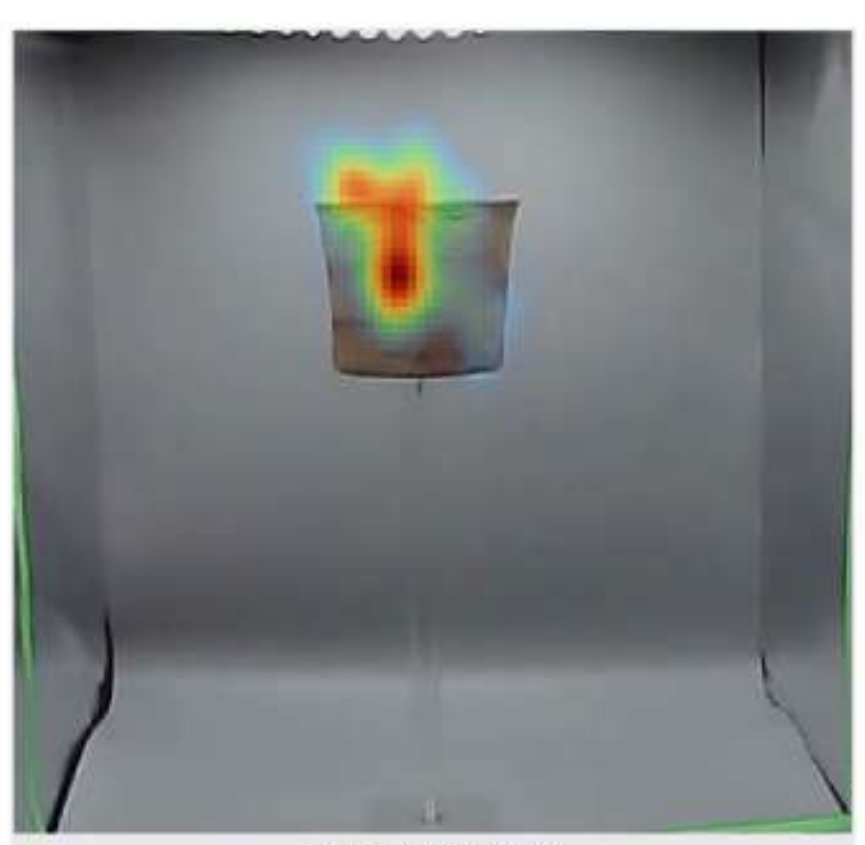
Part0109

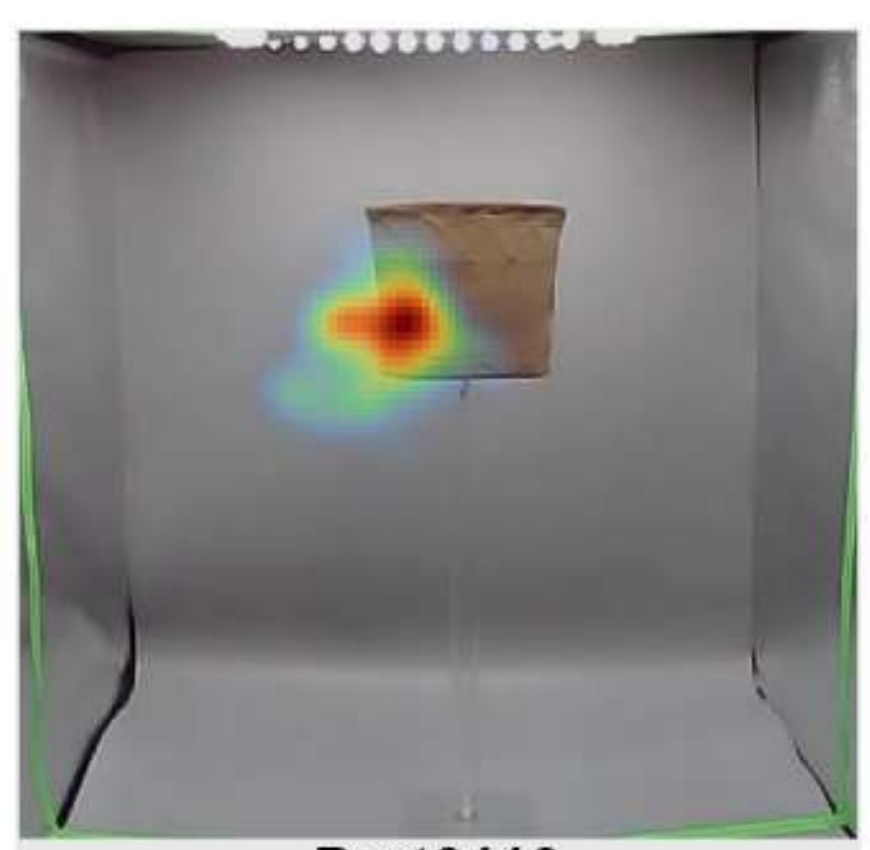
Part0110

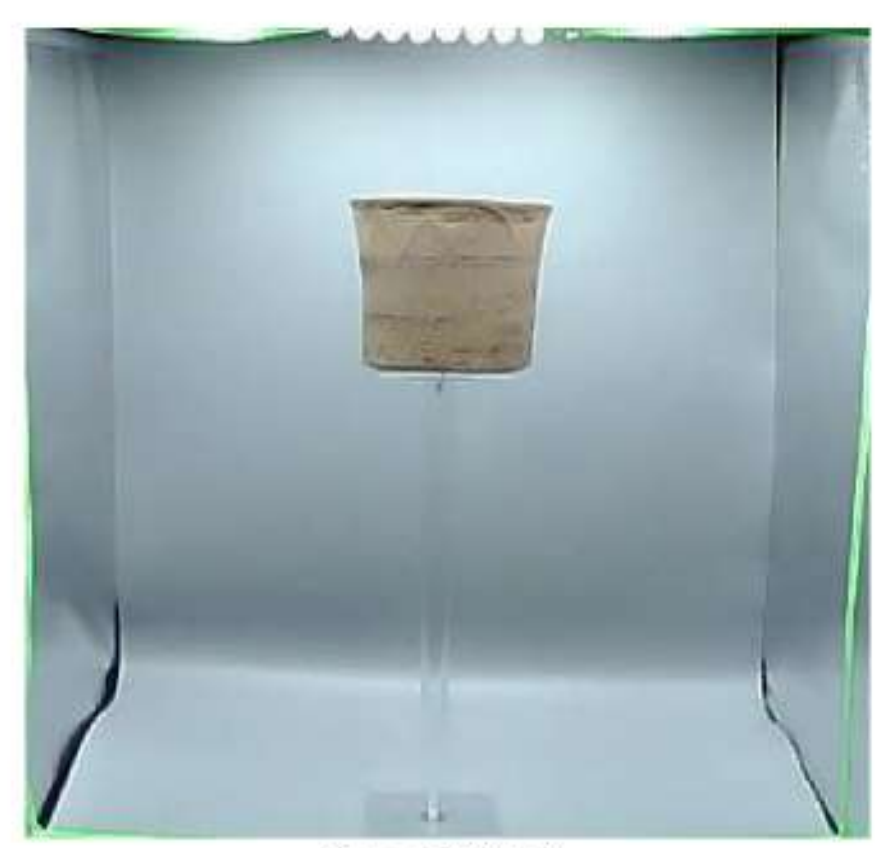
Part0111

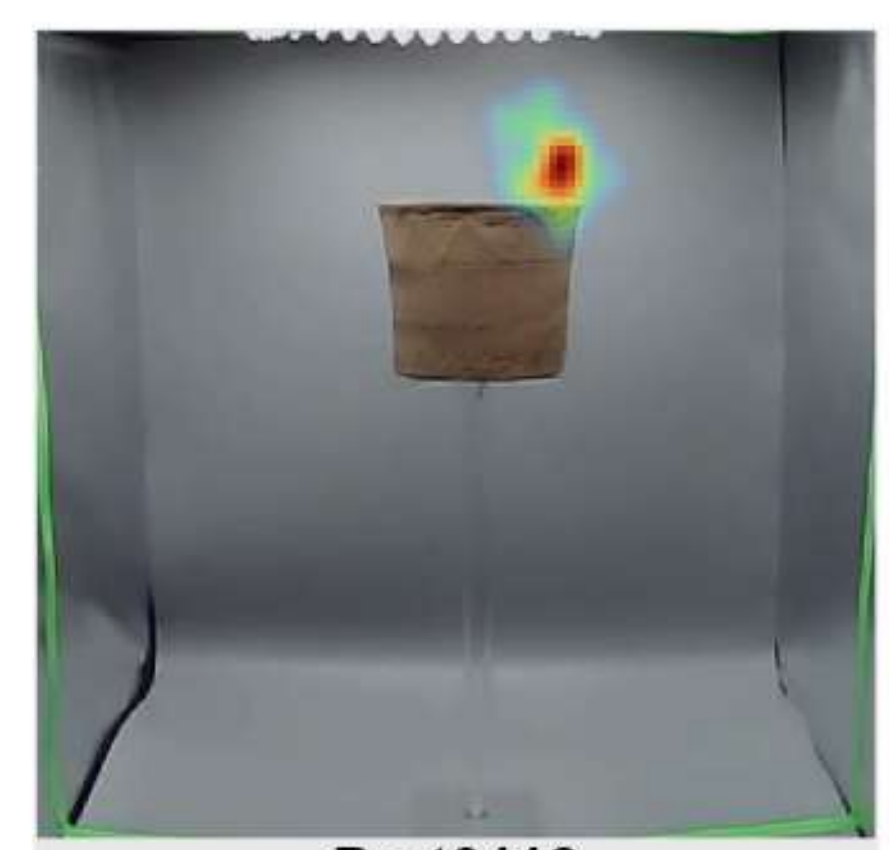
Part0112

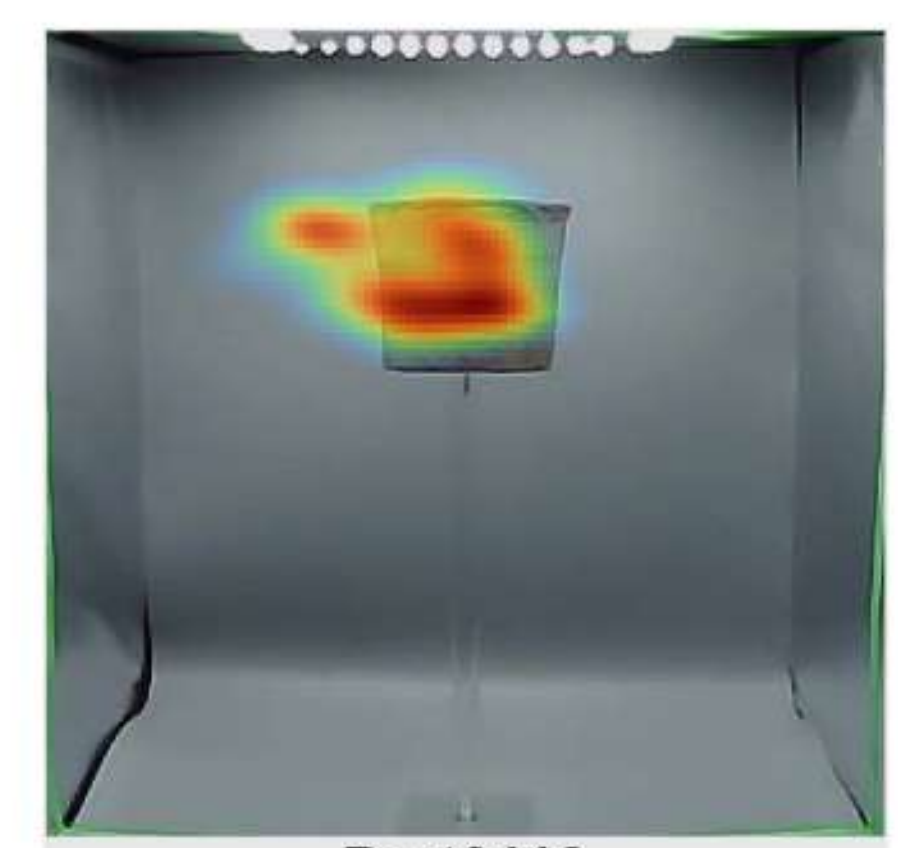
Part0113

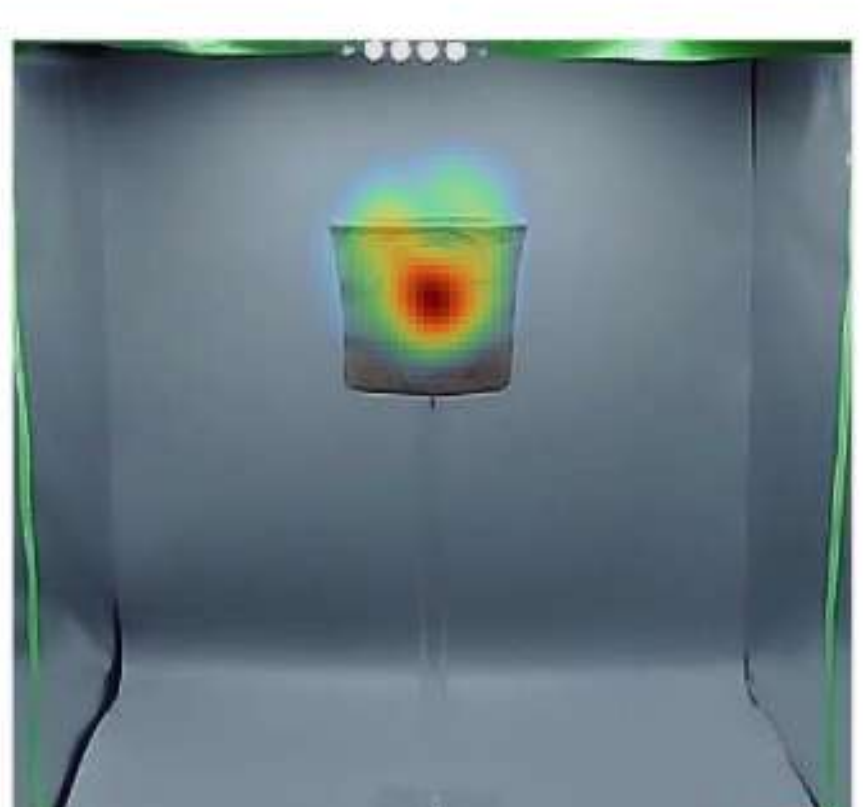
Part0114

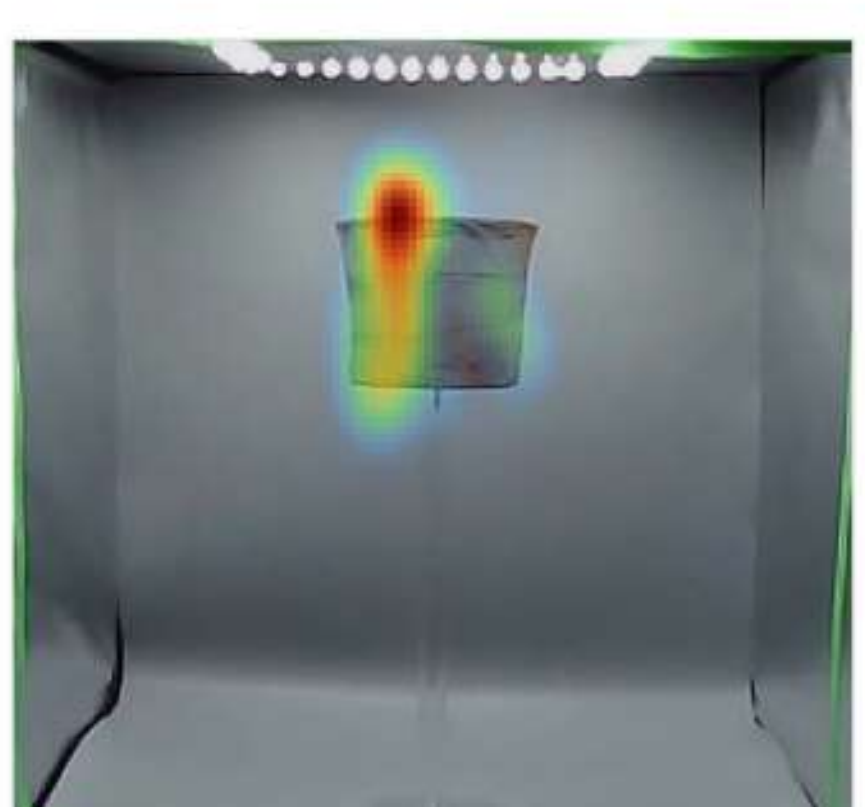
Part0115

Obj0256

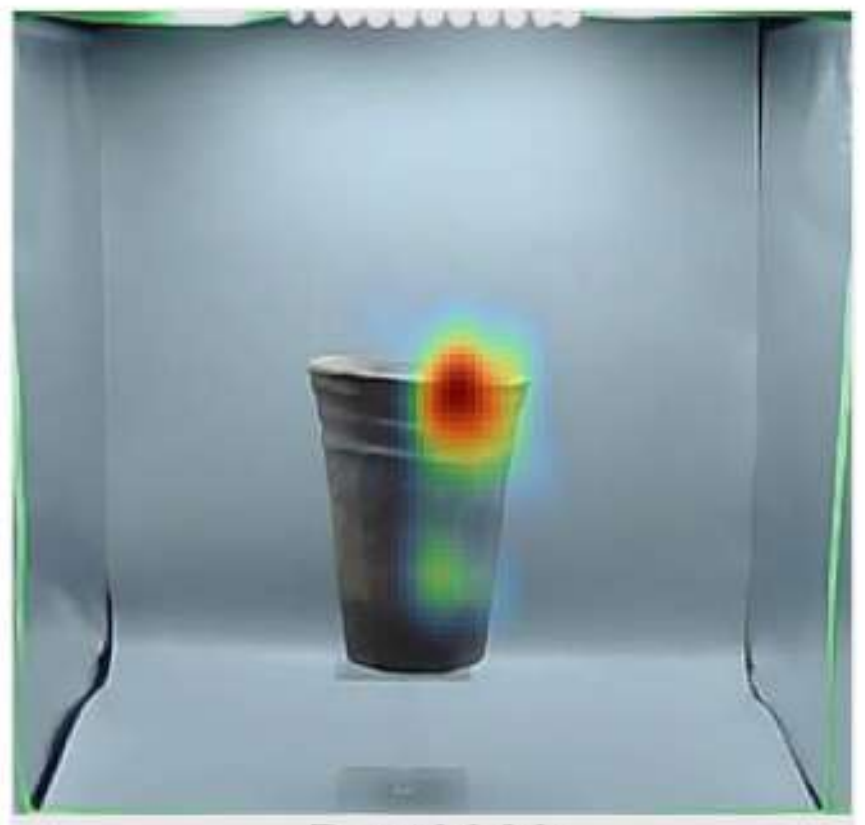
Part0089

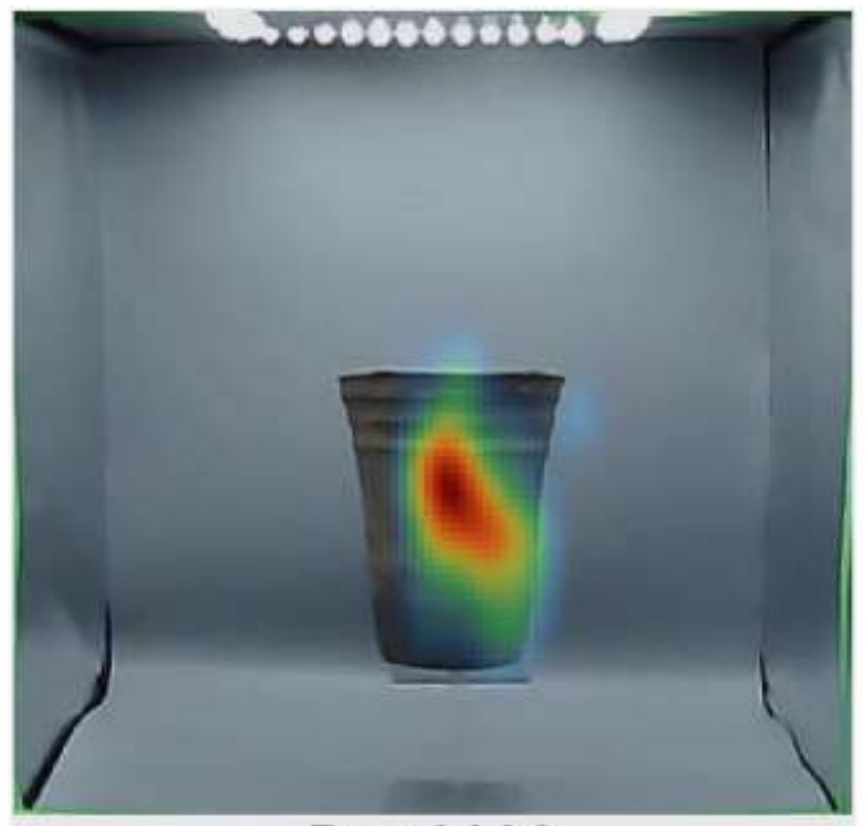
Part0090

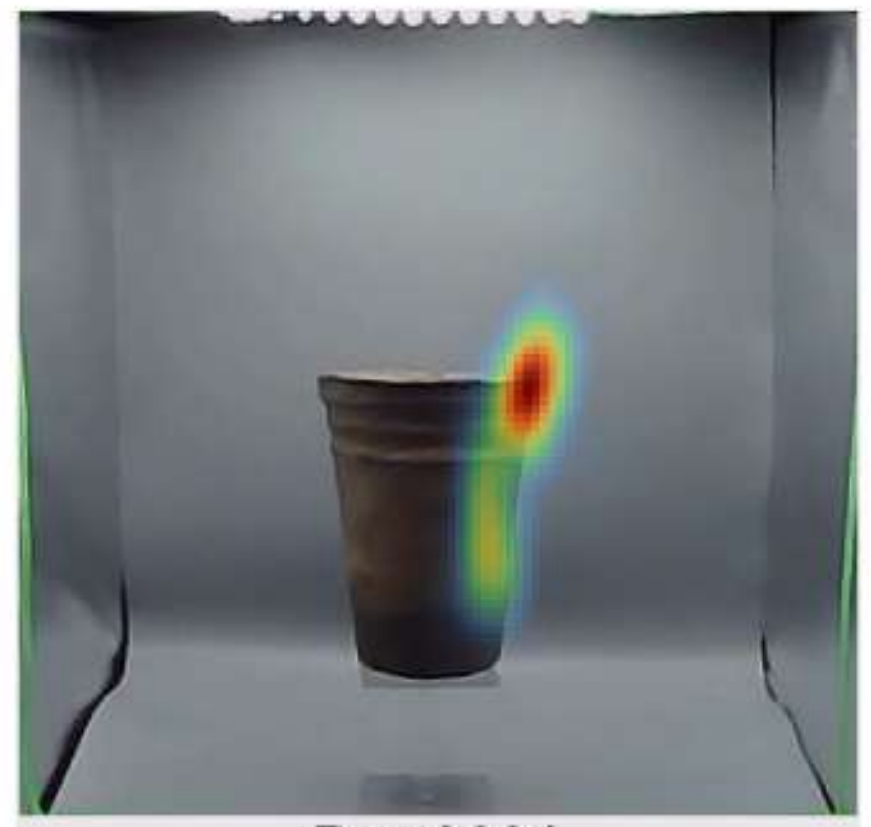
Part0091

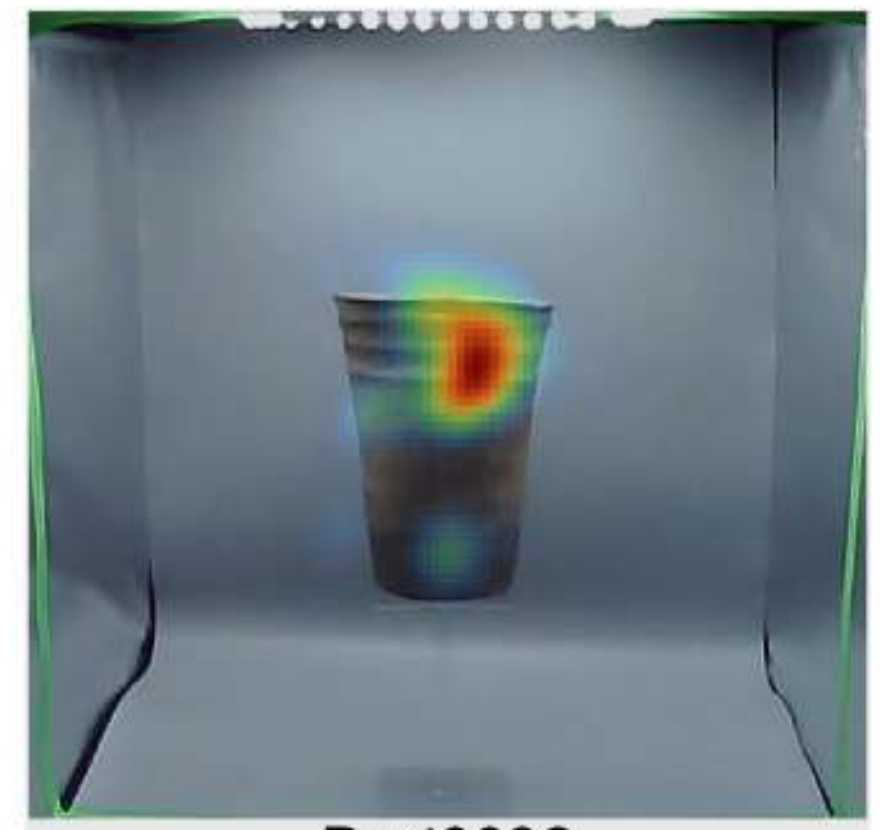
Part0092

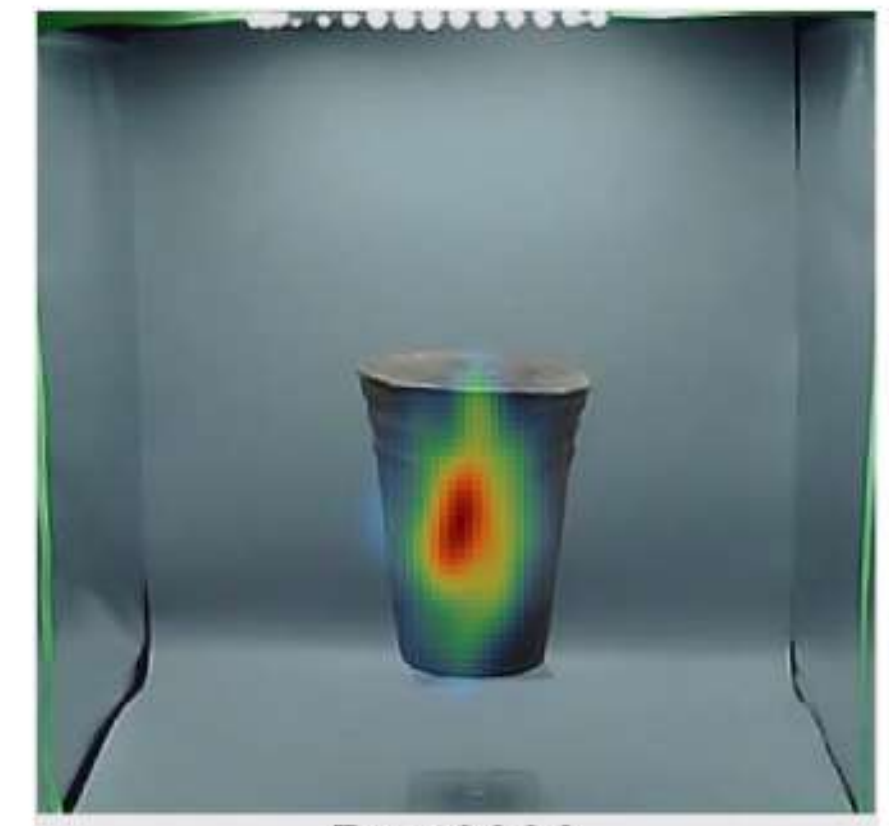
Part0093

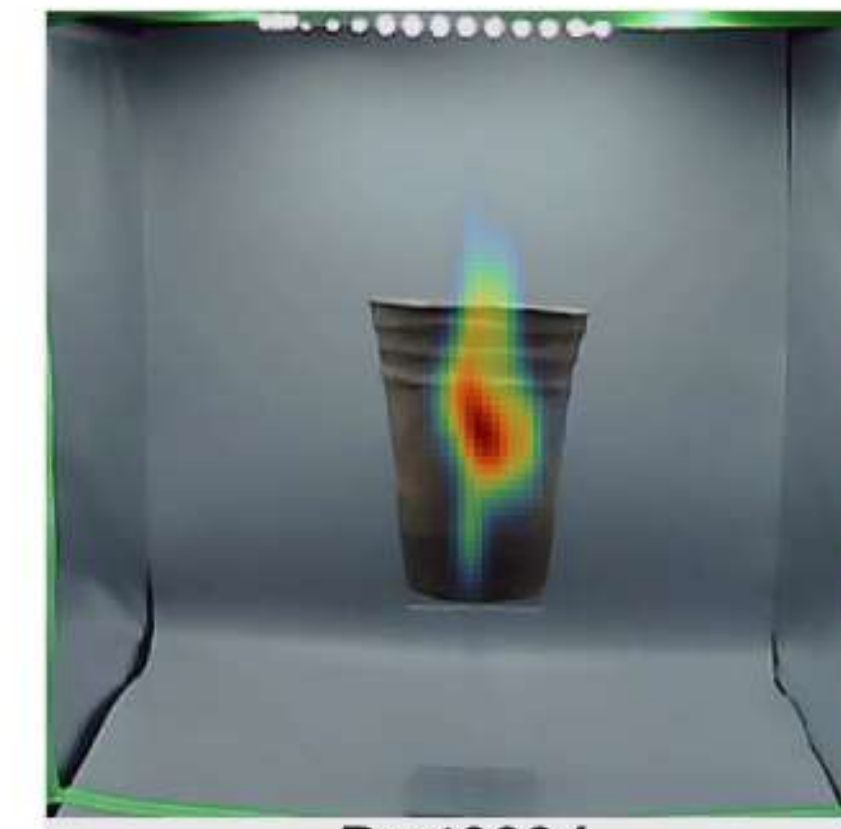
Part0094

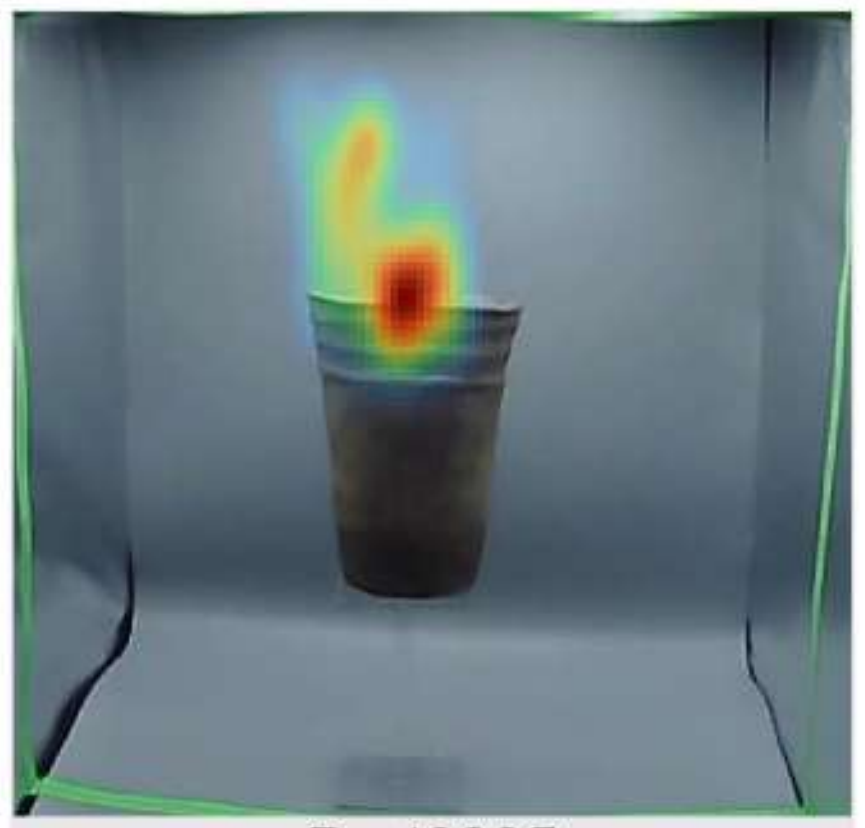
Part0095

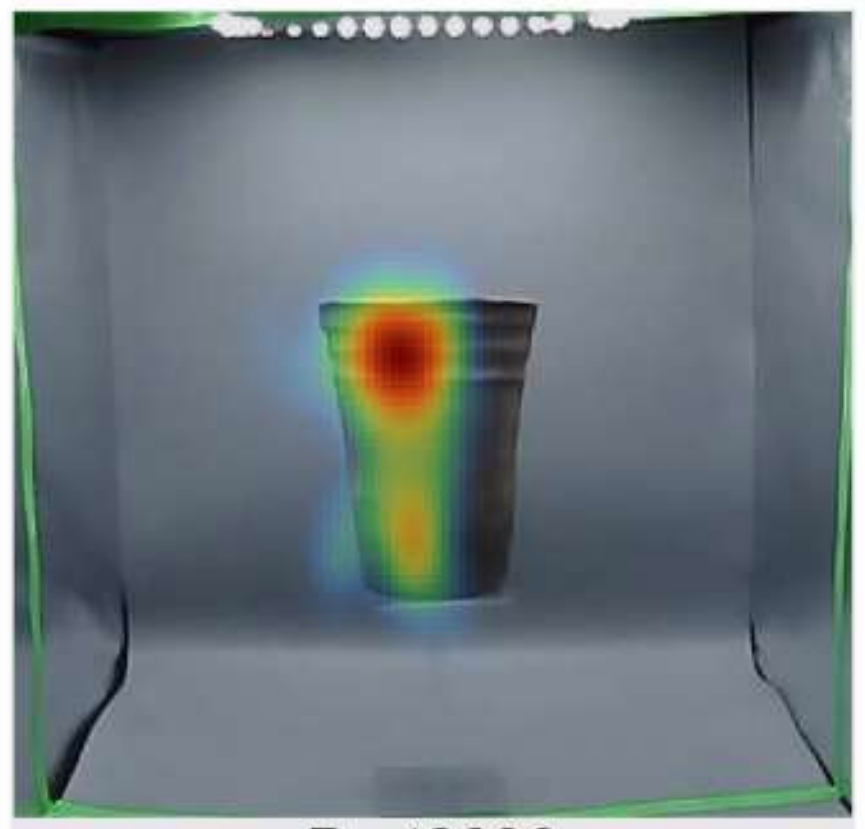
Part0096

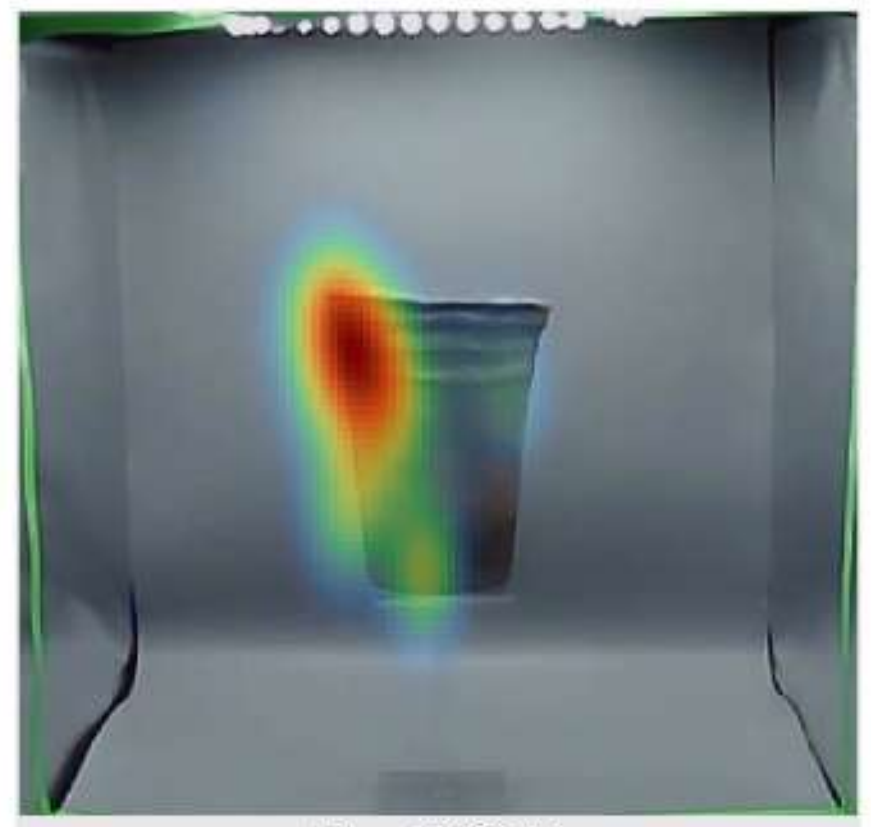
Part0097

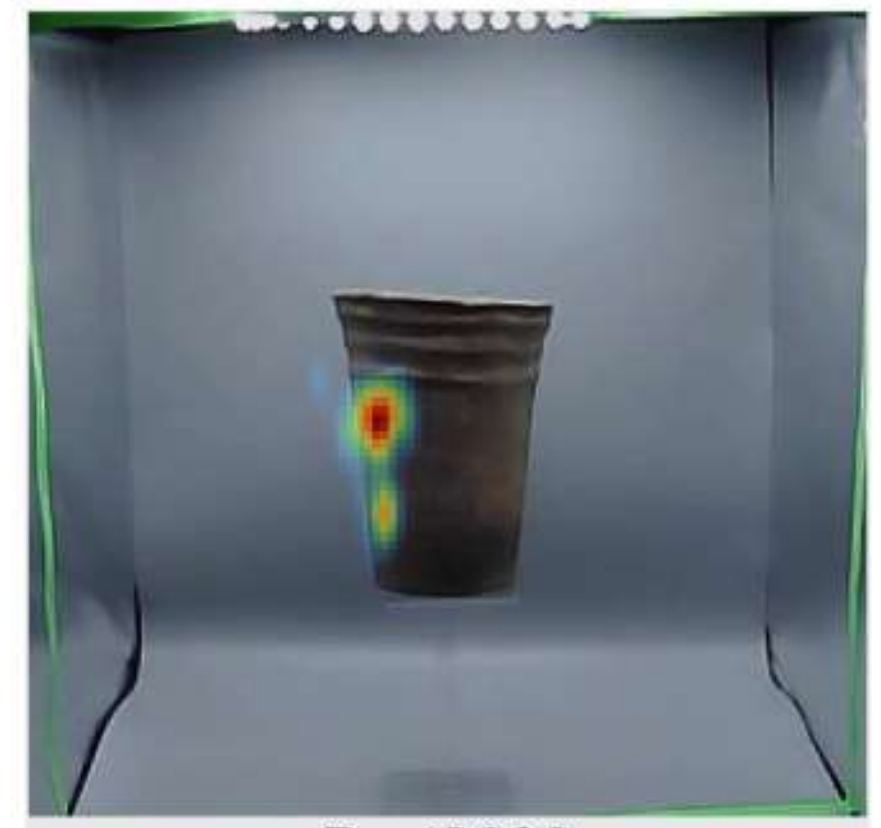
Part0098

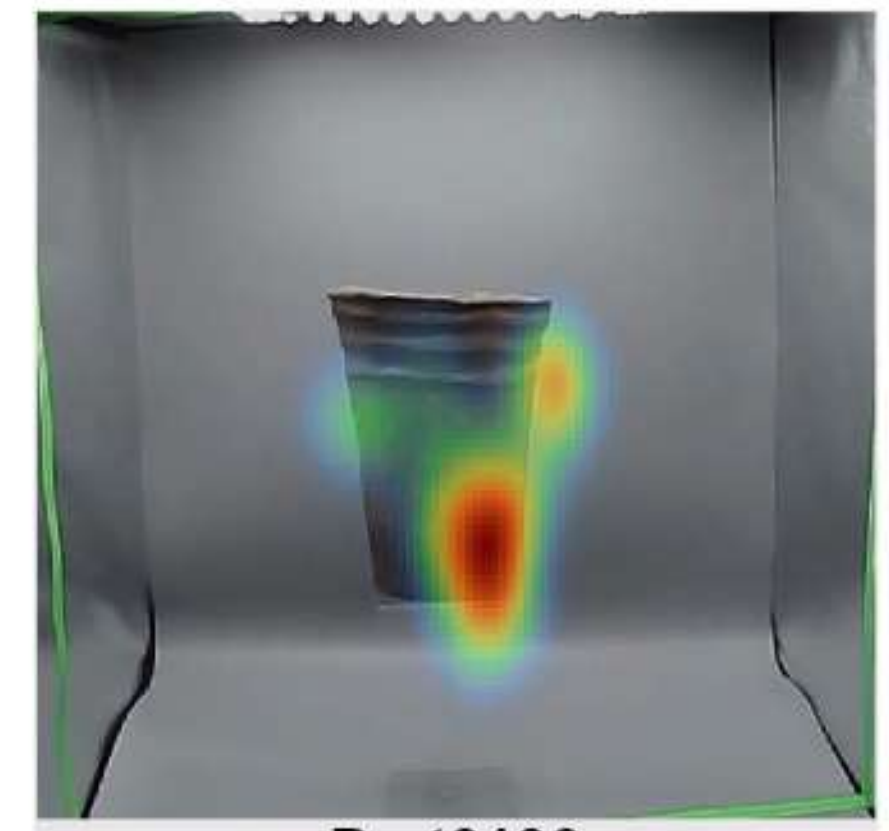
Part0100

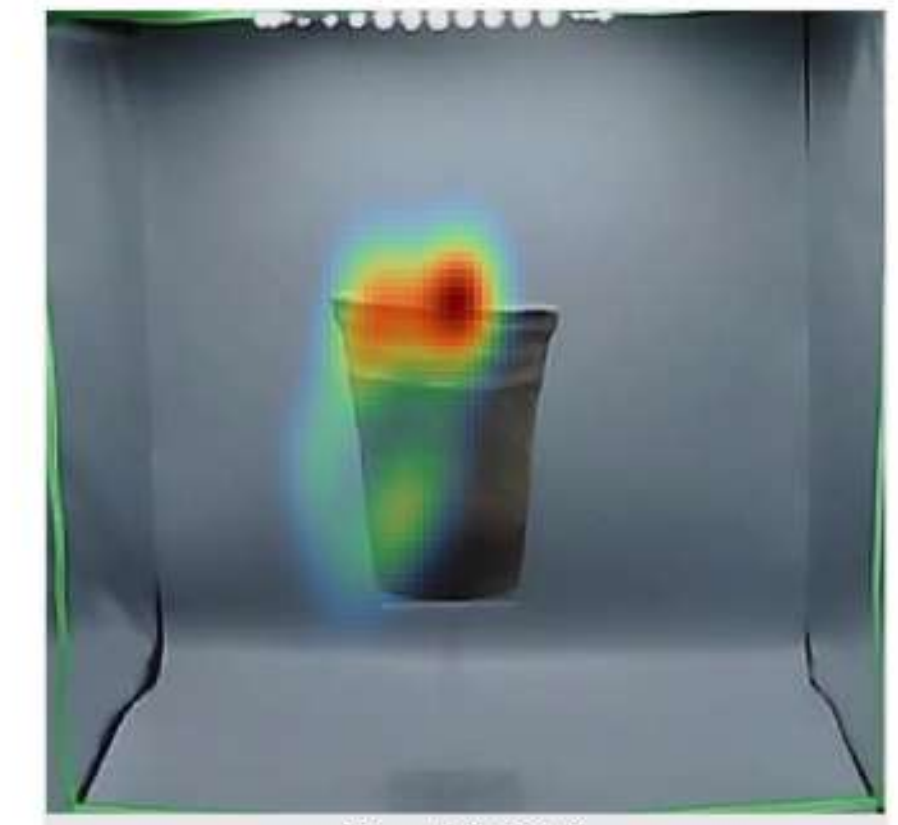
Part0101

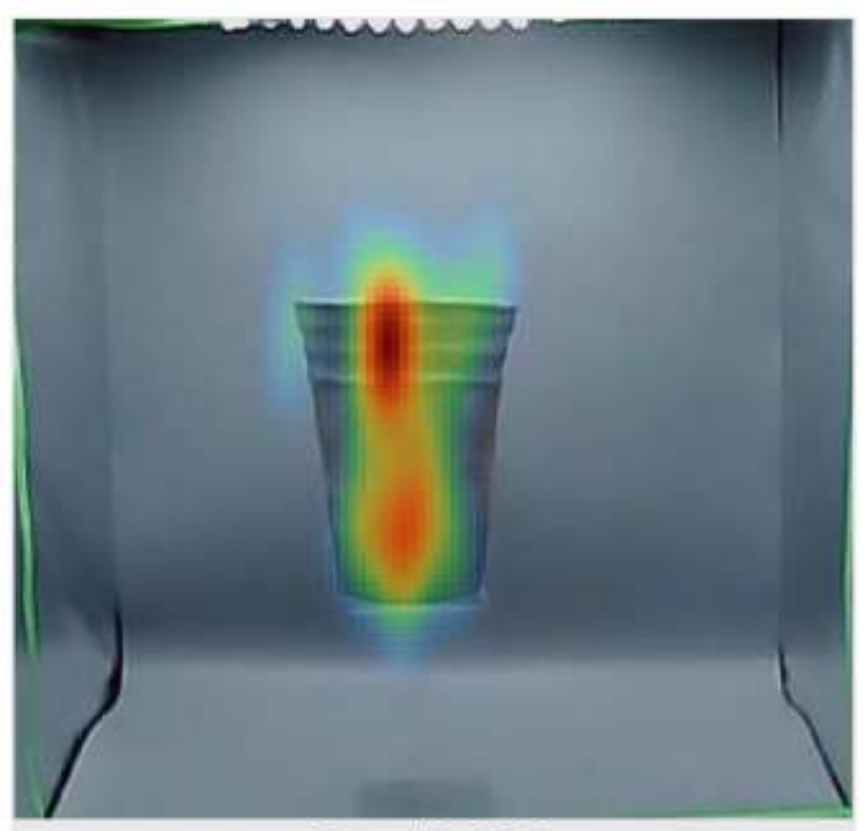
Part0102

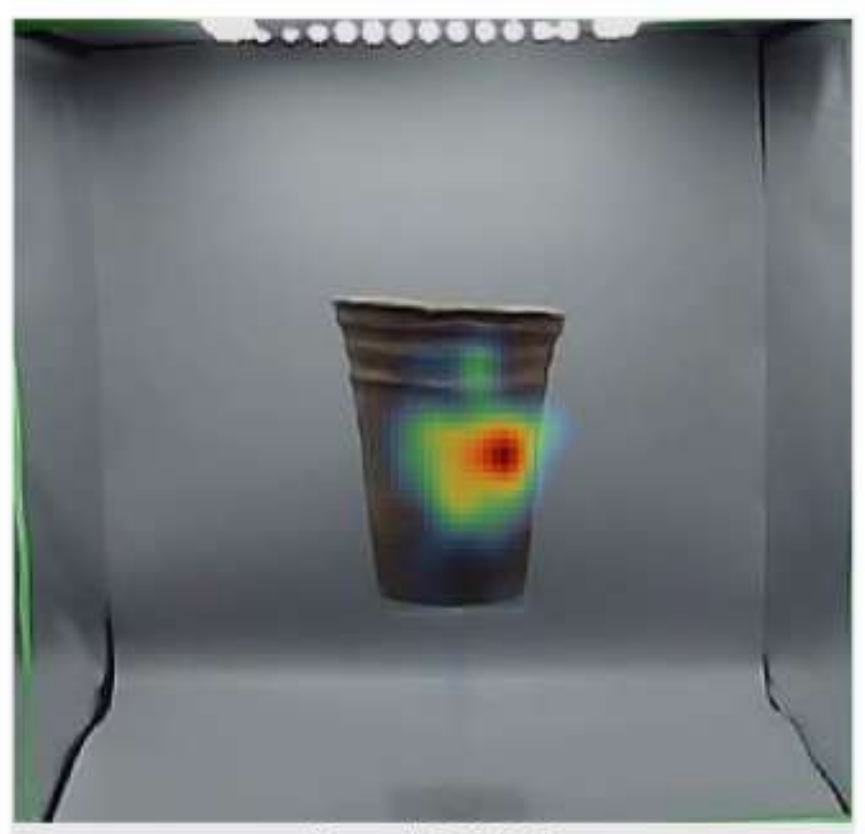
Part0103

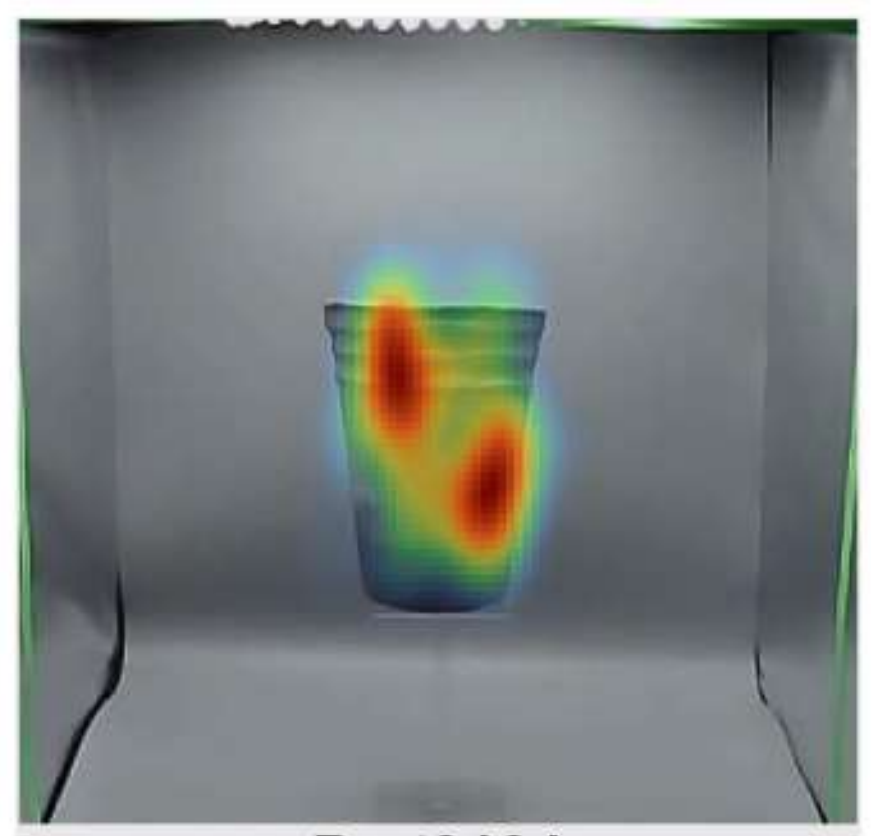
Part0104

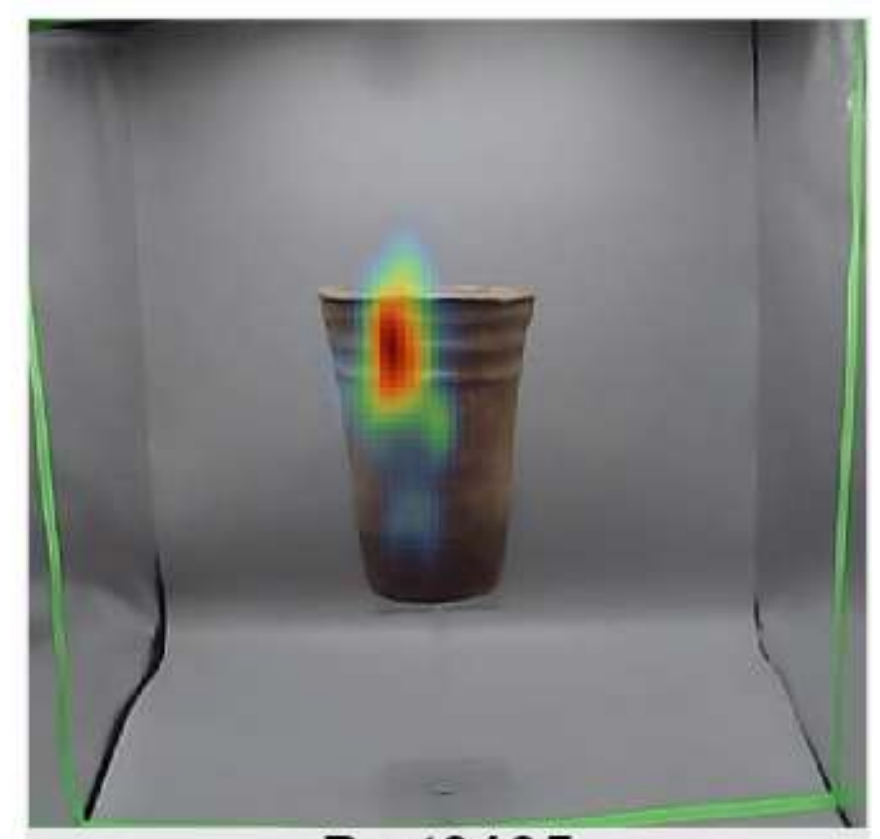
Part0105

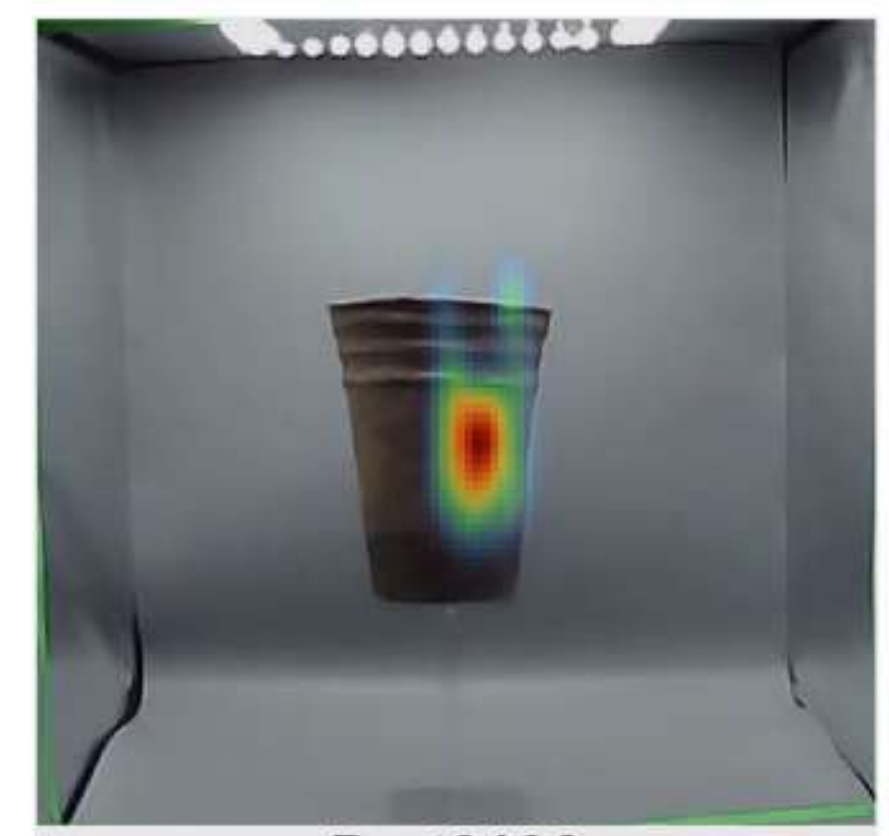
Part0106

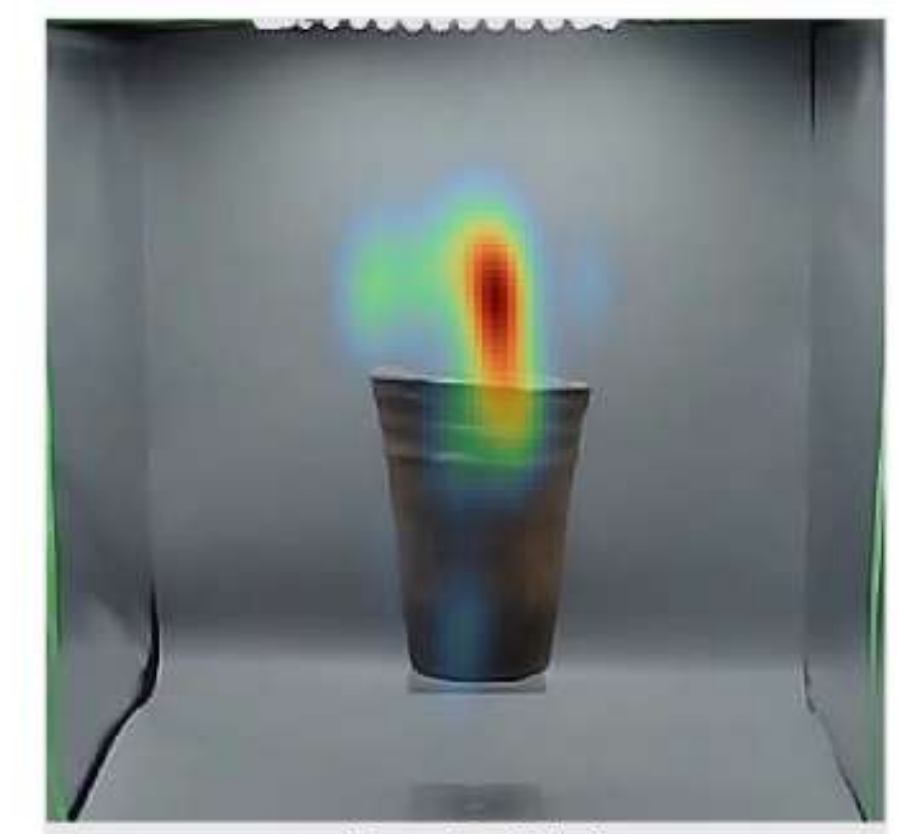
Part0107

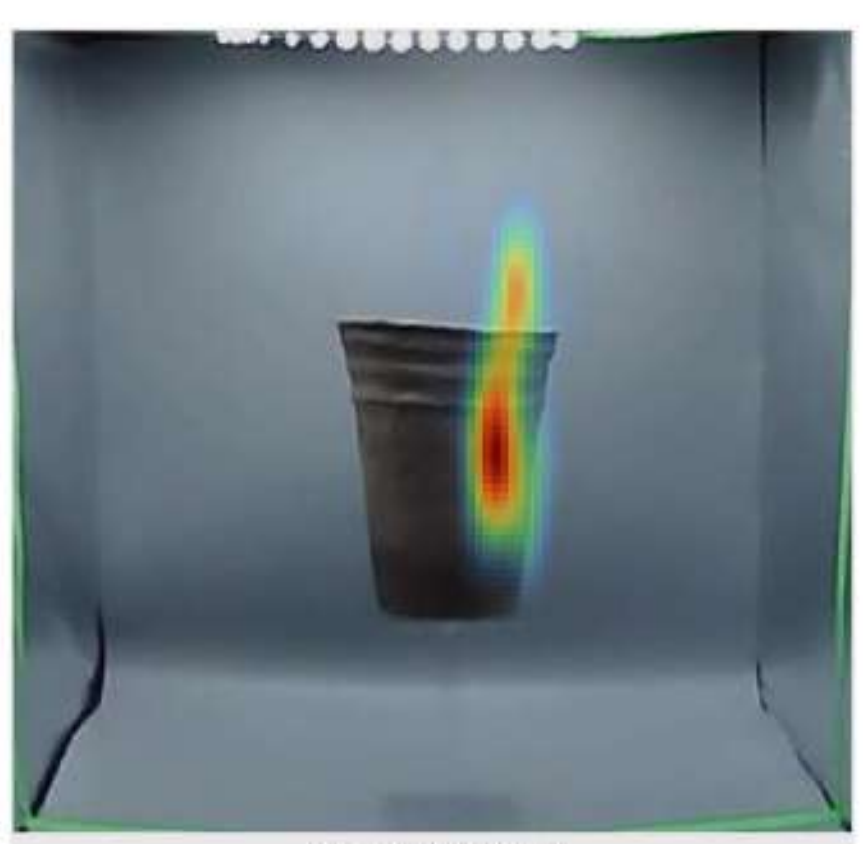
Part0108

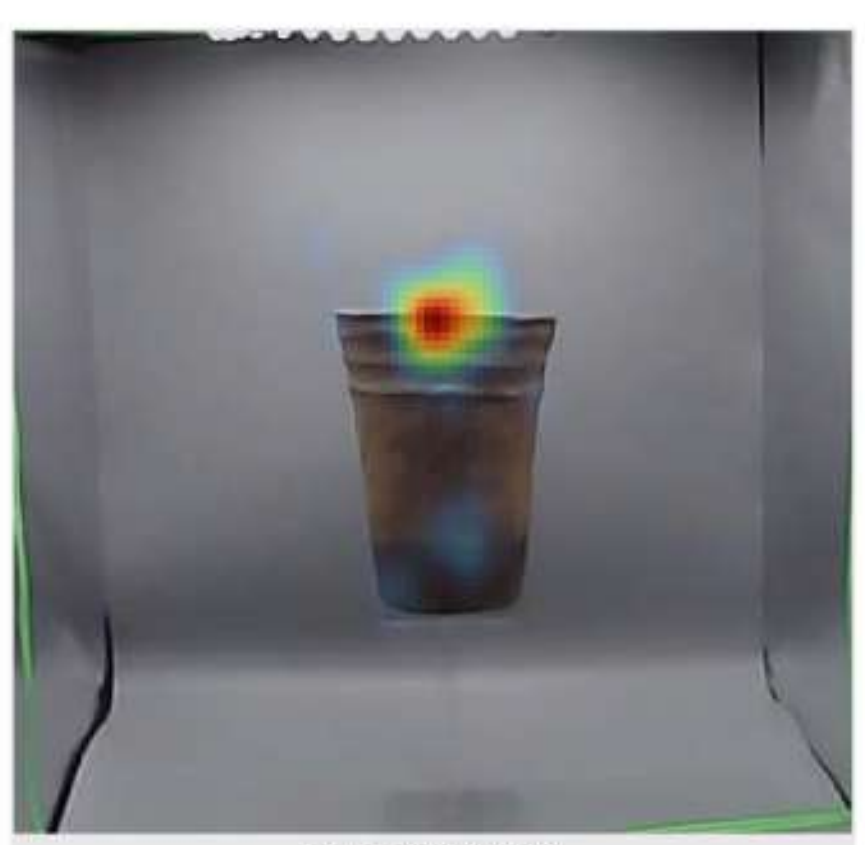
Part0109

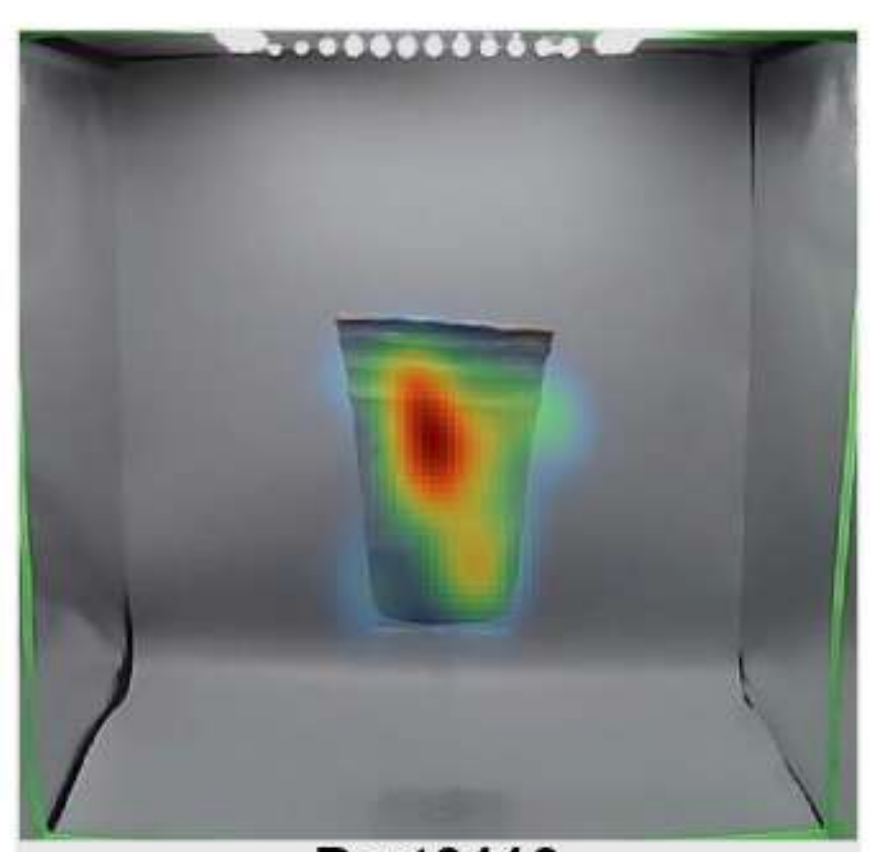
Part0110

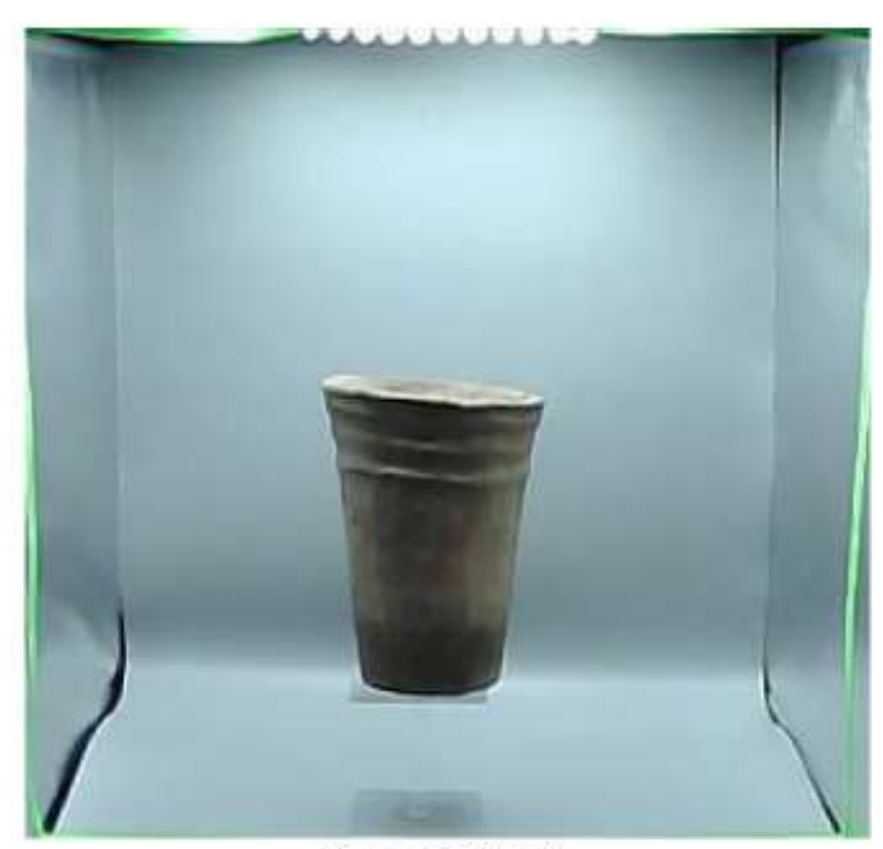
Part0111

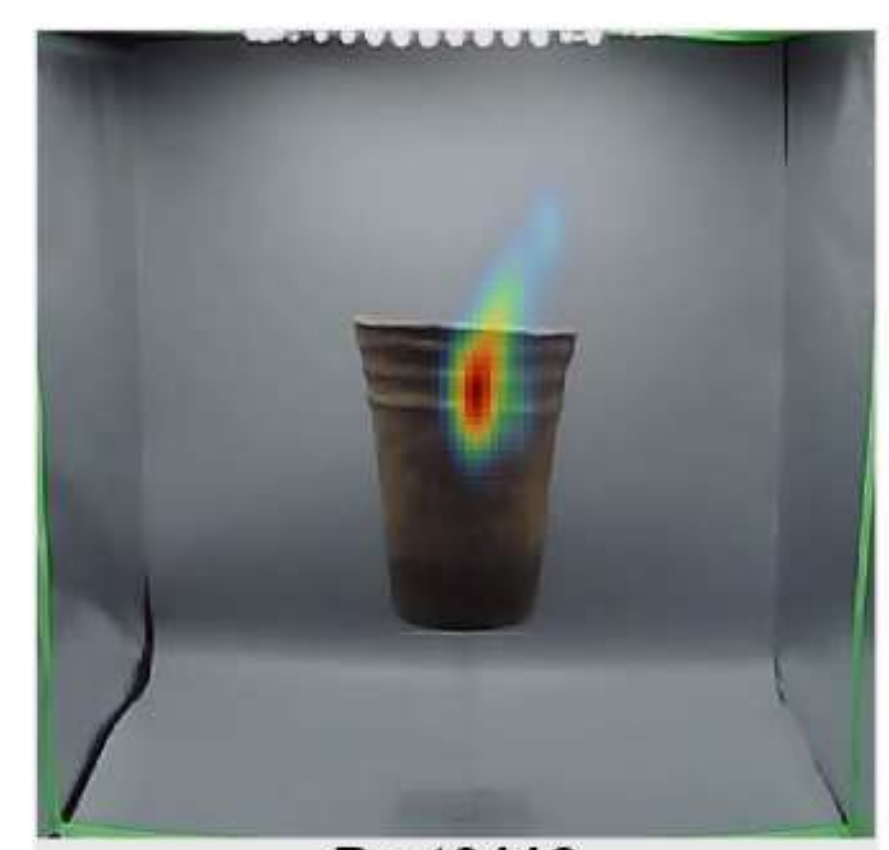
Part0112

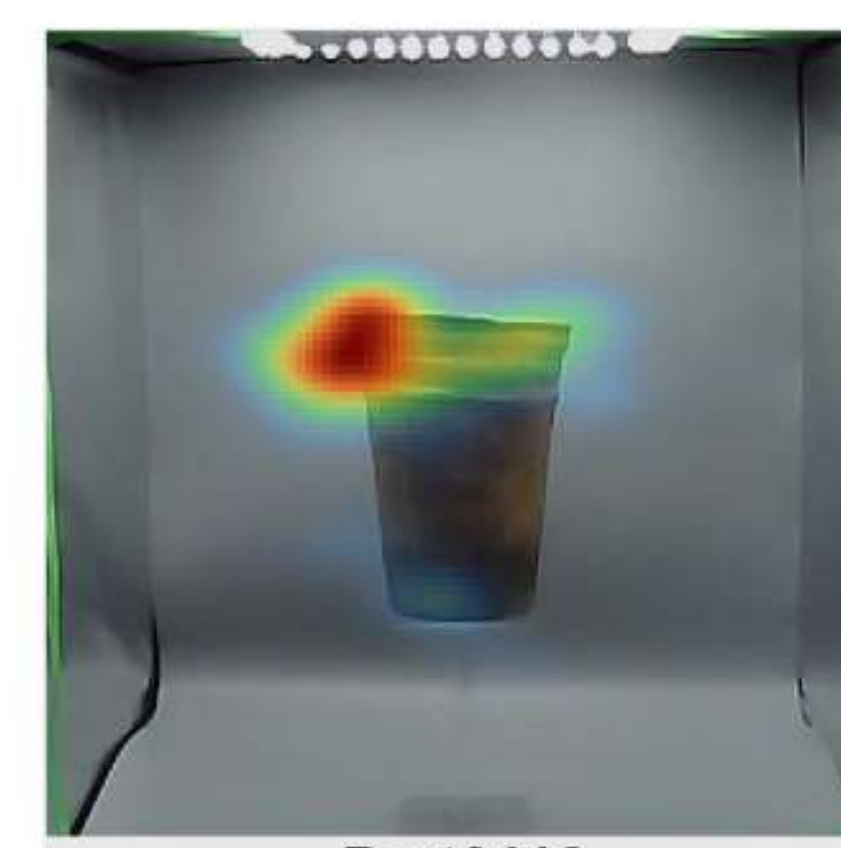
Part0113

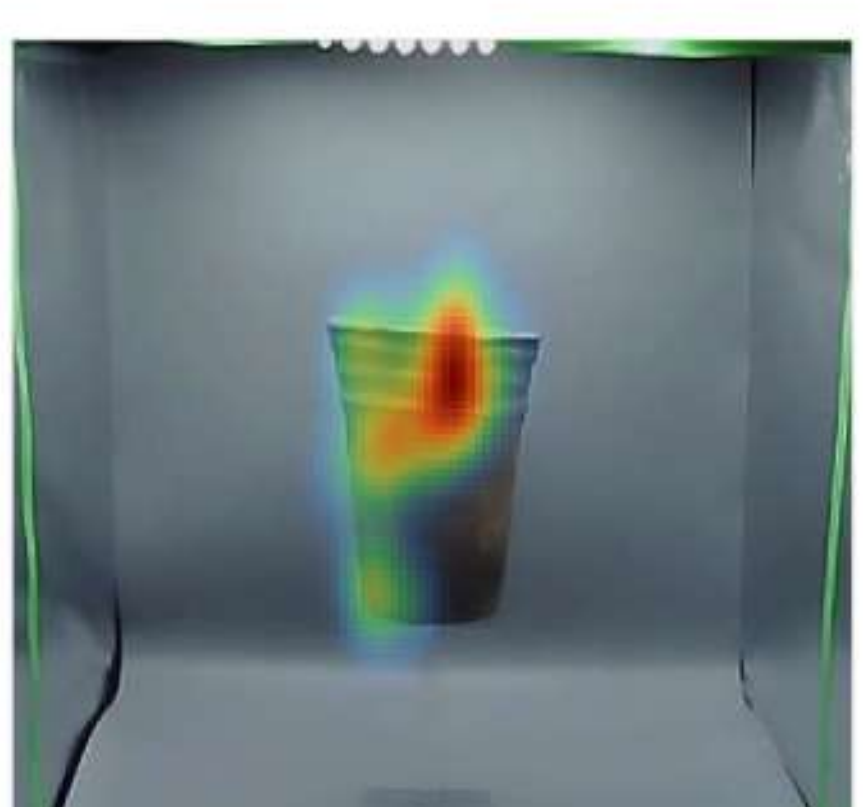
Part0114

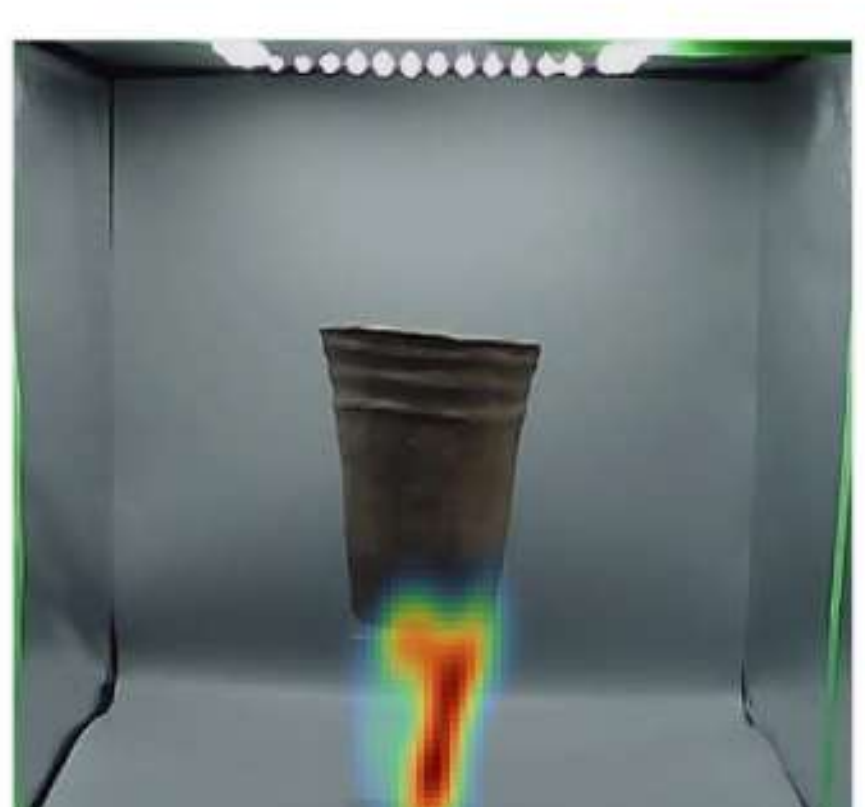
Part0115

Obj0258

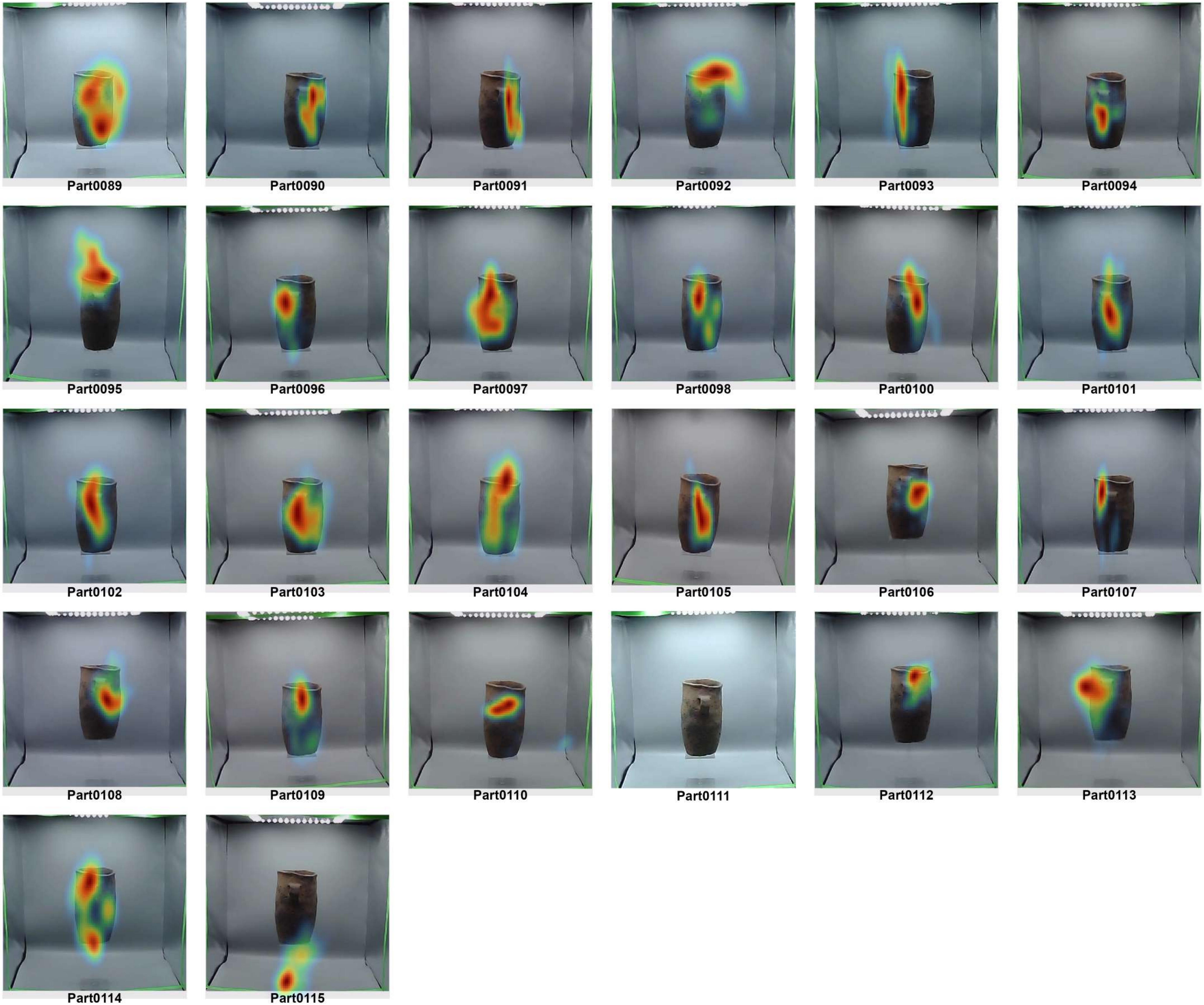
Part0089
Part0090
Part0091
Part0092
Part0093
Part0094
Part0095
Part0096
Part0097
Part0098
Part0100
Part0101
Part0102
Part0103
Part0104
Part0105
Part0106
Part0107
Part0108
Part0109
Part0110
Part0111
Part0112
Part0113
Part0114
Part0115

Obj0260

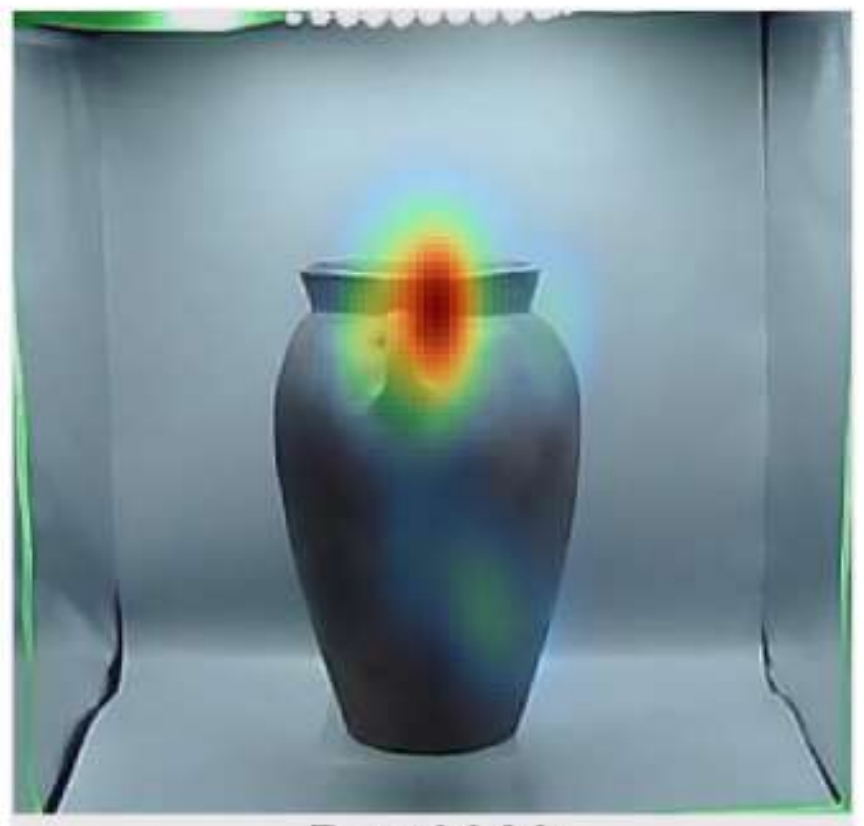
Part0089

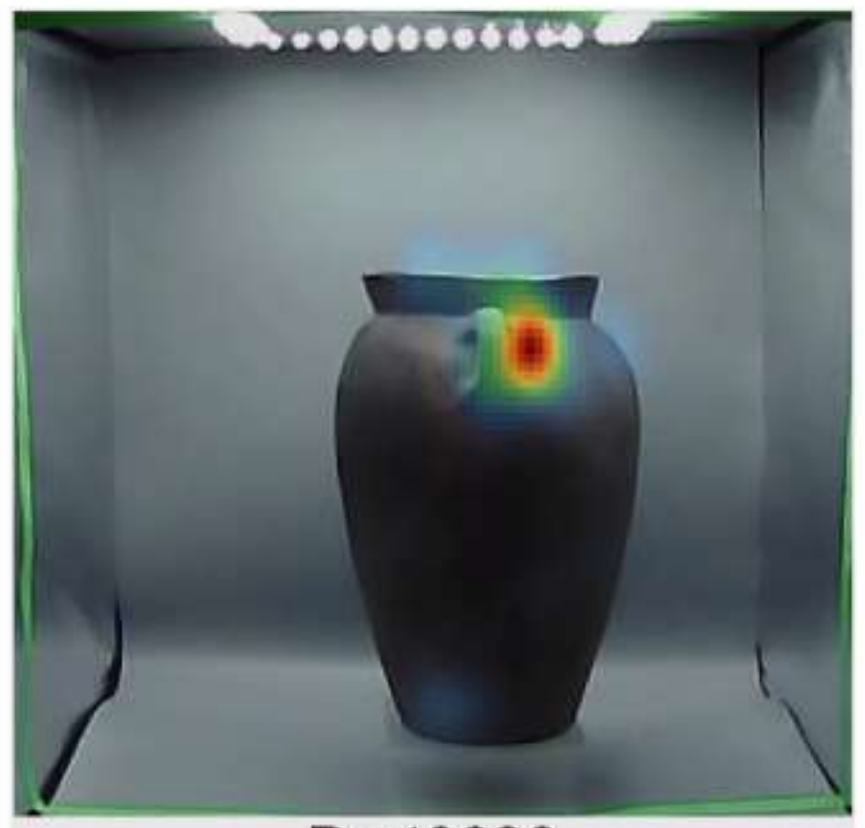
Part0090

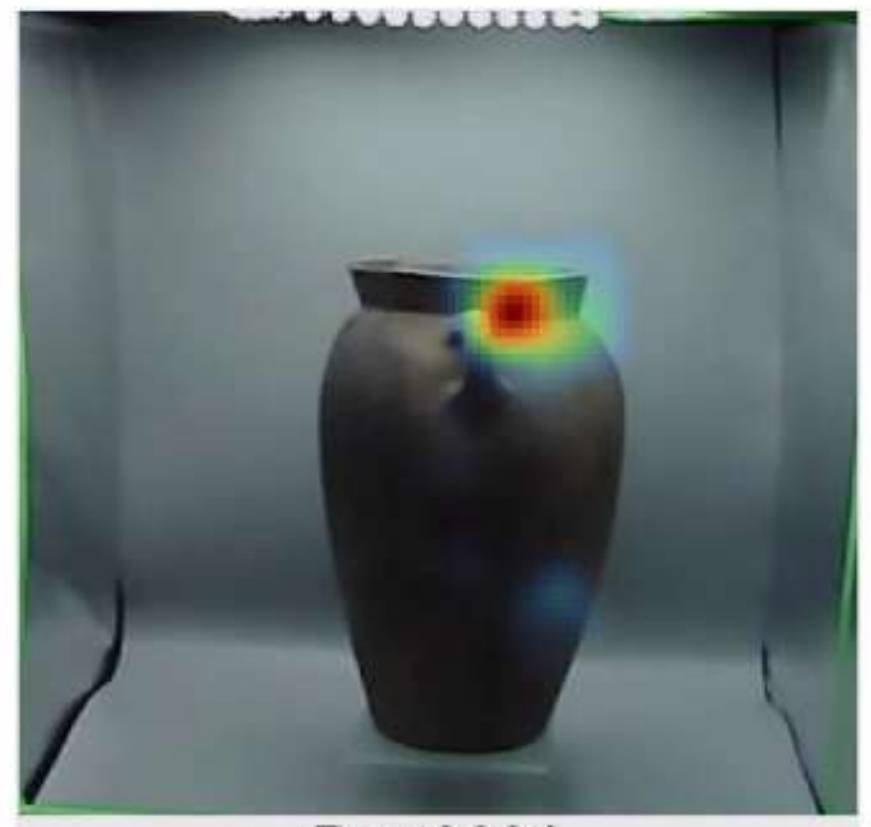
Part0091

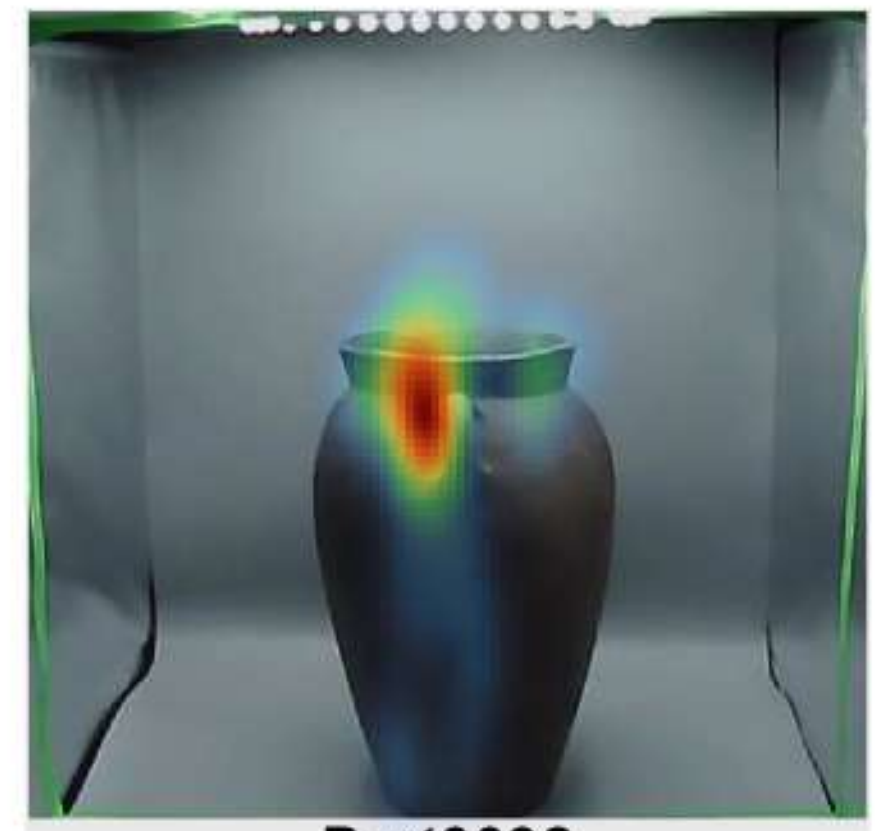
Part0092

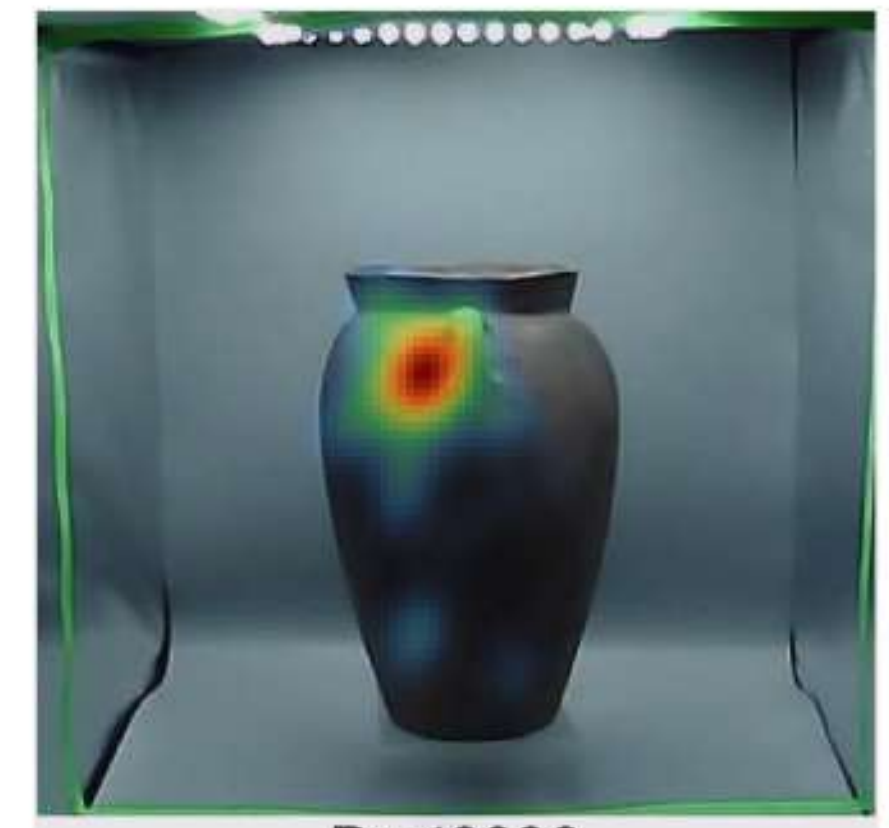
Part0093

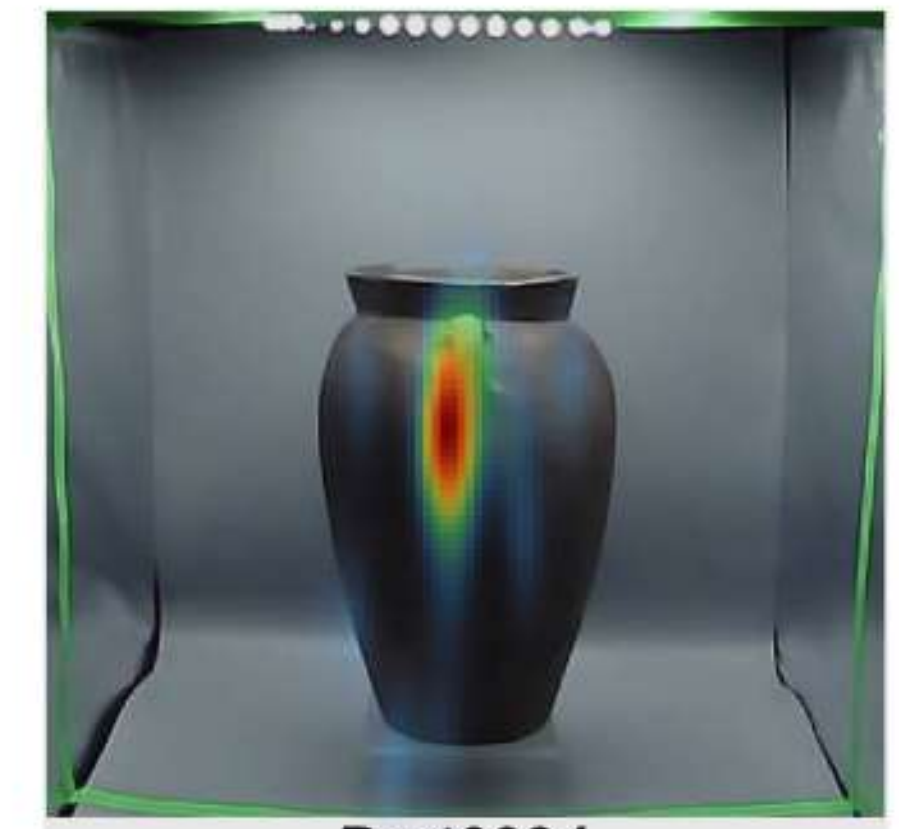
Part0094

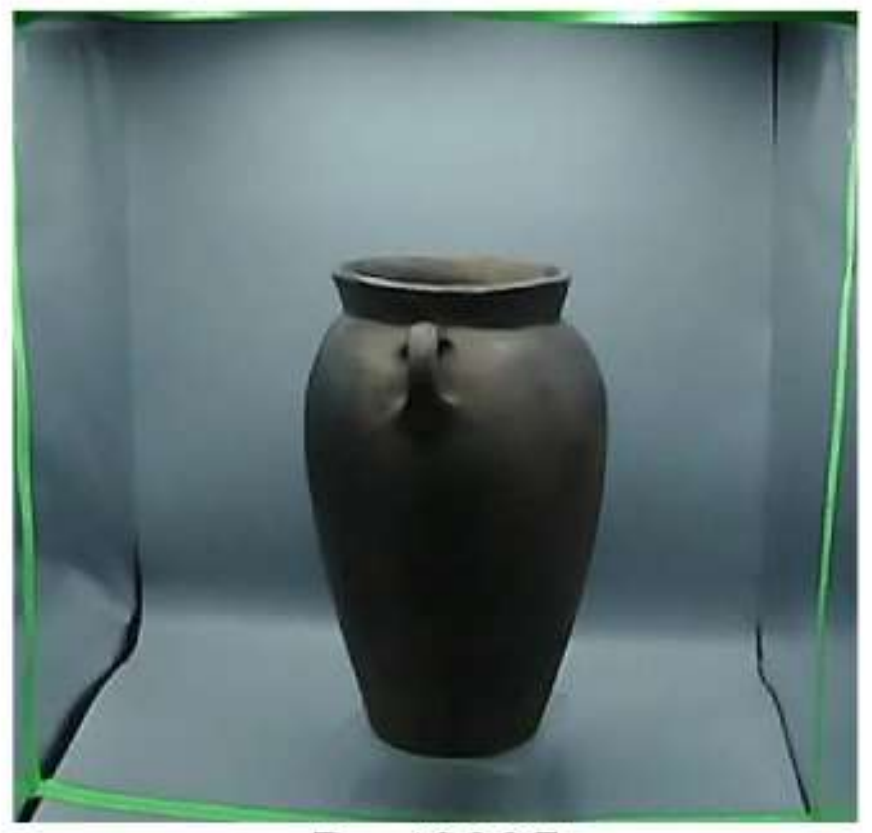
Part0095

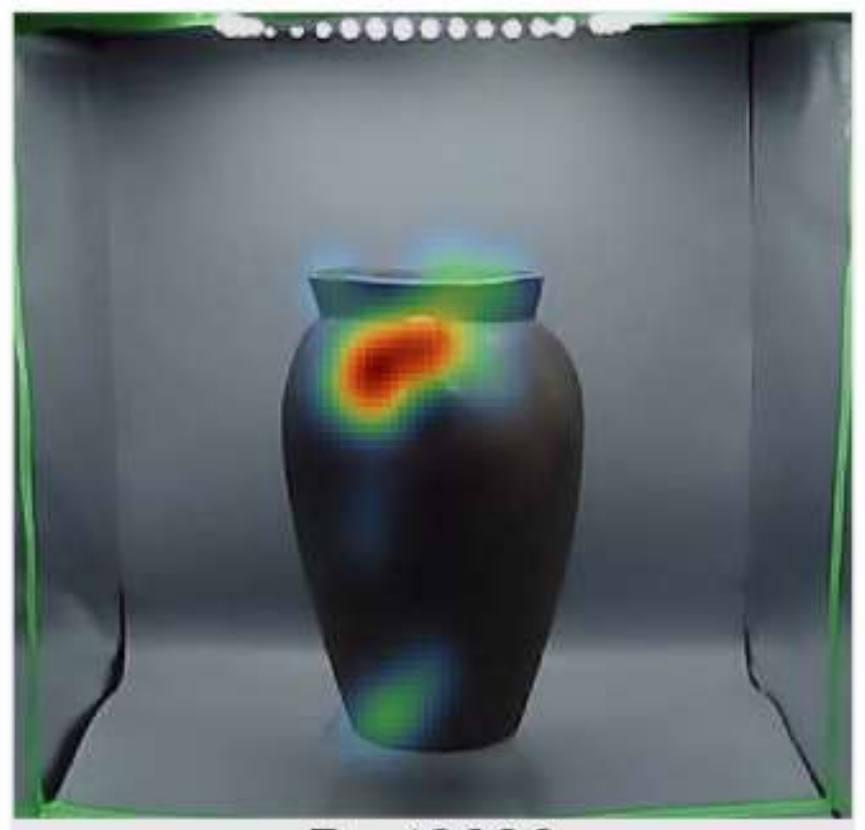
Part0096

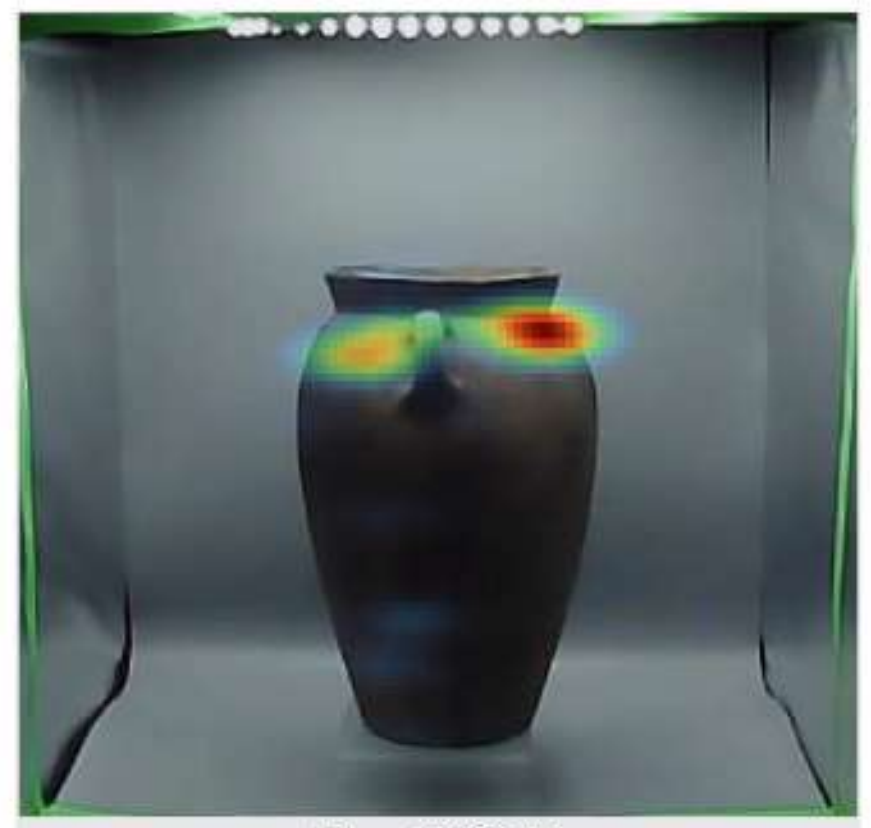
Part0097

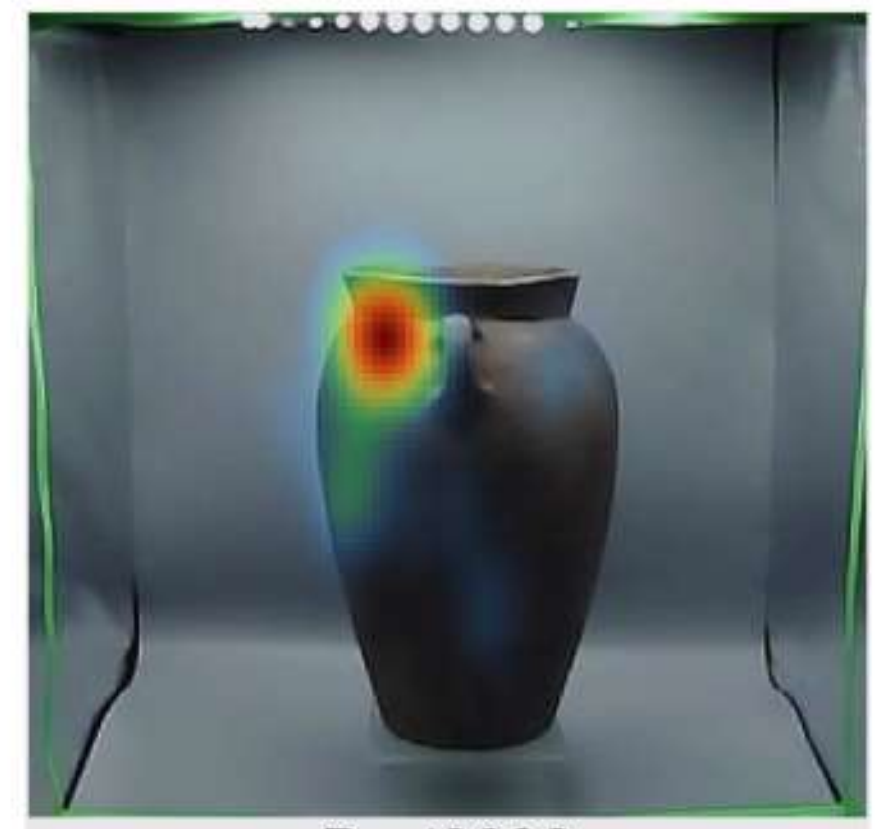
Part0098

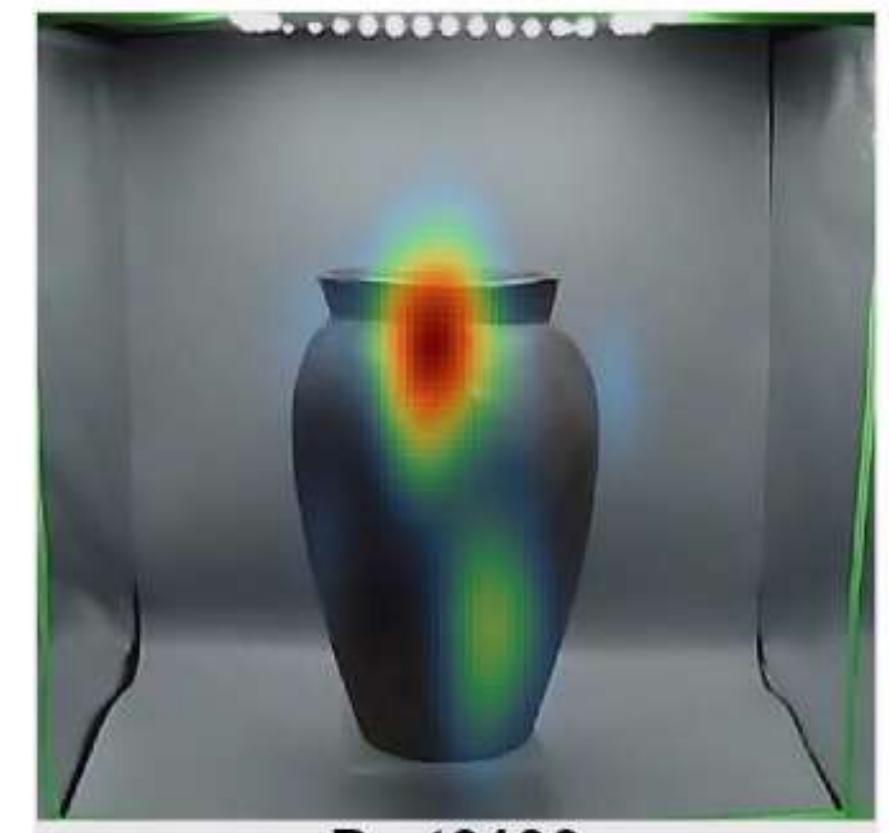
Part0100

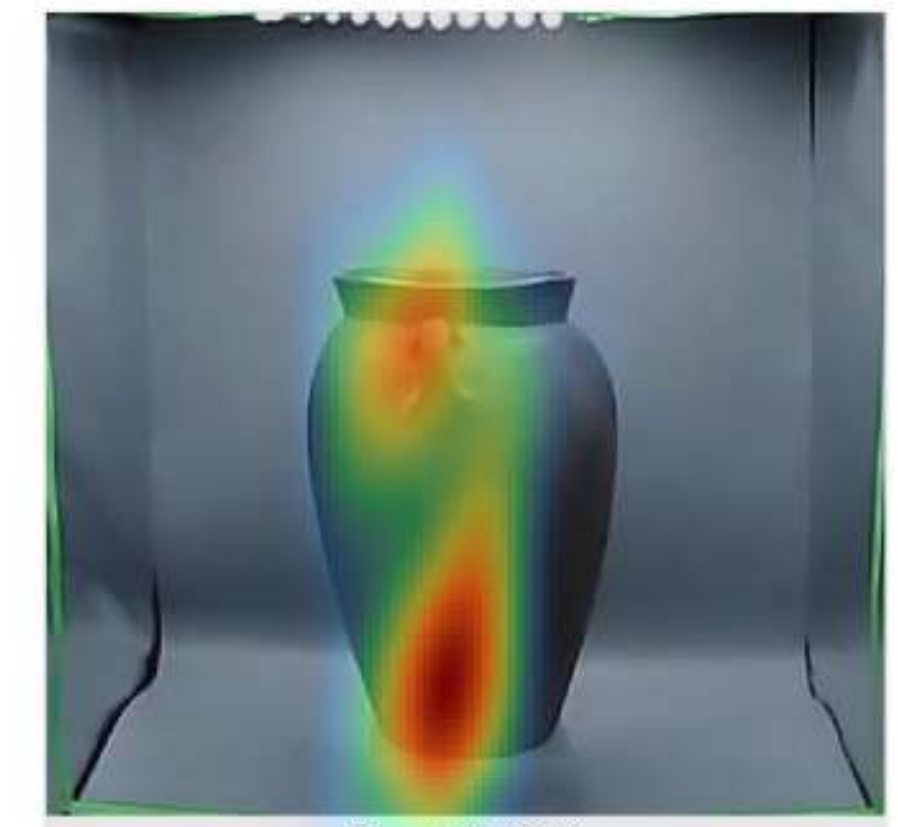
Part0101

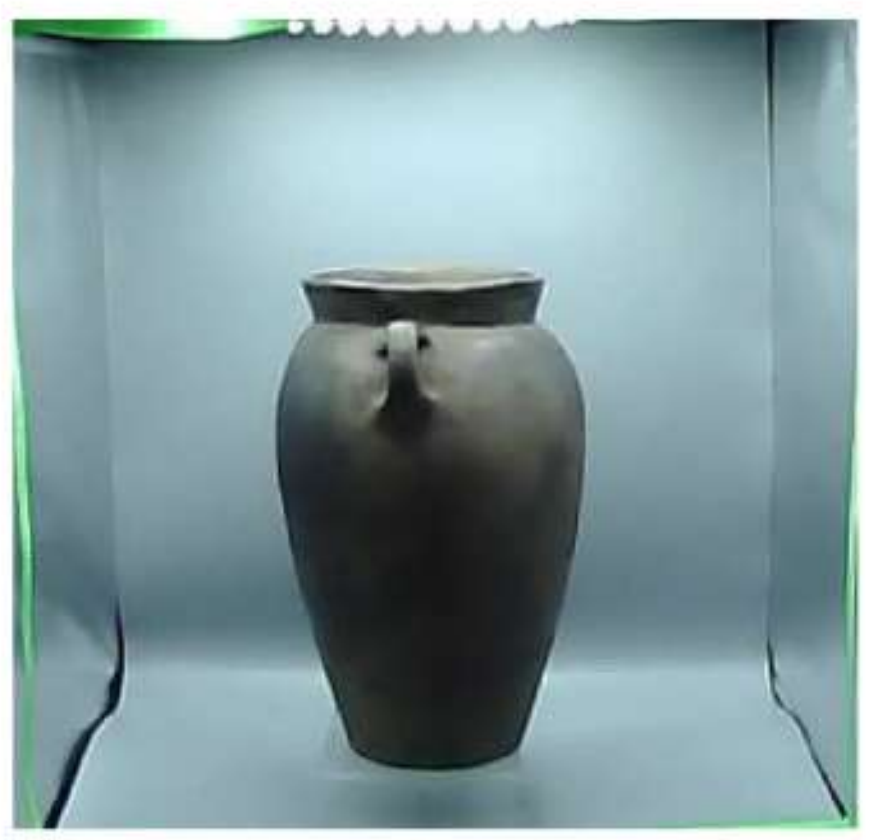
Part0102

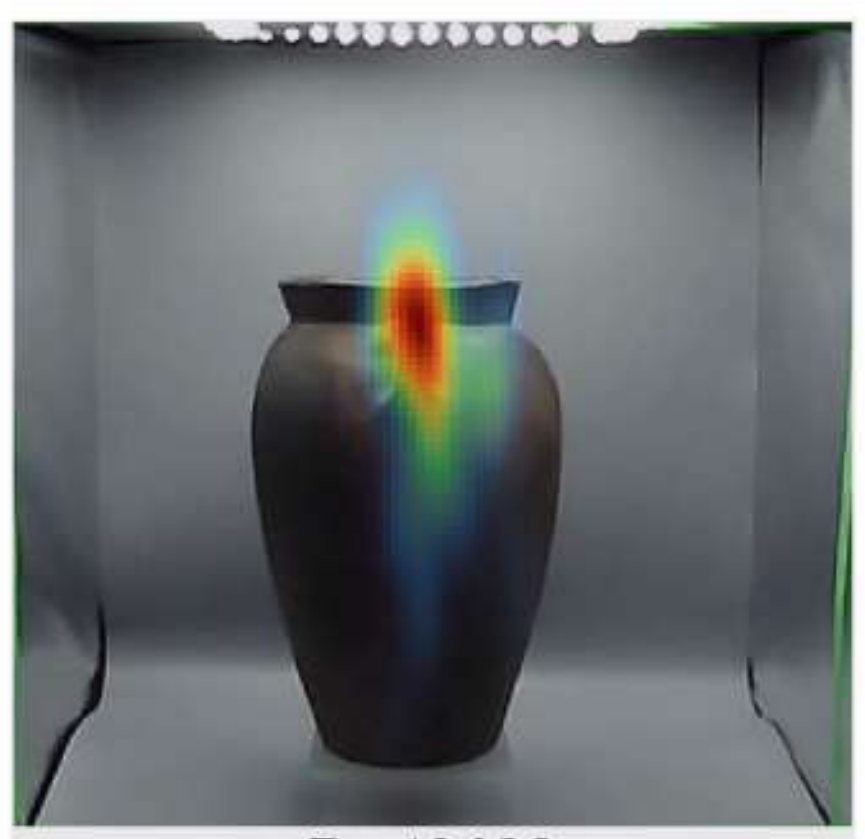
Part0103

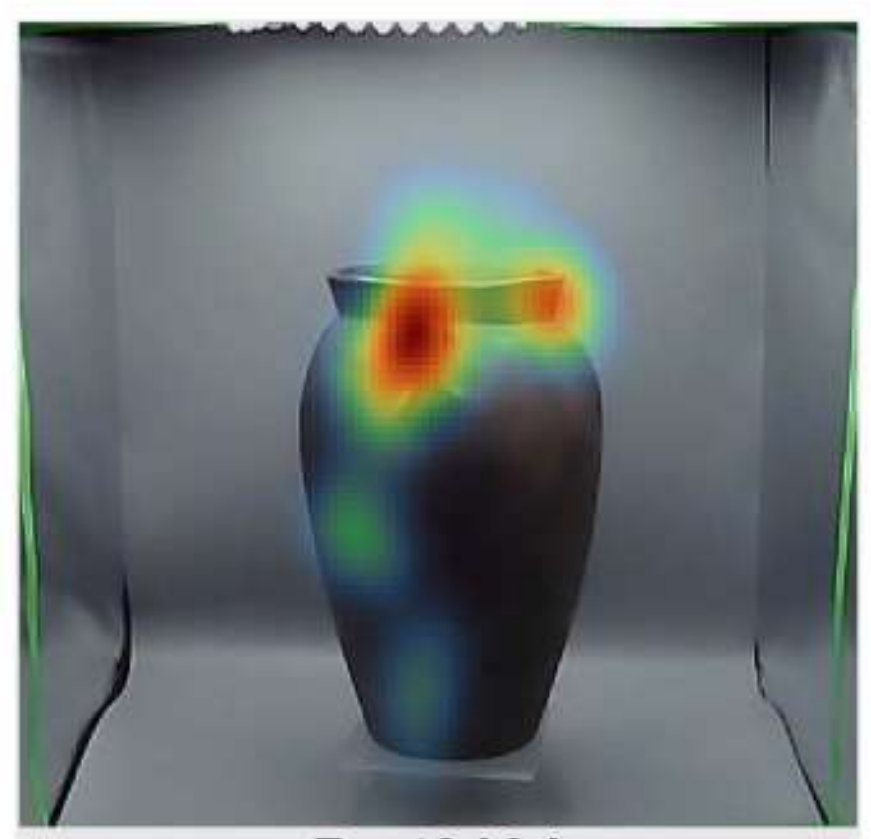
Part0104

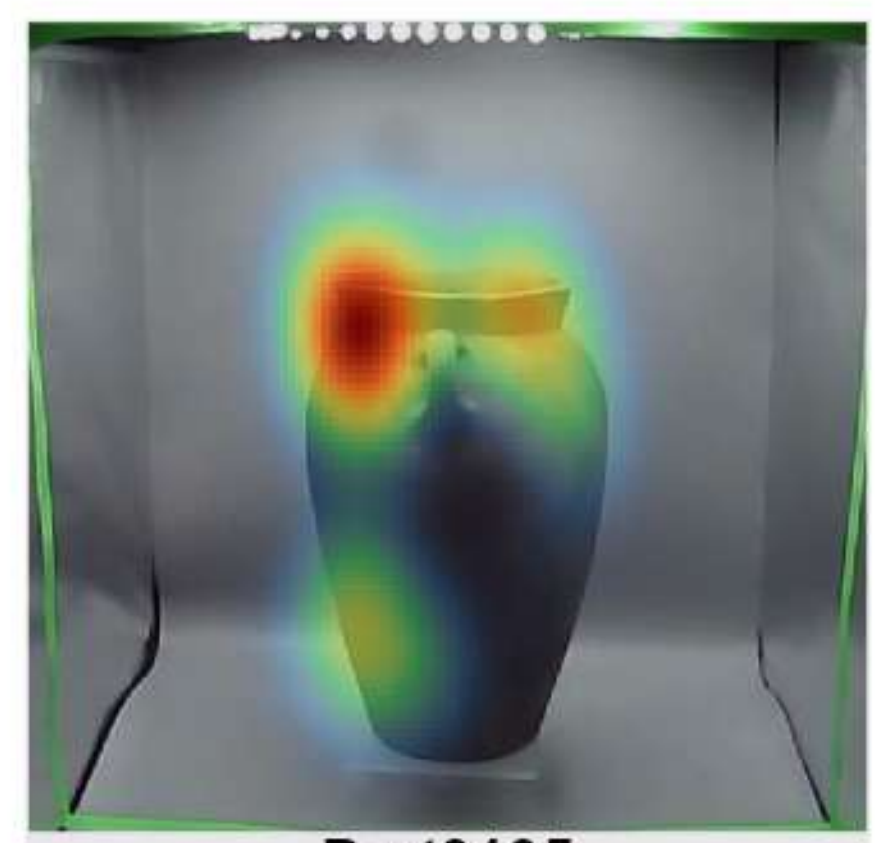
Part0105

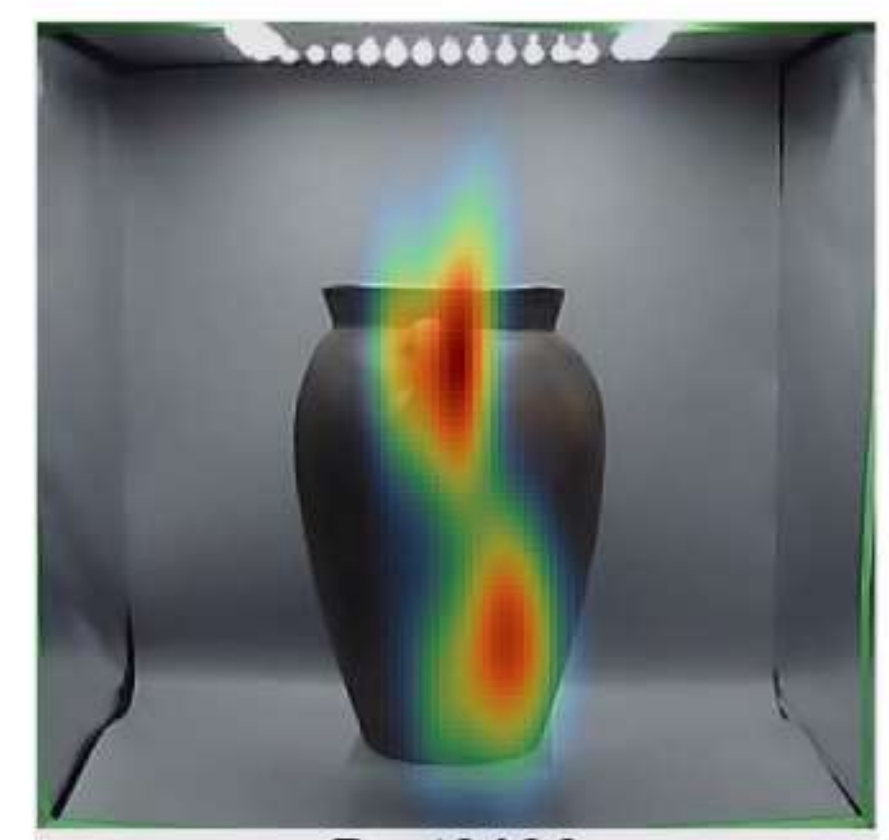
Part0106

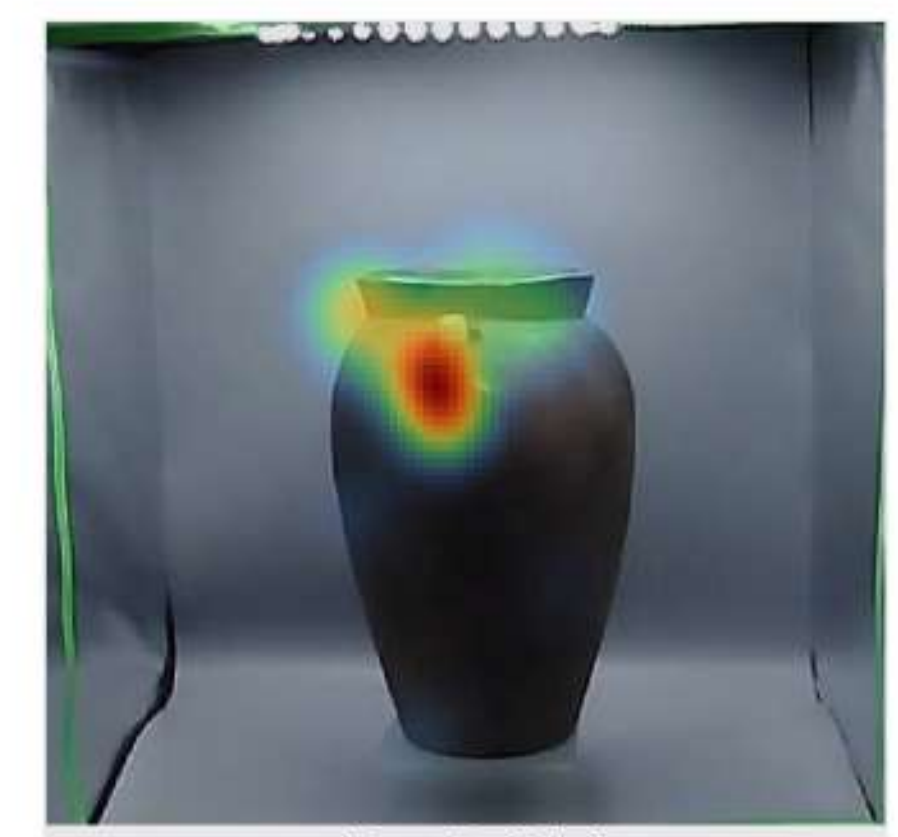
Part0107

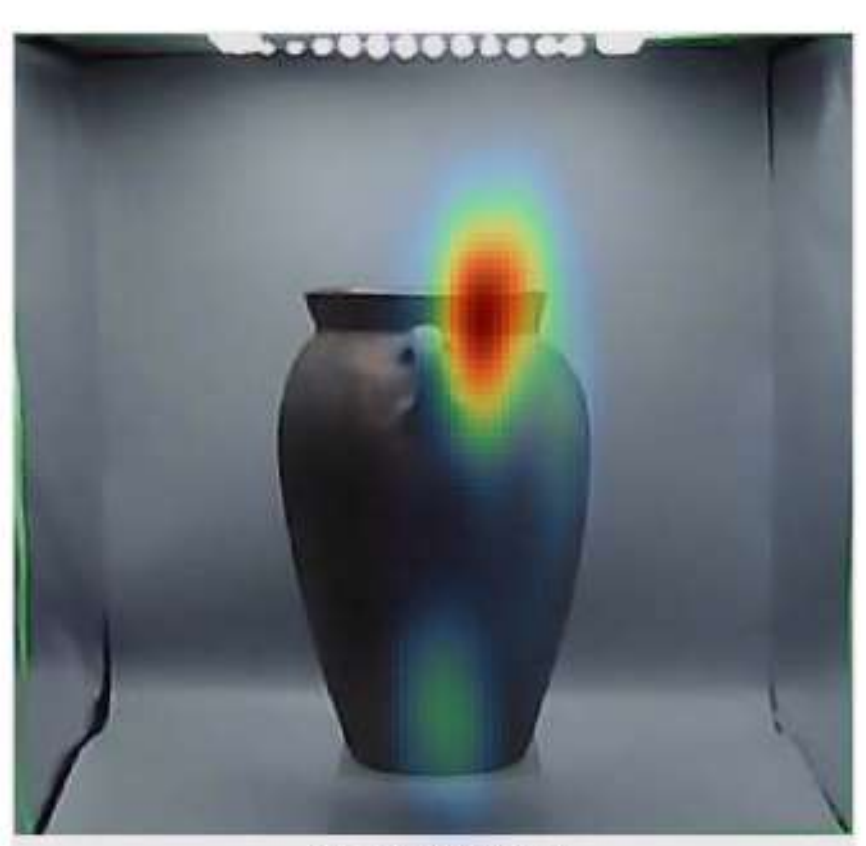
Part0108

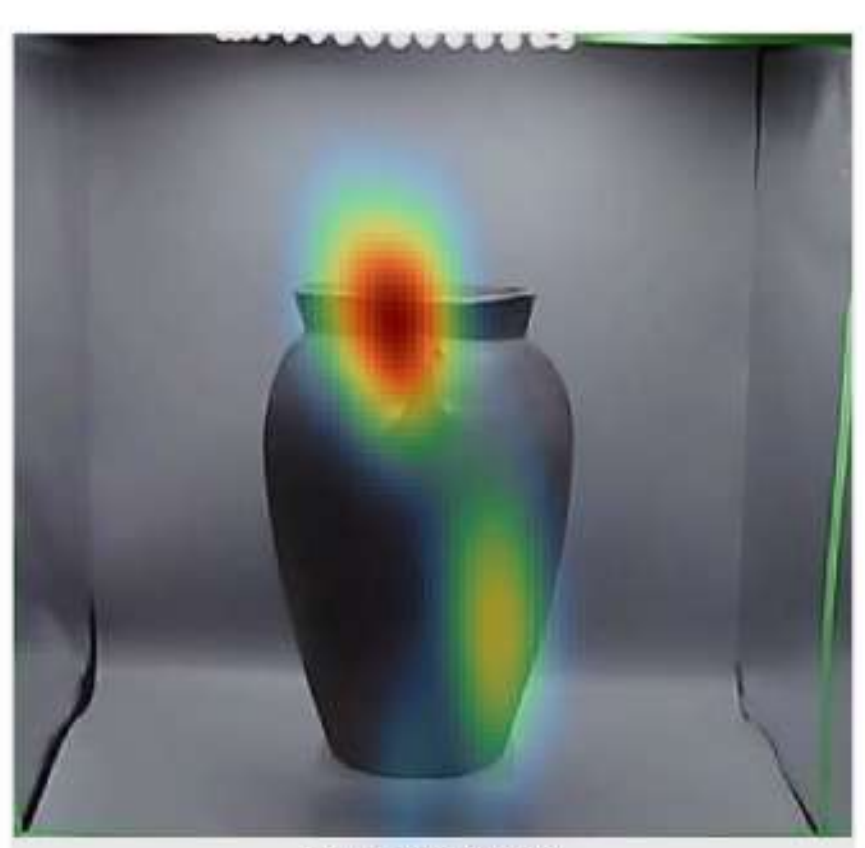
Part0109

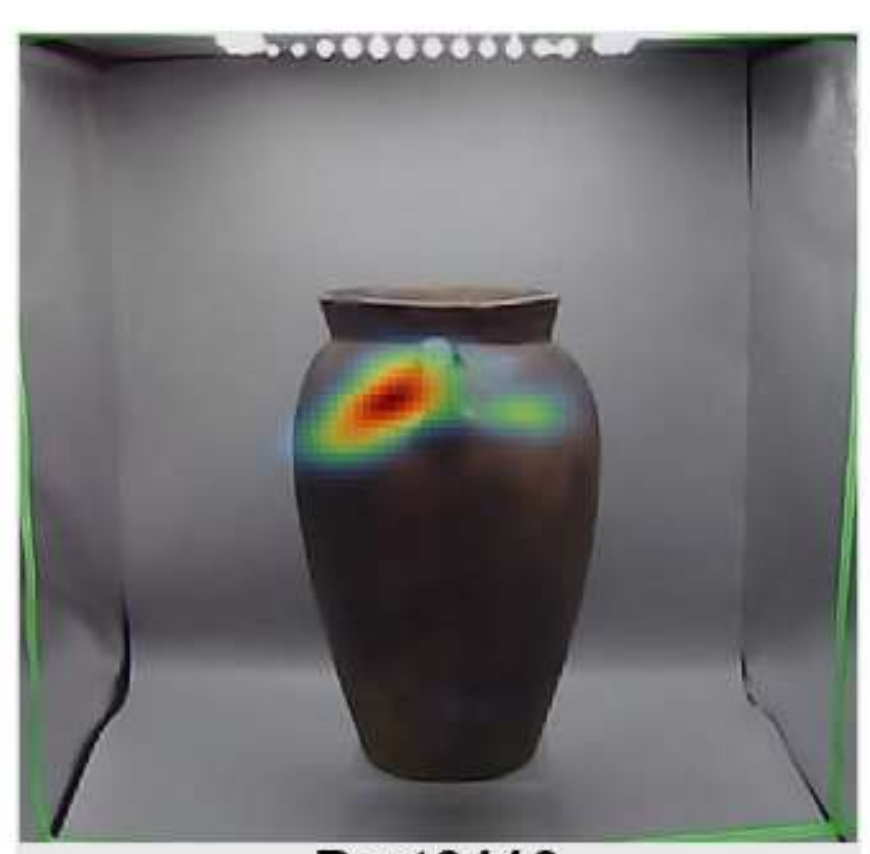
Part0110

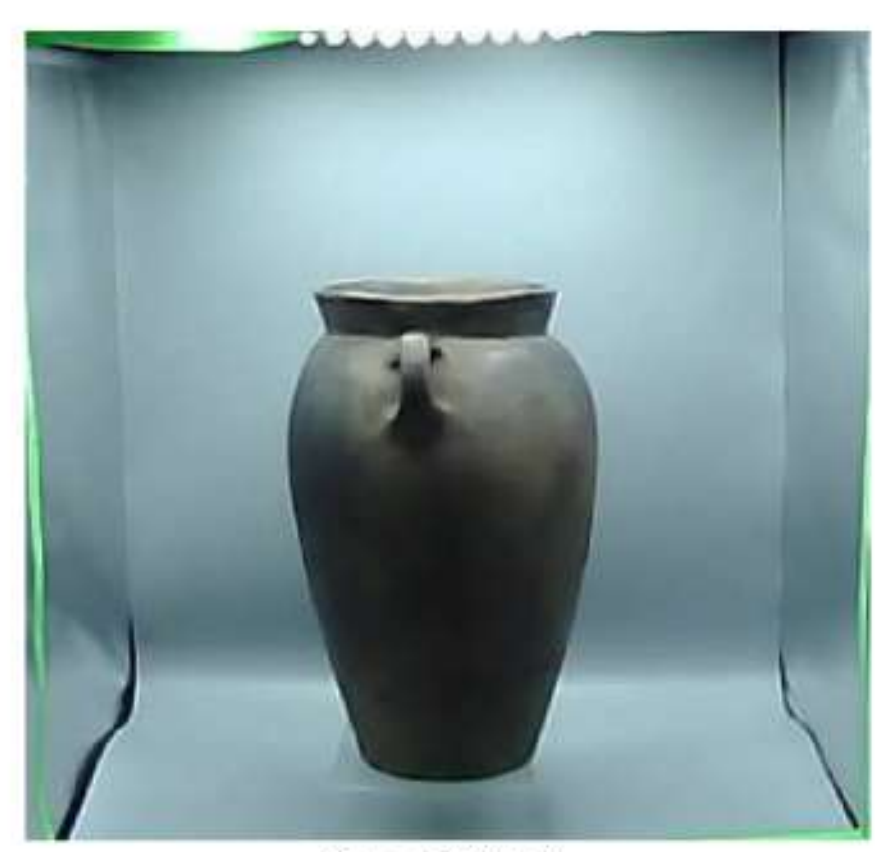
Part0111

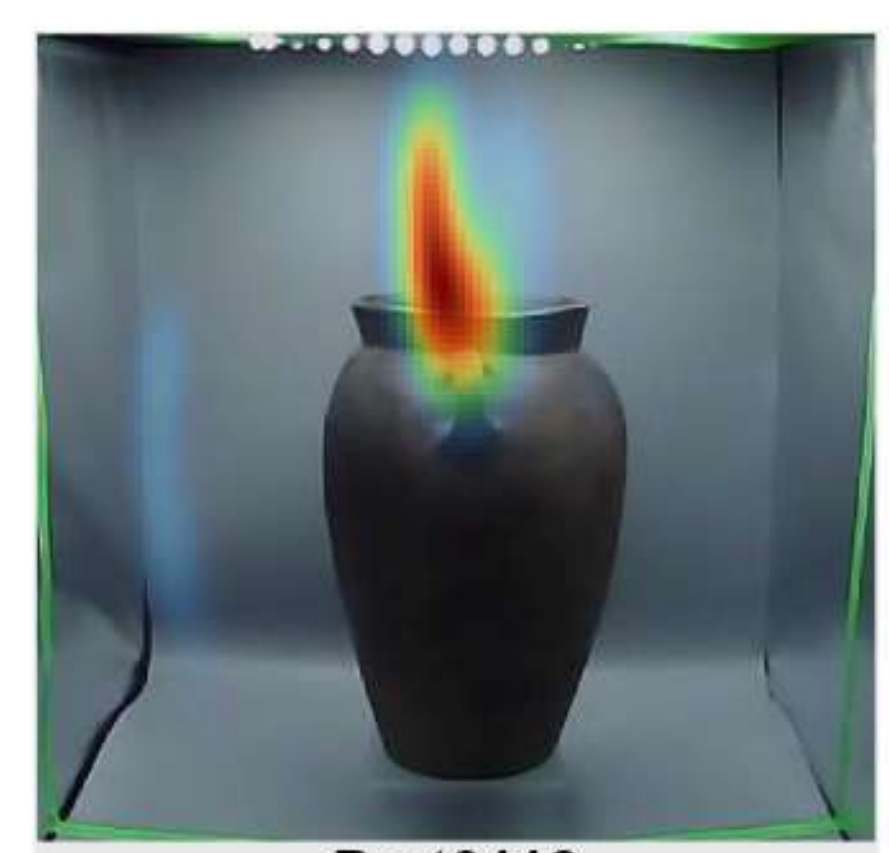
Part0112

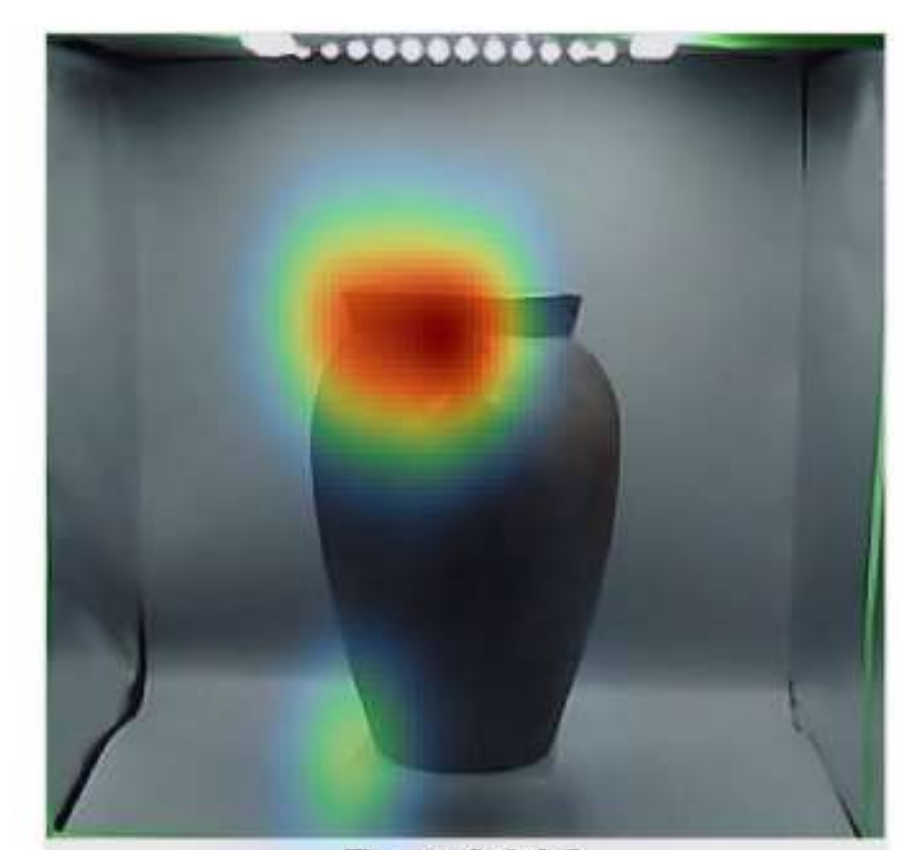
Part0113

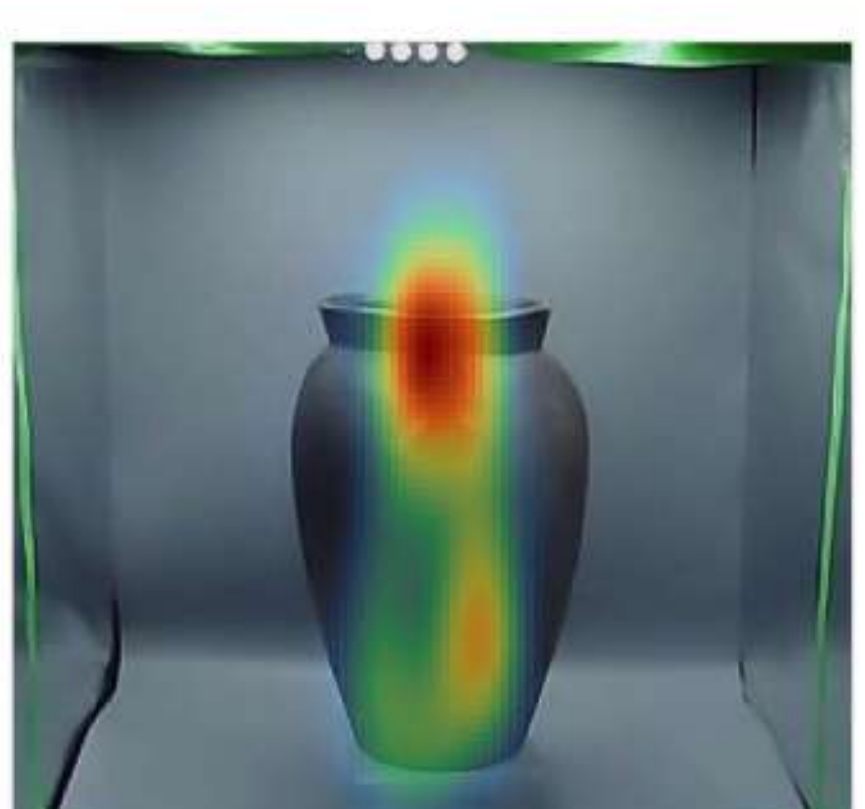
Part0114

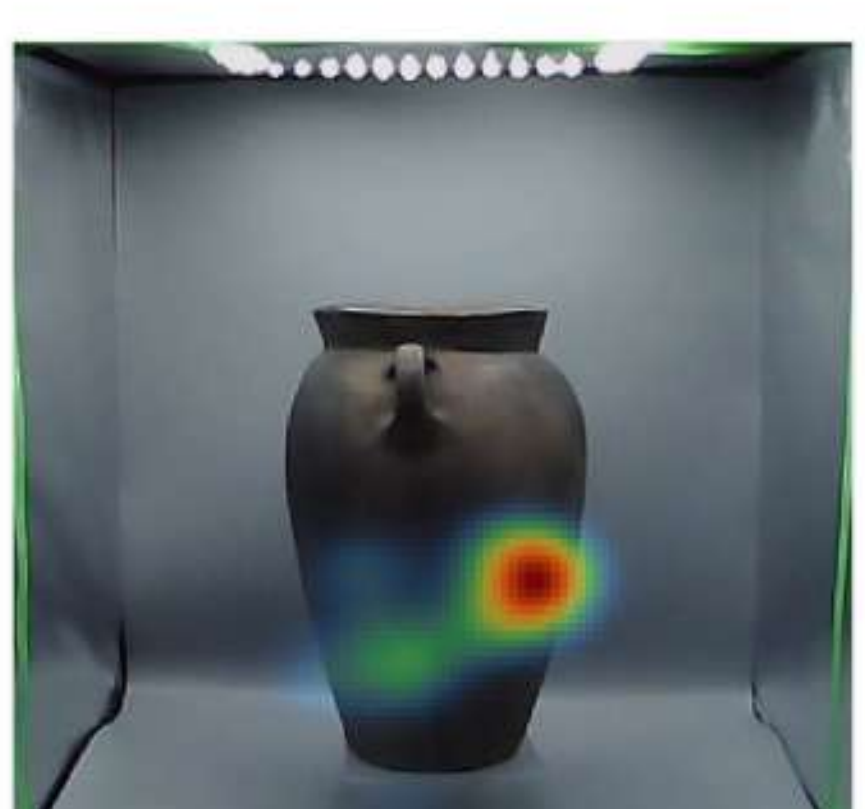
Part0115

Obj0262

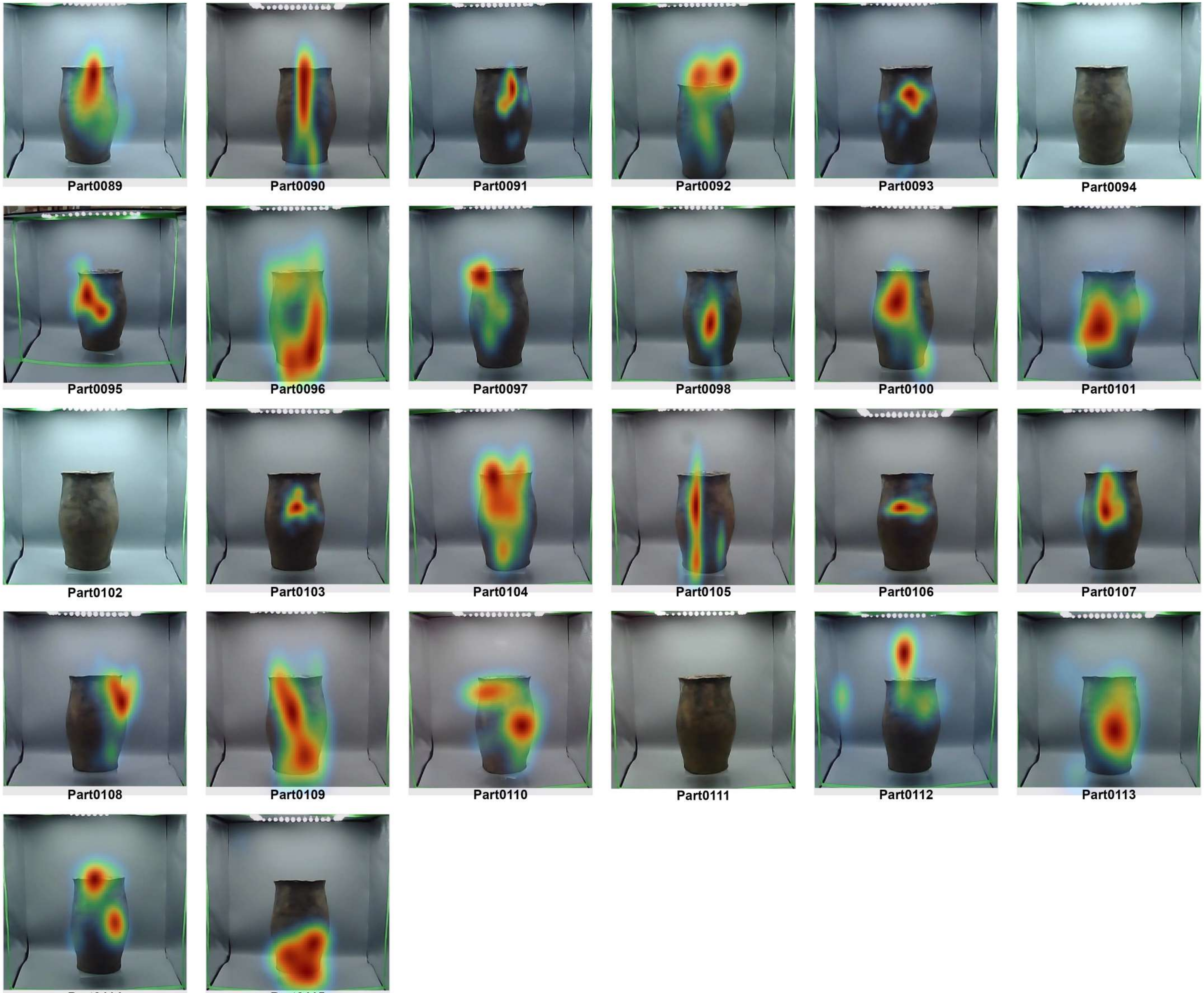
Part0089
Part0090
Part0091
Part0092
Part0093
Part0094
Part0095
Part0096
Part0097
Part0098
Part0100
Part0101
Part0102
Part0103
Part0104
Part0105
Part0106
Part0107
Part0108
Part0109
Part0110
Part0111
Part0112
Part0113
Part0114
Part0115

Obj0264

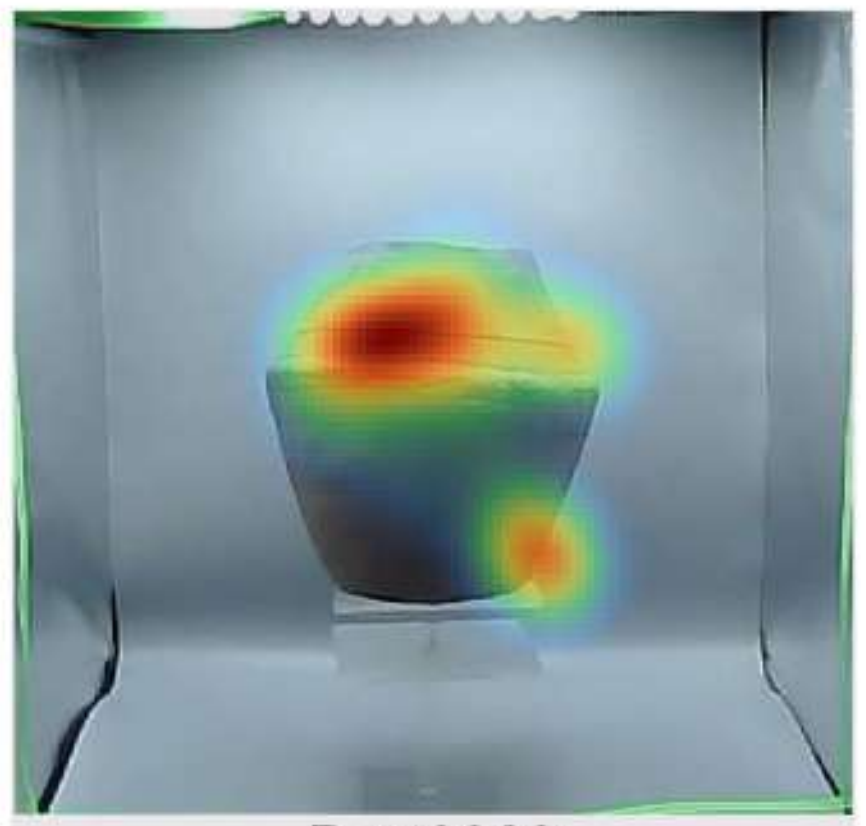
Part0089
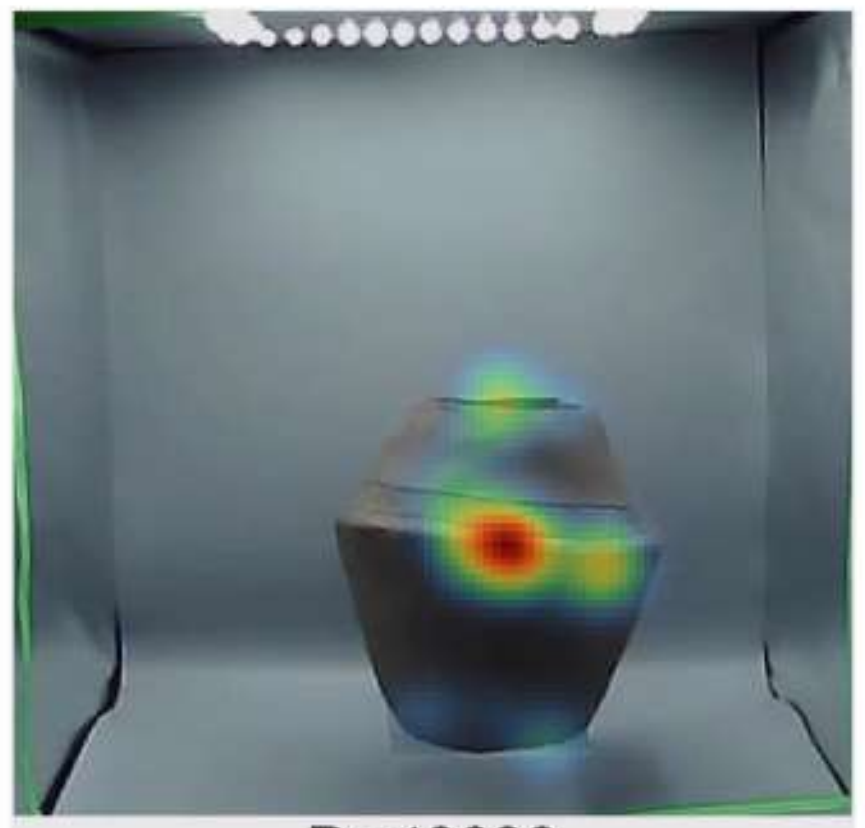
Part0090
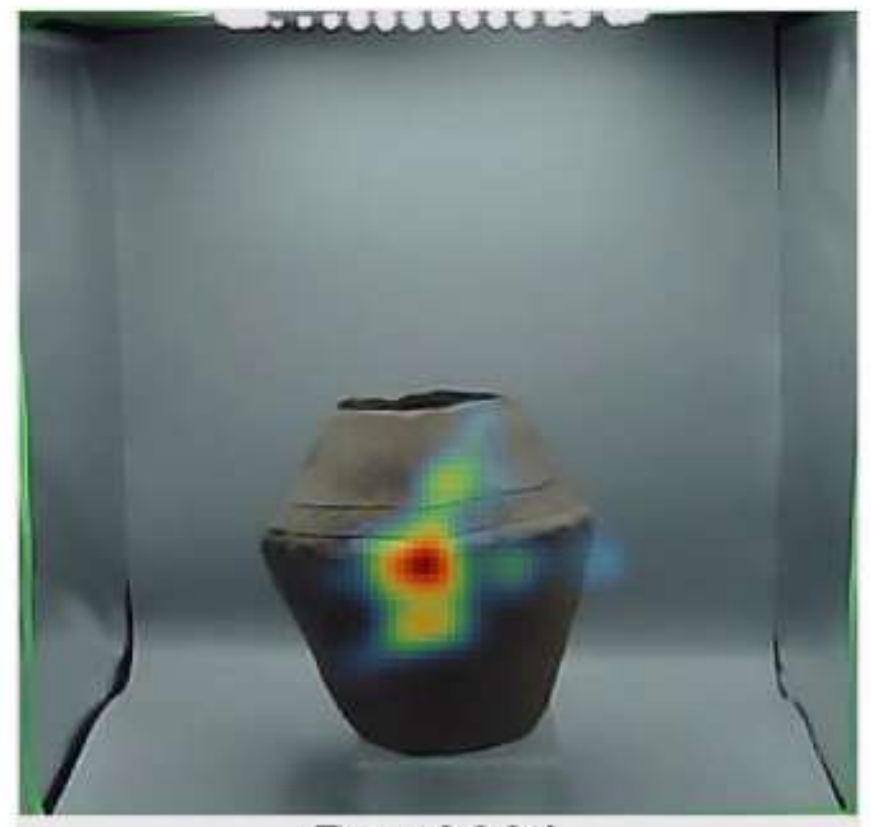
Part0091
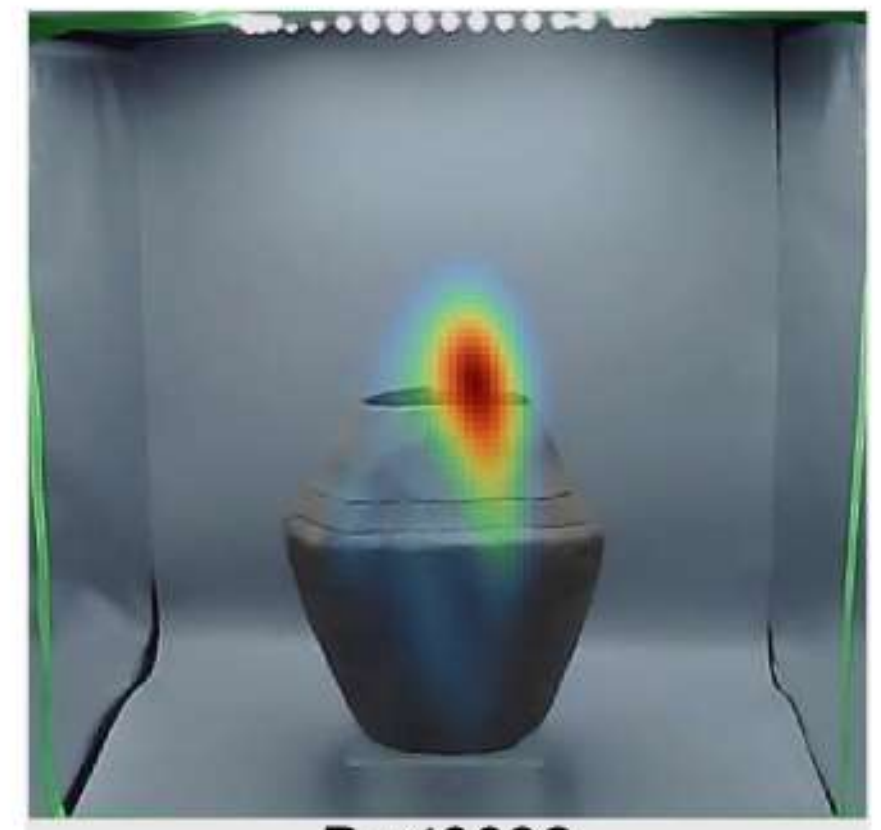
Part0092
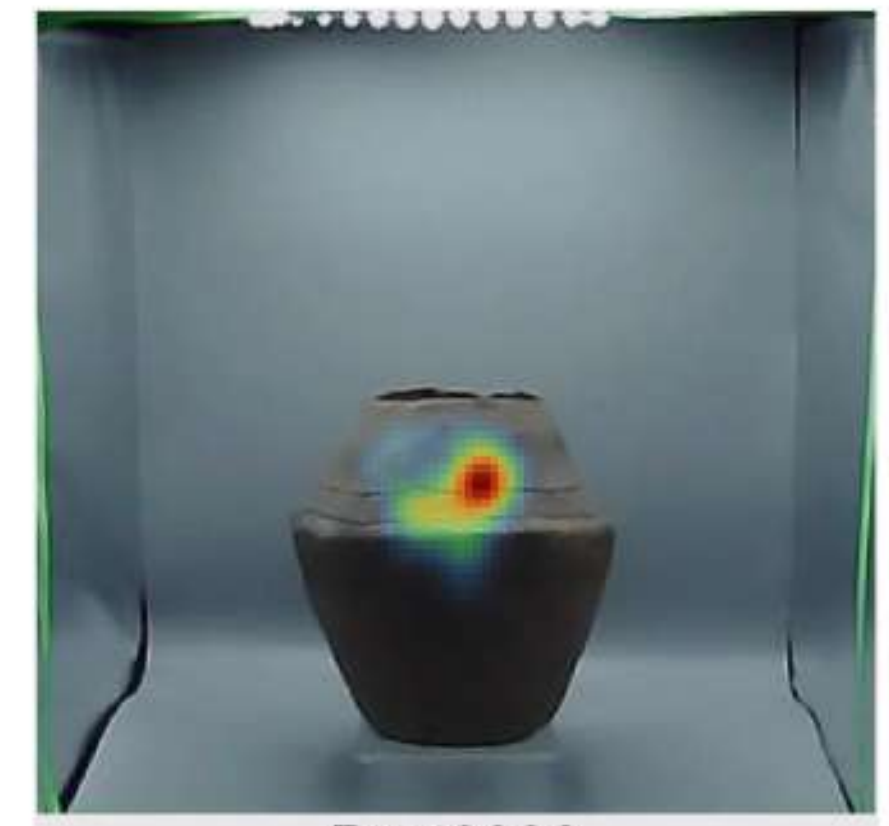
Part0093
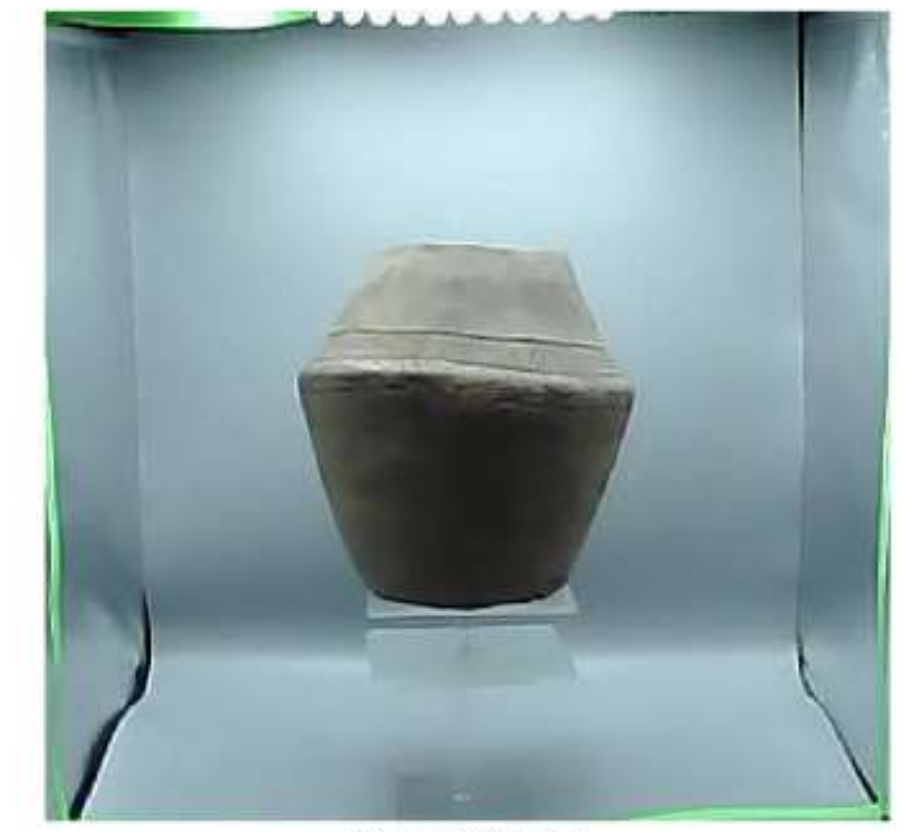
Part0094
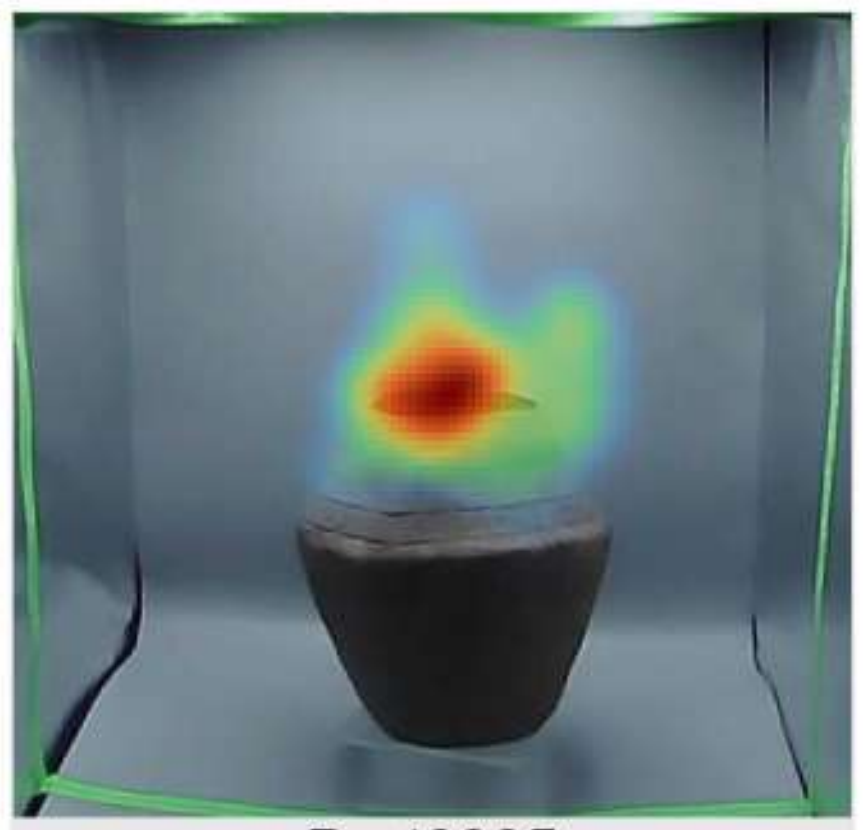
Part0095
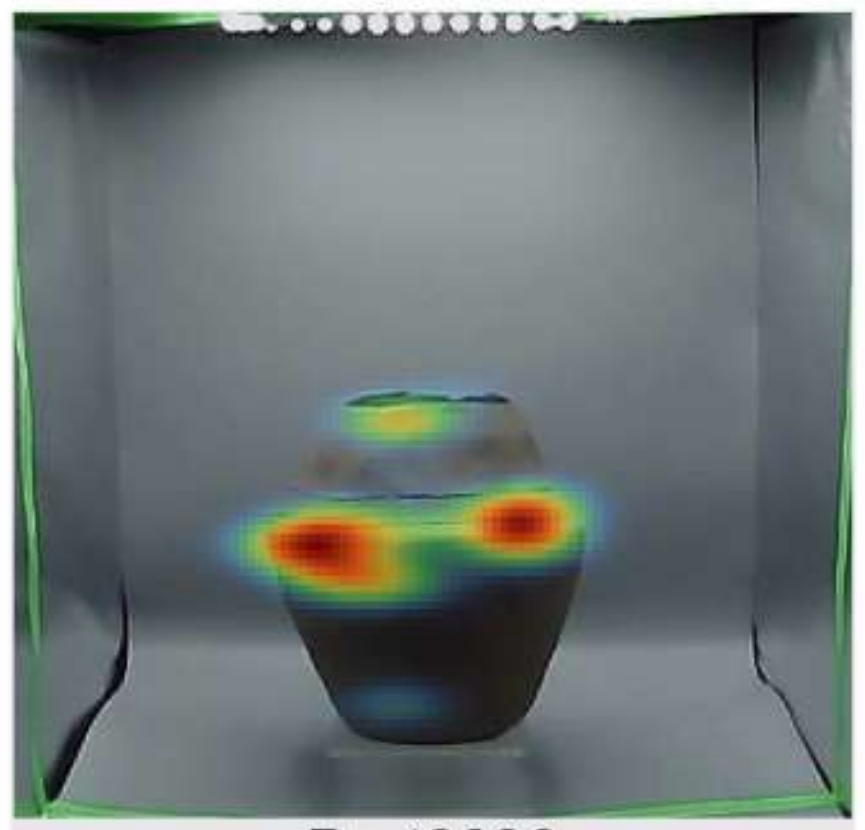
Part0096
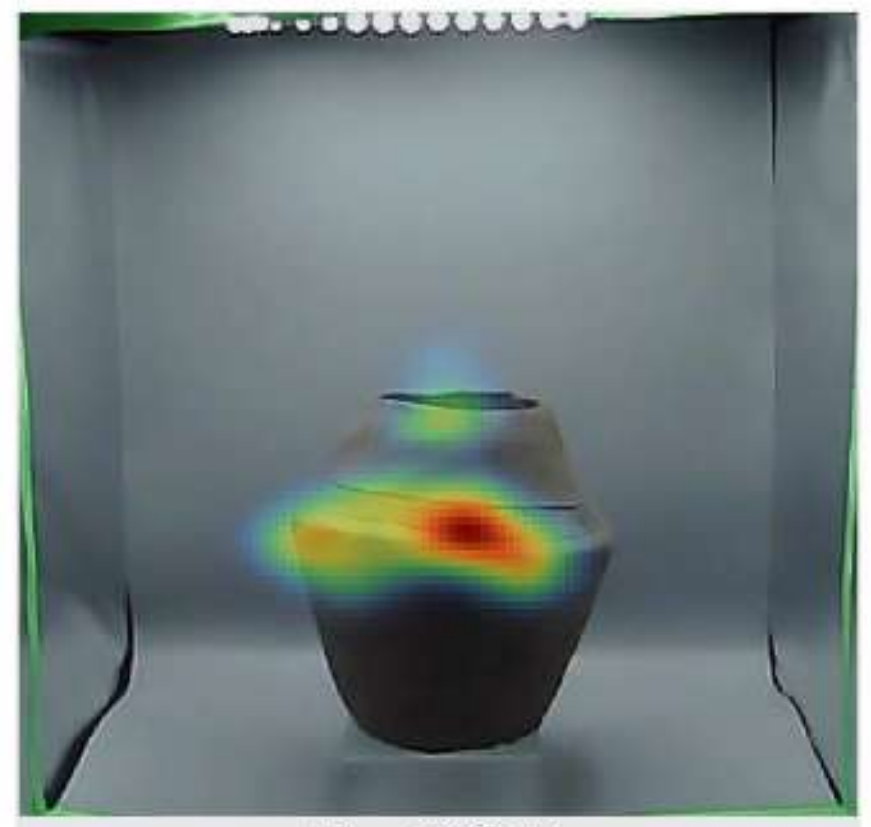
Part0097
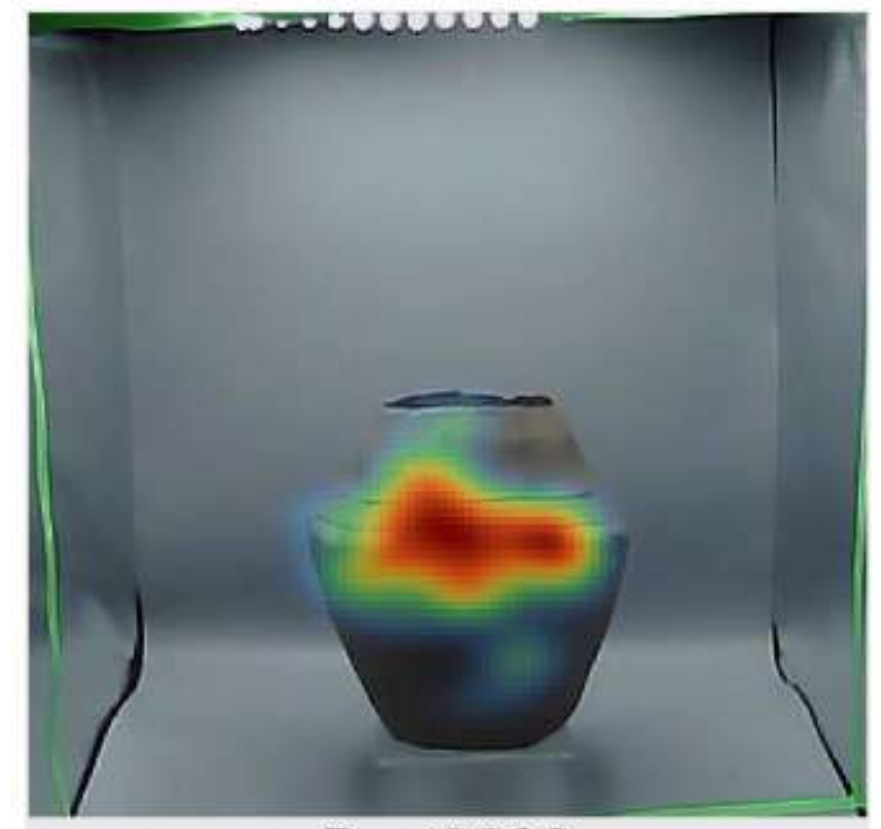
Part0098
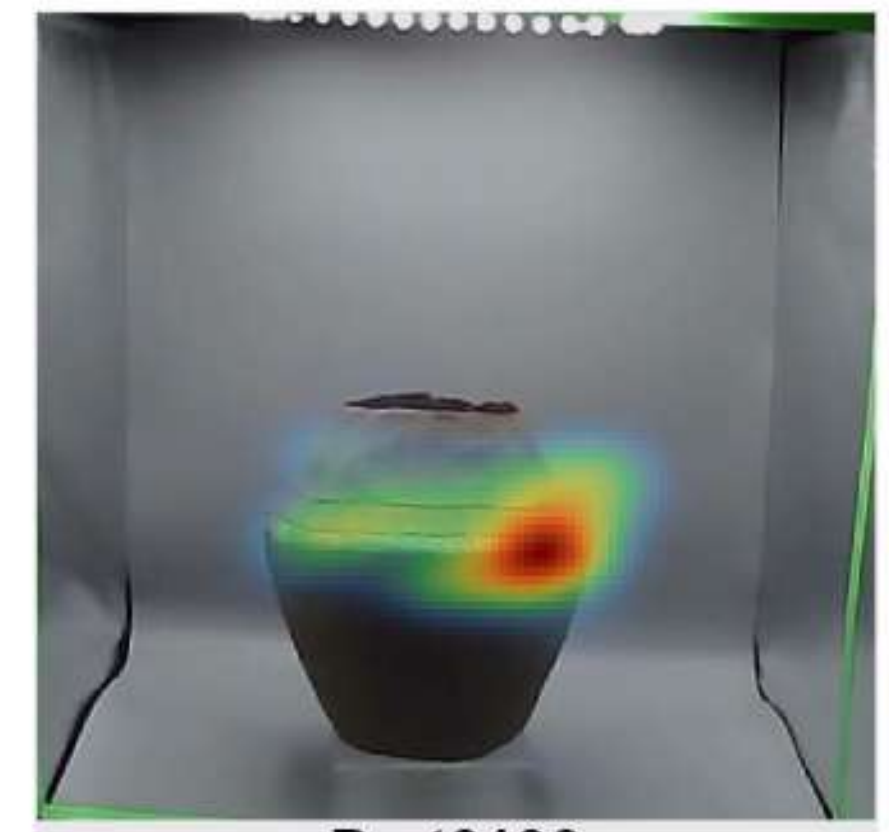
Part0100
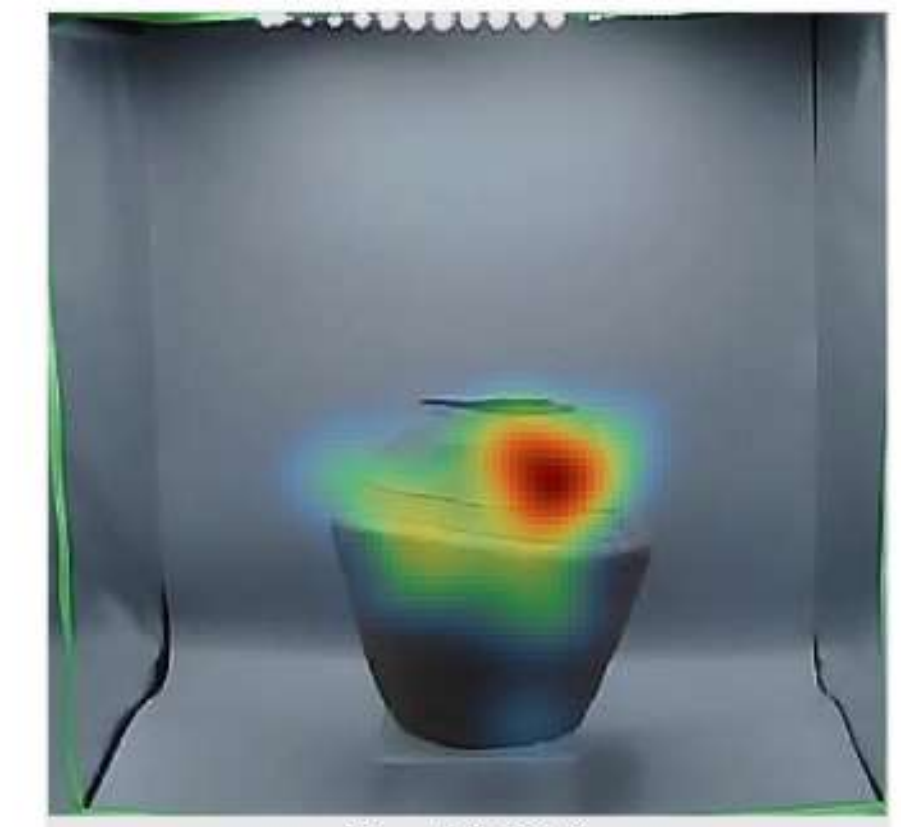
Part0101
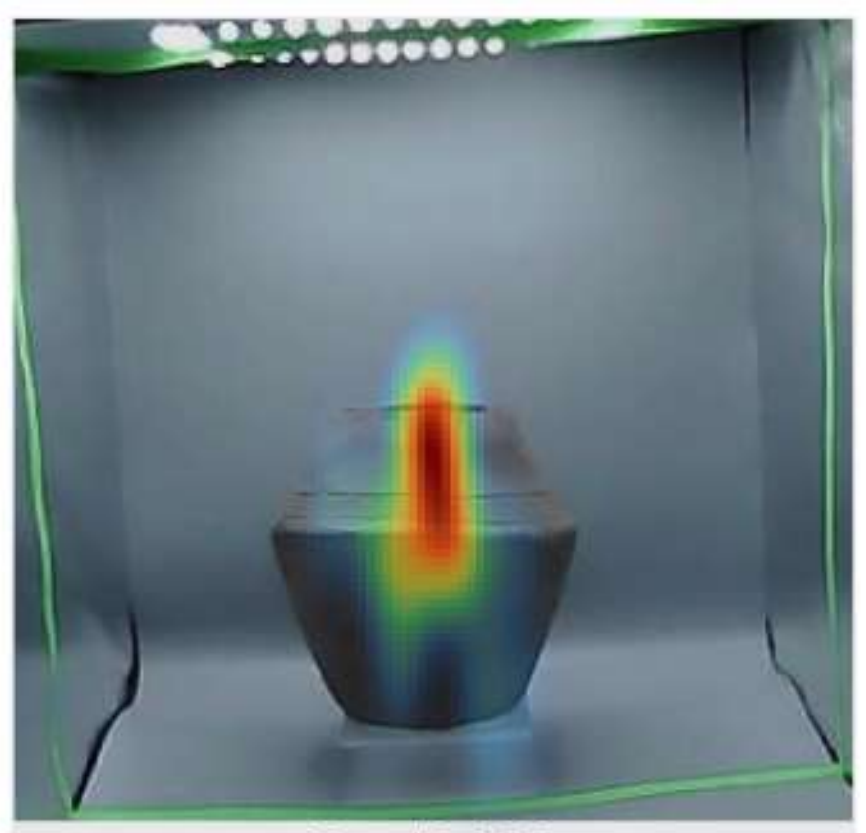
Part0102
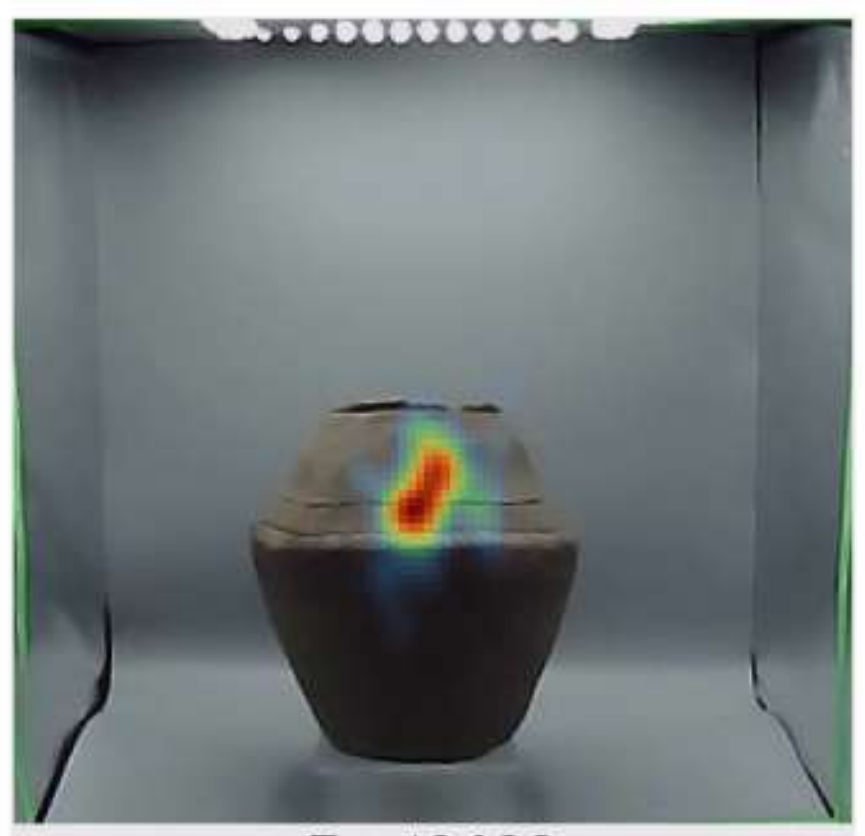
Part0103
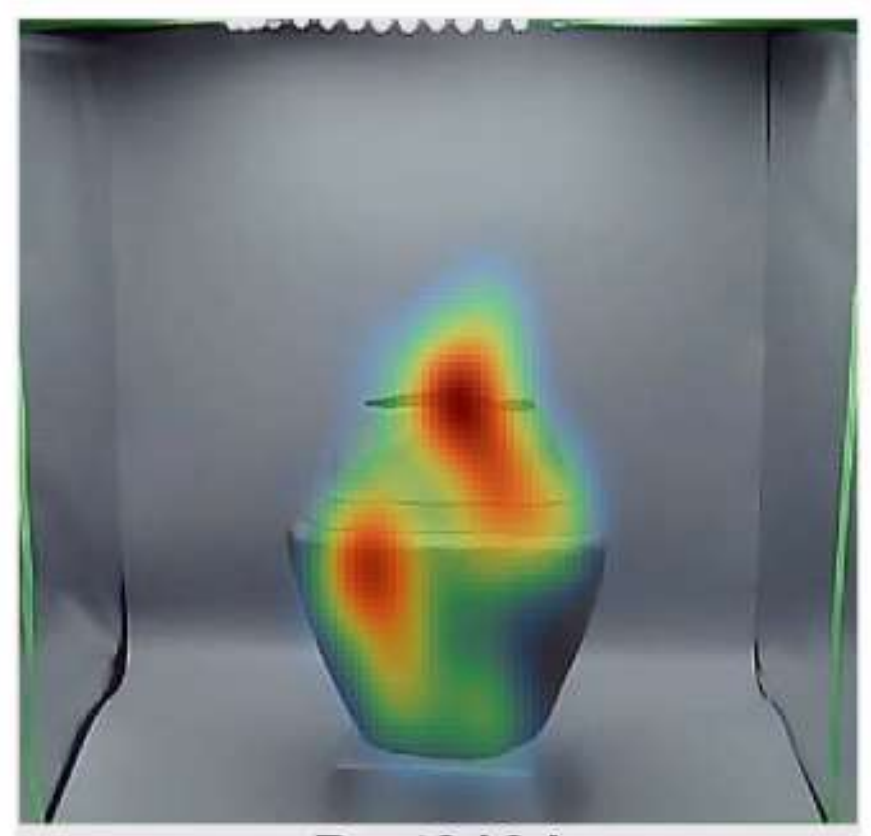
Part0104
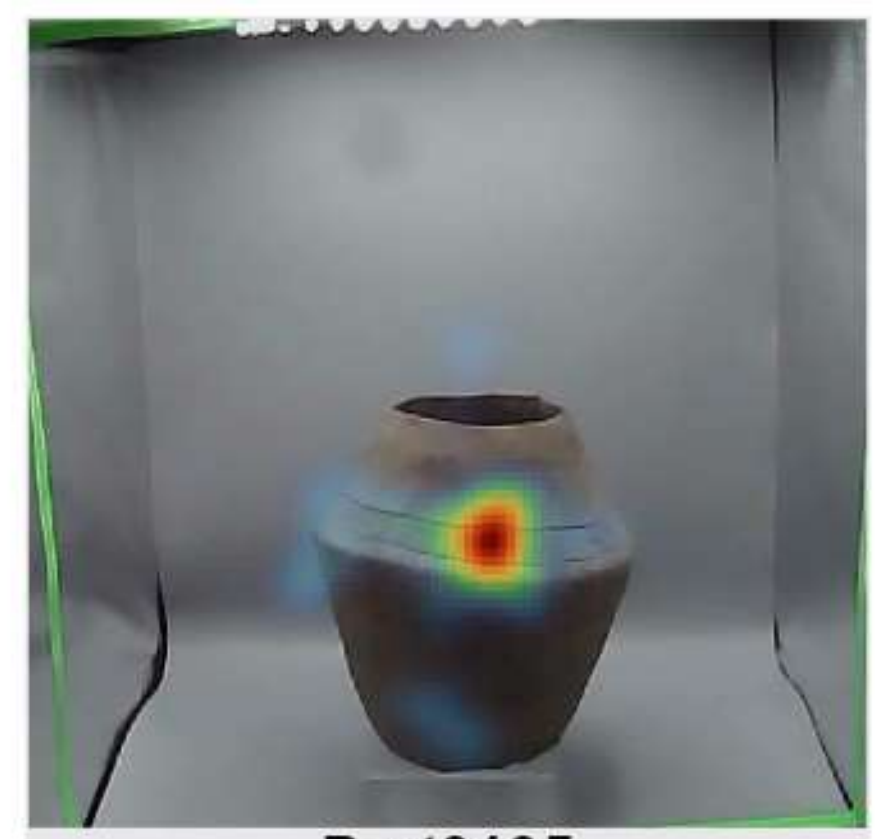
Part0105
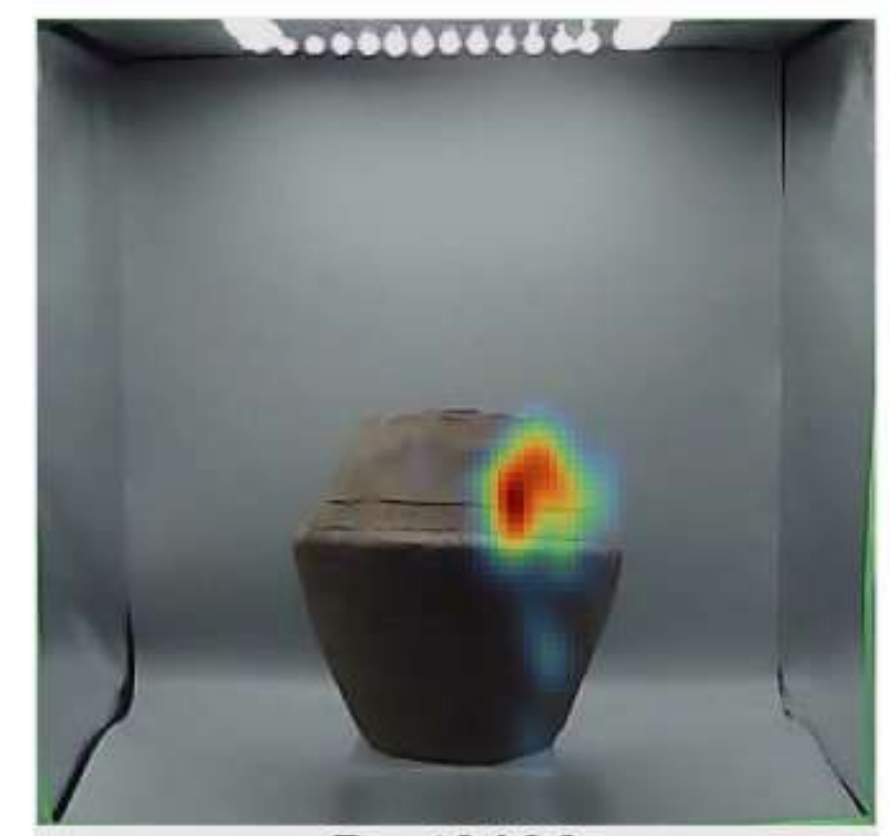
Part0106
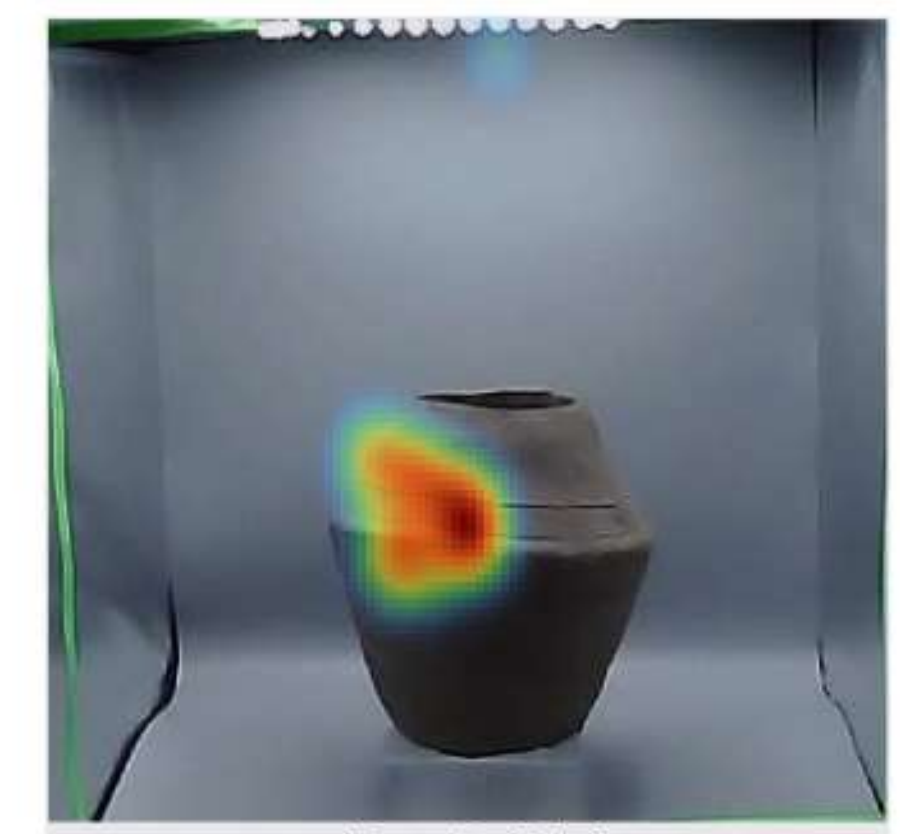
Part0107
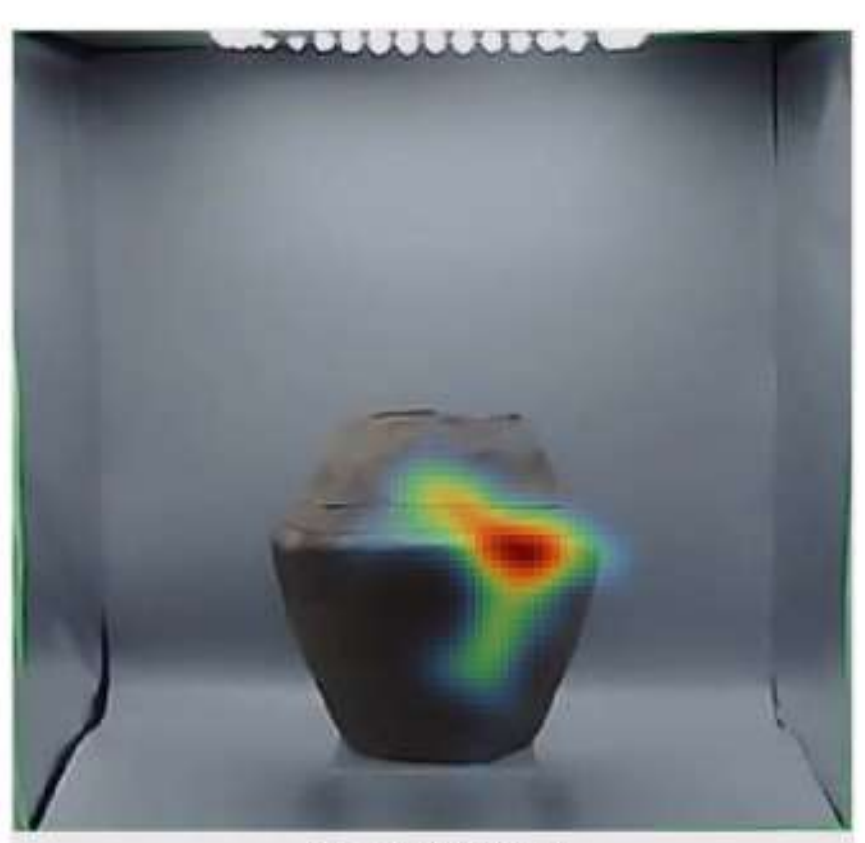
Part0108
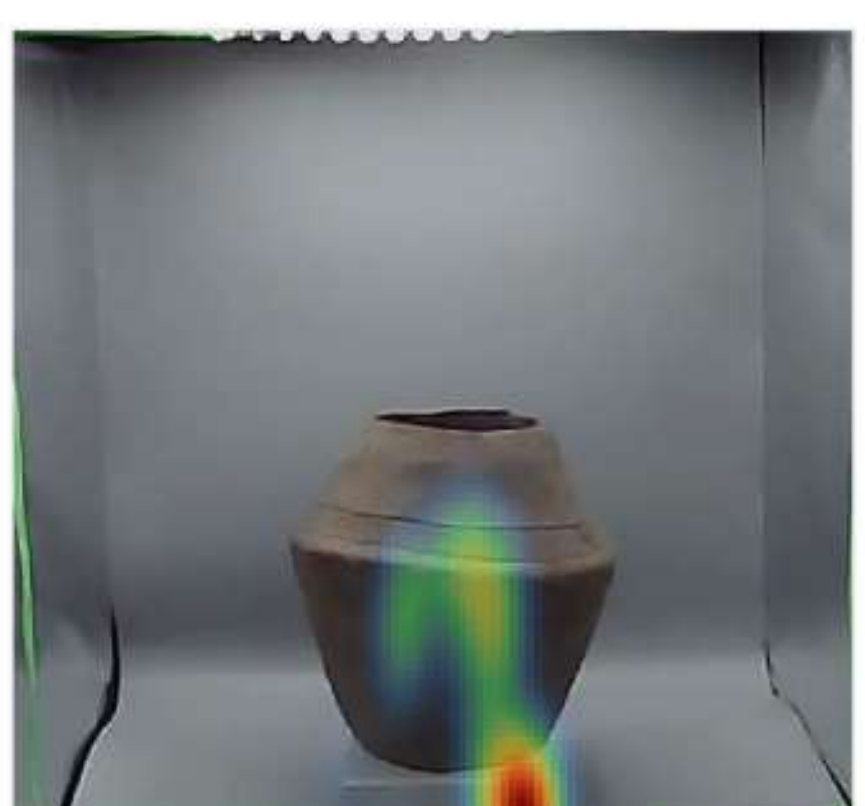
Part0109
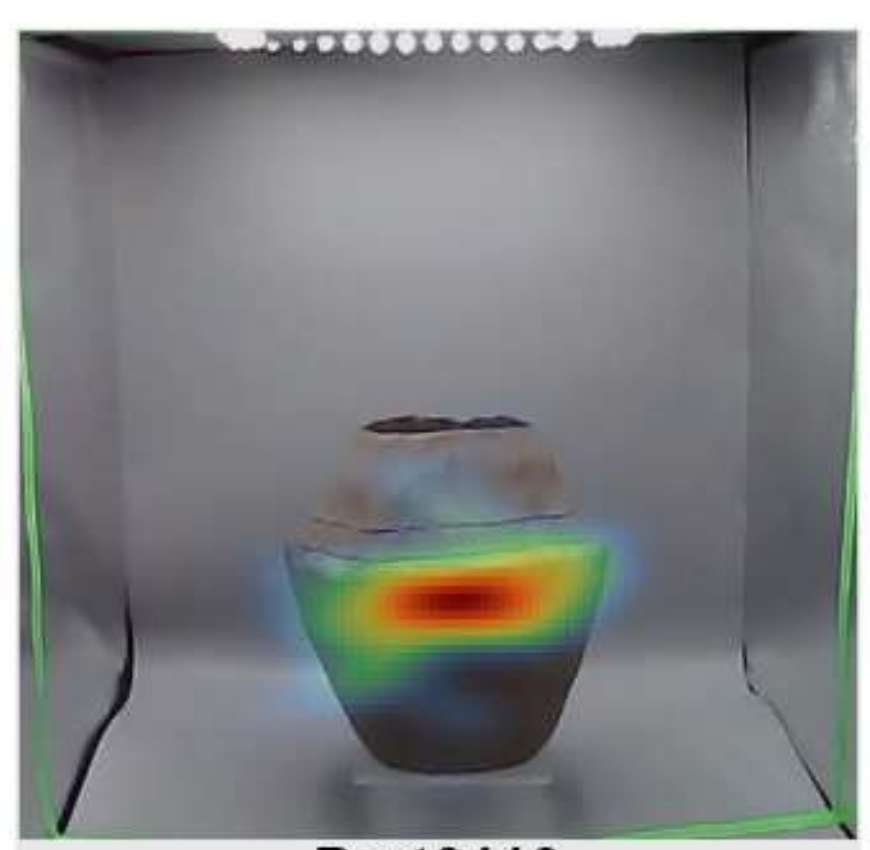
Part0110
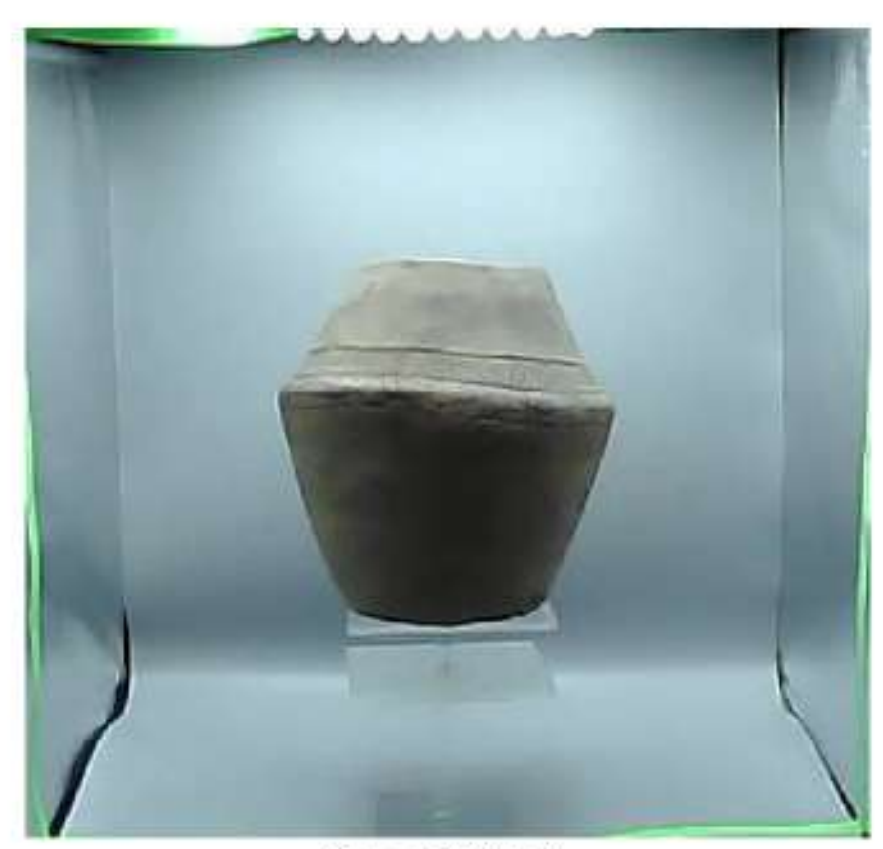
Part0111
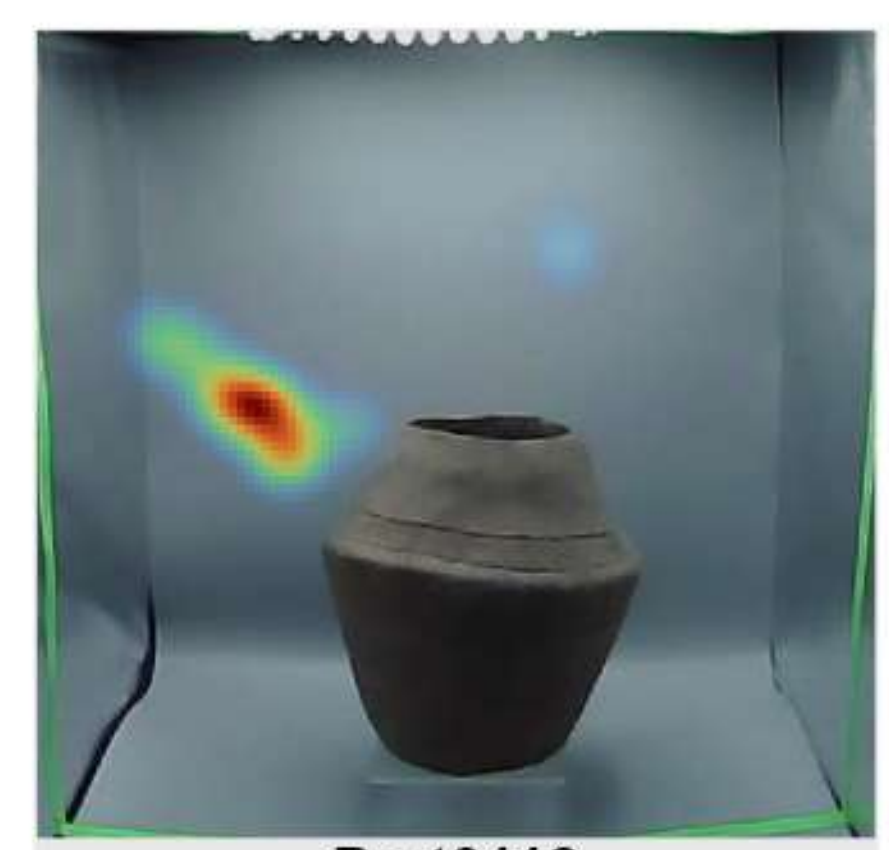
Part0112
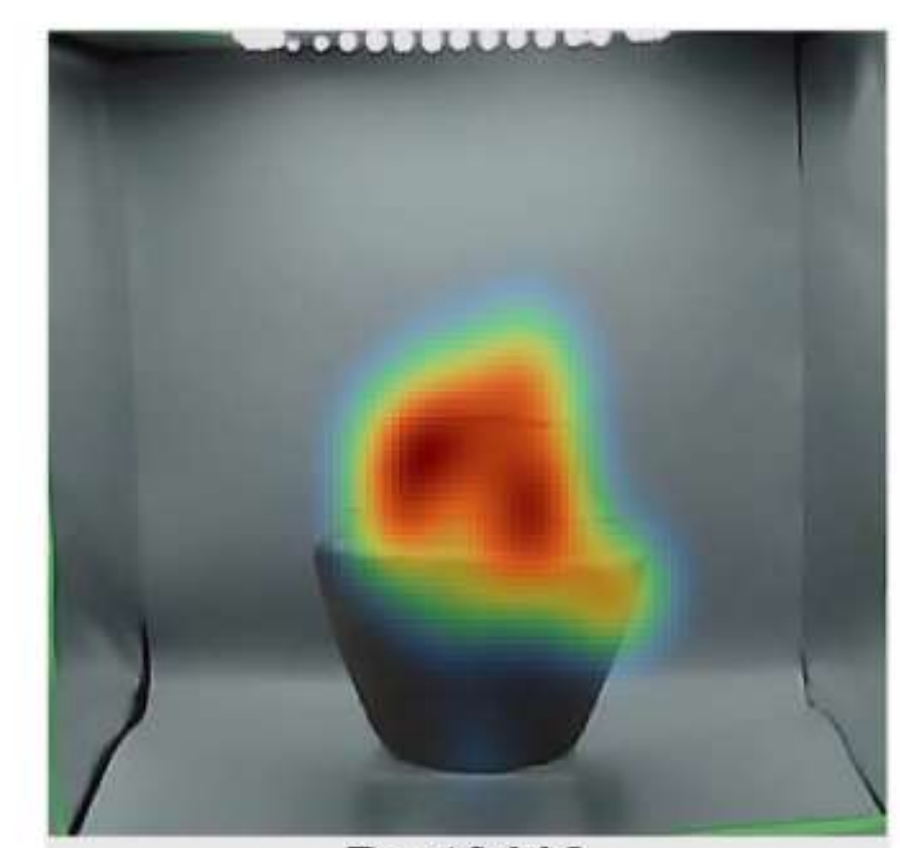
Part0113
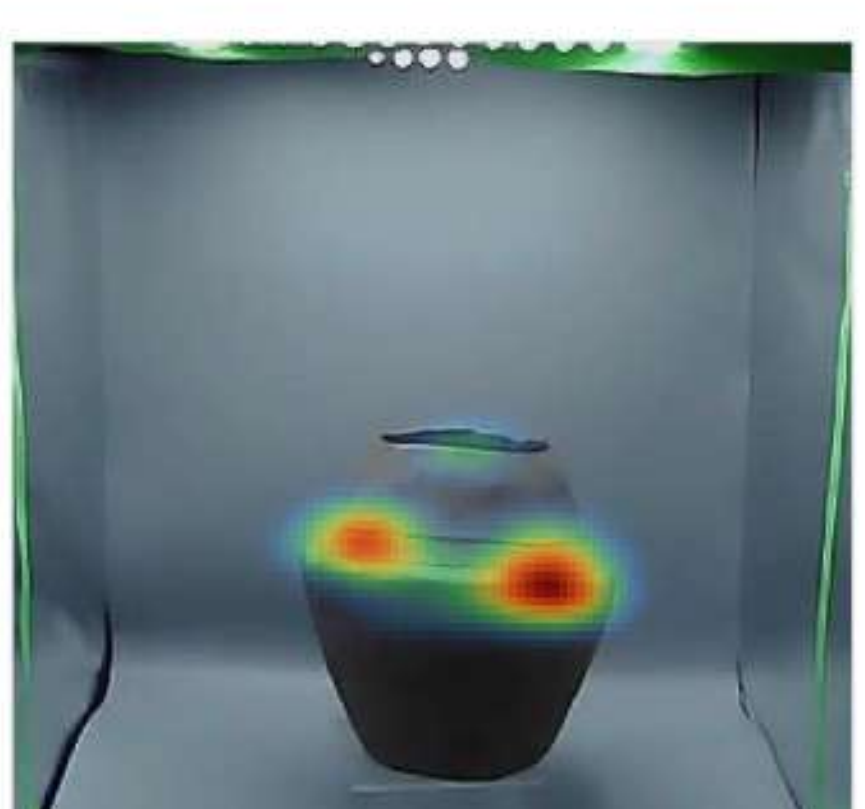
Part0114
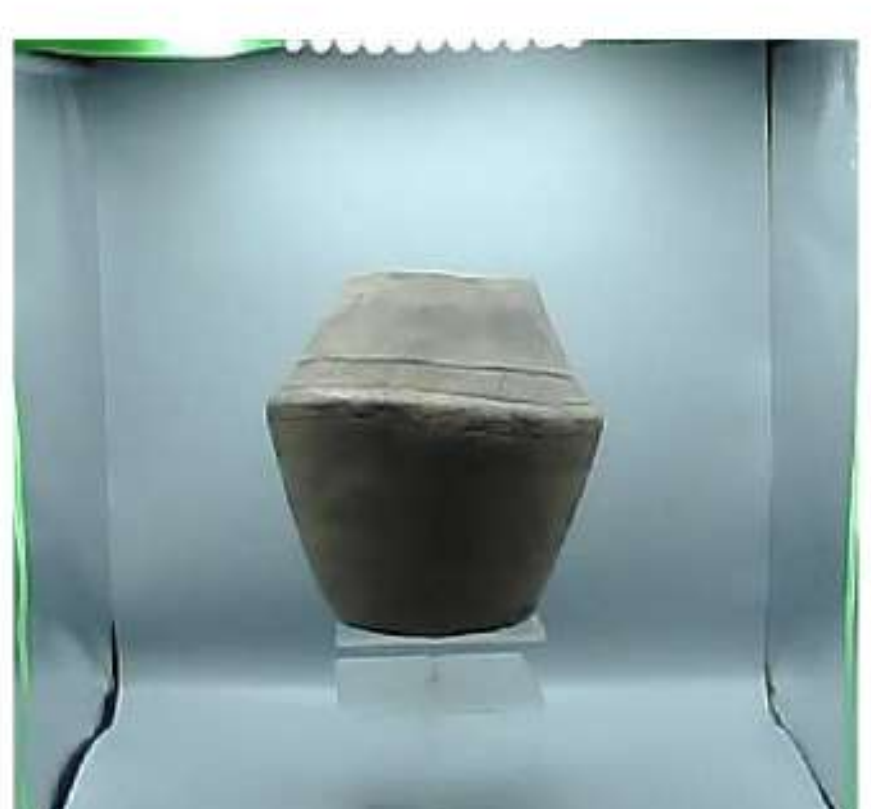
Part0115

Obj0266

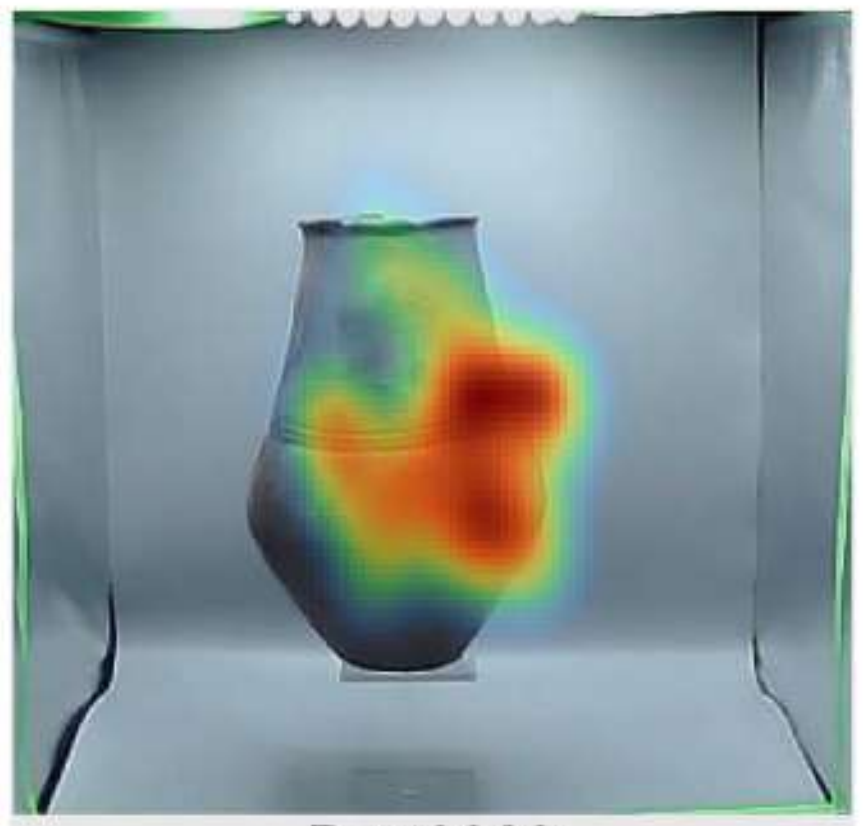
Part0089

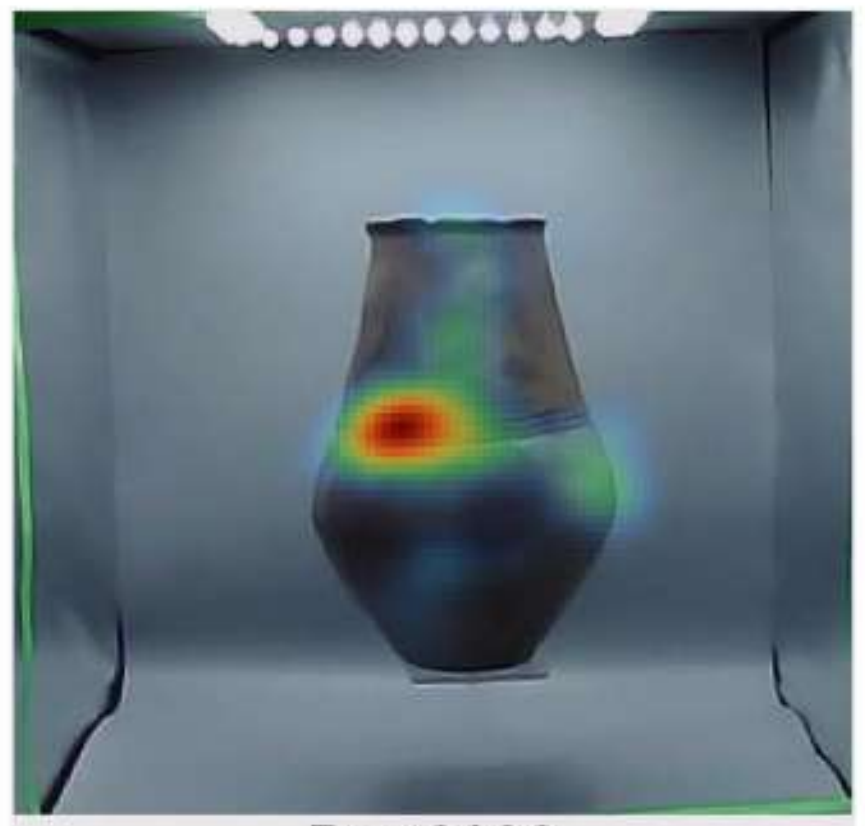
Part0090

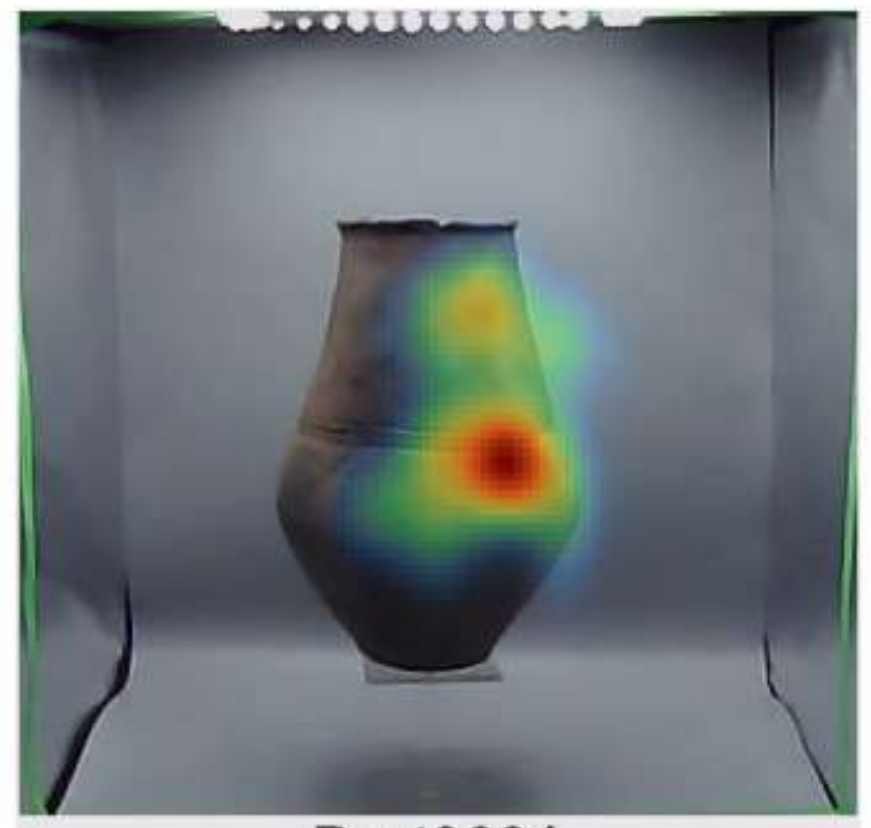
Part0091

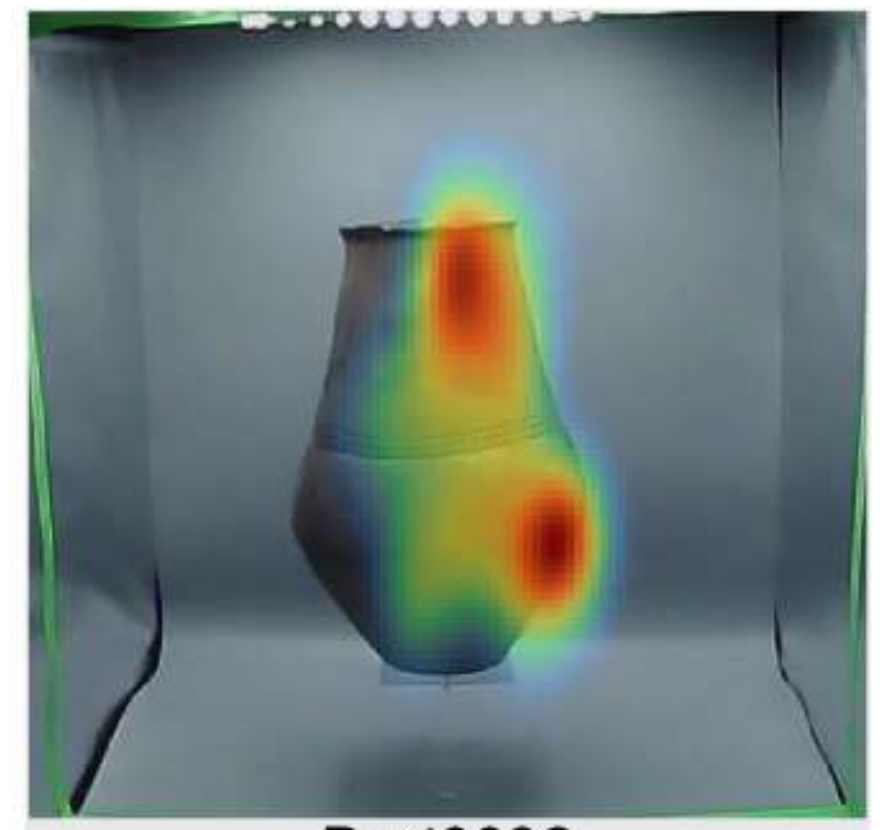
Part0092

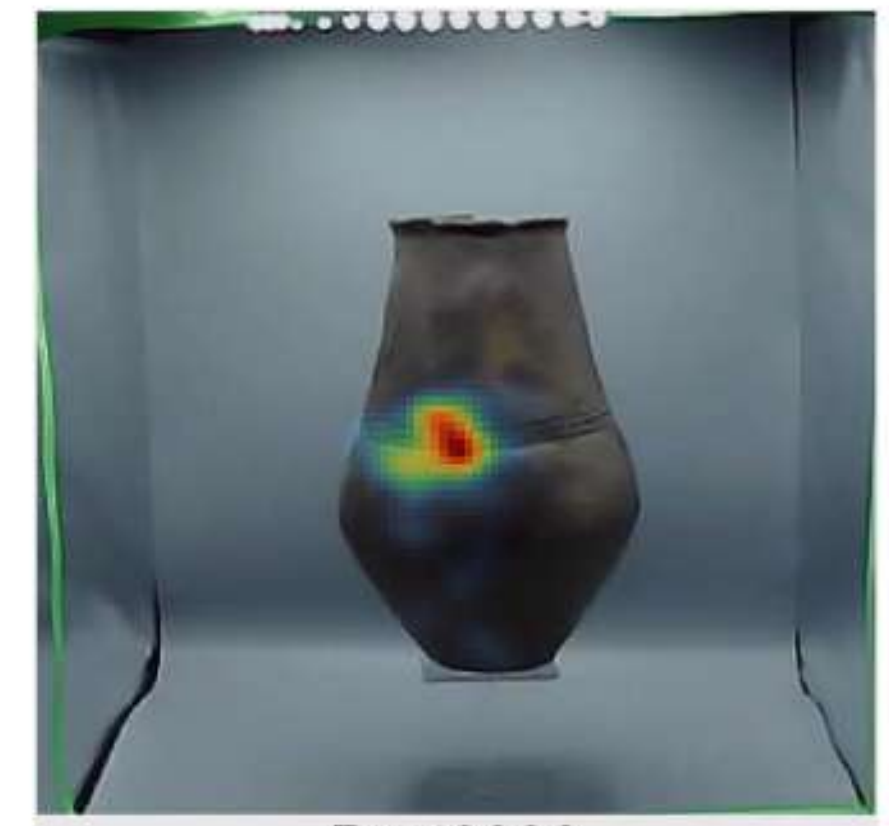
Part0093

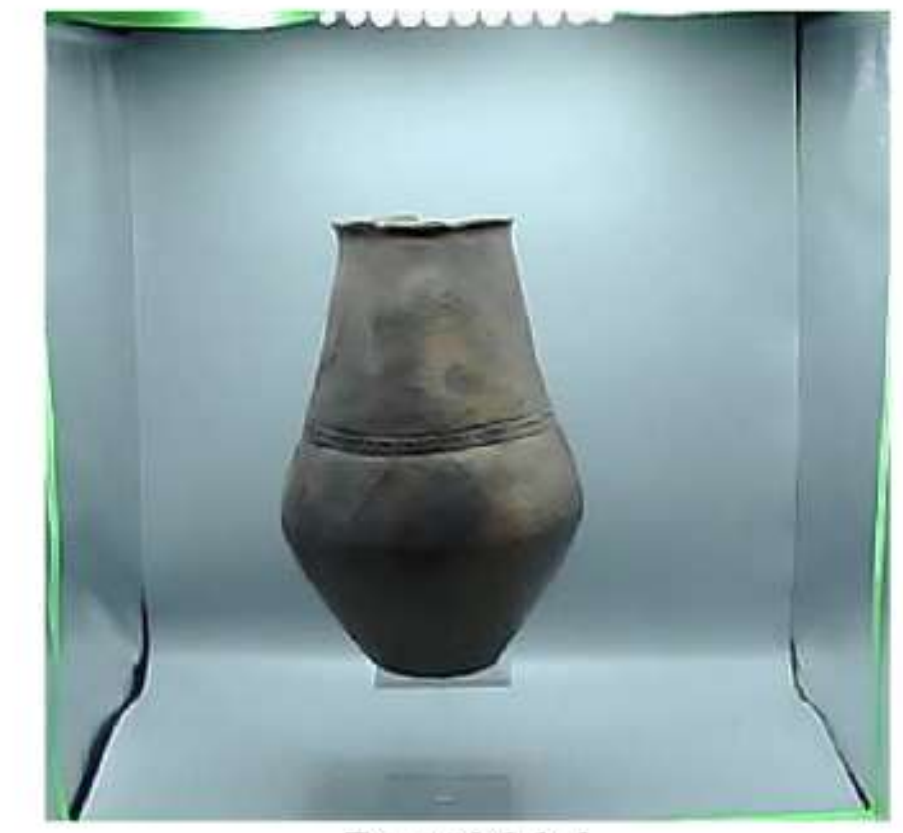
Part0094

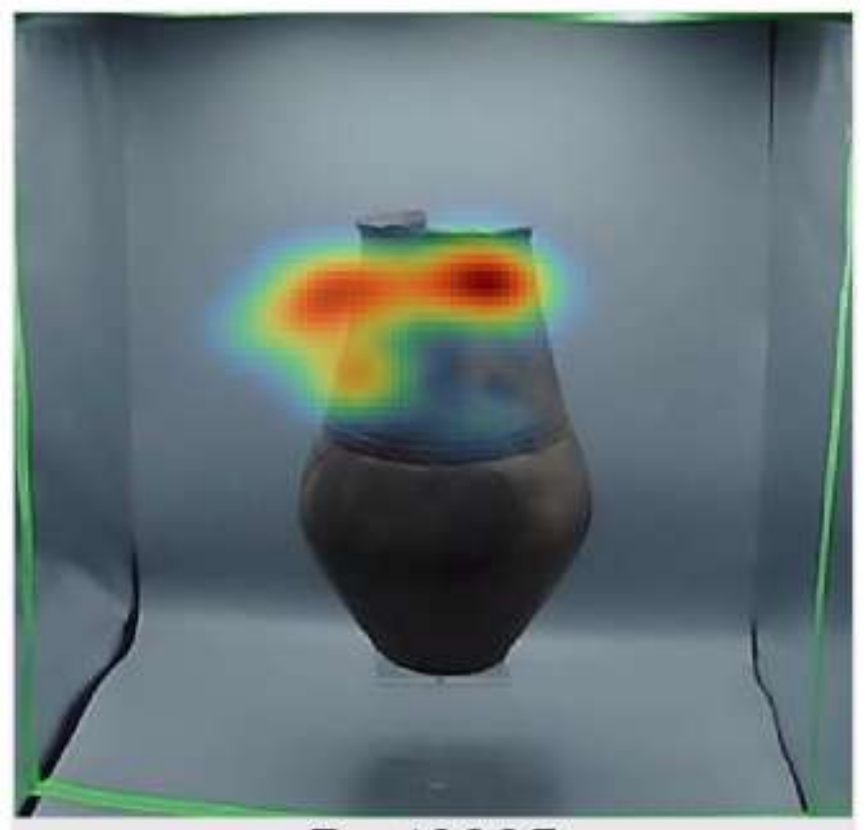
Part0095

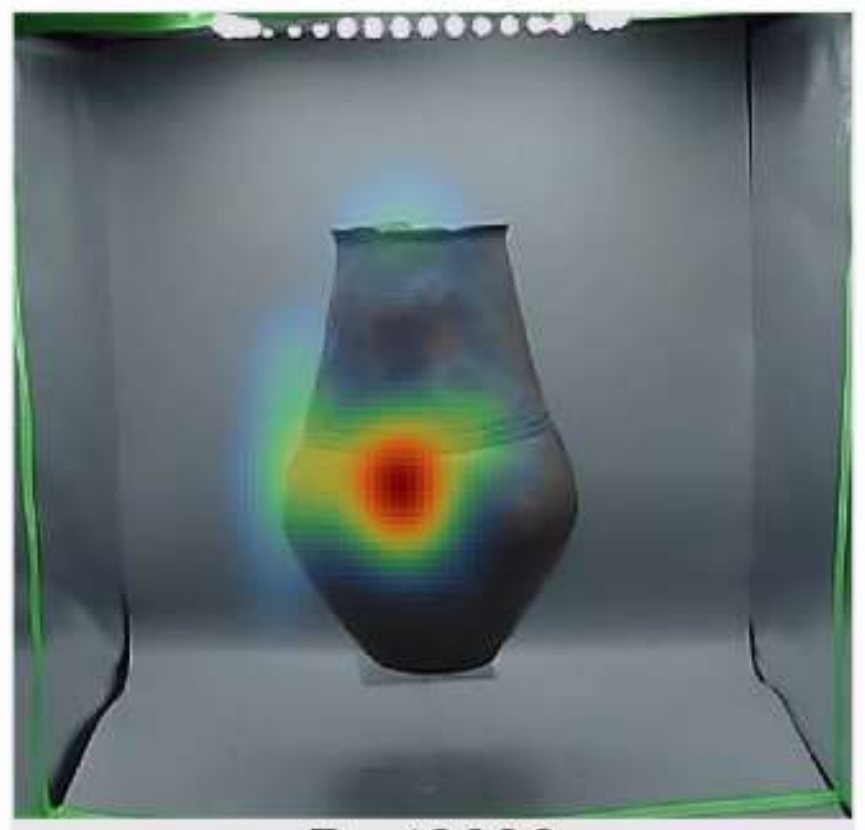
Part0096

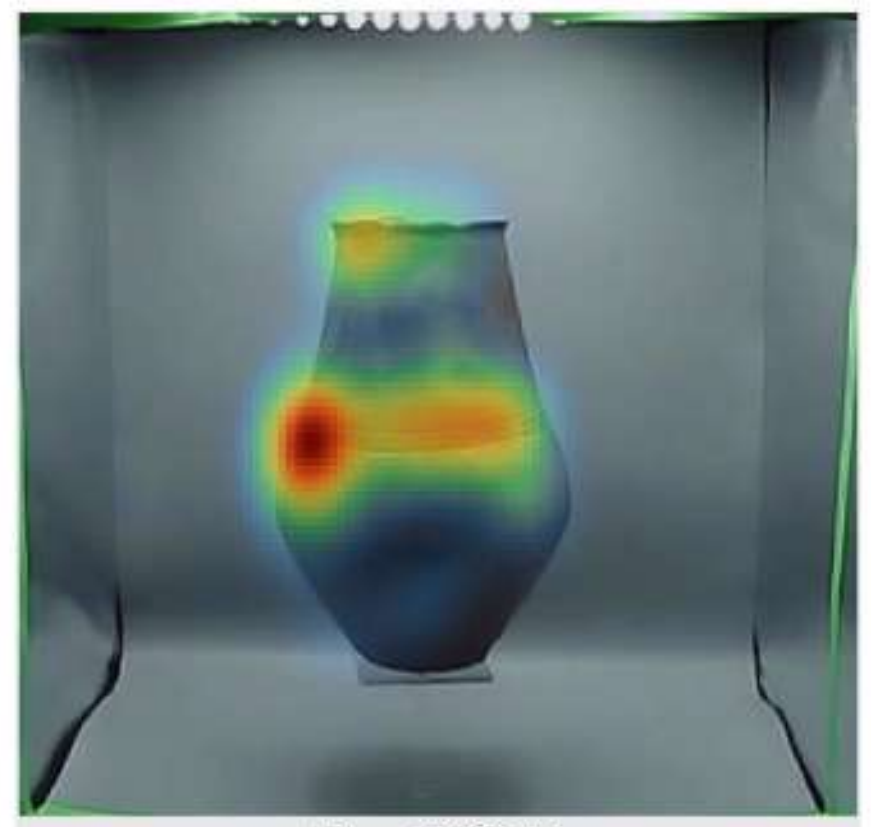
Part0097

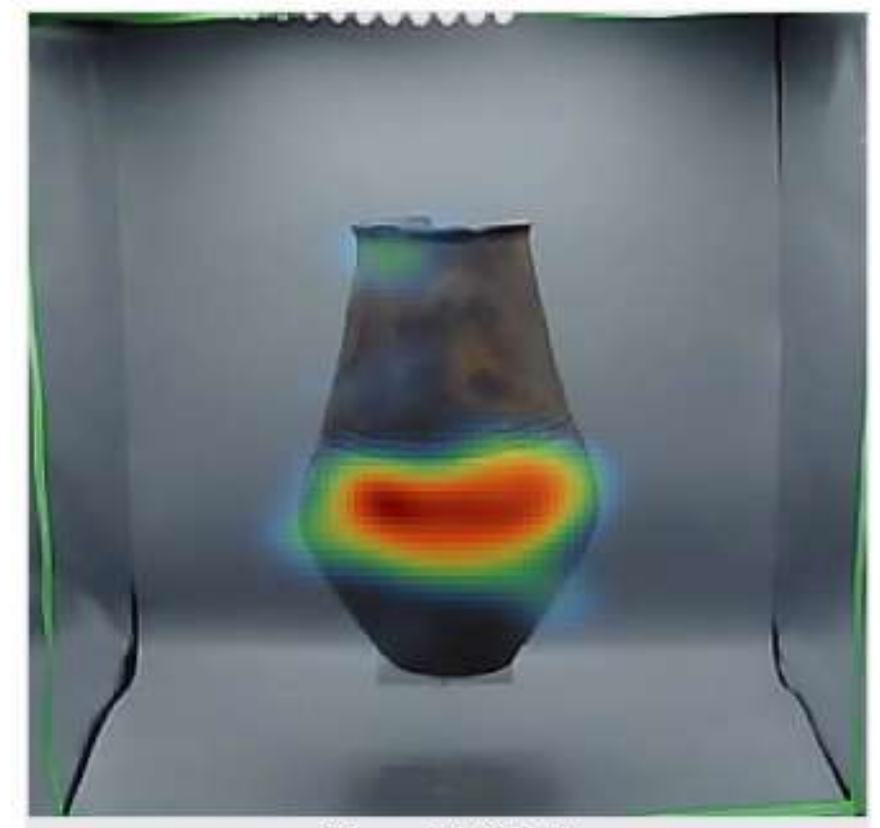
Part0098

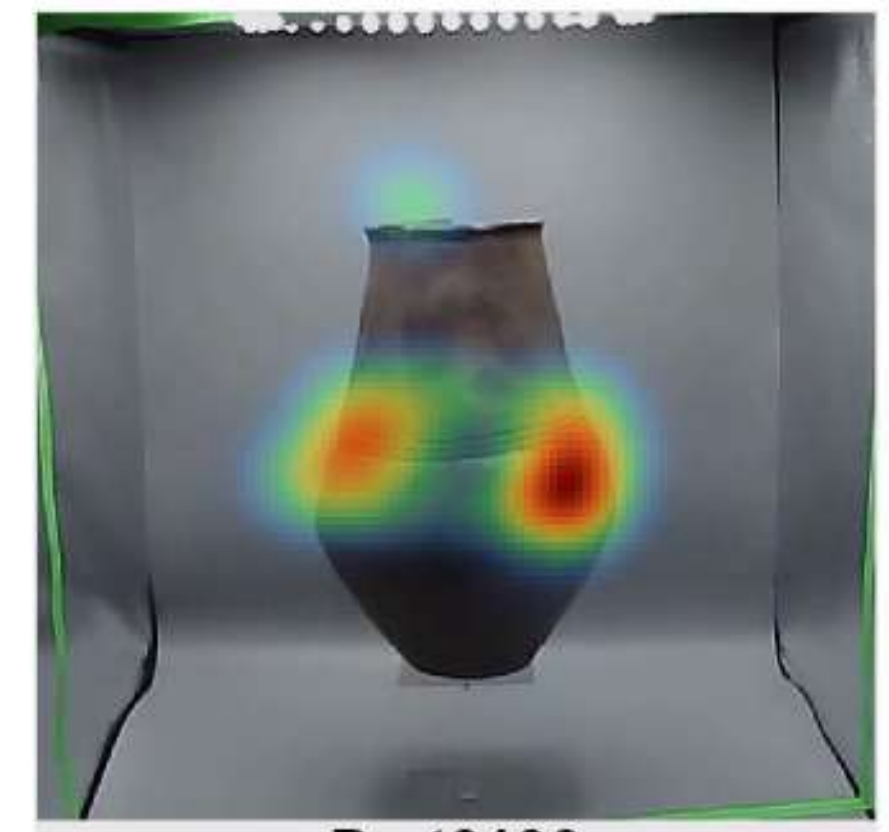
Part0100

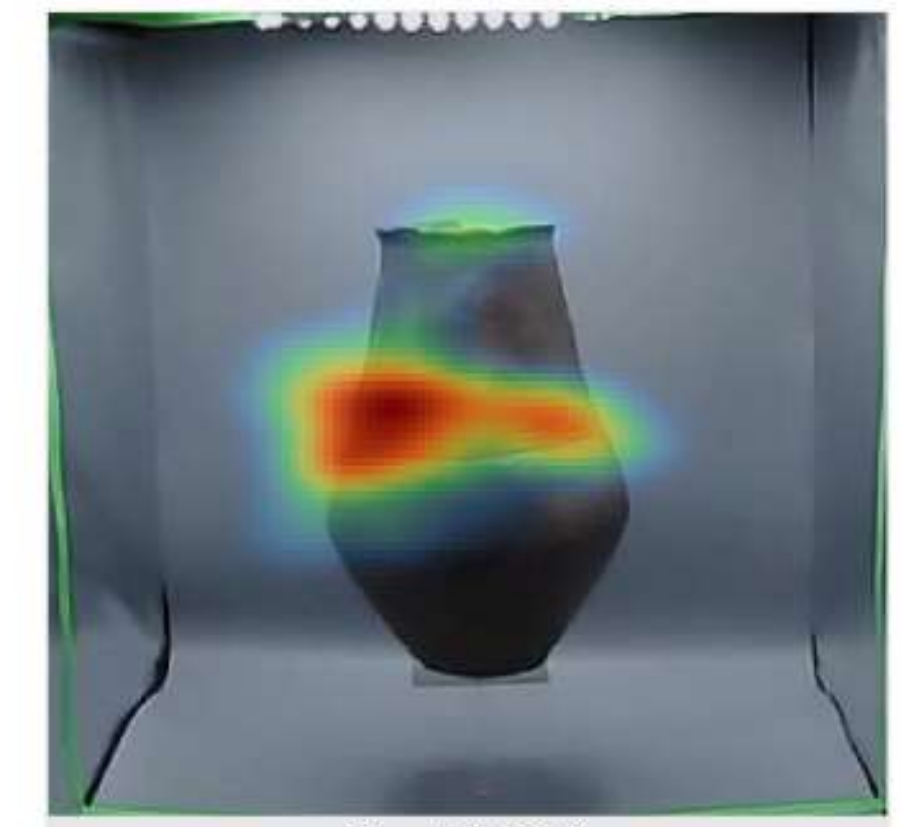
Part0101

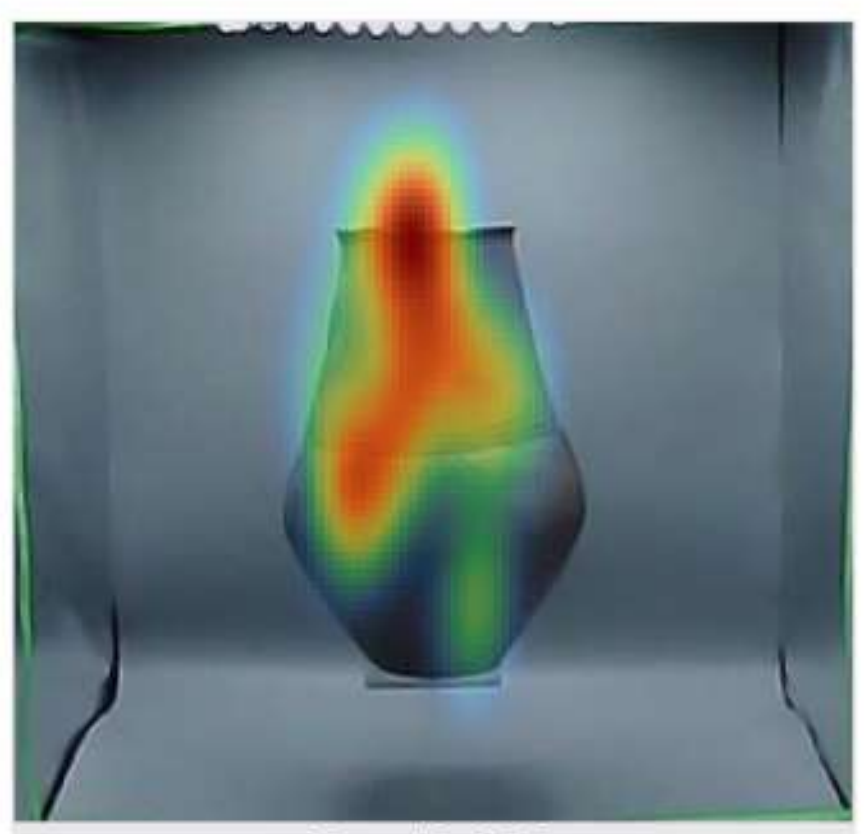
Part0102

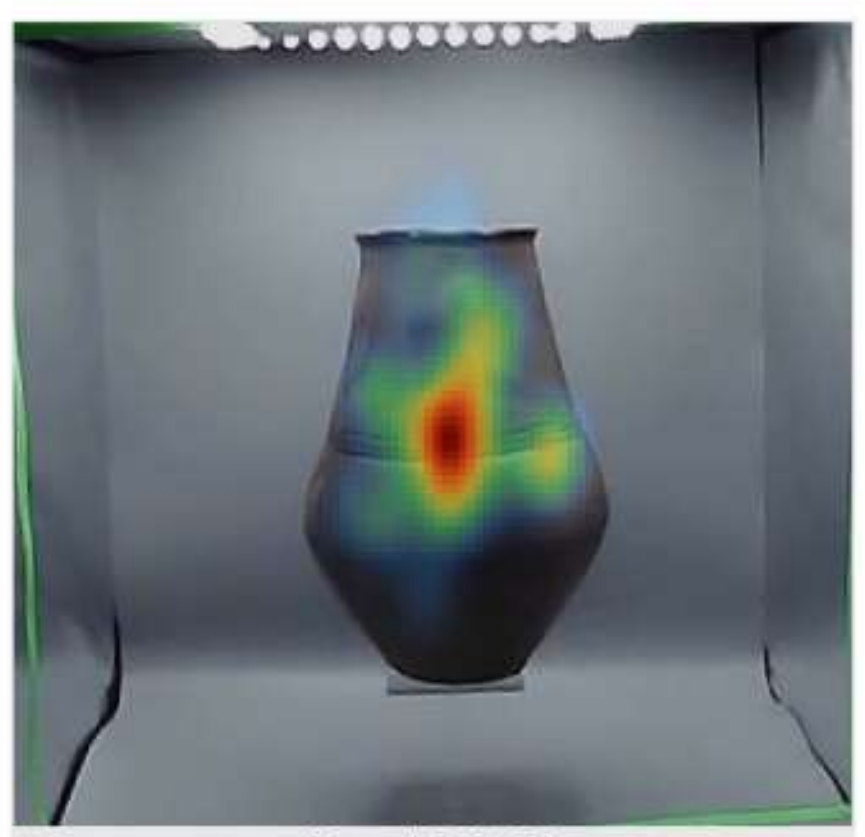
Part0103

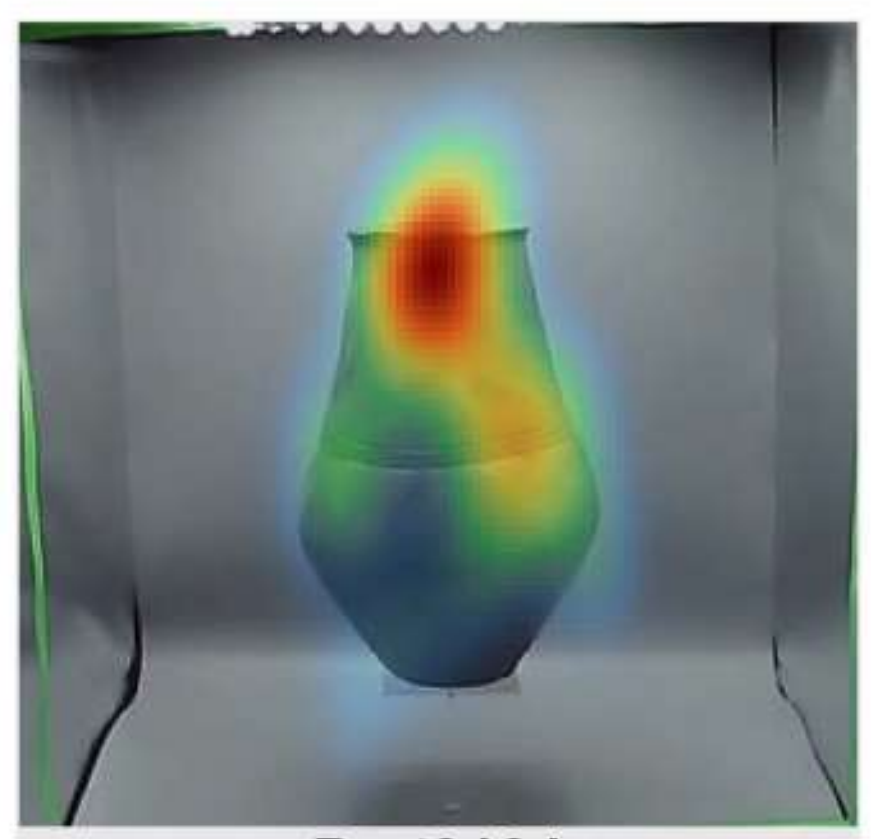
Part0104

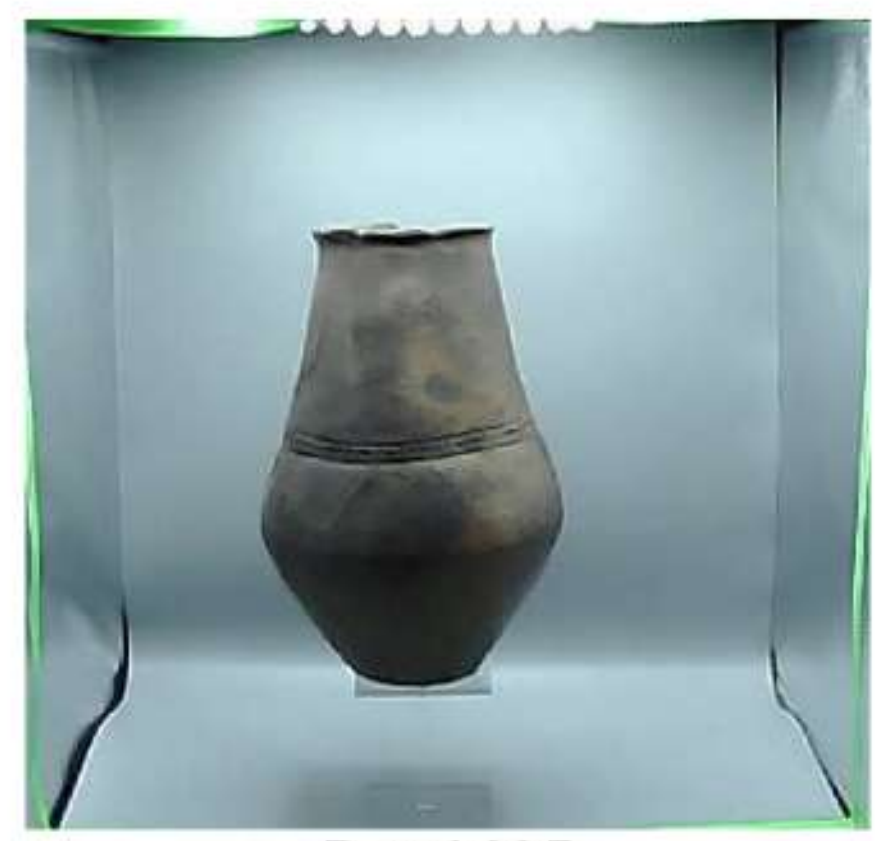
Part0105

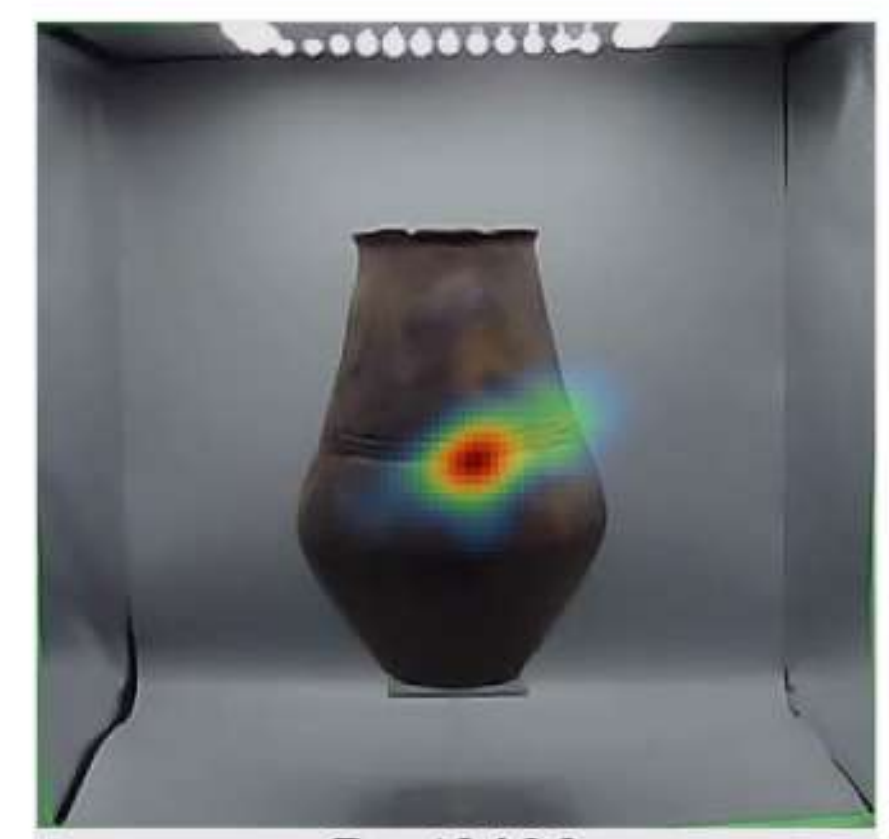
Part0106

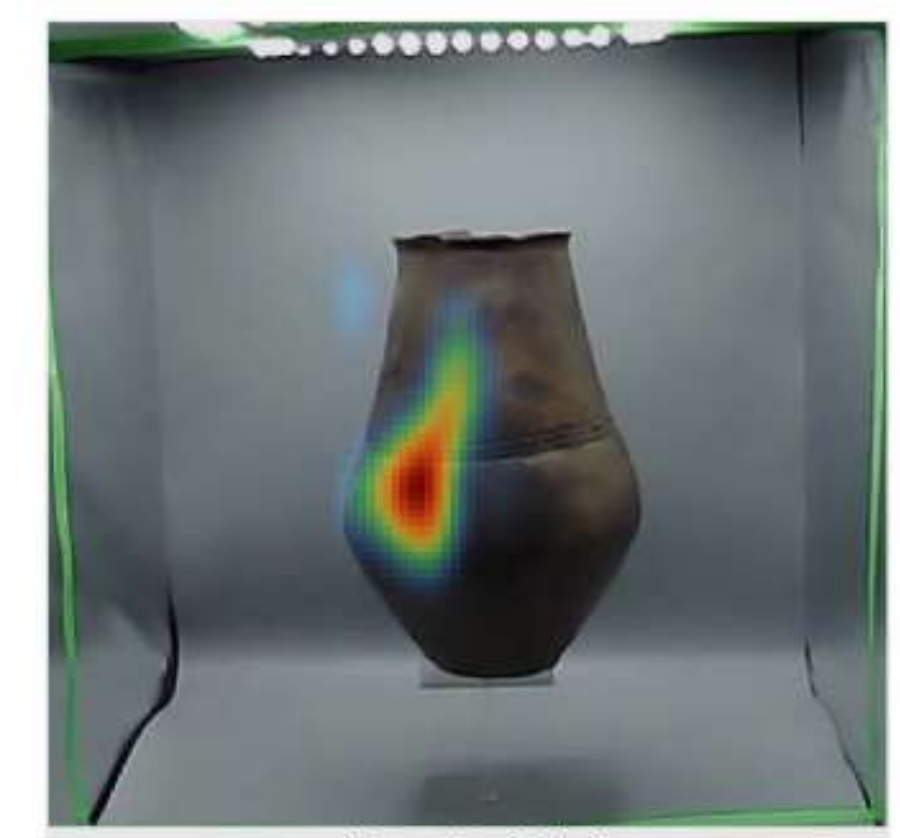
Part0107

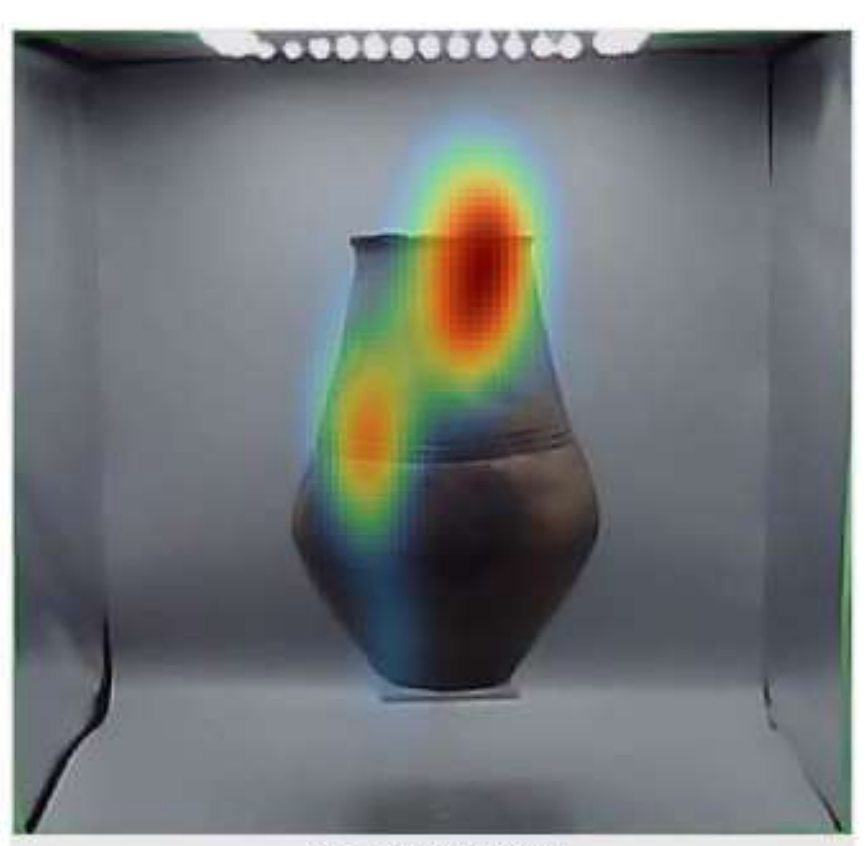
Part0108

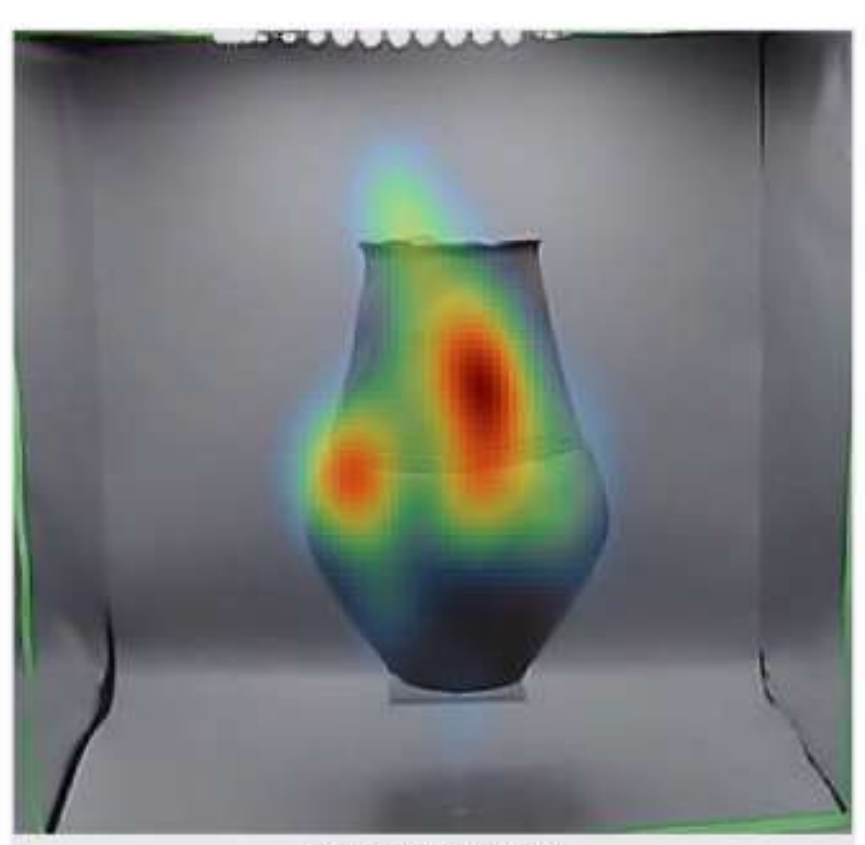
Part0109

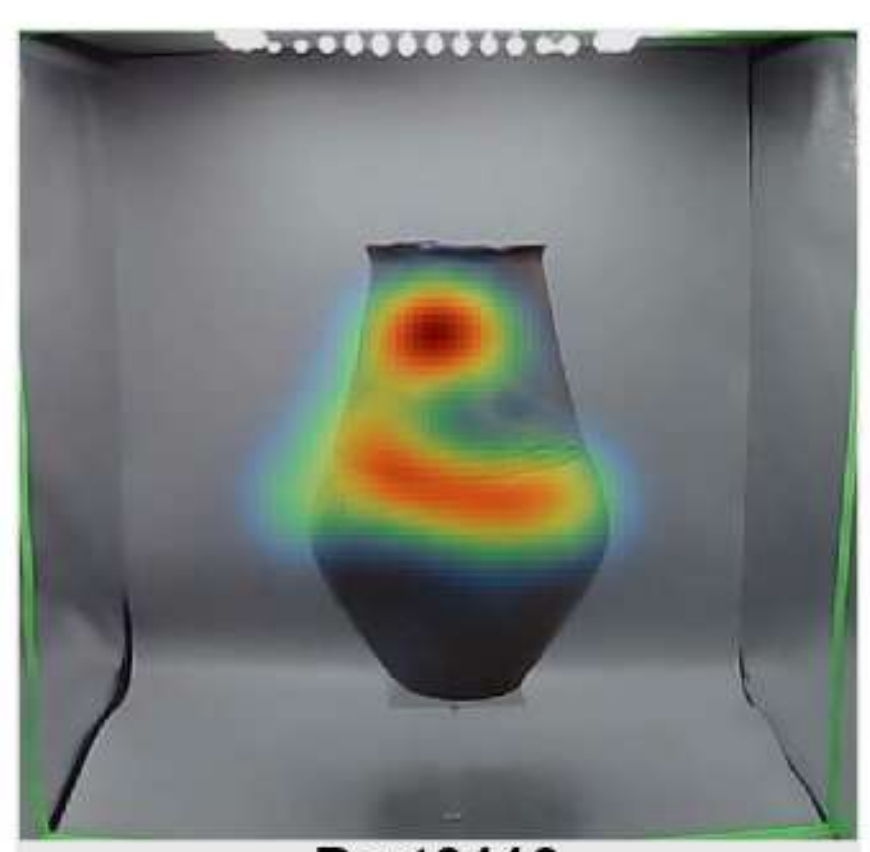
Part0110

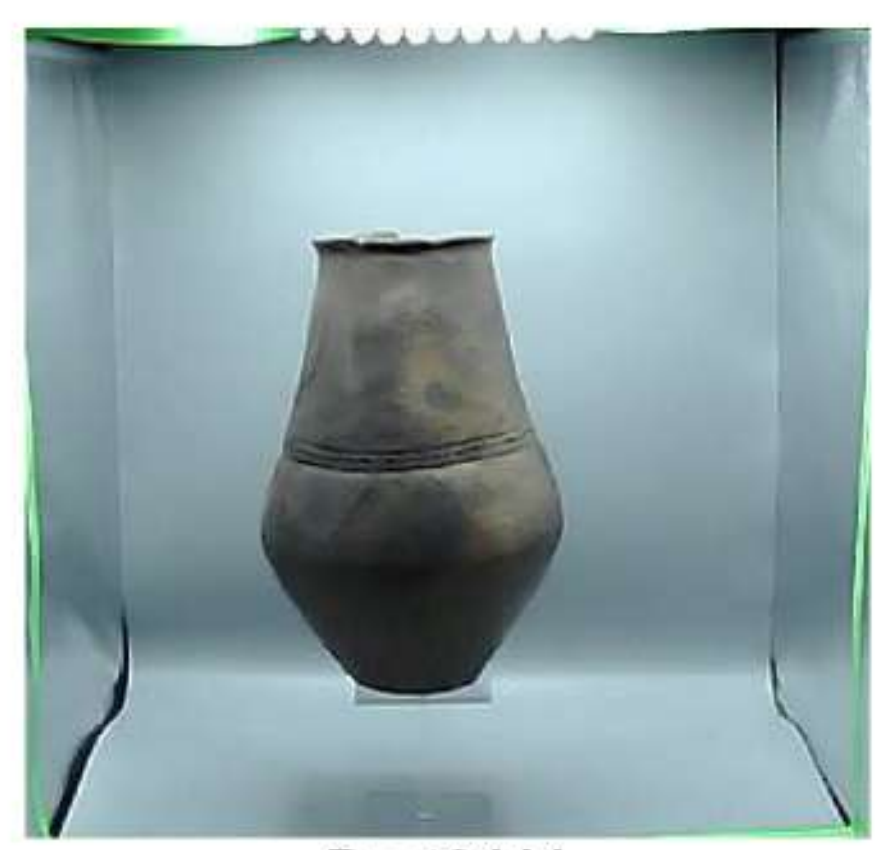
Part0111

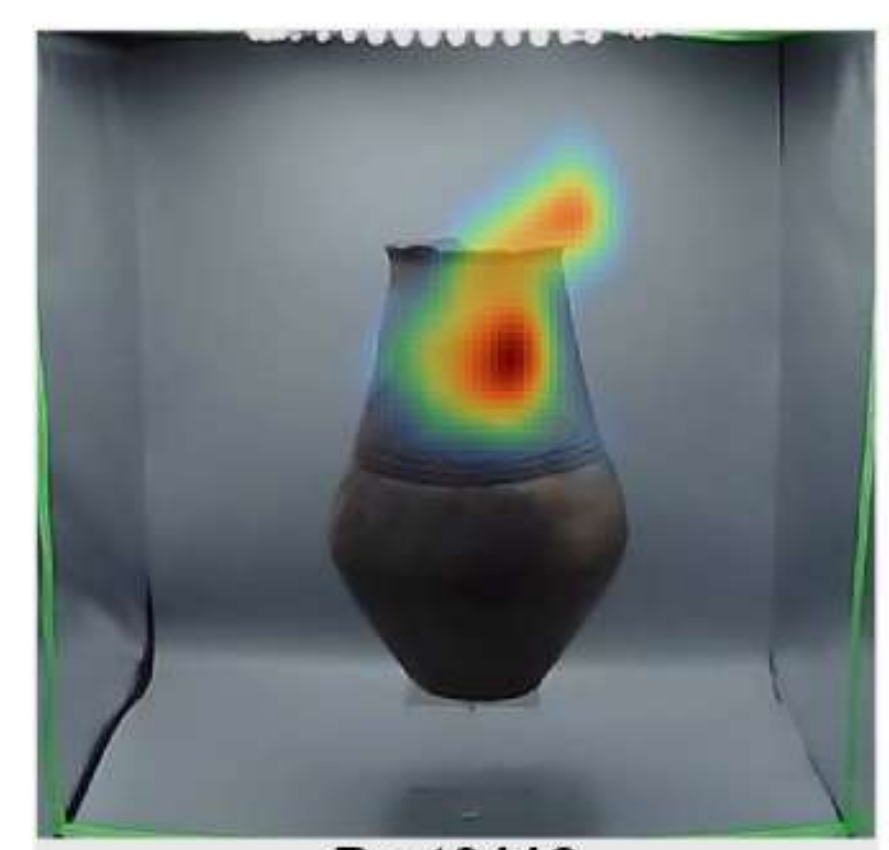
Part0112

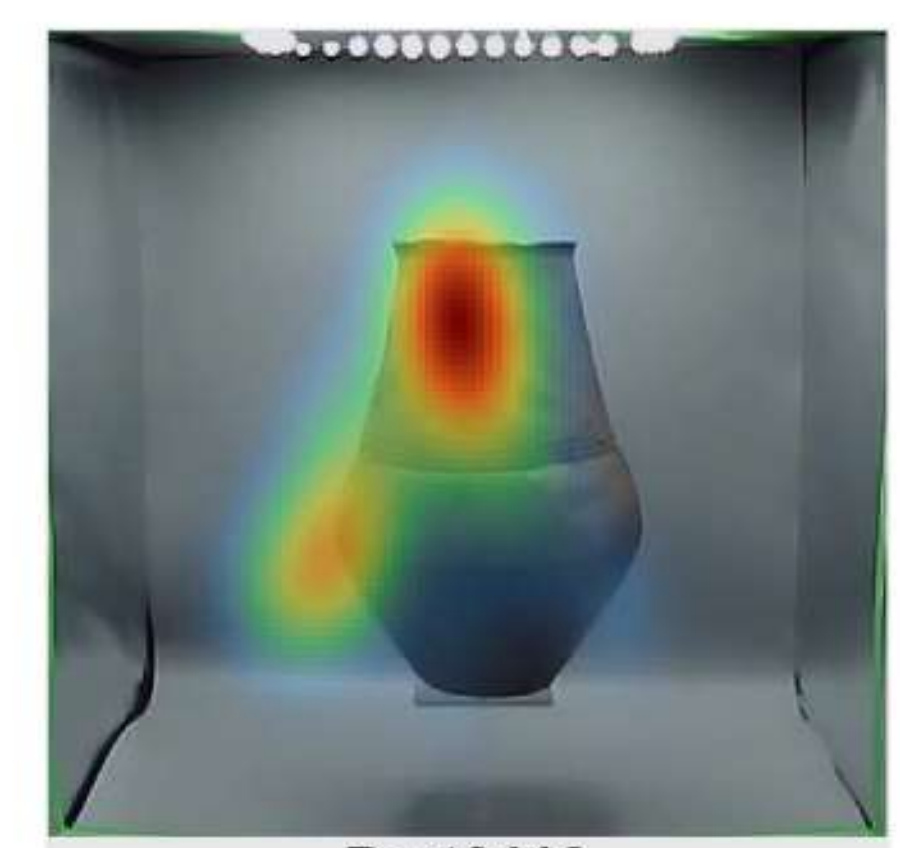
Part0113

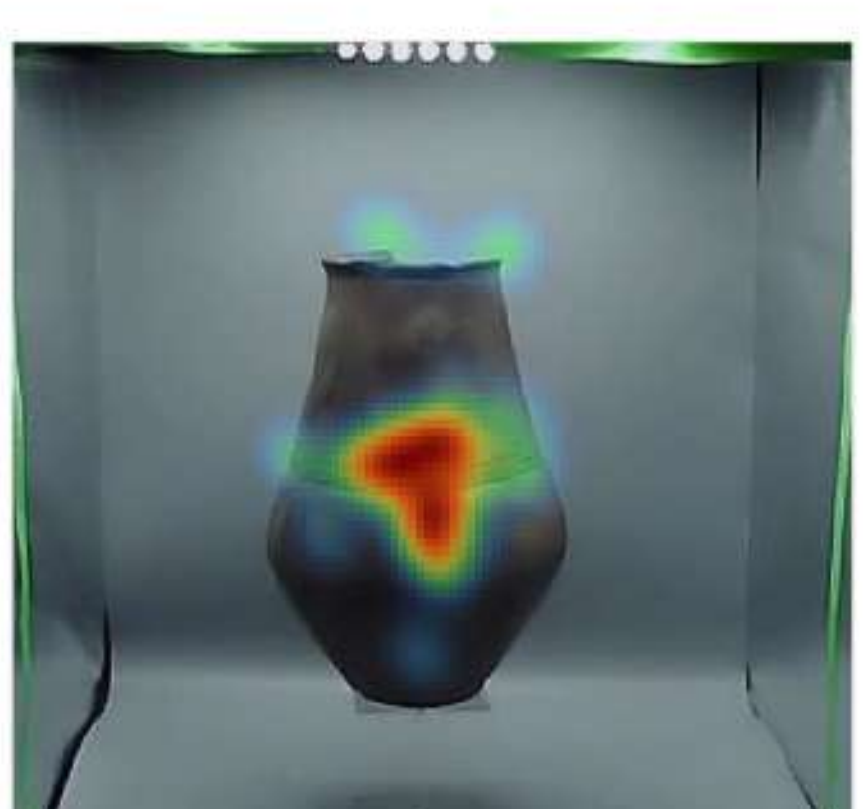
Part0114

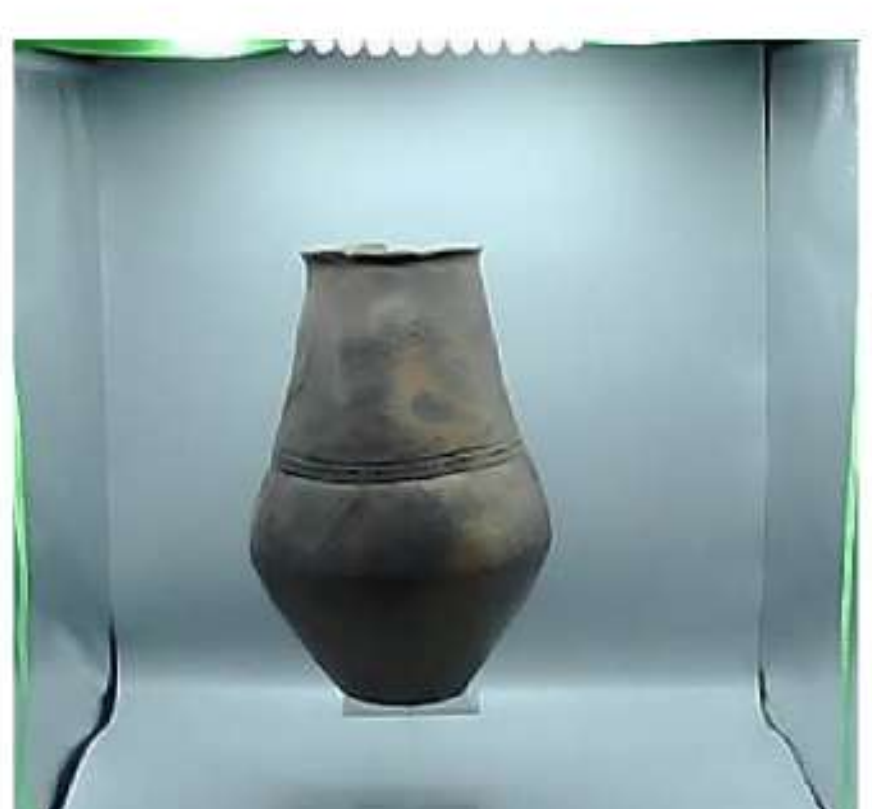
Part0115

Obj0268

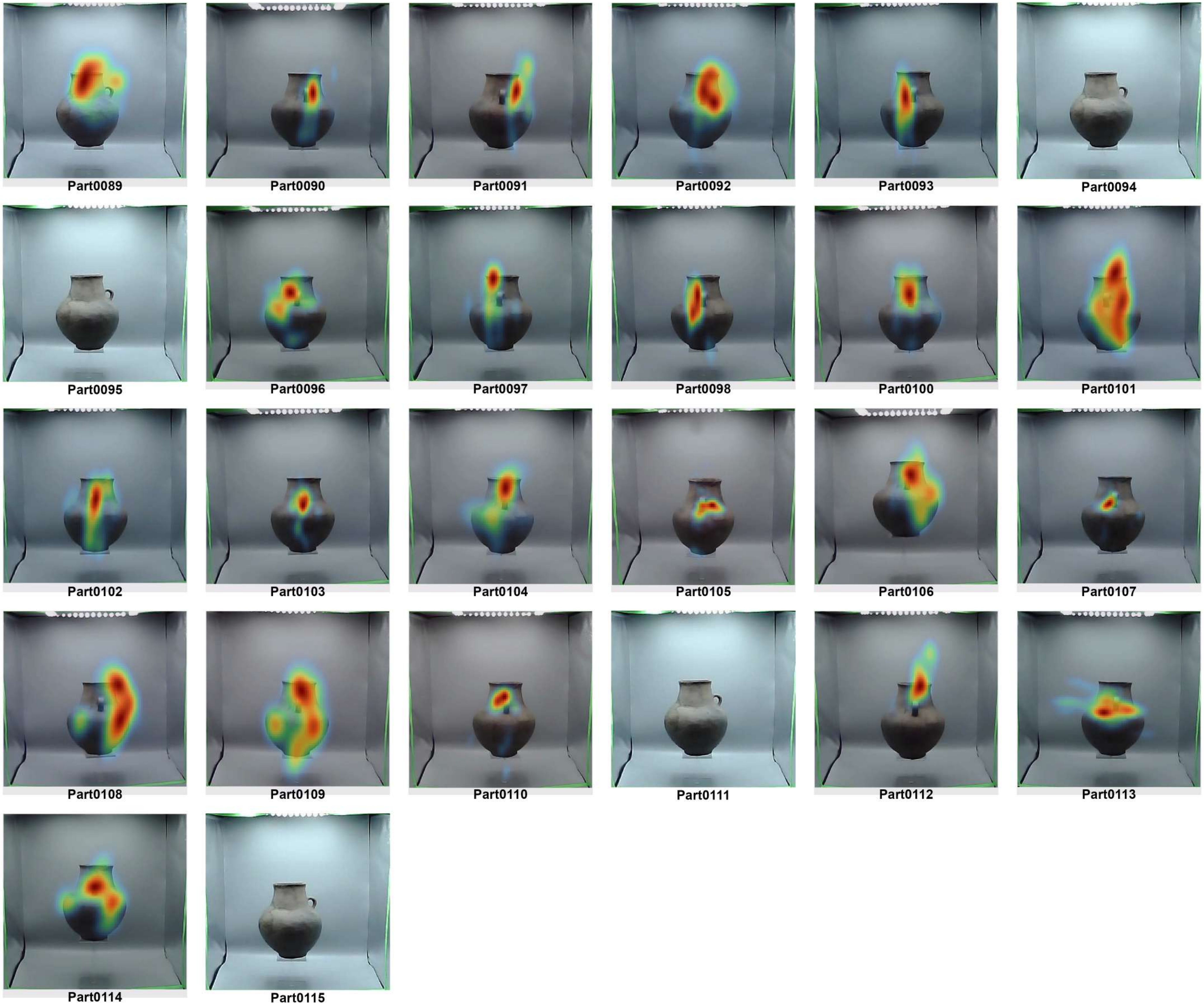
Part0089
Part0090
Part0091
Part0092
Part0093
Part0094
Part0095
Part0096
Part0097
Part0098
Part0100
Part0101
Part0102
Part0103
Part0104
Part0105
Part0106
Part0107
Part0108
Part0109
Part0110
Part0111
Part0112
Part0113
Part0114
Part0115

Obj0270

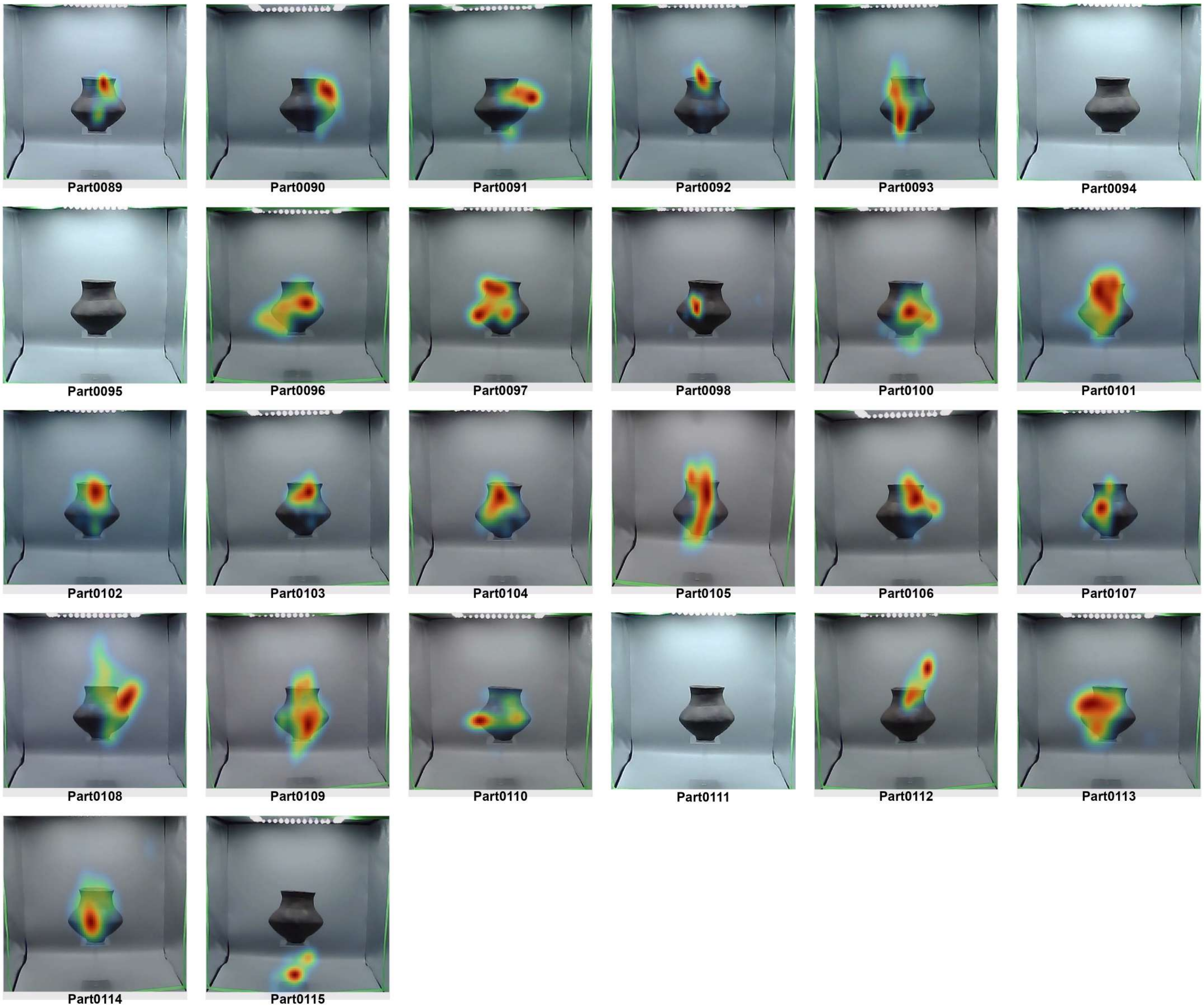
Part0089
Part0090
Part0091
Part0092
Part0093
Part0094
Part0095
Part0096
Part0097
Part0098
Part0100
Part0101
Part0102
Part0103
Part0104
Part0105
Part0106
Part0107
Part0108
Part0109
Part0110
Part0111
Part0112
Part0113
Part0114
Part0115
